# In Search of Radical Transformations from Metal Enolates. Direct Reactions of *N*‑Acyl-1,3-oxazolidin-2-ones with TEMPO Catalyzed by Copper(II) Acetate

**DOI:** 10.1021/acs.joc.5c00457

**Published:** 2025-05-27

**Authors:** Eduard Balaguer-Garcia, Marina Pérez-Palau, Cristina Bello, Anna Maria Costa, Pedro Romea, Fèlix Urpí

**Affiliations:** Department of Inorganic and Organic Chemistry, Section of Organic Chemistry, and Institut de Biomedicina de la Universitat de Barcelona, 16724 Universitat de Barcelona, Carrer Martí i Franqués 1−11, 08028 Barcelona, Catalonia, Spain

## Abstract

The direct reactions of a diverse range of *N*-acyl-1,3-oxazolidin-2-ones with TEMPO, catalyzed by copper­(II) acetate and 4,7-dimethyl-1,10-phenanthroline without the need for any base, are herein described. These reactions afford the corresponding aminoxylated derivatives with high chemoselectivity and complete regioselectivity, achieving excellent yields under mild conditions. Further treatment of the resulting imides enables access to a variety of formally protected hydroxy compounds, which can be regarded as valuable synthetic intermediates. The efficient formal synthesis of isoproterenol highlights the potential of this methodology and sets the stage for further advancements in the catalytic chemistry of metal enolates.

## Graphical abstract

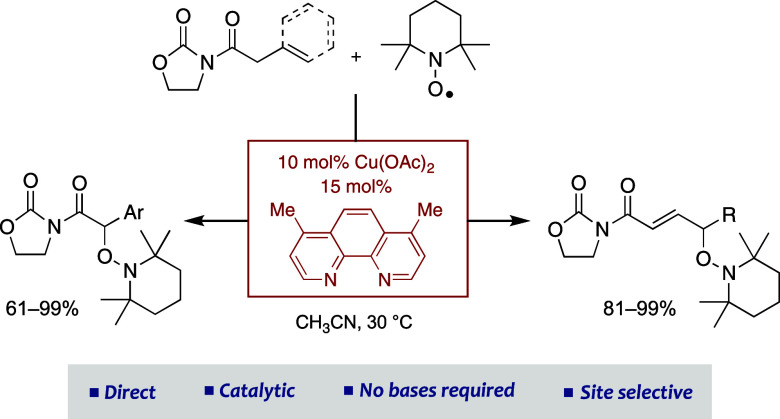

## Introduction

Metal enolates are widely regarded as some of the most versatile nucleophilic intermediates in organic synthesis, playing a pivotal role in carbon–carbon and carbon–heteroatom bond–forming reactions that proceed through polar mechanisms.^1,2^ These include alkylation, aldol and Mannich reactions, Michael additions, and Davis oxidations, among others.^3^ Largely due to their extensive utility, however, the potential of metal enolates to undergo alternative transformations through one-electron mechanisms,^4^ via ligand-to-metal charge transfer (LMCT) or valence tautomerism (Scheme 1),^5^ has been relatively overlooked, representing an underexplored area of research.

**Scheme 1:**
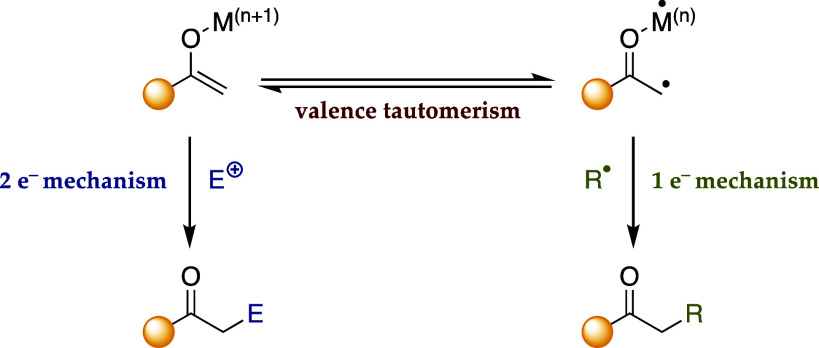
Nucleophilic- and Radical-like Reactivity of Metal Enolates

Given the unpredictable biradical nature of metal enolates, the persistent oxygen-centered radical (2,2,6,6-tetramethylpiperidin-1-yl)­oxyl (TEMPO) has proven invaluable both as a probe to elucidate radical pathways and as an oxidizing reagent.^6^ Indeed, TEMPO was instrumental in the discovery of the biradical character of titanium enolates derived from chiral imides,^7^ thereby enabling the stereoselective formation of the corresponding α-hydroxy derivatives (eq 1, Scheme 2).^8,9^ Importantly, this mechanistic insight facilitated the development of new radical-based, stereoselective alkylation reactions from such substrates.^10,11^ In turn, Yazaki and Ohshima demonstrated that the anaerobic iron–catalyzed α-oxidation of carboxylic acids with TEMPO proceeds through a radical mechanism (eq 2, Scheme 2),^12^ while Liu and Feng utilized TEMPO to confirm the radical pathway in the iron-catalyzed asymmetric alkylation of 2-acylimidazoles (eq 3, Scheme 2).^13^ Furthermore, Maulide showed that treatment of β,γ-unsaturated amides with triflic anhydride and TEMPO induces highly regioselective γ-oxidations via a radical mechanism (eq 4, Scheme 2).^14^

**Scheme 2:**
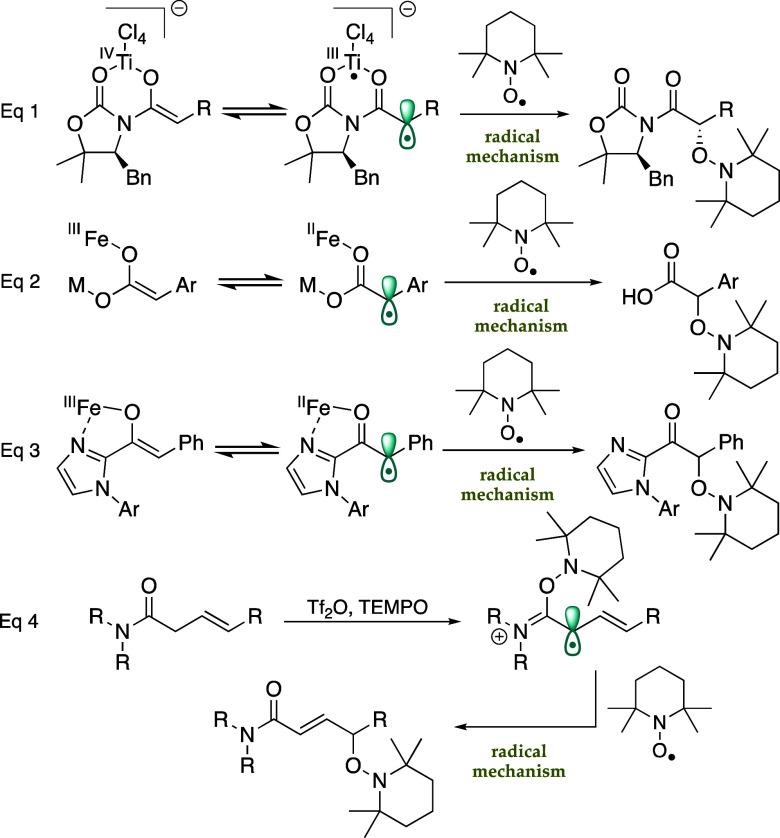
Radical-like Reactions of Metal Enolates with TEMPO

Motivated by these insights^15^ and building upon our previous work on the radical reactions of titanium enolates derived from *N*-acyl-1,3-oxazolidin-2-ones (eq 1, Scheme 2), we hypothesized that Lewis acids could catalyze direct radical-like reactions from these imides. In this context and given the lack of knowledge of the electronic character of the metal enolates from such imides, we decided to explore the reaction of model *N*-phenylacetyl-1,3-oxazolidin-2-one with TEMPO in the presence of substoichiometric amounts of first–period transition metal salts. Encouragingly, preliminary experiments demonstrated that copper­(II) acetate effectively catalyzes their oxidation by TEMPO, provided that relatively acidic substrates are used. This finding prompted further investigation into the optimal reaction conditions and the scope of the overall transformation. Herein, we present our results on the reactions of *N*-(arylacetyl) and *N*-(β,γ-unsaturated acyl) oxazolidinones with TEMPO, catalyzed by copper­(II) acetate and 4,7-dimethyl-1,10-phenanthroline leading to the selective formation of α- and γ-oxygenated derivatives respectively in high yields (Scheme 3).

**Scheme 3:**
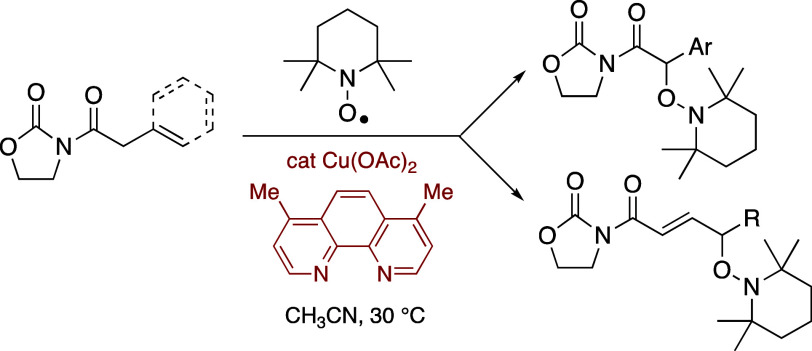
Current Work. Reaction of Imides with TEMPO Catalyzed by Cu­(OAc)_2_ and 4,7-Dimethyl-1,10-phenanthroline

## Results and Discussion

To identify the most appropriate experimental conditions, we first investigated the reaction of *N*-phenylacetyl-1,3-oxazolidin-2-one (**1a**) with TEMPO in the presence of copper­(II) acetate as the catalyst (Table 1). Thus, a series of solvents and temperatures were systematically evaluated to optimize the conversion of **1a** using two equivalents of TEMPO and one equivalent of triethylamine at 70 °C. The results, summarized in Table 1, indicated that the choice of solvent was critical. A protic solvent, such as isopropanol, led to a very poor yield of the Cα oxidized derivative **2a** (entry 1 in Table 1), while nonprotic solvents like THF and DCE significantly improved the conversion of **1a** (entries 2 and 3 in Table 1). Among the solvents tested, polar aprotic solvents, including DMSO, DMF, ethyl acetate, or acetonitrile gave the best results (entries 4–7 in Table 1). Notably, acetonitrile provided a significant 45% conversion, which was increased to completion when three equivalents of TEMPO were used (entry 8 in Table 1). Temperature also had a significant impact on the reaction outcome. Indeed, the conversion of **1a** was less than 30% at room temperature but raised to 60% at 30 °C (entries 8–12 in Table 1), which proved that mild heating was beneficial for the reaction. At this stage, we speculated that ligands known to coordinate to copper­(II) might enhance the reaction outcome. To test this hypothesis, we evaluated the effect of 15 mol % of various ligands, including 1,4-diazabicyclo[2.2.2]­octane (DABCO, **L1** in Table 1), 2,2′-bipyridine (bipy, **L2** in Table 1), and 1,10-phenanthroline (phen, **L3** in Table 1) at 30 °C for 16 h. These reactions were conducted in the presence of a bulkier tertiary amine such as diisopropylethylamine, to minimize potential coordination with the metal. The presence of DABCO (**L1**) was found to be detrimental and **2a** was obtained with a poor 15% of conversion (compare entries 11 and 13). In contrast, bipyridine (**L2**) and especially 1,10-phenanthroline (**L3**) improved the conversion up to 88% (compare entry 11 with entries 14–15). Surprisingly, we observed that a tertiary amine such as diisopropylethylamine, commonly used to deprotonate carbonyl substrates, was unnecessary. This suggested that copper­(II) acetate could effectively catalyze the TEMPO–oxidation without the need for an external base to produce **2a** in up to 77% by simple addition of 15 mol % of 1,10-phenanthroline (**L3**). Varying the equivalents of copper­(II) acetate and ligand **L3** led to the anticipated changes in conversion (compare entries 17–19 in Table 1). Finally, extending the reaction time to 24 h resulted in nearly quantitative conversion (entry 20 in Table 1).

**Table 1:**
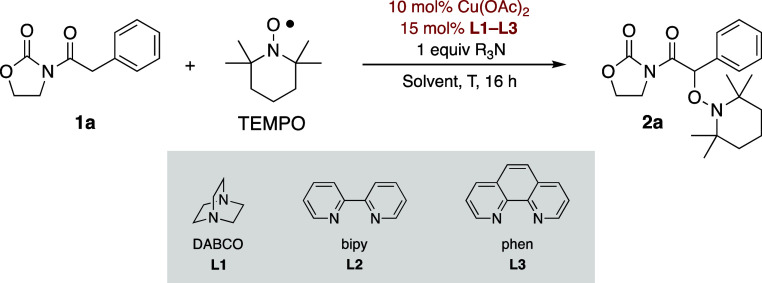
Preliminary Studies

entry	solvent	TEMPO (equiv)	R_3_N	ligand	temperature (°C)	conversion (%)
1	*i*-PrOH	2	Et_3_N		70	<10
2	THF	2	Et_3_N		70	21
3	DCE	2	Et_3_N		70	23
4	DMSO	2	Et_3_N		70	34
5	DMF	2	Et_3_N		70	31
6	EtOAc	2	Et_3_N		70	35
7	CH_3_CN	2	Et_3_N		70	45^a^
8	CH_3_CN	3	Et_3_N		70	>95
9	CH_3_CN	3	Et_3_N		50	>95
10	CH_3_CN	3	Et_3_N		40	75
11	CH_3_CN	3	Et_3_N		30	60
12	CH_3_CN	3	Et_3_N		20	30
13	CH_3_CN	3	*i*-Pr_2_NEt	**L1**	30	15
14	CH_3_CN	3	*i*-Pr_2_NEt	**L2**	30	75
15	CH_3_CN	3	*i*-Pr_2_NEt	**L3**	30	88
16	CH_3_CN	3		**L2**	30	59
17	CH_3_CN	3		**L3**	30	77
18^b^	CH_3_CN	3		**L3**	30	65
19^c^	CH_3_CN	3		**L3**	30	85
20^d^	CH_3_CN	3		**L3**	30	95

a TEMPO was completely consumed.

b 5 mol % of Cu­(OAc)_2_ and 7.5 mol % of **L3** were used.

c 15 mol % of Cu­(OAc)_2_ and 22.5 mol % of **L3** were used.

d Reaction time of 24 h.

Considering these findings, we explored whether other ligands derived from aromatic amines could also enhance the efficiency of such a straightforward transformation. Aiming to identify the most suitable ligand, we assessed the influence of substoichiometric amounts (15 mol %) of **L3–L11** on the oxidation of **1a** with two equivalents of TEMPO at 30 °C for a short reaction time (6 h). Bipyridine **L4** and phenanthroline **L5**, both containing strong electron–withdrawing groups, were found to be ineffective (entries 2 and 3 in Table 2). In turn, biquinoline **L6** yielded worse results than phenanthroline **L3** (entries 1 and 4 in Table 2), while 2,9-dimethyl-1,10-phenanthroline **L7** led to a reduced yield, likely due to the steric hindrance from the two methyl groups (entry 5 in Table 2). Interestingly, 4,7-disubstituted-1,10-phenanthrolines **L8–L11**, which feature groups with varying electronic properties, gave a wide range of conversion rates (entries 6–10 in Table 2). The methoxy substituted phenanthroline **L9** proved particularly ineffective, affording only a modest 15% yield after 24 h (entries 7 and 8 in Table 2), likely due to the poor solubility of the resulting copper complex. In contrast, 4,7-dimethyl-1,10-phenanthroline **L11** emerged as the most promising ligand, delivering **2a** in 47% yield (entry 10 in Table 2). Finally, extending the reaction time to 24 h gave the desired adduct **2a** in an outstanding 99% yield (entry 11 in Table 2).

**Table 2:**
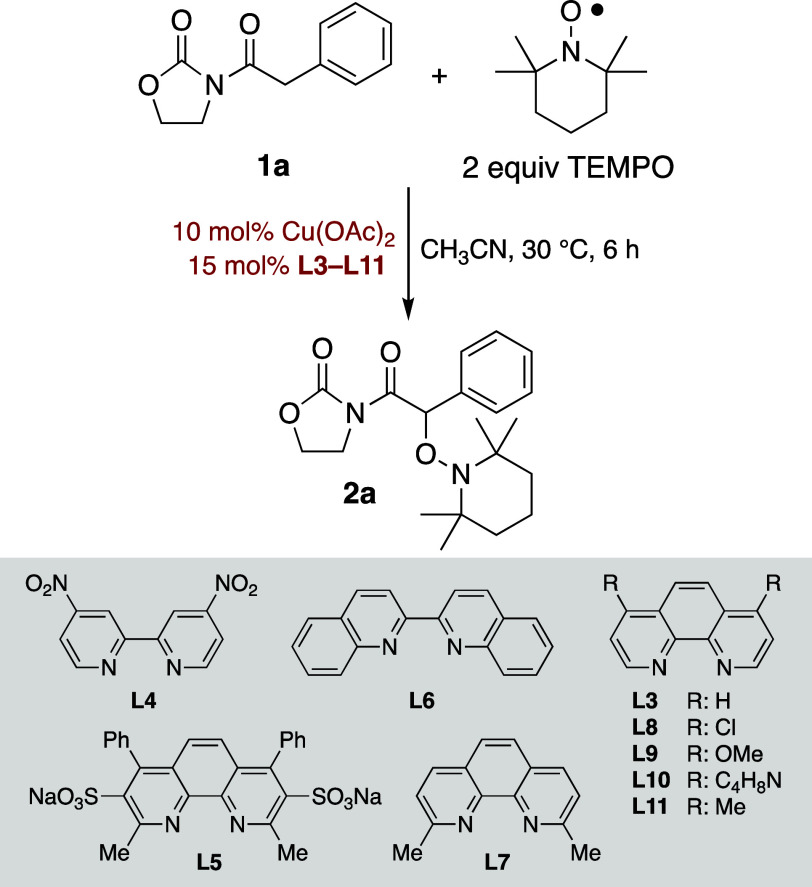
Influence of the Ligands

entry	ligand	yield (%)
1	**L3**	38
2	**L4**	—
3	**L5**	—
4	**L6**	19
5	**L7**	21
6	**L8**	16
7	**L9**	6
8^a^	**L9**	15
9	**L10**	26
10	**L11**	47
11^a^	**L11**	99

a Reaction time of 24 h.

Having established the optimal experimental conditions to achieve a quantitative yield with model imide **1a**, we next investigated the scope of the reaction. This involved examining the reaction of *N*-arylacetyl-1,3-oxazolidin-2-ones **1a–1r** with TEMPO catalyzed by Cu­(OAc)_2_/**L11**.

The results summarized in Table 3 demonstrate that the reaction exhibits a broad substrate scope, showing tolerance for a variety of aryl groups. Substrates possessing electron-donating groups, such as ethers (OBn or OMe, **1b–1e** in Table 3) and alkyl groups (Me, **1f–1h** in Table 3), consistently afforded the expected adducts (**2b–2h**) in yields of up to 99%, regardless of the relative position of the substituent. Notably, *ortho*-methoxy and *ortho*-methyl imides **1e** and **1h**, respectively, gave the lowest yields of the corresponding derivatives (**2e** and **2h**, 61% and 67% respectively), likely due to significant steric hindrance at the ortho position. Otherwise, electron-withdrawing groups had minimal impact on the reaction outcome, with adducts **2i**–**2k** being isolated in excellent yields. Finally, more complex oxygenated (**1l**–**1n**) and nitrogenated (**1o–1p**) imides also performed well, providing the corresponding adducts **2l**–**2p** in high yields. The only exceptions were imides **1q** and **1r**, which contain an unprotected alcohol or amine, respectively, in the aromatic ring, as they turned out to be completely unreactive.

**Table 3:**
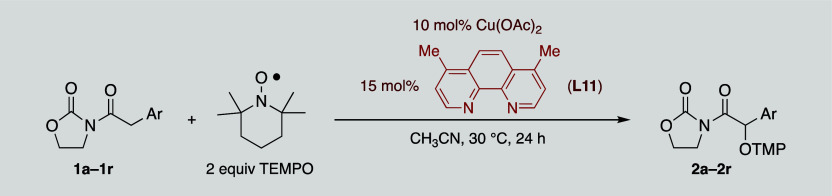

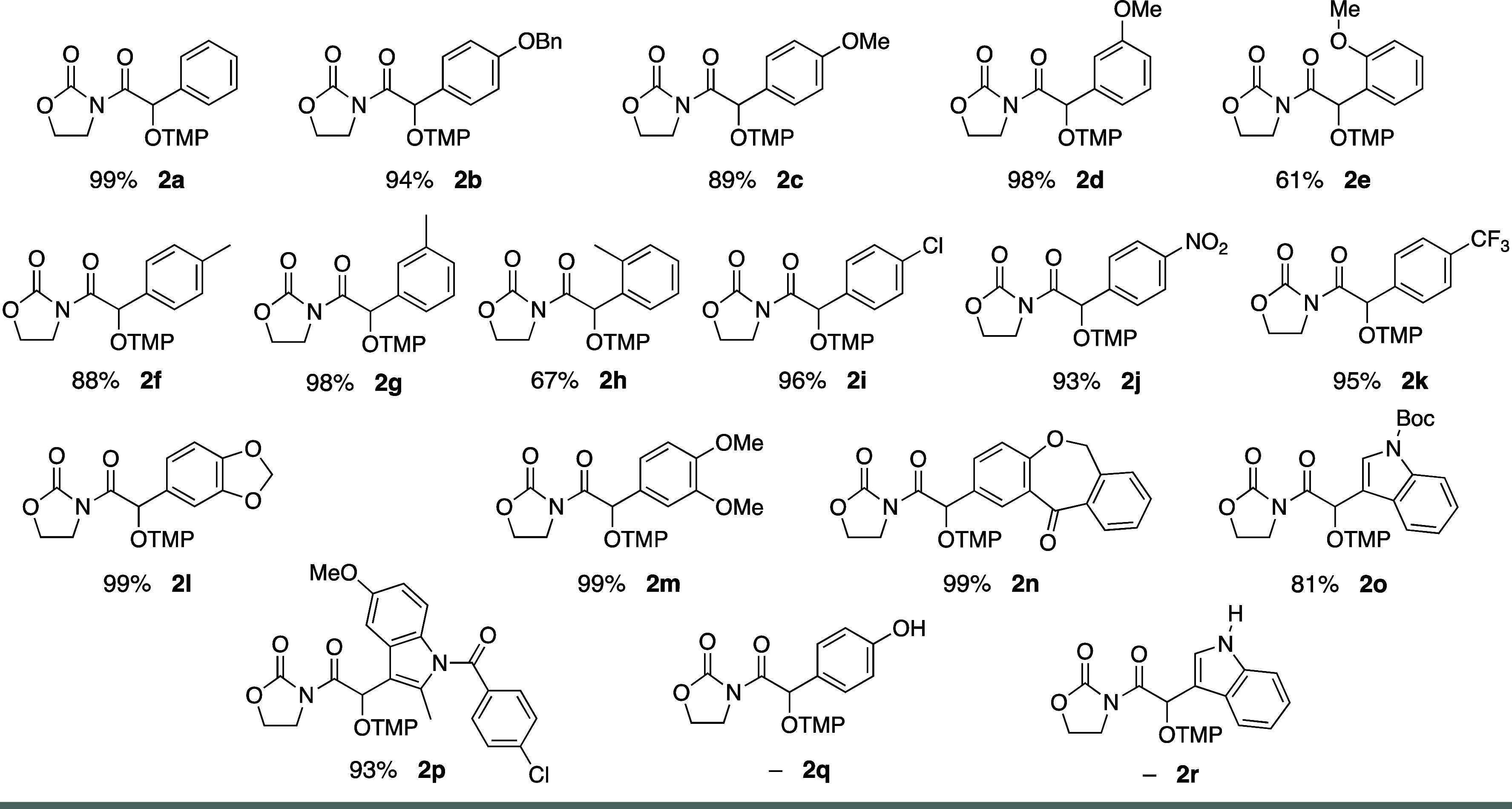
Scope of the Reaction^a^

a Reactions at 0.3 mmol scale and 0.2 M.

All in all, these results highlight the broad applicability of the reaction, which efficiently accommodates a wide range of *N*-arylacetyl imides **1**, yielding Cα-oxygenated adducts **2** with remarkable efficiency.

Next, we investigated the reduction of the N–O bond of the OTMP group to obtain the Cα hydroxy derivative.^9a,16^ Unfortunately, none of the conditions tested (Zn/AcOH, catalytic hydrogenation, ...) proved effective, so we shifted our focus to the removal of the oxazolidinone scaffold (Scheme 4). Treatment of the model adduct **2l** with LiOOH, aiming to obtain the corresponding carboxylic acid, was unsuccessful, as the oxidative conditions compromised the OTMP moiety. The use of LiOH resulted in the formation of the undesired amide **3l** in 87% yield as the steric bulk of the OTMP group directed the nucleophilic attack of the hydroxyl anion to the endocyclic carbonyl. In contrast, reductive conditions proved much more effective. Indeed, treatment with LiBH_4_ at 0 °C gave alcohol **4l** in 97% yield. On the other hand, the stronger reducing agent LiAlH_4_ at −50 °C led to the formation of aldehyde **5l** in 64% yield. Finally, methyl ester **6l** was isolated in an excellent 96% yield by heating a methanol solution of **2l** in the presence 5 mol % of ytterbium triflate.^17^

**Scheme 4:**
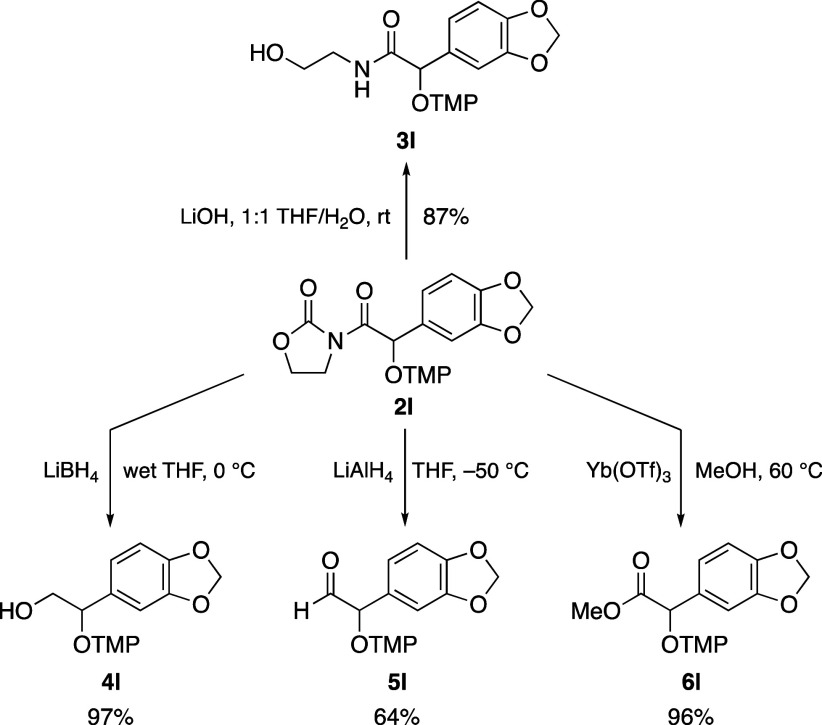
Removal of the Oxazolidinone Heterocycle

Once we had demonstrated the broad scope of the reaction and the ability to efficiently remove the heterocycle, we next applied the reported procedure to the formal synthesis of isoproterenol, an amino alcohol used in the treatment of respiratory diseases.^18^ The synthesis commenced with the well–known oxidation of **1m** on a large scale (Scheme 5).^19^ To our pleasure, the desired adduct **2m** was isolated in 97% yield, which was then converted into alcohol **4m** with a remarkable 87% yield. Taken together, these results highlight the robustness of the overall method. Activation of the hydroxyl group followed by treatment of the resultant mesylate with isopropylamine afforded amine **7** in a 57% two-step yield. Subsequent reduction of the N–O bond with zinc in acetic acid was feasible following the removal of the oxazolidinone scaffold, and it successfully gave the desired amino alcohol **8** in quantitative yield. In summary, advanced intermediate **8**, which provides a direct route to isoproterenol, was obtained from imide **1m** in five steps with an overall yield of 47%.

**Scheme 5:**
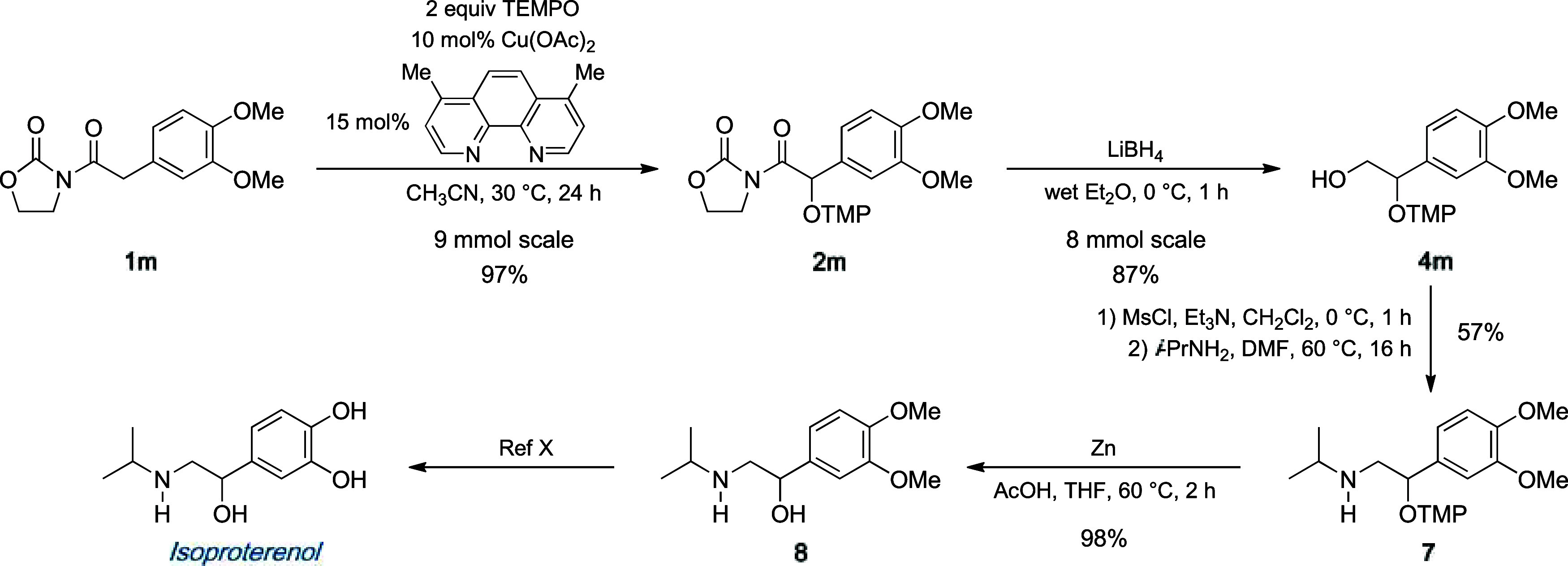
Formal Synthesis of Isoproterenol

The satisfactory Cα oxidation of *N*-arylacetyl oxazolidinones **1** prompted us to explore whether similar experimental conditions could be extended to other substrates, aiming to broaden the scope of the oxidation method. With this objective in mind, we examined the reactivity of tertiary arylacetic amides, structurally related thioimides, as well as fully saturated *N*-acyl oxazolidinones, along with their α,β- and β,γ-unsaturated counterparts (Scheme 6). Amide **9a** did not show any reactivity under the established reaction conditions. In contrast, thioimides **10a** and **11a** underwent oxidation more rapidly than **1a**, although the yields of the Cα oxidized adducts, **15a** and **16a** respectively, were lower. Altogether, these findings suggest that the presence of an imide or thioimide group, capable of chelating the putative metal enolate, is critical for the reaction and highlight the advantage of employing the more robust oxazolidinone scaffold. Among the other substrates tested, *N*-propanoyl oxazolidinone **12a** resulted totally unreactive, whereas the α,β-unsaturated imide **13a** yielded only tiny amounts of the γ-aminoxylated adduct **17a**. However, the slightly more acidic *N*-[3-butenoyl]-1,3-oxazolidin-2-one **14a** proved to be highly reactive and exclusively produced the γ-oxidized adduct **17a** in 82% yield, along with 15% of the unreactive *N*-(2-butenoyl) oxazolidinone **13a** (see Scheme 6 and Table 4).

**Scheme 6:**
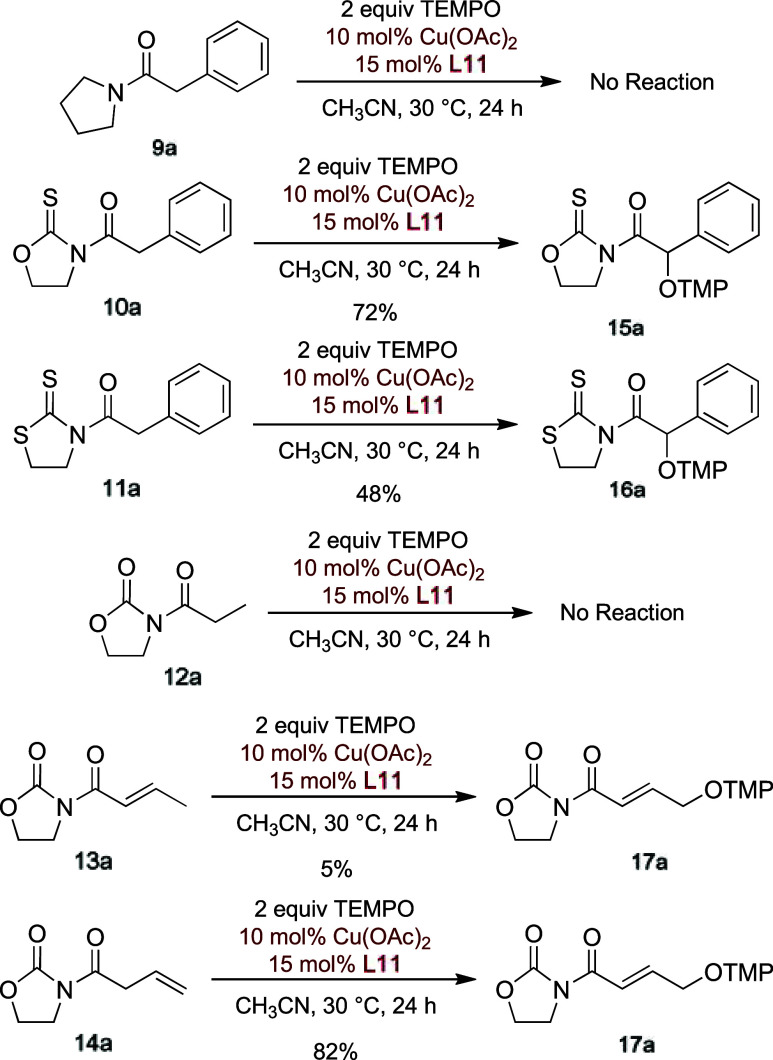
Substrate Tests

**Table 4:**
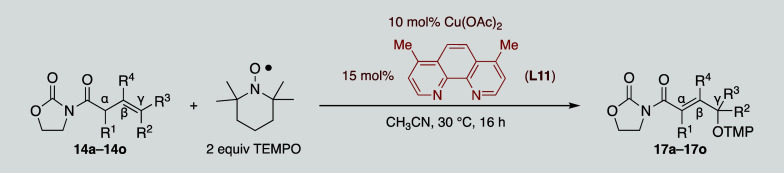

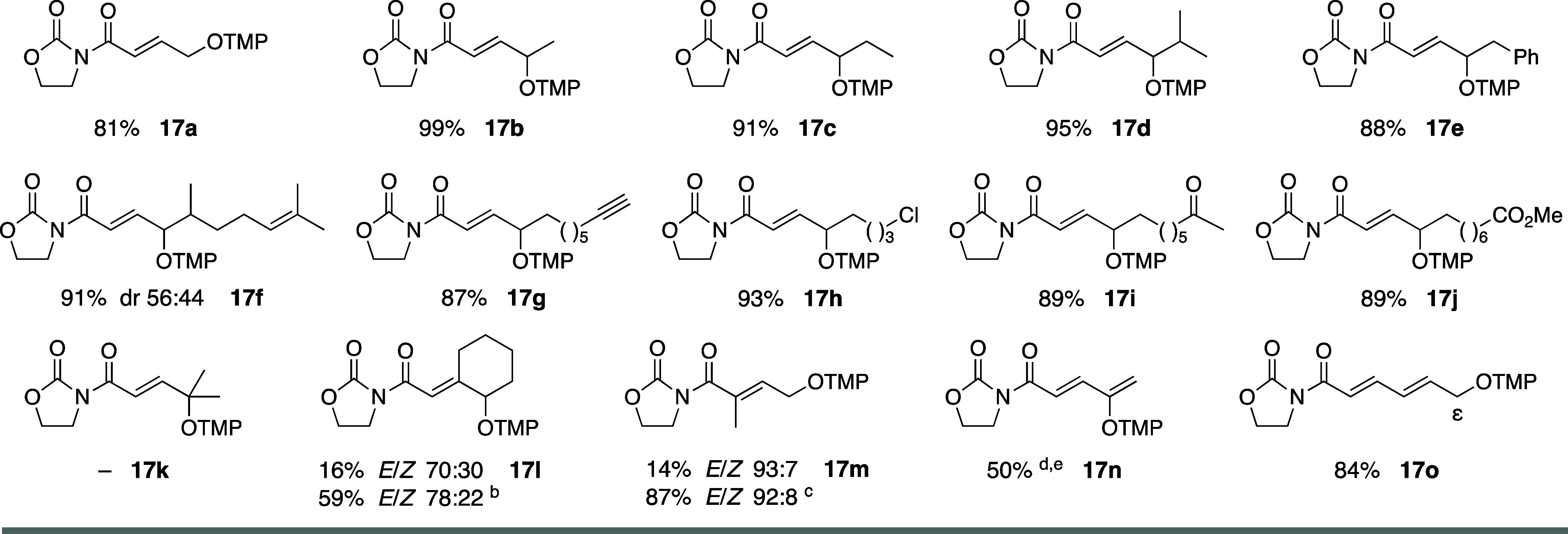
Scope of the Reaction^a^

a Reactions at 0.3 mmol scale and 0.2 M.

b Reaction conditions: 3 equiv TEMPO, 70 °C, 48 h.

c Reaction conditions: 3 equiv TEMPO, 70 °C, 72 h.

d Reaction carried out at 0.1 mmol.

e A 40% of the α,β-γ,δ-doubly unsaturated imide was also isolated.

From such a promising result, experimental parameters, including solvent, ligand, temperature, and time, were reevaluated carefully. The optimized conditions were essentially the same as those used for arylacetyl imides **1**, apart from the reaction time, which was safely reduced to 16 h. Next, these conditions were applied to a broad range of β,γ-unsaturated imides **14a–14o** to efficiently provide the corresponding oxidized derivatives **17a–17o** (Table 4). Importantly, only the Cγ-aminoxylated adducts **17a–17n** were obtained, without observing the formation of the corresponding Cα adducts, consistent with the results reported by Maulide in a related procedure proceeding through a radical pathway (see eq 4 in Scheme 2).^13^ The only exception was the doubly unsaturated imide **14o**, which yielded the fully unsaturated Cε-oxidized adduct **17o** in 84% (Table 4).

The data presented in Table 4 demonstrated that the experimental conditions could be applicable to a wide range of substrates with satisfactory yields. Importantly, β,γ-unsaturated imides containing disubstituted olefins (R^1^ = R^2^ = R^4^ = H) produced the corresponding α,β-unsaturated γ-oxidized adducts with complete regioselectivity in yields ranging from 81% to 99%. Indeed, imides bearing simple alkyl groups (R^3^ = H, alkyl in **14a–14d**) afforded adducts **17a–17d** in high yields, regardless of the steric hindrance of the substituents. Furthermore, the reaction tolerated the presence of a variety of functional groups, including phenyl, alkene, alkyne, chloride, ketone, and ester groups (**14e–14j**), with γ-oxidized adducts **17e–17j** isolated in yields around 90%. Otherwise, imide **14k** possessing a doubly substituted γ position (R^2^ = R^3^ = Me), produced the α,β-γ,δ doubly unsaturated imide instead of the desired adduct **17k**. To our pleasure, imides **14l** and **14m**, which contain substituents at R^1^ and R^4^ that could potentially hinder the reaction, underwent a totally regioselective oxidation, albeit under more stringent conditions. In this context, the high yield obtained from α-methyl-β,γ-unsaturated imide **14m** was particularly rewarding, as the generation of chelated metal enolates from such substrates is typically challenging. It is also worth mentioning that imide **14n**, which contains an allene group, provided the fully conjugated adduct **17n** in 50% yield. Finally, β,γ-δ,ε-doubly unsaturated imide **14o** afforded the ε-aminoxylated adduct **17o** with a complete regioselectivity in an 84% yield.

The removal of the oxazolidinone scaffold of adducts **17** was troublesome. The use of a reducing agent, such as LiBH_4_, with model adduct **17b**, a transformation successfully employed for arylacetyl derivatives **2**, was in this case useless and produced an almost equimolar mixture of the expected alcohol **18b** and the fully saturated alcohol **19b** in a good overall yield (Scheme 7).^20^ Instead, heating a mixture of **17b** and a catalytic amount of ytterbium triflate in methanol gave the small but densely functionalized ester **20b** in excellent yield (Scheme 7).

**Scheme 7:**
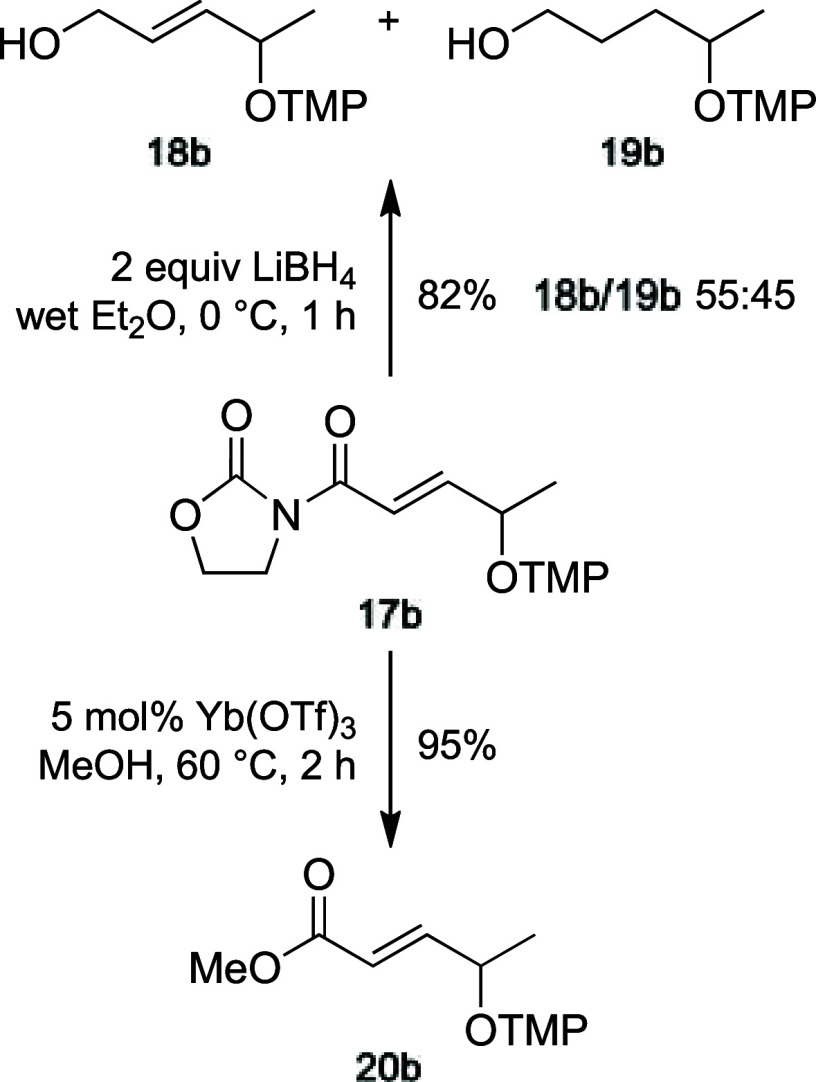
Methyl Ester from γ-Oxidized Adduct **17b**

The results summarized in Table 4 demonstrate the efficiency of the direct and catalytic oxidation of β,γ-unsaturated imides with TEMPO to provide the corresponding γ-aminoxylated adducts in high yields with remarkable chemo- and regioselectivities.

Finally, and in the absence of a more detailed mechanistic study or experimental evidence, our working hypothesis to account for the observed results is based on the reaction of the biradical form of a copper­(II) enolate with TEMPO, followed by the subsequent oxidation of the resulting copper­(I) complex with a second TEMPO molecule (Scheme 8).^21^ We are fully aware that the complex reactivity of TEMPO may conceal alternative behavior, and a more detailed mechanistic study is thus necessary to confirm the radical character of the process.

**Scheme 8:**
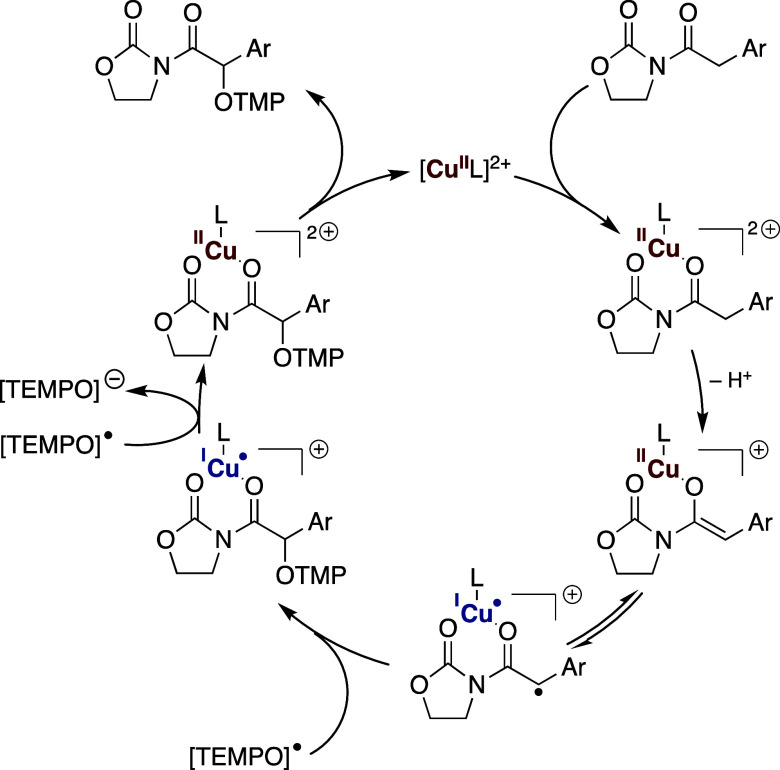
Mechanistic Working Hypothesis

## Conclusion

In summary, we have developed a direct anaerobic oxidation method for *N*-(arylacetyl) and *N*-(β,γ-unsaturated acyl)-1,3-oxazolidin-2-ones with TEMPO, catalyzed by copper­(II) acetate and 4,7-dimethyl-1,10-phenanthroline under mild experimental conditions. This method is applicable to a wide variety of substrates and exhibits high chemo- and regioselectivity, providing exclusively the corresponding α- and γ-OTMP derivatives in high yields. As proof of its synthetic utility, the method has been successfully employed in the formal synthesis of isoproterenol, an amino alcohol used in the treatment of respiratory diseases.

## Experimental Section

### General Procedure for the Synthesis of **2**

A round-bottom flask equipped with a magnetic stirring bar was charged with *N*-arylacetyl-1,3-oxazolidin-2-one **1** (0.3 mmol, 1.0 equiv), anhydrous Cu­(OAc)_2_ (5.45 mg, 30 μmol, 0.10 equiv, 10 mol %), 4,7-dimethyl-1,10-phenanthroline (9.37 mg, 45 μmol, 0.15 equiv, 15 mol %), and TEMPO (93.8 mg, 0.6 mmol, 2.0 equiv), followed by the addition of acetonitrile (1.5 mL) to get a 0.2 M solution. The resultant mixture was stirred under N_2_ at 30 °C for 24 h and then quenched with sat. NH_4_Cl (1.5 mL).

The aqueous layer was extracted with EtOAc (3 × 1.5 mL). The combined organic extracts were dried (MgSO_4_) and concentrated in vacuo. The resulting residue was purified by column chromatography to yield the Cα aminoxylated adduct **2**.

### General Procedure for the Synthesis of **14**

A round-bottom flask equipped with a magnetic stirring bar was charged with *N*-(β,γ-unsaturated acyl)-1,3-oxazolidin-2-one **14** (0.3 mmol, 1.0 equiv), anhydrous Cu­(OAc)_2_ (5.45 mg, 30 μmol, 0.10 equiv, 10 mol %), 4,7-dimethyl-1,10-phenanthroline (9.37 mg, 45 μmol, 0.15 equiv, 15 mol %), and TEMPO (93.8 mg, 0.6 mmol, 2.0 equiv), followed by the addition of acetonitrile (1.5 mL) to get a 0.2 M solution. The resultant mixture was stirred under N_2_ at 30 °C for 16 h and then quenched with sat. NH_4_Cl (1.5 mL).

The aqueous layer was extracted with EtOAc (3 × 1.5 mL). The combined organic extracts were dried (MgSO_4_) and concentrated in vacuo. The resulting residue was purified by column chromatography to yield the α,β-unsaturated Cγ aminoxylated adduct **17**.

## Supplementary Material



## Data Availability

The data underlying this study are available in the published article and its Supporting Information.

## Notes

Experimental procedures, characterization data of all new compounds, and NMR spectra (PDF)

## †

M.P.-P.: Postdoctoral Researcher at Institut de Química Avançada–CSIC, Barcelona, 08034, Spain

## ‡

C.B.: Almirall, Carretera Martorell 41–61, Sant Andreu de la Barca, 08740, Spain.
