# Supplementary material for: In Search of Radical Transformations from Metal Enolates. Direct Reactions of N‑Acyl-1,3-oxazolidin-2-ones with TEMPO Catalyzed by Copper(II) Acetate
Source: J Org Chem. 2025 May 27;90(22):7332–9. doi: 10.1021/acs.joc.5c00457 (PMC12169681; doi:10.1021/acs.joc.5c00457)

# Supporting Information

# In Search of Radical Transformations from Metal Enolates. Direct Reactions of *N*-Acyl-1,3-oxazolidin-2-ones with TEMPO Catalyzed by Copper(II) Acetate

Eduard Balaguer-Garcia[a], Marina Pérez-Palau[a], Cristina Bello[a], Anna M. Costa[a],*

Pedro Romea[a],* and Fèlix Urpí[a],*

*[a] Department of Inorganic and Organic Chemistry, Section of Organic Chemistry, and Institut de Biomedicina de la Universitat de Barcelona (IBUB), Universitat de Barcelona, 08028 Barcelona, Catalonia, Spain.*

## Table of Contents

1. General Methods ........ S2
2. Preparation of *N*-Acyl-1,3-oxazolidin-2-ones ........ S3
3. Copper Salts Screening ........ S11
   3.1. General Procedure ........ S11
   3.2. Table S1. Influence of the Catalyst on the Reaction ........ S11
4. Scope of the α-Aminoxylation Reaction ........ S12
5. Summary of the Synthesis ........ S21
6. Synthetic Steps ........ S22
7. Removal of the Achiral Auxiliary from **2l** ........ S25
8. Miscellaneous ........ S28
9. Preparation of *N*-(β,γ-Unsaturated)acyl-1,3-oxazolidin-2-ones ........ S32
   9.1. Synthesis of Aldehydes ........ S32
   9.2. Synthesis of β,γ-Unsaturated Carboxylic Acids [7] ........ S33
   9.3. Synthesis of Imides ........ S37
10. Scope of the γ-Aminoxylation Reaction ........ S44
11. Removal of the Heterocycle from **17b** ........ S52
12. References ........ S54
13. NMR Spectra of Synthesized Compounds ........ S55

## 1. General Methods

Unless otherwise stated, all reactions were conducted in oven-dried glassware under nitrogen atmosphere. The solvents and reagents were dried and purified when necessary, according to standard procedures. Commercially available reagents were used as received.

Analytical thin-layer chromatography (TLC) was carried out on Merck silica gel 60 $F_{254}$ plates and analyzed by UV (254 nm) and stained either with phosphomolybdic acid or potassium permanganate. Column chromatographies were carried under low pressure (flash) conditions and performed on SDS silica gel 60 (35–70 μm). Eluents are indicated in brackets in each case. $R_f$ values are approximate.

Melting points (Mp) were determined with a Stuart SMP10 apparatus and are uncorrected.

IR spectra (Attenuated Total Reflectance, ATR) were recorded on a Nicolet 6700 FT-IR Thermo Scientific spectrometer and only the more representative frequencies (ν) are reported in $cm^{-1}$.

$^{1}H$ NMR (400 MHz or 500 MHz) and $^{13}C\{^{1}H\}$ NMR (101 MHz or 126 MHz) spectra were recorded at room temperature on a Varian Mercury 400, a Bruker 400 Advance III or a Bruker 500 Avance Neo. Chemical shifts (δ) are quoted in ppm and referenced to internal TMS (δ 0.00 for $^{1}H$ NMR), $CDCl_3$ (δ 7.26 for $^{1}H$ NMR and δ 77.0 for $^{13}C$ NMR) or DMSO-$d_6$ (δ 2.50 for $^{1}H$ NMR and δ 39.5 for $^{13}C$ NMR). Data are reported as follows: chemical shift (number of protons, multiplicity, coupling constants, proton); multiplicity is reported as follows: s, singlet; br s, broad singlet; d, doublet; t, triplet; q, quartet; p, quintet; h, heptuplet; or m, multiplet (and their corresponding combinations); coupling constants ($J$) are quoted in Hz. Where necessary, 2D techniques (COSY, HSQC, NOESY) were also used to assist on structure elucidation.

High resolution mass spectra (HRMS) were obtained with an Agilent 1100 spectrometer using a TOF analyzer by the Unitat d'Espectrometria de Masses, Universitat de Barcelona.

## 2. Preparation of *N*-Acyl-1,3-oxazolidin-2-ones

**General Procedure 1**

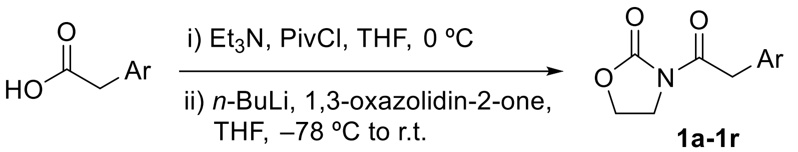


**1a-1r**

Neat $Et_3N$ (1.3 equiv.) and PivCl (1.3 equiv.) were added to a 0.2 M solution of the corresponding carboxylic acid (1.3 equiv.) in THF at 0 °C under nitrogen atmosphere. The resulting mixture was stirred at this temperature for 90 min, and it was cooled to −78 °C. Meanwhile, a 2.5 M solution of *n*-BuLi in hexanes (1.1 equiv.) was added dropwise to a 0.4 M solution of 1,3-oxazolidin-2-one (1.0 equiv.) in THF under nitrogen at −78 °C and stirred for 15 min. The resulting solution was added to the first mixture via cannula. The reaction mixture was stirred at −78 °C for 20 min and it was allowed to warm to r.t. and stirred for 2 h.
Then, it was quenched with sat. $NH_4Cl$ and extracted with EtOAc. The combined organic extracts were washed with sat. $NaHCO_3$, brine and dried with anhydrous $MgSO_4$. The volatiles were evaporated under reduced pressure and the residue was purified using flash column chromatography.

**_N_-Phenylacetyl-1,3-oxazolidin-2-one (1a)**

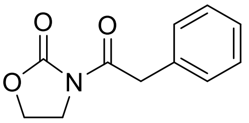

It was prepared following General Procedure 1 from phenylacetic acid (1.77 g, 13 mmol), $Et_3N$ (1.8 mL, 13 mmol), PivCl (1.6 mL, 13 mmol), 1,3-oxazolidin-2-one (871 mg, 10 mmol) and *n*-BuLi (2.5 M in hexanes, 4.4 mL, 11 mmol). Purification of the crude product by flash column chromatography (from 70:30 to 50:50 hexanes/EtOAc) afforded **1a** (1.32 g, 6.4 mmol, 64% yield) as a white solid. **Mp** 67–69 °C; **$R_f$** (60:40 hexanes/EtOAc) 0.4; **IR** (ATR) ν 3029, 2922, 1767, 1691, 1385, 1363, 1264, 1219, 1175, 1032, 1015 $cm^{-1}$; **$^1$H NMR** (500 MHz, $CDCl_3$) δ 7.35–7.24 (5H, m, ArH), 4.41–4.35 (2H, m, $OCH_2$), 4.28 (2H, s, $COCH_2$), 4.03–3.99 (2H, m, $NCH_2$); **$^{13}$C{$^1$H} NMR** (126 MHz, $CDCl_3$) δ 171.4 (C), 153.6 (C), 133.6 (C), 129.8 (CH), 128.7 (CH), 127.3 (CH), 62.1 ($CH_2$), 42.8 ($CH_2$), 41.2 ($CH_2$); **HRMS** (+ESI): *m/z* calcd. for $C_{11}H_{11}NNaO_3$ $[M+Na]^+$: 228.0631, found: 228.0630.

**_N_-[(4-Benzyloxyphenyl)acetyl]-1,3-oxazolidin-2-one (1b)** [1]

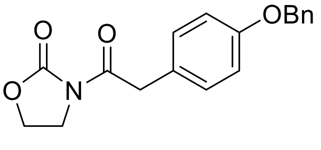


It was prepared following General Procedure 1 from (4-benzyloxyphenyl)acetic acid (1.57 g, 6.5 mmol), $Et_3N$ (0.9 mL, 6.5 mmol), PivCl (0.8 mL, 6.5 mmol), 1,3-oxazolidin-2-one (435 mg, 5.0 mmol) and *n*-BuLi (2.5 M in hexanes, 2.2 mL, 5.5 mmol). Purification of the crude product by flash column chromatography (from 70:30 to 50:50 hexanes/EtOAc) afforded **1b** (882 mg, 2.8

mmol, 57% yield) as a white solid. **Mp** 115–116 °C; **R$_f$** (60:40 hexanes/EtOAc) 0.2; **IR** (ATR) ν 2920, 2863, 1763, 1705, 1510, 1382, 1238, 1218, 1201, 1174, 1103, 1014, 753 cm$^{-1}$; **$^1$H NMR** (400 MHz, CDCl$_3$) δ 7.47–7.29 (5H, m, ArH), 7.28–7.21 (2H, m, ArH), 6.97–6.90 (2H, m, ArH), 5.05 (2H, s, OCH$_2$Ph), 4.43–4.34 (2H, m, OCH$_2$CH$_2$), 4.22 (2H, s, COCH$_2$), 4.05–3.97 (2H, m, NCH$_2$); **$^{13}$C{$^1$H} NMR** (101 MHz, CDCl$_3$) δ 171.7 (C), 158.2 (C), 153.6 (C), 137.1 (C), 130.9 (CH), 128.7 (CH), 128.1 (CH), 127.6 (CH), 125.9 (C), 115.0 (CH), 70.1 (CH$_2$), 62.1 (CH$_2$), 42.8 (CH$_2$), 40.3 (CH$_2$).

### *N*-[(4-Methoxyphenyl)acetyl]-1,3-oxazolidin-2-one (1c) [2]

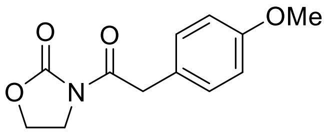


It was prepared following General Procedure 1 from (4-methoxyphenyl)acetic acid (1.08 g, 6.5 mmol), Et$_3$N (0.9 mL, 6.5 mmol), PivCl (0.8 mL, 6.5 mmol), 1,3-oxazolidin-2-one (435 mg, 5.0 mmol) and *n*-BuLi (2.5 M in hexanes, 2.2 mL, 5.5 mmol). Purification of the crude product by flash column chromatography (from 70:30 to 50:50 hexanes/EtOAc) afforded **1c** (827 mg, 3.5 mmol, 70% yield) as a white solid. **Mp** 117–118 °C; **R$_f$** (60:40 hexanes/EtOAc) 0.2; **IR** (ATR) ν 3041, 2977, 2908, 2838, 1784, 1695, 1517, 1387, 1367, 1238, 1218, 1194, 1184, 1036, 1020, 754 cm$^{-1}$; **$^1$H NMR** (500 MHz, CDCl$_3$) δ 7.28–7.20 (2H, m, ArH), 6.89–6.82 (2H, m, ArH), 4.42–4.35 (2H, m, OCH$_2$), 4.22 (2H, s, COCH$_2$), 4.04–3.97 (2H, m, NCH$_2$), 3.79 (3H, s, OCH$_3$); **$^{13}$C{$^1$H} NMR** (126 MHz, CDCl$_3$) δ 171.8 (C), 158.9 (C), 153.6 (C), 130.9 (CH), 125.6 (C), 114.1 (CH), 62.1 (CH$_2$), 55.4 (CH$_3$), 42.8 (CH$_2$), 40.3 (CH$_2$).

### *N*-[(3-Methoxyphenyl)acetyl]-1,3-oxazolidin-2-one (1d) [3]

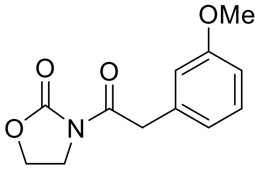


It was prepared following General Procedure 1 from (3-methoxyphenyl)acetic acid (1.08 g, 6.5 mmol), Et$_3$N (0.9 mL, 6.5 mmol), PivCl (0.8 mL, 6.5 mmol), 1,3-oxazolidin-2-one (435 mg, 5.0 mmol) and *n*-BuLi (2.5 M in hexanes, 2.2 mL, 5.5 mmol). Purification of the crude product by flash column chromatography (from 70:30 to 50:50 hexanes/EtOAc) afforded **1d** (664 mg, 2.8 mmol, 56% yield) as a yellow oil. **R$_f$** (60:40 hexanes/EtOAc) 0.2; **IR** (ATR) ν 2921, 2851, 1769, 1693, 1385, 1362, 1272, 1251, 1220, 1106, 1035, 1014, 756, 704, 690 cm$^{-1}$; **$^1$H NMR** (400 MHz, CDCl$_3$) δ 7.26–7.21 (1H, m, ArH), 6.92–6.78 (3H, m, ArH), 4.44–4.35 (2H, m, OCH$_2$), 4.26 (2H, s, COCH$_2$), 4.05–3.98 (2H, m, NCH$_2$), 3.79 (3H, s, OCH$_3$); **$^{13}$C{$^1$H} NMR** (101 MHz, CDCl$_3$) δ 171.2 (C), 159.8 (C), 153.6 (C), 135.1 (C), 129.6 (CH), 122.2 (CH), 115.4 (CH), 113.0 (CH), 62.1 (CH$_2$), 55.3 (CH$_3$), 42.8 (CH$_2$), 41.2 (CH$_2$).

**$N$-[(2-Methoxyphenyl)acetyl]-1,3-oxazolidin-2-one (1e)**

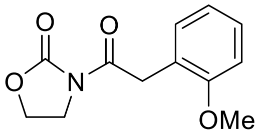

It was prepared following General Procedure 1 from (2-methoxyphenyl)acetic acid (1.08 g, 6.5 mmol), $Et_3N$ (0.9 mL, 6.5 mmol), PivCl (0.8 mL, 6.5 mmol), 1,3-oxazolidin-2-one (435 mg, 5.0 mmol) and *n*-BuLi (2.5 M in hexanes, 2.2 mL, 5.5 mmol). Purification of the crude product by flash column chromatography (from 70:30 to 50:50 hexanes/EtOAc) afforded **1e** (735 mg, 3.1 mmol, 63% yield) as a white solid. **Mp** 98–100 °C; **$R_f$** (50:50 hexanes/EtOAc) 0.3; **IR** (ATR) ν 2920, 2848, 2828, 1762, 1697, 1385, 1367, 1243, 1203, 1113, 1013, 957, 754, 691 $cm^{-1}$; **$^1H$ NMR** (400 MHz, $CDCl_3$) δ 7.27 (1H, ddd, $J$ = 8.2, 7.5, 1.8 Hz, ArH), 7.13 (1H, dd, $J$ = 7.5, 1.8 Hz, ArH), 6.92 (1H, td, $J$ = 7.5, 1.2 Hz, ArH), 6.89 (1H, dd, $J$ = 8.2, 1.2 Hz), 4.46–4.39 (2H, m, $OC\underline{H}_2$), 4.24 (2H, s, $COC\underline{H}_2$), 4.07–4.01 (2H, m, $NC\underline{H}_2$), 3.80 (3H, s, $OC\underline{H}_3$); **$^{13}C\{^1H\}$ NMR** (101 MHz, $CDCl_3$) δ 171.3 (C), 157.8 (C), 153.9 (C), 131.3 (CH), 128.8 (CH), 122.7 (C), 120.6 (CH), 110.6 (CH), 62.2 ($CH_2$), 55.6 ($CH_3$), 42.8 ($CH_2$), 37.0 ($CH_2$); **HRMS** (+ESI): *m/z* calcd. for $C_{12}H_{13}NNaO_4$ $[M+Na]^+$: 258.0737, found: 258.0737.

**$N$-($p$-Tolylacetyl)-1,3-oxazolidin-2-one (1f)** [3]

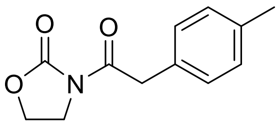

It was prepared following General Procedure 1 from *p*-tolylacetic acid (976 mg, 6.5 mmol), $Et_3N$ (0.9 mL, 6.5 mmol), PivCl (0.8 mL, 6.5 mmol), 1,3-oxazolidin-2-one (435 mg, 5.0 mmol) and *n*-BuLi (2.5 M in hexanes, 2.2 mL, 5.5 mmol). Purification of the crude product by flash column chromatography (from 70:30 to 60:40 hexanes/EtOAc) afforded **1f** (799 mg, 3.6 mmol, 73% yield) as a white solid. **Mp** 78–80 °C; **$R_f$** (60:40 hexanes/EtOAc) 0.3; **IR** (ATR) ν 2924, 1760, 1697, 1394, 1367, 1261, 1234, 1112, 1037, 1015, 761, 687 $cm^{-1}$; **$^1H$ NMR** (500 MHz, $CDCl_3$) δ 7.23–7.18 (2H, m, ArH), 7.16–7.11 (2H, m, ArH), 4.42–4.35 (2H, m, $OC\underline{H}_2$), 4.24 (2H, s, $COC\underline{H}_2$), 4.05–3.98 (2H, m, $NC\underline{H}_2$), 2.33 (3H, s, $C\underline{H}_3$); **$^{13}C\{^1H\}$ NMR** (126 MHz, $CDCl_3$) δ 171.6 (C), 153.6 (C), 137.0 (C), 130.6 (C), 129.7 (CH), 129.4 (CH), 62.1 ($CH_2$), 42.8 ($CH_2$), 40.8 ($CH_2$), 21.2 ($CH_3$).

**$N$-($m$-Tolylacetyl)-1,3-oxazolidin-2-one (1g)** [3]

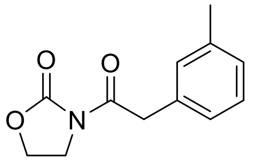

It was prepared following General Procedure 1 from *m*-tolylacetic acid (976 mg, 6.5 mmol), $Et_3N$ (0.9 mL, 6.5 mmol), PivCl (0.8 mL, 6.5 mmol), 1,3-oxazolidin-2-one (435 mg, 5.0 mmol) and *n*-BuLi (2.5 M in hexanes, 2.2 mL, 5.5 mmol). Purification of the crude product by flash column chromatography (from 70:30 to 60:40 hexanes/EtOAc) afforded **1g** (691 mg, 3.2 mmol, 63% yield) as a white solid. **Mp** 60–61 °C; **$R_f$** (60:40 hexanes/EtOAc) 0.3; **IR** (ATR) ν 3009, 2920, 1763, 1682, 1391, 1340, 1225, 1180, 1030, 772, 756 $cm^{-1}$; **$^1H$ NMR** (500 MHz, $CDCl_3$) δ 7.22

(1H, t, $J$ = 7.5 Hz, ArH), 7.16–7.06 (3H, m, ArH), 4.42–4.36 (2H, m, $OCH_2$), 4.25 (2H, s, $COCH_2$), 4.05–3.99 (2H, m, $NCH_2$), 2.34 (3H, s, $CH_3$); **$^{13}C\{^1H\}$ NMR** (126 MHz, $CDCl_3$) δ 171.5 (C), 153.6 (C), 138.3 (C), 133.5 (C), 130.6 (CH), 128.6 (CH), 128.1 (CH), 126.9 (CH), 62.1 ($CH_2$), 42.8 ($CH_2$), 41.1 ($CH_2$), 21.5 ($CH_3$).

**_N_-(_o_-Tolylacetyl)-1,3-oxazolidin-2-one (1h)** [3]

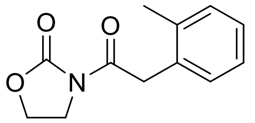

It was prepared following General Procedure 1 from *o*-tolylacetic acid (976 mg, 6.5 mmol), $Et_3N$ (0.9 mL, 6.5 mmol), PivCl (0.8 mL, 6.5 mmol), 1,3-oxazolidin-2-one (435 mg, 5.0 mmol) and *n*-BuLi (2.5 M in hexanes, 2.2 mL, 5.5 mmol). Purification of the crude product by flash column chromatography (from 70:30 to 60:40 hexanes/EtOAc) afforded **1h** (404 mg, 1.8 mmol, 37% yield) as a white solid. **Mp** 69–71 °C; **$R_f$** (60:40 hexanes/EtOAc) 0.3; **IR** (ATR) ν 3015, 2920, 1768, 1689, 1386, 1372, 1269, 1252, 1207, 1102, 1035, 1011, 965, 746 cm$^{-1}$; **$^1H$ NMR** (500 MHz, $CDCl_3$) δ 7.22–7.12 (4H, m, ArH), 4.46–4.39 (2H, m, $OCH_2$), 4.28 (2H, s, $COCH_2$), 4.07–4.01 (2H, m, $NCH_2$), 2.28 (3H, s, $CH_3$); **$^{13}C\{^1H\}$ NMR** (126 MHz, $CDCl_3$) δ 171.2 (C), 153.8 (C), 137.3 (C), 132.5 (C), 130.4 (CH), 130.2 (CH), 127.6 (CH), 126.2 (CH), 62.2 ($CH_2$), 42.8 ($CH_2$), 39.5 ($CH_2$), 19.7 ($CH_3$).

**_N_-[(4-Chlorophenyl)acetyl]-1,3-oxazolidin-2-one (1i)** [3]

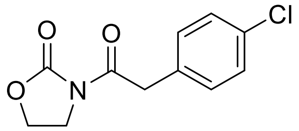

It was prepared following General Procedure 1 from (4-chlorophenyl)acetic acid (1.11 g, 6.5 mmol), $Et_3N$ (0.9 mL, 6.5 mmol), PivCl (0.8 mL, 6.5 mmol), 1,3-oxazolidin-2-one (435 mg, 5.0 mmol) and *n*-BuLi (2.5 M in hexanes, 2.2 mL, 5.5 mmol). Purification of the crude product by flash column chromatography (from 80:20 to 60:40 hexanes/EtOAc) afforded **1i** (629 mg, 2.6 mmol, 52% yield) as a white solid. **Mp** 108–110 °C; **$R_f$** (60:40 hexanes/EtOAc) 0.3; **IR** (ATR) ν 2924, 2851, 1763, 1696, 1492, 1476, 1403, 1387, 1362, 1219, 1203, 1110, 1091, 761 cm$^{-1}$; **$^1H$ NMR** (500 MHz, $CDCl_3$) δ 7.33–7.21 (4H, m, ArH), 4.45–4.37 (2H, m, $OCH_2$), 4.25 (2H, s, $COCH_2$), 4.05–3.99 (2H, m, $NCH_2$); **$^{13}C\{^1H\}$ NMR** (126 MHz, $CDCl_3$) δ 171.0 (C), 153.6 (C), 133.3 (C), 132.0 (C), 131.2 (CH), 128.8 (CH), 62.2 ($CH_2$), 42.8 ($CH_2$), 40.6 ($CH_2$).

**_N_-[(4-Nitrophenyl)acetyl]-1,3-oxazolidin-2-one (1j)**

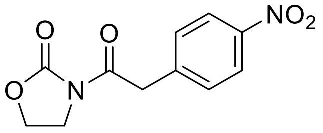

It was prepared following General Procedure 1 from (4-nitrophenyl)acetic acid (1.18 g, 6.5 mmol), $Et_3N$ (0.9 mL, 6.5 mmol), PivCl (0.8 mL, 6.5 mmol), 1,3-oxazolidin-2-one (435 mg, 5.0 mmol) and *n*-BuLi (1.6 M in hexanes, 3.4 mL, 5.5 mmol). Purification of the crude product by flash column chromatography (from 70:30 to 40:60 hexanes/EtOAc) afforded **1j** (233 mg, 0.9 mmol, 19% yield) as a light-orange solid. **Mp** 157–161 °C; **$R_f$** (60:40 hexanes/EtOAc) 0.2; **IR**

(ATR) ν 3107, 3082, 2924, 2851, 1775, 1692, 1605, 1513, 1391, 1346, 1273, 1226, 1201, 1109, 1042, 711 cm$^{-1}$; **$^1$H NMR** (400 MHz, $CDCl_3$) δ 8.21–8.15 (2H, m, Ar<u>H</u>), 7.52–7.44 (2H, m, Ar<u>H</u>), 4.49–4.42 (2H, m, OC<u>H</u>$_2$), 4.39 (2H, s, COC<u>H</u>$_2$), 4.09–4.01 (2H, m, NC<u>H</u>$_2$); **$^{13}$C{$^1$H} NMR** (101 MHz, $CDCl_3$) δ 169.9 (C), 153.6 (C), 147.4 (C), 141.0 (C), 130.9 (CH), 123.8 (CH), 62.3 ($CH_2$), 42.8 ($CH_2$), 41.1 ($CH_2$); **HRMS** (–ESI): *m/z* calcd. for $C_{11}H_9N_2O_5$ [M–H]$^-$: 249.0506, found: 249.0512.

### *N*-[(4-Trifluoromethylphenyl)acetyl]-1,3-oxazolidin-2-one (1k) [2,3]

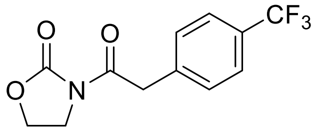

It was prepared following <u>General Procedure 1</u> from (4-trifluoromethylphenyl)acetic acid (1.33 g, 6.5 mmol), $Et_3N$ (0.9 mL, 6.5 mmol), PivCl (0.8 mL, 6.5 mmol), 1,3-oxazolidin-2-one (384 mg, 4.4 mmol) and *n*-BuLi (2.5 M in hexanes, 2.2 mL, 5.5 mmol). Purification of the crude product by flash column chromatography (from 70:30 to 50:50 hexanes/EtOAc) afforded **1k** (619 mg, 2.3 mmol, 51% yield) as a white solid. **Mp** 139–142 °C; **R$_f$** (60:40 hexanes/EtOAc) 0.3; **IR** (ATR) ν 2930, 2857, 1761, 1693, 1408, 1322, 1232, 1108, 1067, 1038, 1014, 697 cm$^{-1}$; **$^1$H NMR** (400 MHz, DMSO-*d*$_6$) δ 7.71–7.64 (2H, m, Ar<u>H</u>), 7.51–7.43 (2H, m, Ar<u>H</u>), 4.43–4.35 (2H, m, OC<u>H</u>$_2$), 4.30 (2H, s, COC<u>H</u>$_2$), 3.95–3.86 (2H, m, NC<u>H</u>$_2$); **$^{13}$C{$^1$H} NMR** (101 MHz, DMSO-*d*$_6$) δ 170.2 (C), 153.8 (C), 139.4 (C), 130.8 (CH), 127.5 (<u>C</u>CF$_3$, q, $J$ = 31.7 Hz), 125.0 (<u>C</u>HCCF$_3$, q, $J$ = 3.7 Hz), 124.4 (<u>C</u>F$_3$, q, $J$ = 272.2 Hz), 62.5 ($CH_2$), 42.6 ($CH_2$), 40.6 ($CH_2$).

### *N*-[(Benzo[*d*][1,3]dioxol-5-yl)acetyl]-1,3-oxazolidin-2-one (1l) [2]

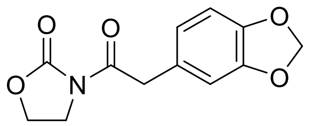

It was prepared following <u>General Procedure 1</u> from (benzo[*d*][1,3]dioxol-5-yl)acetic acid (2.34 g, 13 mmol), $Et_3N$ (1.8 mL, 13 mmol), PivCl (1.6 mL, 13 mmol), 1,3-oxazolidin-2-one (871 mg, 10 mmol) and *n*-BuLi (2.5 M in hexanes, 4.4 mL, 11 mmol). Purification of the crude product by flash column chromatography (from 60:40 to 50:50 hexanes/EtOAc) afforded **1l** (1.99 g, 8.0 mmol, 80% yield) as a white solid. **Mp** 99–100 °C; **R$_f$** (60:40 hexanes/EtOAc) 0.2; **IR** (ATR) ν 2927, 2889, 2790, 1764, 1702, 1483, 1441, 1385, 1340, 1243, 1222, 1188, 1178, 1104, 1033, 1011, 921, 757, 702 cm$^{-1}$; **$^1$H NMR** (400 MHz, $CDCl_3$) δ 6.83–6.71 (3H, m, Ar<u>H</u>), 5.93 (2H, s, OC<u>H</u>$_2$O), 4.43–4.36 (2H, m, OC<u>H</u>$_2$), 4.18 (2H, s, COC<u>H</u>$_2$), 4.05–3.98 (2H, m, NC<u>H</u>$_2$); **$^{13}$C{$^1$H} NMR** (101 MHz, $CDCl_3$) δ 171.5 (C), 153.6 (C), 147.8 (C), 146.9 (C), 127.1 (C), 123.0 (CH), 110.3 (CH), 108.4 (CH), 101.1 ($CH_2$), 62.1 ($CH_2$), 42.8 ($CH_2$), 40.8 ($CH_2$); **HRMS** (+ESI): *m/z* calcd. for $C_{12}H_{12}NO_5$ [M+H]$^+$: 250.0710, found: 250.0708.

### *N*-[2-(3,4-Dimethoxyphenyl)acetyl]-1,3-oxazolidin-2-one (1m)

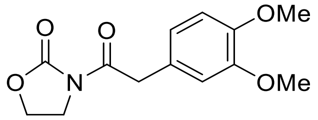

It was prepared following <u>General Procedure 1</u> from 2-(3,4-dimethoxyphenyl)acetic acid (5.10 g, 26 mmol), $Et_3N$ (3.6 mL, 26 mmol), PivCl (3.2 mL, 26 mmol), 1,3-oxazolidin-2-one (1.74 g, 20

mmol) and *n*-BuLi (2.5 M in hexanes, 8.8 mL, 22 mmol). Purification of the crude by crystallization with EtOAc/hexanes afforded **1m** (3.72 g, 14 mmol, 70% yield) as a white solid. **Mp** 105–108 ºC; **R$_f$** (50:50 hexanes/EtOAc) 0.2; **IR** (ATR) ν 2999, 2961, 2935, 1769, 1696, 1513, 1388, 1264, 1226, 1187, 1155, 1142, 1019, 760 cm$^{-1}$; **$^1$H NMR** (400 MHz, $CDCl_3$) δ 6.90–6.77 (3H, m, ArH), 4.43–4.34 (2H, m, OCH$_2$), 4.21 (2H, s, COCH$_2$), 4.06–3.96 (2H, m, NCH$_2$), 3.86 (3H, s, OCH$_3$), 3.85 (3H, s, OCH$_3$); **$^{13}$C NMR{$^1$H}** (101 MHz, $CDCl_3$) δ 171.6 (C), 153.6 (C), 148.9 (C), 148.3 (C), 126.0 (C), 122.0 (CH), 113.0 (CH), 111.3 (CH), 62.1 (CH$_2$), 56.0 (CH$_3$), 55.9 (CH$_3$), 42.8 (CH$_2$), 40.6 (CH$_2$); **HRMS** (+ESI): *m/z* calcd. for $C_{13}H_{15}NNaO_5$ [M+Na]$^+$: 288.0842, found: 288.0838.

**N-[(11-Oxo-6,11-dihydrodibenzo[b,e]oxepin-2-yl)acetyl]-1,3-oxazolidin-2-one (1n)**

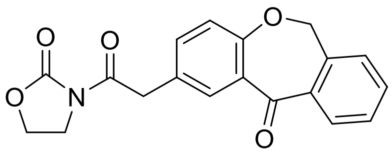

It was prepared following General Procedure 1 from (11-oxo-6,11-dihydrodibenzo[*b*,*e*]oxepin-2-yl)acetic acid (1.74 g, 6.5 mmol), $Et_3N$ (0.9 mL, 6.5 mmol), PivCl (0.8 mL, 6.5 mmol), 1,3-oxazolidin-2-one (435 mg, 5.0 mmol) and *n*-BuLi (2.5 M in hexanes, 2.2 mL, 5.5 mmol). Purification of the crude product by flash column chromatography (96:4 DCM/EtOAc) afforded **1n** (1.22 g, 3.6 mmol, 72% yield) as a white solid. **Mp** 186–188 ºC; **R$_f$** (50:50 hexanes/EtOAc) 0.3; **IR** (ATR) ν 3063, 2977, 2904, 1764, 1698, 1650, 1490, 1387, 1367, 1258, 1220, 1208, 1112, 1005, 997, 756 cm$^{-1}$; **$^1$H NMR** (400 MHz, DMSO-*d$_6$*) δ 7.97 (1H, d, *J* = 2.4 Hz, ArH), 7.78 (1H, dd, *J* = 8.0, 1.2 Hz, ArH), 7.66 (1H, td, *J* = 7.4, 1.4 Hz, ArH), 7.59–7.50 (2H, m, ArH), 7.46 (1H, dd, *J* = 8.4, 2.4 Hz, ArH), 7.06 (1H, d, *J* = 8.4 Hz, ArH), 5.29 (2H, s, OCH$_2$), 4.43–4.35 (2H, m, OCH$_2$CH$_2$), 4.22 (2H, s, COCH$_2$), 3.95–3.86 (2H, m, NCH$_2$); **$^{13}$C{$^1$H} NMR** (101 MHz, DMSO-*d$_6$*) δ 190.1 (C), 170.7 (C), 159.8 (C), 153.7 (C), 140.0 (C), 137.3 (CH), 135.9 (C), 133.0 (CH), 132.2 (CH), 129.2 (CH), 128.8 (CH), 128.3 (CH), 128.2 (C), 124.5 (C), 120.5 (CH), 72.7 (CH$_2$), 62.4 (CH$_2$), 42.6 (CH$_2$), 39.6 (CH$_2$); **HRMS** (+ESI): *m/z* calcd. for $C_{19}H_{16}NO_5$ [M+H]$^+$: 338.1023, found: 338.1021.

**N-[(N'-tert-Butyloxocarbonyl-1H-indol-3-yl)acetyl]-1,3-oxazolidin-2-one (1o)**

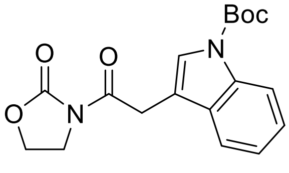

Solid DMAP (15 mg, 0.12 mmol) and $(Boc)_2O$ (402 mg, 1.84 mmol) were added to a solution of **1r** (300 mg, 1.23 mmol) in DCM (8 mL). The resultant mixture was stirred for 1 h at r.t, quenched with sat. $NH_4Cl$ (10 mL) and extracted with DCM (3 × 10 mL). The combined organic extracts were washed with brine, dried with $MgSO_4$, and concentrated under reduced pressure. Purification of the crude product by flash column chromatography (from 90:10 to 70:30 hexanes/EtOAc) afforded **1o** (312 mg, 0.9 mmol, 74% yield) as a white solid. **Mp** 115–118 ºC; **R$_f$** (60:40 hexanes/EtOAc) 0.3; **IR** (ATR) ν 2987, 2920, 1784, 1724, 1704, 1451, 1383, 1362, 1308, 1255, 1222, 1205, 1154, 1095, 1021, 750 cm$^{-1}$; **$^1$H NMR** (400 MHz, $CDCl_3$) δ 8.14 (1H, d, *J* = 8.3 Hz, ArH), 7.62 (1H, s, CHNBoc), 7.58 (1H, d, *J* = 7.7 Hz, ArH), 7.32 (1H, ddd, *J* =

8.3, 7.2, 1.3 Hz, ArH), 7.28–7.20 (1H, m, ArH), 4.47–4.38 (2H, m, OCH$_2$), 4.37 (2H, s, COCH$_2$), 4.08–3.97 (2H, m, NCH$_2$), 1.66 (9H, s, C(CH$_3$)$_3$); **$^{13}$C{$^1$H} NMR** (101 MHz, CDCl$_3$) δ 170.5 (C), 153.6 (C), 149.7 (C), 135.5 (C), 130.4 (C), 125.3 (CH), 124.6 (CH), 122.8 (CH), 119.4 (CH), 115.4 (CH), 112.6 (C), 83.8 (C), 62.2 (CH$_2$), 42.8 (CH$_2$), 31.6 (CH$_2$), 28.3 (CH$_3$); **HRMS** (+ESI): *m/z* calcd. for $C_{18}H_{20}N_2NaO_5$ [M+Na]$^+$: 367.1264, found: 367.1263.

### *N*-[*N'*-(4-Chlorobenzoyl)-5-methoxy-2-methyl-1*H*-indol-3-yl)acetyl]-1,3-oxazolidin-2-one (1p)

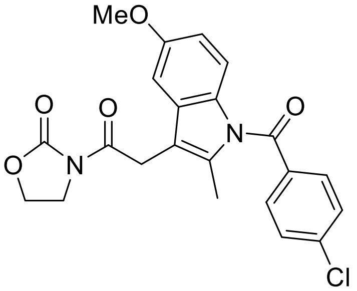


It was prepared following General Procedure 1 from [*N*-(4-chlorobenzoyl)-5-methoxy-2-methyl-1*H*-indol-3-yl]acetic acid (2.33 g, 6.5 mmol), Et$_3$N (0.9 mL, 6.5 mmol), PivCl (0.8 mL, 6.5 mmol), 1,3-oxazolidin-2-one (435 mg, 5.0 mmol) and *n*-BuLi (1.6 M in hexanes, 3.4 mL, 5.5 mmol). Purification of the crude product by flash column chromatography (from 70:30 to 50:50 hexanes/EtOAc) afforded **1p** (1.09 g, 2.6 mmol, 51% yield) as a white solid. **Mp** 161–162 °C; **R$_f$** (50:50 hexanes/EtOAc) 0.3; **IR** (ATR) ν 2930, 2838, 1769, 1698, 1679, 1476, 1391, 1370, 1320, 1221, 1202, 1180, 1154, 1107, 1073, 1032, 1006, 846, 794, 753 cm$^{-1}$; **$^1$H NMR** (400 MHz, CDCl$_3$) δ 7.72–7.61 (2H, m, ArH), 7.50–7.43 (2H, m, ArH), 6.93 (1H, dd, *J* = 2.6, 0.5 Hz, ArH), 6.90 (1H, dd, *J* = 9.0, 0.5 Hz, ArH), 6.66 (1H, dd, *J* = 9.0, 2.6 Hz, ArH), 4.49–4.41 (2H, m, OCH$_2$CH$_2$), 4.34 (2H, s, COCH$_2$), 4.10–4.02 (2H, m, NCH$_2$), 3.82 (3H, s, OCH$_3$), 2.35 (3H, s, CH$_3$); **$^{13}$C{$^1$H} NMR** (101 MHz, CDCl$_3$) δ 170.4 (C), 168.4 (C), 156.2 (C), 153.9 (C), 139.3 (C), 136.7 (C), 134.1 (C), 131.3 (CH), 131.0 (C), 131.0 (C), 129.2 (CH), 115.1 (CH), 112.1 (C), 111.7 (CH), 101.5 (CH), 62.3 (CH$_2$), 55.8 (CH$_3$), 42.9 (CH$_2$), 30.7 (CH$_2$), 13.8 (CH$_3$); **HRMS** (+ESI): *m/z* calcd. for $C_{22}H_{20}ClN_2O_5$ [M+H]$^+$: 427.1055, found: 427.10551.

### *N*-[(4-Hydroxyphenyl)acetyl]-1,3-oxazolidin-2-one (1q)

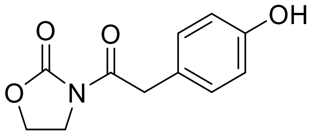


Neat TiCl$_4$ (1.1 mL, 3.2 mmol) was added dropwise to a solution of **1b** (0.5 g, 1.6 mmol) in DCM (14 mL) at 0 °C. The reaction mixture was stirred at room temperature for 30 min and quenched with sat. NH$_4$Cl (15 mL) at 0 °C. The mixture was extracted with DCM (3 × 10 mL). The combined organic layers were dried over anhydrous MgSO$_4$ and concentrated under reduced pressure. Purification of the crude product by flash column chromatography (80:20 DCM/EtOAc) afforded **1q** (258 mg, 1.2 mmol, 73% yield) as a white solid. **Mp** 181–185 °C (decomposes); **R$_f$** (80:20 DCM/EtOAc) 0.3; **IR** (ATR) ν 3336, 3028, 2924, 2854, 1793, 1682, 1519, 1385, 1370, 1274, 1219, 1119, 1046, 1022, 961, 795 cm$^{-1}$; **$^1$H NMR** (500 MHz, DMSO-*d$_6$*) δ 9.27 (1H, s, OH), 7.06–6.99 (2H, m, ArH), 6.72–6.66 (2H, m, ArH), 4.40–4.32 (2H, m, OCH$_2$), 4.05 (2H, s,

COCH̲₂), 3.91–3.84 (2H, m, NCH̲₂); **$^{13}$C{$^1$H} NMR** (126 MHz, DMSO-*d*$_6$) δ 171.2 (C), 156.2 (C), 153.7 (C), 130.6 (CH), 124.4 (C), 115.0 (CH), 62.2 ($CH_2$), 42.6 ($CH_2$), 39.6 ($CH_2$); **HRMS** (+ESI): *m/z* calcd. for $C_{11}H_{12}NO_4$ $[M+H]^+$: 222.0761, found: 222.0760.

**N-[(1H-Indol-3-yl)acetyl]-1,3-oxazolidin-2-one (1r)**

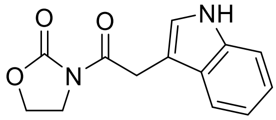


It was prepared following General Procedure 1 from (1*H*-indol-3-yl)acetic acid (1.14 g, 6.5 mmol), $Et_3N$ (0.9 mL, 6.5 mmol), PivCl (0.8 mL, 6.5 mmol), 1,3-oxazolidin-2-one (435 mg, 5.0 mmol) and *n*-BuLi (2.5 M in hexanes, 2.2 mL, 5.5 mmol). Purification of the crude product by flash column chromatography (from 70:30 to 50:50 hexanes/EtOAc) afforded **1r** (768 mg, 3.1 mmol, 63% yield) as a white solid. **Mp** 148–150 °C; **R$_f$** (50:50 hexanes/EtOAc) 0.2; **IR** (ATR) ν 3386, 2977, 2920, 1759, 1688, 1395, 1365, 1213, 1116, 1041, 1018, 959, 742 $cm^{-1}$; **$^1$H NMR** (400 MHz, DMSO-*d*$_6$) δ 10.93 (1H, s, NH̲), 7.53–7.47 (1H, m, ArH̲), 7.36 (1H, dt, *J* = 8.1, 0.9 Hz, ArH̲), 7.23 (1H, d, *J* = 2.4 Hz, CH̲NH), 7.08 (1H, ddd, *J* = 8.1, 7.0, 1.2 Hz, ArH̲), 6.98 (1H, ddd, *J* = 7.9, 7.0, 1.0 Hz, ArH̲), 4.40–4.33 (2H, m, OCH̲₂), 4.27 (2H, s, COCH̲₂), 3.93–3.86 (2H, m, NCH̲₂); **$^{13}$C{$^1$H} NMR** (101 MHz, DMSO-*d*$_6$) δ 170.8 (C), 153.8 (C), 136.0 (C), 127.4 (C), 124.4 (CH), 121.0 (CH), 118.7 (CH), 118.4 (CH), 111.4 (CH), 106.8 (C), 62.2 ($CH_2$), 42.6 ($CH_2$), 31.1 ($CH_2$); **HRMS** (+ESI): *m/z* calcd. for $C_{13}H_{13}N_2O_3$ $[M+H]^+$: 245.0921, found: 245.0921.

## 3. Copper Salts Screening

### 3.1. General Procedure

A microwave vial equipped with a magnetic stirring bar was charged with **1a** (20.5 mg, 0.10 mmol), the corresponding metal catalyst (50 µmol, 50 mol%), TEMPO (31.2 mg, 0.20 mmol), followed by the addition of a 0.2 M solution of $Et_3N$ in THF (0.5 mL, 0.10 mmol). After sealing it, the reaction mixture was stirred under nitrogen atmosphere in an oil bath at 50 °C for 16 h and then quenched with sat. $NH_4Cl$ (0.5 mL). The aqueous layer was extracted with EtOAc (3 × 0.5 mL), the combined organic extracts were dried with anhydrous $MgSO_4$ and concentrated *in vacuo*. The resulting residue was analyzed by HPLS-MS.

### 3.2. Table S1. Influence of the Catalyst on the Reaction

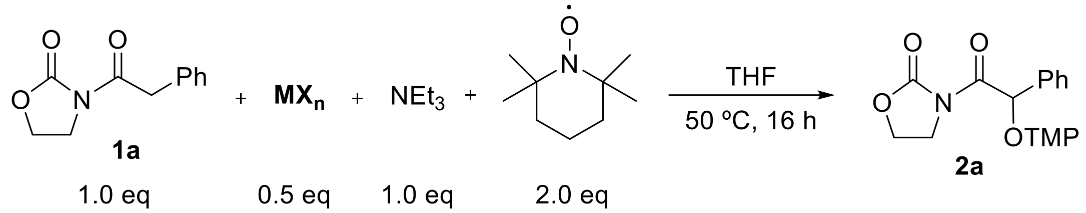


| M | $MX_n$ | Conversion (HPLC) |
|---|---|---|
| Cu(II) | $Cu(OAc)_2$ | 36% |
| | $CuCl_2$ | - |
| | $CuBr_2$ | - |
| | $Cu(OTf)_2$ | Salt reacts with TEMPO |
| | $Cu(acac)_2$ | 7% |
| Cu(I) | CuOAc | 31% |
| | CuCl | - |
| | CuBr | - |

## 4. Scope of the α-Aminoxylation Reaction

**General Procedure 2**

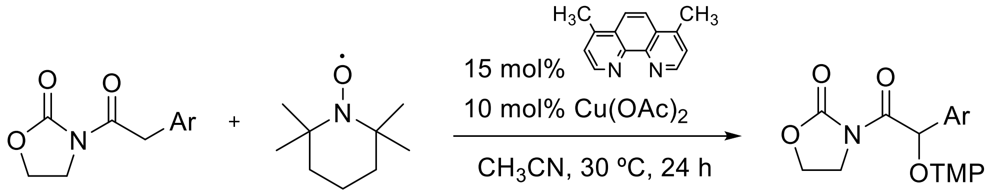


A round bottom flask equipped with a magnetic stirring bar was charged with *N*-arylacetyl-1,3-oxazolidin-2-one **1** (0.3 mmol, 1.0 equiv), anhydrous $Cu(OAc)_2$ (5.45 mg, 30 μmol, 0.10 equiv, 10 mol%), 4,7-dimethyl-1,10-phenanthroline (9.37 mg, 45 μmol, 0.15 equiv, 15 mol%), and TEMPO (93.8 mg, 0.6 mmol, 2.0 equiv), followed by the addition of acetonitrile (1.5 mL) to get a 0.2 M solution. The resultant mixture was stirred under nitrogen atmosphere at 30 °C in a water bath for 24 h and then quenched with sat. $NH_4Cl$ (1.5 mL).
The aqueous layer was extracted with EtOAc (3 × 2 mL). The combined organic extracts were dried with anhydrous $MgSO_4$ and concentrated *in vacuo*. The resulting residue was purified by column chromatography to yield the Cα aminoxylated adduct **2**.

### *N*-[Phenyl-(2,2,6,6-tetramethylpiperidin-1-yloxy)acetyl]-1,3-oxazolidin-2-one (2a)

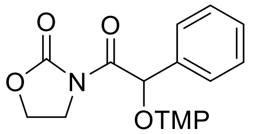


It was prepared following General Procedure 2 from **1a** (61.6 mg, 0.30 mmol), $Cu(OAc)_2$ (5.45 mg, 30 μmol, 10 mol%), 4,7-dimethyl-1,10-phenanthroline (9.37 mg, 45 μmol, 15 mol%), and TEMPO (93.8 mg, 0.60 mmol). Purification of the crude product by flash column chromatography (80:20 hexanes/EtOAc) afforded **2a** (107 mg, 0.30 mmol, 99% yield) as a white solid. **Mp** 42–46 °C; **R$_f$** (80:20 hexanes/EtOAc) 0.2; **IR** (ATR) ν 2971, 2930, 1775, 1703, 1385, 1361, 1218, 1182, 1108, 1041 $cm^{-1}$; **$^1$H NMR** (500 MHz, $CDCl_3$) δ 7.60–7.53 (2H, m, Ar<u>H</u>), 7.34–7.25 (3H, m, Ar<u>H</u>), 6.63 (1H, s, COC<u>H</u>), 4.42–4.37 (1H, m, $OC\underline{H}_xH_y$), 4.32–4.27 (1H, m, $OCH_x\underline{H}_y$), 4.04 (1H, ddd, $J$ = 11.0, 9.5, 7.6 Hz, $NC\underline{H}_xH_y$), 3.86 (1H, ddd, $J$ = 11.0, 9.3, 6.0 Hz, $NCH_x\underline{H}_y$), 1.68–1.24 (6H, m, 3 × $C\underline{H}_2$), 1.22 (3H, s, $C\underline{H}_3$), 1.10 (3H, s, $C\underline{H}_3$), 1.02 (3H, s, $C\underline{H}_3$), 0.67 (3H, s, $C\underline{H}_3$); **$^{13}$C{$^1$H} NMR** (126 MHz, $CDCl_3$) δ 172.5 (C), 153.2 (C), 138.0 (C), 128.4 (CH), 128.3 (CH), 128.2 (CH), 85.4 (CH), 62.2 ($CH_2$), 60.0 (C), 59.6 (C), 42.6 ($CH_2$), 40.1 ($CH_2$), 40.0 ($CH_2$), 33.8 ($CH_3$), 32.9 ($CH_3$), 20.2 ($CH_3$), 20.1 ($CH_3$), 17.2 ($CH_2$); **HRMS** (+ESI): *m/z* calcd. for $C_{20}H_{29}N_2O_4$ $[M+H]^+$: 361.2122, found: 361.2123.

### *N*-[2-(4-Benzyloxyphenyl)-2-(2,2,6,6-tetramethylpiperidin-1-yloxy)acetyl]-1,3-oxazolidin-2-one (2b)

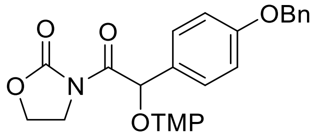


It was prepared following General Procedure 2 from **1b** (93.4 mg, 0.30 mmol), $Cu(OAc)_2$ (5.45 mg, 30 μmol, 10 mol%), 4,7-dimethyl-1,10-phenanthroline (9.37 mg, 45 μmol, 15 mol%), and TEMPO (93.8 mg, 0.60 mmol). Purification of the crude product by flash column chromatography (70:30 hexanes/EtOAc) afforded **2b** (131 mg, 0.28 mmol, 94% yield) as a white solid. **Mp** 165–166 °C; $\mathbf{R_f}$ (80:20 hexanes/EtOAc) 0.2; **IR** (ATR) ν 2923, 2866, 2854, 1770, 1706, 1509, 1388, 1216, 1178, 1113, 1001, 751, 700 $cm^{-1}$; $^{1}$**H NMR** (400 MHz, $CDCl_3$) δ 7.55–7.28 (7H, m, ArH), 6.98–6.87 (2H, m, ArH), 6.56 (1H, s, COCH), 5.04 (2H, s, $CH_2Ph$), 4.39 (1H, td, $J$ = 9.3, 6.1 Hz, $OCH_xH_y$), 4.29 (1H, td, $J$ = 9.3, 7.4 Hz, $OCH_xH_y$), 4.05 (1H, ddd, $J$ = 11.0, 9.3, 7.4 Hz, $NCH_xH_y$), 3.87 (1H, ddd, $J$ = 11.0, 9.3, 6.1 Hz, $NCH_xH_y$), 1.78–1.25 (6H, m, 3 × $CH_2$), 1.21 (3H, s, $CH_3$), 1.10 (3H, s, $CH_3$), 1.02 (3H, s, $CH_3$), 0.70 (3H, s, $CH_3$); $^{13}$**C{$^{1}$H} NMR** (101 MHz, $CDCl_3$) δ 172.7 (C), 158.8 (C), 153.2 (C), 137.0 (C), 130.4 (C), 129.7 (CH), 128.6 (CH), 128.1 (CH), 127.6 (CH), 114.6 (CH), 84.9 (CH), 70.0 ($CH_2$), 62.2 ($CH_2$), 60.0 (C), 59.5 (C), 42.6 ($CH_2$), 40.1 ($CH_2$), 40.0 ($CH_2$), 34.0 ($CH_3$), 32.9 ($CH_3$), 20.1 ($CH_3$), 20.1 ($CH_3$), 17.2 ($CH_2$); **HRMS** (+ESI): *m/z* calcd. for $C_{27}H_{35}N_2O_5$ $[M+H]^+$: 467.2540, found: 467.2539.

### *N*-[2-(4-Methoxyphenyl)-2-(2,2,6,6-tetramethylpiperidin-1-yloxy)acetyl]-1,3-oxazolidin-2-one (2c)

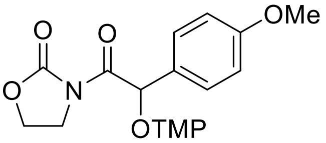


It was prepared following General Procedure 2 from **1c** (70.6 mg, 0.30 mmol), $Cu(OAc)_2$ (5.45 mg, 30 μmol, 10 mol%), 4,7-dimethyl-1,10-phenanthroline (9.37 mg, 45 μmol, 15 mol%), and TEMPO (93.8 mg, 0.60 mmol). Purification of the crude product by flash column chromatography (70:30 hexanes/EtOAc) afforded **2c** (104 mg, 0.27 mmol, 89% yield) as a white solid. **Mp** 108–111 °C; $\mathbf{R_f}$ (80:20 hexanes/EtOAc) 0.2; **IR** (ATR) ν 2962, 2925, 1774, 1702, 1606, 1509, 1385, 1361, 1251, 1223, 1173, 1107, 1036, 823 $cm^{-1}$; $^{1}$**H NMR** (400 MHz, $CDCl_3$) δ 7.53–7.44 (2H, m, ArH), 6.88–6.81 (2H, m, ArH), 6.55 (1H, s, COCH), 4.40 (1H, td, $J$ = 9.1, 6.1 Hz, $OCH_xH_y$), 4.29 (1H, td, $J$ = 9.1, 7.5 Hz, $OCH_xH_y$), 4.05 (1H, ddd, $J$ = 11.1, 9.1, 7.5 Hz, $NCH_xH_y$), 3.91–3.81 (1H, m, $NCH_xH_y$), 3.79 (3H, s, $OCH_3$), 1.63–1.24 (6H, m, 3 × $CH_2$), 1.21 (3H, s, $CH_3$), 1.09 (3H, s, $CH_3$), 1.01 (3H, s, $CH_3$), 0.69 (3H, s, $CH_3$); $^{13}$**C{$^{1}$H} NMR** (101 MHz, $CDCl_3$) δ 172.6 (C), 159.5 (C), 153.1 (C), 130.1 (C), 129.6 (CH), 113.6 (CH), 84.9 (CH), 62.2 ($CH_2$), 60.0 (C), 59.4 (C), 55.2 ($CH_3$), 42.5 ($CH_2$), 40.1 ($CH_2$), 40.0 ($CH_2$), 34.0 ($CH_3$), 32.9 ($CH_3$), 20.1 ($CH_3$), 20.1 ($CH_3$), 17.2 ($CH_2$); **HRMS** (+ESI): *m/z* calcd. for $C_{21}H_{31}N_2O_5$ $[M+H]^+$: 391.2227, found: 391.2217.

***N*-[2-(3-Methoxyphenyl)-2-(2,2,6,6-tetramethylpiperidin-1-yloxy)acetyl]-1,3-oxazolidin-2-one (2d)**

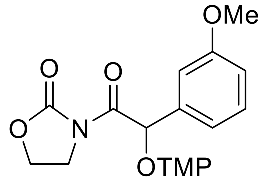


It was prepared following General Procedure 2 from **1d** (70.6 mg, 0.30 mmol), $Cu(OAc)_2$ (5.45 mg, 30 μmol, 10 mol%), 4,7-dimethyl-1,10-phenanthroline (9.37 mg, 45 μmol, 15 mol%), and TEMPO (93.8 mg, 0.60 mmol). Purification of the crude product by flash column chromatography (80:20 hexanes/EtOAc) afforded **2d** (115 mg, 0.29 mmol, 98% yield) as a white solid. **Mp** 48–51 °C; **$R_f$** (70:30 hexanes/EtOAc) 0.4; **IR** (ATR) ν 2965, 2927, 1775, 1704, 1598, 1585, 1487, 1382, 1361, 1253, 1207, 1106, 1038, 710 $cm^{-1}$; **$^1$H NMR** (500 MHz, $CDCl_3$) δ 7.22 (1H, t, *J* = 8.1 Hz, ArH), 7.18–7.12 (2H, m, ArH), 6.83 (1H, ddd, *J* = 8.1, 2.7, 1.1 Hz, ArH), 6.62 (1H, s, COCH), 4.40 (1H, ddd, *J* = 9.5, 8.8, 6.0 Hz, $OCH_xH_y$), 4.31 (1H, td, *J* = 9.5, 7.6 Hz, $OCH_xH_y$), 4.05 (1H, ddd, *J* = 11.1, 9.5, 7.6 Hz, $NCH_xH_y$), 3.87 (1H, ddd, *J* = 11.1, 9.5, 6.0 Hz, $NCH_xH_y$), 3.81 (3H, s, $OCH_3$), 1.50–1.24 (6H, m, 3 × $CH_2$), 1.21 (3H, s, $CH_3$), 1.09 (3H, s, $CH_3$), 1.04 (3H, s, $CH_3$), 0.71 (3H, s, $CH_3$); **$^{13}C\{^1H\}$ NMR** (126 MHz, $CDCl_3$) δ 172.4 (C), 159.5 (C), 153.2 (C), 139.4 (C), 129.3 (CH), 120.5 (CH), 114.3 (CH), 113.0 (CH), 85.2 (CH), 62.2 ($CH_2$), 60.0 (C), 59.6 (C), 55.4 ($CH_3$), 42.6 ($CH_2$), 40.1 ($CH_2$), 40.0 ($CH_2$), 33.8 ($CH_3$), 32.9 ($CH_3$), 20.2 ($CH_3$), 20.1 ($CH_3$), 17.2 ($CH_2$); **HRMS** (+ESI): *m/z* calcd. for $C_{21}H_{31}N_2O_5$ $[M+H]^+$: 391.2227, found: 391.2229.

***N*-[2-(2-Methoxyphenyl)-2-(2,2,6,6-tetramethylpiperidin-1-yloxy)acetyl]-1,3-oxazolidin-2-one (2e)**

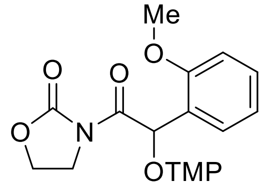


It was prepared following General Procedure 2 from **1e** (70.6 mg, 0.30 mmol), $Cu(OAc)_2$ (5.45 mg, 30 μmol, 10 mol%), 4,7-dimethyl-1,10-phenanthroline (9.37 mg, 45 μmol, 15 mol%), and TEMPO (93.8 mg, 0.60 mmol). Purification of the crude product by flash column chromatography (70:30 hexanes/EtOAc) afforded **2e** (71 mg, 0.18 mmol, 61% yield) as a white solid. **Mp** 136–140 °C; **$R_f$** (70:30 hexanes/EtOAc) 0.2; **IR** (ATR) ν 2977, 2926, 1794, 1691, 1487, 1382, 1373, 1291, 1244, 1188, 1103, 1040, 1022, 755 $cm^{-1}$; **$^1$H NMR** (400 MHz, $CDCl_3$) δ 7.71 (1H, dd, *J* = 7.4, 1.7 Hz, ArH), 7.31–7.21 (1H, m, ArH), 7.05–6.98 (2H, m, COCH, ArH), 6.82 (1H, dd, *J* = 8.2, 1.1 Hz, ArH), 4.39 (1H, td, *J* = 9.0, 5.5 Hz, $OCH_xH_y$), 4.29 (1H, td, *J* = 9.0, 8.1 Hz, $OCH_xH_y$), 4.02 (1H, ddd, *J* = 11.0, 9.0, 8.1 Hz, $NCH_xH_y$), 3.92 (1H, ddd, *J* = 11.0, 9.0, 5.5 Hz, $NCH_xH_y$), 3.78 (3H, s, $OCH_3$), 1.70–1.23 (6H, m, 3 × $CH_2$), 1.20 (3H, s, $CH_3$), 1.10 (6H, s, 2 × $CH_3$), 0.86 (3H, s, $CH_3$); **$^{13}C\{^1H\}$ NMR** (101 MHz, $CDCl_3$) δ 173.1 (C), 156.2 (C), 152.4 (C), 128.9 (CH), 128.6 (CH), 127.0 (C), 120.9 (CH), 110.4 (CH), 78.6 (CH), 61.8 ($CH_2$), 60.2 (C), 59.6 (C), 55.6 ($CH_3$), 42.9 ($CH_2$), 40.1 ($CH_2$), 40.0 ($CH_2$), 32.9 ($CH_3$), 32.5 ($CH_3$), 20.3

($CH_3$), 20.2 ($CH_3$), 17.2 ($CH_2$); **HRMS** (+ESI): *m/z* calcd. for $C_{21}H_{31}N_2O_5$ $[M+H]^+$: 391.2227, found: 391.2233.

**N-[2-(2,2,6,6-Tetramethylpiperidin-1-yloxy)-2-(p-tolyl)acetyl]-1,3-oxazolidin-2-one (2f)**

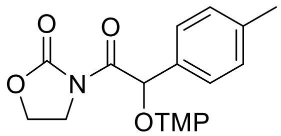

It was prepared following General Procedure 2 from **1f** (65.8 mg, 0.30 mmol), $Cu(OAc)_2$ (5.45 mg, 30 μmol, 10 mol%), 4,7-dimethyl-1,10-phenanthroline (9.37 mg, 45 μmol, 15 mol%), and TEMPO (93.8 mg, 0.60 mmol). Purification of the crude product by flash column chromatography (80:20 hexanes/EtOAc) afforded **2f** (99 mg, 0.26 mmol, 88% yield) as a white solid. **Mp** 125–127 °C; **$R_f$** (80:20 hexanes/EtOAc) 0.2; **IR** (ATR) ν 2958, 2927, 2870, 1774, 1704, 1386, 1361, 1224, 1186, 1104, 1042, 813, 713 $cm^{-1}$; **$^1$H NMR** (400 MHz, $CDCl_3$) δ 7.49–7.41 (2H, m, ArH), 7.17–7.09 (2H, m, ArH), 6.59 (1H, s, COCH), 4.39 (1H, ddd, *J* = 9.4, 8.8, 6.0 Hz, $OCH_xH_y$), 4.28 (1H, td, *J* = 9.4, 7.5 Hz, $OCH_xH_y$), 4.04 (1H, ddd, *J* = 11.0, 9.4, 7.5 Hz, $NCH_xH_y$), 3.85 (1H, ddd, *J* = 11.0, 9.4, 6.0 Hz, $NCH_xH_y$), 2.33 (3H, s, $C{=}CCH_3$), 1.64–1.25 (6H, m, 3 × $CH_2$), 1.21 (3H, s, $CH_3$), 1.09 (3H, s, $CH_3$), 1.02 (3H, s, $CH_3$), 0.69 (3H, s, $CH_3$); **$^{13}C\{^1H\}$ NMR** (101 MHz, $CDCl_3$) δ 172.7 (C), 153.2 (C), 138.0 (C), 135.0 (C), 129.0 (CH), 128.2 (CH), 85.2 (CH), 62.2 ($CH_2$), 60.0 (C), 59.5 (C), 42.6 ($CH_2$), 40.1 ($CH_2$), 40.0 ($CH_2$), 33.9 ($CH_3$), 32.9 ($CH_3$), 21.3 ($CH_3$), 20.2 ($CH_3$), 20.1 ($CH_3$), 17.2 ($CH_2$); **HRMS** (+ESI): *m/z* calcd. for $C_{21}H_{31}N_2O_4$ $[M+H]^+$: 375.2278, found: 375.2278.

**N-[2-(2,2,6,6-Tetramethylpiperidin-1-yloxy)-2-(m-tolyl)acetyl]-1,3-oxazolidin-2-one (2g)**

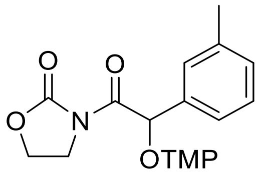

It was prepared following General Procedure 2 from **1g** (65.8 mg, 0.30 mmol), $Cu(OAc)_2$ (5.45 mg, 30 μmol, 10 mol%), 4,7-dimethyl-1,10-phenanthroline (9.37 mg, 45 μmol, 15 mol%), and TEMPO (93.8 mg, 0.60 mmol). Purification of the crude product by flash column chromatography (80:20 hexanes/EtOAc) afforded **2g** (110 mg, 0.29 mmol, 98% yield) as a white solid. **Mp** 112–115 °C; **$R_f$** (80:20 hexanes/EtOAc) 0.2; **IR** (ATR) ν 2968, 2925, 2873, 1774, 1706, 1384, 1361, 1342, 1206, 1102, 1039, 711 $cm^{-1}$; **$^1$H NMR** (500 MHz, $CDCl_3$) δ 7.42–7.34 (2H, m, ArH), 7.20 (1H, td, *J* = 7.5, 0.8 Hz, ArH), 7.13–7.07 (1H, m, ArH), 6.61 (1H, s, COCH), 4.40 (1H, td, *J* = 9.3, 6.0 Hz, $OCH_xH_y$), 4.30 (1H, td, *J* = 9.3, 7.6 Hz, $OCH_xH_y$), 4.05 (1H, ddd, *J* = 11.1, 9.3, 7.6 Hz, $NCH_xH_y$), 3.87 (1H, ddd, *J* = 11.1, 9.3, 6.0 Hz, $NCH_xH_y$), 2.34 (3H, s, $C{=}CCH_3$), 1.66–1.25 (6H, m, 3 × $CH_2$), 1.22 (3H, s, $CH_3$), 1.10 (3H, s, $CH_3$), 1.03 (3H, s, $CH_3$), 0.69 (3H, s, $CH_3$); **$^{13}C\{^1H\}$ NMR** (126 MHz, $CDCl_3$) δ 172.6 (C), 153.1 (C), 137.9 (C), 137.8 (C), 129.0 (CH), 128.6 (CH), 128.1 (CH), 125.3 (CH), 85.3 (CH), 62.1 ($CH_2$), 59.9 (C), 59.5 (C), 42.5 ($CH_2$), 40.1 ($CH_2$), 40.0 ($CH_2$), 33.8 ($CH_3$), 32.9 ($CH_3$), 21.4 ($CH_3$), 20.1 ($CH_3$), 20.1 ($CH_3$), 17.1 ($CH_2$); **HRMS** (+ESI): *m/z* calcd. for $C_{21}H_{31}N_2O_4$ $[M+H]^+$: 375.2278, found: 375.2276.

**_N_-[2-(2,2,6,6-Tetramethylpiperidin-1-yloxy)-2-(_o_-tolyl)acetyl]-1,3-oxazolidin-2-one (2h)**

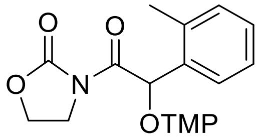


It was prepared following General Procedure 2 from **1h** (65.8 mg, 0.30 mmol), $Cu(OAc)_2$ (5.45 mg, 30 µmol, 10 mol%), 4,7-dimethyl-1,10-phenanthroline (9.37 mg, 45 µmol, 15 mol%), and TEMPO (93.8 mg, 0.60 mmol). Purification of the crude product by flash column chromatography (80:20 hexanes/EtOAc) afforded **2h** (75 mg, 0.20 mmol, 67% yield) as an orange oil. $\mathbf{R_f}$ (80:20 hexanes/EtOAc) 0.2; **IR** (ATR) ν 2974, 2927, 1775, 1702, 1381, 1360, 1196, 1109, 1040, 755 $cm^{-1}$; **$^1$H NMR** (400 MHz, $CDCl_3$) δ 7.61 (1H, dd, $J$ = 7.2, 1.9 Hz, Ar$\underline{H}$), 7.23–7.10 (3H, m, Ar$\underline{H}$), 6.83 (1H, s, COC$\underline{H}$), 4.40 (1H, ddd, $J$ = 9.5, 8.8, 5.1 Hz, OC$\underline{H}_x$H$_y$), 4.28 (1H, q, $J$ ≈ 8.8 Hz, OCH$_x\underline{H}_y$), 4.08 (1H, ddd, $J$ = 11.1, 9.5, 8.5 Hz, NC$\underline{H}_x$H$_y$), 3.86 (1H, ddd, $J$ = 11.1, 9.0, 5.1 Hz, NCH$_x\underline{H}_y$), 2.53 (3H, s, C=CC$\underline{H}_3$), 1.63–1.25 (6H, m, 3 × C$\underline{H}_2$), 1.21 (3H, s, C$\underline{H}_3$), 1.12 (3H, s, C$\underline{H}_3$), 1.02 (3H, s, C$\underline{H}_3$), 0.67 (3H, s, C$\underline{H}_3$); **$^{13}$C{$^1$H} NMR** (101 MHz, $CDCl_3$) δ 172.2 (C), 152.9 (C), 137.5 (C), 136.1 (C), 130.8 (CH), 129.7 (CH), 128.1 (CH), 125.8 (CH), 83.7 (CH), 62.0 ($CH_2$), 60.0 (C), 59.6 (C), 42.9 ($CH_2$), 40.0 ($CH_2$), 39.9 ($CH_2$), 33.0 ($CH_3$), 33.0 ($CH_3$), 20.4 ($CH_3$), 20.2 ($CH_3$), 20.1 ($CH_3$), 17.1 ($CH_2$); **HRMS** (+ESI): _m/z_ calcd. for $C_{21}H_{31}N_2O_4$ $[M+H]^+$: 375.2278, found: 375.2289.

**_N_-[2-(4-Chlorophenyl)-2-(2,2,6,6-tetramethylpiperidin-1-yloxy)acetyl]-1,3-oxazolidin-2-one (2i)**

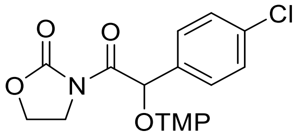


It was prepared following General Procedure 2 from **1i** (71.9 mg, 0.30 mmol), $Cu(OAc)_2$ (5.45 mg, 30 µmol, 10 mol%), 4,7-dimethyl-1,10-phenanthroline (9.37 mg, 45 µmol, 15 mol%), and TEMPO (93.8 mg, 0.60 mmol). Purification of the crude product by flash column chromatography (80:20 hexanes/EtOAc) afforded **2i** (114 mg, 0.29 mmol, 96% yield) as a white solid. **Mp** 52–55 $^{o}$C; $\mathbf{R_f}$ (70:30 hexanes/EtOAc) 0.5; **IR** (ATR) ν 2971, 2927, 1775, 1704, 1488, 1384, 1361, 1208, 1182, 1108, 1039, 817 $cm^{-1}$; **$^1$H NMR** (400 MHz, $CDCl_3$) δ 7.56–7.47 (2H, m, Ar$\underline{H}$), 7.32–7.27 (2H, m, Ar$\underline{H}$), 6.60 (1H, s, COC$\underline{H}$), 4.42 (1H, td, $J$ = 9.2, 6.2 Hz, OC$\underline{H}_x$H$_y$), 4.33 (1H, td, $J$ = 9.2, 7.3 Hz, OCH$_x\underline{H}_y$), 4.05 (1H, ddd, $J$ = 11.1, 9.2, 7.3 Hz, NC$\underline{H}_x$H$_y$), 3.87 (1H, ddd, $J$ = 11.1, 9.2, 6.2 Hz, NCH$_x\underline{H}_y$), 1.63–1.24 (6H, m, 3 × C$\underline{H}_2$), 1.20 (3H, s, C$\underline{H}_3$), 1.08 (3H, s, C$\underline{H}_3$), 1.01 (3H, s, C$\underline{H}_3$), 0.67 (3H, s, C$\underline{H}_3$); **$^{13}$C{$^1$H} NMR** (101 MHz, $CDCl_3$) δ 172.2 (C), 153.2 (C), 136.4 (C), 134.2 (C), 129.6 (CH), 128.6 (CH), 84.7 (CH), 62.3 ($CH_2$), 60.1 (C), 59.6 (C), 42.6 ($CH_2$), 40.1 ($CH_2$), 40.0 ($CH_2$), 34.0 ($CH_3$), 32.9 ($CH_3$), 20.1 ($CH_3$), 20.1 ($CH_3$), 17.1 ($CH_2$); **HRMS** (+ESI): _m/z_ calcd. for $C_{20}H_{28}ClN_2O_4$ $[M+H]^+$: 395.1732, found: 395.1730.

### *N*-[2-(4-Nitrophenyl)-2-(2,2,6,6-tetramethylpiperidin-1-yloxy)acetyl]-1,3-oxazolidin-2-one (2j)

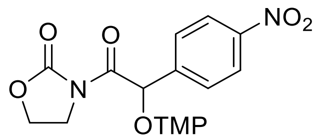

It was prepared following General Procedure 2 from **1j** (75.1 mg, 0.30 mmol), $Cu(OAc)_2$ (5.45 mg, 30 μmol, 10 mol%), 4,7-dimethyl-1,10-phenanthroline (9.37 mg, 45 μmol, 15 mol%), and TEMPO (93.8 mg, 0.60 mmol). Purification of the crude product by flash column chromatography (80:20 hexanes/EtOAc) afforded **2j** (113 mg, 0.28 mmol, 93% yield) as a light-yellow solid. **Mp** 180–182 °C; $\mathbf{R_f}$ (80:20 hexanes/EtOAc) 0.2; **IR** (ATR) ν 2993, 2968, 2925, 1770, 1704, 1514, 1386, 1343, 1220, 1106, 1037, 846, 727 $cm^{-1}$; $^1$**H NMR** (400 MHz, $CDCl_3$) δ 8.23–8.14 (2H, m, Ar$\underline{H}$), 7.81–7.72 (2H, m, Ar$\underline{H}$), 6.75 (1H, s, COC$\underline{H}$), 4.45 (1H, td, *J* = 9.2, 6.4 Hz, OC$\underline{H}_x$H$_y$), 4.36 (1H, td, *J* = 9.2, 7.1 Hz, OCH$_x\underline{H}_y$), 4.06 (1H, ddd, *J* = 11.1, 9.2, 7.1 Hz, NC$\underline{H}_x$H$_y$), 3.88 (1H, ddd, *J* = 11.1, 9.2, 6.4 Hz, NCH$_x\underline{H}_y$), 1.62–1.24 (6H, m, 3 × C$\underline{H}_2$), 1.21 (3H, s, C$\underline{H}_3$), 1.09 (3H, s, C$\underline{H}_3$), 1.02 (3H, s, C$\underline{H}_3$), 0.64 (3H, s, C$\underline{H}_3$); $^{13}$**C{$^1$H} NMR** (101 MHz, $CDCl_3$) δ 171.4 (C), 153.3 (C), 147.9 (C), 144.9 (C), 129.0 (CH), 123.7 (CH), 84.6 (CH), 62.5 ($CH_2$), 60.2 (C), 59.8 (C), 42.6 ($CH_2$), 40.1 ($CH_2$), 40.0 ($CH_2$), 33.9 ($CH_3$), 32.9 ($CH_3$), 20.2 ($CH_3$), 17.1 ($CH_2$); **HRMS** (+ESI): *m/z* calcd. for $C_{20}H_{28}N_3O_6$ $[M+H]^+$: 406.1973, found: 406.1974.

### *N*-[2-(2,2,6,6-Tetramethylpiperidin-1-yloxy)-2-(4-trifluoromethylphenyl)acetyl]-1,3-oxazolidin-2-one (2k)

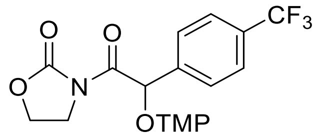

It was prepared following General Procedure 2 from **1k** (82.0 mg, 0.30 mmol), $Cu(OAc)_2$ (5.45 mg, 30 μmol, 10 mol%), 4,7-dimethyl-1,10-phenanthroline (9.37 mg, 45 μmol, 15 mol%), and TEMPO (93.8 mg, 0.60 mmol). Purification of the crude product by flash column chromatography (80:20 hexanes/EtOAc) afforded **2k** (122 mg, 0.28 mmol, 95% yield) as a white solid. **Mp** 50–52 °C; $\mathbf{R_f}$ (80:20 hexanes/EtOAc) 0.2; **IR** (ATR) ν 2971, 2930, 1775, 1705, 1385, 1362, 1320, 1218, 1163, 1108, 1065, 823, 710 $cm^{-1}$; $^1$**H NMR** (400 MHz, $CDCl_3$) δ 7.74–7.67 (2H, m, Ar$\underline{H}$), 7.62–7.54 (2H, m, Ar$\underline{H}$), 6.71 (1H, s, COC$\underline{H}$), 4.43 (1H, ddd, *J* = 9.4, 8.8, 6.3 Hz, OC$\underline{H}_x$H$_y$), 4.34 (1H, td, *J* = 9.4, 7.3 Hz, OCH$_x\underline{H}_y$), 4.05 (1H, ddd, *J* = 11.1, 9.4, 7.3 Hz, NC$\underline{H}_x$H$_y$), 3.87 (1H, ddd, *J* = 11.1, 9.4, 6.3 Hz, NCH$_x\underline{H}_y$), 1.62–1.24 (6H, m, 3 × C$\underline{H}_2$), 1.22 (3H, s, C$\underline{H}_3$), 1.10 (3H, s, C$\underline{H}_3$), 1.02 (3H, s, C$\underline{H}_3$), 0.65 (3H, s, C$\underline{H}_3$); $^{13}$**C{$^1$H} NMR** (101 MHz, $CDCl_3$) δ 171.9 (C), 153.3 (C), 141.8 (C), 130.4 ($\underline{C}CF_3$, q, $^2J$ = 32.3 Hz), 128.5 (CH), 125.4 ($\underline{C}HCCF_3$, q, $^3J$ = 3.8 Hz), 124.2 ($CF_3$, q, $^1J$ = 272.2 Hz), 84.9 (CH), 62.4 ($CH_2$), 60.2 (C), 59.8 (C), 42.6 ($CH_2$), 40.2 ($CH_2$), 40.0 ($CH_2$), 33.9 ($CH_3$), 32.9 ($CH_3$), 20.2 ($CH_3$), 20.2 ($CH_3$), 17.2 ($CH_2$); **HRMS** (+ESI): *m/z* calcd. for $C_{21}H_{28}F_3N_2O_4$ $[M+H]^+$: 429.1996, found: 429.1998.

***N*-[2-(Benzo[*d*][1,3]dioxol-5-yl)-2-(2,2,6,6-tetramethylpiperidin-1-yloxy)acetyl]-1,3-oxazolidin-2-one (2l)**

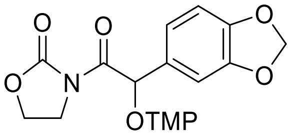


It was prepared following General Procedure 2 from **1l** (74.8 mg, 0.30 mmol), $Cu(OAc)_2$ (5.45 mg, 30 μmol, 10 mol%), 4,7-dimethyl-1,10-phenanthroline (9.37 mg, 45 μmol, 15 mol%), and TEMPO (93.8 mg, 0.60 mmol). Purification of the crude product by flash column chromatography (from 80:20 to 70:30 hexanes/EtOAc) afforded **2l** (121 mg, 0.30 mmol, 99% yield) as a white solid. **Mp** 127–130 °C; $\mathbf{R_f}$ (70:30 hexanes/EtOAc) 0.3; **IR** (ATR) ν 2962, 2918, 1780, 1694, 1490, 1479, 1386, 1361, 1252, 1220, 1205, 1112, 1041, 710 $cm^{-1}$; $^1$**H NMR** (400 MHz, $CDCl_3$) δ 7.08 (1H, d, *J* = 1.7 Hz, Ar<u>H</u>), 7.04 (1H, dd, *J* = 8.0, 1.7 Hz, Ar<u>H</u>), 6.74 (1H, d, *J* = 8.0 Hz, Ar<u>H</u>), 6.53 (1H, s, COC<u>H</u>), 5.96–5.90 (2H, m, OC<u>H</u>$_2$O), 4.41 (1H, td, *J* = 9.2, 6.1 Hz, OC<u>H</u>$_x$H$_y$), 4.32 (1H, td, *J* = 9.2, 7.4 Hz, OCH$_x$<u>H</u>$_y$), 4.05 (1H, ddd, *J* = 11.0, 9.2, 7.4 Hz, NC<u>H</u>$_x$H$_y$), 3.88 (1H, ddd, *J* = 11.0, 9.2, 6.1 Hz, NCH$_x$<u>H</u>$_y$), 1.63–1.24 (6H, m, 3 × C<u>H</u>$_2$), 1.20 (3H, s, C<u>H</u>$_3$), 1.07 (3H, s, C<u>H</u>$_3$), 1.03 (3H, s, C<u>H</u>$_3$), 0.74 (3H, s, C<u>H</u>$_3$); $^{13}$**C{$^1$H} NMR** (101 MHz, $CDCl_3$) δ 172.5 (C), 153.1 (C), 147.6 (C), 147.5 (C), 131.8 (C), 122.1 (CH), 108.5 (CH), 108.0 (CH), 101.0 ($CH_2$), 84.9 (CH), 62.2 ($CH_2$), 60.0 (C), 59.5 (C), 42.6 ($CH_2$), 40.1 ($CH_2$), 40.0 ($CH_2$), 34.0 ($CH_3$), 32.9 ($CH_3$), 20.1 ($CH_3$), 17.2 ($CH_2$); **HRMS** (+ESI): *m/z* calcd. for $C_{21}H_{29}N_2O_6$ $[M+H]^+$: 405.2020, found: 405.2012.

***N*-[2-(3,4-Dimethoxyphenyl)-2-(2,2,6,6-tetramethylpiperidin-1-yloxy)acetyl]-1,3-oxazolidin-2-one (2m)**

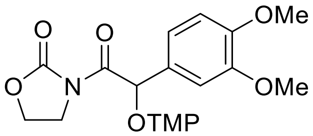


It was prepared following General Procedure 2 from **1m** (79.6 mg, 0.30 mmol), $Cu(OAc)_2$ (5.45 mg, 30 μmol, 10 mol%), 4,7-dimethyl-1,10-phenanthroline (9.37 mg, 45 μmol, 15 mol%), and TEMPO (93.8 mg, 0.60 mmol). Purification of the crude product by flash column chromatography (from 70:30 to 50:50 hexanes/EtOAc) afforded **2m** (126 mg, 0.30 mmol, 99% yield) as a yellow solid. **Mp** 148–150 °C; $\mathbf{R_f}$ (70:30 hexanes/EtOAc) 0.2; **IR** (ATR) ν 2993, 2961, 2928, 2872, 2838, 1780, 1769, 1701, 1515, 1388, 1364, 1346, 1260, 1238, 1142, 1113, 1042, 1027, 760, 710 $cm^{-1}$; $^1$**H NMR** (400 MHz, $CDCl_3$) δ 7.14–7.07 (2H, m, Ar<u>H</u>), 6.79 (1H, d, *J* = 8.8 Hz, Ar<u>H</u>), 6.53 (1H, s, COC<u>H</u>), 4.40 (1H, ddd, *J* = 9.4, 8.8, 6.1 Hz, OC<u>H</u>$_x$H$_y$), 4.30 (1H, td, *J* = 9.4, 7.5 Hz, OCH$_x$<u>H</u>$_y$), 4.05 (1H, ddd, *J* = 11.0, 9.4, 7.5 Hz, NC<u>H</u>$_x$H$_y$), 3.94–3.81 (7H, m, 2 × OC<u>H</u>$_3$, NCH$_x$<u>H</u>$_y$), 1.67–1.23 (6H, m, 3 × C<u>H</u>$_2$), 1.21 (3H, s, C<u>H</u>$_3$), 1.09 (3H, s, C<u>H</u>$_3$), 1.02 (3H, s, C<u>H</u>$_3$), 0.68 (3H, s, C<u>H</u>$_3$); $^{13}$**C{$^1$H} NMR** (101 MHz, $CDCl_3$) δ 172.5 (C), 153.2 (C), 149.0 (C), 148.8 (C), 130.6 (C), 121.0 (CH), 111.1 (CH), 110.6 (CH), 85.2 (CH), 62.2 ($CH_2$), 60.0 (C), 59.5 (C), 56.1 ($CH_3$), 55.8 ($CH_3$), 42.6 ($CH_2$), 40.1 ($CH_2$), 34.0 ($CH_3$), 32.9 ($CH_3$), 20.2 ($CH_3$), 20.1 ($CH_3$), 17.2 ($CH_2$); **HRMS** (+ESI): *m/z* calcd. for $C_{22}H_{33}N_2O_6$ $[M+H]^+$: 421.2333, found: 421.2336.

**$N$-[2-(11-Oxo-6,11-dihydrodibenzo[$b$,$e$]oxepin-2-yl)-2-(2,2,6,6-tetramethylpiperidin-1-yloxy)acetyl]-1,3-oxazolidin-2-one (2n)**

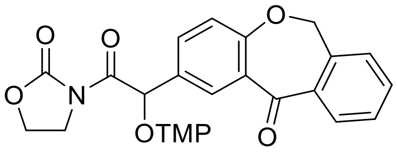


It was prepared following General Procedure 2 from **1n** (101 mg, 0.30 mmol), $Cu(OAc)_2$ (5.45 mg, 30 μmol, 10 mol%), 4,7-dimethyl-1,10-phenanthroline (9.37 mg, 45 μmol, 15 mol%), and TEMPO (93.8 mg, 0.60 mmol). Purification of the crude product by flash column chromatography (70:30 hexanes/EtOAc) afforded **2n** (146 mg, 0.30 mmol, 99% yield) as a white solid. **Mp** 90–94 °C; **R$_f$** (70:30 hexanes/EtOAc) 0.2; **IR** (ATR) ν 2968, 2925, 2863, 1771, 1703, 1645, 1606, 1481, 1381, 1361, 1299, 1238, 1204, 1104, 1039, 1008, 828, 758 cm$^{-1}$; **$^1$H NMR** (500 MHz, $CDCl_3$) δ 8.38 (1H, d, $J$ = 2.3 Hz, ArH), 7.90 (1H, dd, $J$ = 7.7, 1.4 Hz, ArH), 7.71 (1H, dd, $J$ = 8.5, 2.3 Hz, ArH), 7.55 (1H, td, $J$ = 7.5, 1.4 Hz, ArH), 7.47 (1H, td, $J$ = 7.7, 1.3 Hz, ArH), 7.35 (1H, dd, $J$ = 7.5, 1.3 Hz, ArH), 7.02 (1H, d, $J$ = 8.5 Hz, ArH), 6.67 (1H, s, COCH), 5.18 (2H, s, $OCH_2$), 4.41 (1H, td, $J$ = 9.2, 6.3 Hz, $OCH_xH_y$), 4.32 (1H, td, $J$ = 9.2, 7.3 Hz, $OCH_xH_y$), 4.06 (1H, ddd, $J$ = 11.1, 9.2, 7.3 Hz, $NCH_xH_y$), 3.88 (1H, ddd, $J$ = 11.1, 9.2, 6.3 Hz, $NCH_xH_y$), 1.59–1.24 (6H, m, 3 × $CH_2$), 1.22 (3H, s, $CH_3$), 1.10 (3H, s, $CH_3$), 1.03 (3H, s, $CH_3$), 0.71 (3H, s, $CH_3$); **$^{13}$C{$^1$H} NMR** (126 MHz, $CDCl_3$) δ 190.6 (C), 172.2 (C), 161.1 (C), 153.1 (C), 140.7 (C), 135.4 (C), 135.2 (CH), 132.7 (CH), 131.9 (C), 131.6 (CH), 129.6 (CH), 129.3 (CH), 127.8 (CH), 125.2 (C), 120.9 (CH), 84.3 (CH), 73.6 ($CH_2$), 62.2 ($CH_2$), 60.0 (C), 59.7 (C), 42.5 ($CH_2$), 40.1 ($CH_2$), 40.0 ($CH_2$), 34.1 ($CH_3$), 32.9 ($CH_3$), 20.2 ($CH_3$), 20.2 ($CH_3$), 17.2 ($CH_2$); **HRMS** (+ESI): $m/z$ calcd. for $C_{28}H_{33}N_2O_6$ [M+H]$^+$: 493.2333, found: 493.2334.

**$N$-[2-($N$'-*tert*-Butyloxocarbonyl-1$H$-indol-3-yl)-2-(2,2,6,6-tetramethylpiperidin-1-yloxy)acetyl]-1,3-oxazolidin-2-one (2o)**

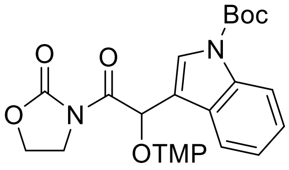


It was prepared following General Procedure 2 from **1o** (103 mg, 0.30 mmol), $Cu(OAc)_2$ (5.45 mg, 30 μmol, 10 mol%), 4,7-dimethyl-1,10-phenanthroline (9.37 mg, 45 μmol, 15 mol%), and TEMPO (93.8 mg, 0.60 mmol). Purification of the crude product by flash column chromatography (80:20 hexanes/EtOAc) afforded **2o** (121 mg, 0.24 mmol, 81% yield) as a white solid. **Mp** 148–150 °C; **R$_f$** (80:20 hexanes/EtOAc) 0.2; **IR** (ATR) ν 2971, 2927, 1779, 1731, 1709, 1450, 1360, 1234, 1153, 1084, 746 cm$^{-1}$; **$^1$H NMR** (500 MHz, $CDCl_3$) δ 8.11 (1H, d, $J$ = 8.2 Hz, ArH), 8.04–7.99 (1H, m, ArH), 7.72 (1H, s, CHNBoc), 7.33–7.22 (2H, m, ArH), 6.79 (1H, s, COCH), 4.39 (1H, td, $J$ = 9.2, 6.1 Hz, $OCH_xH_y$), 4.29 (1H, td, $J$ = 9.2, 7.4 Hz, $OCH_xH_y$), 4.05 (1H, ddd, $J$ = 11.0, 9.2, 7.4 Hz, $NCH_xH_y$), 3.84 (1H, ddd, $J$ = 11.0, 9.2, 6.1 Hz, $NCH_xH_y$), 1.66 (9H, s, 3 × $CH_3$), 1.58–1.28 (6H, m, 3 × $CH_2$), 1.26 (3H, s, $CH_3$), 1.14 (3H, s, $CH_3$), 0.97 (3H, s, $CH_3$), 0.77 (3H, s, $CH_3$); **$^{13}$C{$^1$H} NMR** (126 MHz, $CDCl_3$) δ 171.2 (C), 153.0 (C), 149.7 (C), 135.5 (C), 128.8 (C), 125.2 (CH), 124.5 (CH), 122.8 (CH), 122.3 (CH), 117.0 (C), 114.9 (CH), 83.9 (C), 80.8 (CH), 62.2 ($CH_2$), 60.0 (C), 59.5 (C), 42.5 ($CH_2$), 40.1 ($CH_2$), 40.0 ($CH_2$),

33.9 ($CH_3$), 33.0 ($CH_3$), 28.2 ($CH_3$), 20.2 ($CH_3$), 20.1 ($CH_3$), 17.2 ($CH_2$); **HRMS** (+ESI): *m/z* calcd. for $C_{27}H_{38}N_3O_6$ $[M+H]^+$: 500.2755, found: 500.2752.

### *N*-[2-(*N'*-(4-Chlorobenzoyl)-5-methoxy-2-methyl-1*H*-indol-3-yl)-2-(2,2,6,6-tetramethylpiperidin-1-yloxy)acetyl)-1,3-oxazolidin-2-one (2p)

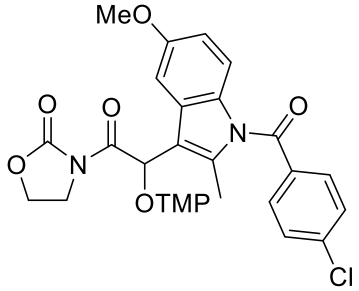


It was prepared following General Procedure 2 from **1p** (128 mg, 0.30 mmol), $Cu(OAc)_2$ (5.45 mg, 30 μmol, 10 mol%), 4,7-dimethyl-1,10-phenanthroline (9.37 mg, 45 μmol, 15 mol%), and TEMPO (93.8 mg, 0.60 mmol). Purification of the crude product by flash column chromatography (80:20 hexanes/EtOAc) afforded **2p** (162 mg, 0.28 mmol, 93% yield) as a yellow solid. **Mp** 171–172 °C; $\mathbf{R_f}$ (70:30 hexanes/EtOAc) 0.4; **IR** (ATR) ν 2971, 2927, 1774, 1699, 1684, 1586, 1471, 1393, 1359, 1328, 1215, 1039, 755 $cm^{-1}$; $^1$**H NMR** (500 MHz, $CDCl_3$) δ 7.65–7.59 (2H, m, ArH), 7.57 (1H, d, *J* = 2.5 Hz, ArH), 7.48–7.44 (2H, m, ArH), 6.91 (1H, br s, COCH), 6.83 (1H, d, *J* = 9.0 Hz, ArH), 6.66 (1H, dd, *J* = 9.0, 2.5 Hz, ArH), 4.47–4.38 (1H, m, $OCH_xH_y$), 4.31 (1H, q, *J* = 8.8 Hz, $OCH_xH_y$), 4.10 (1H, ddd, *J* = 11.0, 9.5, 8.4 Hz, $NCH_xH_y$), 3.93–3.84 (4H, m, $NCH_xH_y$, $OCH_3$), 2.52 (3H, s, $NCCH_3$), 1.62–1.25 (6H, m, 3 × $CH_2$), 1.24 (3H, s, $CH_3$), 1.16 (3H, s, $CH_3$), 0.98 (3H, s, $CH_3$), 0.80 (3H, s, $CH_3$); $^{13}$**C{$^1$H} NMR** (126 MHz, $CDCl_3$) δ 171.5 (C), 168.6 (C), 155.9 (C), 153.0 (C), 139.6 (C), 137.7 (C), 133.8 (C), 131.4 (CH), 131.0 (C), 129.2 (C), 129.2 (CH), 116.2 (C), 114.3 (CH), 112.2 (CH), 104.0 (CH), 79.6 (CH), 62.2 ($CH_2$), 60.1 (C), 59.7 (C), 55.7 ($CH_3$), 42.9 ($CH_2$), 40.0 ($CH_2$), 39.9 ($CH_2$), 33.1 ($CH_3$), 33.0 ($CH_3$), 20.2 ($CH_3$), 20.2 ($CH_3$), 17.2 ($CH_2$), 14.4 ($CH_3$); **HRMS** (+ESI): *m/z* calcd. for $C_{31}H_{37}ClN_3O_6$ $[M+H]^+$: 582.2365, found: 582.2388.

<u>**5. Summary of the Synthesis**</u>

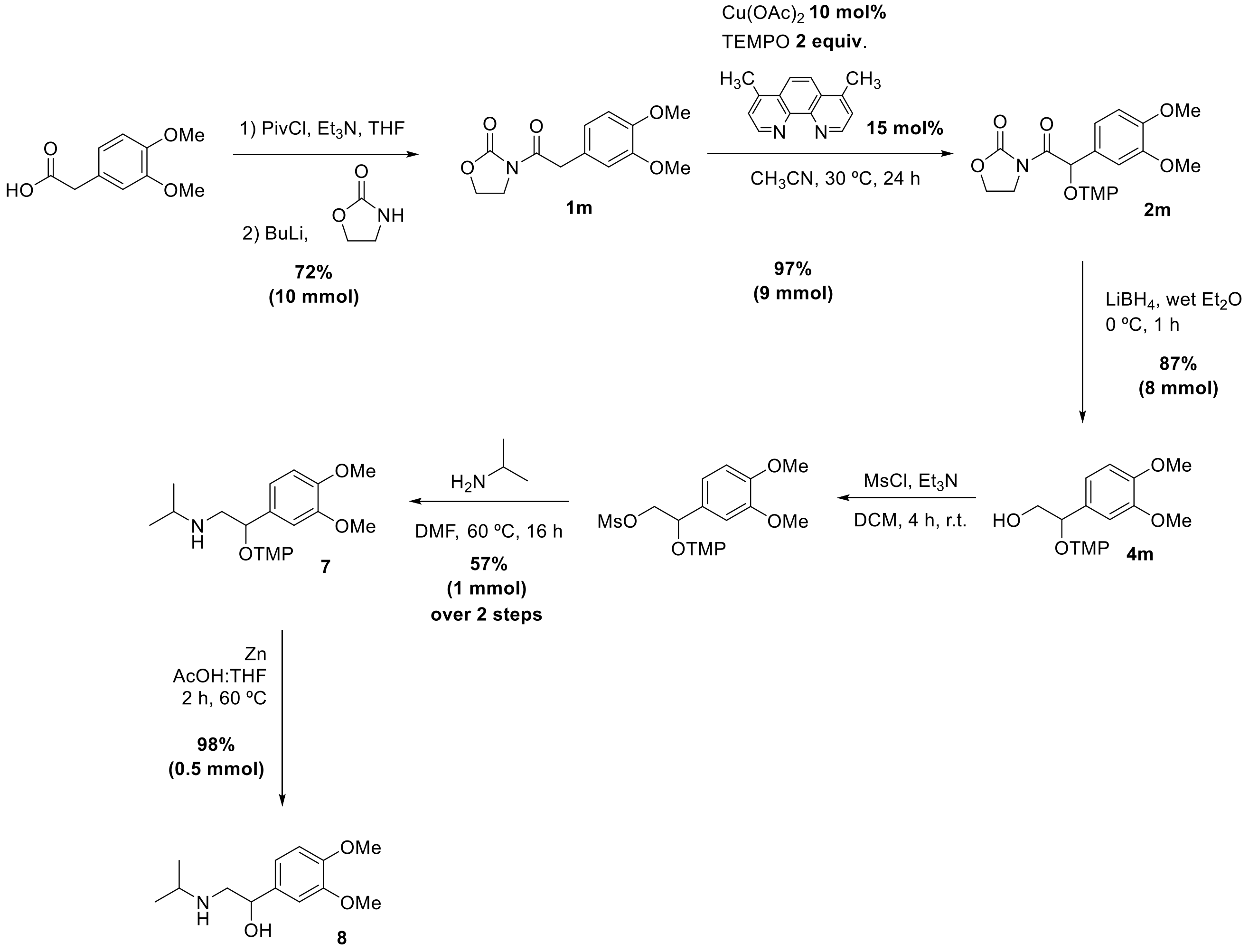


**Scheme S2**. Synthetic route towards **8**

## 6. Synthetic Steps

### *N*-[2-(3,4-Dimethoxyphenyl)-2-(2,2,6,6-tetramethylpiperidin-1-yloxy)acetyl]-1,3-oxazolidin-2-one (2m)

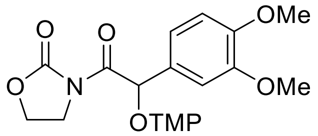


It was prepared following General Procedure 2 from **1m** (2.39 g, 9.0 mmol), $Cu(OAc)_2$ (163 mg, 0.9 mmol, 10 mol%), 4,7-dimethyl-1,10-phenanthroline (281 mg, 1.35 mmol, 15 mol%), and TEMPO (2.81 g, 18 mmol). Purification of the crude product by flash column chromatography (from 70:30 to 50:50 hexanes/EtOAc) afforded **2m** (3.67 g, 8.7 mmol, 97% yield) as a yellow solid.

### 2-(3,4-Dimethoxyphenyl)-2-((2,2,6,6-tetramethylpiperidin-1-yl)oxy)-1-ethanol (4m)

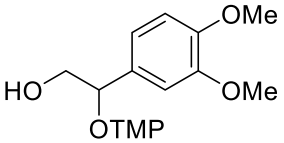


A solution of the α-aminoxylated compound **2m** (3.36 g, 8.0 mmol) in $Et_2O$ (160 mL) was cooled to 0 °C under a nitrogen atmosphere. Water (0.29 mL, 16.0 mmol) was added, followed by a solution of $LiBH_4$ (2 M in THF, 8.0 mL, 16.0 mmol) and the reaction mixture was stirred at 0 °C for 1 h.

The mixture was quenched with 1 M NaOH (50 mL) and extracted with EtOAc (3 × 50 mL). The combined organic extracts were washed with brine (150 mL), dried with anhydrous $MgSO_4$, filtered, and concentrated *in vacuo*. Purification of the crude product by flash column chromatography (from 80:20 to 60:40 hexanes/EtOAc) afforded **4m** (2.34 g, 6.9 mmol, 87% yield) as a colorless oil. **$R_f$** (70:30 hexanes/EtOAc) 0.4; **IR** (ATR) ν 3412, 2930, 2870, 2835, 1606, 1593, 1515, 1463, 1416, 1362, 1254, 1232, 1133, 1027, 805, 732 $cm^{-1}$; **$^1$H NMR** (400 MHz, $CDCl_3$) δ 6.93–6.87 (2H, m, ArH), 6.84 (1H, d, $J$ = 8.5 Hz, ArH), 5.67 (1H, br s, OH), 5.22 (1H, dd, $J$ = 9.5, 2.9 Hz, NOCH), 4.20 (1H, dd, $J$ = 12.2, 9.5 Hz, $CH_xH_yOH$), 3.88 (3H, s, $OCH_3$), 3.86 (3H, s, $OCH_3$), 3.74–3.64 (1H, m, $CH_xH_yOH$), 1.80–1.35 (9H, m, $CH_3$, 3 × $CH_2$), 1.32 (3H, s, $CH_3$), 1.21 (3H, s, $CH_3$), 1.15 (3H, s, $CH_3$); **$^{13}$C{$^1$H} NMR** (101 MHz, $CDCl_3$) 148.9 (C), 148.8 (C), 131.7 (C), 119.2 (CH), 111.1 (CH), 110.1 (CH), 83.5 (CH), 69.8 ($CH_2$), 61.7 (C), 60.4 (C), 56.0 ($CH_3$), 55.9 ($CH_3$), 40.5 ($CH_2$), 40.3 ($CH_2$), 34.7 ($CH_3$), 33.0 ($CH_3$), 20.8 ($CH_3$), 20.5 ($CH_3$), 17.2 ($CH_2$); **HRMS** (+ESI): $m/z$ calcd. for $C_{19}H_{32}NO_4$ $[M+H]^+$: 338.2326, found: 338.2327.

**2-(3,4-Dimethoxyphenyl)-2-((2,2,6,6-tetramethylpiperidin-1-yl)oxy)-1-ethyl methanesulfonate**

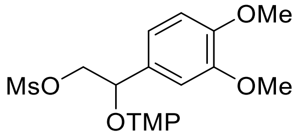


Methanesulfonyl chloride (117 µL, 1.5 mmol) was added dropwise to a solution of **4m** (337 mg, 1.0 mmol) and $Et_3N$ (209 µL, 1.5 mmol) in DCM (13 mL) at 0 °C. The reaction mixture was stirred at 25 °C for 4 h.

The resulting mixture was quenched with water (20 mL), and the aqueous layer was extracted with DCM (3 × 20 mL). The combined organic extracts were dried over anhydrous $MgSO_4$ and concentrated *in vacuo* to afford the crude product as a yellow oil, which was used in the next step without further purification.

***N*-[2-(3,4-Dimethoxyphenyl)-2-((2,2,6,6-tetramethylpiperidin-1-yl)oxy)-1-ethyl]isopropylamine (7)**

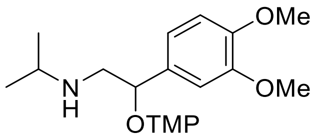


Neat isopropylamine (344 µL, 4.0 mmol) was added to a solution of the above-mentioned crude product (1.0 mmol) in DMF (13 mL) at room temperature. The reaction mixture was then heated in an oil bath to 60 °C and stirred for 16 h. The resultant mixture was then partitioned between $H_2O$ (20 mL) and EtOAc (20 mL).

The aqueous layer was basified with 2 M NaOH until pH 10 and extracted with EtOAc (3 × 20 mL). The combined organic extracts were washed with sat. $NaHCO_3$ (6 × 80 mL), dried with anhydrous $MgSO_4$, and concentrated *in vacuo*. Purification of the crude product by flash column chromatography (from 98:2 to 90:10 DCM/MeOH) afforded **7** (216 mg, 0.57 mmol, 57% yield over two steps) as a yellow oil. $\mathbf{R_f}$ (94:6 DCM/MeOH) 0.3; **IR** (ATR) ν 2999, 2961, 2930, 2870, 2835, 1605, 1593, 1508, 1463, 1360, 1258, 1236, 1133, 1029, 807, 732 $cm^{-1}$; **$^1$H NMR** (400 MHz, $CDCl_3$) δ 6.92–6.85 (2H, m, Ar$\underline{H}$), 6.81 (1H, d, $J$ = 8.0 Hz, Ar$\underline{H}$), 4.74 (1H, dd, $J$ = 8.4, 5.5 Hz, NOC$\underline{H}$), 3.89 (3H, s, OC$\underline{H}_3$), 3.88 (3H, s, OC$\underline{H}_3$), 3.28 (1H, dd, $J$ = 11.5, 5.5 Hz, C$\underline{H}_x$H$_y$N), 2.79 (1H, dd, $J$ = 11.5, 8.4 Hz, CH$_x\underline{H}_y$N), 2.71 (1H, h, $J$ = 6.2 Hz, NC$\underline{H}$(CH$_3$)$_2$), 1.63–1.23 (9H, m, 3 × C$\underline{H}_2$, CC$\underline{H}_3$), 1.16 (3H, s, CC$\underline{H}_3$), 0.99 (6H, d, $J$ = 6.2 Hz, CHC$\underline{H}_3$ ,CC$\underline{H}_3$), 0.96 (3H, d, $J$ = 6.2 Hz, CHC$\underline{H}_3$), 0.63 (3H, s, CC$\underline{H}_3$); **$^{13}$C{$^1$H} NMR** (101 MHz, $CDCl_3$) δ 148.7 (C), 148.4 (C), 134.9 (C), 120.4 (CH), 110.9 (CH), 110.6 (CH), 85.5 (CH), 60.1 (C), 59.6 (C), 55.9 ($CH_3$), 55.8 ($CH_3$), 51.9 ($CH_2$), 48.4 (CH), 40.5 ($CH_2$), 40.5 ($CH_2$), 34.2 ($CH_3$), 34.2 ($CH_3$), 23.2 ($CH_3$), 22.7 ($CH_3$), 20.4 ($CH_3$), 20.4 ($CH_3$), 17.2 ($CH_2$); **HRMS** (+ESI): *m/z* calcd. for $C_{22}H_{39}N_2O_3$ [M+H]$^+$: 379.2955, found: 379.2958.

**1-(3,4-Dimethoxyphenyl)-2-(isopropylamino)-1-ethanol (8)**

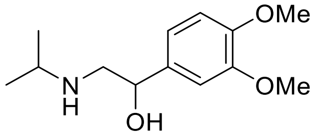


Zinc dust (1.31 g, 20 mmol) was added to a solution of **7** (189 mg, 0.50 mmol) in AcOH (3 mL) and THF (1 mL). The reaction mixture was stirred for 2 h in an oil bath at 60 °C and then it was diluted with EtOAc.

The diluted solution was filtered through cotton and the organic layer was extracted with 1 M HCl (3 × 5 mL). Subsequently, the aqueous layer was neutralized with 2 M NaOH until pH 10 and extracted with EtOAc (3 × 20 mL). The combined organic extracts were dried with $K_2CO_3$ and concentrated *in vacuo* affording **8** (117 mg, 0.49 mmol, 98% yield) as a white solid. **Mp** 72–74 °C; **$R_f$** (80:20 DCM/MeOH) 0.2; **IR** (ATR) ν 3288, 3125, 2961, 2937, 2892, 2835, 1590, 1517, 1455, 1414, 1355, 1264, 1226, 1135, 1092, 1075, 1023, 883, 803, 742 $cm^{-1}$; **$^1$H NMR** (400 MHz, $CDCl_3$) δ 6.94 (1H, d, *J* = 1.9 Hz, ArH), 6.87 (1H, dd, *J* = 8.2, 1.9 Hz, ArH), 6.83 (1H, d, *J* = 8.2 Hz, ArH), 4.61 (1H, dd, *J* = 9.0, 3.7 Hz, CHOH), 3.88 (3H, s, $OCH_3$), 3.86 (3H, s, $OCH_3$), 2.89 (1H, dd, *J* = 12.0, 3.7 Hz, $CH_xH_yN$), 2.81 (1H, h, *J* = 6.2 Hz, $NCH(CH_3)_2$), 2.64 (1H, dd, *J* = 12.0, 9.0 Hz, $CH_xH_yN$), 1.06 (6H, d, *J* = 6.2 Hz, $CH(CH_3)_2$); **$^{13}$C{$^1$H} NMR** (101 MHz, $CDCl_3$) δ 149.1 (C), 148.4 (C), 135.7 (C), 118.1 (CH), 111.1 (CH), 109.1 (CH), 72.0 (CH), 56.0 ($CH_3$), 56.0 ($CH_3$), 54.9 ($CH_2$), 48.8 (CH), 23.3 ($CH_3$), 23.2 ($CH_3$); **HRMS** (+ESI): *m/z* calcd. for $C_{13}H_{22}NO_3$ $[M+H]^+$: 240.1594, found: 240.1592.

## 7. Removal of the Achiral Auxiliary from 2l

### 2-(Benzo[*d*][1,3]dioxol-5-yl)-*N*-(2-hydroxyethyl)-2-((2,2,6,6-tetramethylpiperidin-1-yl)oxy)acetamide (3l)

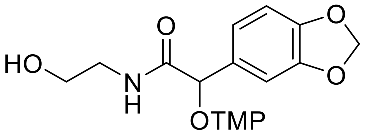


A solution of oxazolidinone **2l** (40.5 mg, 0.10 mmol) and LiOH·$H_2O$ (8.4 mg, 0.20 mmol) in 2 mL of THF/water (1:1) was stirred for 2 h at room temperature.
After completion of the reaction, 2 M HCl was added until pH was adjusted to 1. The layers were separated, and the aqueous layer was extracted with DCM (3 × 5 mL) and the combined organic extracts were dried with anhydrous $MgSO_4$ and concentrated *in vacuo*. Purification of the crude product by flash column chromatography (from 60:40 to 40:60 hexanes/EtOAc) afforded **3l** (33 mg, 87 µmol, 87% yield) as a white solid. **Mp** 50–55 °C; **$R_f$** (50:50 hexanes/EtOAc) 0.2; **IR** (ATR) ν 3412, 3320, 2965, 2928, 2872, 1653, 1487, 1441, 1362, 1241, 1034, 922, 807 $cm^{-1}$; **$^1$H NMR** (400 MHz, $CDCl_3$) δ 7.07 (1H, br s, CON$\underline{H}$), 6.86–6.82 (2H, m, Ar$\underline{H}$), 6.77–6.73 (1H, m, Ar$\underline{H}$), 5.96–5.92 (2H, m, OC$\underline{H}_2$O), 5.00 (1H, s, NOC$\underline{H}$), 3.74 (2H, t, *J* = 5.0 Hz, C$\underline{H}_2$OH), 3.57–3.47 (1H, m, C$\underline{H}_x$$H_y$NH), 3.46–3.36 (1H, m, C$H_x$$\underline{H}_y$NH), 2.51 (1H, br s, O$\underline{H}$), 1.72–1.23 (6H, m, 3 × C$\underline{H}_2$), 1.23–0.96 (9H, m, 3 × C$\underline{H}_3$), 0.62 (3H, s, C$\underline{H}_3$); **$^{13}$C{$^1$H} NMR** (101 MHz, $CDCl_3$) δ 172.8 (C), 147.4 (C), 147.1 (C), 133.1 (C), 121.0 (CH), 107.9 (CH), 107.1 (CH), 100.8 ($CH_2$), 89.6 (CH), 62.0 ($CH_2$), 60.1 (C), 59.4 (C), 41.8 ($CH_2$), 40.1 ($CH_2$), 33.8 ($CH_3$), 32.7 ($CH_3$), 20.1 ($CH_3$), 16.7 ($CH_2$); **HRMS** (+ESI): *m/z* calcd. for $C_{20}H_{31}N_2O_5$ $[M+H]^+$: 379.2227, found: 379.2219.

### 2-(Benzo[*d*][1,3]dioxol-5-yl)-2-(2,2,6,6-tetramethylpiperidin-1-yloxy)-1-ethanol (4l)

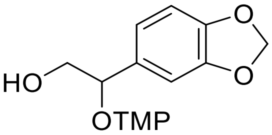


A solution of the α-aminoxylated compound **2l** (121 mg, 0.30 mmol) in $Et_2O$ (6 mL) was cooled to 0 °C under a nitrogen atmosphere. Water (11 µL, 0.60 mmol) was added, followed by a solution of $LiBH_4$ (2 M in THF, 0.3 mL, 0.60 mmol) and the reaction mixture was stirred at 0 °C for 1 h. The mixture was quenched with 1 M NaOH (10 mL) and extracted with EtOAc (3 × 10 mL). The combined organic extracts were washed with brine (30 mL), dried with anhydrous $MgSO_4$, filtered, and concentrated *in vacuo*. Purification of the crude product by flash column chromatography (from 80:20 to 70:30 hexanes/EtOAc) afforded **4l** (94 mg, 0.29 mmol, 97% yield) as a white solid. **Mp** 47–51 °C; **$R_f$** (80:20 hexanes/EtOAc) 0.4; **IR** (ATR) ν 3259, 2917, 1490, 1438, 1362, 1238, 1081, 1028, 933, 804 $cm^{-1}$; **$^1$H NMR** (500 MHz, $CDCl_3$) δ 6.87 (1H, d, *J* = 1.6 Hz, Ar$\underline{H}$), 6.83–6.74 (2H, m, Ar$\underline{H}$), 5.98–5.92 (2H, m, OC$\underline{H}_2$O), 5.68 (1H, br s, O$\underline{H}$), 5.19 (1H, dd, *J* = 9.4, 2.8 Hz, C$\underline{H}$ON), 4.19 (1H, dd, *J* = 12.2, 9.4 Hz, C$\underline{H}_x$$H_y$OH), 3.67 (1H, dd, *J* = 12.2, 2.8 Hz, C$H_x$$\underline{H}_y$OH), 1.69–1.35 (9H, m, 3 × C$\underline{H}_2$, C$\underline{H}_3$), 1.31 (3H, s, C$\underline{H}_3$), 1.20 (3H, s, C$\underline{H}_3$), 1.15 (3H, s, C$\underline{H}_3$); **$^{13}$C{$^1$H} NMR** (126 MHz, $CDCl_3$) δ 147.7 (C), 147.3 (C), 132.9 (C), 120.5 (CH), 108.3 (CH), 107.4 (CH), 101.2 ($CH_2$), 83.6 (CH), 69.7 ($CH_2$), 61.8 (C), 60.5 (C),

40.5 ($CH_2$), 40.3 ($CH_2$), 34.7 ($CH_3$), 32.9 ($CH_3$), 20.8 ($CH_3$), 20.6 ($CH_3$), 17.3 ($CH_2$); **HRMS** (+ESI): *m/z* calcd. for $C_{18}H_{28}NO_4$ $[M+H]^+$: 322.2013, found: 322.2021.

### 2-(Benzo[*d*][1,3]dioxol-5-yl)-2-((2,2,6,6-tetramethylpiperidin-1-yl)oxy)acetaldehyde (5l)

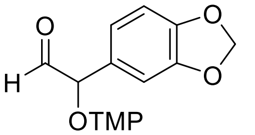


A solution of α-aminoxylated adduct **2l** (121 mg, 0.30 mmol) in THF (1 mL) was transferred via cannula to a suspension of $LiAlH_4$ (23 mg, 0.60 mmol) in THF (0.40 mL) at –78 °C. The resulting mixture was stirred at –78 °C for 1.5 h, followed by stirring at –50 °C for an additional 1.5 h. Water (23 µL) was carefully added to the mixture at –78 °C, and the solution was stirred vigorously for 5 min. Next, 3 M NaOH (23 µL) was added, and the mixture was allowed to reach room temperature. Additional water (72 µL) was added, and the mixture was stirred for 15 min before filtering through a Celite® pad using DCM.
The organic solution was concentrated under reduced pressure, and the resulting oil was purified by flash column chromatography (from 90:10 to 80:20 hexanes/EtOAc), yielding **5l** (61 mg, 0.19 mmol, 64% yield) as an orange oil. **$R_f$** (80:20 hexanes/EtOAc) 0.6; **IR** (ATR) ν 2970, 2930, 2870, 2848, 2787, 2686, 1728, 1504, 1488, 1441, 1375, 1361, 1244, 1132, 1037, 917, 809, 732 $cm^{-1}$; **$^1$H NMR** (400 MHz, $CDCl_3$) δ 9.62 (1H, d, *J* = 3.9 Hz, CO<u>H</u>), 6.87–6.85 (1H, m, Ar<u>H</u>), 6.84–6.82 (2H, m, Ar<u>H</u>), 6.00–5.96 (2H, m, OC<u>H</u>$_2$O), 4.98 (1H, d, *J* = 3.9 Hz, NOC<u>H</u>), 1.69–1.27 (6H, m, 3 × C<u>H</u>$_2$), 1.25 (3H, s, C<u>H</u>$_3$), 1.18 (3H, s, C<u>H</u>$_3$), 1.14 (3H, s, C<u>H</u>$_3$), 0.91 (3H, s, C<u>H</u>$_3$); **$^{13}$C{$^1$H} NMR** (101 MHz, $CDCl_3$) δ 198.8 (CH), 148.0 (C), 147.5 (C), 129.1 (C), 120.7 (CH), 108.5 (CH), 107.3 (CH), 101.1 ($CH_2$), 92.6 (CH), 60.1 (C), 59.9 (C), 39.9 ($CH_2$), 33.9 ($CH_3$), 33.4 ($CH_3$), 20.4 ($CH_3$), 20.1 ($CH_3$), 17.0 ($CH_2$); **HRMS** (+ESI): *m/z* calcd. for $C_{18}H_{26}NO_4$ $[M+H]^+$: 320.1856, found: 320.1850.

### Methyl 2-(benzo[*d*][1,3]dioxol-5-yl)-2-((2,2,6,6-tetramethylpiperidin-1-yl)oxy)acetate (6l)

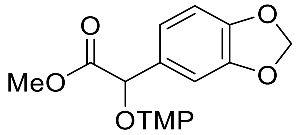


On the benchtop, $Yb(OTf)_3$ (9.3 mg, 15 µmol, 5 mol%) was weighted in a 5 mL microwave vial, followed by the addition of anhydrous methanol (1.5 mL). The vial was sealed with a pressure-relief cap and heated at 60 °C in an oil bath for 30 min. Then, a solution of **2l** (121 mg, 0.30 mmol) in anhydrous methanol (1.5 mL) was transferred via cannula. The mixture was then stirred at 60 °C for 2 h and the solvent was carefully evaporated under reduced pressure. The crude product was purified by flash column chromatography (from 95:5 to 80:20 hexanes/EtOAc) to afford **6l** (101 mg, 0.29 mmol, 96% yield) as a white solid. **Mp** 100–102 °C; **$R_f$** (80:20 hexanes/EtOAc) 0.7; **IR** (ATR) ν 2969, 2924, 2864, 2775, 1744, 1502, 1437, 1364, 1334, 1238, 1196, 1165, 1072, 1038, 930, 814, 751 $cm^{-1}$; **$^1$H NMR** (400 MHz, $CDCl_3$) δ 6.96 (1H, d, *J* = 1.7 Hz, Ar<u>H</u>), 6.87 (1H, dd, *J* = 8.0, 1.7 Hz, Ar<u>H</u>), 6.76 (1H, d, *J* = 8.0 Hz, Ar<u>H</u>), 5.97–5.94 (2H, m, OC<u>H</u>$_2$O), 5.11 (1H, s, NOC<u>H</u>), 3.66 (3H, s, OC<u>H</u>$_3$), 1.65–1.25 (6H, m, 3 × C<u>H</u>$_2$), 1.21

(3H, s, C$\underline{H}_3$), 1.11 (3H, s, C$\underline{H}_3$), 1.07 (3H, s, C$\underline{H}_3$), 0.78 (3H, s, C$\underline{H}_3$); **$^{13}$C{$^1$H} NMR** (101 MHz, $CDCl_3$) δ 172.5 (C), 147.7 (C), 147.4 (C), 132.1 (C), 120.7 (CH), 108.1 (CH), 107.4 (CH), 101.1 ($CH_2$), 88.2 (CH), 59.9 (C), 59.9 (C), 51.8 ($CH_3$), 40.2 ($CH_2$), 40.1 ( $CH_2$), 33.7 ($CH_3$), 32.9 ($CH_3$), 20.2 ($CH_3$), 17.1 ($CH_2$); **HRMS** (+ESI): *m/z* calcd. for $C_{19}H_{28}NO_5$ $[M+H]^+$: 350.1962, found: 350.1954.

## 8. Miscellaneous

### *N*-Phenylacetylpyrrolidine (9a) [4]

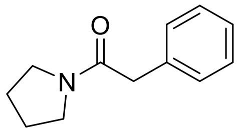

A flame-dried round-bottom flask was charged with phenylacetic acid (1.36 g, 10 mmol, 1.0 equiv.) and dissolved in DCM (50 mL). Triethylamine (1.7 mL, 12 mmol, 1.2 equiv.), 1-ethyl-3-(3-dimethylaminopropyl)carbodiimide hydrochloride (2.3 g, 12 mmol, 1.2 equiv.), and DMAP (0.12 g, 1 mmol, 0.1 equiv.) were sequentially added to the flask. After stirring for 5 min, neat pyrrolidine (1.0 mL, 12 mmol,1.2 equiv.) was added, and the reaction mixture was stirred at room temperature overnight.

The reaction was diluted with DCM (25 mL) and quenched with water (25 mL). The mixture was transferred to a separatory funnel, and the organic layer was washed successively with sat. $NaHCO_3$ solution (70 mL), sat. $NH_4Cl$ solution (70 mL), water (70 mL), and brine (70 mL). The organic layer was dried over anhydrous $MgSO_4$ and concentrated *in vacuo*. The crude product was purified by flash column chromatography (from 40:60 to 20:80 hexanes/EtOAc) to afford **9a** (1.26 g, 6.7 mmol, 67% yield) as a white solid. **Mp** 39–40 °C; **$R_f$** (40:60 hexanes/EtOAc) 0.3; **IR** (ATR) ν 3079, 3053, 3027, 2967, 2876, 1620, 1493, 1420, 1338, 1152, 915, 719, 695 $cm^{-1}$; **$^1$H NMR** (400 MHz, $CDCl_3$) δ 7.34–7.19 (5H, m, Ar<u>H</u>), 3.66 (2H, s, COC<u>H</u>$_2$), 3.49 (2H, t, *J* = 6.8 Hz, NC<u>H</u>$_2$), 3.42 (2H, t, *J* = 6.7 Hz, NC<u>H</u>$_2$), 1.96–1.87 (2H, m, $NCH_2C$<u>H</u>$_2$), 1.87–1.78 (2H, m, $NCH_2C$<u>H</u>$_2$); **$^{13}$C{$^1$H} NMR** (101 MHz, $CDCl_3$) δ 169.6 (C), 135.1 (C), 129.1 (CH), 128.7 (CH), 126.8 (CH), 47.0 ($CH_2$), 46.0 ($CH_2$), 42.4 ($CH_2$), 26.3 ($CH_2$), 24.5 ($CH_2$).

### *N*-Phenylacetyl-1,3-oxazolidine-2-thione (10a) [5]

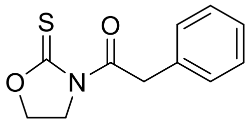

A flame-dried round-bottom flask was charged with 1,3-oxazolidin-2-thione (413 mg, 4.0 mmol, 1.0 equiv.), 1-ethyl-3-(3-dimethylaminopropyl)carbodiimide hydrochloride (1.0 g, 5.2 mmol, 1.3 equiv.), and DMAP (0.10 g, 0.8 mmol, 0.2 equiv.) and dissolved in DCM (5 mL) at 0 °C. A solution of phenylacetic acid (654 mg, 4.8 mmol, 1.2 equiv.) in DCM (7 mL) was transferred to the flask via cannula at 0 °C. After stirring for 5 min, the reaction mixture was stirred overnight at room temperature.

The reaction was quenched with sat. $NH_4Cl$ solution (10 mL). The mixture was transferred to a separatory funnel, and the organic layer was washed successively with water (20 mL) and sat. $NaHCO_3$ solution (2 × 20 mL). The organic layer was dried over anhydrous $MgSO_4$ and concentrated *in vacuo*. The crude product was purified by flash column chromatography (80:20 hexanes/EtOAc) to afford **10a** (716 mg, 3.2 mmol, 81% yield) as a white solid. **Mp** 68–70 °C; **$R_f$** (70:30 hexanes/EtOAc) 0.3; **IR** (ATR) ν 3025, 2968, 2917, 2904, 2851, 1693, 1481, 1470, 1391, 1354, 1321, 1219, 1195, 1159, 941, 691 $cm^{-1}$; **$^1$H NMR** (400 MHz, $CDCl_3$) δ 7.39–7.23 (5H, m, Ar<u>H</u>), 4.72 (2H, s, COC<u>H</u>$_2$), 4.58–4.48 (2H, m, OC<u>H</u>$_2$), 4.27–4.18 (2H, m, NC<u>H</u>$_2$);

**$^{13}C\{^1H\}$ NMR** (101 MHz, $CDCl_3$) δ 185.7 (C), 172.5 (C), 133.6 (C), 129.9 (CH), 128.7 (CH), 127.3 (CH), 66.5 ($CH_2$), 47.4 ($CH_2$), 43.1 ($CH_2$).

**_N_-Phenylacetyl-1,3-thiazolidine-2-thione (11a)** [5]

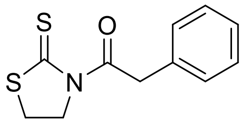

A flame-dried round-bottom flask was charged with 1,3-thiazolidine-2-thione (477 mg, 4.0 mmol, 1.0 equiv.), 1-ethyl-3-(3-dimethylaminopropyl)carbodiimide hydrochloride (1.0 g, 5.2 mmol, 1.3 equiv.), and DMAP (0.10 g, 0.8 mmol, 0.2 equiv.) and dissolved in DCM (5 mL) at 0 °C. A solution of phenylacetic acid (654 mg, 4.8 mmol, 1.2 equiv.) in DCM (7 mL) was transferred to the flask via cannula at 0 °C. After stirring for 5 min, the reaction mixture was stirred overnight at room temperature.

The reaction was quenched with sat. $NH_4Cl$ solution (10 mL). The mixture was transferred to a separatory funnel, and the organic layer was washed successively with water (20 mL) and sat. $NaHCO_3$ solution (2 × 20 mL). The organic layer was dried over anhydrous $MgSO_4$ and concentrated *in vacuo*. The crude product was purified by flash column chromatography (from 90:10 to 80:20 hexanes/EtOAc) to afford **11a** (823 mg, 3.5 mmol, 87% yield) as a yellow solid. **Mp** 84–85 °C; **$R_f$** (70:30 hexanes/EtOAc) 0.5; **IR** (ATR) ν 3056, 3022, 2933, 2882, 1699, 1496, 1451, 1384, 1369, 1344, 1282, 1268, 1221, 1158, 1146, 1036, 996, 765, 709, 694 $cm^{-1}$; **$^1H$ NMR** (400 MHz, $CDCl_3$) δ 7.38–7.20 (5H, m, Ar$\underline{H}$), 4.65 (2H, s, CO$C\underline{H}_2$), 4.58 (2H, t, *J* = 7.5 Hz, N$C\underline{H}_2$), 3.27 (2H, t, *J* = 7.5 Hz, S$C\underline{H}_2$); **$^{13}C\{^1H\}$ NMR** (101 MHz, $CDCl_3$) δ 201.9 (C), 173.0 (C), 133.9 (C), 129.9 (CH), 128.6 (CH), 127.3 (CH), 56.4 ($CH_2$), 44.5 ($CH_2$), 28.4 ($CH_2$).

**_N_-Propanoyl-1,3-oxazolidin-2-one (12a)** [6]

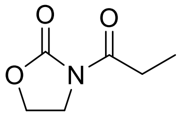

A solution of *n*-butyllithium (2.5 M in hexanes, 2.2 mL, 5.5 mmol) was slowly added using a syringe to a stirred solution of 1,3-oxazolidin-2-one (435 mg, 5.0 mmol) in THF (13 mL) at −78 °C under nitrogen atmosphere. The mixture was stirred for 15 min before adding propanoyl chloride (0.57 mL, 6.5 mmol) dropwise at −78 °C. Stirring was continued at −78 °C for 30 min, and the reaction was gradually warmed to room temperature and stirred for 2 h.

A saturated $NH_4Cl$ aqueous solution was then added (15 mL), and the reaction mixture was extracted with EtOAc (3 × 15 mL). The combined organic layers were washed with a sat. $NaHCO_3$ solution (70 mL), brine (70 mL), dried over anhydrous $MgSO_4$, and concentrated under reduced pressure. The crude product was purified by flash column chromatography (60:40 hexanes/EtOAc) to afford **12a** (616 mg, 4.3 mmol, 86% yield) as a white solid. **Mp** 82–83 °C; **$R_f$** (60:40 hexanes/EtOAc) 0.3; **IR** (ATR) ν 2988, 2920, 2885, 1763, 1694, 1385, 1360, 1260, 1206, 1124, 1081, 1044, 1025, 939, 807, 758, 691 $cm^{-1}$; **$^1H$ NMR** (400 MHz, $CDCl_3$) δ 4.43–4.37 (2H, m, O$C\underline{H}_2$), 4.04–3.98 (2H, m, N$C\underline{H}_2$), 2.92 (2H, q, *J* = 7.4 Hz, $C\underline{H}_2CH_3$), 1.16 (3H, t, *J* = 7.4 Hz, $CH_2C\underline{H}_3$); **$^{13}C\{^1H\}$ NMR** (101 MHz, $CDCl_3$) δ 174.4 (C), 153.7 (C), 62.2 ($CH_2$), 42.6 ($CH_2$), 28.9 ($CH_2$), 8.4 ($CH_3$).

**N-[(E)-2-Butenoyl]-1,3-oxazolidin-2-one (13a)**

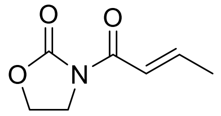

It was prepared following General Procedure 1 from (*E*)-2-butenoic acid (560 mg, 6.5 mmol), $Et_3N$ (0.9 mL, 6.5 mmol), PivCl (0.8 mL, 6.5 mmol), 1,3-oxazolidin-2-one (435 mg, 5.0 mmol) and *n*-BuLi (2.5 M in hexanes, 2.2 mL, 5.5 mmol). Purification of the crude product by flash column chromatography (from 80:20 to 60:40 hexanes/EtOAc) afforded **13a** (445 mg, 2.9 mmol, 58% yield) as a white solid. **Mp** 40–42 °C; $\mathbf{R_f}$ (70:30 hexanes/EtOAc) 0.3; **IR** (ATR) ν 3092, 3021, 2988, 2920, 2853, 1772, 1681, 1636, 1388, 1338, 1286, 1210, 1129, 1092, 1042, 971, 747, 706 cm$^{-1}$; **$^1$H NMR** (400 MHz, $CDCl_3$) δ 7.29–7.22 (1H, m, COC<u>H</u>=CH), 7.17 (1H, dq, *J* = 15.2, 6.5 Hz, COCH=C<u>H</u>), 4.47–4.38 (2H, m, OC<u>H</u>$_2$), 4.11–4.02 (2H, m, NC<u>H</u>$_2$), 1.96 (1H, dd, *J* = 6.5, 1.2 Hz, C<u>H</u>$_3$); **$^{13}$C{$^1$H} NMR** (101 MHz, $CDCl_3$) δ 165.3 (C), 153.6 (C), 146.9 (CH), 121.5 (CH), 62.1 ($CH_2$), 42.8 ($CH_2$), 18.6 ($CH_3$); **HRMS** (+ESI): *m/z* calcd. for $C_7H_9NNaO_3$ $[M+Na]^+$: 178.0475, found: 178.0474.

**N-[2-Phenyl-2-(2,2,6,6-tetramethylpiperidin-1-yloxy)acetyl]-1,3-oxazolidin-2-thione (15a)**

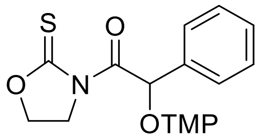

It was prepared following General Procedure 2 from **10a** (66.4 mg, 0.30 mmol), $Cu(OAc)_2$ (5.45 mg, 30 μmol, 10 mol%), 4,7-dimethyl-1,10-phenanthroline (9.37 mg, 45 μmol, 15 mol%), and TEMPO (93.8 mg, 0.60 mmol). Purification of the crude product by flash column chromatography (from 90:10 to 70:30 hexanes/EtOAc) afforded **15a** (81 mg, 0.22 mmol, 72% yield) as a white solid. **Mp** 105–107 °C; $\mathbf{R_f}$ (80:20 hexanes/EtOAc) 0.4; **IR** (ATR) ν 3008, 2961, 2930, 2868, 1709, 1470, 1456, 1374, 1325, 1303, 1215, 1195, 1180, 1152, 1047, 1018, 945, 755, 699 cm$^{-1}$; **$^1$H NMR** (400 MHz, $CDCl_3$) δ 7.77 (1H, s, COC<u>H</u>), 7.70–7.60 (2H, m, Ar<u>H</u>), 7.38–7.26 (3H, m, Ar<u>H</u>), 4.51 (1H, td, *J* = 9.2, 5.9 Hz, OC<u>H</u>$_x$H$_y$), 4.42–4.31 (1H, m, OCH$_x$<u>H</u>$_y$), 4.25 (1H, dt, *J* = 11.3, 9.2 Hz, NC<u>H</u>$_x$H$_y$), 4.02 (1H, ddd, *J* = 11.3, 9.2, 5.9 Hz, NCH$_x$<u>H</u>$_y$), 1.67–1.25 (6H, m, 3 × C<u>H</u>$_2$), 1.21 (3H, s, C<u>H</u>$_3$), 1.14 (3H, s, C<u>H</u>$_3$), 1.03 (3H, s, C<u>H</u>$_3$), 0.72 (3H, s, C<u>H</u>$_3$); **$^{13}$C{$^1$H} NMR** (101 MHz, $CDCl_3$) δ 185.0 (C), 173.7 (C), 137.8 (C), 128.2 (CH), 128.2 (CH), 128.1 (CH), 83.8 (CH), 66.3 ($CH_2$), 59.9 (C), 59.5 (C), 47.3 ($CH_2$), 40.0 ($CH_2$), 39.9 ($CH_2$), 33.7 ($CH_3$), 33.2 ($CH_3$), 20.1 ($CH_3$), 20.0 ($CH_3$), 17.1 ($CH_2$); **HRMS** (+ESI): *m/z* calcd. for $C_{20}H_{29}N_2O_3S$ $[M+H]^+$: 377.1893, found: 377.1883.

**N-[2-Phenyl-2-(2,2,6,6-tetramethylpiperidin-1-yloxy)acetyl]-1,3-thiazolidine-2-thione (16a)**

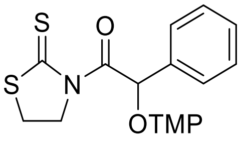

It was prepared following General Procedure 2 from **11a** (71.2 mg, 0.30 mmol), $Cu(OAc)_2$ (5.45 mg, 30 μmol, 10 mol%), 4,7-dimethyl-1,10-phenanthroline (9.37 mg, 45 μmol, 15 mol%), and

TEMPO (93.8 mg, 0.60 mmol). Purification of the crude product by flash column chromatography (from 92:8 to 90:10 hexanes/EtOAc) afforded **16a** (57 mg, 0.14 mmol, 48% yield) as a yellow solid. **Mp** 111–115 °C; **R$_f$** (80:20 hexanes/EtOAc) 0.6; **IR** (ATR) ν 3010, 2967, 2930, 2866, 1701, 1452, 1364, 1312, 1280, 1265, 1221, 1150, 1046, 1019, 999, 740, 693 cm$^{-1}$; **$^1$H NMR** (400 MHz, $CDCl_3$) δ 7.60–7.54 (3H, m, ArH, COCH), 7.35–7.27 (3H, m, ArH), 4.51 (1H, dt, *J* = 12.1, 9.0 Hz, NC$\underline{H}_x$$H_y$), 4.41–4.30 (1H, m, NC$H_x$$\underline{H}_y$), 3.14–3.08 (2H, m, SC$\underline{H}_2$), 1.69–1.25 (6H, m, 3 × C$\underline{H}_2$), 1.23 (3H, s, C$\underline{H}_3$), 1.17 (3H, s, C$\underline{H}_3$), 1.02 (3H, s, C$\underline{H}_3$), 0.67 (3H, s, C$\underline{H}_3$); **$^{13}$C{$^1$H} NMR** (101 MHz, $CDCl_3$) δ 201.5 (C), 174.2 (C), 138.2 (C), 128.4 (CH), 128.3 (CH), 127.9 (CH), 85.8 (CH), 60.1 (C), 59.7 (C), 56.7 ($CH_2$), 40.1 ($CH_2$), 40.1 ($CH_2$), 33.8 ($CH_3$), 33.6 ($CH_3$), 28.6 ($CH_2$), 20.3 ($CH_3$), 20.1 ($CH_3$), 17.2 ($CH_2$); **HRMS** (+ESI): *m/z* calcd. for $C_{20}H_{29}N_2O_2S_2$ $[M+H]^+$: 393.1665, found: 393.1660.

## 9. Preparation of *N*-(β,γ-Unsaturated)acyl-1,3-oxazolidin-2-ones

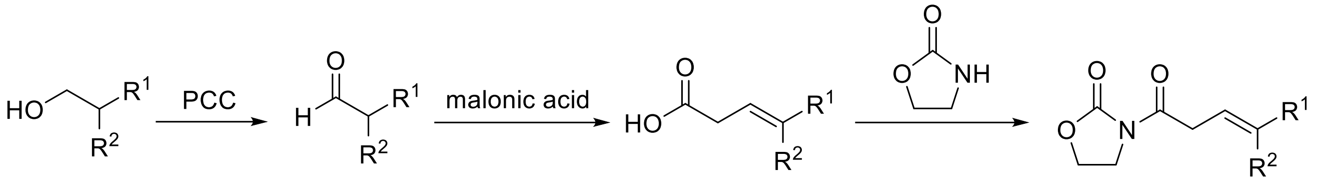


**a-o** **14a-14o**

### 9.1. Synthesis of Aldehydes

**General Procedure 3**

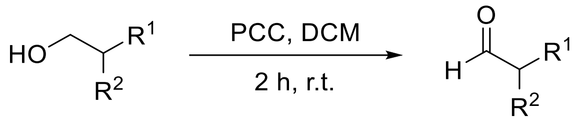


The alcohol (1.0 equiv.) was gradually added to a solution of pyridinium chlorochromate (PCC, 1.5 equiv.) in DCM (0.36 M). The reaction mixture was stirred at r.t. for 2 h, and the resulting suspension was filtered through a silica gel pad, eluting with $Et_2O$. The solvent was then evaporated under reduced pressure, and the crude residue was used directly in the following step without further purification.

**3,7-Dimethyl-6-octenal**

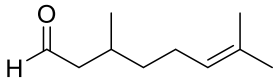


It was prepared following General Procedure 3 from citronellol (1.25 mL, 8.0 mmol) and PCC (2.59 g, 12.0 mmol).

**9-Decynal**

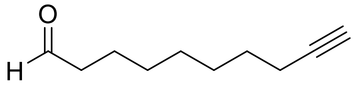


It was prepared following General Procedure 3 from 9-decyn-1-ol (1.42 mL, 8.0 mmol) and PCC (2.59 g, 12.0 mmol).

**6-Chlorohexanal**

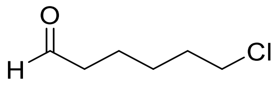


It was prepared following General Procedure 3 from 6-chloro-1-hexanol (1.07 ml, 8.0 mmol) and PCC (2.59 g, 12.0 mmol).

## 9.2. Synthesis of β,γ-Unsaturated Carboxylic Acids [7]

**General Procedure 4**

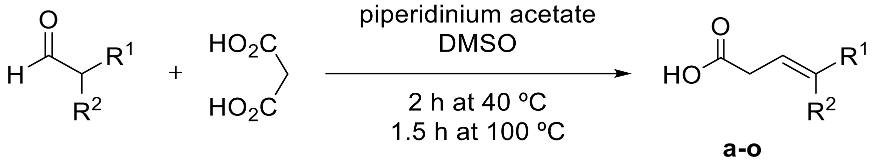


Aldehyde (1.0 equiv.), malonic acid (2.0 equiv.), and piperidinium acetate (0.02 equiv.) were dissolved in DMSO (0.5 M). The reaction mixture was heated in an oil bath at 40 °C and stirred for 2 h. It was then further heated at 100 °C with continued stirring to facilitate decarboxylation. After 1.5 h, $CO_2$ evolution stopped. The mixture was allowed to cool to r.t. and poured into 50 mL of ice-cooled water. The product was extracted with diethyl ether (3 × 50 mL), and the combined organic layers were washed with water (3 × 100 mL) and brine. The organic phase was dried over anhydrous $MgSO_4$, filtered, and concentrated under reduced pressure.

### (*E*)-3-Hexenoic acid (c)

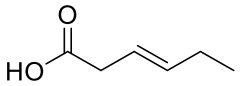


It was prepared following General Procedure 4 from butanal (0.85 mL, 9.5 mmol), malonic acid (1.98 g, 19 mmol) and piperidinium acetate (28 mg, 0.19 mmol). The resulting crude was used directly in the next step.

### (*E*)-5-Methyl-3-hexenoic acid (d)

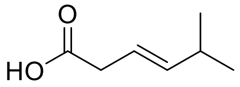


It was prepared following General Procedure 4 from isovaleraldehyde (1.0 mL, 9.5 mmol), malonic acid (1.98 g, 19 mmol) and piperidinium acetate (28 mg, 0.19 mmol). The resulting crude was used directly in the next step.

### (*E*)-5-Phenyl-3-pentenoic acid (e)

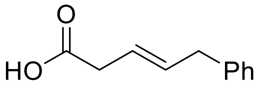


It was prepared following General Procedure 4 from 3-phenylpropanal (0.86 mL, 6.5 mmol), malonic acid (1.35 g, 13 mmol) and piperidinium acetate (19 mg, 0.13 mmol). The resulting crude was used directly in the next step.

**(*E*)-5,9-Dimethyl-3,8-decadienoic acid (f)**

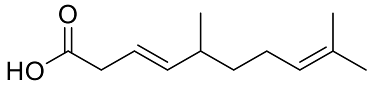

It was prepared following General Procedure 4 from 3,7-dimethyl-6-octenal (0.97 mL, 6.3 mmol), malonic acid (1.31 g, 12.6 mmol) and piperidinium acetate (19 mg, 0.13 mmol). The crude material was used directly in the next step.

**(*E*)-3-Dodecen-11-ynoic acid (g)**

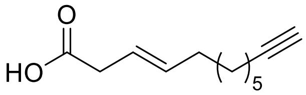

It was prepared following General Procedure 4 from 9-decynal (1.0 mL, 6.7 mmol), malonic acid (1.39 g, 13.4 mmol) and piperidinium acetate (19 mg, 0.13 mmol). The crude material was used directly in the next step.

**(*E*)-8-Chloro-3-octenoic acid (h)**

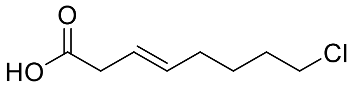

It was prepared following General Procedure 4 from 6-chlorohexanal (0.78 mL, 5.8 mmol), malonic acid (1.21 g, 11.6 mmol) and piperidinium acetate (17 mg, 0.12 mmol). The reaction crude was purified via flash column chromatography (98:2 DCM/MeOH) to afford the pure product **h** (517 mg, 2.9 mmol, 50% yield) as a yellowish oil. **$R_f$** (98:2 DCM/MeOH) 0.3; **IR** (ATR) ν 2935, 1704, 1405, 1288, 1220 $cm^{-1}$; **$^1$H NMR** (500 MHz, $CDCl_3$) δ 11.59–9.84 (1H, br s, O<u>H</u>), 5.63–5.49 (2H, m, C<u>H</u>=C<u>H</u>), 3.53 (2H, t, *J* = 6.7 Hz, ClC<u>H</u>$_2$), 3.09 (2H, d, *J* = 5.8 Hz, COC<u>H</u>$_2$), 2.11–2.05 (2H, m, CH=CHC<u>H</u>$_2$), 1.81–1.73 (2H, m, C<u>H</u>$_2$CH$_2$Cl), 1.53 (2H, p, *J* = 7.6 Hz, CH$_2$C<u>H</u>$_2$CH$_2$); **$^{13}$C{$^1$H} NMR** (126 MHz, $CDCl_3$) δ 178.4 (C), 134.6 (CH), 121.5 (CH), 44.9 ($CH_2$), 37.9 ($CH_2$), 32.0 ($CH_2$), 31.7 ($CH_2$), 26.3 ($CH_2$).

**(*E*)-12-Methoxy-12-oxo-3-dodecenoic acid (j)**

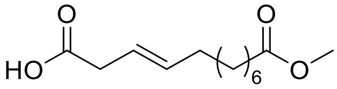

It was prepared following General Procedure 4 from methyl 9-formylnonanoate (1.0 mL, 5 mmol), malonic acid (1.04 g, 10 mmol) and piperidinium acetate (15 mg, 0.10 mmol). The crude material was used directly in the next step.

**Mixture of 4-methyl-3-pentenoic acid (k) and (*E*)-4-methyl-2-pentenoic acid**

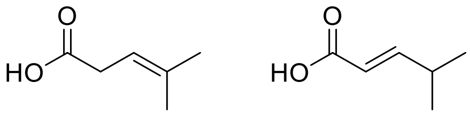

**k**

It was prepared following General Procedure 4 from isobutyraldehyde (9.25 mL, 83.5 mmol), malonic acid (9.55 g, 92 mmol) and piperidinium acetate (17 mg, 0.12 mmol), afforded a mixture of both isomers (42:58) as a yellowish oil.

The mixture was dissolved in a 6 M KOH-solution and heated in an oil bath at 105 °C for 48 h. After cooling to room temperature, the reaction mixture was acidified to pH 1 by the addition of 1M HCl. The mixture was extracted with DCM, and the combined organic phases were dried over anhydrous $MgSO_4$. The dried solution was filtered, and the filtrate was concentrated under reduced pressure to afford a crude mixture of alkene **k** and its α,β-unsaturated isomer (67:33), used directly in the next step without further purification.

**Ethyl penta-3,4-dienoate** [8]

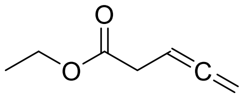

In a two-neck flask equipped with a still head (with the other neck sealed by a rubber septum), propargyl alcohol (1.2 mL, 20 mmol) and triethyl orthoacetate (8.1 mL, 44.0 mmol) were heated in an oil bath at 100 °C. Propionic acid (30 µL, 0.4 mmol) was added, and the temperature was raised to 160 °C. Ethanol generated during the reaction was continuously removed by distillation. Once ethanol evolution stopped, a second portion of propargyl alcohol (1.2 mL, 20 mmol) was added gradually over 15 min at 160 °C, and the reaction was stirred for 2 h. Propionic acid (90 µL, 1.2 mmol) was then added slowly, and the mixture was cooled to room temperature. After cooling, 50 mL of 2 M HCl was added, and the resulting biphasic mixture was separated. The aqueous layer was extracted with $Et_2O$ (3 × 50 mL). The combined organic extracts were washed with sat. $NaHCO_3$ solution (2 × 50 mL), dried over anhydrous $MgSO_4$, and filtered. The filtrate was concentrated under reduced pressure, and the crude product was directly used in the next step.

**Penta-3,4-dienoic acid (n)** [8]

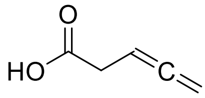

Crude product of ethyl penta-3,4-dienoate (40 mmol, 1.0 equiv.) was dissolved in acetone (1 M, 40 mL), and 20% aqueous HCl (60 mL) was added. The reaction mixture was stirred at room temperature for 27 h, and the biphasic mixture was separated. The aqueous layer was extracted with $Et_2O$ (3 × 100 mL), and the combined organic extracts were dried over anhydrous $MgSO_4$. The solution was then filtered, and the solvent was removed under reduced pressure. The resulting crude product was carried forward directly to the next step without further purification.

**(*E*)-Hexa-3,5-dienoic acid (o)**

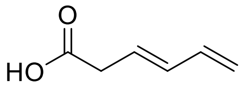

To a cooled solution of diisopropylamine (5.7 mL, 40 mmol) in dry THF (36 mL) at −10 °C, *n*-BuLi (16 mL, 40 mmol, 2.5 M in hexanes) was added, and the resulting mixture was stirred for 30 min. Subsequently, a solution of (2*E*,4*E*)-hexa-2,4-dienoic acid (2.02 g, 18 mmol) in THF (10 mL) was added dropwise to the yellow reaction mixture. Upon addition, the solution turned orange, and a precipitate formed. The reaction mixture was allowed to warm to room temperature and stirred for 1 h before being cooled on ice, followed by the addition of 3 M HCl (40 mL). The aqueous layer was extracted with $Et_2O$ (3 × 40 mL), dried over anhydrous $Na_2SO_4$, and

concentrated under reduced pressure to yield the crude product *E*-3,5-hexadienoic acid (**o**) as an orange oil, which was used directly in the next step without further purification.

## 9.3. Synthesis of Imides

**General Procedure 5**

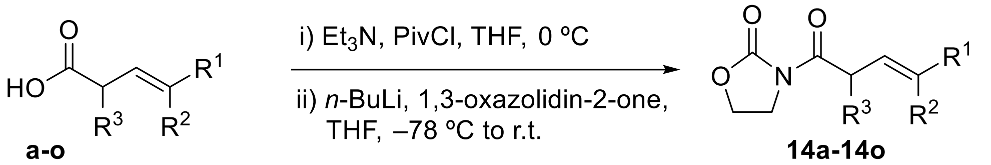

Neat $Et_3N$ (1.3 equiv.) and PivCl (1.3 equiv.) were added to a 0.2 M solution of the corresponding carboxylic acid (1.3 equiv.) in THF at 0 °C under nitrogen atmosphere. The resulting mixture was stirred at this temperature for 90 min, and it was cooled to −78 °C. Meanwhile, a 2.5 M solution of *n*-BuLi in hexanes (1.1 equiv.) was added dropwise to a 0.4 M solution of 1,3-oxazolidin-2-one (1.0 equiv.) in THF under nitrogen at −78 °C and stirred for 15 min. The resulting solution was added to the first mixture via cannula. The reaction mixture was stirred at −78 °C for 20 min and it was allowed to warm to r.t. and stirred for 2 h.
Then, it was quenched with sat. $NH_4Cl$ and extracted with EtOAc. The combined organic extracts were washed with sat. $NaHCO_3$, brine and dried with anhydrous $MgSO_4$. The volatiles were evaporated under reduced pressure and the residue was purified using flash column chromatography.

***N*-(3-Butenoyl)-1,3-oxazolidin-2-one (14a)**

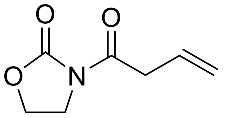

It was prepared following General Procedure 5 from 3-butenoic acid (0.60 mL, 7.0 mmol), $Et_3N$ (0.98 mL, 7.0 mmol), PivCl (0.86 mL, 7.0 mmol), 1,3-oxazolidin-2-one (470 mg, 5.4 mmol) and *n*-BuLi (2.5 M in hexanes, 2.36 mL, 5.9 mmol). Purification of the crude product by flash column chromatography (70:30 hexanes/EtOAc) afforded **14a** (482 mg, 3.1 mmol, 57% yield) as a yellowish oil. $\mathbf{R_f}$ (70:30 hexanes/EtOAc) 0.3; **IR** (ATR) ν 2920, 2851, 1769, 1692, 1644, 1525, 1478, 1426, 1385, 1362, 1296, 1195, 1101, 1037, 1019 cm$^{-1}$; **$^1$H NMR** (500 MHz, $CDCl_3$) δ 6.04–5.92 (1H, m, C<u>H</u>=$CH_xH_y$), 5.22 (1H, dq, *J* = 5.4, 1.5 Hz, CH=C<u>$H_x$</u>$H_y$), 5.20–5.18 (1H, m, CH=$CH_x$<u>$H_y$</u>), 4.45–4.38 (2H, m, OC<u>$H_2$</u>), 4.05–3.99 (2H, m, NC<u>$H_2$</u>), 3.71 (2H, dt, *J* = 6.8, 1.5 Hz, COC<u>$H_2$</u>); **$^{13}$C{$^1$H} NMR** (126 MHz, $CDCl_3$) δ 171.3 (C), 153.5 (C), 129.7 (CH), 119.2 ($CH_2$), 62.1 ($CH_2$), 42.5 ($CH_2$), 39.8 ($CH_2$); **HRMS** (+ESI): *m/z* calcd. for $C_7H_{10}NO_3$ $[M+H]^+$: 156.0655, found: 156.0652.

***N*-[(*E*)-3-Pentenoyl]-1,3-oxazolidin-2-one (14b)**

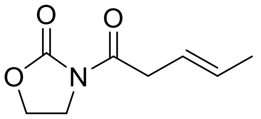

It was prepared following General Procedure 5 from (*E*)-3-pentenoic acid (0.66 mL, 6.5 mmol), $Et_3N$ (0.91 mL, 6.5 mmol), PivCl (0.80 mL, 6.5 mmol), 1,3-oxazolidin-2-one (435 mg, 5.0 mmol) and *n*-BuLi (2.5 M in hexanes, 2.2 mL, 5.5 mmol). Purification of the crude product by flash

column chromatography (from 70:30 to 60:40 hexanes/EtOAc) afforded **14b** (682 mg, 4.0 mmol, 81%, yield) as a white solid. **Mp** 36–38 $^{o}$C; **R$_f$** (60:40 hexanes/EtOAc) 0.3; **IR** (ATR) ν 2993, 2967, 2924, 2855, 1759, 1694, 1482, 1388, 1364, 1306, 1247, 1228, 1206, 1196, 1101, 978, 764, 695 cm$^{-1}$; **$^1$H NMR** (500 MHz, $CDCl_3$) δ 5.68–5.53 (2H, m, CH=CH), 4.44–4.37 (2H, m, $OCH_2$), 4.06–3.97 (2H, m, $NH_2$), 3.63 (2H, d, *J* = 5.3 Hz, $COCH_2$), 1.70 (3H, d, *J* = 4.7 Hz, $CH_3$); **$^{13}$C{$^1$H} NMR** (126 MHz, $CDCl_3$) δ 172.1 (C), 153.6 (C), 130.2 (CH), 122.2 (CH), 62.2 ($CH_2$), 42.6 ($CH_2$), 38.9 ($CH_2$), 18.1 ($CH_3$); **HRMS** (+ESI): *m/z* calcd. for $C_8H_{11}NNaO_3$ [M+Na]$^+$: 192.0631, found: 192.0632.

### *N*-[(*E*)-3-Hexenoyl]-1,3-oxazolidin-2-one (14c)

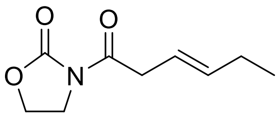

It was prepared following General Procedure 5 from (*E*)-3-hexenoic acid (830 mg, 7.3 mmol), $Et_3N$ (1.0 mL, 7.3 mmol), PivCl (0.9 mL, 7.3 mmol), 1,3-oxazolidin-2-one (488 mg, 5.6 mmol) and *n*-BuLi (2.5 M in hexanes, 3.9 mL, 6.2 mmol). Purification of the crude product by flash column chromatography (70:30 hexanes/EtOAc) afforded **14c** (457 mg, 2.5 mmol, 45% yield) as a yellowish oil. **R$_f$** (60:40 hexanes/EtOAc) 0.4; **IR** (ATR) ν 2961, 2920, 2851, 1771, 1694, 1479, 1461, 1385, 1363, 1329, 1292, 1186, 1104, 1038, 1001 cm$^{-1}$; **$^1$H NMR** (500 MHz, $CDCl_3$) δ 5.72–5.50 (2H, m, CH=CH), 4.44–4.39 (2H, m, $OCH_2$), 4.05–3.99 (2H, m, $NCH_2$), 3.64 (2H, dd, *J* = 6.6, 1.1 Hz, $COCH_2$), 2.12–2.00 (2H, m, $CH_2CH_3$), 0.99 (3H, t, *J* = 7.5 Hz, $CH_2CH_3$); **$^{13}$C{$^1$H} NMR** (126 MHz, $CDCl_3$) δ 172.0 (C), 153.5 (C), 137.1 (CH), 119.9 (CH), 62.1 ($CH_2$), 42.5 ($CH_2$), 38.8 ($CH_2$), 25.6 ($CH_2$), 13.4 ($CH_3$); **HRMS** (+ESI): *m/z* calcd. for $C_9H_{14}NO_3$ [M+H]$^+$: 184.0968, found: 184.0962.

### *N*-[(*E*)-5-Methyl-3-hexenoyl]-1.3-oxazolidin-2-one (14d)

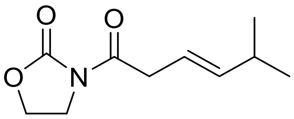

It was prepared following General Procedure 5 from (*E*)-5-methyl-3-hexenoic acid (812 mg, 6.3 mmol), $Et_3N$ (0.9 mL, 6.3 mmol), PivCl (0.8 mL, 6.3 mmol), 1,3-oxazolidin-2-one (418 mg, 4.8 mmol) and *n*-BuLi (2.5 M in hexanes, 3.3 mL, 5.3 mmol). Purification of the crude product by flash column chromatography (50:50 DCM/toluene) afforded **14d** (880 mg, 4.5 mmol, 93% yield) as a yellowish solid with very low melting point. **R$_f$** (60:40 hexanes/EtOAc) 0.3; **IR** (ATR) ν 2956, 2869, 2359, 1760, 1698, 1519, 1485, 1463, 1383, 1361, 1321, 1284, 1220, 1190, 1096, 1038, 1009 cm$^{-1}$; **$^1$H NMR** (500 MHz, $CDCl_3$) δ 5.63–5.48 (2H, m, CH=CH), 4.43–4.37 (2H, m, $OCH_2$), 4.04–3.98 (2H, m, $NCH_2$), 3.63 (2H, d, *J* = 6.1 Hz, $COCH_2$), 2.36–2.24 (1H, m, CH), 0.98 (6H, d, *J* = 6.7 Hz, 2 × $CH_3$); **$^{13}$C{$^1$H} NMR** (126 MHz, $CDCl_3$) δ 172.1 (C), 153.5 (C), 142.4 (CH), 118.1 (CH), 62.1 ($CH_2$), 42.5 ($CH_2$), 38.7 ($CH_2$), 31.1 (CH), 22.3 ($CH_3$); **HRMS** (+ESI): *m/z* calcd. for $C_{10}H_{16}NO_3$ [M+H]$^+$: 198.1125, found: 198.1119.

### *N*-[(*E*)-5-Phenyl-3-pentenoyl]-1,3-oxazolidin-2-one (14e)

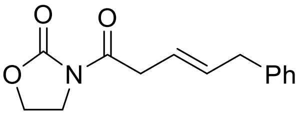


It was prepared following General Procedure 5 from (*E*)-5-phenyl-3-pentenoic acid 1 (1.365 g, 7.7 mmol), $Et_3N$ (1.1 mL, 7.7 mmol), PivCl (0.95 mL, 7.7 mmol), 1,3-oxazolidinone (514 mg, 5.9 mmol) and *n*-BuLi (2.5 M in hexanes, 4.1 mL, 6.5 mmol). Purification of the crude product by flash column chromatography (60:40 hexanes/EtOAc) afforded **14e** (477 mg, 1.9 mmol, 33% yield) as a yellowish solid. **Mp** 40–43 °C; $\mathbf{R_f}$ (60:40 hexanes/EtOAc) 0.3; **IR** (ATR) ν 3023, 2921, 2852, 1764, 1698, 1600, 1492, 1475, 1452, 1431, 1384, 1362, 1320, 1273, 1249, 1228, 1194, 1110, 1086, 1070, 1034, 1007 cm$^{-1}$; **$^{1}$H NMR** (500 MHz, $CDCl_3$) δ 7.32–7.27 (2H, m, ArH), 7.23–7.16 (3H, m, ArH), 5.83–5.65 (2H, m, CH=CH), 4.43–4.37 (2H, m, $OCH_2$), 4.05–3.98 (2H, m, $NCH_2$), 3.70 (2H, dd, *J* = 6.6, 1.1 Hz, $COCH_2$), 3.40 (2H, d, *J* = 6.6 Hz, $CH_2Ph$); **$^{13}$C{$^{1}$H} NMR** (126 MHz, $CDCl_3$) δ 171.7 (C), 153.5 (C), 140.1 (C), 133.8 (CH), 128.5 (CH), 128.4 (CH), 126.1 (CH), 122.6 (CH), 62.1 ($CH_2$), 42.5 ($CH_2$), 39.0 ($CH_2$), 38.7 ($CH_2$); **HRMS** (+ESI): *m/z* calcd. for $C_{14}H_{16}NO_3$ [M+H]$^+$: 246.1125, found: 246.1118.

### *N*-[(*E*)-5,9-Dimethyl-3,8-decadienoyl]-1,3-oxazolidin-2-one (14f)

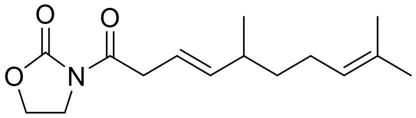


It was prepared following General Procedure 5 from (*E*)-5,9-dimethyl-3,8-decadienoic acid (1.12 g, 5.7 mmol), $Et_3N$ (0.8 mL, 5.7 mmol), PivCl (0.70 mL, mmol), 1,3-oxazolidin-2-one (383 mg, 4.4 mmol) and *n*-BuLi (2.5 M in hexanes, 1.9 mL, 4.8 mmol). Purification of the crude product by flash column chromatography (from 90:10 to 70:30 hexanes/EtOAc) afforded **14f** (670 mg, 2.5 mmol, 57% yield) as a yellowish oil. $\mathbf{R_f}$ (80:20 hexanes/EtOAc) 0.3; **IR** (ATR) ν 2962, 2914, 1775, 1697, 1479, 1453, 1384, 1288, 1191, 1106, 1039, 1007 cm$^{-1}$; **$^{1}$H NMR** (500 MHz, $CDCl_3$) δ 5.58–5.43 (2H, m, CH=CH), 5.11–5.03 (1H, m, C=CH), 4.43–4.36 (2H, m, $OCH_2$), 4.04–3.98 (2H, m, $NCH_2$), 3.64 (2H, d, *J* = 6.2 Hz, $COCH_2$), 2.14 (1H, hept, *J* = 6.9 Hz, $CHCH_3$), 1.96–1.90 (2H, m, $CH_2CH=CH(CH_3)_2$), 1.66 (3H, d, *J* = 1.4 Hz, $CH_3$), 1.58 (3H, s, $CH_3$), 1.32–1.26 (2H, m, $CHCH_2$), 0.97 (3H, d, *J* = 6.7 Hz, $CHCH_3$); **$^{13}$C{$^{1}$H} NMR** (126 MHz, $CDCl_3$) δ 172.0 (C), 153.5 (C), 141.2 (CH), 131.3 (C), 124.6 (CH), 119.3 (CH), 62.1 ($CH_2$), 42.5 ($CH_2$), 38.8 ($CH_2$), 36.9 ($CH_2$), 36.3 (CH), 25.8 ($CH_2$), 25.7 ($CH_3$), 20.4 ($CH_3$), 17.7 ($CH_3$); **HRMS** (+ESI): *m/z* calcd. for $C_{15}H_{24}NO_3$ [M+H]$^+$: 266.1751, found: 266.1745.

### *N*-[(*E*)-3-Dodecen-11-ynoyl]-1,3-oxazolidin-2-one (14g)

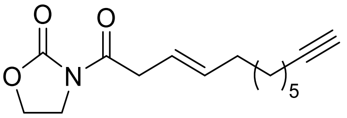


It was prepared following General Procedure 5 from (*E*)-3-dodecen-11-ynoic acid (1.24 g, 6.4 mmol), $Et_3N$ (0.90 mL, 6.4 mmol), PivCl (0.80 mL, 6.4 mmol), 1,3-oxazolidin-2-one (427 mg, 4.9 mmol) and *n*-BuLi (2.5 M in hexanes, 2.2 mL, 5.4 mmol). Purification of the crude product by flash column chromatography (70:30 hexanes/EtOAc) afforded **14g** (750 mg, 2.8 mmol, 58% yield) as a white solid. **Mp** 69–72 °C; $\mathbf{R_f}$ (70:30 hexanes/EtOAc) 0.3; **IR** (ATR) ν 3241, 2921,

2850, 2358, 2342, 1765, 1691, 1473, 1460, 1441, 1392, 1371, 1343, 1321, 1292, 1257, 1242, 1226, 1212, 1190, 1115, 1083, 1040, 1013 cm$^{-1}$; **$^{1}$H NMR** (400 MHz, $CDCl_3$) δ 5.67–5.50 (2H, m, CH=CH), 4.44–4.37 (2H, m, OCH$_2$), 4.04–3.97 (2H, m, NCH$_2$), 3.64 (2H, d, $J$ = 5.4 Hz, COCH$_2$), 2.17 (2H, td, $J$ = 7.0, 2.7 Hz, CH$_2$C≡CH), 2.08–2.00 (2H, m, CH=CHCH$_2$), 1.93 (1H, t, $J$ = 2.7 Hz, C≡CH), 1.55–1.47 (2H, m, CH$_2$), 1.43–1.23 (6H, m, 3 × CH$_2$); **$^{13}$C{$^{1}$H} NMR** (101 MHz, $CDCl_3$) δ 172.0 (C), 153.5 (C), 135.5 (CH), 121.0 (CH), 84.7 (C), 68.1 (CH), 62.1 (CH$_2$), 42.5 (CH$_2$), 38.8 (CH$_2$), 32.5 (CH$_2$), 29.0 (CH$_2$), 28.5 (CH$_2$), 28.5 (CH$_2$), 28.4 (CH$_2$), 18.4 (CH$_2$); **HRMS** (+ESI): *m/z* calcd. for $C_{15}H_{22}NO_3$ [M+H]$^+$: 264.1594, found: 264.1594.

**_N_-[(_E_)-8-Chloro-3-octenoyl]-1,3-oxazolidin-2-one (14h)**

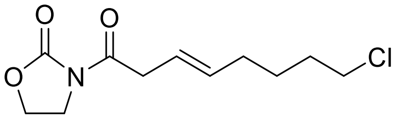

It was prepared following General Procedure 5 from (*E*)-8-chloro-3-octenoic acid (517 mg, 2.9 mmol), $Et_3N$ (0.40 mL, 2.9 mmol), PivCl (0.40 mL, 2.9 mmol), 1,3-oxazolidin-2-one (192 mg, 2.2 mmol) and *n*-BuLi (2.5 M in hexanes, 1.0 mL, 2.5 mmol). Purification of the crude product by flash column chromatography (70:30 hexanes/EtOAc) afforded **14h** (226 mg, 0.9 mmol, 42% yield) as a yellowish oil. **$R_f$** (70:30 hexanes/EtOAc) 0.2; **IR** (ATR) ν 2917, 2849, 1766, 1695, 1471, 1461, 1373, 1337, 1312, 1273, 1222, 1113, 1086, 1039, 1008 cm$^{-1}$; **$^{1}$H NMR** (500 MHz, $CDCl_3$) δ 5.62–5.59 (2H, m, CH=CH), 4.45–4.39 (2H, m, OCH$_2$), 4.05–3.99 (2H, m, NCH$_2$), 3.67–3.64 (2H, m, COCH$_2$), 3.53 (2H, t, $J$ = 6.7 Hz, ClCH$_2$), 2.12–2.05 (2H, m, CHCH$_2$), 1.82–1.74 (2H, m, ClCH$_2$CH$_2$), 1.58–1.47 (2H, m, CH$_2$CH$_2$CH$_2$); **$^{13}$C{$^{1}$H} NMR** (126 MHz, $CDCl_3$) δ 171.8 (C), 153.5 (C), 134.6 (CH), 121.7 (CH), 62.1 (CH$_2$), 44.9 (CH$_2$), 42.5 (CH$_2$), 38.7 (CH$_2$), 32.0 (CH$_2$), 31.7 (CH$_2$), 26.3 (CH$_2$); **HRMS** (+ESI): *m/z* calcd. for $C_{11}H_{17}ClNO_3$ [M+H]$^+$: 246.0891, found: 246.0893.

**_N_-[(_E_)-11-Oxo-3-dodecenoyl]-1,3-oxazolidin-2-one (14i)**

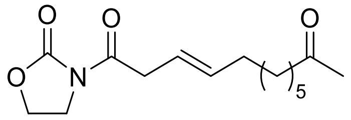

Imide **14g** (395 mg, 1.5 mmol, 1.0 equiv.) was combined with $Hg(OAc)_2$ (48 mg, 0.1 mmol, 0.1 equiv.) in methanol (45 mL). Six drops of concentrated sulfuric acid (98%) were added to the solution, which was then heated in an oil bath at 60 °C and stirred for 3 h. Once the reaction was complete, the mixture was cooled to room temperature, and 2 M HCl (2 mL) was added, followed by stirring for 2 min. The solution was subsequently neutralized using sat. $NaHCO_3$ (4 mL). The organic compounds were extracted using $Et_2O$ (3 × 15 mL), and the combined organic layers were dried over anhydrous $MgSO_4$. After filtration, the solvent was removed under reduced pressure. The crude product was purified by flash column chromatography (from 70:30 to 60:40 hexane/EtOAc) to yield **14i** as a white solid (312 mg, 1.1 mmol, 74% yield). **Mp** 51–53 °C; **$R_f$** (50:50 hexanes/EtOAc) 0.4; **IR** (ATR) ν 2988, 2922, 2850, 1770, 1702, 1685, 1478, 1459, 1387, 1360, 1219, 1198, 1116, 1090, 1042, 960, 757, 704 cm$^{-1}$; **$^{1}$H NMR** (400 MHz, $CDCl_3$) δ 5.65–5.49 (2H, m, CH=CH), 4.46–4.35 (2H, m, OCH$_2$), 4.05–3.96 (2H, m, NCH$_2$), 3.63 (2H, d, $J$ = 5.6 Hz, COCH$_2$CH=CH), 2.40 (2H, t, $J$ = 7.5 Hz, COCH$_2$CH$_2$), 2.12 (3H, s, CH$_3$), 2.02

(2H, dt, *J* = 7.2, 5.7 Hz, $CH{=}CHC\underline{H}_2$), 1.60–1.48 (2H, m, $COCH_2C\underline{H}_2$), 1.41–1.19 (6H, m, 3 × $C\underline{H}_2$); **$^{13}$C{$^1$H} NMR** (101 MHz, $CDCl_3$) δ 209.4 (C), 172.1 (C), 153.6 (C), 135.5 (CH), 121.1 (CH), 62.2 ($CH_2$), 43.9 ($CH_2$), 42.6 ($CH_2$), 38.9 ($CH_2$), 32.6 ($CH_2$), 30.0 ($CH_3$), 29.1 ($CH_2$), 29.0 ($CH_2$), 28.9 ($CH_2$), 23.9 ($CH_2$); **HRMS** (+ESI): *m/z* calcd. for $C_{15}H_{23}NNaO_4$ $[M+Na]^+$: 304.1519, found: 304.1510.

### *N*-[(*E*)-12-Methoxy-12-oxo-3-dodecenoyl]-1,3-oxazolidin-2-one (14j)

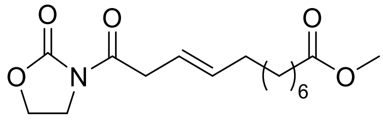


It was prepared following General Procedure 5 from (*E*)-12-methoxy-12-oxo-3-dodecenoic acid (1.15 g, 5.0 mmol), $Et_3N$ (0.70 mL, 5.0 mmol), PivCl (0.6 mL, 5.0 mmol), 1,3-oxazolidin-2-one (331 mg, 3.8 mmol) and *n*-BuLi (2.5 M in hexanes, 1.7 mL, 4.2 mmol). Purification of the crude product by flash column chromatography (from 70:30 to 50:50 hexanes/EtOAc) afforded **14j** (712 mg, 2.3 mmol, 60% yield) as a white solid. **Mp** 35–37 ºC; **$R_f$** (80:20 hexanes/EtOAc) 0.3; **IR** (ATR) ν 2919, 2850, 2363, 1766, 1736, 1693, 1471, 1439, 1408, 1381, 1343, 1296, 1279, 1254, 1238, 1195, 1167, 1114, 1087, 1040, 1012 $cm^{-1}$; **$^1$H NMR** (400 MHz, $CDCl_3$) δ 5.66–5.49 (2H, m, $C\underline{H}{=}C\underline{H}$), 4.43–4.37 (2H, m, $OC\underline{H}_2$), 4.04–3.98 (2H, m, $NC\underline{H}_2$), 3.65 (3H, s, $OC\underline{H}_3$), 3.64 (2H, d, *J* = 5.4 Hz, $NCOC\underline{H}_2$), 2.29 (2H, t, *J* = 7.5 Hz, $CH_3OCOC\underline{H}_2$), 2.07–1.97 (2H, m, $CH{=}CHC\underline{H}_2$), 1.60 (2H, p, *J* = 7.6 Hz, $COCH_2C\underline{H}_2$), 1.40–1.22 (8H, m, 4 × $C\underline{H}_2$); **$^{13}$C{$^1$H} NMR** (101 MHz, $CDCl_3$) δ 174.3 (C), 172.0 (C), 153.5 (C), 135.5 (CH), 120.9 (CH), 62.1 ($CH_2$), 51.5 ($CH_3$), 42.5 ($CH_2$), 38.8 ($CH_2$), 34.1 ($CH_2$), 32.5 ($CH_2$), 29.1 ($CH_2$), 29.1 ($CH_2$), 29.0 ($CH_2$), 28.9 ($CH_2$), 24.9 ($CH_2$); **HRMS** (+ESI): *m/z* calcd. for $C_{16}H_{26}NO_5$ $[M+H]^+$: 312.1805, found: 312.1800.

### *N*-(4-Methyl-3-pentenoyl)-1,3-oxazolidin-2-one (14k)

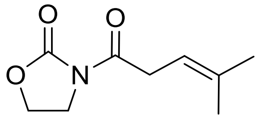


It was prepared following General Procedure 5 using the 67:33 mixture of **k** and its α,β-unsaturated isomer (679 mg, 4.5 mmol), $Et_3N$ (0.63 mL, 4.5 mmol), PivCl (0.55 mL, 4.5 mmol), 1,3-oxazolidin-2-one (305 mg, 3.5 mmol) and *n*-BuLi (2.5 M in hexanes, 1.5 mL, 3.8 mmol). Purification of the crude product by flash column chromatography (70:30 hexanes/EtOAc) afforded a 90:10 mixture of **14k** and its α,β-unsaturated isomer respectively (400 mg, 2.2 mmol, 62% yield), as a colorless oil. **$R_f$** (70:30 hexanes/EtOAc) 0.3; **IR** (ATR) ν 2969, 2918, 2861, 1769, 1694, 1478, 1385, 1362, 1325, 1217, 1172, 1101, 1034, 1004, 958, 758, 699 $cm^{-1}$; *Major*: **$^1$H NMR** (400 MHz, $CDCl_3$) δ 5.35 (1H, tqq, *J* = 7.2, 2.9, 1.4 Hz, $C\underline{H}{=}C$), 4.45–4.35 (2H, m, $OC\underline{H}_2$), 4.04–3.97 (2H, m, $NC\underline{H}_2$), 3.64 (2H, d, *J* = 7.2 Hz, $COC\underline{H}_2$), 1.75 (3H, s, $C\underline{H}_3$), 1.66 (3H, s, $C\underline{H}_3$); *Major*: **$^{13}$C{$^1$H} NMR** (101 MHz, $CDCl_3$) δ 172.1 (C), 153.6 (C), 136.4 (C), 115.1 (CH), 62.1 ($CH_2$), 42.7 ($CH_2$), 34.7 ($CH_2$), 25.9 ($CH_3$), 18.3 ($CH_3$); **HRMS** (+ESI): *m/z* calcd. for $C_9H_{13}NNaO_3$ $[M+Na]^+$: 206.0788, found: 206.0782.

### *N*-[(2-(1-Cyclohexen-1-yl)acetyl)]-1,3-oxazolidin-2-one (14l)

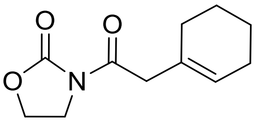

It was prepared following General Procedure 5 from 2-(1-cyclohexen-1-yl)acetic acid (1.01 g, 7.2 mmol), $Et_3N$ (1.01 mL, 7.2 mmol), PivCl (0.89 mL, 7.2 mmol), 1,3-oxazolidin-2-one (484 mg, 5.6 mmol) and *n*-BuLi (2.5 M in hexanes, 2.7 mL, 6.1 mmol). Purification of the crude product by flash column chromatography (from 80:20 to 60:40 hexanes/EtOAc) afforded **14l** (815 mg, 3.9 mmol, 70% yield) as a white solid. **Mp** 49–50 °C; **$R_f$** (70:30 hexanes/EtOAc) 0.3; **IR** (ATR) ν 2988, 2926, 2857, 2838, 1763, 1690, 1422, 1385, 1362, 1329, 1312, 1224, 1187, 1137, 1113, 1038, 975, 755, 718 $cm^{-1}$; **$^1H$ NMR** (400 MHz, $CDCl_3$) δ 5.57–5.50 (1H, m, C=C<u>H</u>), 4.44–4.35 (2H, m, OC<u>H</u>$_2$), 4.06–3.97 (2H, m, NC<u>H</u>$_2$), 3.56 (3H, s, COC<u>H</u>$_2$), 2.07–1.94 (4H, m, CC<u>H</u>$_2$CH$_2$, C=CHC<u>H</u>$_2$), 1.68–1.51 (4H, m, CH$_2$(C<u>H</u>$_2$)$_2$CH$_2$); **$^{13}C\{^1H\}$ NMR** (101 MHz, $CDCl_3$) δ 171.8 (C), 153.5 (C), 131.1 (C), 125.9 (CH), 62.0 ($CH_2$), 43.5 ($CH_2$), 42.7 ($CH_2$), 28.8 ($CH_2$), 25.4 ($CH_2$), 22.9 ($CH_2$), 22.1 ($CH_2$); **HRMS** (+ESI): *m/z* calcd. for $C_{11}H_{15}NNaO_3$ $[M+Na]^+$: 232.0944, found: 232.0942.

### *N*-(2-Methyl-3-butenoyl)-1,3-oxazolidin-2-one (14m)

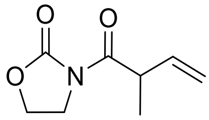

It was prepared following General Procedure 5 from 2-methylbut-3-enoic acid (0.67 mL, 6.5 mmol), $Et_3N$ (0.91 mL, 6.5 mmol), PivCl (0.80 mL, 6.5 mmol), 1,3-oxazolidin-2-one (435 mg, 5.0 mmol) and *n*-BuLi (2.5 M in hexanes, 2.2 mL, 5.5 mmol). Purification of the crude product by flash column chromatography (from 80:20 to 60:40 hexanes/EtOAc) afforded **14m** (575 mg, 3.4 mmol, 68% yield) as a colorless oil. **$R_f$** (60:40 hexanes/EtOAc) 0.5; **IR** (ATR) ν 3084, 2980, 2924, 2853, 1769, 1692, 1383, 1360, 1245, 1198, 1109, 1038, 999, 945, 922, 758, 706 $cm^{-1}$; **$^1H$ NMR** (400 MHz, $CDCl_3$) δ 6.05–5.77 (1H, m, C<u>H</u>=CH$_2$), 5.36–4.97 (2H, m, CH=C<u>H</u>$_2$), 4.54–4.32 (3H, m, OC<u>H</u>$_2$, CH$_3$C<u>H</u>), 4.08–3.90 (2H, m, NC<u>H</u>$_2$), 1.28 (3H, d, $J$ = 6.9 Hz, C<u>H</u>$_3$); **$^{13}C\{^1H\}$ NMR** (101 MHz, $CDCl_3$) δ 175.0 (C), 153.2 (C), 137.0 (CH), 116.7 ($CH_2$), 62.0 ($CH_2$), 42.9 ($CH_2$), 41.5 (CH), 17.1 ($CH_3$); **HRMS** (+ESI): *m/z* calcd. for $C_8H_{11}NNaO_3$ $[M+Na]^+$: 192.0631, found: 192.0628.

### *N*-(3,4-Pentadienoyl)-1,3-oxazolidin-2-one (14n)

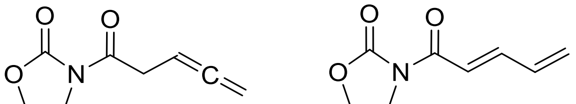

**14n**

It was prepared following General Procedure 5 using the crude product **n**, $Et_3N$ (1.3 mL, 9.2 mmol), PivCl (1.1 mL, 9.2 mmol), 1,3-oxazolidin-2-one (618 mg, 7.1 mmol) and *n*-BuLi (2.5 M in hexanes, 3.1 mL, 7.8 mmol). Purification of the crude product by flash column chromatography (from 80:20 to 60:40 hexanes/EtOAc) afforded a 62:38 mixture of **14n** and its α,β-γ,δ-diunsaturated isomer respectively (243 mg, 1.45 mmol, 4% yield over three steps) as a white solid. **Mp** 60–77 °C; **$R_f$** (80:20 hexanes/EtOAc) 0.2; **IR** (ATR) ν 3081, 3060, 2989, 2913, 2853,

1765, 1677, 1620, 1590, 1478, 1388, 1362, 1327, 1277, 1223, 1206, 1109, 1032, 954, 870, 859, 757, 703 $cm^{-1}$; **14n**: **$^1$H NMR** (500 MHz, $CDCl_3$) δ 5.33 (1H, tt, *J* = 7.2, 6.7 Hz, CH=C=$CH_2$), 4.76 (2H, dt, *J* = 6.7, 2.9 Hz, CH=C=$CH_2$), 4.46–4.39 (2H, m, $OCH_2$), 4.05–3.99 (2H, m, $NCH_2$), 3.67 (2H, dt, *J* = 7.2, 2.9 Hz, $COCH_2$); **α,β-γ,δ isomer**: **$^1$H NMR** (400 MHz, $CDCl_3$) δ 7.45 (1H, ddt, *J* = 15.1, 10.7, 0.7 Hz, COCH=CH), 7.33 (1H, d, *J* = 15.1 Hz, COCH=CH), 6.63–6.50 (1H, m, COCH=CH-CH=$CH_ZH_E$), 5.68 (1H, ddt, *J* = 16.9, 1.4, 0.7 Hz, COCH=CH-CH=$CH_ZH_E$), 5.57 (1H, ddt, *J* = 9.9, 1.4, 0.7 Hz, COCH=CH-CH=$CH_ZH_E$), 4.47–4.39 (2H, m, $OCH_2$), 4.13–4.05 (2H, m, $NCH_2$); **14n**: **$^{13}$C{$^1$H} NMR** (126 MHz, $CDCl_3$) δ 209.6 (C), 171.1 (C), 83.0 (CH), 76.0 ($CH_2$), 62.3 ($CH_2$), 42.6 ($CH_2$), 35.4 ($CH_2$); **α,β-γ,δ isomer**: **$^{13}$C{$^1$H} NMR** (101 MHz, $CDCl_3$) δ 165.4 (C), 153.6 (C), 146.4 (CH), 135.3 (CH), 127.1 ($CH_2$), 120.7 (CH), 62.2 ($CH_2$), 42.9 ($CH_2$); **HRMS** (+ESI): *m/z* calcd. for $C_8H_9NNaO_3$ $[M+Na]^+$: 190.0475, found: 190.0473; **α,β-γ,δ isomer**: **HRMS** (+ESI): *m/z* calcd. for $C_8H_9NNaO_3$ $[M+Na]^+$: 190.0475, found: 190.0473.

**_N_-[(_E_)-(3,5-Hexadienoyl)]-1,3-oxazolidin-2-one (14o)**

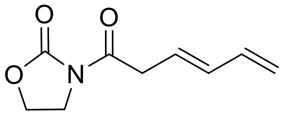

It was prepared following General Procedure 5 from (*E*)-3,5-Hexadienoic acid (819 mg, 7.3 mmol), $Et_3N$ (1.0 mL, 7.3 mmol), PivCl (0.9 mL, 7.3 mmol), 1,3-oxazolidin-2-one (488 mg, 5.6 mmol) and *n*-BuLi (2.5 M in hexanes, 2.5 mL, 6.2 mmol). Purification of the crude product by flash column chromatography (from 70:30 to 50:50 hexanes/EtOAc) afforded **14o** (319 mg, 1.8 mmol, 31% yield) as a yellow solid. **Mp** 70–74 °C; **$R_f$** (70:30 hexanes/EtOAc) 0.2; **IR** (ATR) ν 2974, 2913, 1761, 1687, 1484, 1388, 1362, 1277, 1223, 1208, 1114, 1038, 1008, 952, 911, 833, 759, 706 $cm^{-1}$; **$^1$H NMR** (400 MHz, $CDCl_3$) δ 6.34 (1H, dt, *J* = 16.8, 10.2 Hz, CH=$CH_ZH_E$), 6.25–6.13 (1H, m, $CH_2$CH=CH), 5.93–5.71 (1H, m, $CH_2$CH=CH), 5.22–5.13 (1H, m, CH=$CH_ZH_E$), 5.10–5.03 (1H, m, CH=$CH_ZH_E$), 4.47–4.37 (2H, m, $OCH_2$), 4.07–3.97 (2H, m, $NCH_2$), 3.74 (2H, dd, *J* = 7.1, 1.4 Hz, $COCH_2$); **$^{13}$C{$^1$H} NMR** (101 MHz, $CDCl_3$) δ 171.3 (C), 153.6 (C), 136.5 (CH), 135.1 (CH), 125.1 (CH), 117.3 ($CH_2$), 62.2 ($CH_2$), 42.6 ($CH_2$), 38.7 ($CH_2$); **HRMS** (+ESI): *m/z* calcd. for $C_9H_{12}NO_3$ $[M+H]^+$: 182.0812, found: 182.0810.

## 10. Scope of the γ-Aminoxylation Reaction

**General Procedure 6**

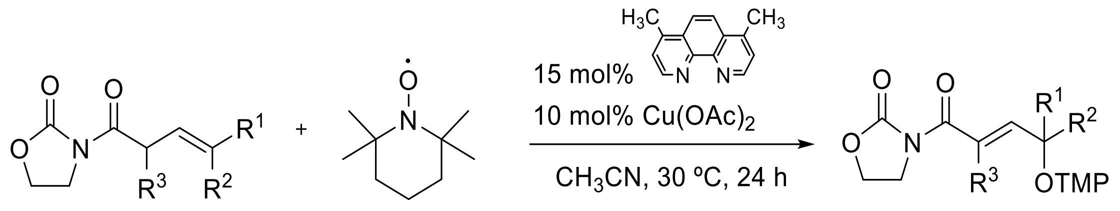


**14a-14o** **17a-17o**

A round bottom flask equipped with a magnetic stirring bar was charged with *N*-(β,γ-unsaturated acyl)-1,3-oxazolidin-2-one **14** (0.3 mmol, 1.0 equiv), anhydrous $Cu(OAc)_2$ (5.45 mg, 30 μmol, 0.10 equiv, 10 mol%), 4,7-dimethyl-1,10-phenanthroline (9.37 mg, 45 μmol, 0.15 equiv, 15 mol%), and TEMPO (93.8 mg, 0.6 mmol, 2.0 equiv), followed by the addition of acetonitrile (1.5 mL) to get a 0.2 M solution. The resultant mixture was stirred under nitrogen atmosphere in a water bath at 30 °C for 16 h and then quenched with sat. $NH_4Cl$ (1.5 mL).
The aqueous layer was extracted with EtOAc (3 × 2 mL). The combined organic extracts were dried with anhydrous $MgSO_4$ and concentrated *in vacuo*. The resulting residue was purified by column chromatography to yield the α,β-unsaturated Cγ aminoxylated adduct **17**.

***N*-[(*E*)-4-((2,2,6,6-Tetramethylpiperidin-1-yl)oxy)-2-butenoyl]-1,3-oxazolidin-2-one (17a)**

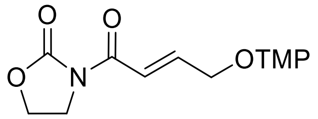


It was prepared following General Procedure 6 from **14a** (46.6 mg, 0.30 mmol), $Cu(OAc)_2$ (5.45 mg, 30 μmol, 10 mol%), 4,7-dimethyl-1,10-phenanthroline (9.37 mg, 45 μmol, 15 mol%), and TEMPO (93.8 mg, 0.60 mmol). Purification of the crude product by flash column chromatography (80:20 hexanes/EtOAc) afforded **17a** (75 mg, 0.24 mmol, 81% yield) as a white solid. **Mp** 96–99 °C; $\mathbf{R_f}$ (80:20 hexanes/EtOAc) 0.2; **IR** (ATR) ν 3000, 2970, 2927, 2865, 2358, 1787, 1682, 1637, 1479, 1468, 1446, 1389, 1364, 1346, 1332, 1275, 1228, 1196, 1131, 1112, 1085, 1042, 1024 $cm^{-1}$; $^1$**H NMR** (400 MHz, $CDCl_3$) δ 7.54 (1H, dt, *J* = 15.6, 2.2 Hz, COCH=CH), 7.12 (1H, dt, *J* = 15.6, 4.2 Hz, COCH=CH), 4.54 (2H, dd, *J* = 4.2, 2.2 Hz, $NOCH_2$), 4.46–4.39 (2H, m, $OCH_2$), 4.11–4.05 (2H, m, $NCH_2$), 1.66–1.51 (1H, m, $CH_xH_y$), 1.48–1.42 (4H, m, 2 × $CH_2$), 1.37–1.28 (1H, m, $CH_xH_y$), 1.15 (12H, s, 4 × $CH_3$); $^{13}$**C{$^1$H} NMR** (101 MHz, $CDCl_3$) δ 165.2 (C), 153.4 (C), 146.1 (CH), 119.0 (CH), 76.3 ($CH_2$), 62.0 ($CH_2$), 60.0 (C), 42.7 ($CH_2$), 39.7 ($CH_2$), 32.8 ($CH_3$), 20.1 ($CH_3$), 17.1 ($CH_2$); **HRMS** (+ESI): *m/z* calcd. for $C_{16}H_{27}N_2O_4$ $[M+H]^+$: 311.1965, found: 311.1969.

***N*-[(*E*)-4-((2,2,6,6-Tetramethylpiperidin-1-yl)oxy)-2-pentenoyl]-1,3-oxazolidin-2-one (17b)**

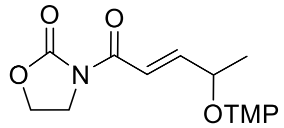


It was prepared following General Procedure 6 from **14b** (50.8 mg, 0.30 mmol), $Cu(OAc)_2$ (5.45 mg, 30 μmol, 10 mol%), 4,7-dimethyl-1,10-phenanthroline (9.37 mg, 45 μmol, 15 mol%), and TEMPO (93.8 mg, 0.60 mmol). Purification of the crude product by flash column chromatography (from 80:20 to 70:30 hexanes/EtOAc) afforded **17b** (96 mg, 0.3 mmol, 99% yield) as a white solid. **Mp** 54–56 °C; **$R_f$** (80:20 hexanes/EtOAc) 0.2; **IR** (ATR) ν 2999, 2973, 2932, 2872, 1778, 1677, 1633, 1478, 1388, 1366, 1346, 1299, 1232, 1193, 1131, 1107, 1042, 1027, 936, 760, 701 $cm^{-1}$; **$^1$H NMR** (400 MHz, $CDCl_3$) δ 7.32 (1H, dd, *J* = 15.5, 1.0 Hz, COC<u>H</u>=CH), 7.18 (1H, dd, *J* = 15.5, 6.9 Hz, COCH=C<u>H</u>), 4.59–4.48 (1H, m, NOC<u>H</u>), 4.45–4.36 (2H, m, OC<u>H</u>$_2$), 4.11–4.02 (2H, m, NC<u>H</u>$_2$), 1.69–1.38 (6H, m, 3 × C<u>H</u>$_2$), 1.31 (3H, d, *J* = 6.7 Hz, CHC<u>H</u>$_3$), 1.18 (3H, s, C<u>H</u>$_3$), 1.12 (3H, s, C<u>H</u>$_3$), 1.11 (3H, s, C<u>H</u>$_3$), 1.07 (3H, s, C<u>H</u>$_3$); **$^{13}$C{$^1$H} NMR** (101 MHz, $CDCl_3$) δ 165.2 (C), 153.5 (CH), 153.5 (C), 118.2 (CH), 79.6 (CH), 62.1 ($CH_2$), 59.9 (C), 59.7 (C), 42.8 ($CH_2$), 40.2 ($CH_2$), 40.2 ($CH_2$), 34.6 ($CH_3$), 34.2 ($CH_3$), 20.4 ($CH_3$), 20.3 ($CH_3$), 24.3 ($CH_3$), 17.3 ($CH_2$); **HRMS** (+ESI): *m/z* calcd. for $C_{17}H_{29}N_2O_4$ $[M+H]^+$: 325.2122, found: 325.2121.

***N*-[(*E*)4-((2,2,6,6-Tetramethylpiperidin-1-yl)oxy)-2-hexenoyl]-1,3-oxazolidin-2-one (17c)**

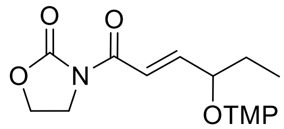


It was prepared following General Procedure 6 from **14c** (55.0 mg, 0.30 mmol), $Cu(OAc)_2$ (5.45 mg, 30 μmol, 10 mol%), 4,7-dimethyl-1,10-phenanthroline (9.37 mg, 45 μmol, 15 mol%), and TEMPO (93.8 mg, 0.60 mmol). Purification of the crude product by flash column chromatography (80:20 hexanes/EtOAc) afforded **17c** (92 mg, 0.27 mmol, 91% yield) as a brownish oil. **$R_f$** (80:20 hexanes/EtOAc) 0.2; **IR** (ATR) ν 2969, 2927, 1774, 1683, 1637, 1464, 1359, 1275, 1205, 1131, 1104, 1034 $cm^{-1}$; **$^1$H NMR** (500 MHz, $CDCl_3$) δ 7.29 (1H, d, *J* = 15.6 Hz, COC<u>H</u>=CH), 7.15 (1H, dd, *J* = 15.6, 7.9 Hz, COCH=C<u>H</u>), 4.46–4.40 (2H, m, OC<u>H</u>$_2$), 4.34 (1H, td, *J* = 7.8, 4.8 Hz, NOC<u>H</u>), 4.12–4.06 (2H, m, NC<u>H</u>$_2$), 1.85–1.75 (1H, m, OCHC<u>H</u>$_x$H$_y$), 1.66–1.56 (1H, m, OCHCH$_x$<u>H</u>$_y$), 1.56–1.25 (6H, m, (C<u>H</u>$_2$)$_3$), 1.19 (3H, s, C<u>H</u>$_3$), 1.14 (3H, s, C<u>H</u>$_3$), 1.10 (3H, s, C<u>H</u>$_3$), 1.07 (3H, s, C<u>H</u>$_3$), 0.90 (3H, t, *J* = 7.5 Hz, C<u>H</u>$_3$); **$^{13}$C{$^1$H} NMR** (126 MHz, $CDCl_3$) δ 164.9 (C), 153.3 (C), 152.5 (CH), 119.4 (CH), 84.5 (CH), 62.0 ($CH_2$), 60.1 (C), 59.4 (C), 42.6 ($CH_2$), 40.1 ($CH_2$), 34.6 ($CH_3$), 33.9 ($CH_3$), 26.7 ($CH_2$), 20.2 ($CH_3$), 20.2 ($CH_3$), 17.1 ($CH_2$), 9.3 ($CH_3$); **HRMS** (+ESI): *m/z* calcd. for $C_{18}H_{31}N_2O_4$ $[M+H]^+$: 339.2278, found: 339.2277.

### *N*-[(*E*)-5-Methyl-4-((2,2,6,6-tetramethylpiperidin-1-yl)oxy)-2-hexenoyl]-1,3-oxazolidin-2-one (17d)

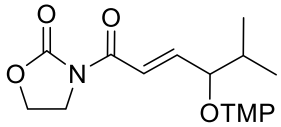

It was prepared following General Procedure 6 from **14d** (59.2 mg, 0.30 mmol), $Cu(OAc)_2$ (5.45 mg, 30 μmol, 10 mol%), 4,7-dimethyl-1,10-phenanthroline (9.37 mg, 45 μmol, 15 mol%), and TEMPO (93.8 mg, 0.60 mmol). Purification of the crude product by flash column chromatography (80:20 hexanes/EtOAc) afforded **17d** (100 mg, 0.28 mmol, 95% yield) as a brownish oil. **$R_f$** (80:20 hexanes/EtOAc) 0.2; **IR** (ATR) ν 2926, 2872, 2358, 1775, 1681, 1637, 1467, 1359, 1268, 1198, 1132, 1104, 1034 cm$^{-1}$; **$^1$H NMR** (500 MHz, $CDCl_3$) δ 7.26 (1H, d, *J* = 15.5 Hz, COC<u>H</u>=CH), 7.21 (1H, dd, *J* = 15.5, 8.0 Hz, COCH=C<u>H</u>) 4.47–4.40 (2H, m, OC<u>H</u>$_2$), 4.23 (1H, dd, *J* = 8.0, 5.3 Hz, NOC<u>H</u>), 4.14–4.07 (2H, m, NC<u>H</u>$_2$), 2.13–2.03 (1H, m, C<u>H</u>($CH_3$)$_2$), 1.63–1.23 (6H, m, 3 × C<u>H</u>$_2$), 1.19 (3H, s, C<u>H</u>$_3$), 1.16 (3H, s, C<u>H</u>$_3$), 1.10 (3H, s, C<u>H</u>$_3$), 1.08 (3H, s, C<u>H</u>$_3$), 0.95 (3H, d, *J* = 6.9 Hz, CHC<u>H</u>$_3$), 0.92 (3H, d, *J* = 6.9 Hz, CHC<u>H</u>$_3$); **$^{13}$C{$^1$H} NMR** (126 MHz, $CDCl_3$) δ 164.7 (C), 153.3 (C), 151.0 (CH), 120.9 (CH), 87.4 (CH), 61.9 ($CH_2$), 60.4 (C), 59.3 (C), 42.6 ($CH_2$), 40.2 ($CH_2$), 40.0 ($CH_2$), 34.6 ($CH_3$), 34.0 ($CH_3$), 32.0 (CH), 20.2 ($CH_3$), 20.2 ($CH_3$) 18.8 ($CH_3$), 17.6 ($CH_3$), 17.1 ($CH_2$); **HRMS** (+ESI): *m/z* calcd. for $C_{19}H_{33}N_2O_4$ [M+H]$^+$: 353.2435, found: 353.2433.

### *N*-[(*E*)-[5-Phenyl-4-((2,2,6,6-tetramethylpiperidin-1-yl)oxy)-2-pentenoyl]-1,3-oxazolidin-2-one (17e)

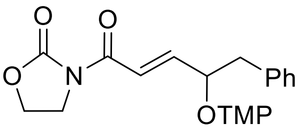

It was prepared following General Procedure 6 from **14e** (73.5 mg, 0.30 mmol), $Cu(OAc)_2$ (5.45 mg, 30 μmol, 10 mol%), 4,7-dimethyl-1,10-phenanthroline (9.37 mg, 45 μmol, 15 mol%), and TEMPO (93.8 mg, 0.60 mmol). Purification of the crude product by flash column chromatography (80:20 hexanes/EtOAc) afforded **17e** (106 mg, 0.26 mmol, 88% yield) as a brownish oil. **$R_f$** (80:20 hexanes/EtOAc) 0.2; **IR** (ATR) ν 3025, 2920, 2853, 1772, 1696, 1385, 1362, 1219, 1195, 1111, 1038, 1021, 969, 956, 740, 699 cm$^{-1}$; **$^1$H NMR** (500 MHz, $CDCl_3$) δ 7.28–7.24 (2H, m, Ar<u>H</u>), 7.20–7.16 (4H, m, Ar<u>H</u>, COC<u>H</u>=CH), 7.12 (1H, dd, *J* = 15.5, 7.7 Hz, COCH=C<u>H</u>), 4.63 (1H, td, *J* = 7.4, 6.0 Hz, NOC<u>H</u>), 4.42–4.37 (2H, m, OC<u>H</u>$_2$), 4.07–4.03 (2H, m, NC<u>H</u>$_2$), 3.17 (1H, dd, *J* = 13.4, 6.0 Hz, OCHC<u>H</u>$_x$H$_y$), 2.80 (1H, dd, *J* = 13.4, 7.4 Hz, OCHCH$_x$<u>H</u>$_y$), 1.44–1.36 (4H, m, 2 × C<u>H</u>$_2$), 1.32–1.22 (2H, m, C<u>H</u>$_2$), 1.12 (3H, s, C<u>H</u>$_3$), 1.10 (3H, s, C<u>H</u>$_3$), 1.05 (6H, s, 2 × C<u>H</u>$_3$); **$^{13}$C{$^1$H} NMR** (126 MHz, $CDCl_3$) δ 164.8 (C), 153.3 (C), 151.6 (CH), 137.5 (C), 129.8 (CH), 128.2 (CH), 126.2 (CH), 119.8 (CH), 83.8 (CH), 62.0 ($CH_2$), 60.4 (C), 59.6 (C), 42.6 ($CH_2$), 40.9 ($CH_2$), 40.2 ($CH_2$), 34.8 ($CH_3$), 34.0 ($CH_3$), 20.5 ($CH_3$), 20.3 ($CH_3$), 17.2 ($CH_2$); **HRMS** (+ESI): *m/z* calcd. for $C_{23}H_{33}N_2O_4$ [M+H]$^+$: 401.2435, found: 401.2426.

**_N_-[(_E_)-5,9-Dimethyl-4-((2,2,6,6-tetramethylpiperidin-1-yl)oxy)-2,8-decadienoyl]-1,3-oxazolidin-2-one (17f)**

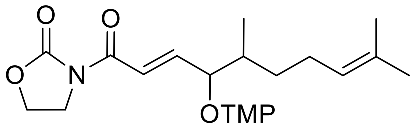


It was prepared following General Procedure 6 from **14f** (79.6 mg, 0.30 mmol), $Cu(OAc)_2$ (5.45 mg, 30 µmol, 10 mol%), 4,7-dimethyl-1,10-phenanthroline (9.37 mg, 45 µmol, 15 mol%), and TEMPO (93.8 mg, 0.60 mmol). Purification of the crude product by flash column chromatography (80:20 hexanes/EtOAc) afforded the mixture of isomers **17f** (115 mg, 0.27 mmol, 91% yield, dr 56:44) as a brownish oil. **$R_f$** (80:20 hexanes/EtOAc) 0.2; **IR** (ATR) ν 2970, 2927, 2359, 2339, 1781, 1683, 1637, 1467, 1359, 1346, 1333, 1275, 1226, 1196, 1131, 1110, 1087, 1039 $cm^{-1}$; **$^1$H NMR** (400 MHz, $CDCl_3$) δ 7.33–7.15 (2H, m, C<u>H</u>=C<u>H</u>), 5.16–5.05 (1H, m, C<u>H</u>=C), 4.46–4.40 (2H, m, OC<u>H</u>$_2$), 4.34–4.25 (1H, m, NOC<u>H</u>), 4.13–4.07 (2H, m, NC<u>H</u>$_2$), 2.11–1.88 (3H, m, C<u>H</u>$CH_3$, C<u>H</u>$_2$CH=C), 1.69 (3H, s, CC<u>H</u>$_3$), 1.61 (3H, s, CC<u>H</u>$_3$), 1.59–1.24 (8H, m, $CH_3$CHC<u>H</u>$_2$, 3 × C<u>H</u>$_2$), 1.18 (3H, s, C<u>H</u>$_3$), 1.15 (3H, s, C<u>H</u>$_3$), 1.10 (3H, s, C<u>H</u>$_3$), 1.07 (3H, s, C<u>H</u>$_3$), 0.95–0.91 (3H, m, CHC<u>H</u>$_3$); **$^{13}$C{$^1$H} NMR** (101 MHz, $CDCl_3$) δ 164.7 (C), 153.3 (C), 153.3 (C), 151.4 (CH), 150.7 (CH), 131.4 (C), 131.3 (C), 124.6 (CH), 124.4 (CH), 120.8 (CH), 120.5 (CH), 87.1 (CH), 86.6 (CH), 62.0 ($CH_2$), 60.5 (C), 60.3 (C), 59.5 (C), 42.6 ($CH_2$), 40.2 ($CH_2$), 37.2 (CH), 36.6 (CH), 34.6 ($CH_3$), 34.0 ($CH_3$), 33.3 ($CH_2$), 32.0 ($CH_2$), 25.8 ($CH_3$), 25.6 ($CH_3$), 25.6 ($CH_2$), 20.3 ($CH_3$), 17.6 ($CH_3$), 17.6 ($CH_3$), 17.1 ($CH_2$), 15.9 ($CH_3$), 14.6 ($CH_3$); **HRMS** (+ESI): *m/z* calcd. for $C_{24}H_{41}N_2O_4$ $[M+H]^+$: 421.3061, found: 421.3054.

**_N_-[(_E_)-4-((2,2,6,6-Tetramethylpiperidin-1-yl)oxy)-2-dodecen-11-ynoyl]-1,3-oxazolidin-2-one (17g)**

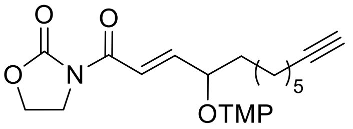


It was prepared following General Procedure 6 from **14g** (78.9 mg, 0.30 mmol), $Cu(OAc)_2$ (5.45 mg, 30 µmol, 10 mol%), 4,7-dimethyl-1,10-phenanthroline (9.37 mg, 45 µmol, 15 mol%), and TEMPO (93.8 mg, 0.60 mmol). Purification of the crude product by flash column chromatography (80:20 hexanes/EtOAc) afforded **17g** (109 mg, 0.26 mmol, 87% yield) as a brownish oil. **$R_f$** (80:20 hexanes/EtOAc) 0.2; **IR** (ATR) ν 3295, 2927, 2857, 2361, 1774, 1682, 1636, 1464, 1359, 1327, 1273, 1206, 1131, 1103, 1034 $cm^{-1}$; **$^1$H NMR** (400 MHz, $CDCl_3$) δ 7.27 (1H, d, *J* = 15.5 Hz, COC<u>H</u>=CH), 7.15 (1H, dd, *J* = 15.5, 8.1 Hz, COCH=C<u>H</u>), 4.46–4.40 (2H, m, OC<u>H</u>$_2$), 4.38 (1H, td, *J* = 8.1, 5.1 Hz, NOC<u>H</u>), 4.13–4.05 (2H, m, NC<u>H</u>$_2$), 2.18 (2H, td, *J* = 7.1, 2.6 Hz, C<u>H</u>$_2$C≡CH), 1.94 (1H, t, *J* = 2.6 Hz, C≡C<u>H</u>), 1.82–1.70 (1H, m, OCHC<u>H</u>$_x$$H_y$), 1.64–1.22 (15H, m, OCHCH$_x$<u>H</u>$_y$, 7 × C<u>H</u>$_2$), 1.16 (3H, s, C<u>H</u>$_3$), 1.13 (3H, s, C<u>H</u>$_3$), 1.08 (3H, s, C<u>H</u>$_3$), 1.06 (3H, s, C<u>H</u>$_3$); **$^{13}$C{$^1$H} NMR** (101 MHz, $CDCl_3$) δ 165.0 (C), 153.4 (C), 152.8 (CH), 119.4 (CH), 84.6 (C), 83.4 (CH), 68.1 (CH), 62.0 ($CH_2$), 60.2 (C), 59.4 (C), 42.7 ($CH_2$), 40.1 ($CH_2$), 34.7 ($CH_3$), 34.0 ($CH_3$), 33.9 ($CH_2$), 29.1 ($CH_2$), 28.6 ($CH_2$), 28.3 ($CH_2$), 24.8 ($CH_2$), 20.3 ($CH_3$), 20.3 ($CH_3$), 18.3 ($CH_2$), 17.2 ($CH_2$); **HRMS** (+ESI): *m/z* calcd. for $C_{24}H_{39}N_2O_4$ $[M+H]^+$: 419.2904, found: 419.2901.

***N*-[(*E*)-8-Chloro-4-((2,2,6,6-tetramethylpiperidin-1-yl)oxy)-2-octenoyl]-1,3-oxazolidin-2-one (17h)**

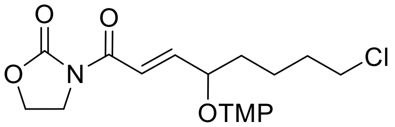


It was prepared following General Procedure 6 from **14h** (73.9 mg, 0.30 mmol), $Cu(OAc)_2$ (5.45 mg, 30 µmol, 10 mol%), 4,7-dimethyl-1,10-phenanthroline (9.37 mg, 45 µmol, 15 mol%), and TEMPO (93.8 mg, 0.60 mmol). Purification of the crude product by flash column chromatography (from 80:20 to 70:30 hexanes/EtOAc) afforded **17h** (111 mg, 0.28 mmol, 93% yield) as a brownish oil. **$R_f$** (80:20 hexanes/EtOAc) 0.2; **IR** (ATR) ν 2927, 2869, 2358, 1774, 1682, 1638, 1462, 1359, 1327, 1275, 1207, 1131, 1102, 1033 $cm^{-1}$; **$^1$H NMR** (500 MHz, $CDCl_3$) δ 7.28 (1H, d, *J* = 19.3 Hz, COCH=CH), 7.16 (1H, dd, *J* = 15.5, 8.1 Hz, COCH=CH), 4.47–4.37 (3H, m, $OCH_2$, NOCH), 4.13–4.06 (2H, m, $NCH_2$), 3.53 (2H, t, *J* = 6.7 Hz, $CH_2Cl$), 1.87–1.72 (3H, m, $OCHCH_xH_y$, $CH_2$), 1.67–1.58 (1H, m, $OCHCH_xH_y$), 1.58–1.37 (7H, m, $CH_xH_y$, 3 × $CH_2$), 1.35–1.24 (1H, m, $CH_xH_y$), 1.18 (3H, s, $CH_3$), 1.14 (3H, s, $CH_3$), 1.09 (3H, s, $CH_3$), 1.07 (3H, s, $CH_3$); **$^{13}$C{$^1$H} NMR** (126 MHz, $CDCl_3$) δ 164.7 (C), 153.3 (C), 152.1 (CH), 119.6 (CH), 83.0 (CH), 61.9 ($CH_2$), 60.1 (C), 59.3 (C), 44.6 ($CH_2$), 42.6 ($CH_2$), 40.0 ($CH_2$), 34.6 ($CH_3$), 33.9 ($CH_3$), 33.1 ($CH_2$), 32.5 ($CH_2$), 22.2 ($CH_2$), 20.2 ($CH_3$), 20.2 ($CH_3$), 17.0 ($CH_2$); **HRMS** (+ESI): *m/z* calcd. for $C_{20}H_{34}ClN_2O_4$ $[M+H]^+$: 401.2202, found: 401.2193.

***N*-[(*E*)-11-Oxo-4-((2,2,6,6-tetramethylpiperidin-1-yl)oxy)-2-dodecenoyl]-1,3-oxazolidin-2-one (17i)**

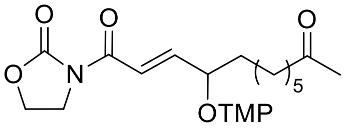


It was prepared following General Procedure 6 from **14i** (84.4 mg, 0.30 mmol), $Cu(OAc)_2$ (5.45 mg, 30 µmol, 10 mol%), 4,7-dimethyl-1,10-phenanthroline (9.37 mg, 45 µmol, 15 mol%), and TEMPO (93.8 mg, 0.60 mmol). Purification of the crude product by flash column chromatography (70:30 hexanes/EtOAc) afforded **17i** (116 mg, 0.27 mmol, 89% yield) as a yellow oil. **$R_f$** (70:30 hexanes/EtOAc) 0.2; **IR** (ATR) ν 2969, 2928, 2857, 1774, 1713, 1683, 1636, 1359, 1327, 1273, 1208, 1131, 1103, 1034, 758, 699 $cm^{-1}$; **$^1$H NMR** (400 MHz, $CDCl_3$) δ 7.25 (1H, d, *J* = 15.5 Hz, COCHCH), 7.14 (1H, dd, *J* = 15.5, 8.1 Hz, COCHCH), 4.48–4.39 (2H, m, $OCH_2$), 4.36 (1H, td, *J* = 8.1, 5.0 Hz, NOCH), 4.14–4.04 (2H, m, $NCH_2$), 2.40 (2H, t, *J* = 7.4 Hz, $COCH_2$), 2.12 (3H, s, $COCH_3$), 1.80–1.68 (1H, m, $OCHCH_xH_y$), 1.61–1.49 (3H, m, $COCH_2CH_2$, $OCHCH_xH_y$), 1.47–1.36 (4H, m, 2 × $CH_2$), 1.35–1.21 (8H, m, 4 × $CH_2$), 1.17 (3H, s, $CH_3$), 1.12 (3H, s, $CH_3$), 1.07 (3H, s, $CH_3$), 1.05 (3H, s, $CH_3$); **$^{13}$C{$^1$H} NMR** (101 MHz, $CDCl_3$) δ 209.0 (C), 164.7 (C), 153.1 (C), 152.5 (CH), 119.1 (CH), 83.2 (CH), 61.8 ($CH_2$), 60.0 (C), 59.1 (C), 43.4 ($CH_2$), 42.4 ($CH_2$), 39.8 ($CH_2$), 34.4 ($CH_3$), 33.7 ($CH_3$), 33.6 ($CH_2$), 29.5 ($CH_3$), 29.1 ($CH_2$), 28.7 ($CH_2$), 24.5 ($CH_2$), 23.4 ($CH_2$), 20.1 ($CH_3$), 20.0 ($CH_3$), 16.9 ($CH_2$); **HRMS** (+ESI): *m/z* calcd. for $C_{24}H_{41}N_2O_5$ $[M+H]^+$: 437.3010, found: 437.2998.

**$N$-[($E$)-(12-Methoxy-12-oxo-4-(2,2,6,6-tetramethylpiperidin-1-yl)oxy)-2-dodecenoyl]-1,3-oxazolidin-2-one (17j)**

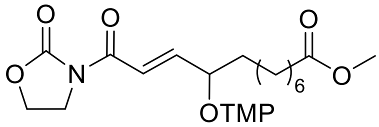

It was prepared following General Procedure 6 from **14j** (89.2 mg, 0.30 mmol), $Cu(OAc)_2$ (5.45 mg, 30 μmol, 10 mol%), 4,7-dimethyl-1,10-phenanthroline (9.37 mg, 45 μmol, 15 mol%), and TEMPO (93.8 mg, 0.60 mmol). Purification of the crude product by flash column chromatography (70:30 hexanes/EtOAc) afforded **17j** (125 mg, 0.27 mmol, 89% yield) as a brownish oil. $\mathbf{R_f}$ (70:30 hexanes/EtOAc) 0.2; **IR** (ATR) ν 2926, 2856, 1776, 1735, 1683, 1637, 1463, 1436, 1359, 1327, 1274, 1197, 1132, 1104, 1035 $cm^{-1}$; **$^1$H NMR** (500 MHz, $CDCl_3$) δ 7.26 (1H, d, $J$ = 15.5 Hz, COC<u>H</u>=CH), 7.15 (1H, dd, $J$ = 15.5, 8.1 Hz, COCH=C<u>H</u>), 4.46–4.40 (2H, m, OC<u>H</u>$_2$), 4.36 (1H, td, $J$ = 8.1, 5.0 Hz, NOC<u>H</u>), 4.12–4.06 (2H, m, NC<u>H</u>$_2$), 3.66 (s, 3H, OC<u>H</u>$_3$), 2.30 (2H, t, $J$ = 7.6 Hz, COC<u>H</u>$_2$), 1.80–1.69 (1H, m, OCHC<u>H</u>$_x$H$_y$), 1.66–1.49 (5H, m, OCHCH$_x$<u>H</u>$_y$, 2×C<u>H</u>$_2$), 1.48–1.36 (4H, m, 2 × C<u>H</u>$_2$), 1.35–1.22 (8H, m, 4 × C<u>H</u>$_2$), 1.17 (3H, s, C<u>H</u>$_3$), 1.12 (3H, s, C<u>H</u>$_3$), 1.08 (3H, s, C<u>H</u>$_3$), 1.05 (3H, s, C<u>H</u>$_3$); **$^{13}$C{$^1$H} NMR** (126 MHz, $CDCl_3$) δ 174.3 (C), 165.0 (C), 153.4 (C), 152.9 (CH), 119.4 (CH), 83.5 (CH), 62.0 ($CH_2$), 60.2 (C), 59.4 (C), 51.4 ($CH_3$), 42.7 ($CH_2$), 40.1 ($CH_2$), 34.7 ($CH_3$), 34.0 ($CH_2$), 34.0 ($CH_3$), 33.9 ($CH_2$), 29.5 ($CH_2$), 29.1 ($CH_2$), 29.0 ($CH_2$), 24.9 ($CH_2$), 24.9 ($CH_2$), 20.3 ($CH_3$), 20.3 ($CH_3$), 17.2 ($CH_2$); **HRMS** (+ESI): $m/z$ calcd. for $C_{25}H_{43}N_2O_6$ $[M+H]^+$: 467.3116, found: 467.3112.

**$N$-[($E$)-2-(2-((2,2,6,6-Tetramethylpiperidin-1-yl)oxy)cyclohexylidene)acetyl]-1,3-oxazolidin-2-one (17l)**

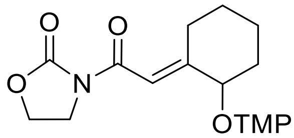

It was prepared following General Procedure 6 performed at 70 °C for 48 h, from **14l** (62.8 mg, 0.30 mmol), $Cu(OAc)_2$ (5.45 mg, 30 μmol, 10 mol%), 4,7-dimethyl-1,10-phenanthroline (9.37 mg, 45 μmol, 15 mol%), and TEMPO (141 mg, 0.90 mmol). Purification of the crude product by flash column chromatography (80:20 hexanes/EtOAc) afforded **17l** (65 mg, 0.18 mmol, 59% yield, dr 78:22) as a yellow oil. $\mathbf{R_f}$ (60:40 hexanes/EtOAc) 0.4; **IR** (ATR) ν 2926, 2857, 1774, 1675, 1627, 1381, 1359, 1275, 1187, 1038, 956, 758, 699 $cm^{-1}$; *Major*: **$^1$H NMR** (400 MHz, $CDCl_3$) δ 7.13 (1H, s, C<u>H</u>=C), 4.43–4.35 (2H, m, OC<u>H</u>$_2$), 4.25–4.17 (1H, m, NOC<u>H</u>), 4.10–4.00 (2H, m, NC<u>H</u>$_2$), 3.19–3.05 (1H, m, CH=CC<u>H</u>$_x$H$_y$), 2.60–2.49 (1H, m, CH=CCH$_x$<u>H</u>$_y$), 2.16–2.03 (1H, m, NOCHC<u>H</u>$_x$H$_y$), 1.89–1.73 (2H, m, NOCHCH$_x$<u>H</u>$_y$, OCHCH$_2$C<u>H</u>$_x$H$_y$), 1.71–1.20 (9H, m, OCHCH$_2$CH$_x$<u>H</u>$_y$, 4 × C<u>H</u>$_2$), 1.17 (6H, s, 2 × C<u>H</u>$_3$), 1.15 (3H, s, C<u>H</u>$_3$), 1.06 (3H, s, C<u>H</u>$_3$); *Major*: **$^{13}$C{$^1$H} NMR** (101 MHz, $CDCl_3$) δ 165.7 (C), 165.1 (C), 153.3 (C), 112.0 (CH), 85.7 (CH), 61.8 ($CH_2$), 60.2 (C), 59.6 (C), 42.7 ($CH_2$), 40.4 ($CH_2$), 35.6 ($CH_2$), 34.3 ($CH_3$), 34.0 ($CH_3$), 29.4 ($CH_2$), 28.4 ($CH_2$), 23.4 ($CH_2$), 20.5 ($CH_3$) 17.2 ($CH_2$); **HRMS** (+ESI): $m/z$ calcd. for $C_{20}H_{33}N_2O_4$ $[M+H]^+$: 365.2435, found: 365.2425.

***N*-[(*E*)-2-Methyl-4-((2,2,6,6-tetramethylpiperidin-1-yl)oxy)-2-butenoyl]-1,3-oxazolidin-2-one (17m)**

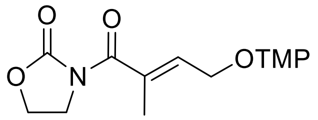


It was prepared following General Procedure 6 performed at 70 °C for 72 h, from **14m** (50.8 mg, 0.30 mmol), Cu(OAc)$_2$ (5.45 mg, 30 μmol, 10 mol%), 4,7-dimethyl-1,10-phenanthroline (9.37 mg, 45 μmol, 15 mol%), and TEMPO (141 mg, 0.90 mmol). Purification of the crude product by flash column chromatography (70:30 hexanes/EtOAc) afforded **17m** (85 mg, 0.26 mmol, 87% yield, dr 92:8) as a white solid. **Mp** 91–94 °C; **R$_f$** (80:20 hexanes/EtOAc) 0.2; **IR** (ATR) ν 3006, 2973, 2924, 2868, 1767, 1679, 1377, 1359, 1332, 1284, 1196, 1105, 1034, 760, 710, 680 cm$^{-1}$; *Major*: **$^1$H NMR** (400 MHz, CDCl$_3$) δ 6.13 (1H, tq, *J* = 5.8, 1.3 Hz, C=CH), 4.48 (2H, dq, *J* = 5.8, 1.3 Hz, NOCH$_2$), 4.46–4.39 (2H, m, OCH$_2$), 4.04–3.98 (2H, m, NCH$_2$), 1.88 (3H, d, *J* = 1.3 Hz, CH$_3$C=CH), 1.63–1.49 (1H, m, CH$_x$H$_y$), 1.49–1.38 (4H, m, 2 × CH$_2$), 1.35–1.27 (1H, m, CH$_x$H$_y$), 1.16 (6H, s, 2 × CH$_3$), 1.08 (6H, s, 2 × CH$_3$); *Major*: **$^{13}$C{$^1$H} NMR** (101 MHz, CDCl$_3$) δ 171.5 (C), 153.1 (C), 134.8 (CH), 130.7 (C), 74.4 (CH$_2$), 62.4 (CH$_2$), 59.9 (C), 43.4 (CH$_2$), 39.7 (CH$_2$), 33.1 (CH$_3$), 20.2 (CH$_3$), 17.2 (CH$_2$), 14.4 (CH$_3$); **HRMS** (+ESI): *m/z* calcd. for $C_{17}H_{29}N_2O_4$ [M+H]$^+$: 325.2122, found: 325.2115.

***N*-[(E)-4-((2,2,6,6-Tetramethylpiperidin-1-yl)oxy)-2,4-pentadienoyl]-1,3-oxazolidin-2-one (17n)**

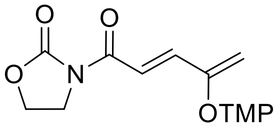


It was prepared following General Procedure 6 using a 62:38 mixture of **14n** and its α,β-γ,δ-diunsaturated isomer (26.9 mg, 0.10 mmol of **14n**), Cu(OAc)$_2$ (1.82 mg, 10 μmol, 10 mol%), 4,7-dimethyl-1,10-phenanthroline (3.12 mg, 15 μmol, 15 mol%), and TEMPO (31.3 mg, 0.20 mmol). Purification of the crude product by flash column chromatography (from 80:20 to 60:40 hexanes/EtOAc) afforded **17n** (16 mg, 50 μmol, 50% yield) as a white solid. **Mp** 99–103 °;C **R$_f$** (80:20 hexanes/EtOAc) 0.2; **IR** (ATR) ν 3110, 2974, 2928, 2868, 2853, 1761, 1687, 1621, 1593, 1482, 1456, 1387, 1360, 1329, 1308, 1262, 1206, 1131, 1109, 1036, 980, 964, 837, 760, 729, 703 cm$^{-1}$; **$^1$H NMR** (400 MHz, CDCl$_3$) δ 7.69 (1H, d, *J* = 15.5 Hz, COCH=CH), 7.16 (1H, d, *J* = 15.5 Hz, COCH=CH), 5.30 (1H, s, C=CH$_x$H$_y$), 4.61 (1H, s, C=CH$_x$H$_y$), 4.47–4.38 (2H, m, OCH$_2$), 4.14–4.04 (2H, m, NCH$_2$), 1.71–1.58 (1H, m, CH$_x$H$_y$), 1.56–1.49 (4H, m, 2 × CH$_2$), 1.43–1.24 (1H, m, CH$_x$H$_y$), 1.22 (6H, s, 2 × CH$_3$), 1.02 (6H, s, 2 × CH$_3$); **$^{13}$C{$^1$H} NMR** (101 MHz, CDCl$_3$) δ 165.7 (C), 159.6 (C), 153.6 (C), 140.6 (CH), 116.2 (CH), 99.8 (CH$_2$), 62.1 (CH$_2$), 60.8 (C), 42.9 (CH$_2$), 39.8 (CH$_2$), 32.4 (CH$_3$), 20.7 (CH$_3$), 17.1 (CH$_2$); **HRMS** (+ESI): *m/z* calcd. for $C_{17}H_{26}N_2NaO_4$ [M+Na]$^+$: 345.1785, found: 345.1783.

**N-[(2E,4E)-6-((2,2,6,6-Tetramethylpiperidin-1-yl)oxy)-2,4-hexadienoyl]-1,3-oxazolidin-2-one (17o)**

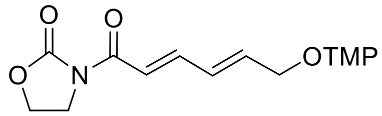


It was prepared following General Procedure 6 from **14o** (54.4 mg, 0.30 mmol), $Cu(OAc)_2$ (5.45 mg, 30 μmol, 10 mol%), 4,7-dimethyl-1,10-phenanthroline (9.37 mg, 45 μmol, 15 mol%), and TEMPO (93.8 mg, 0.60 mmol). Purification of the crude product by flash column chromatography (80:20 hexanes/EtOAc) afforded **17o** (85 mg, 0.25 mmol, 84% yield) as a white solid. **Mp** 113–116 °C; **$R_f$** (80:20 hexanes/EtOAc) 0.2; **IR** (ATR) ν 3086, 3004, 2965, 2924, 2868, 1765, 1681, 1634, 1595, 1478, 1387, 1340, 1264, 1224, 1161, 1131, 1101, 1034, 1006, 971, 954, 788, 758, 706 $cm^{-1}$; **$^1H$ NMR** (400 MHz, $CDCl_3$) δ 7.48 (1H, dd, $J$ = 15.1, 11.1 Hz, COCH=C<u>H</u>CH=CH), 7.30 (1H, d, $J$ = 15.1 Hz, COC<u>H</u>=CHCH=CH), 6.52 (1H, ddt, $J$ = 15.4, 11.1, 1.8 Hz, COCH=CHC<u>H</u>=CH), 6.21 (1H, dt, $J$ = 15.4, 5.1 Hz, COCH=CHCH=C<u>H</u>), 4.48–4.38 (4H, m, NOC$\underline{H}_2$, OC$\underline{H}_2$), 4.13–4.04 (2H, m, NC$\underline{H}_2$), 1.67–1.25 (6H, m, 3 × C$\underline{H}_2$), 1.15 (6H, s, 2 × C$\underline{H}_3$), 1.12 (6H, s, 2 × C$\underline{H}_3$); **$^{13}C\{^1H\}$ NMR** (101 MHz, $CDCl_3$) δ 165.6 (C), 153.6 (C), 146.3 (CH), 140.4 (CH), 128.6 (CH), 119.3 (CH), 76.8 ($CH_2$), 62.1 ($CH_2$), 60.0 (C), 42.9 ($CH_2$), 39.7 ($CH_2$), 33.0 ($CH_3$), 20.3 ($CH_3$), 17.2 ($CH_2$); **HRMS** (+ESI): $m/z$ calcd. for $C_{18}H_{29}N_2O_4$ $[M+H]^+$: 337.2122, found: 337.2124.

## 11. Removal of the Heterocycle from 17b

### Mixture of (*E*)-4-((2,2,6,6-tetramethylpiperidin-1-yl)oxy)-2-penten-1-ol (18b) and 4-((2,2,6,6-tetramethylpiperidin-1-yl)oxy)-1-pentanol (19b)

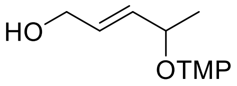


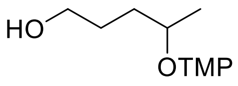


**18b** **19b**

A solution of the γ-aminoxylated compound **17b** (97.3 mg, 0.30 mmol) in $Et_2O$ (6 mL) was cooled to 0 °C under a nitrogen atmosphere. Water (11 μL, 0.60 mmol) was added, followed by a solution of $LiBH_4$ (2 M in THF, 0.3 mL, 0.60 mmol) and the reaction mixture was stirred at 0 °C for 1 h. The mixture was quenched with 1 M NaOH (10 mL) and extracted with EtOAc (3 × 10 mL). The combined organic extracts were washed with brine (30 mL), dried with anhydous $MgSO_4$, filtered, and concentrated *in vacuo*. Purification of the crude product by flash column chromatography (from 80:20 to 70:30 hexanes/EtOAc) afforded a 55:45 mixture of **18b** and **19b** respectively (59 mg, 0.244 mmol, 81% yield), as a yellowish oil. **$R_f$** (70:30 hexanes/EtOAc) 0.3; **IR** (ATR) ν 3330, 2969, 2926, 2870, 1457, 1373, 1359, 1258, 1241, 1131, 1060, 1042, 1010, 956, 712 $cm^{-1}$; **18b**: **$^1$H NMR** (500 MHz, $CDCl_3$) δ 5.79–5.67 (2H, m, CH=CH), 4.33–4.24 (1H, m, NOCH), 4.14 (2H, d, *J* = 4.2 Hz, $CH_2OH$), 1.82 (1H, br s, OH), 1.61–1.25 (6H, m, 3 × $CH_2$), 1.24 (3H, d, *J* = 6.5 Hz, $CHCH_3$), 1.16 (3H, s, $CH_3$), 1.12 (6H, s, 2 × $CH_3$), 1.09 (3H, s, $CH_3$); **19b**: **$^1$H NMR** (500 MHz, $CDCl_3$) δ 3.98–3.90 (1H, m, NOCH), 3.66 (2H, t, *J* = 6.2 Hz, $CH_2OH$), 1.82 (1H, br s, OH), 1.69–1.63 (2H, m, $CH_2CH_2OH$), 1.58–1.25 (8H, m, $NOCHCH_2$, 3 × $CH_2$), 1.18 (3H, d, *J* = 6.3 Hz, $CHCH_3$), 1.14 (3H, s, $CH_3$), 1.09 (9H, br s, 3 × $CH_3$); **18b**: **$^{13}C\{^1H\}$ NMR** (126 MHz, $CDCl_3$) δ 135.8 (CH), 128.6 (CH), 80.1 (CH), 63.5 ($CH_2$), 59.9 (C), 59.6 (C), 40.3 ($CH_2$), 35.1 ($CH_3$), 34.4 ($CH_3$), 20.9 ($CH_3$), 20.6 ($CH_3$), 20.4 ($CH_3$), 17.4 ($CH_2$); **19b**: **$^{13}$C NMR**(126 MHz, $CDCl_3$) δ 78.3 (CH), 63.4 ($CH_2$), 60.3 (C), 59.3 (C), 40.4 ($CH_2$), 35.1 ($CH_3$), 34.4 ($CH_3$), 32.7 ($CH_2$), 29.1 ($CH_2$), 20.6 ($CH_3$), 20.4 ($CH_3$), 19.9 ($CH_3$), 17.4 ($CH_2$); **18b**: **HRMS** (+ESI): *m/z* calcd. for $C_{14}H_{28}NO_2$ $[M+H]^+$: 242.2115, found: 242.2115; **19b**: **HRMS** (+ESI): *m/z* calcd. for $C_{14}H_{30}NO_2$ $[M+H]^+$: 244.2271, found: 244.2271.

### Methyl (*E*)-4-((2,2,6,6-tetramethylpiperidin-1-yl)oxy)-2-pentenoate (20b)

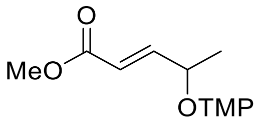


On the benchtop, $Yb(OTf)_3$ (9.3 mg, 15 μmol, 5 mol%) was weighted in a 5 mL microwave vial, followed by the addition of anhydrous methanol (1.5 mL). The vial was sealed with a pressure-relief cap and heated in an oil bath at 60 °C for 30 min. Then, a solution of **17b** (97.3 mg, 0.30 mmol) in anhydrous methanol (1.5 mL) was transferred via cannula. The mixture was then stirred at 60 °C for 2 h and the solvent was carefully evaporated under reduced pressure. The crude product was purified by flash column chromatography (from 95:5 to 80:20 hexanes/EtOAc) to afford **20b** (77 mg, 0.29 mmol, 95% yield) as a yellow oil. **$R_f$** (80:20 hexanes/EtOAc) 0.7; **IR** (ATR) ν 2973, 2930, 2872, 2848, 1726, 1657, 1435, 1360, 1256, 1172, 1131, 1042, 949, 863, 706 $cm^{-1}$; **$^1$H NMR** (400 MHz, $CDCl_3$) δ 6.98 (1H, dd, *J* = 15.8, 6.9 Hz, COCH=CH), 5.91 (1H, dd, *J* = 15.8, 1.2 Hz, COCH=CH), 4.44 (1H, dqd, *J* = 6.9, 6.7, 1.2 Hz, NOCH), 3.74 (3H, s,

OC$\underline{H}_3$), 1.67–1.31 (6H, m, 3 × C$\underline{H}_2$), 1.28 (3H, d, $J$ = 6.7 Hz, CHC$\underline{H}_3$), 1.17 (3H, s, C$\underline{H}_3$), 1.10 (6H, s, 2 × C$\underline{H}_3$), 1.07 (3H, s, C$\underline{H}_3$); **$^{13}$C{$^1$H} NMR** (101 MHz, $CDCl_3$) δ 167.1 (C), 151.5 (CH), 119.4 (CH), 79.2 (CH), 59.8 (C), 51.5 ($CH_3$), 40.2 ($CH_2$), 34.5 ($CH_3$), 34.2 ($CH_3$), 20.4 ($CH_3$), 20.2 ($CH_3$), 17.2 ($CH_2$); **HRMS** (+ESI): *m/z* calcd. for $C_{15}H_{28}NO_3$ [M+H]$^+$: 270.2064, found: 270.2065.

## 12. References


(1) Spectroscopic data are in accordance with data reported in the literature: Ren, J.; Li, Y.; Ke, H.; Li, Y.; Yang, L.; Yu, H.; Huang, R.; Lu, C.; Qiu, Y. Design, synthesis, and biological evaluation of oxazolidone derivatives as highly potent *N*-acylethanolamine acid amidase (NAAA) inhibitors. *RSC Adv.* **2017**, *7*, 12455–12463.

(2) Spectroscopic data are in accordance with data reported in the literature: Evans, D. A.; Nelson, S. G. Chiral Magnesium Bis(sulfonamide) Complexes as Catalysts for the Merged Enolization and Enantioselective Amination of *N*-Acyloxazolidinones. A Catalytic Approach to the Synthesis of Arylglycines. *J. Am. Chem. Soc.* **1997**, *119*, 6452–6453.

(3) Spectroscopic data are in accordance with data reported in the literature: Jin, X.; Yamaguchi, K.; Mizuno, N. A Green Synthetic Route to Imides from Terminal Alkynes and Amides from Simple Solid Catalysts. *Chem. Lett.* **2012**, *41*, 866–867.

(4) Spectroscopic data are in accordance with data reported in the literature: Lanigan, R. M.; Starkov, P.; Sheppard, T. D. Direct Synthesis of Amides from Carboxylic Acids and Amines Using $B(OCH_2CF_3)_3$, *J. Org. Chem.* **2013**, *78*, 4512–4523.

(5) Spectroscopic data are in accordance with data reported in the literature: Evans, D. A.; Thomson, R. J. Ni(II) Tol-BINAP-Catalyzed Enantioselective Orthoester Alkylations of *N*-Acylthiazolidinethiones. *J. Am. Chem. Soc.* **2005**, *127*, 10506–10507.

(6) Feuillet, F. J. P.; Cheeseman, M.; Mahon, M. F.; Bull, S. D. Stereoselective synthesis of (*E*)-trisubstituted α,β-unsaturated amides and acids. *Org. Biomol. Chem.* **2005**, *3*, 2976–2989.

(7) Wang, G.; Liang, X.; Chen, L.; Gao, Q.; Wang, J.-G.; Zhang, P.; Peng, Q.; Xu, S. Iridium-Catalyzed Distal Hydroboration of Aliphatic Internal Alkenes. *Angew. Chem. Int. Ed.* **2019**, *58*, 8187–8191.

(8) (a) Desrat, S.; Gray, P. J.; Penny, M. R.; Motherwell, W. B. Taming the Carboxyl Group for Directed Carbometalation: Observations on the Use of Anions, Dianions and Ester Enolates. *Chem. Eur. J.* **2014**, *20*, 8918–8922. (b) Heindl, S.; Riomet, M.; Matyasovsky, J.; Lemmerer, M.; Malzer, N.; Maulide, N. Chemoselective γ-Oxidation of β,γ-Unsaturated Amides with TEMPO. *Angew. Chem. Int. Ed.* **2021**, *60*, 19123–19127.

# 13. NMR Spectra of Synthesized Compounds

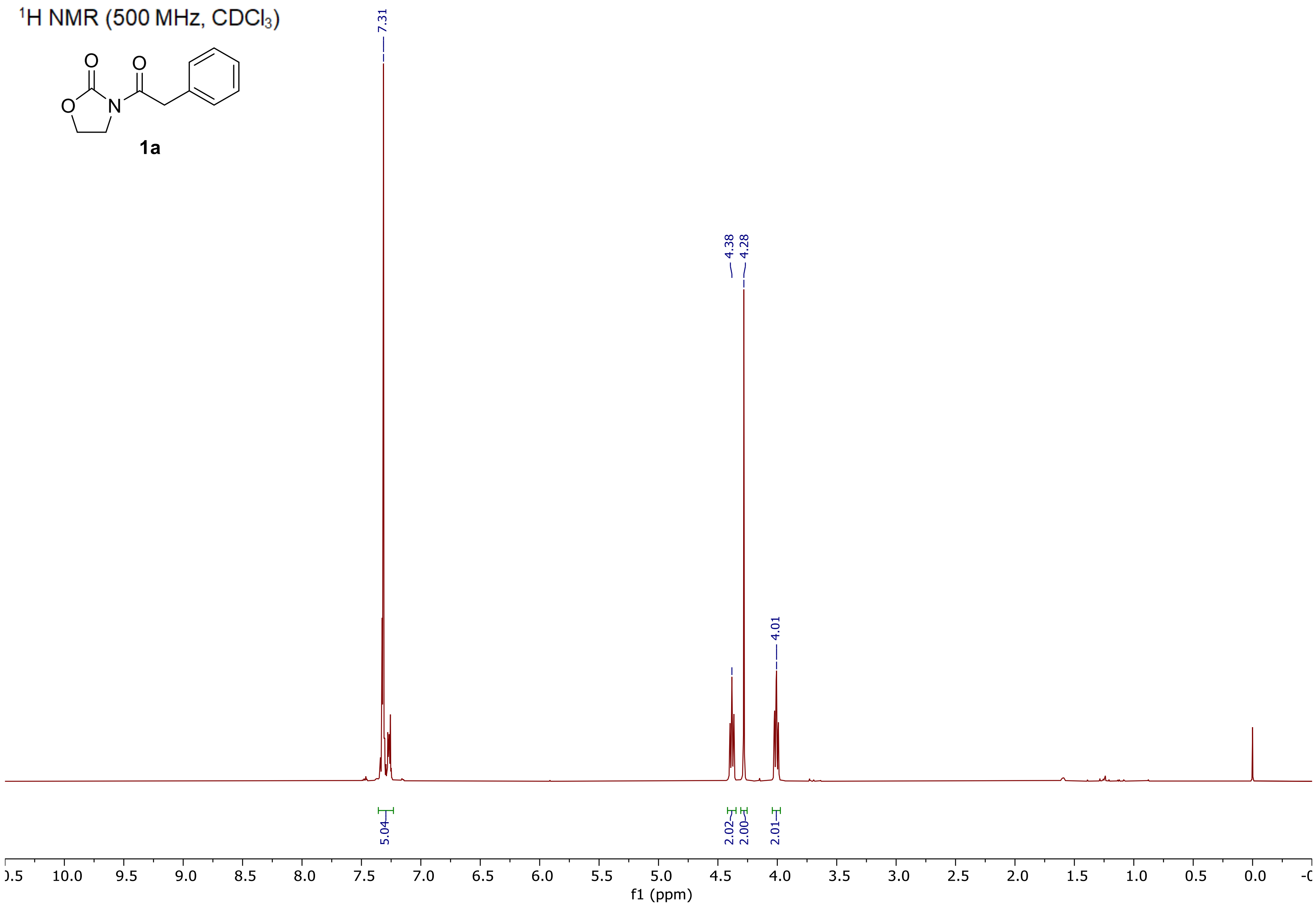
1H NMR (500 MHz, CDCl3)
1a
7.31
4.38
4.28
4.01
5.04
2.02
2.00
2.01
f1 (ppm)

$^{13}C\{^1H\}$ NMR (126 MHz, $CDCl_3$)

**1a**

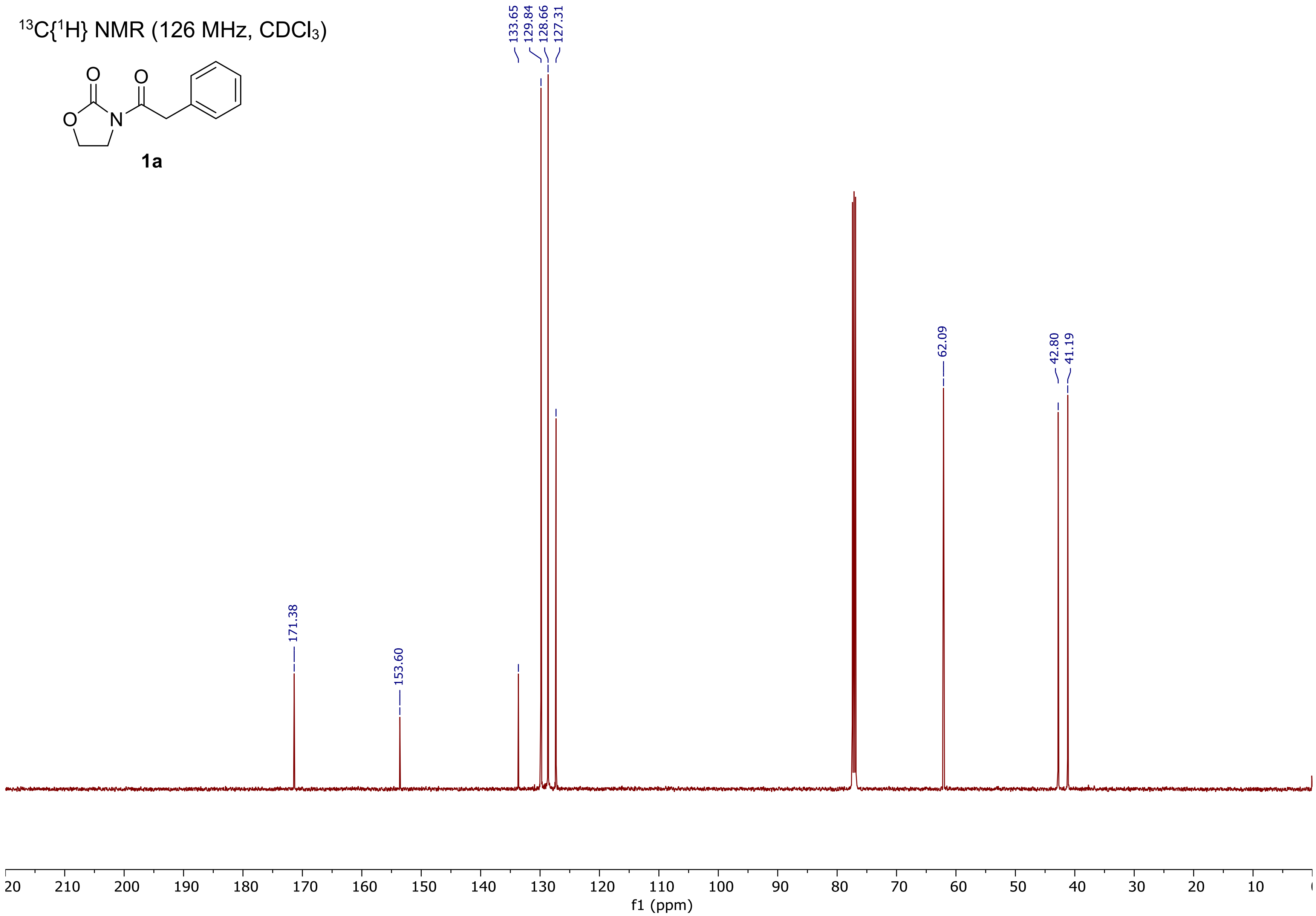

2D $^{1}H$ -$^{1}H$ COSY (500 MHz, $CDCl_3$)

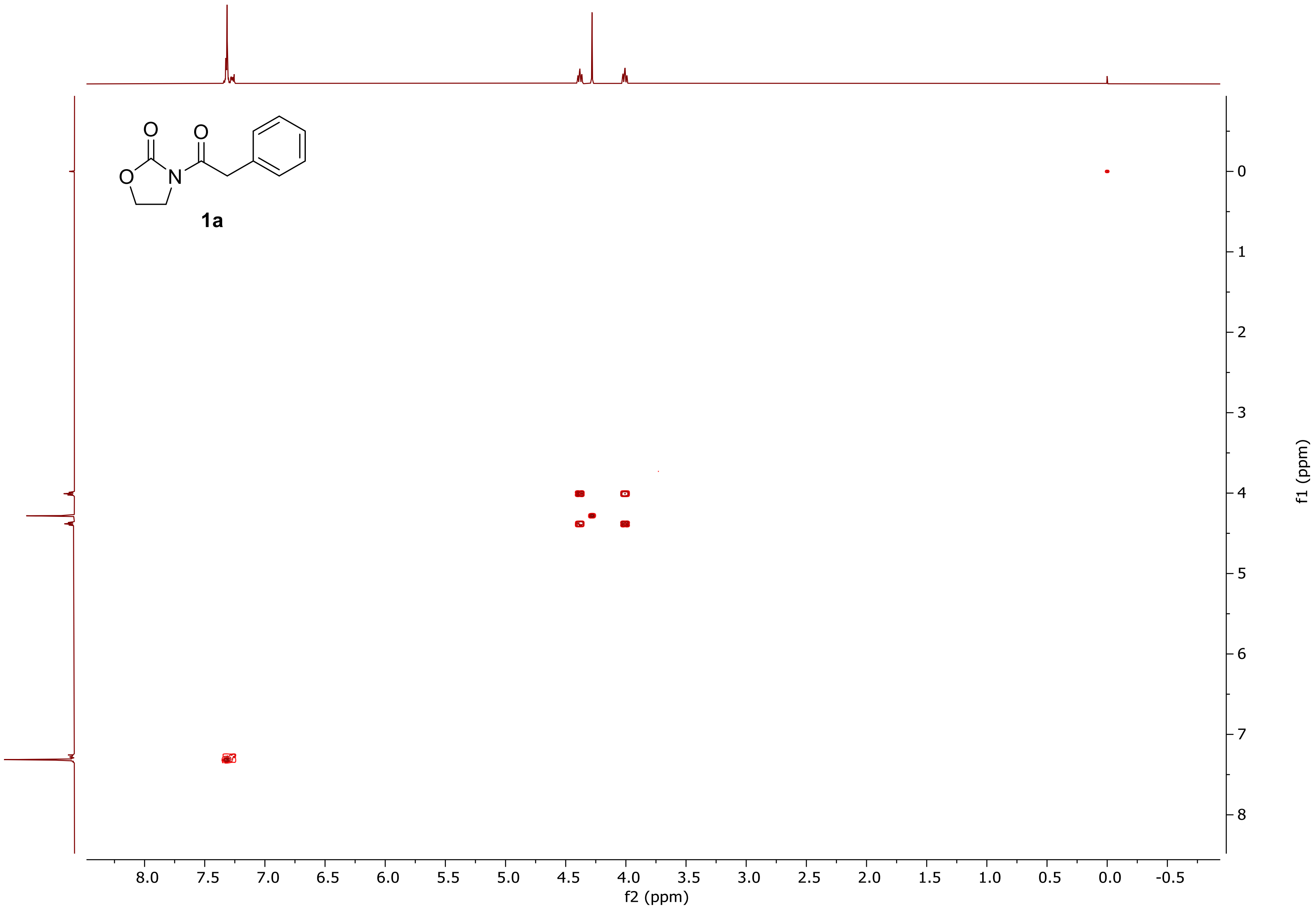

2D $^{1}H$ -$^{13}C$ HSQC (500 MHz, $CDCl_3$)

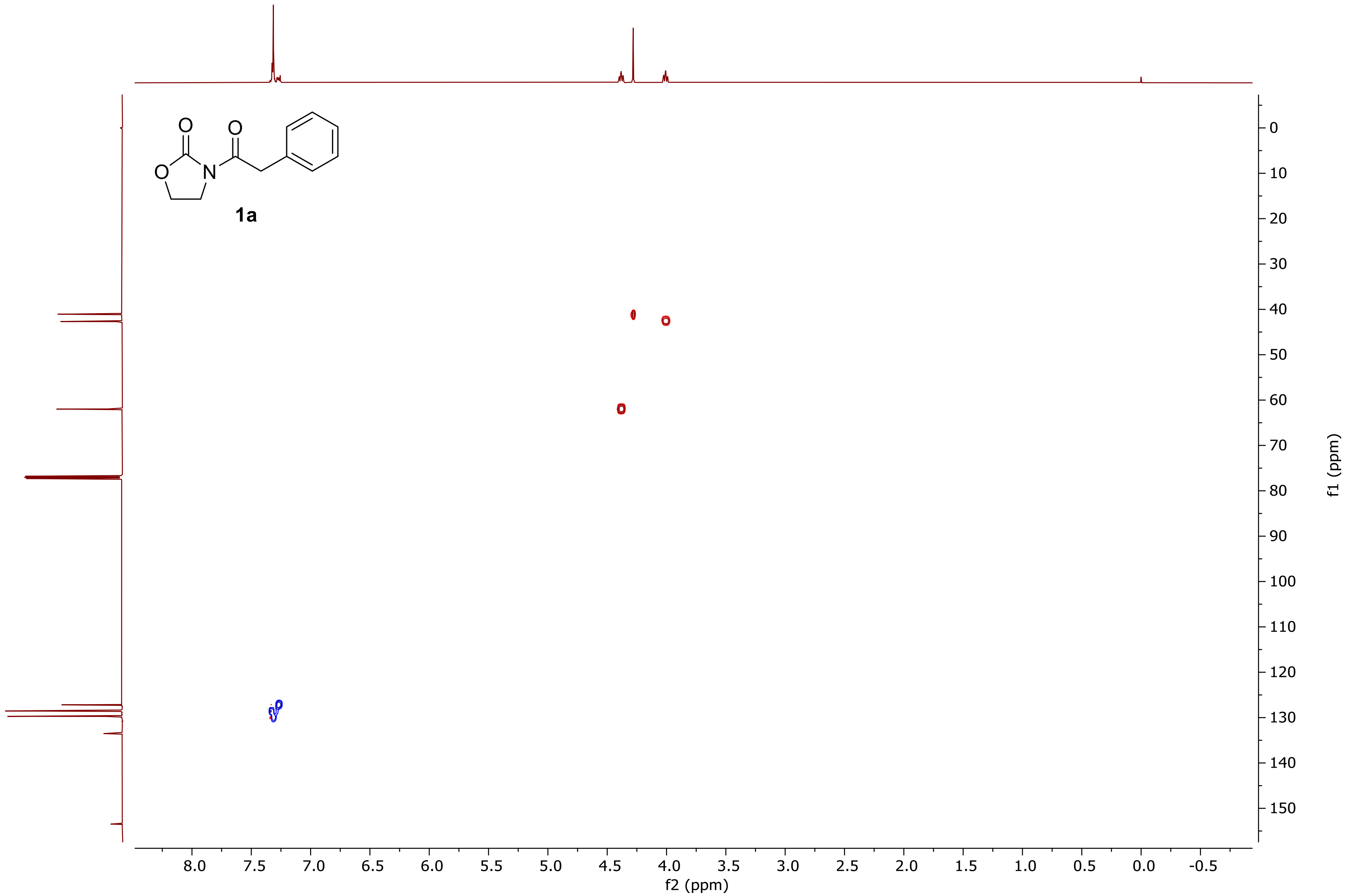

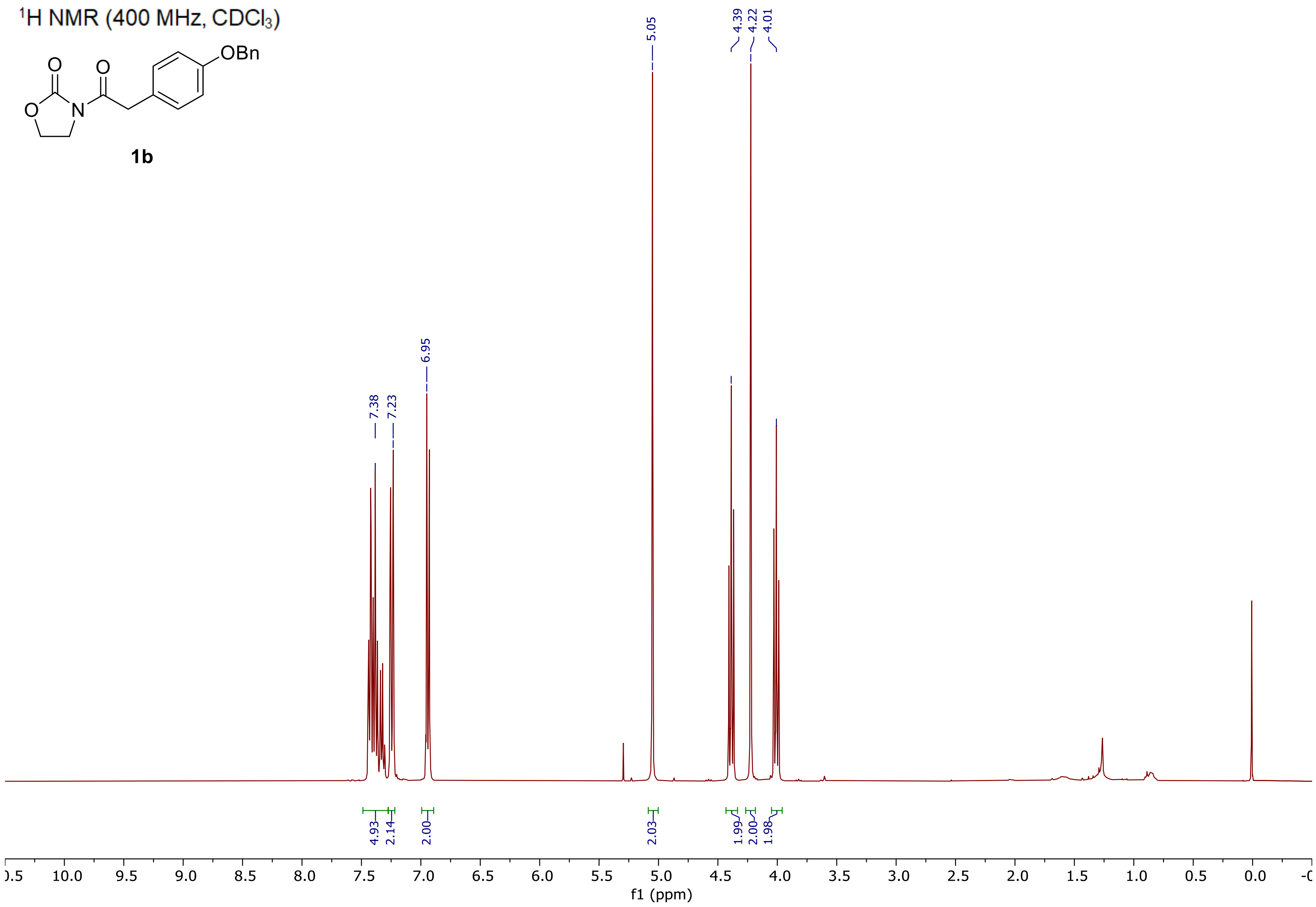
1H NMR (400 MHz, CDCl3)
OBn
O
O
O
N
1b
5.05
4.39
4.22
4.01
6.95
7.38
7.23
4.93
2.14
2.00
2.03
1.99
2.00
1.98
f1 (ppm)

$^{13}C\{^{1}H\}$ NMR (101 MHz, $CDCl_3$)

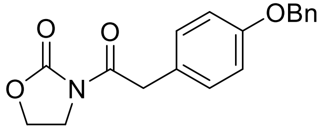


**1b**

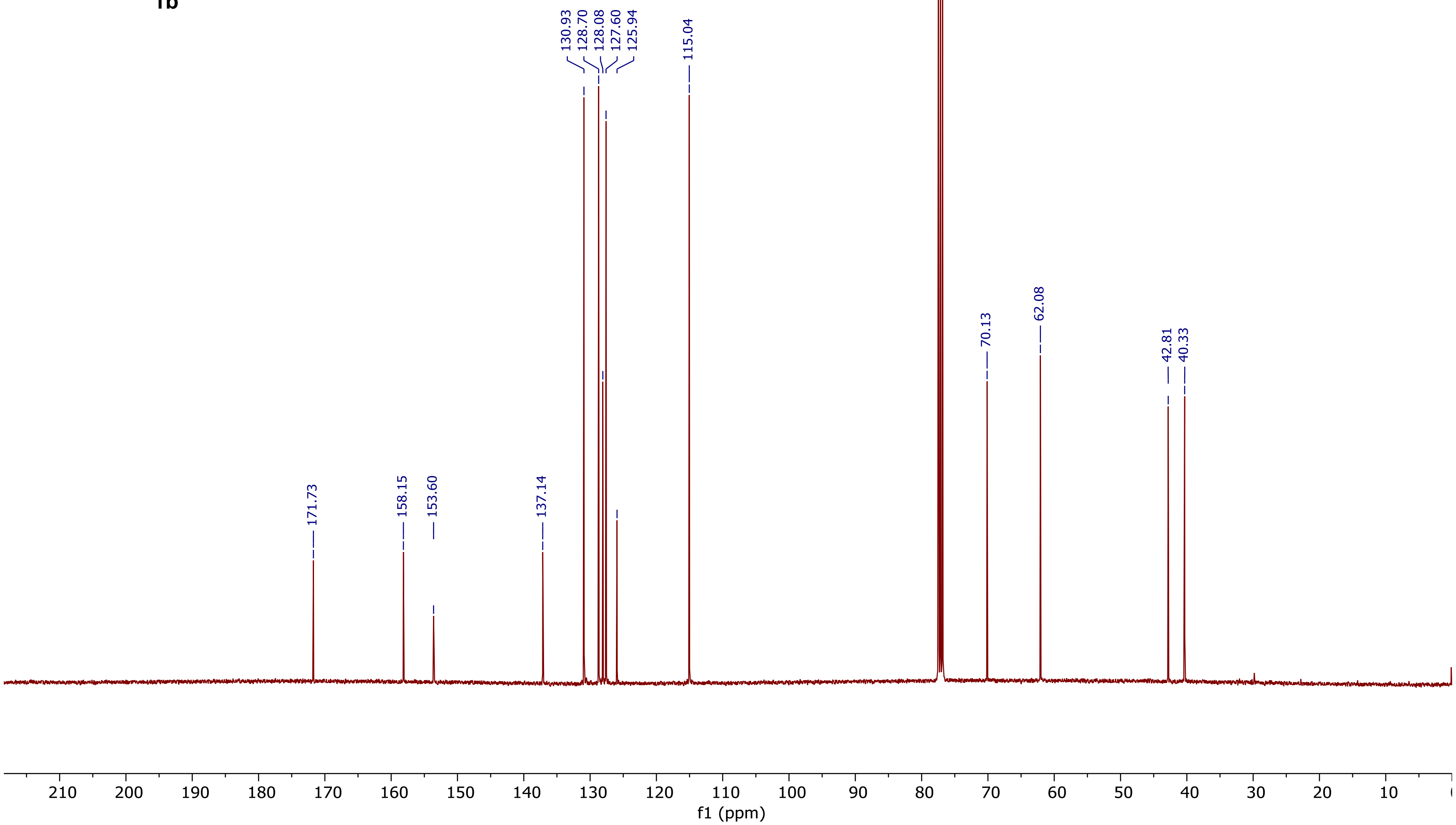

2D $^{1}H$ -$^{1}H$ COSY (400 MHz, $CDCl_3$)

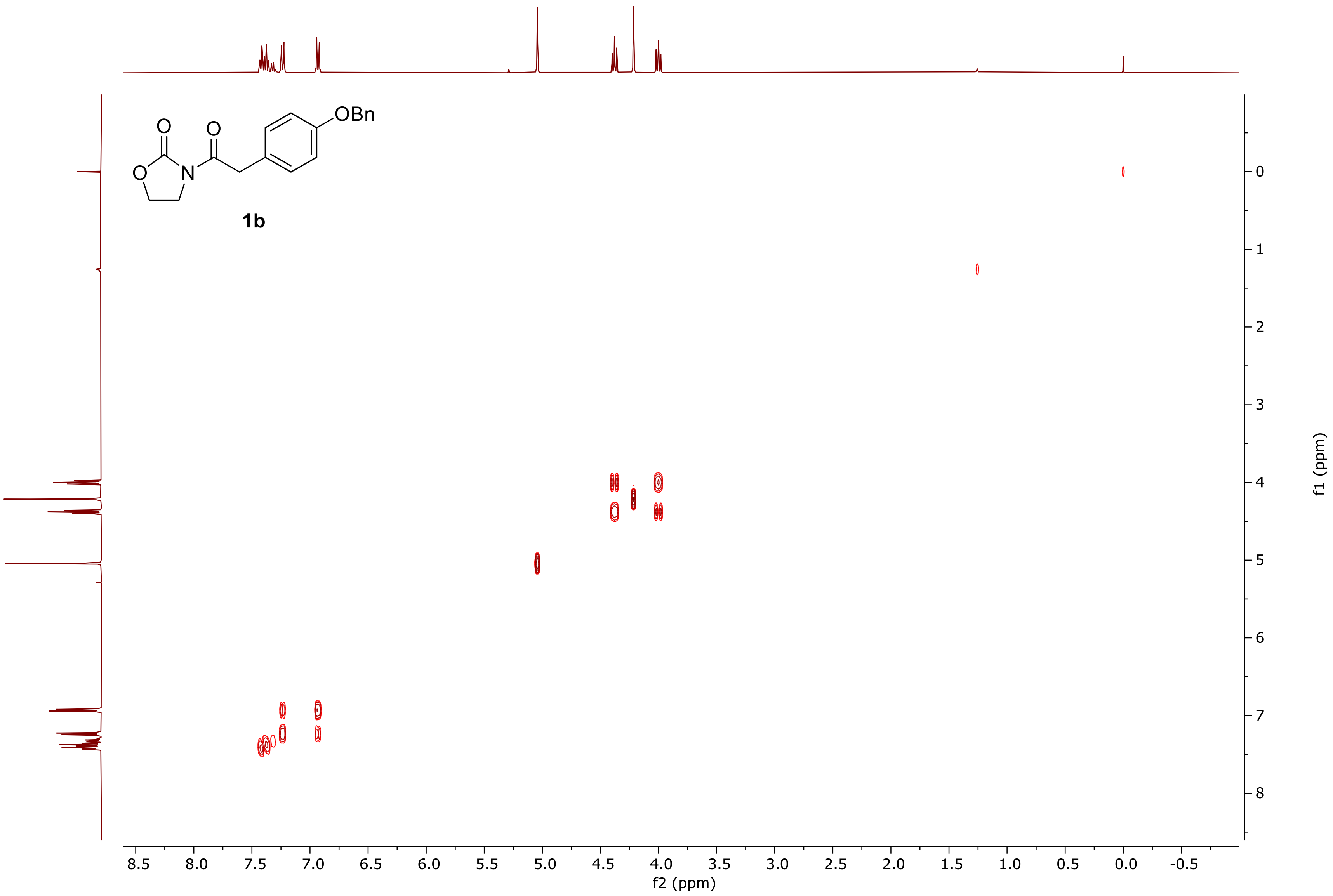

2D $^1$H -$^{13}$C HSQC (400 MHz, $CDCl_3$)

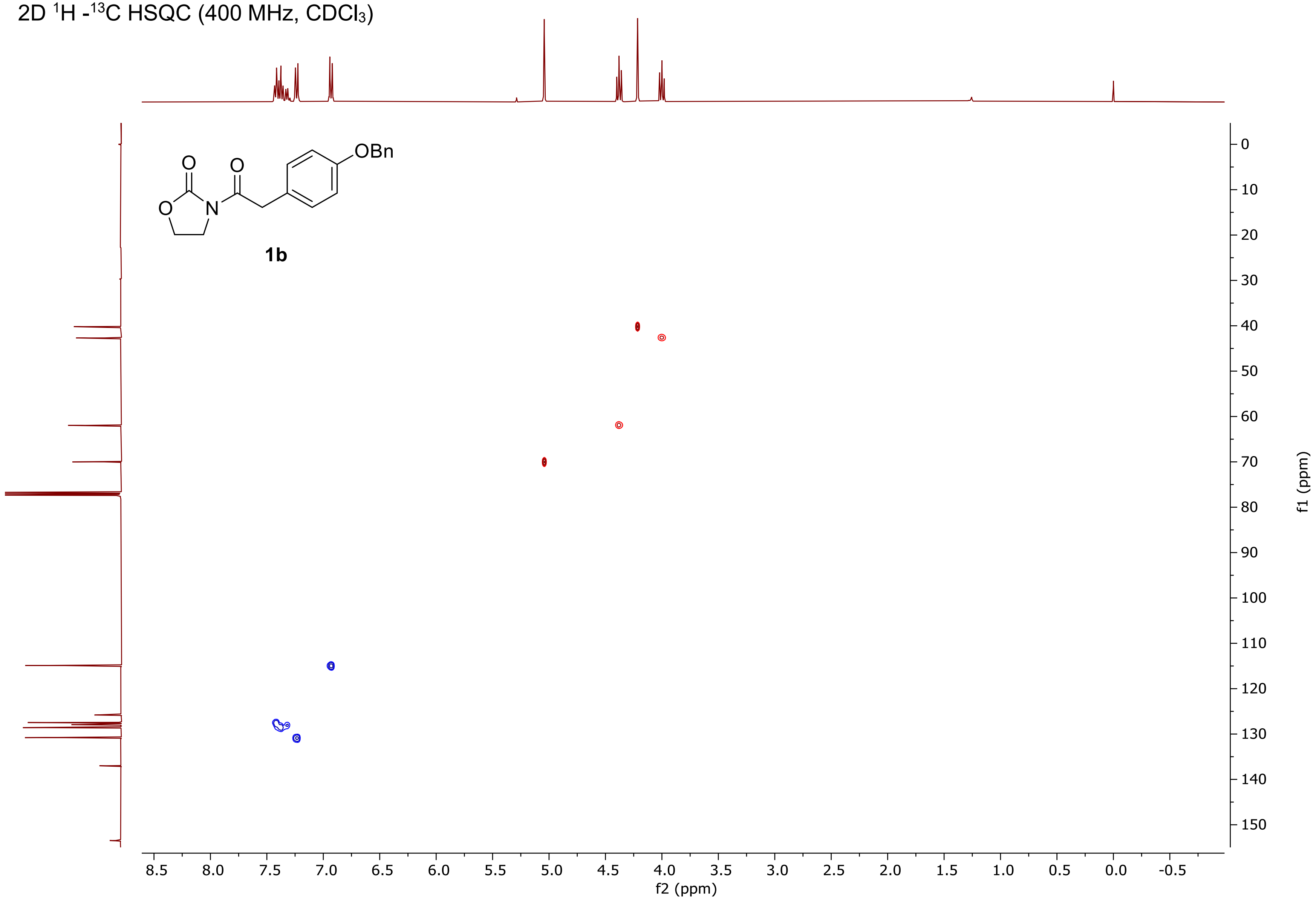

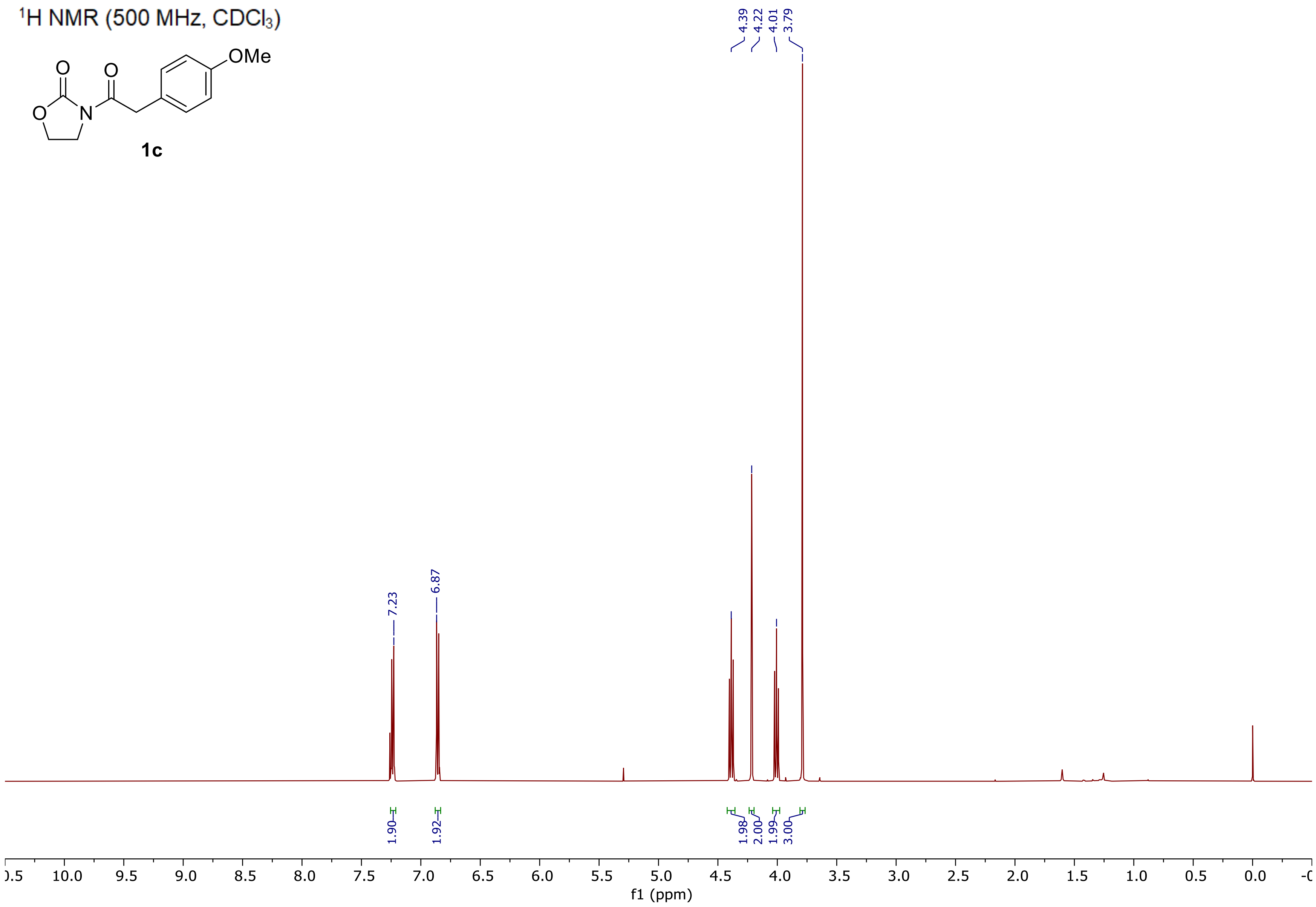

1H NMR (500 MHz, CDCl3)
OMe
O
O
O
N
1c
4.39
4.22
4.01
3.79
7.23
6.87
1.90
1.92
1.98
2.00
1.99
3.00
10.0 9.5 9.0 8.5 8.0 7.5 7.0 6.5 6.0 5.5 5.0 4.5 4.0 3.5 3.0 2.5 2.0 1.5 1.0 0.5 0.0
f1 (ppm)

$^{13}C\{^1H\}$ NMR (126 MHz, $CDCl_3$)

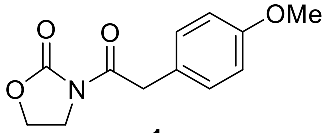

**1c**

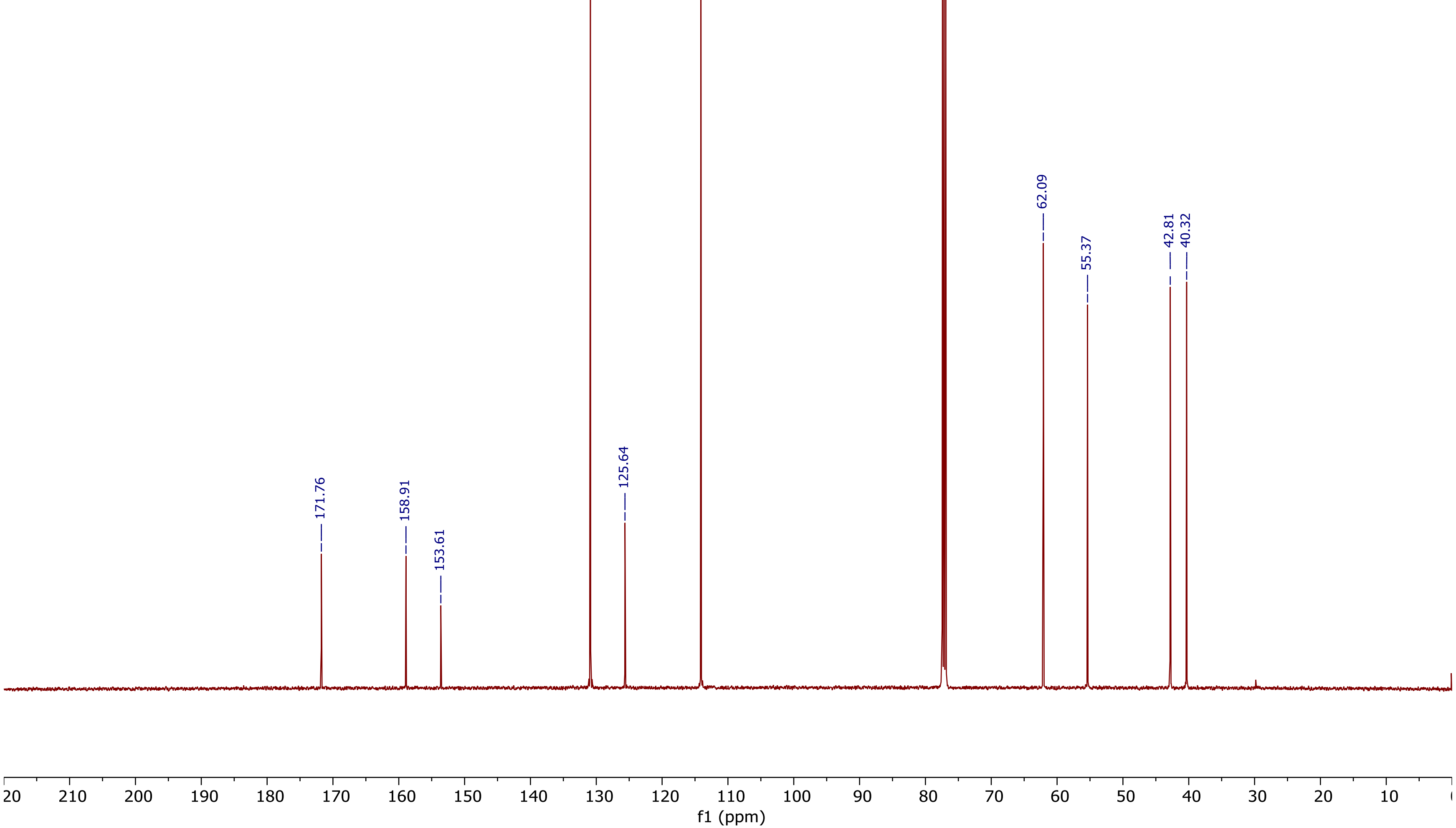

2D $^1$H -$^1$H COSY (500 MHz, $CDCl_3$)

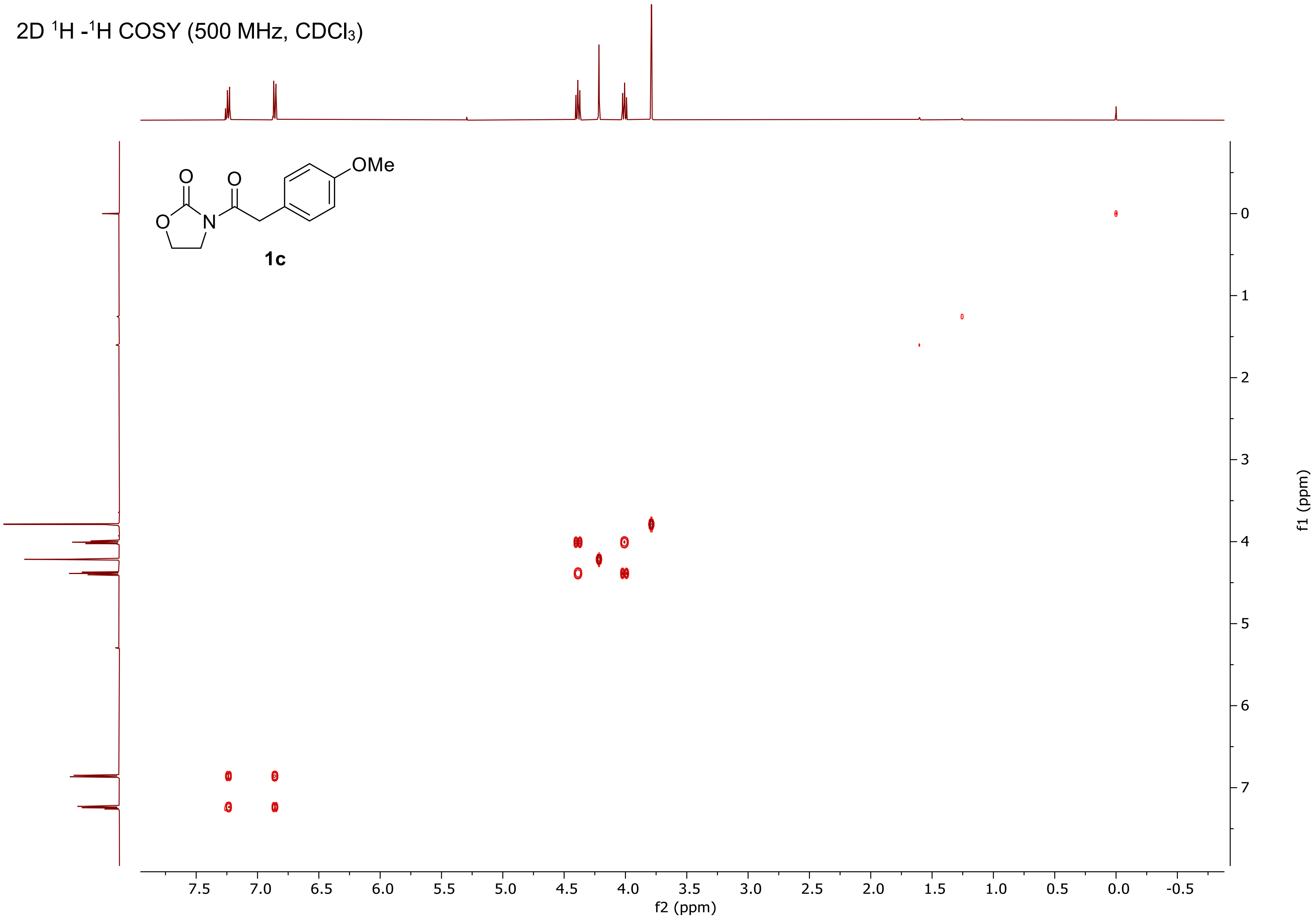

2D $^1$H -$^{13}$C HSQC (500 MHz, $CDCl_3$)

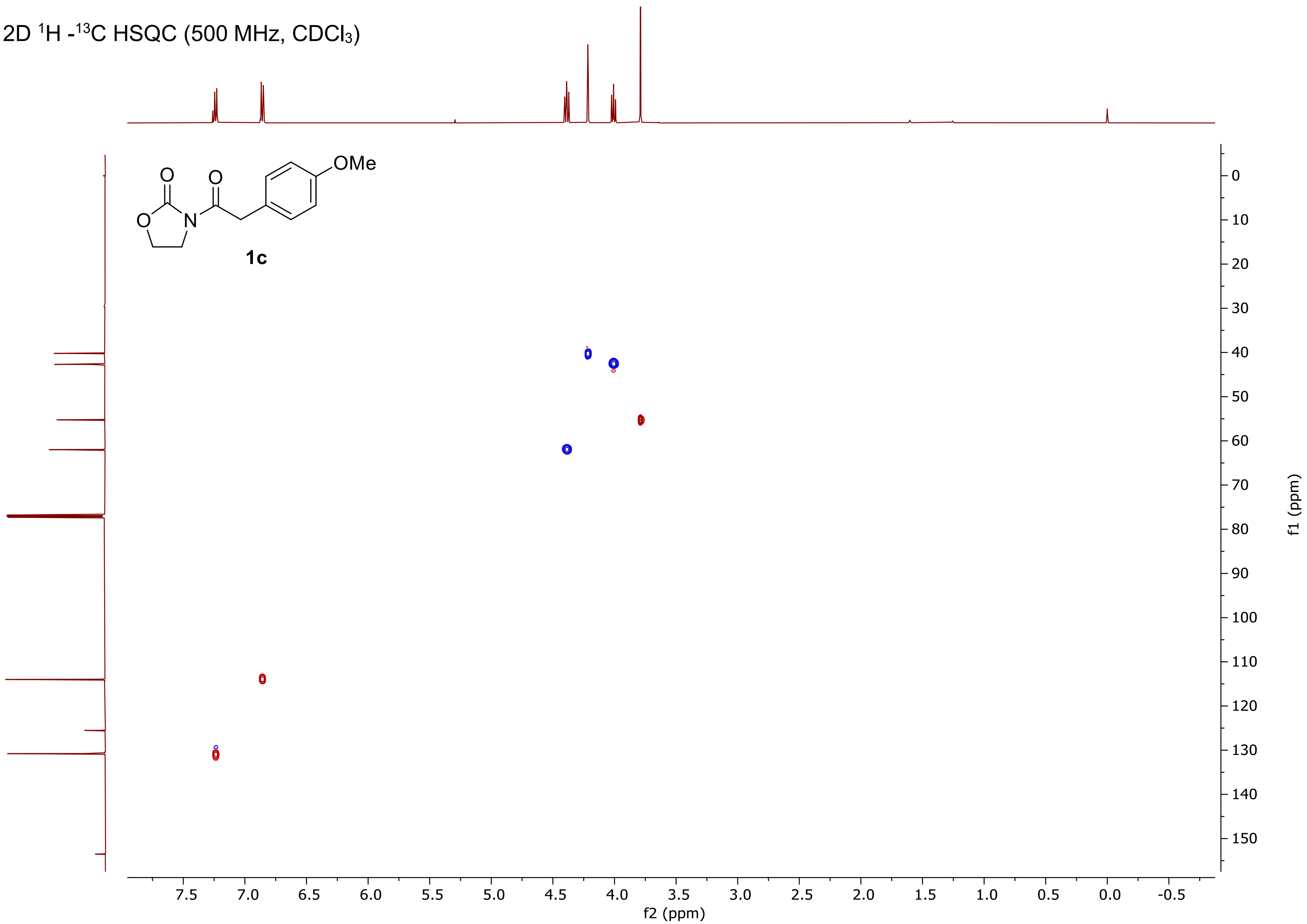

$^1H$ NMR (400 MHz, $CDCl_3$)

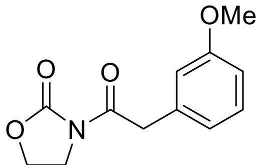


**1d**

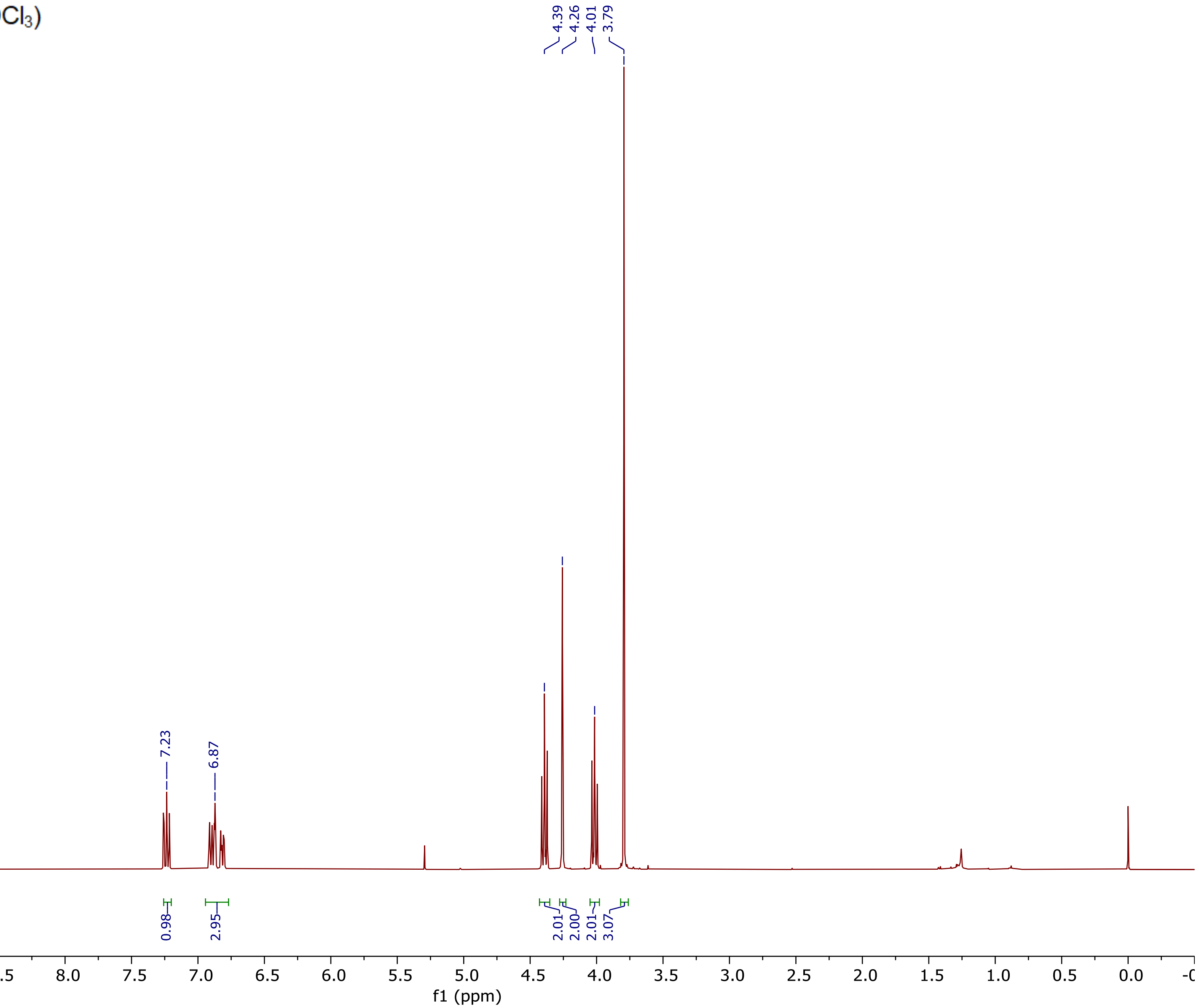

$^{13}C\{^1H\}$ NMR (101 MHz, $CDCl_3$)

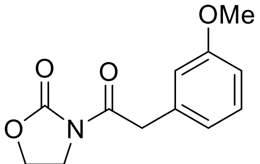

**1d**

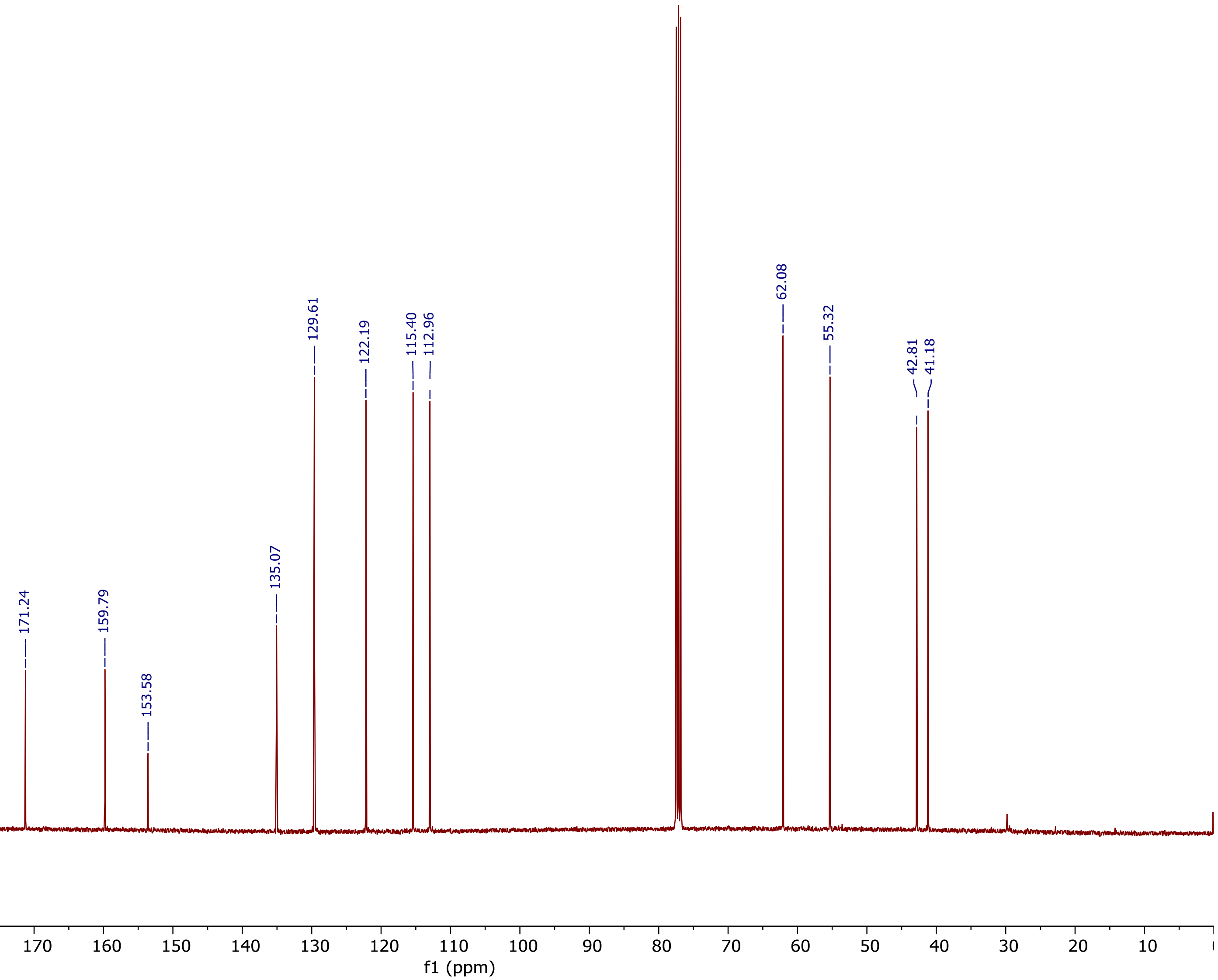

2D $^1H$ -$^1H$ COSY (400 MHz, $CDCl_3$)

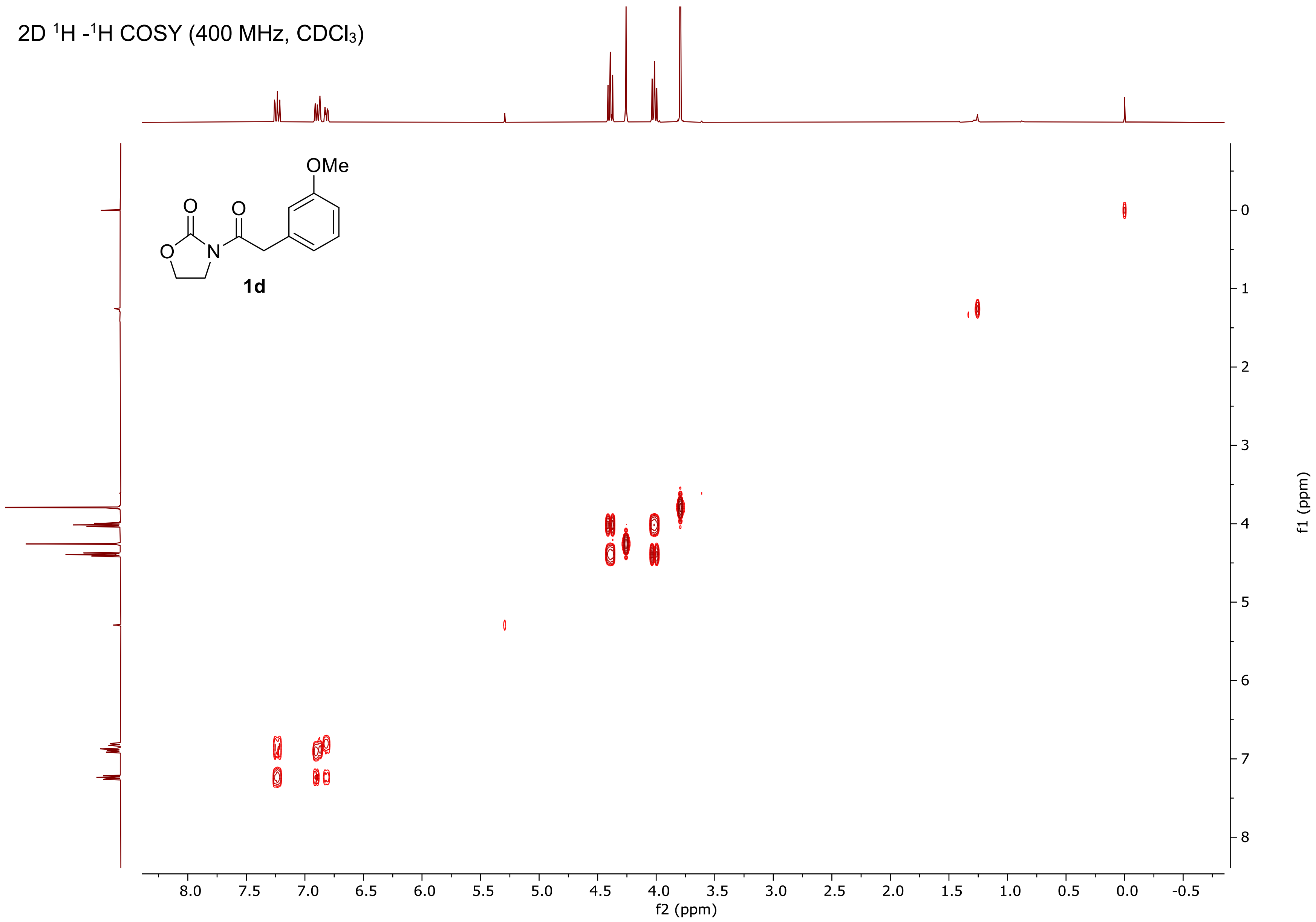

2D $^1$H -$^{13}$C HSQC (400 MHz, $CDCl_3$)

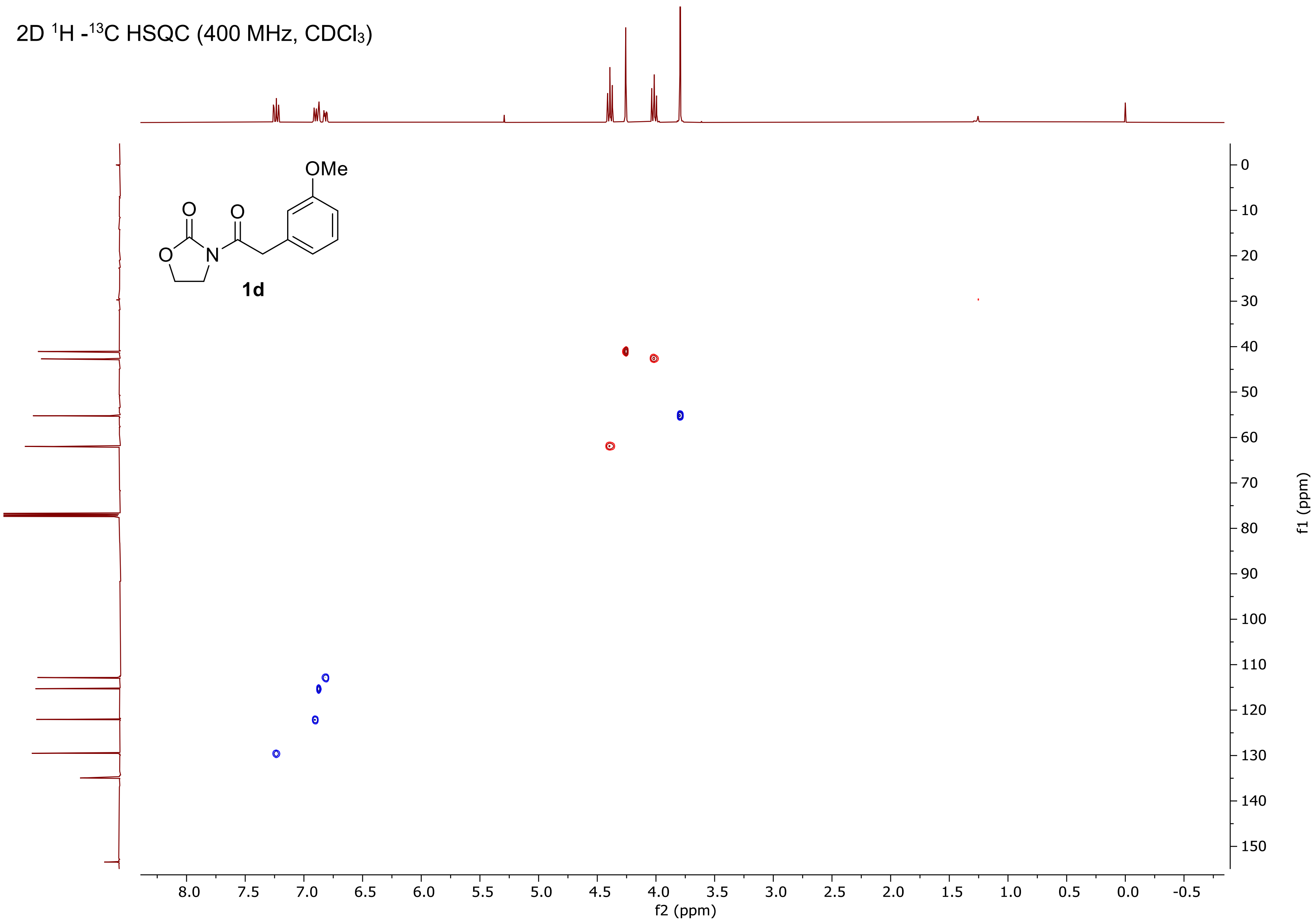

$^1H$ NMR (400 MHz, $CDCl_3$)

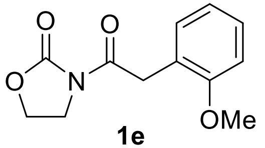

**1e**

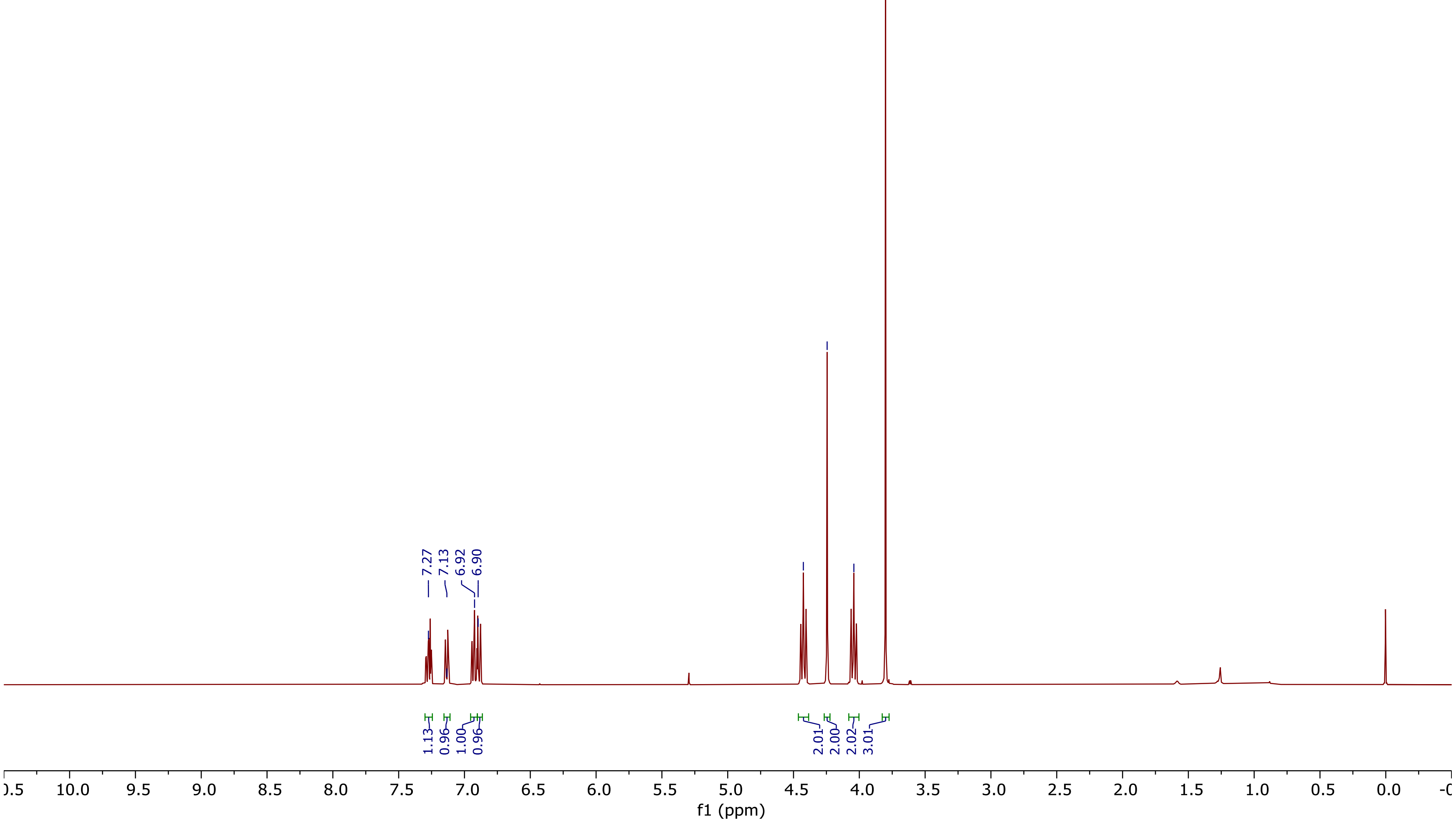

$^{13}C\{^{1}H\}$ NMR (101 MHz, $CDCl_3$)

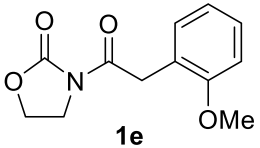

**1e**

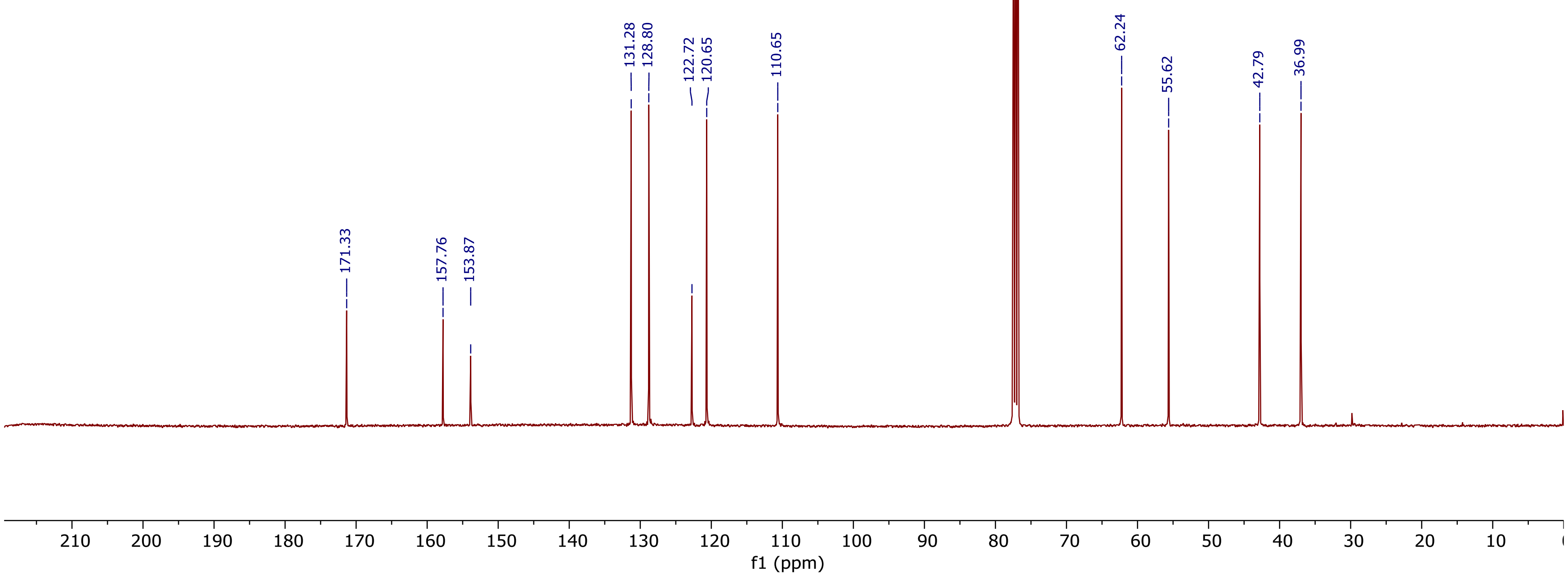

2D $^1$H -$^1$H COSY (400 MHz, $CDCl_3$)

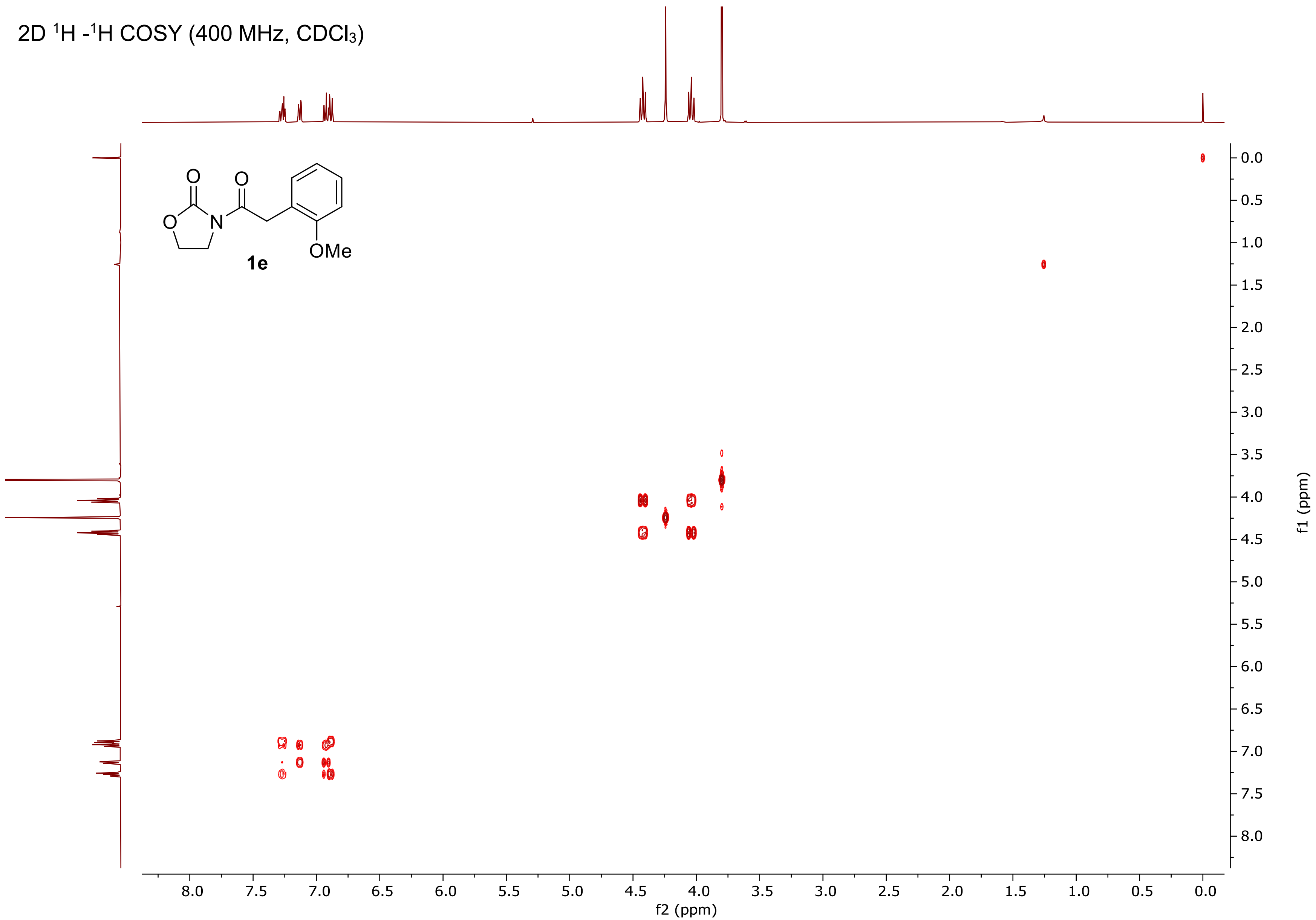

2D $^1$H -$^{13}$C HSQC (400 MHz, $CDCl_3$)

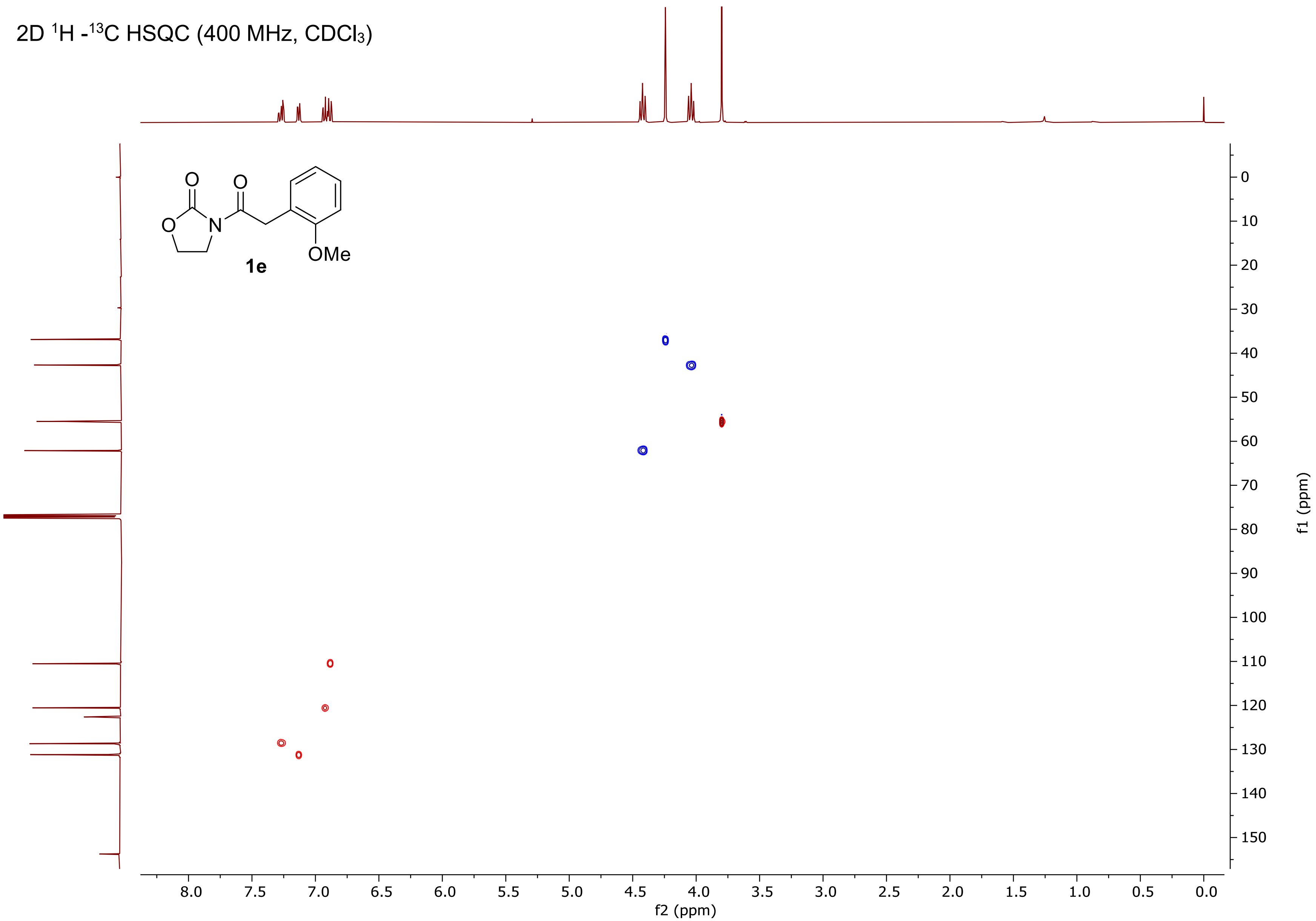

$^{1}H$ NMR (500 MHz, $CDCl_3$)

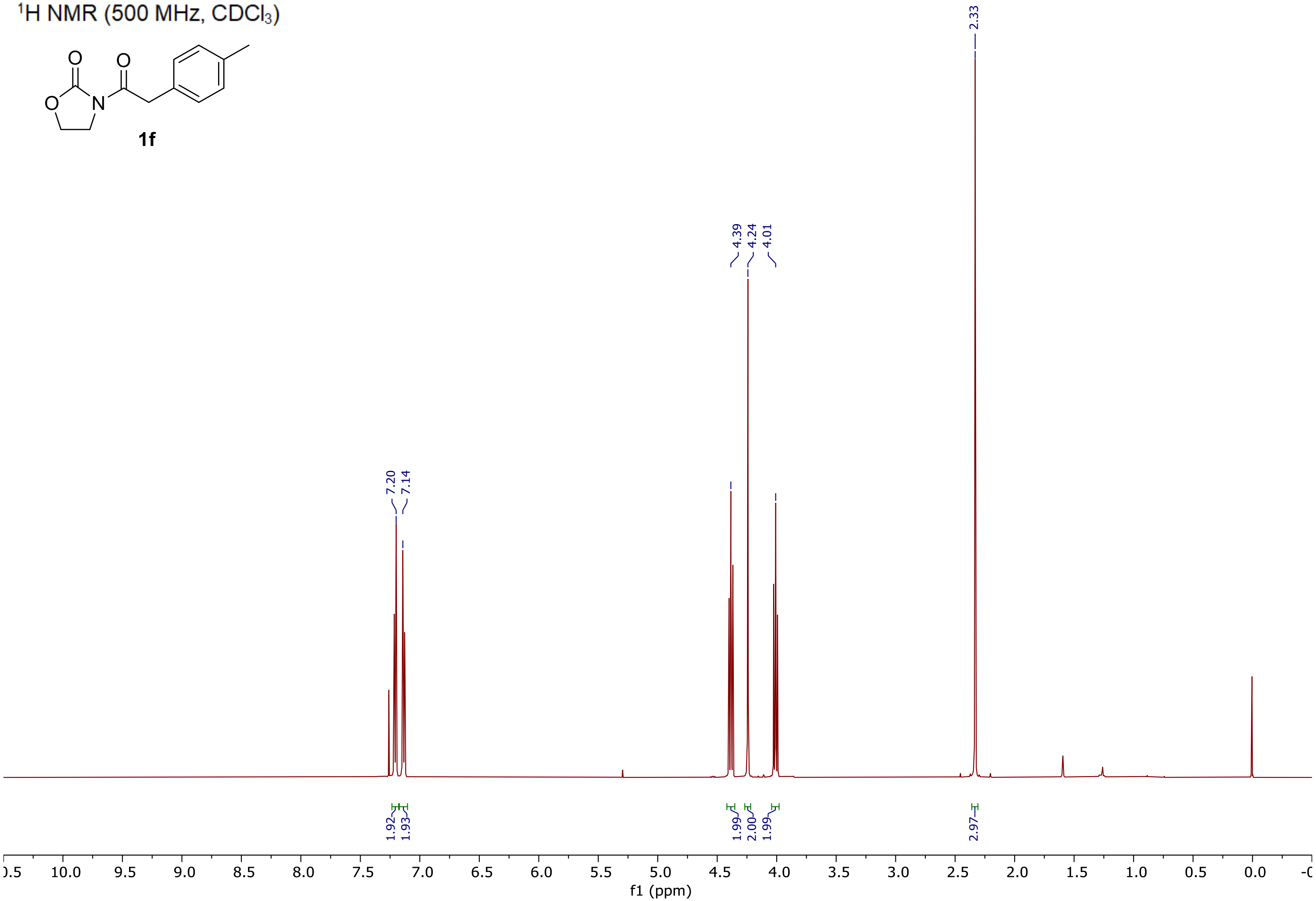

$^{13}C\{^{1}H\}$ NMR (126 MHz, $CDCl_3$)

**1f**

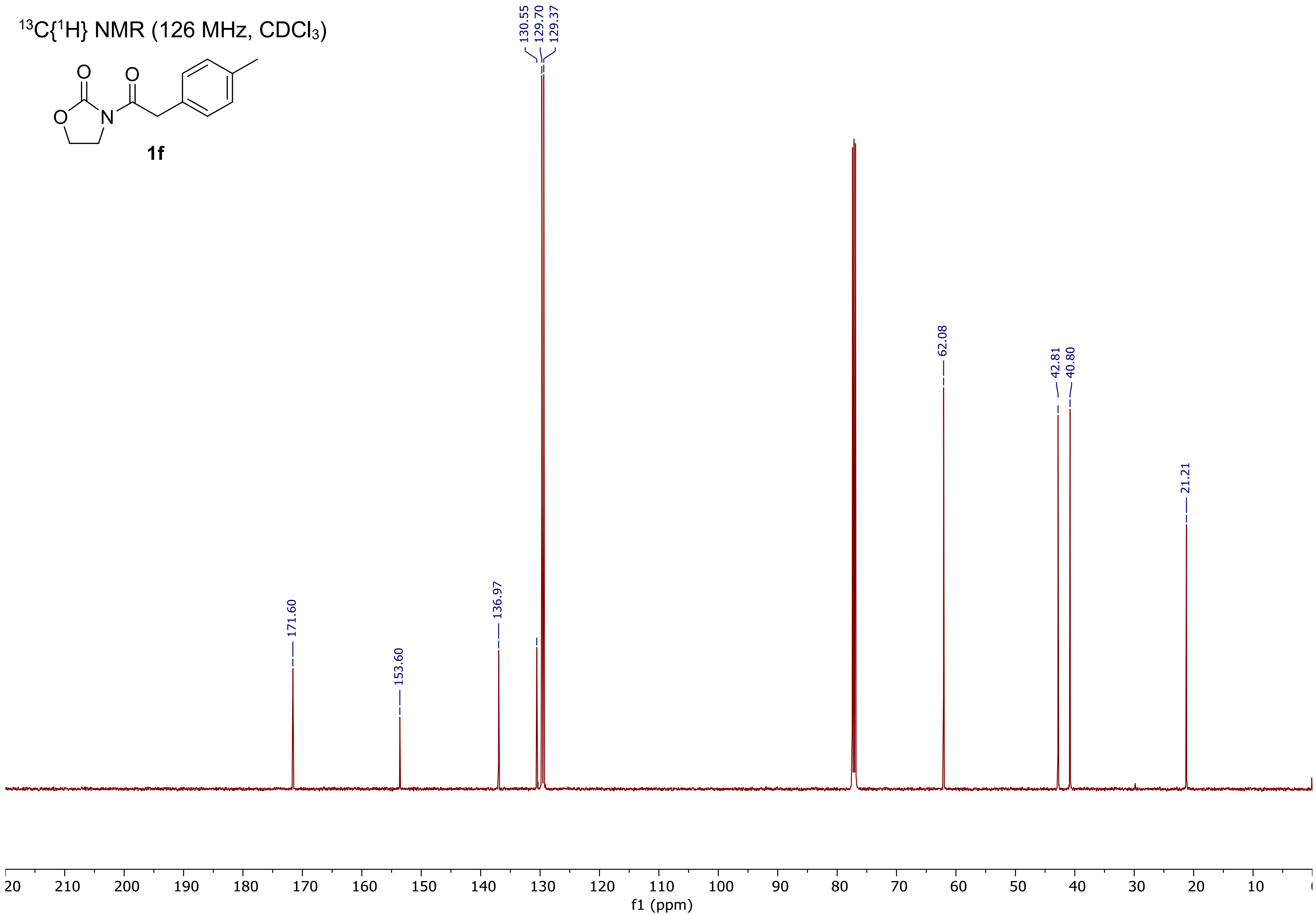

2D $^{1}H$ -$^{1}H$ COSY (500 MHz, $CDCl_3$)

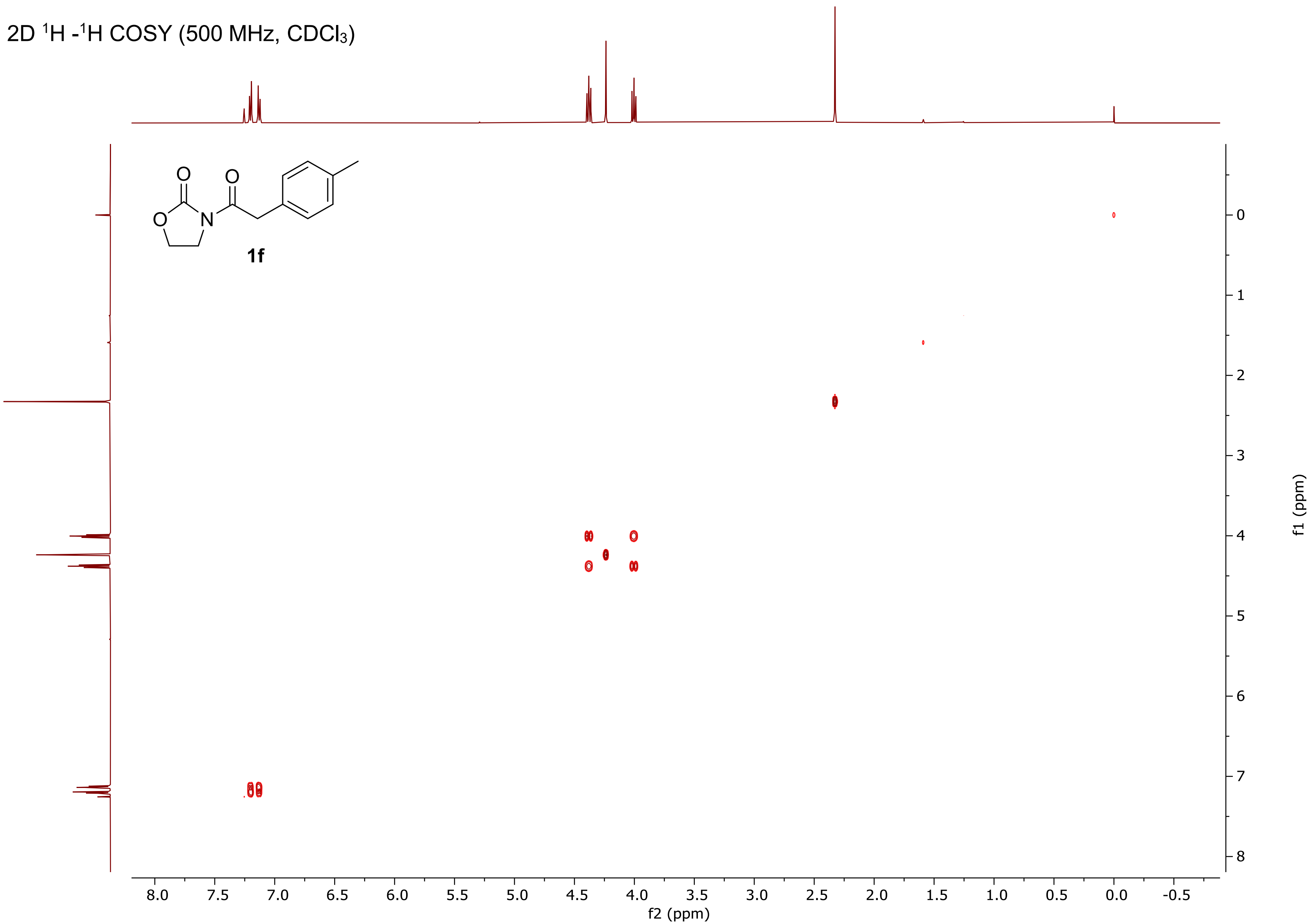

2D $^{1}H$ -$^{13}C$ HSQC (500 MHz, $CDCl_3$)

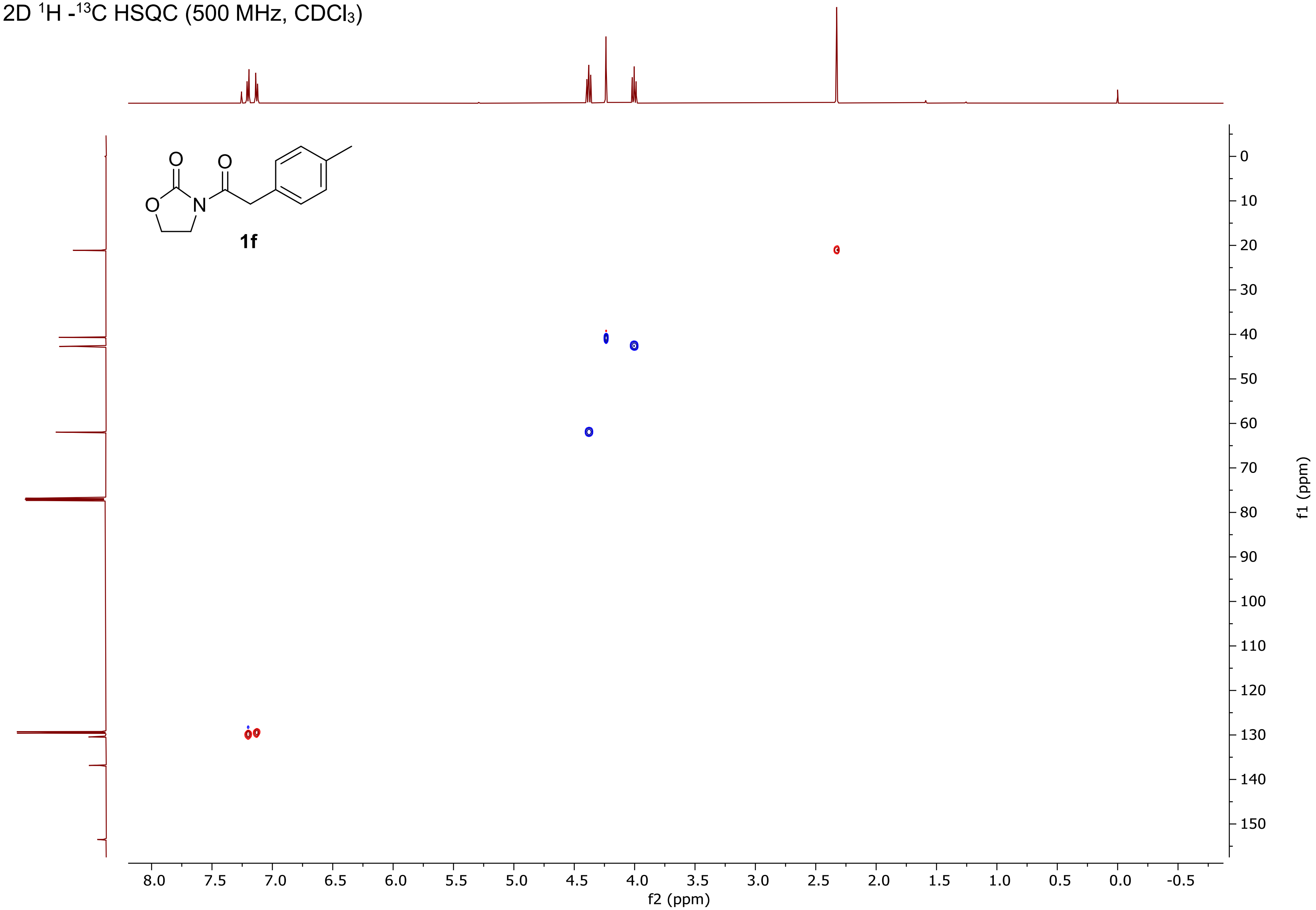

$^{1}$H NMR (500 MHz, $CDCl_3$)

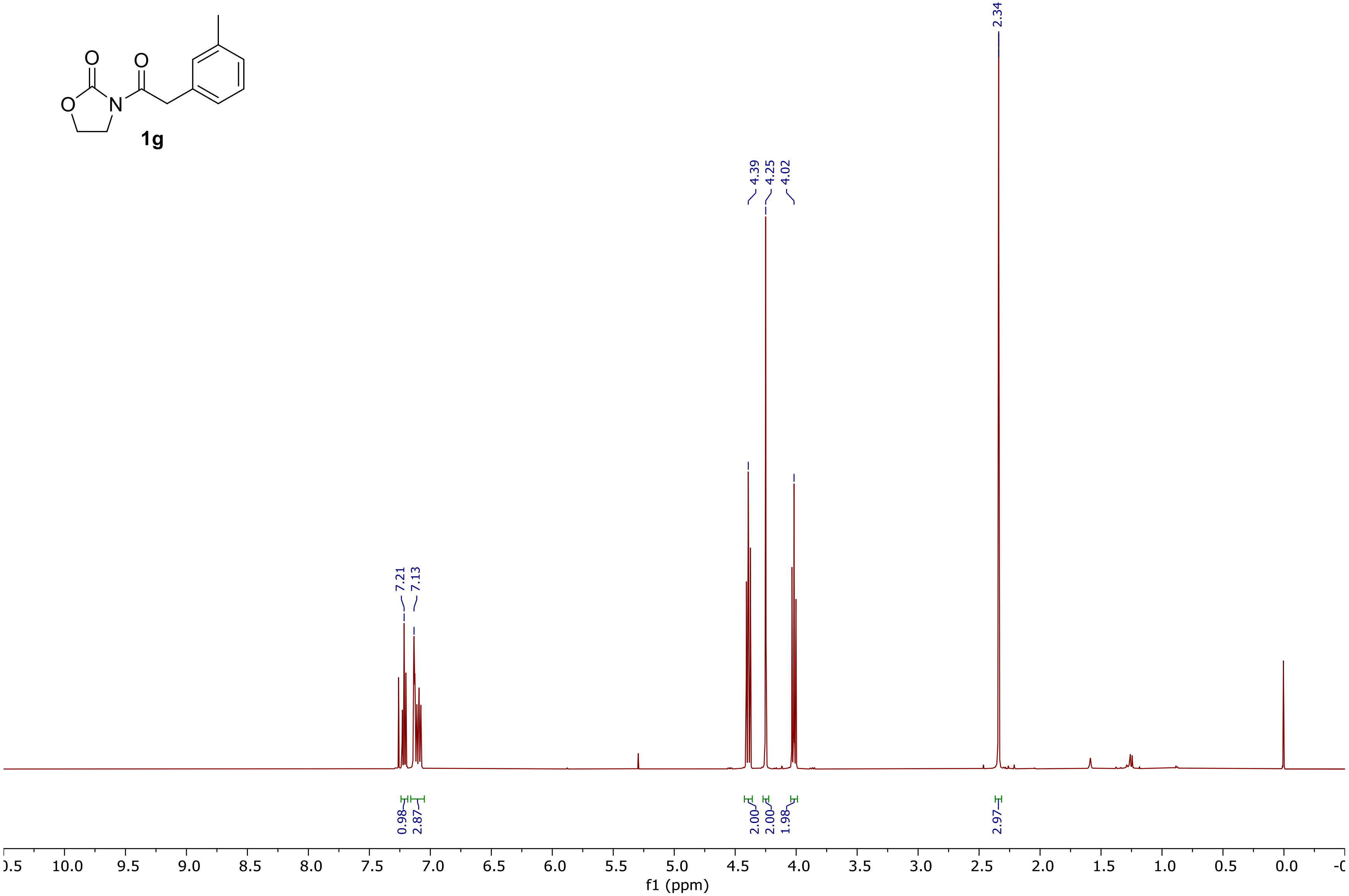

$^{13}C\{^1H\}$ NMR (126 MHz, $CDCl_3$)

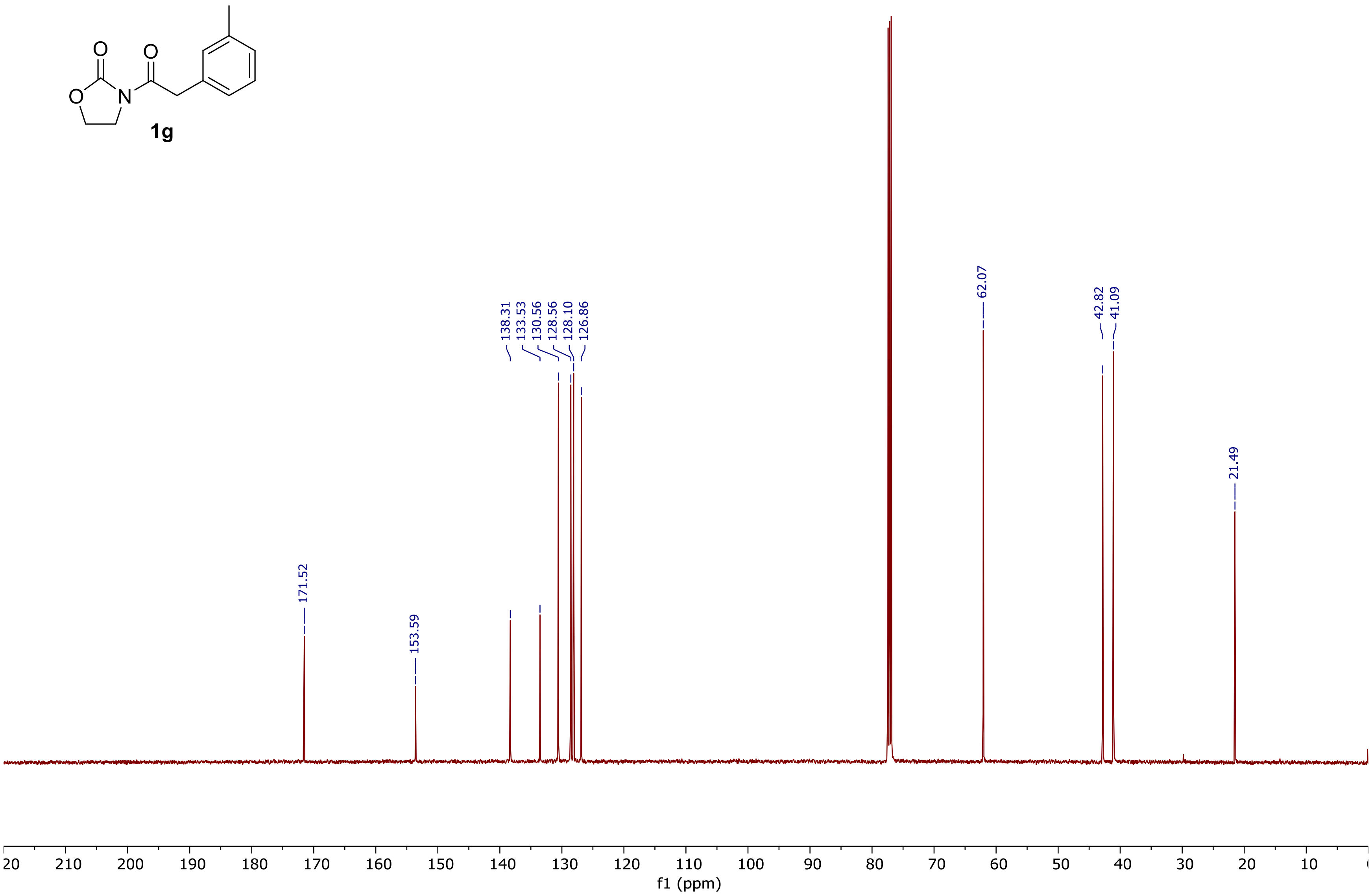

2D $^{1}H$ -$^{1}H$ COSY (500 MHz, $CDCl_3$)

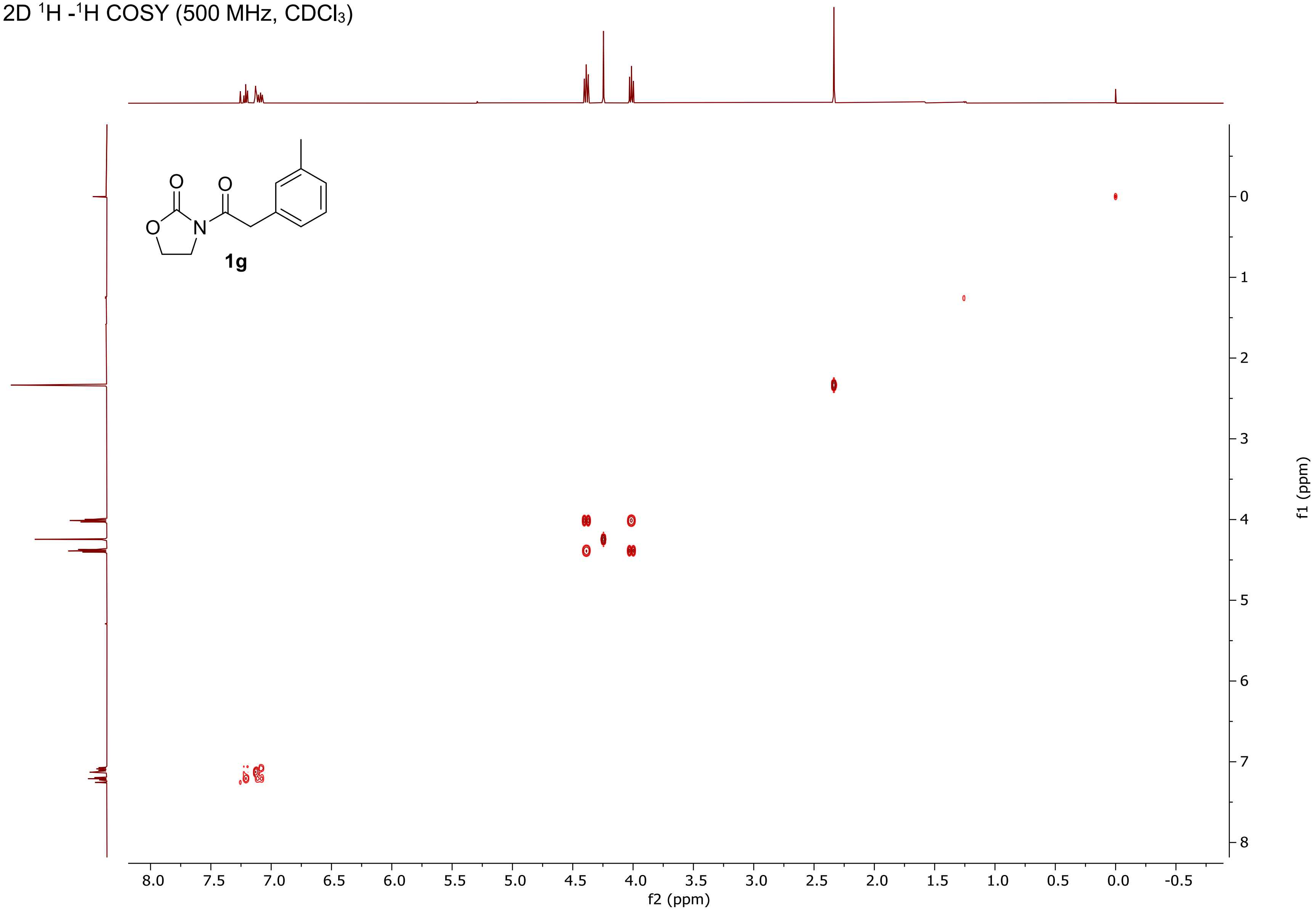

2D $^{1}H$ -$^{13}C$ HSQC (500 MHz, $CDCl_3$)

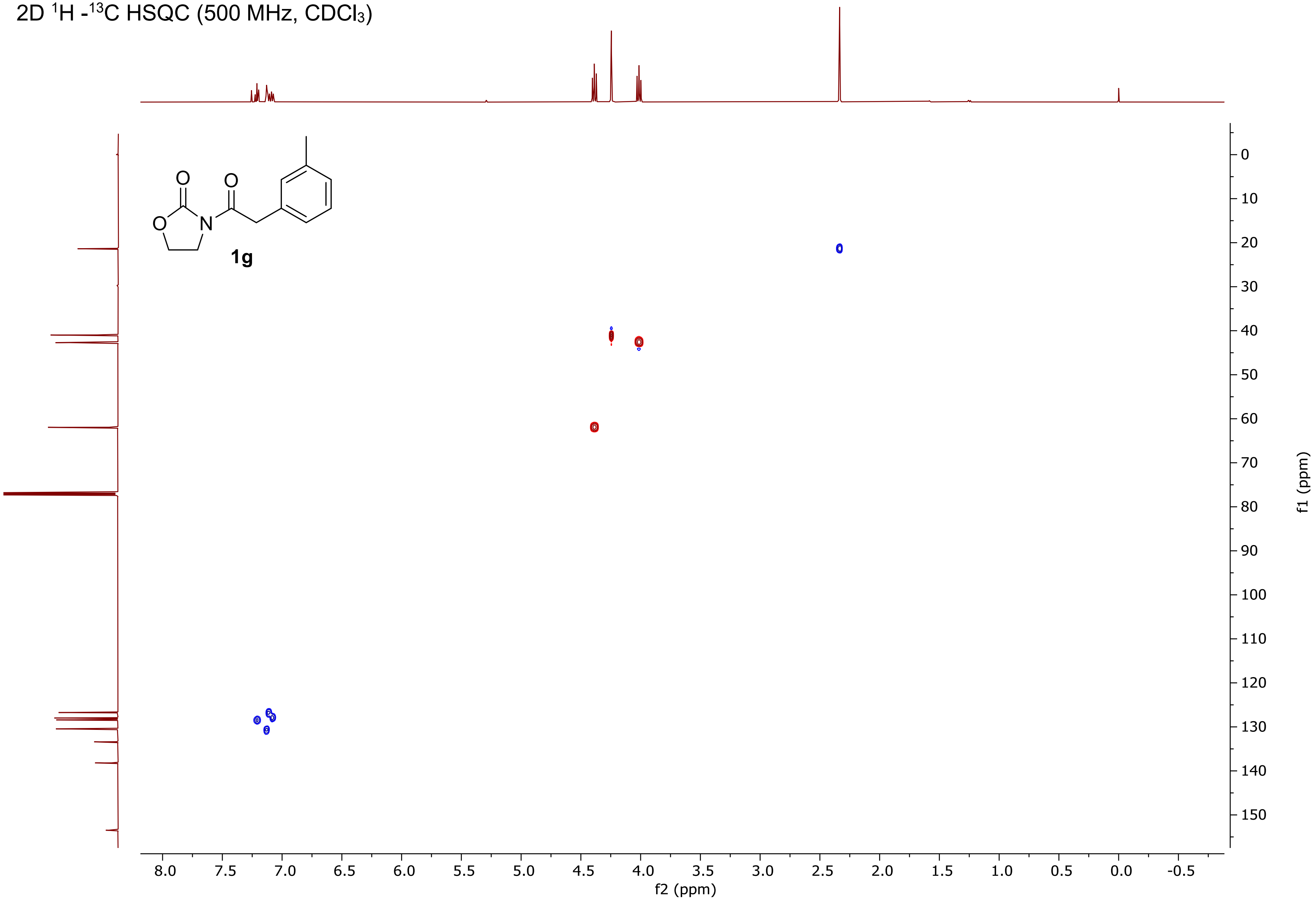

[1]H NMR (500 MHz, $CDCl_3$)

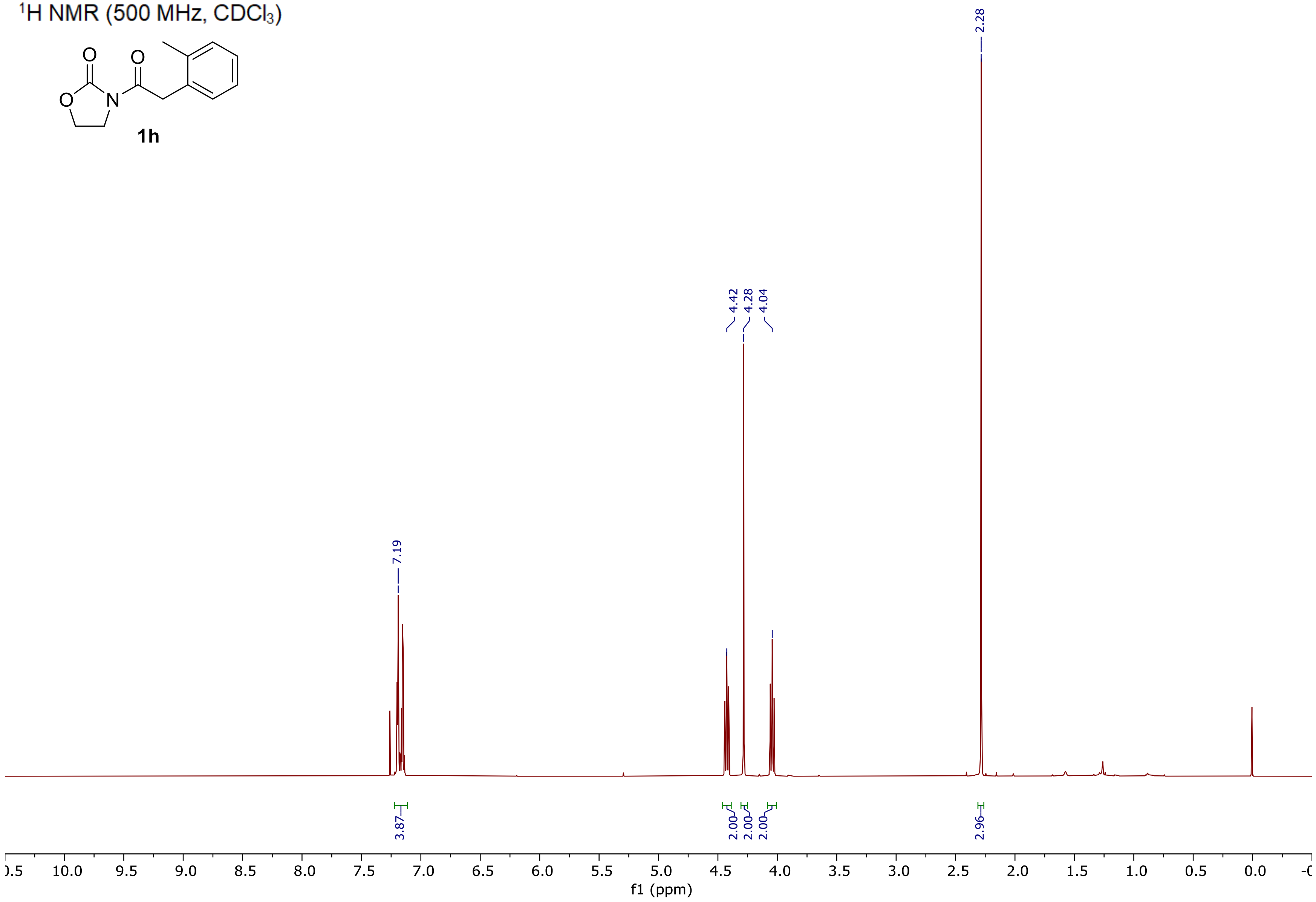

$^{13}C\{^1H\}$ NMR (126 MHz, $CDCl_3$)

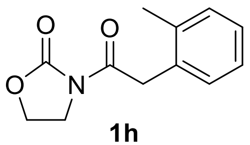

**1h**

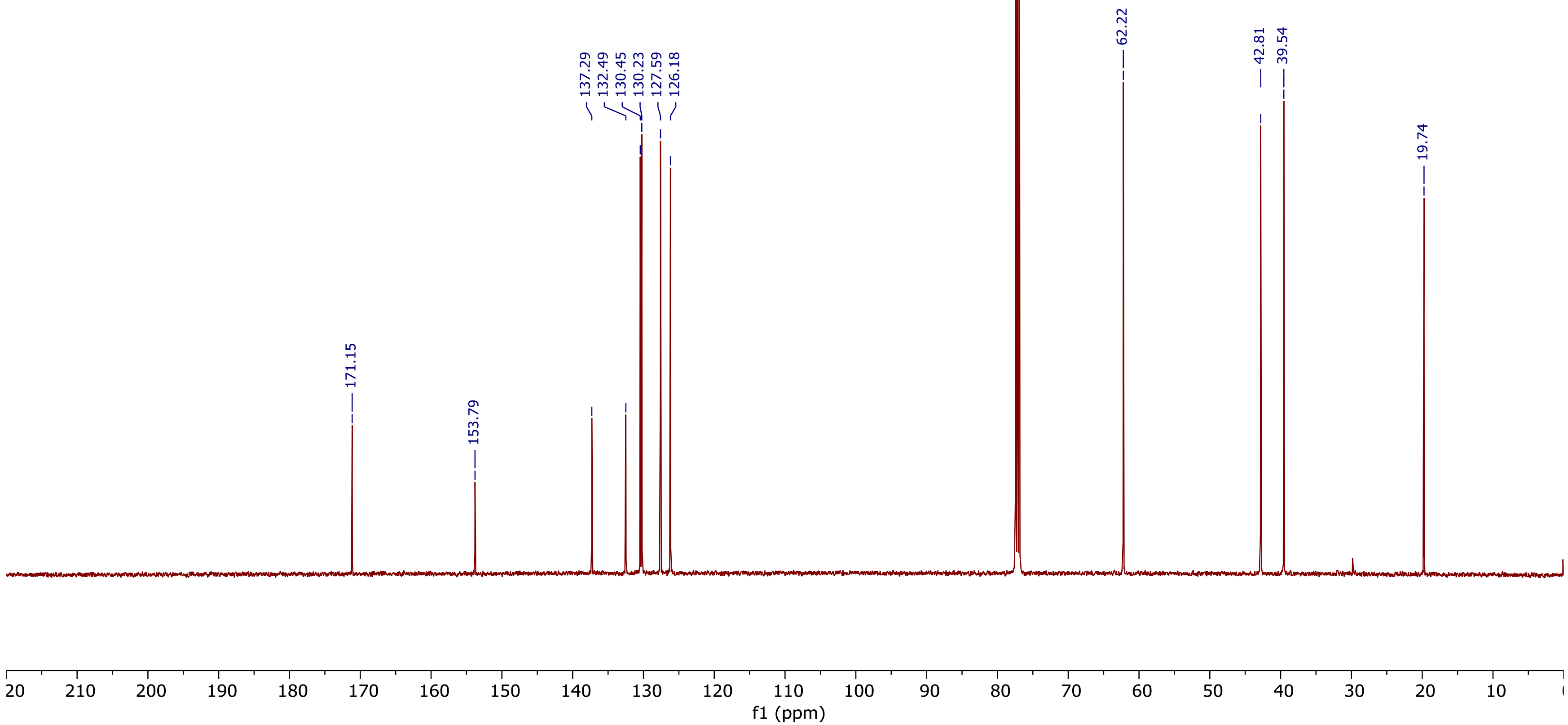

2D $^1H$ -$^1H$ COSY (500 MHz, $CDCl_3$)

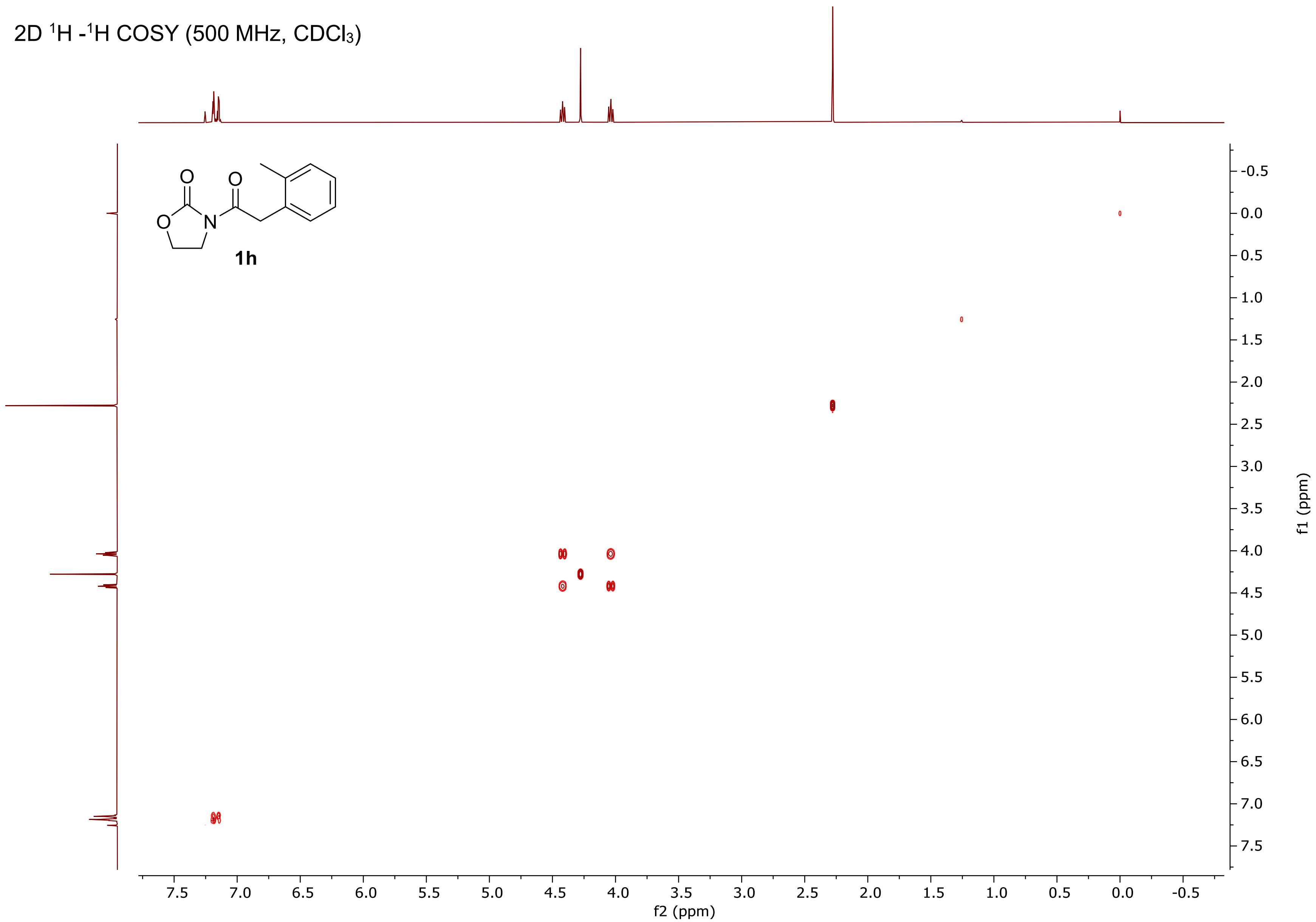

2D $^{1}H$ -$^{13}C$ HSQC (500 MHz, $CDCl_3$)

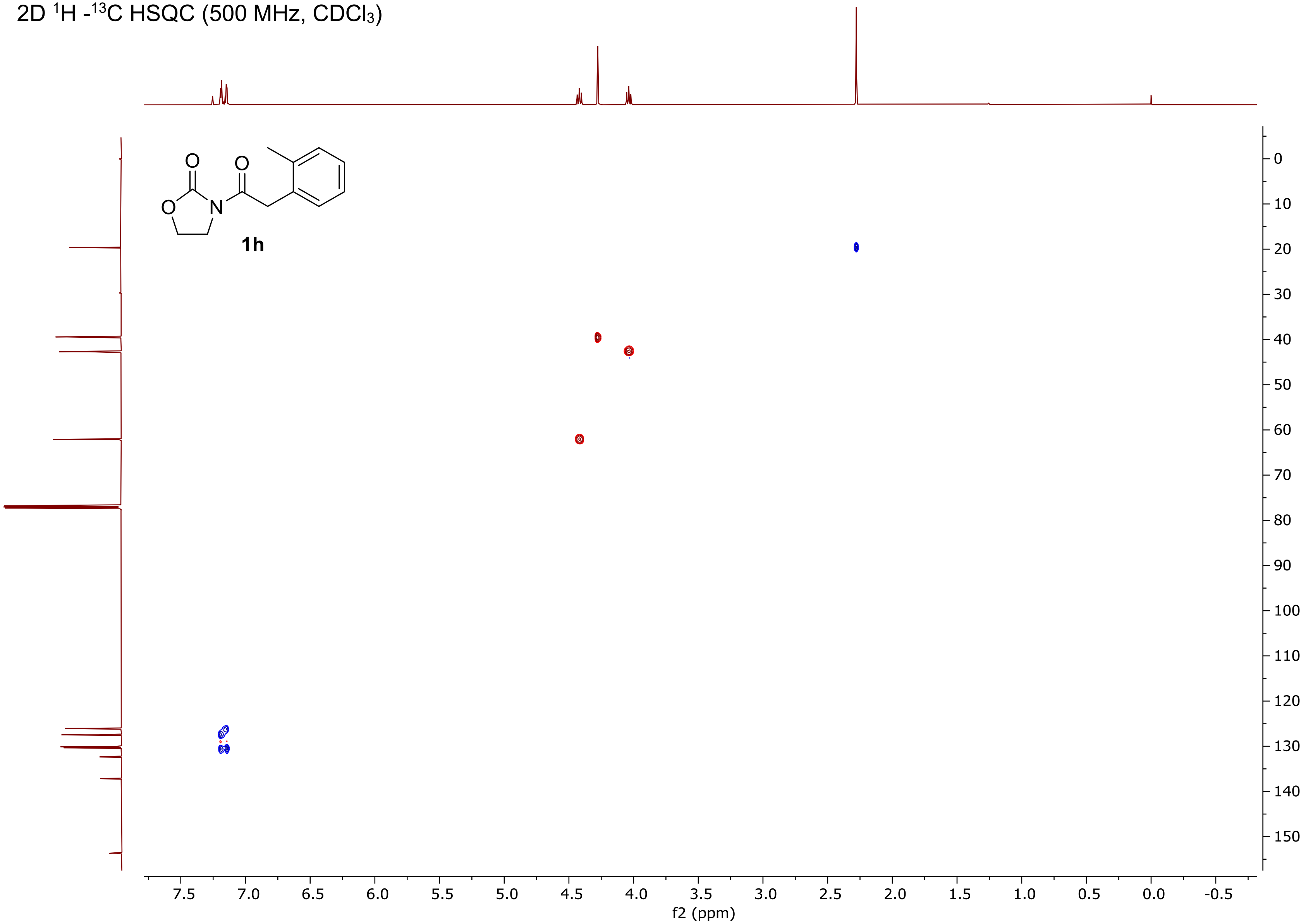

$^1H$ NMR (500 MHz, $CDCl_3$)

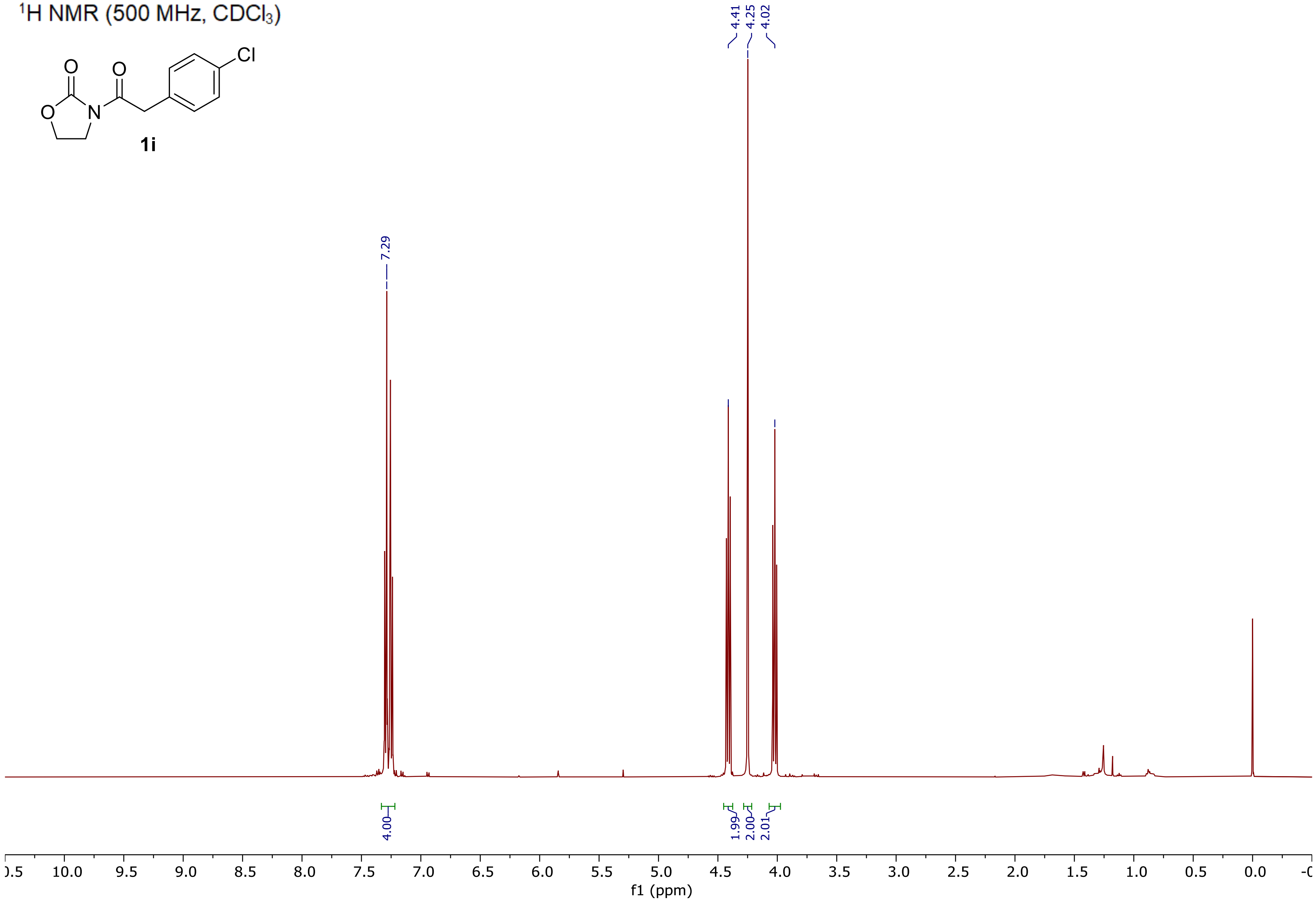

**1i**

$^{13}C\{^{1}H\}$ NMR (126 MHz, $CDCl_3$)

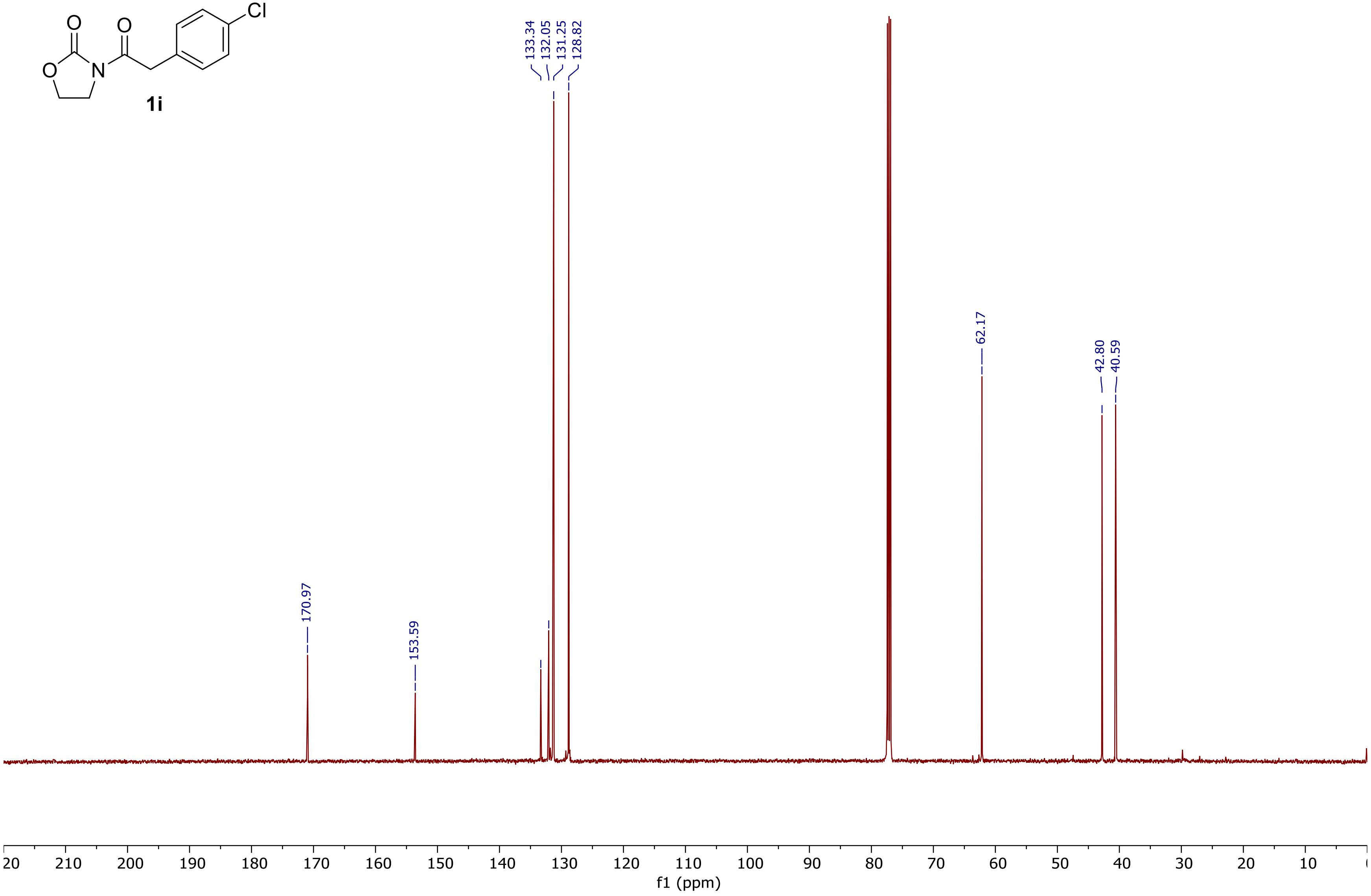

2D $^1H$ -$^1H$ COSY (500 MHz, $CDCl_3$)

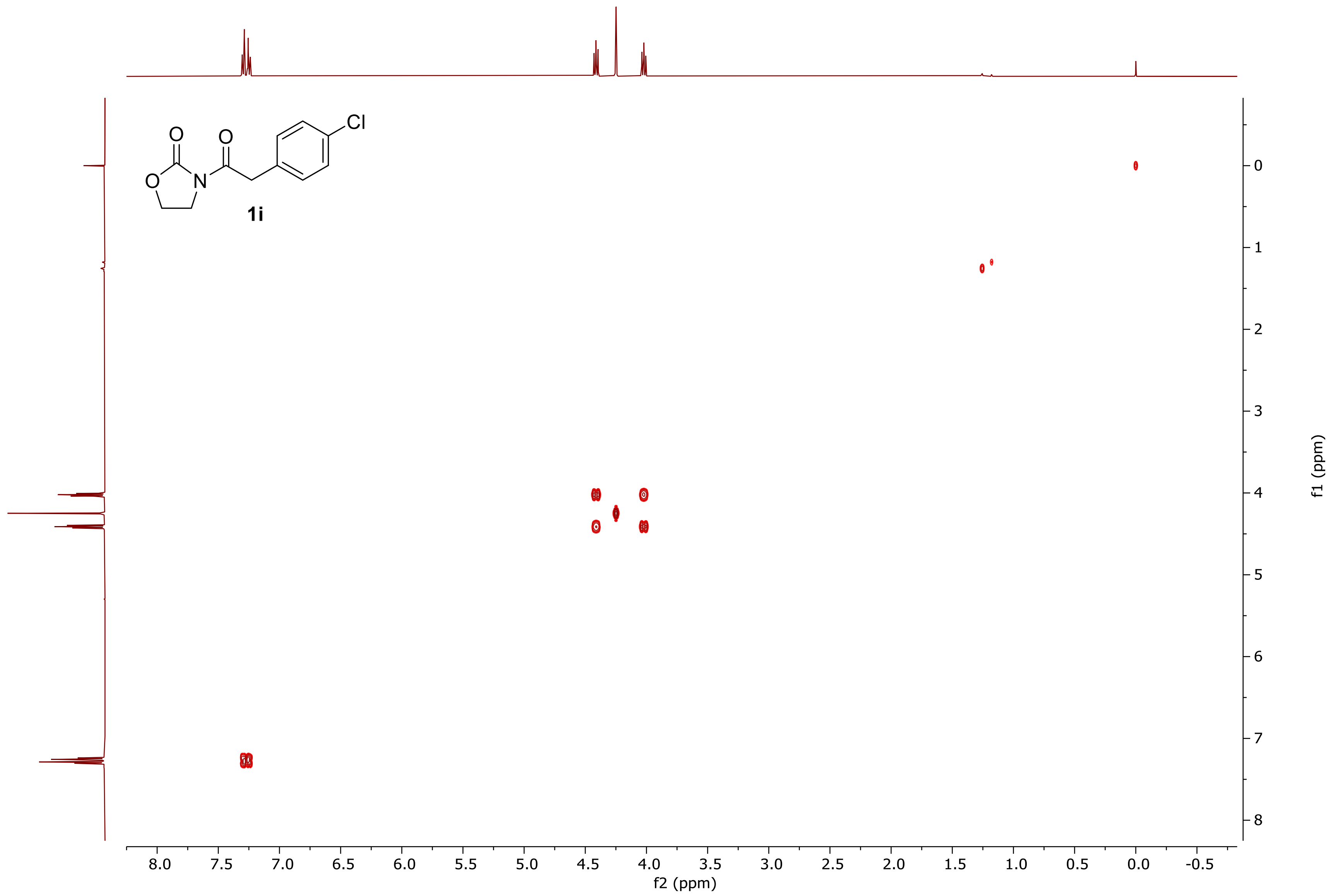

2D $^{1}H$ -$^{13}C$ HSQC (500 MHz, $CDCl_3$)

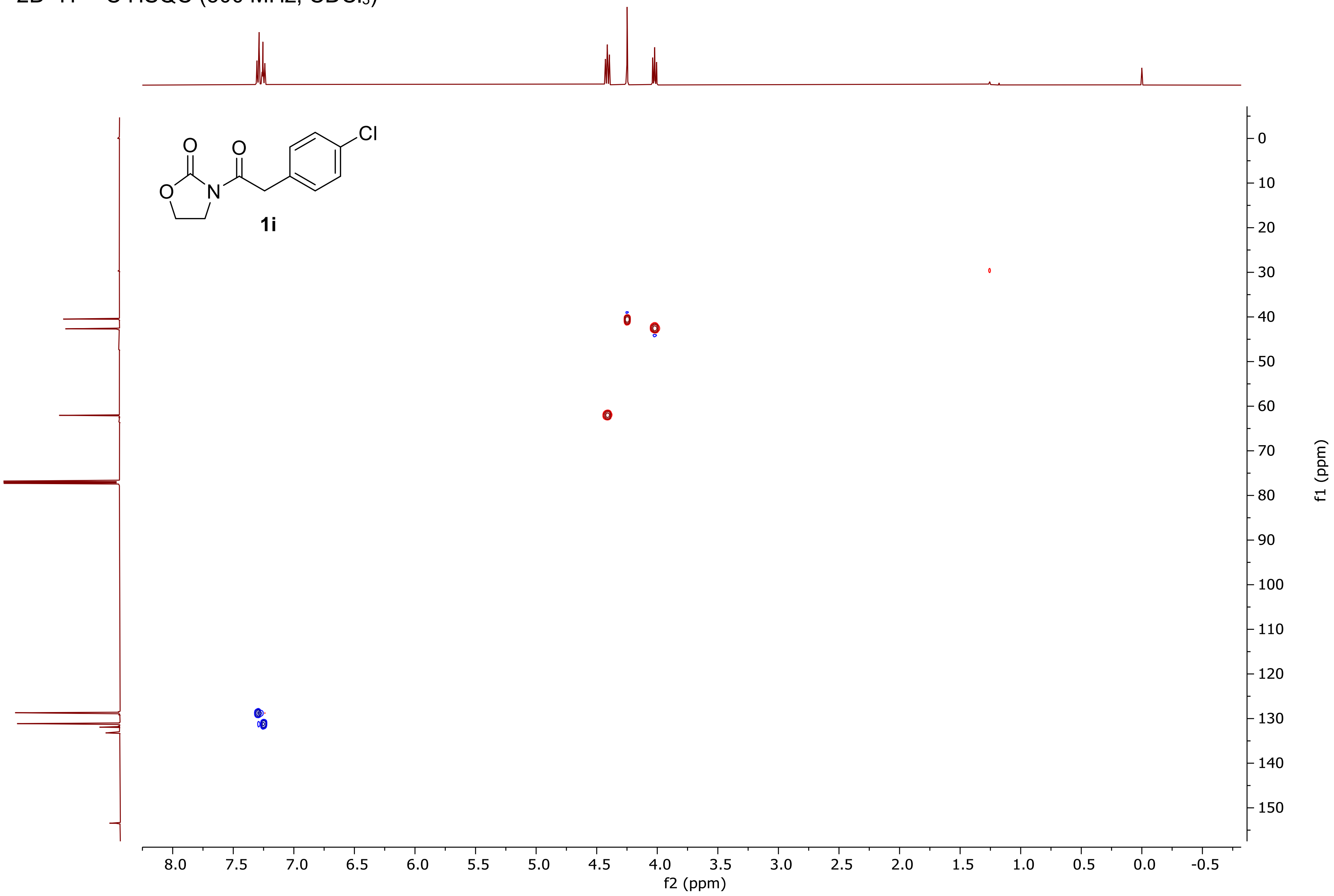

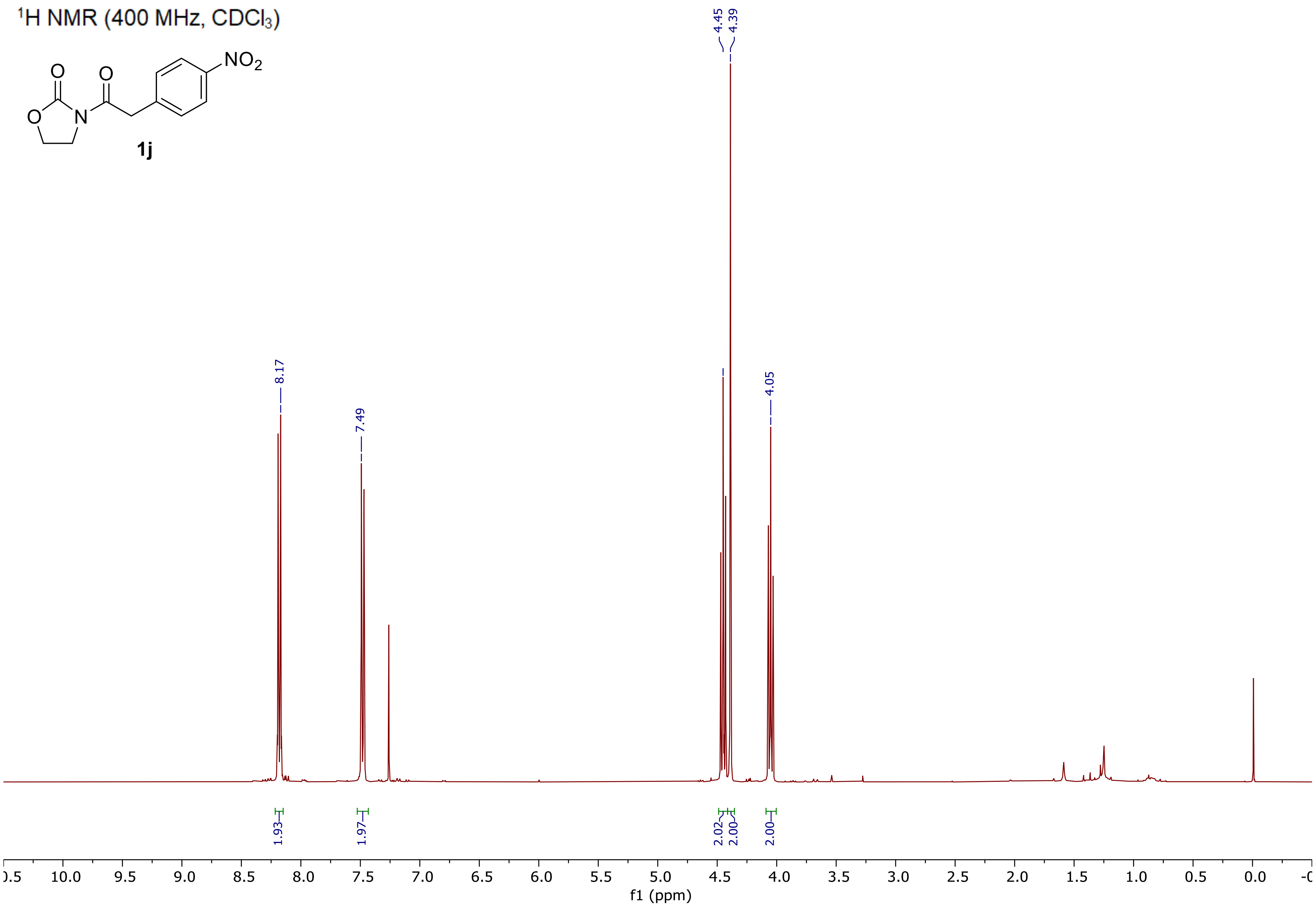

$^1$H NMR (400 MHz, $CDCl_3$)
$NO_2$
1j
8.17
7.49
4.45
4.39
4.05
1.93
1.97
2.02
2.00
2.00
f1 (ppm)

$^{13}C\{^{1}H\}$ NMR (101 MHz, $CDCl_3$)

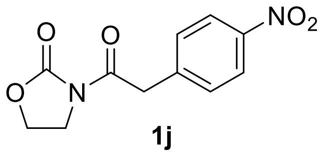


**1j**

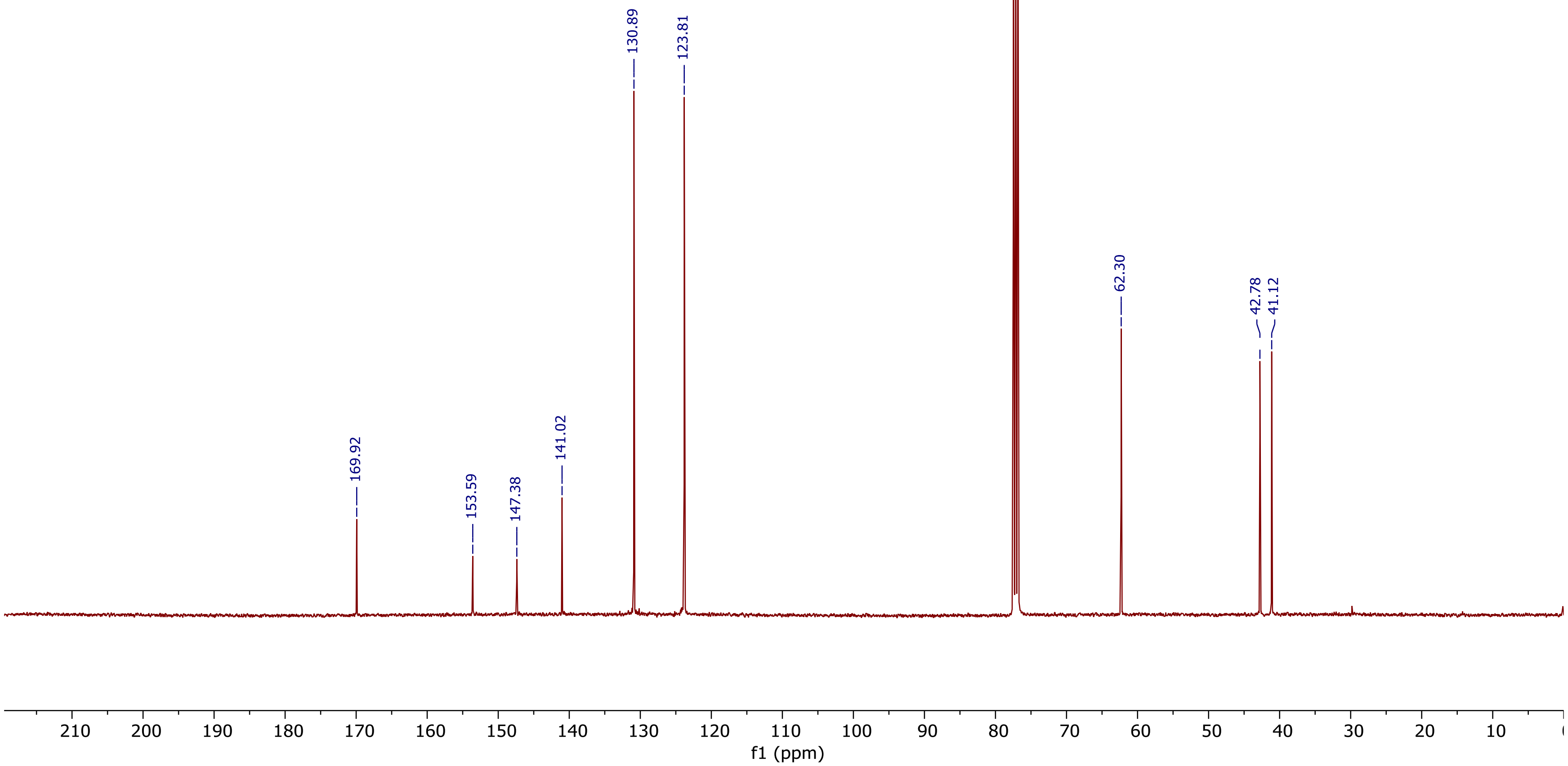

2D ¹H -¹H COSY (400 MHz, $CDCl_3$)

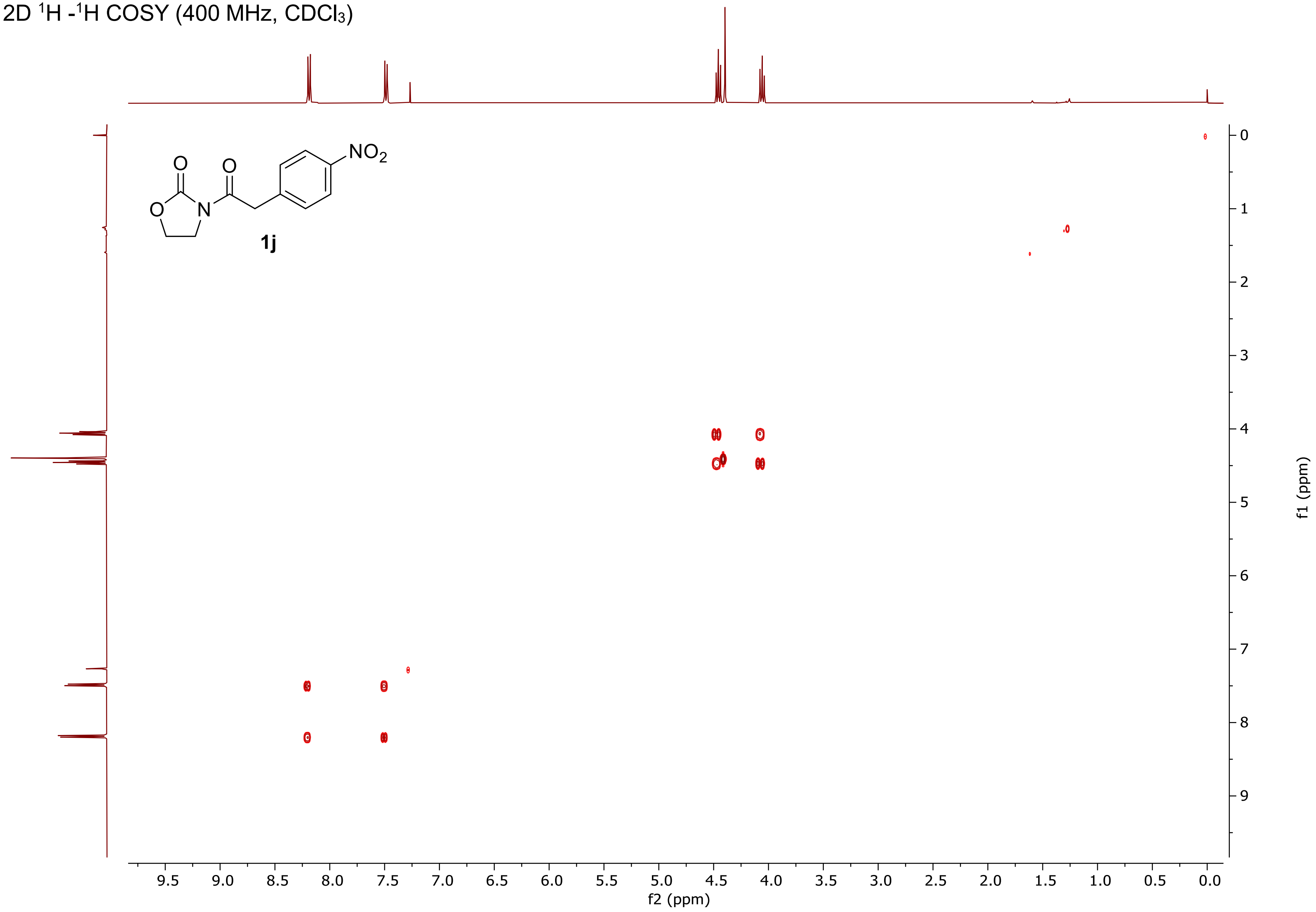

2D $^{1}H$ -$^{13}C$ HSQC (400 MHz, $CDCl_3$)

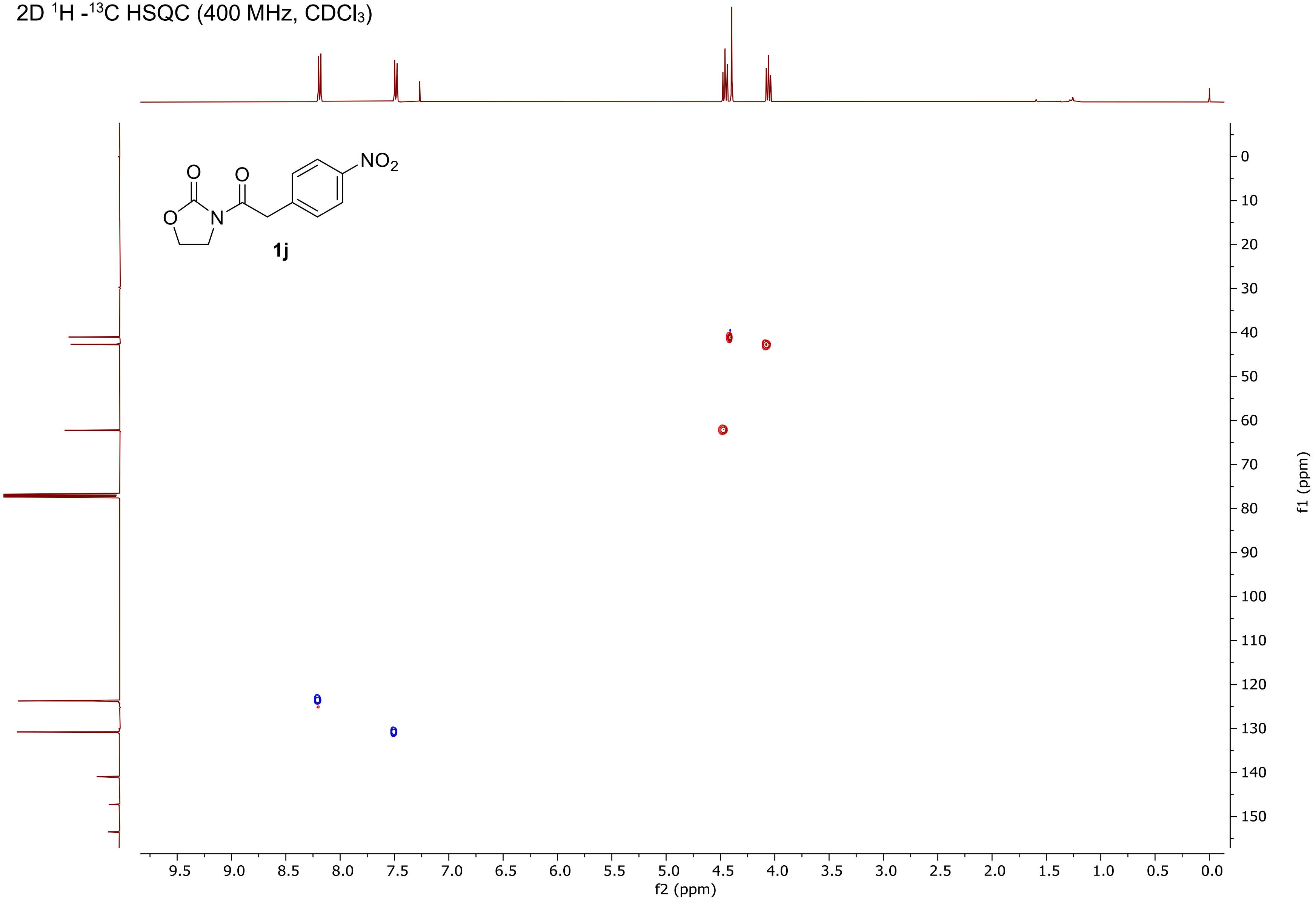

$^{1}H$ NMR (400 MHz, DMSO-$d_6$)

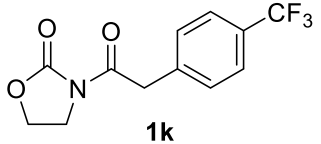


**1k**

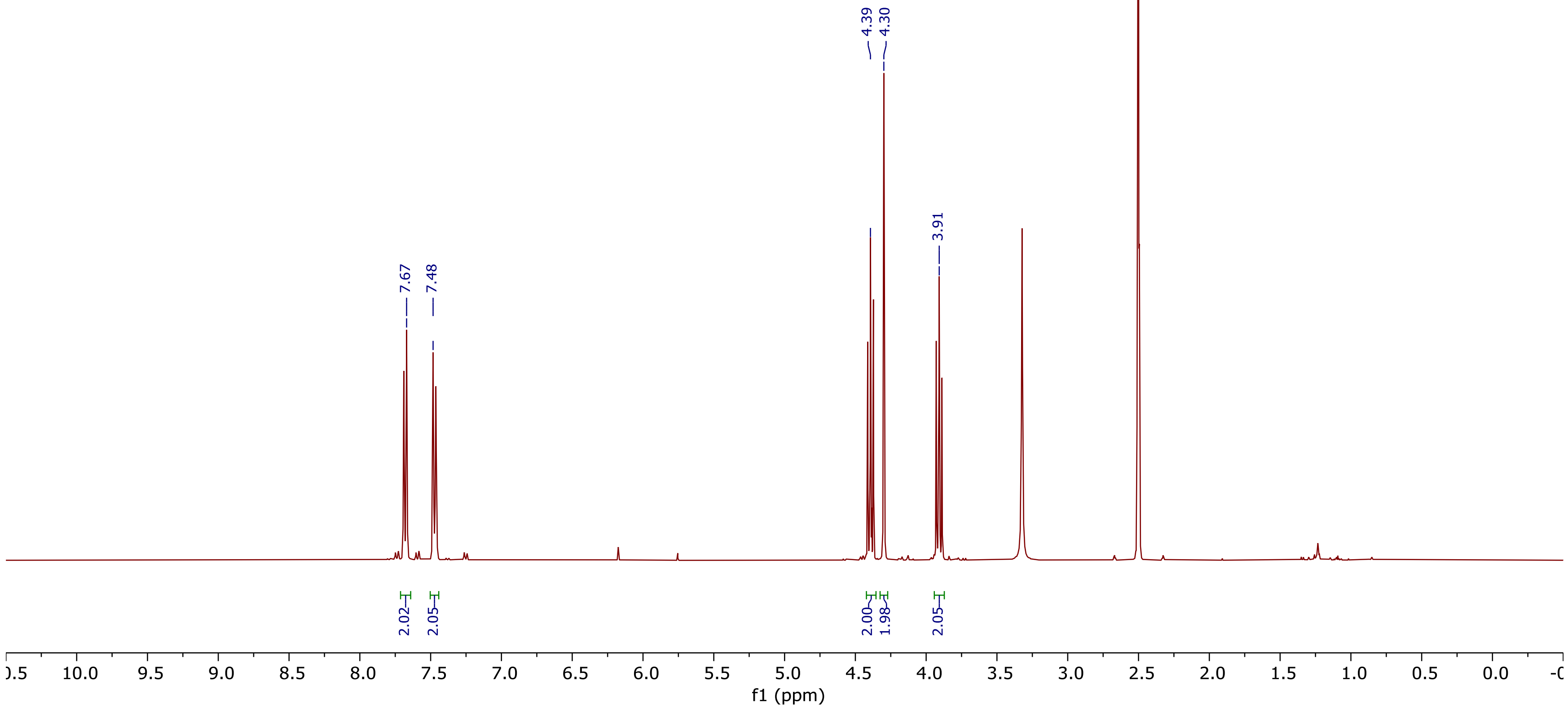

$^{13}C\{^{1}H\}$ NMR (101 MHz, DMSO-$d_6$)

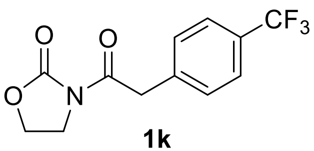

**1k**

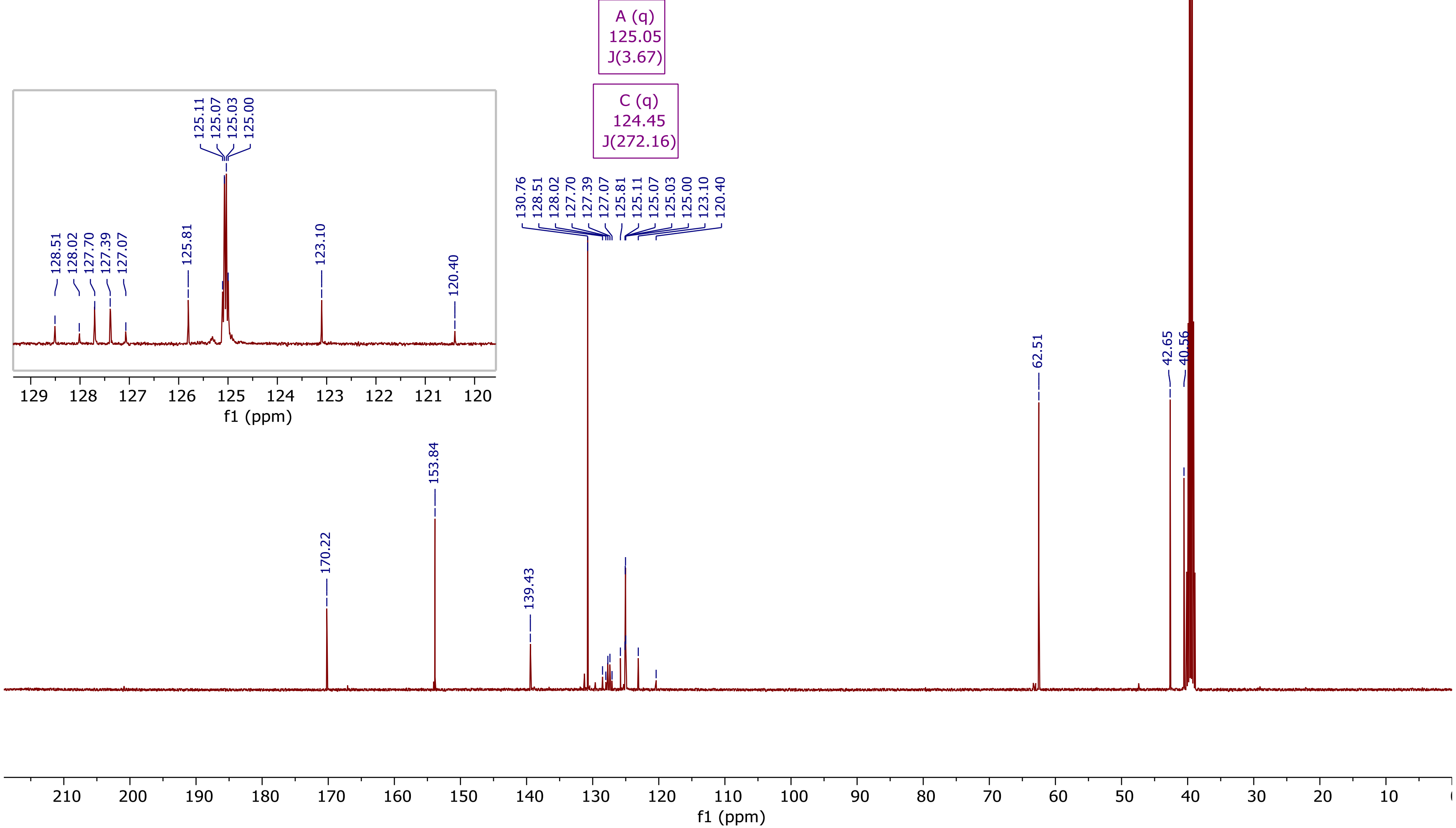

2D $^1H$ -$^1H$ COSY (400 MHz, DMSO-$d_6$)

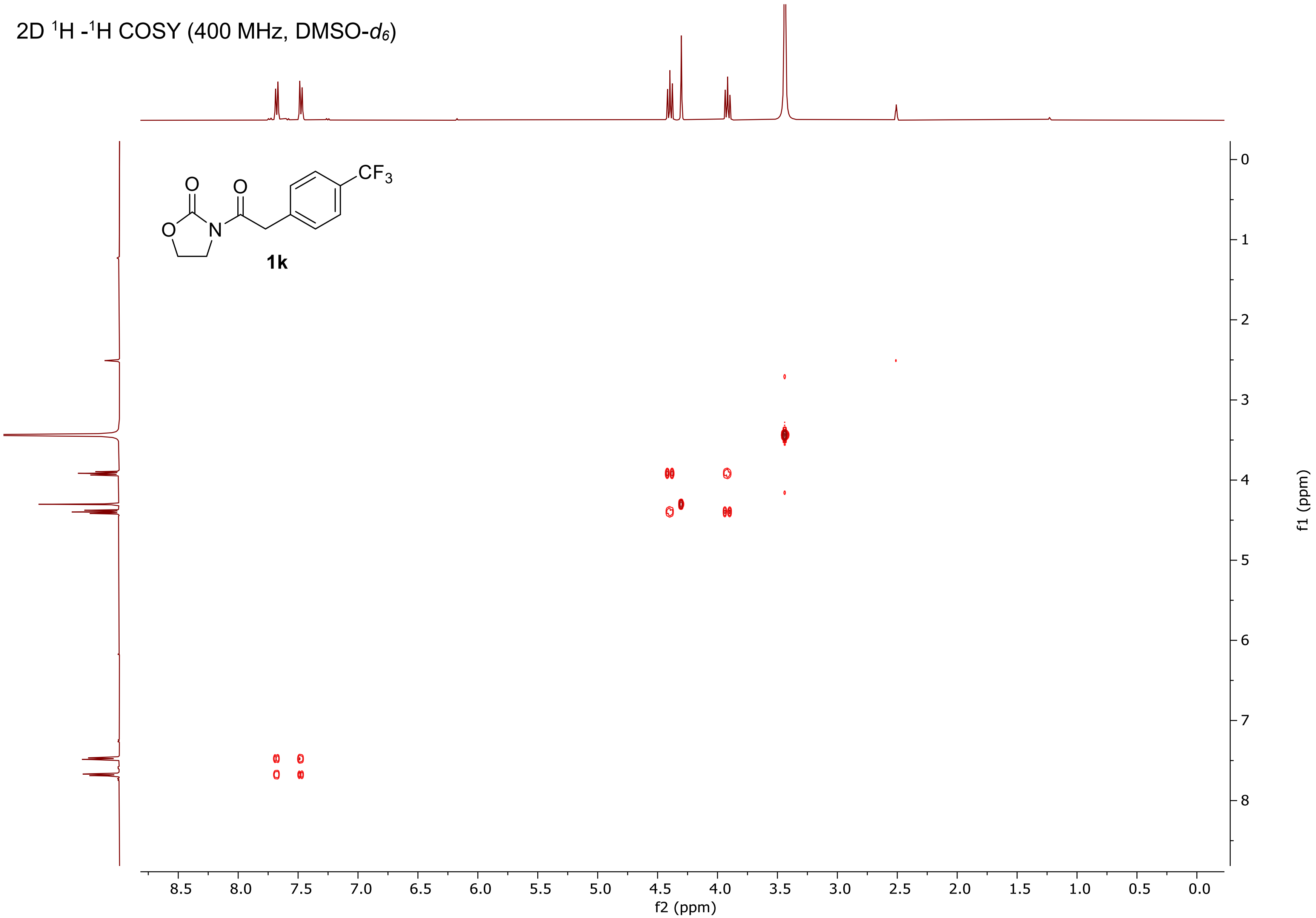

2D $^{1}H$ -$^{13}C$ HSQC (400 MHz, DMSO-$d_6$)

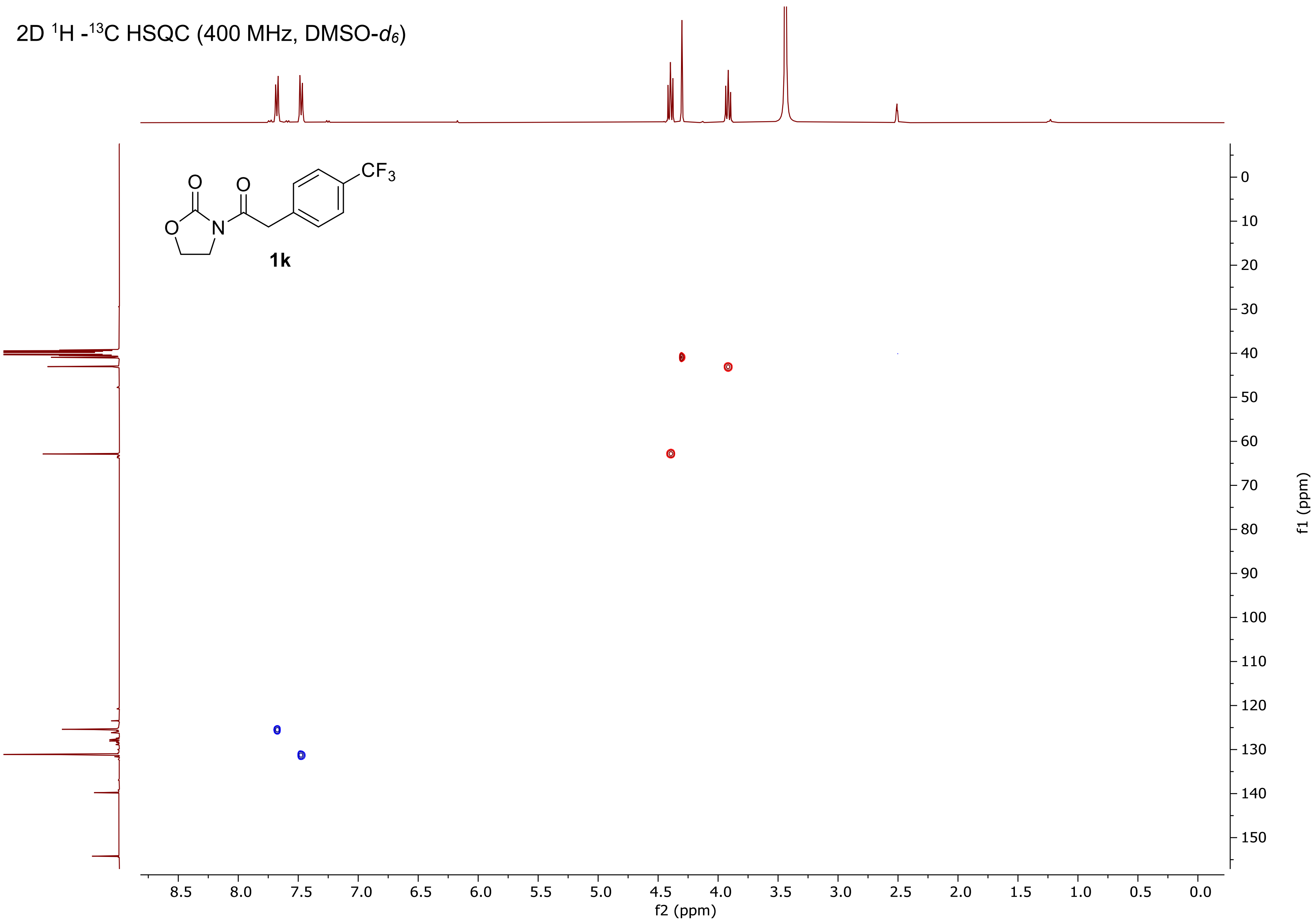

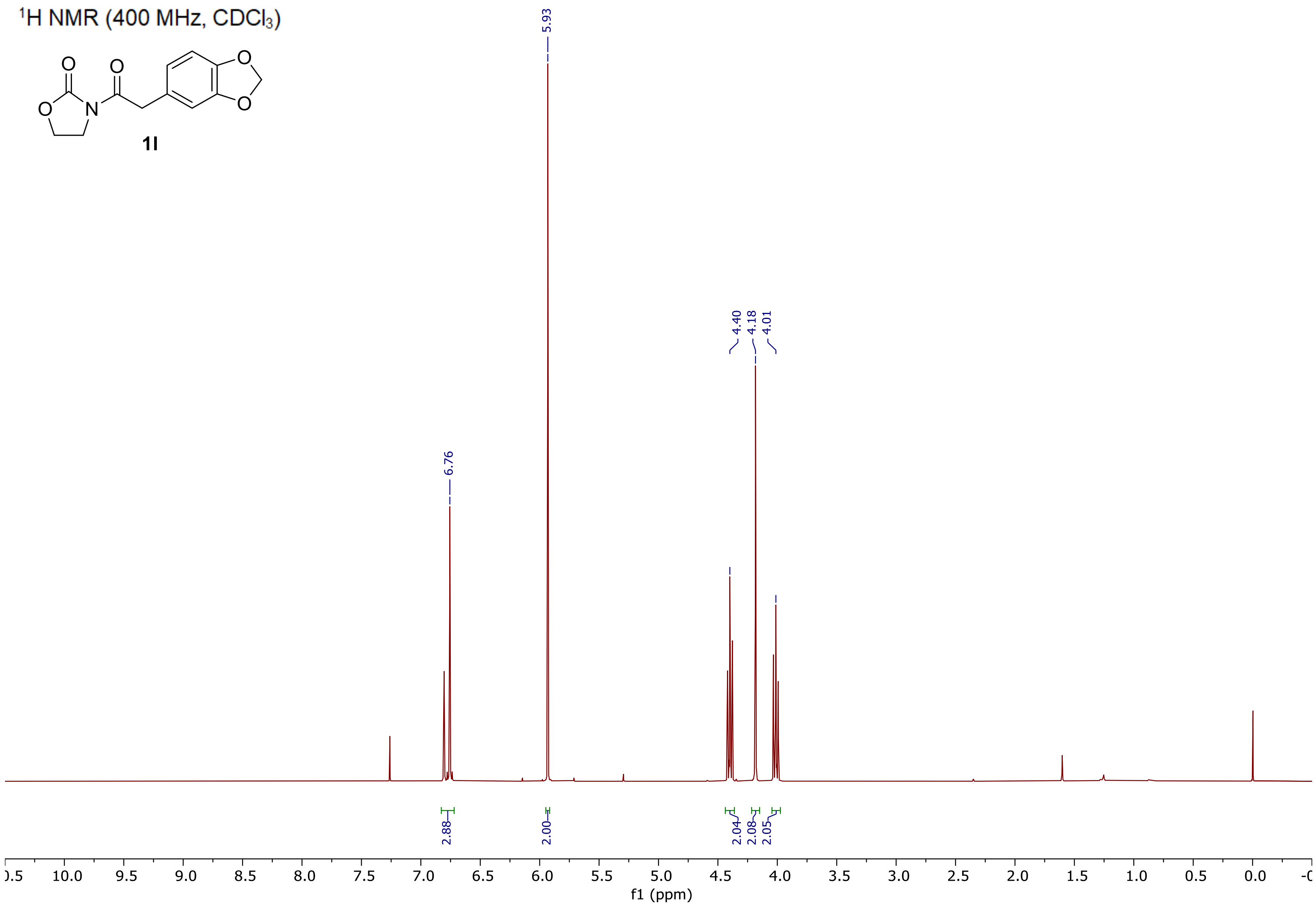

1H NMR (400 MHz, CDCl3)
1l
5.93
6.76
4.40
4.18
4.01
2.88
2.00
2.04
2.08
2.05
f1 (ppm)

$^{13}C\{^{1}H\}$ NMR (101 MHz, $CDCl_3$)

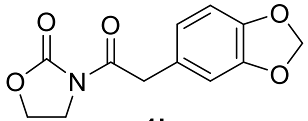

**1l**

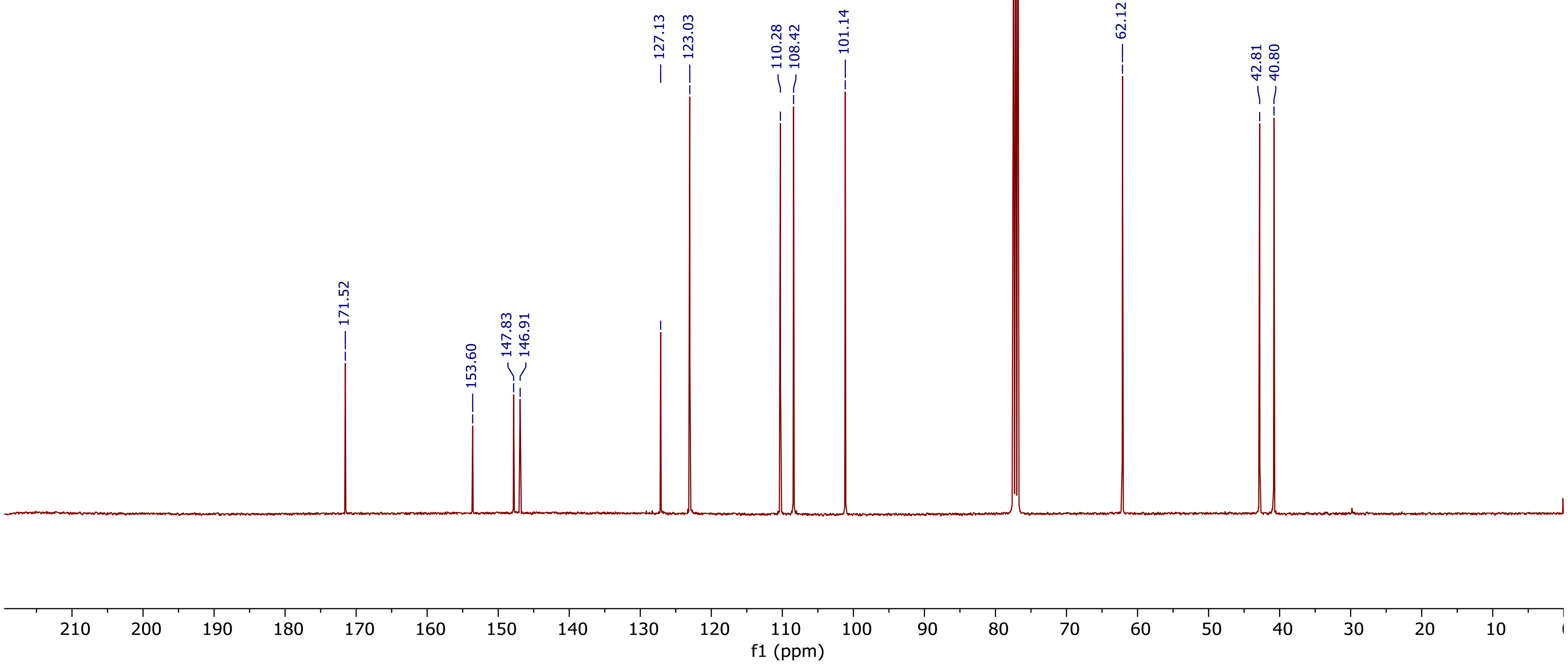

2D $^{1}H$ -$^{1}H$ COSY (400 MHz, $CDCl_3$)

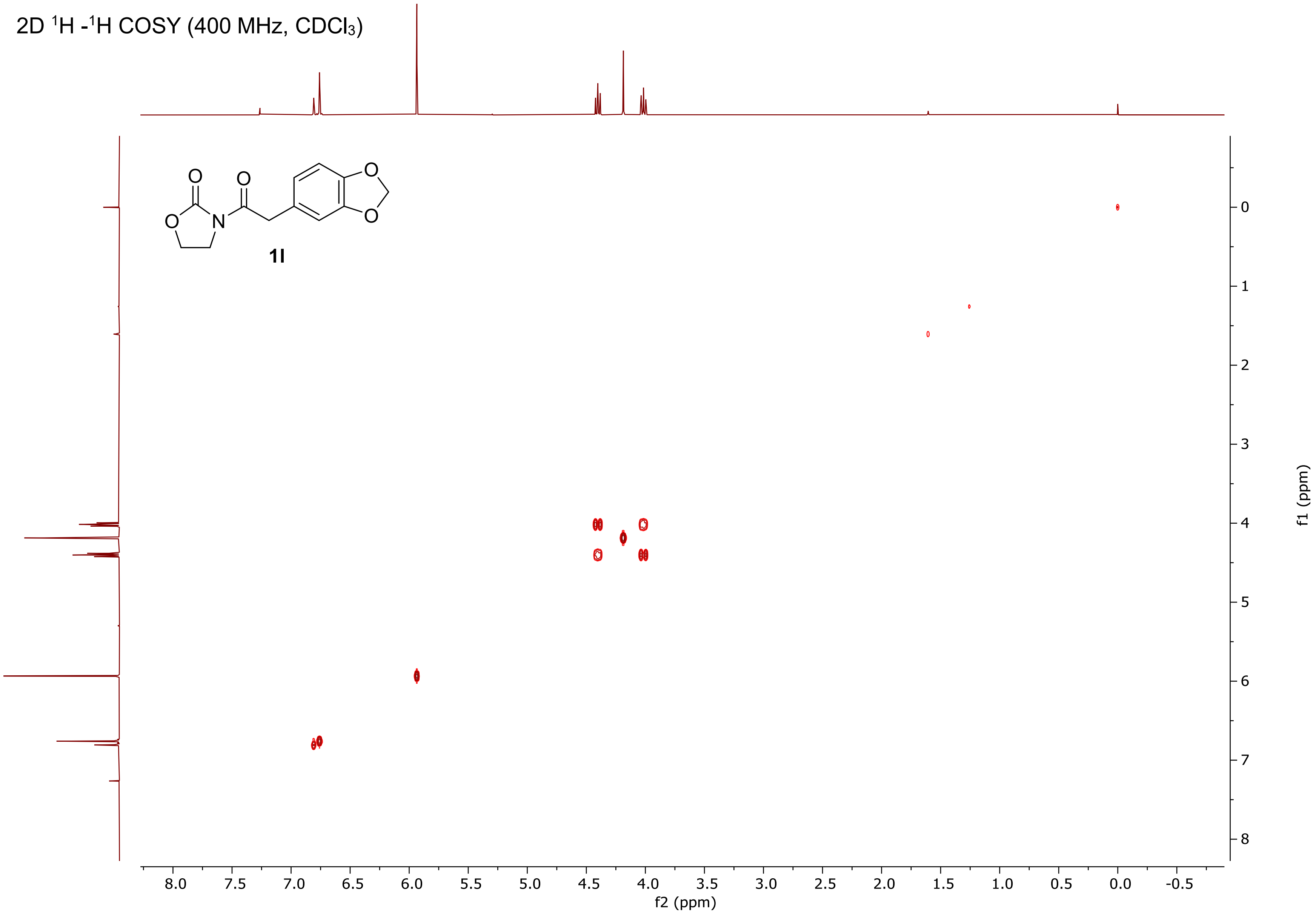

2D $^{1}$H -$^{13}$C HSQC (400 MHz, $CDCl_3$)

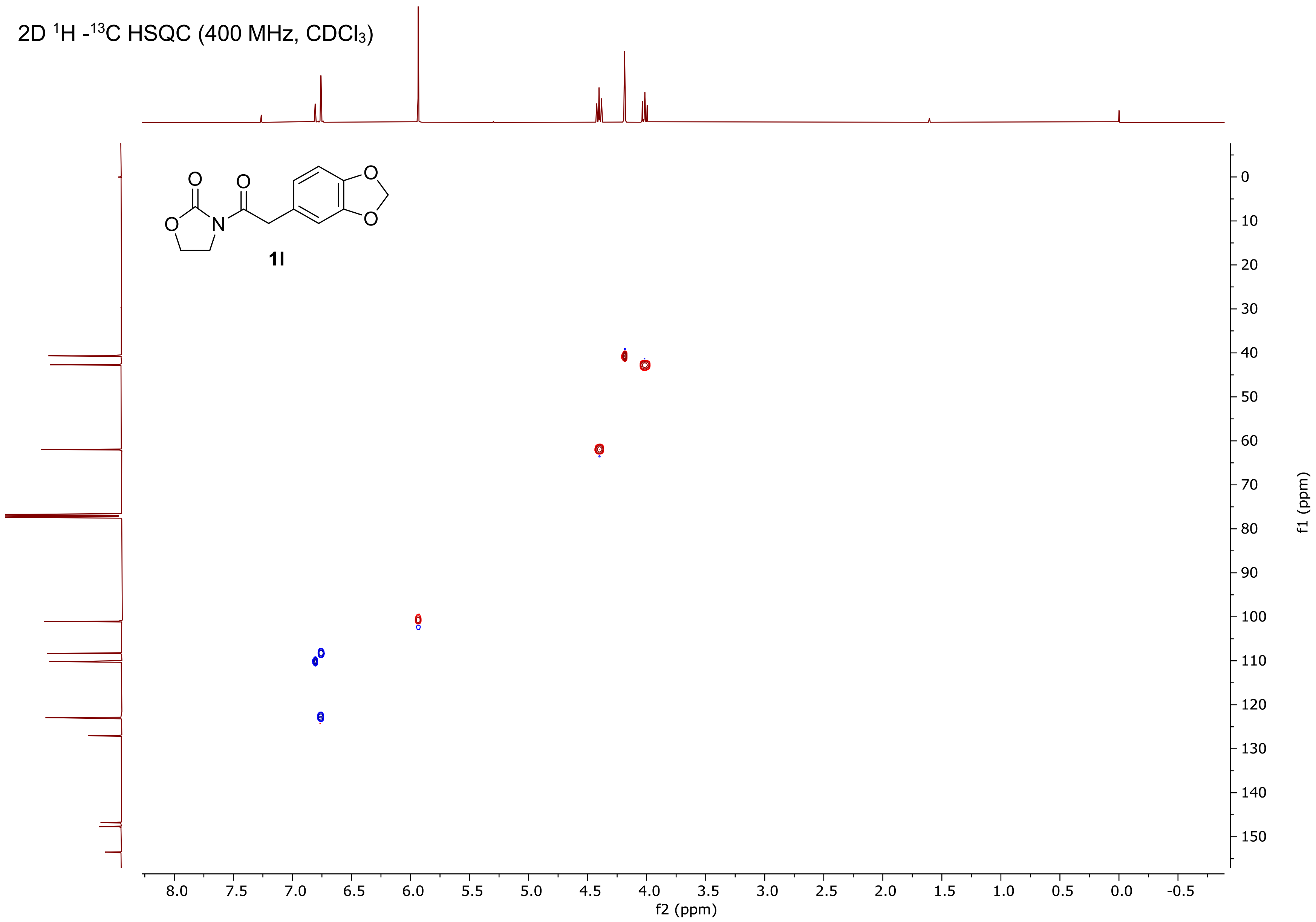

$^{1}H$ NMR (400 MHz, $CDCl_3$)

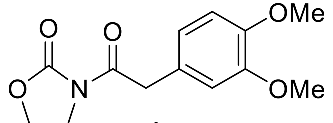


**1m**

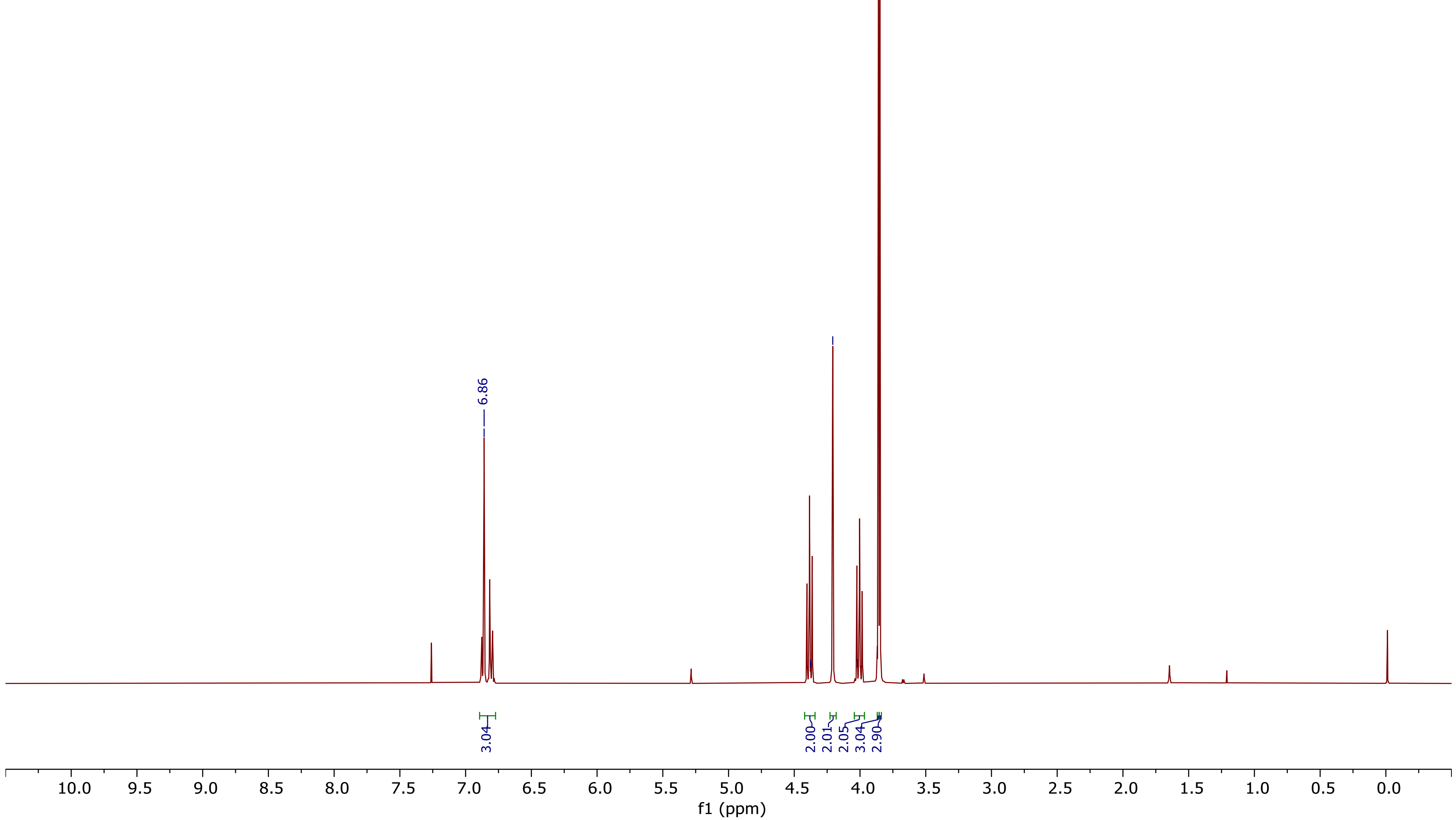

$^{13}C\{^1H\}$ NMR (101 MHz, $CDCl_3$)

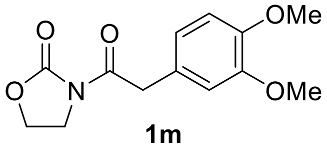


**1m**

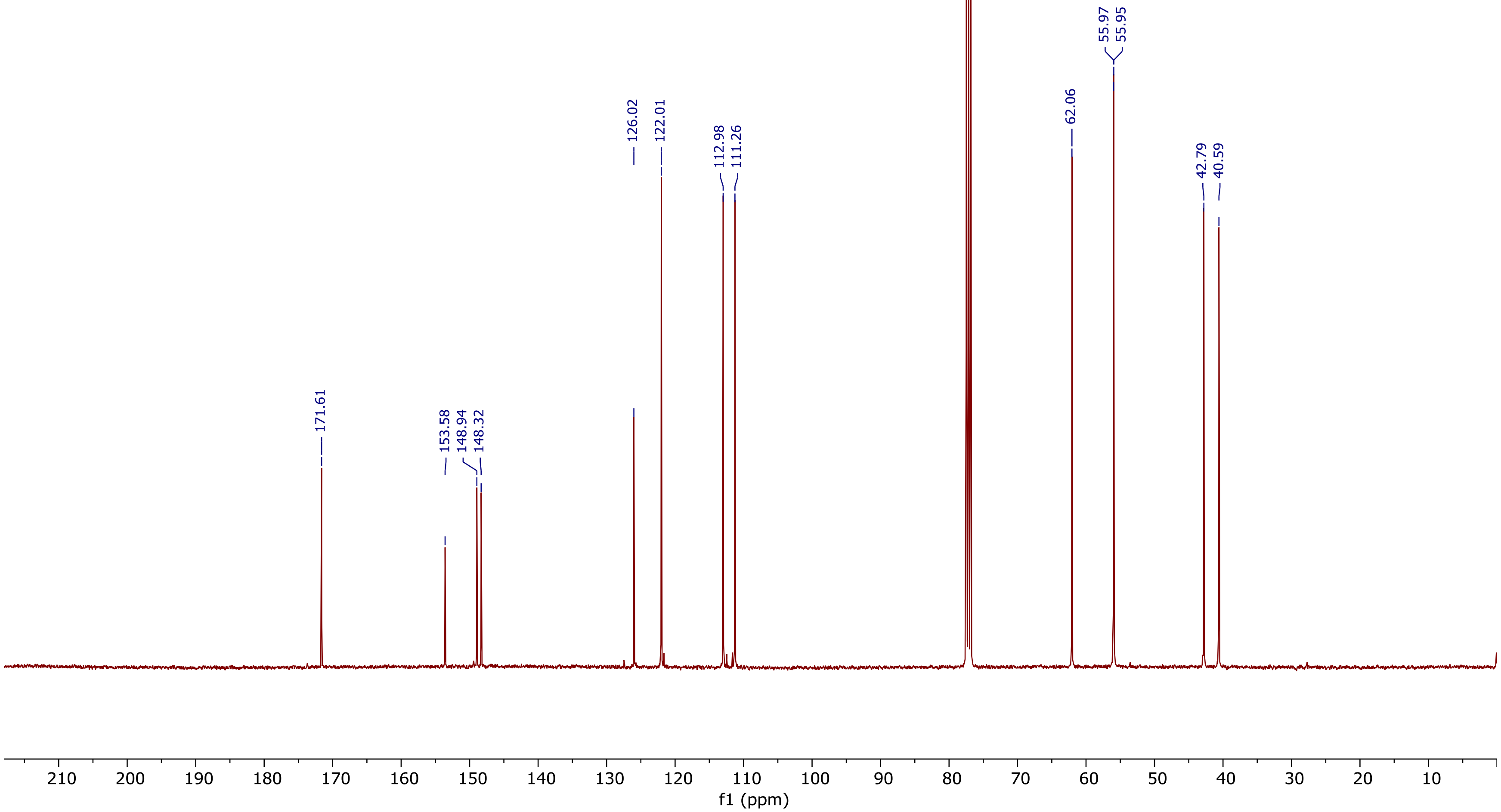

2D $^1H$ -$^1H$ COSY (400 MHz, $CDCl_3$)

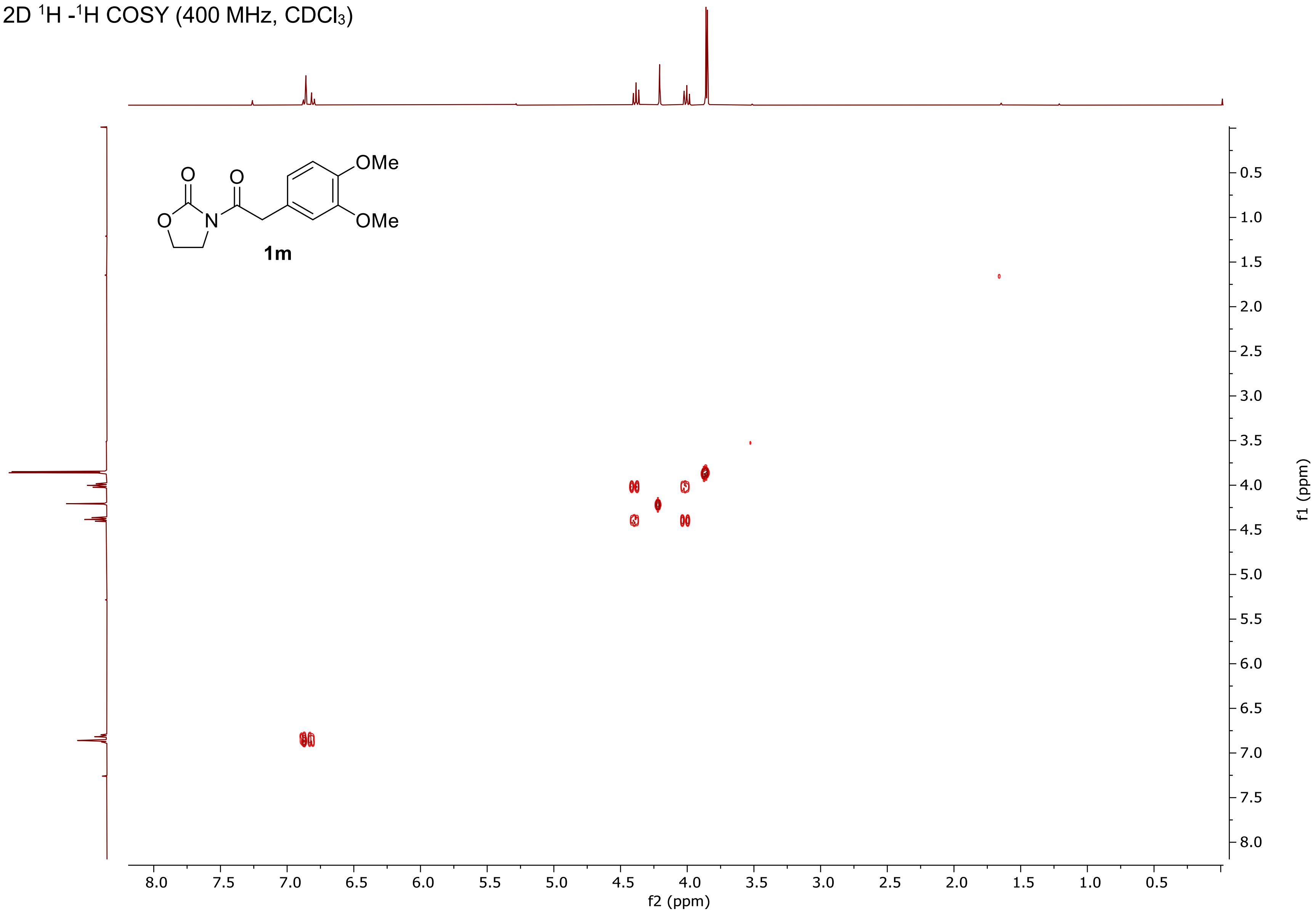

2D $^1H$ -$^{13}C$ HSQC (400 MHz, $CDCl_3$)

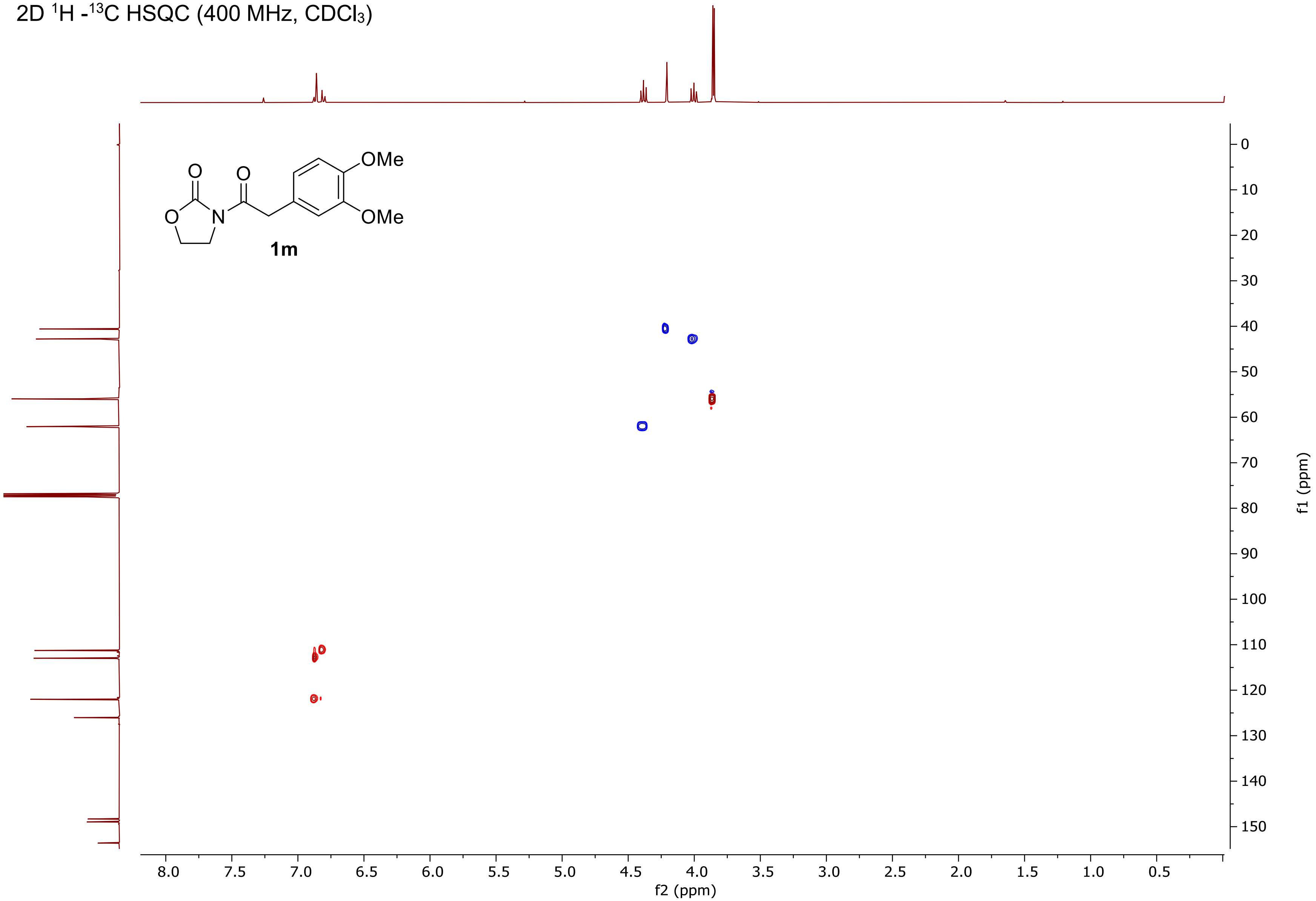

[1]H NMR (400 MHz, DMSO-$d_6$)

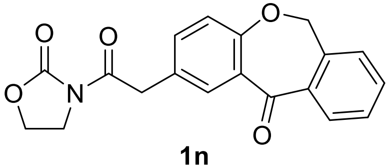

**1n**

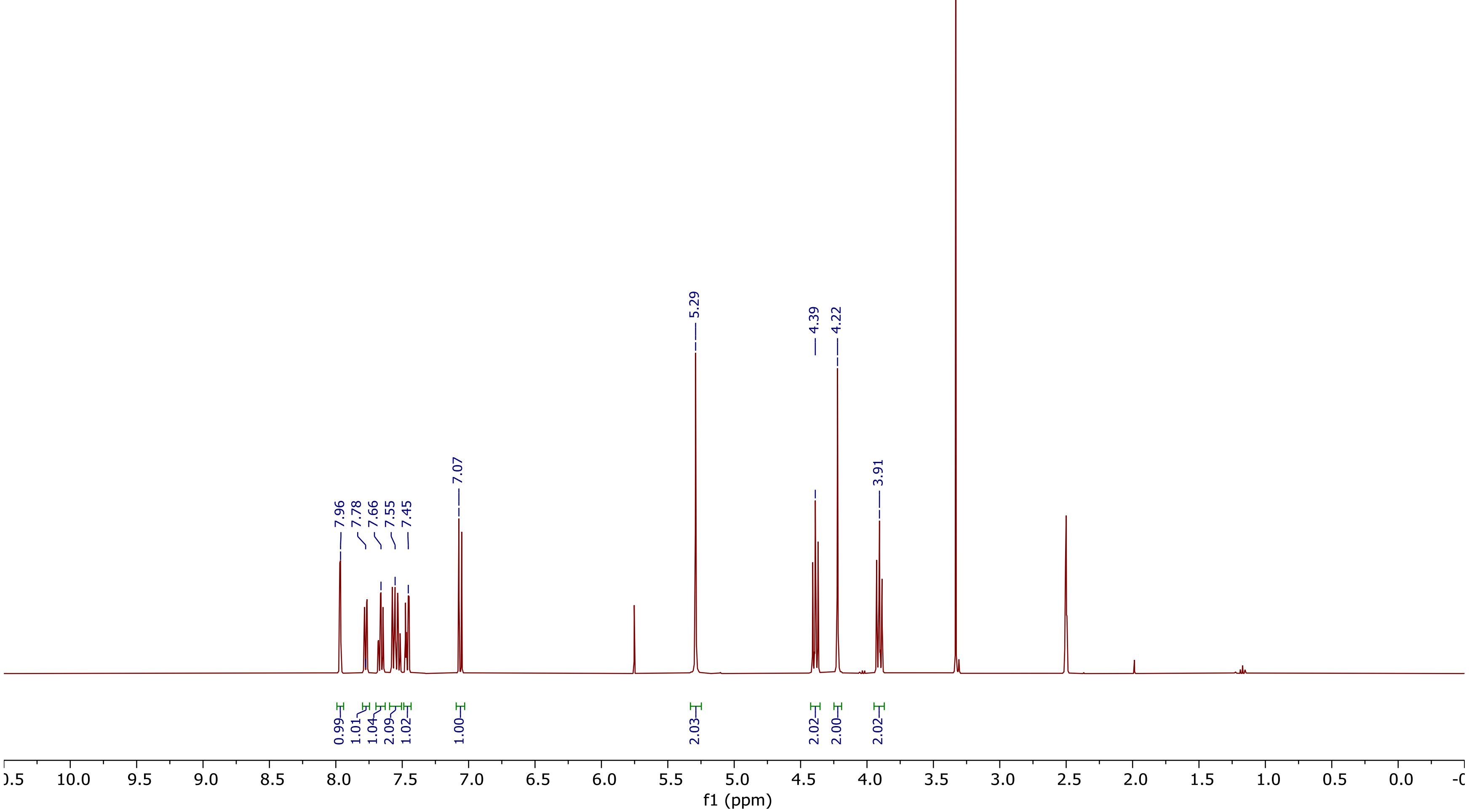

$^{13}C\{^{1}H\}$ NMR (101 MHz, DMSO-$d_6$)

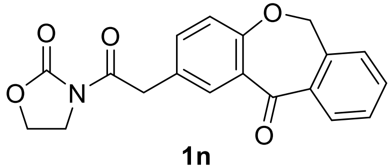

**1n**

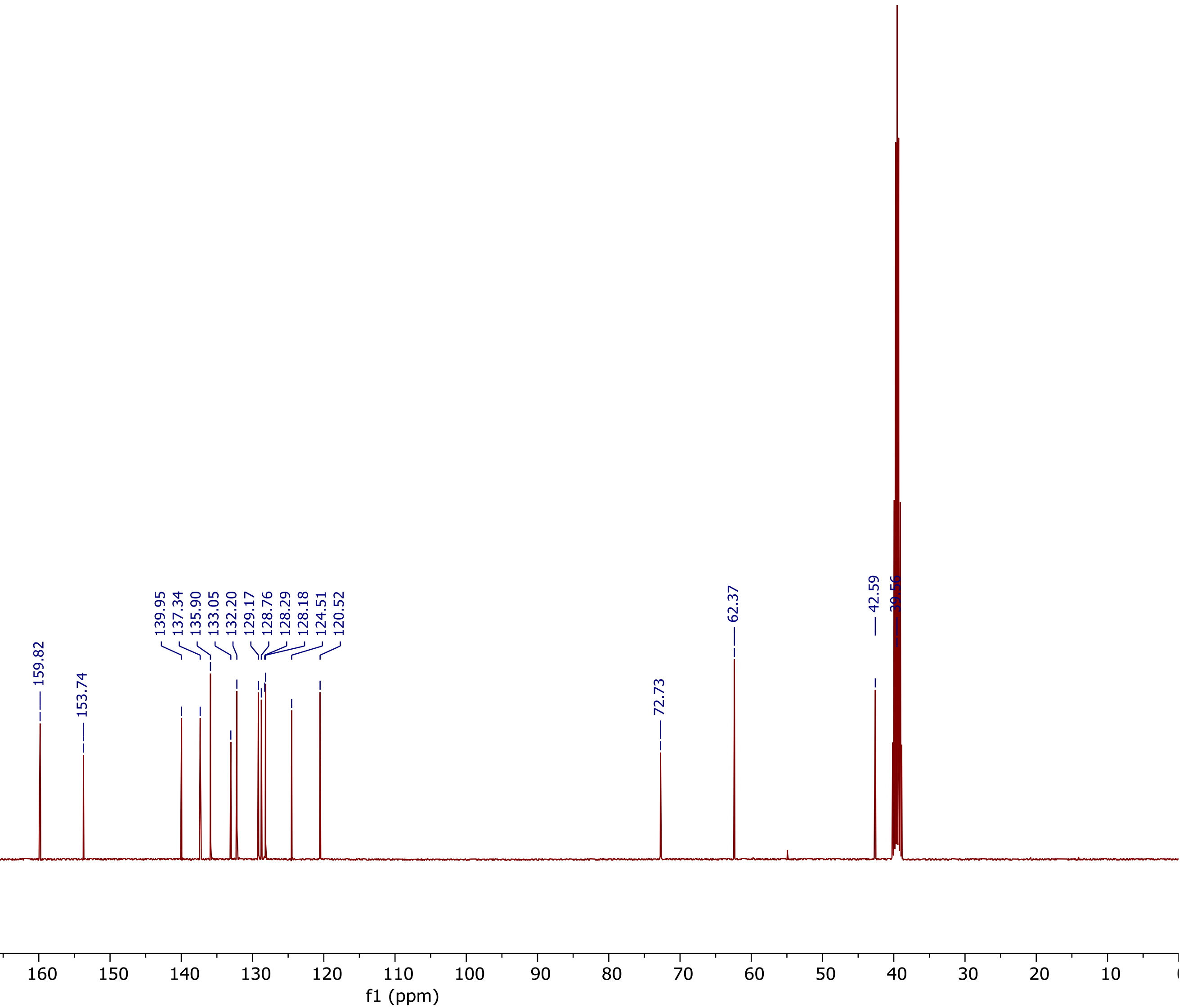

2D $^1H$ -$^1H$ COSY (400 MHz, DMSO-$d_6$)

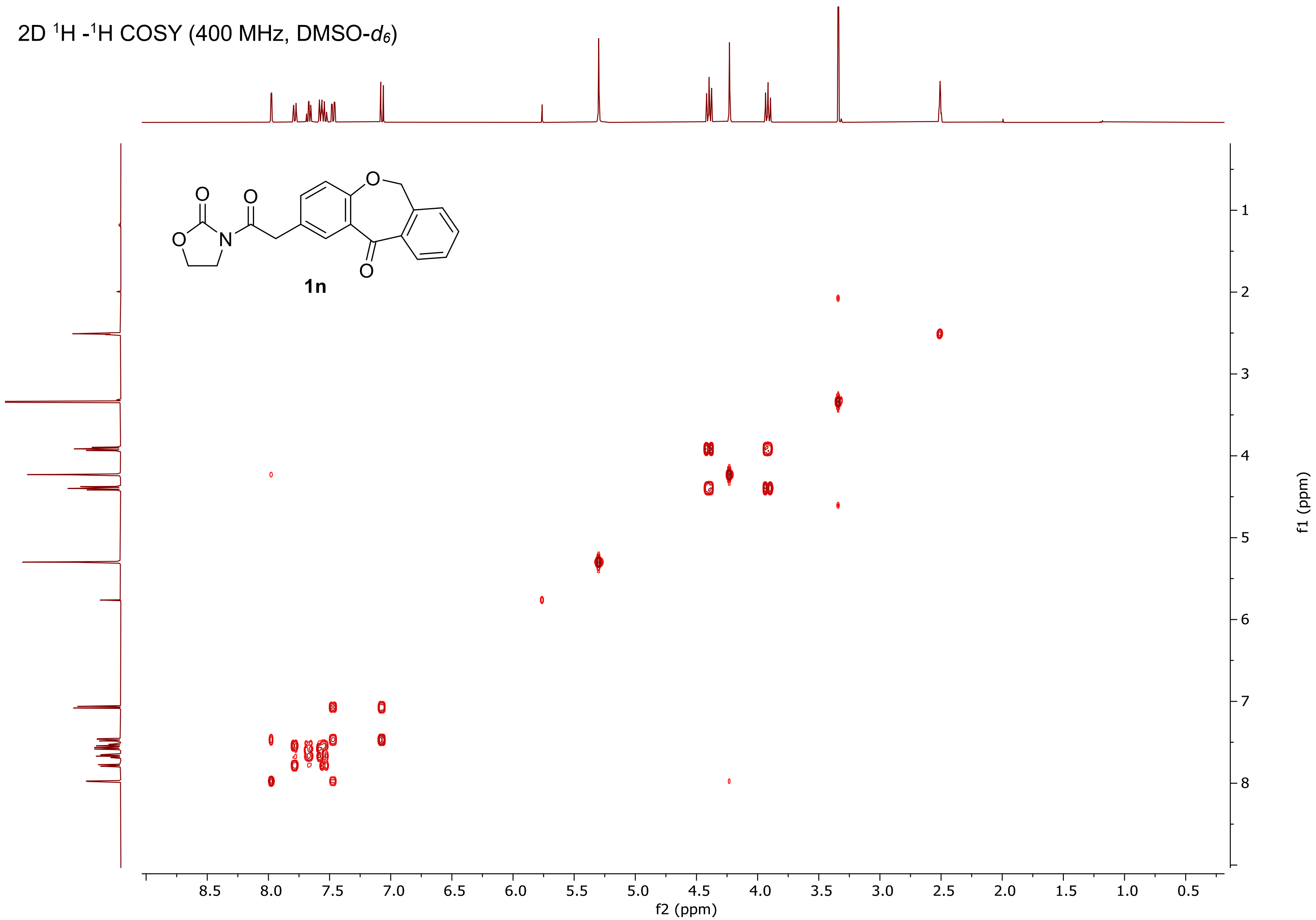

2D $^1$H -$^{13}$C HSQC (400 MHz, DMSO-$d_6$)

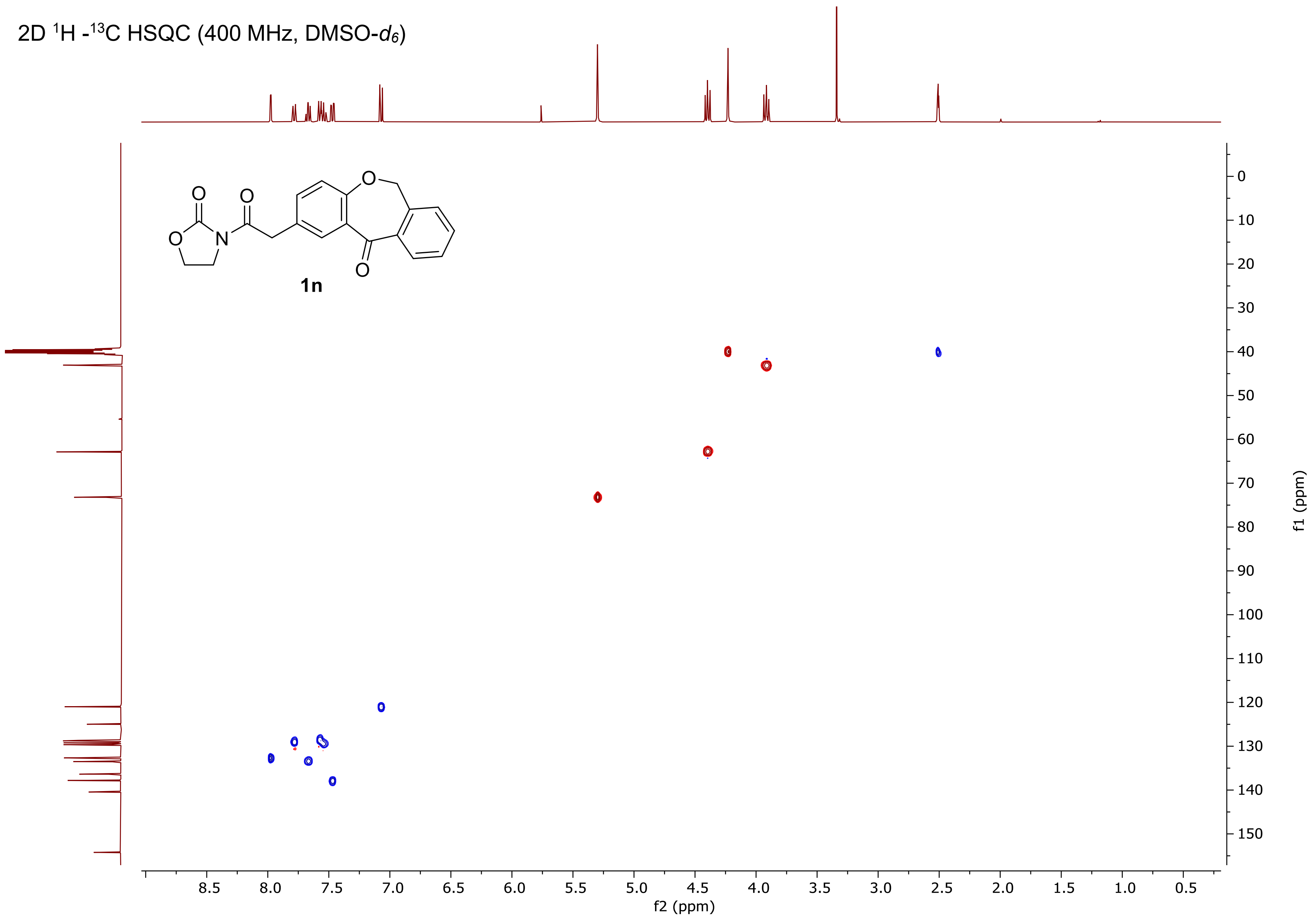

$^1H$ NMR (400 MHz, $CDCl_3$)

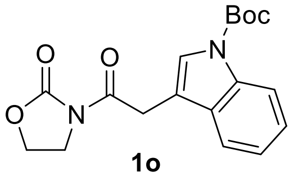


**1o**

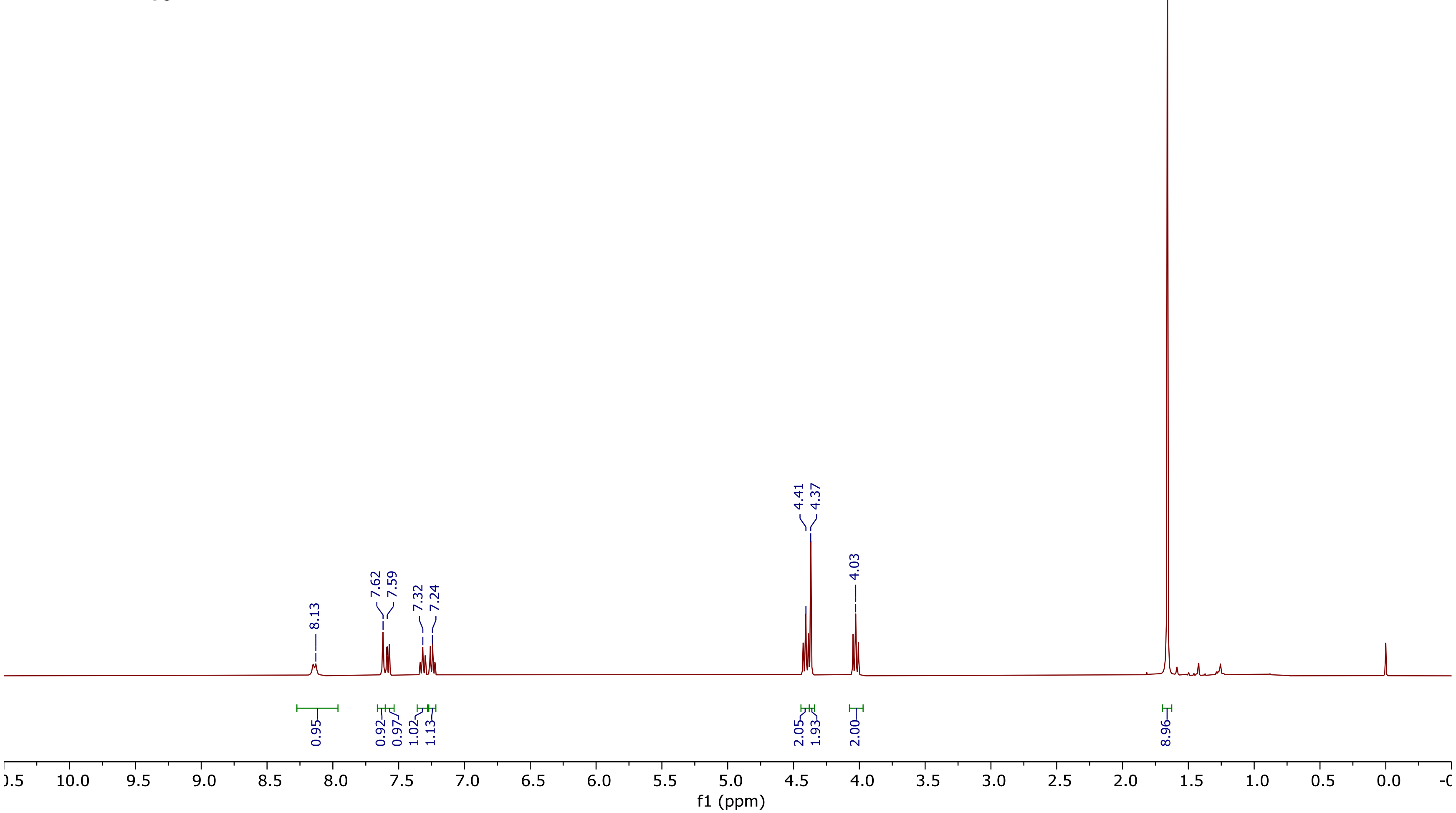

$^{13}C\{^{1}H\}$ NMR (101 MHz, $CDCl_3$)

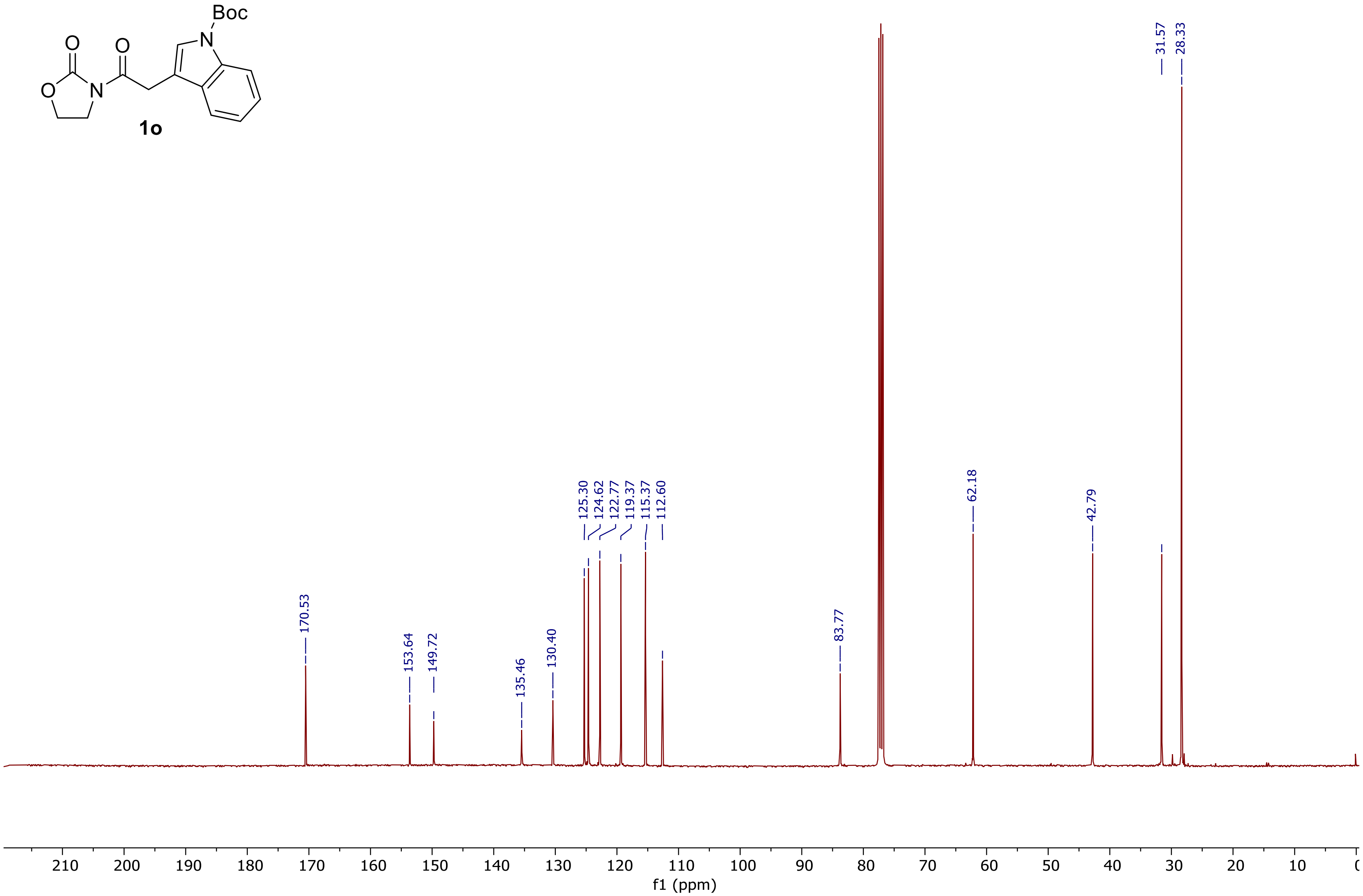

2D $^1H$ -$^1H$ COSY (400 MHz, $CDCl_3$)

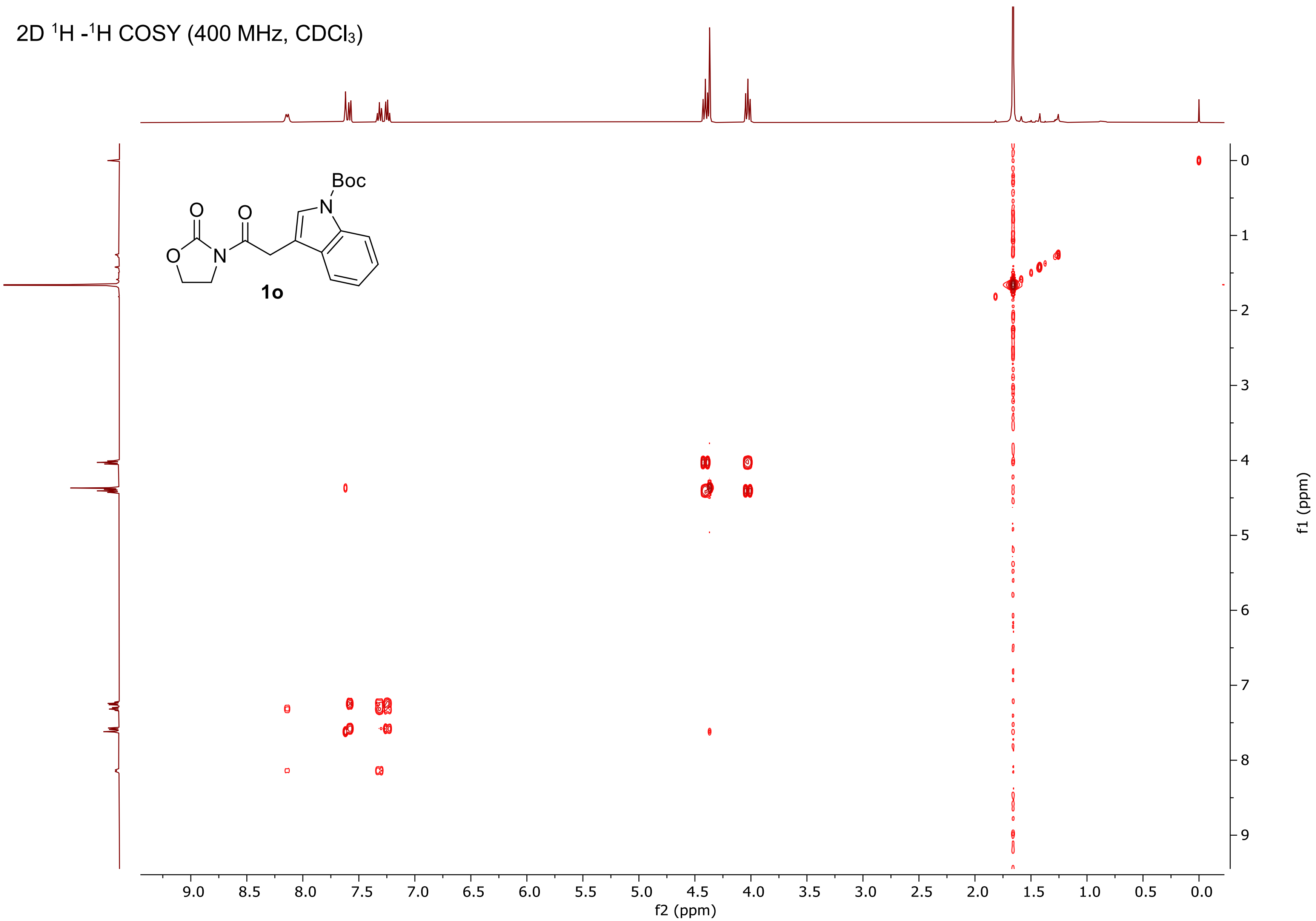

2D $^{1}$H -$^{13}$C HSQC (400 MHz, $CDCl_3$)

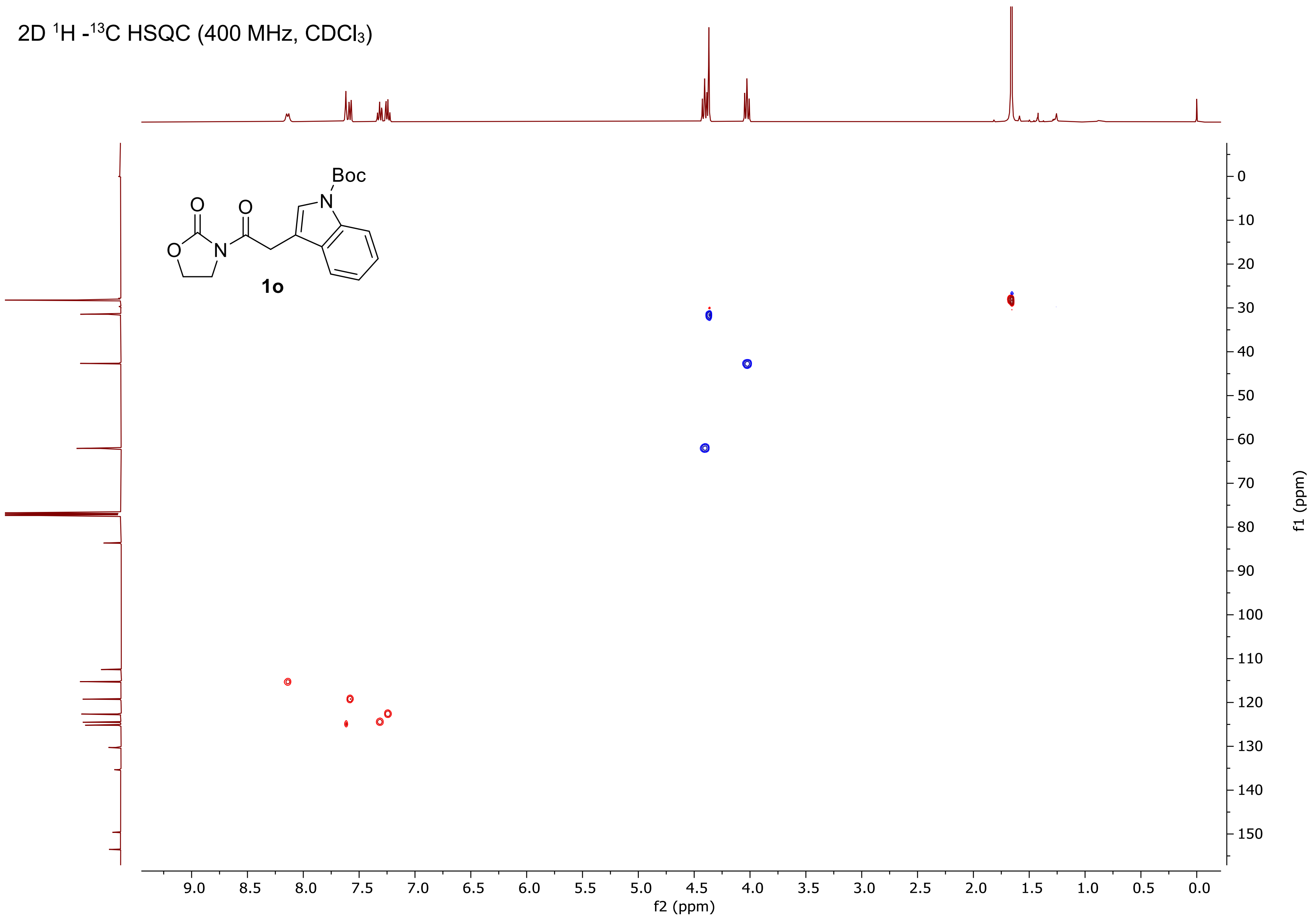

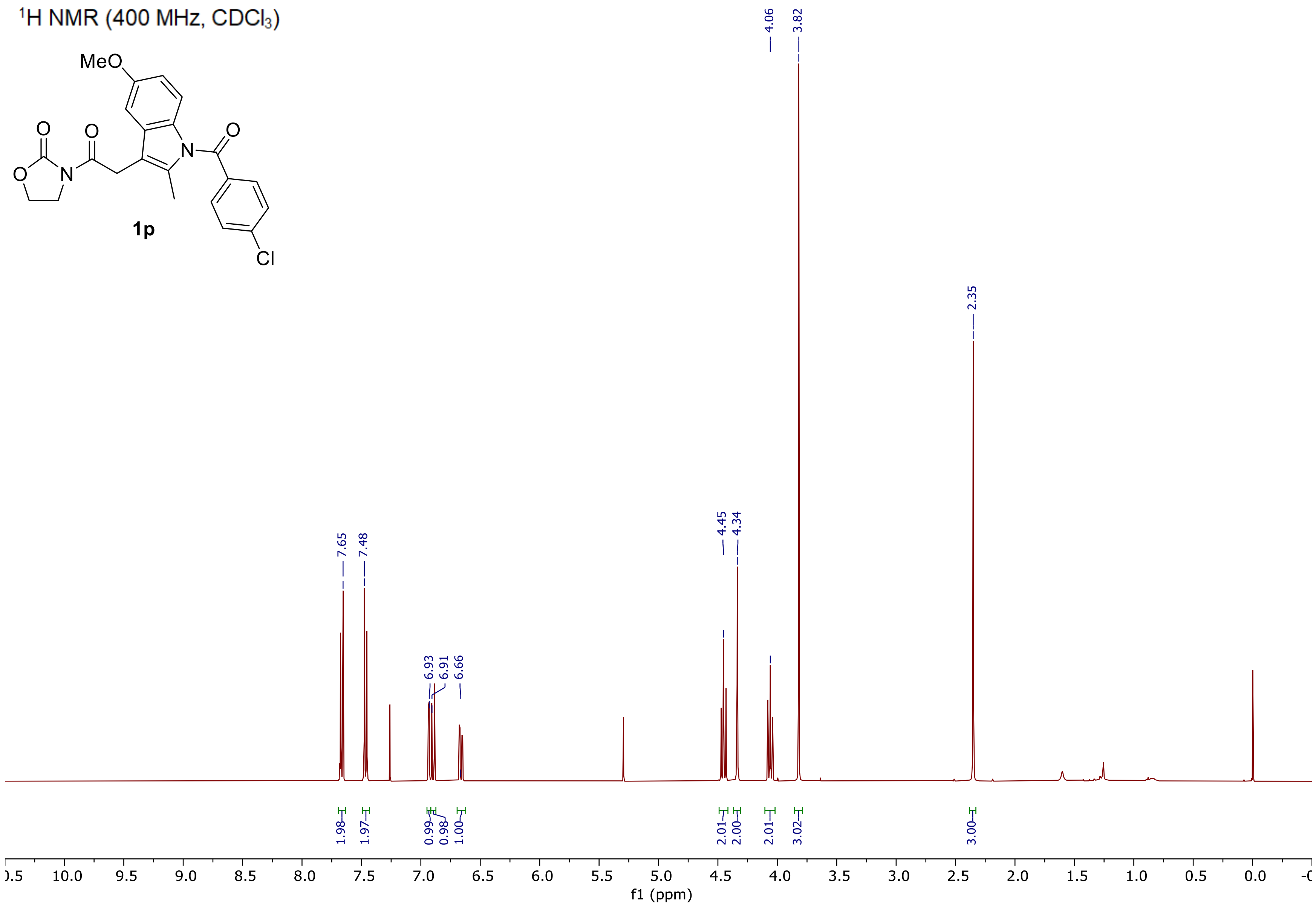

¹H NMR (400 MHz, CDCl₃)
MeO
O
N
Cl
1p
7.65
7.48
6.93
6.91
6.66
4.45
4.34
4.06
3.82
2.35
1.98
1.97
0.99
0.98
1.00
2.01
2.00
2.01
3.02
3.00
10.0
9.5
9.0
8.5
8.0
7.5
7.0
6.5
6.0
5.5
5.0
4.5
4.0
3.5
3.0
2.5
2.0
1.5
1.0
0.5
0.0
f1 (ppm)

$^{13}C\{^{1}H\}$ NMR (101 MHz, $CDCl_3$)

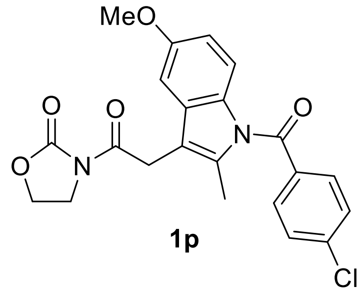


**1p**

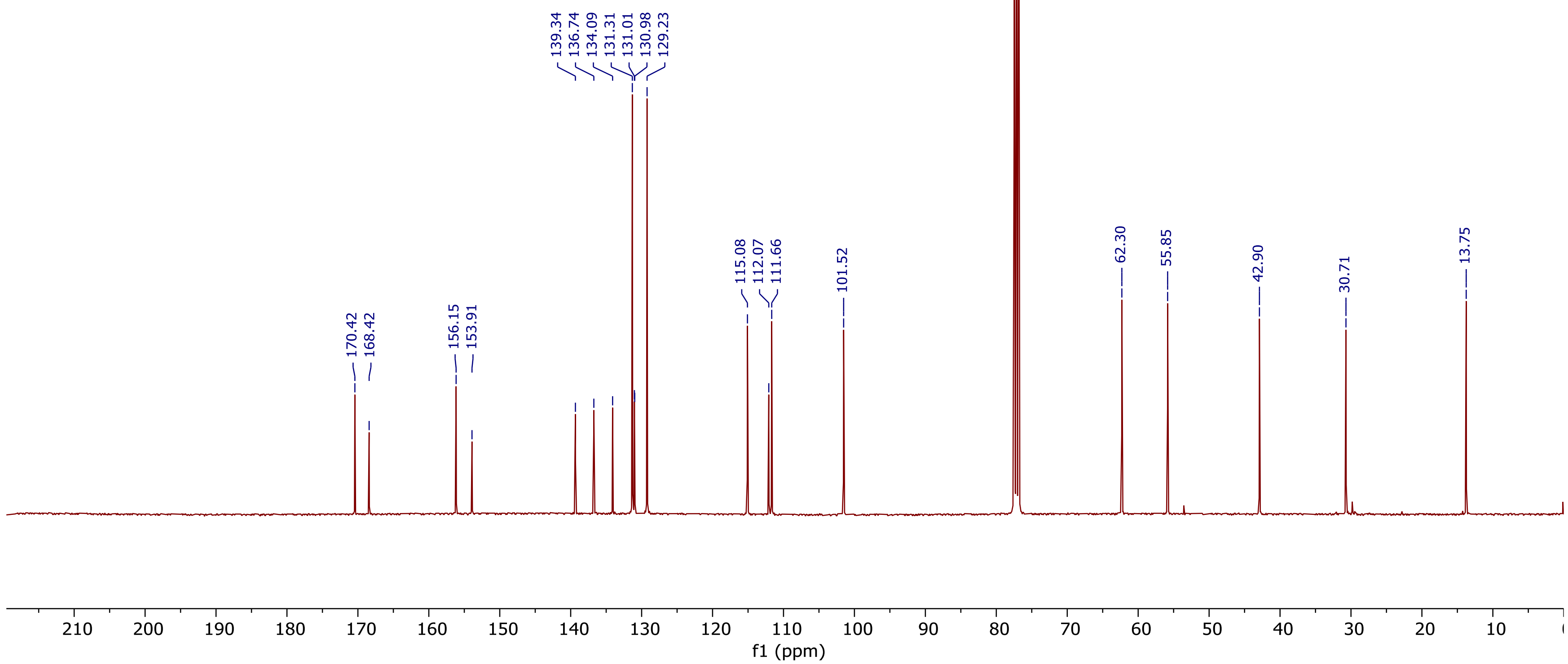

2D $^1H$ -$^1H$ COSY (400 MHz, $CDCl_3$)

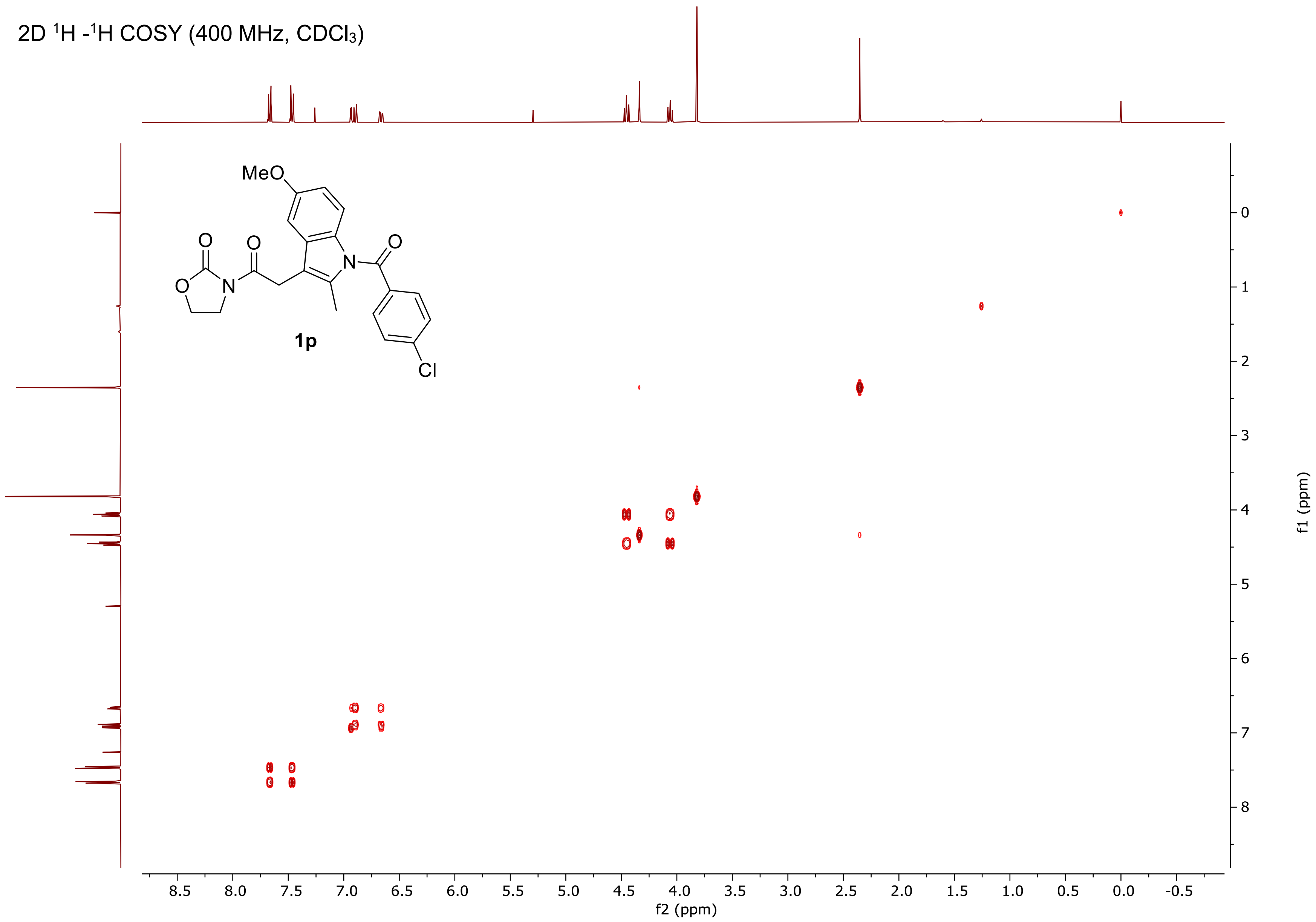

2D $^{1}H$ -$^{13}C$ HSQC (400 MHz, $CDCl_3$)

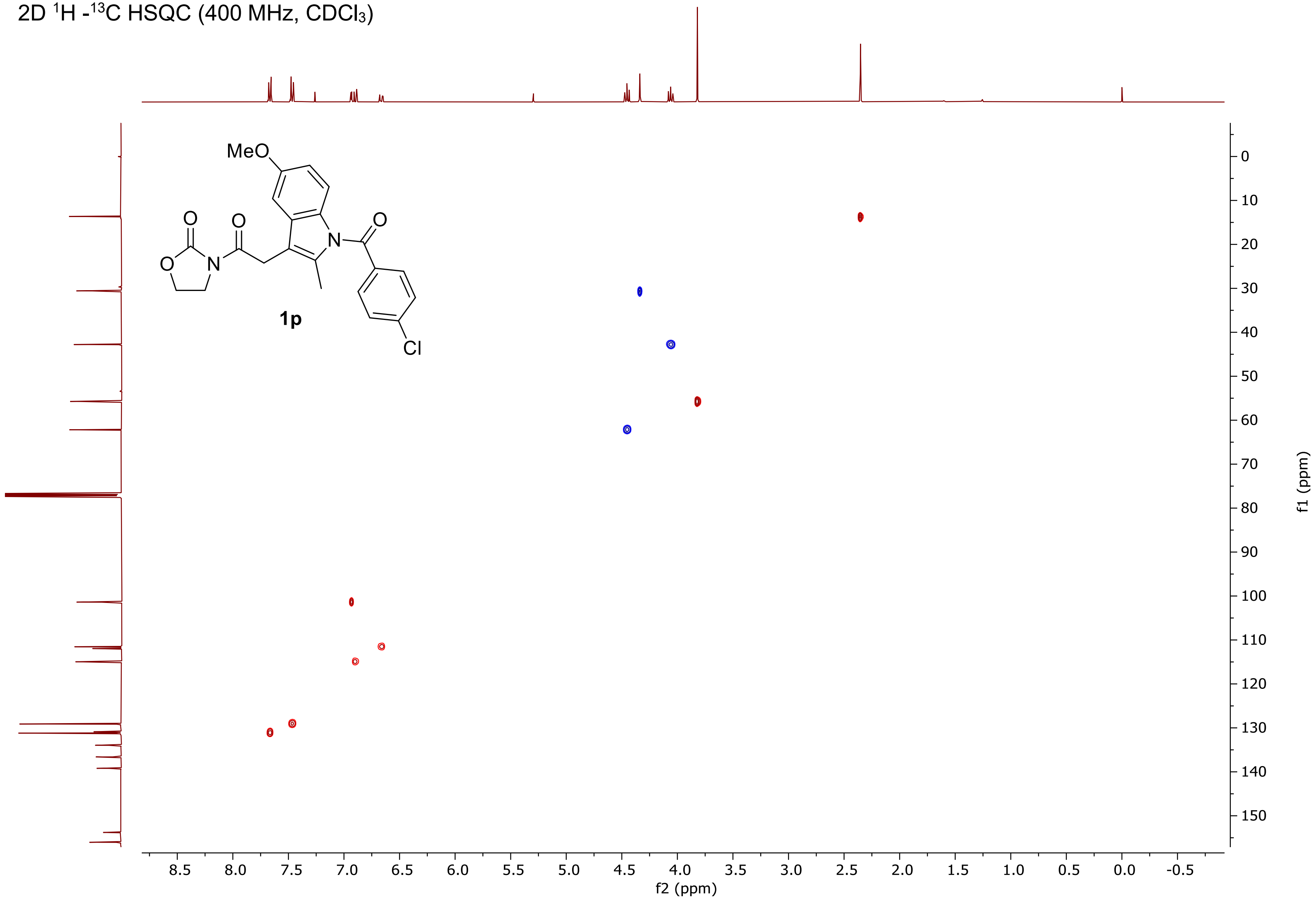

[1]H NMR (500 MHz, DMSO-$d_6$)

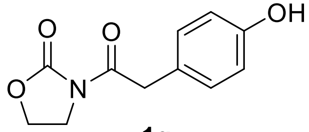


**1q**

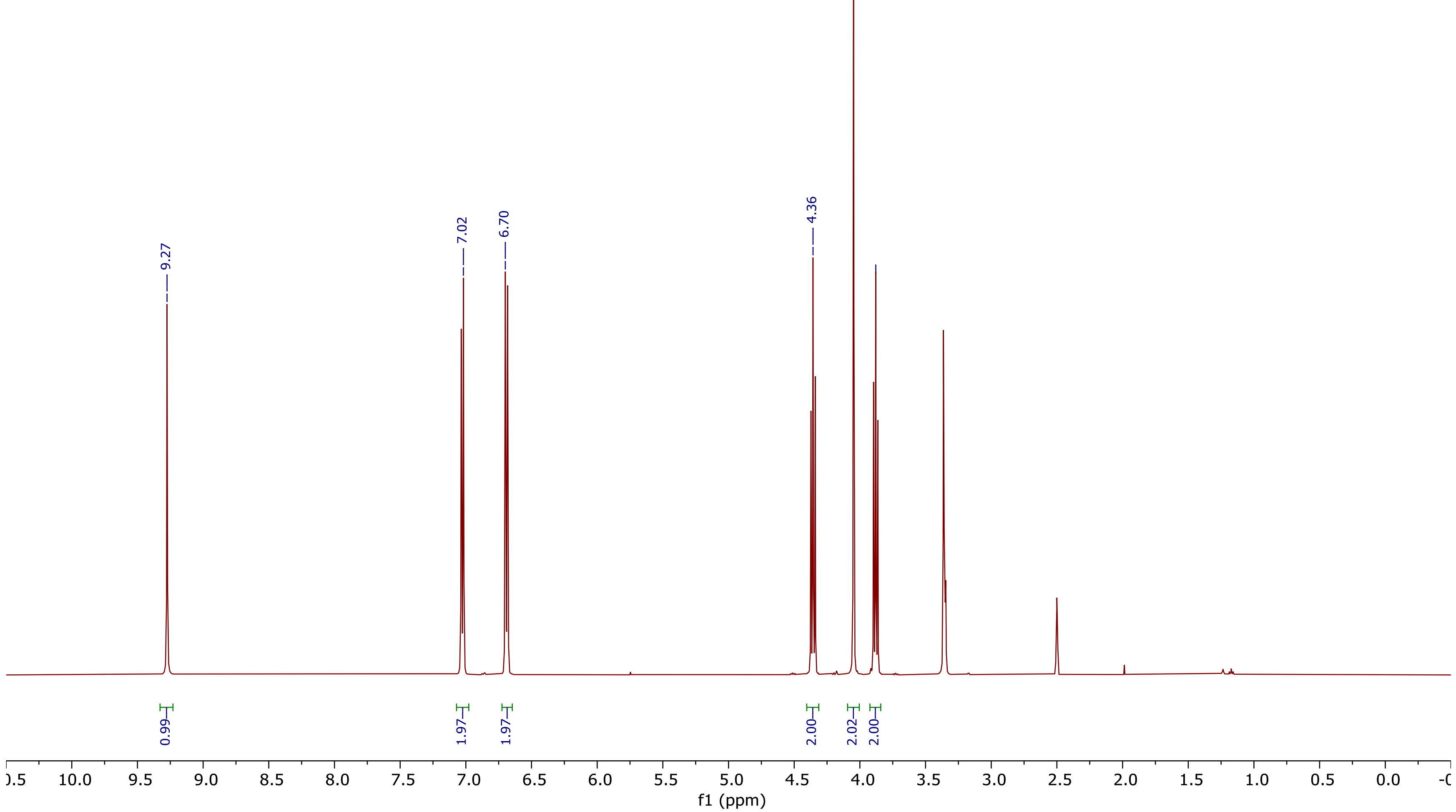

$^{13}C\{^{1}H\}$ NMR (126 MHz, DMSO-$d_6$)

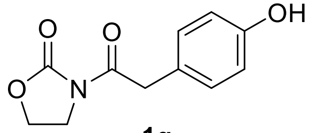


**1q**

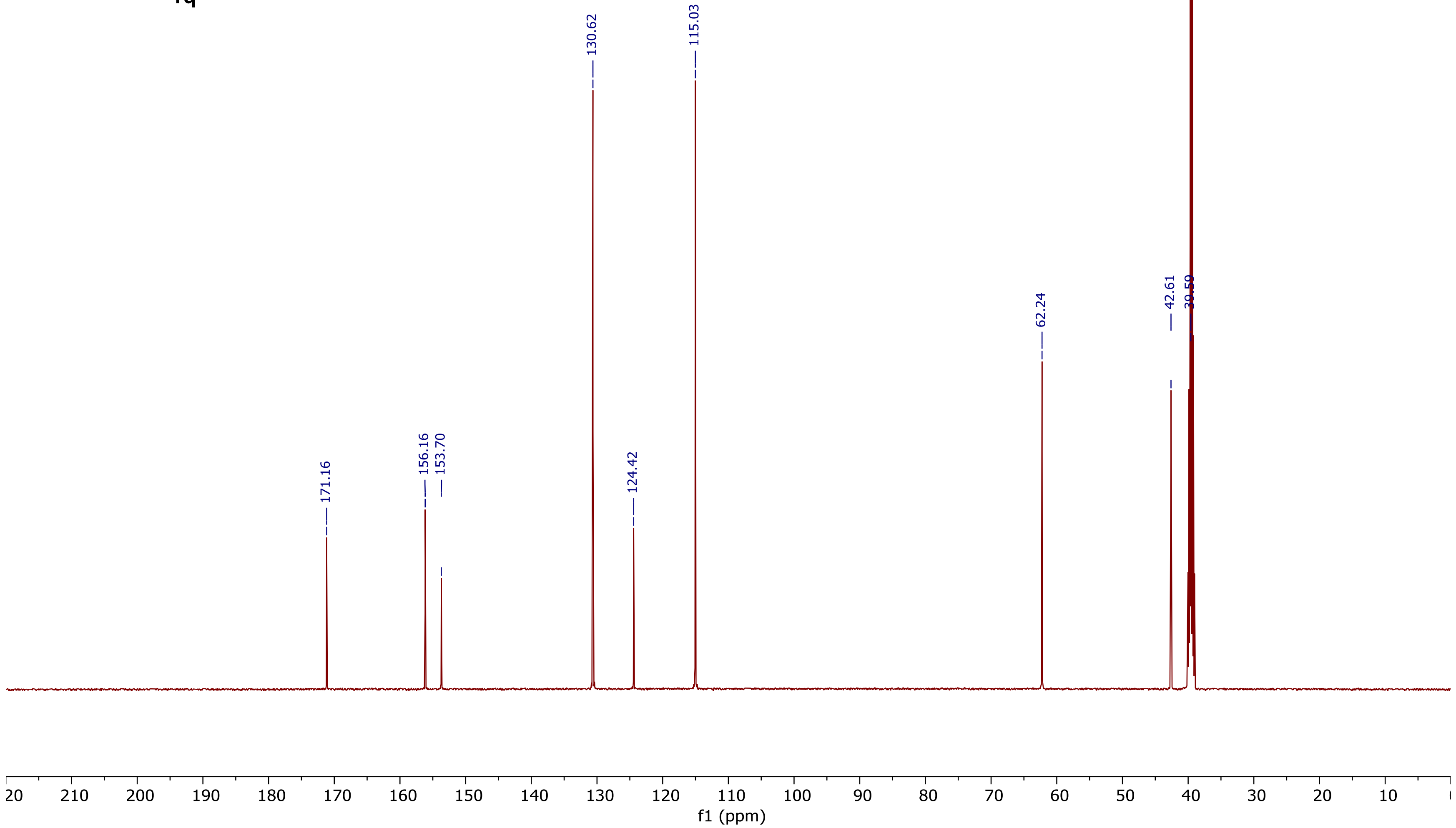

2D $^1$H -$^1$H COSY (500 MHz, DMSO-$d_6$)

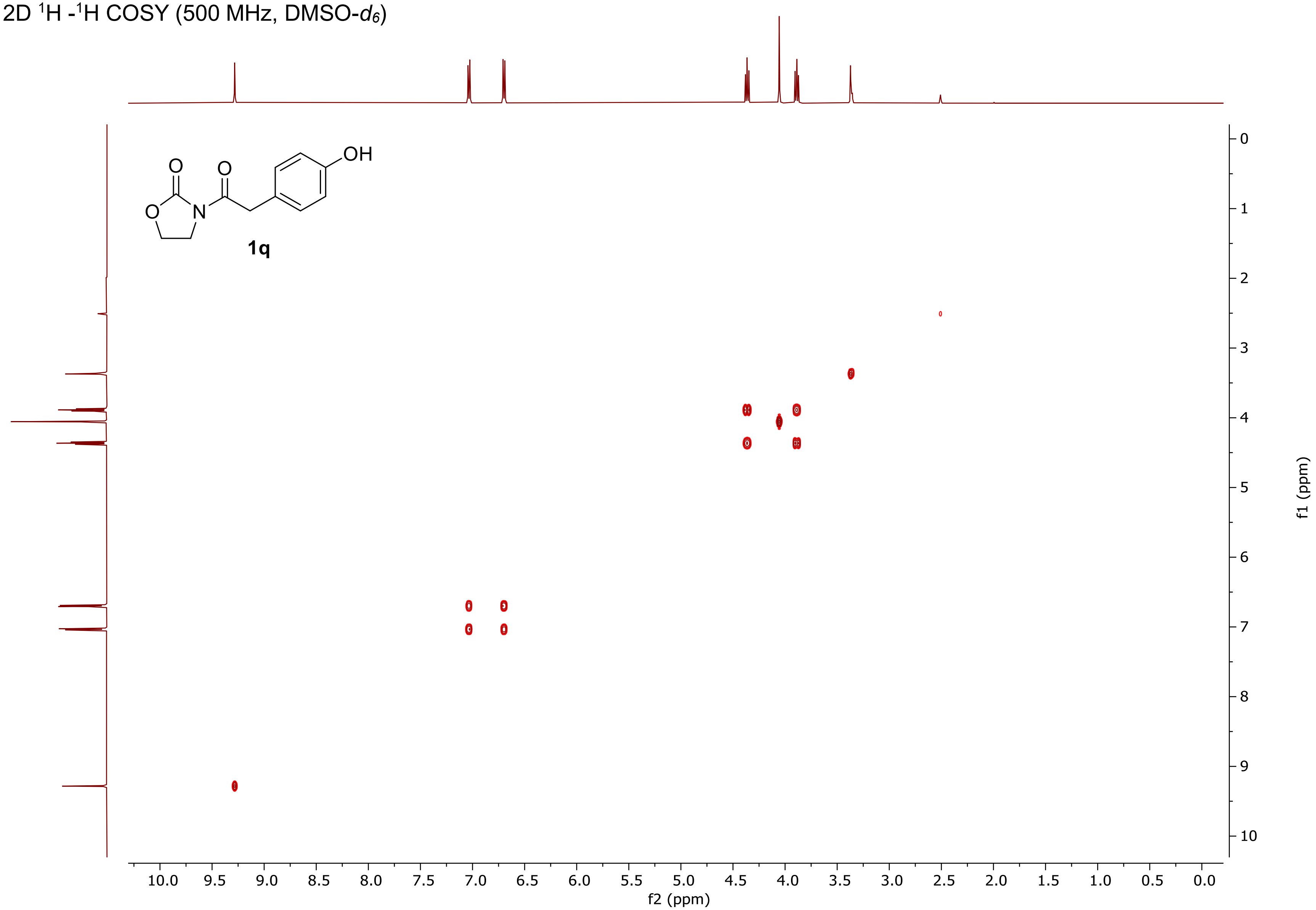

2D $^{1}H$ -$^{13}C$ HSQC (500 MHz, DMSO-$d_6$)

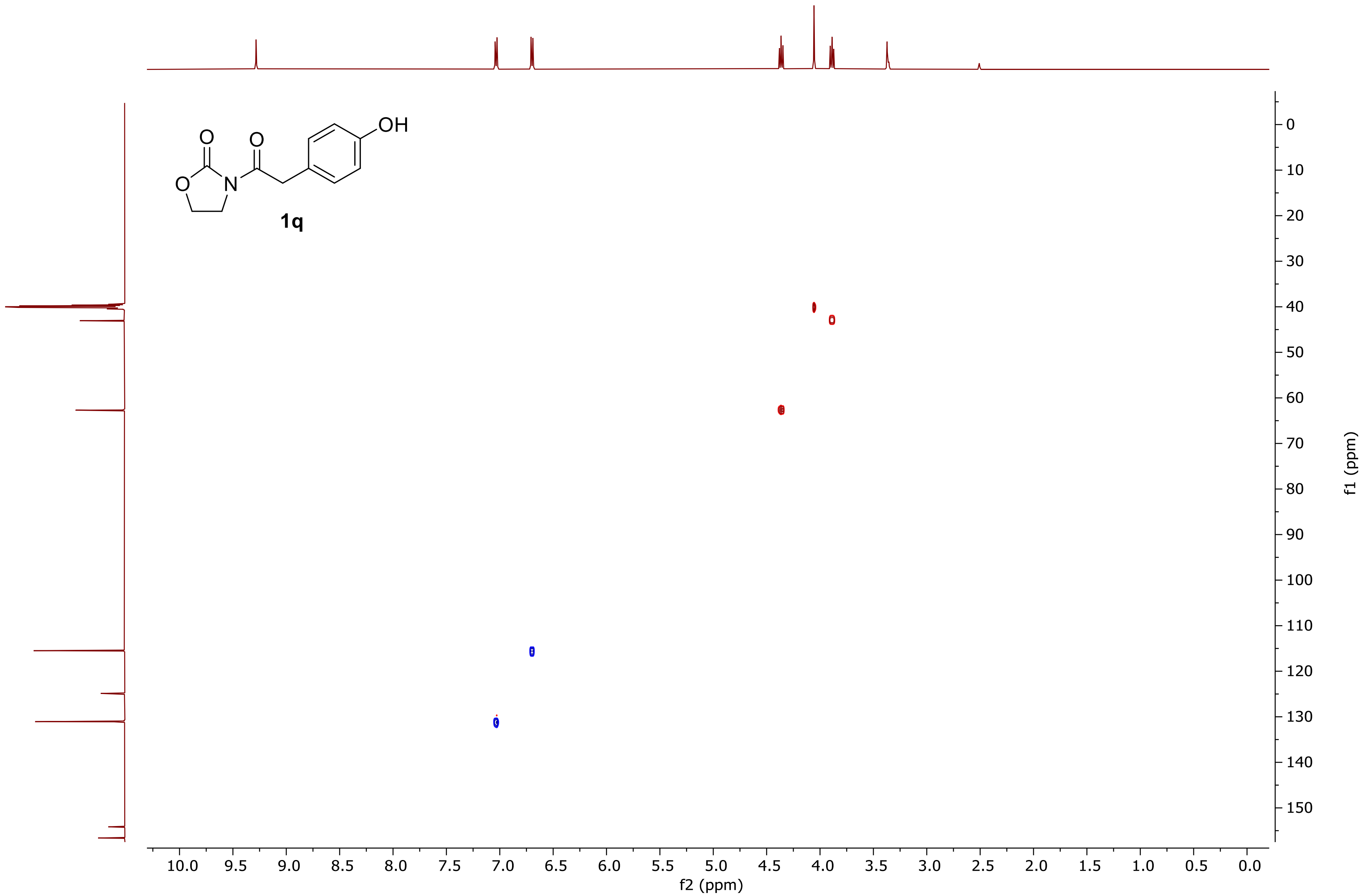

[1]H NMR (400 MHz, DMSO-$d_6$)

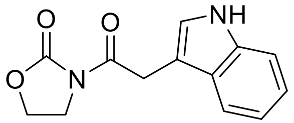

1r

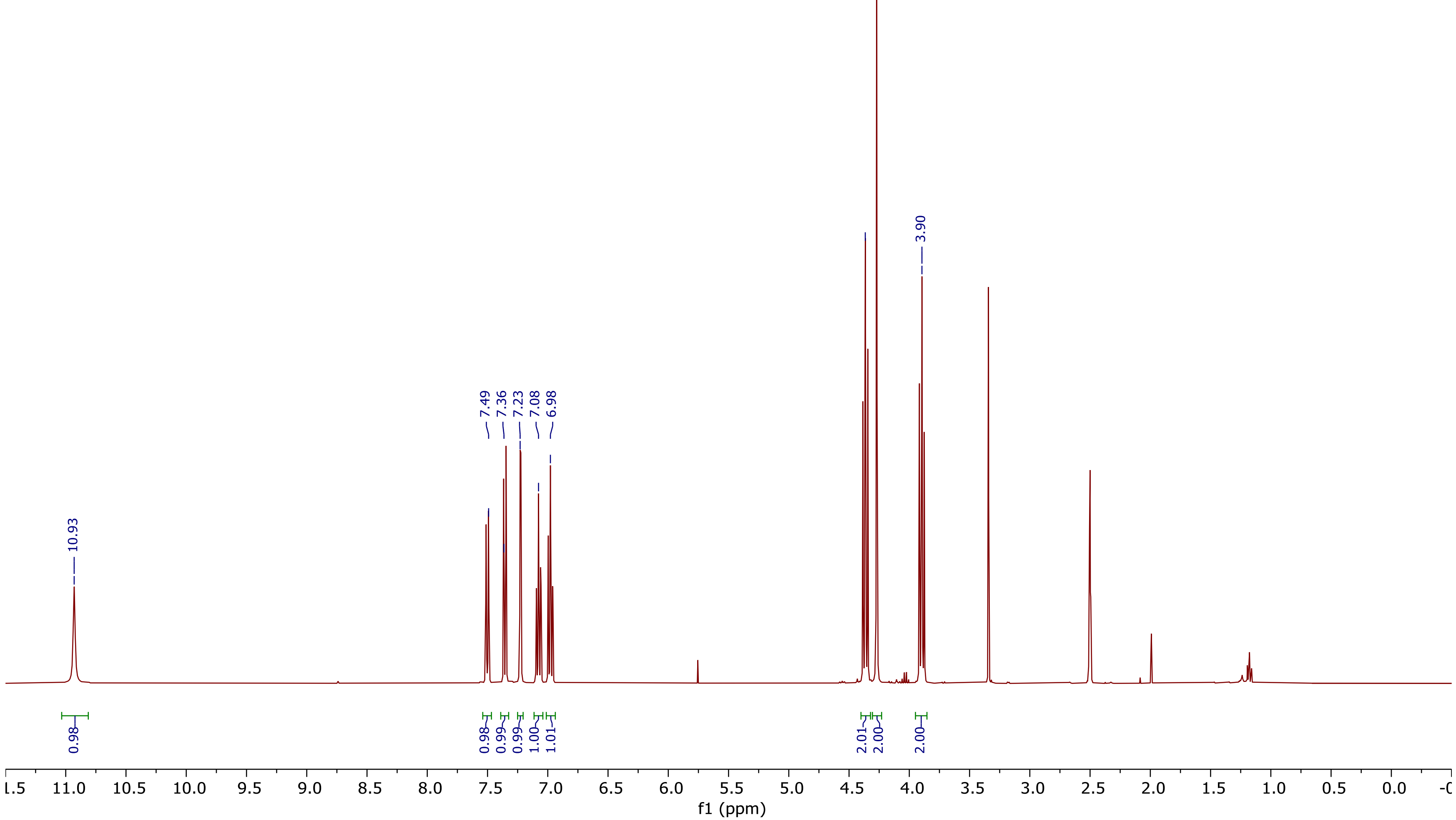

$^{13}C\{^{1}H\}$ NMR (101 MHz, DMSO-$d_6$)

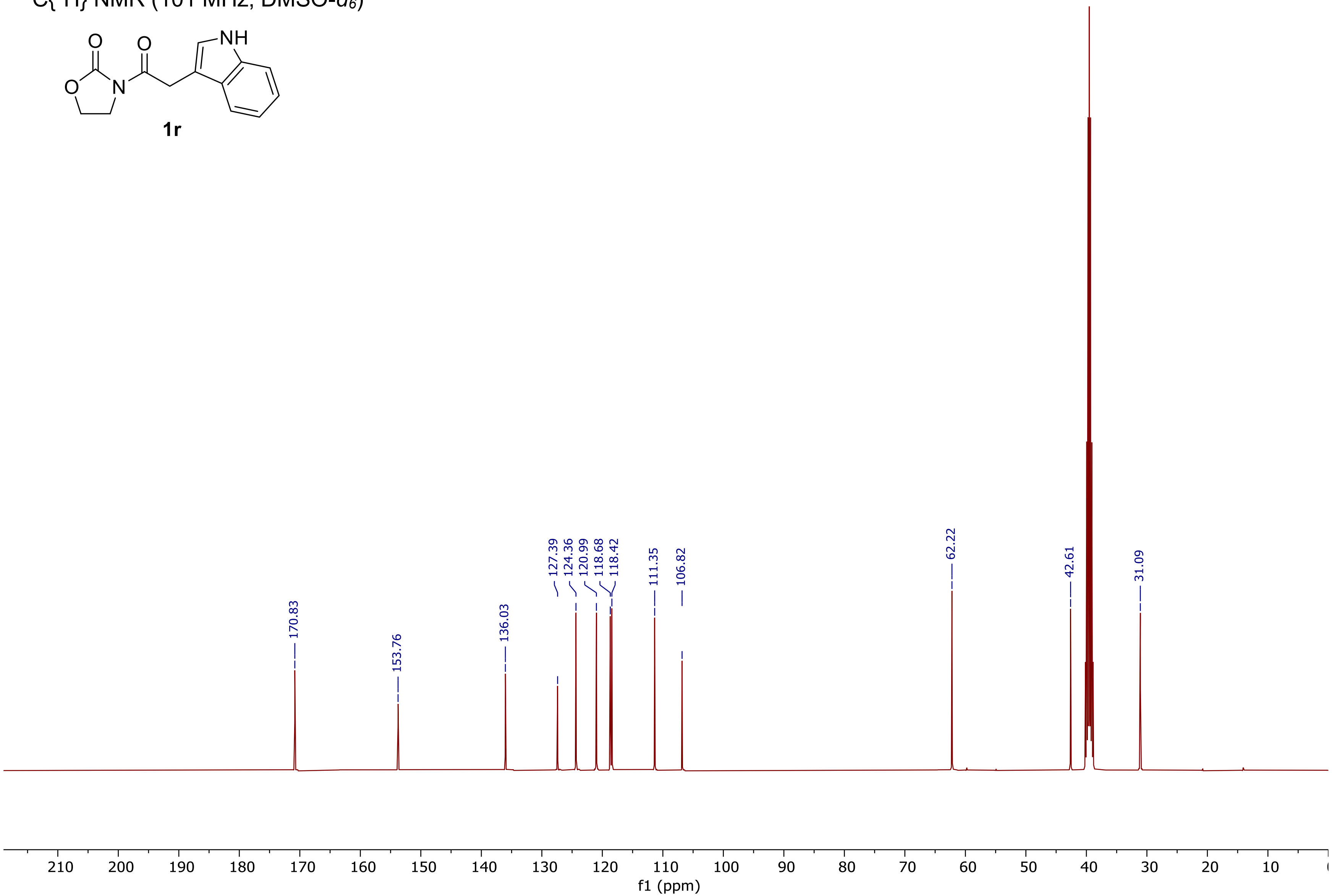

2D $^1$H -$^1$H COSY (400 MHz, DMSO-$d_6$)

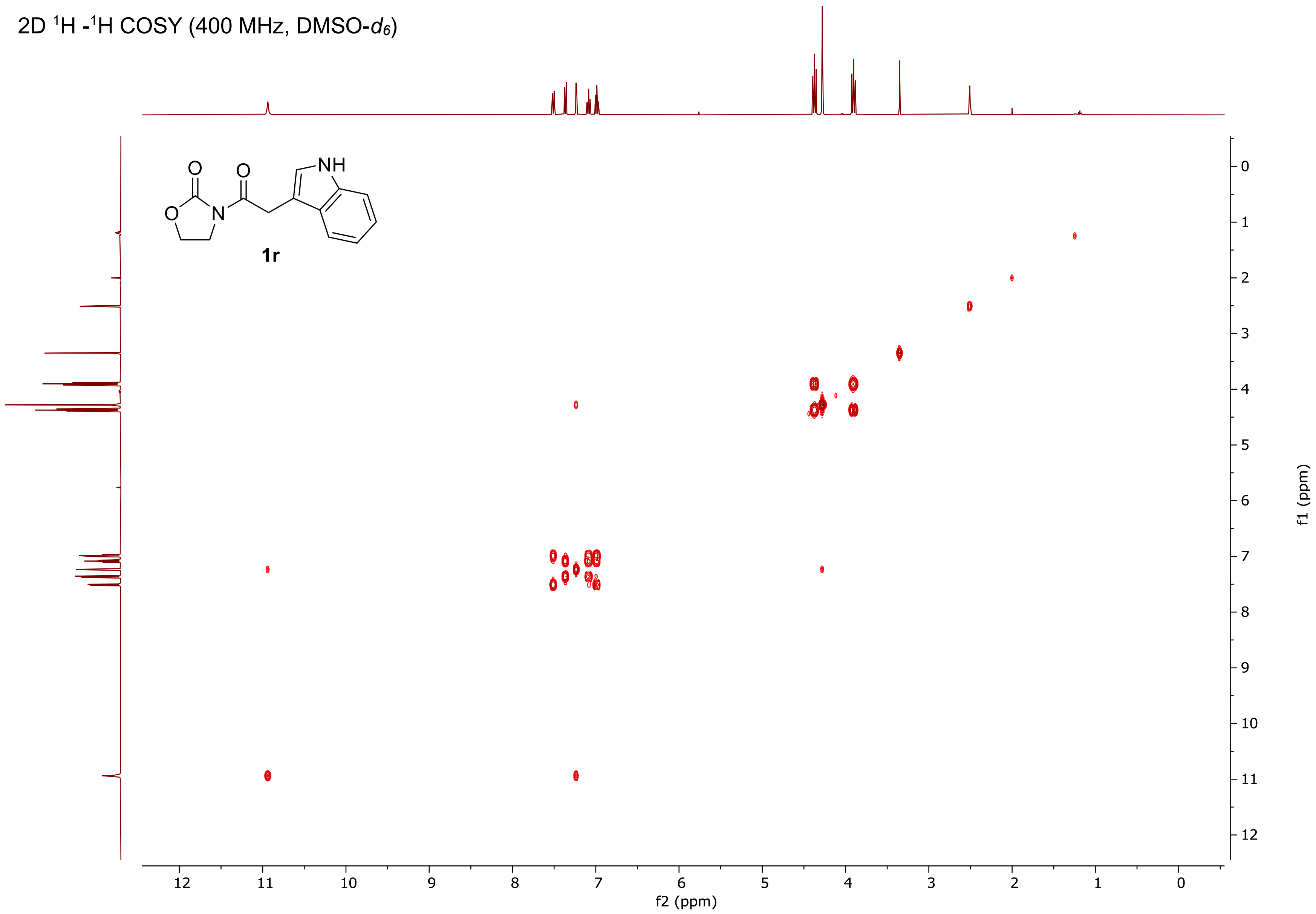

2D $^{1}H$ -$^{13}C$ HSQC (400 MHz, DMSO-$d_6$)

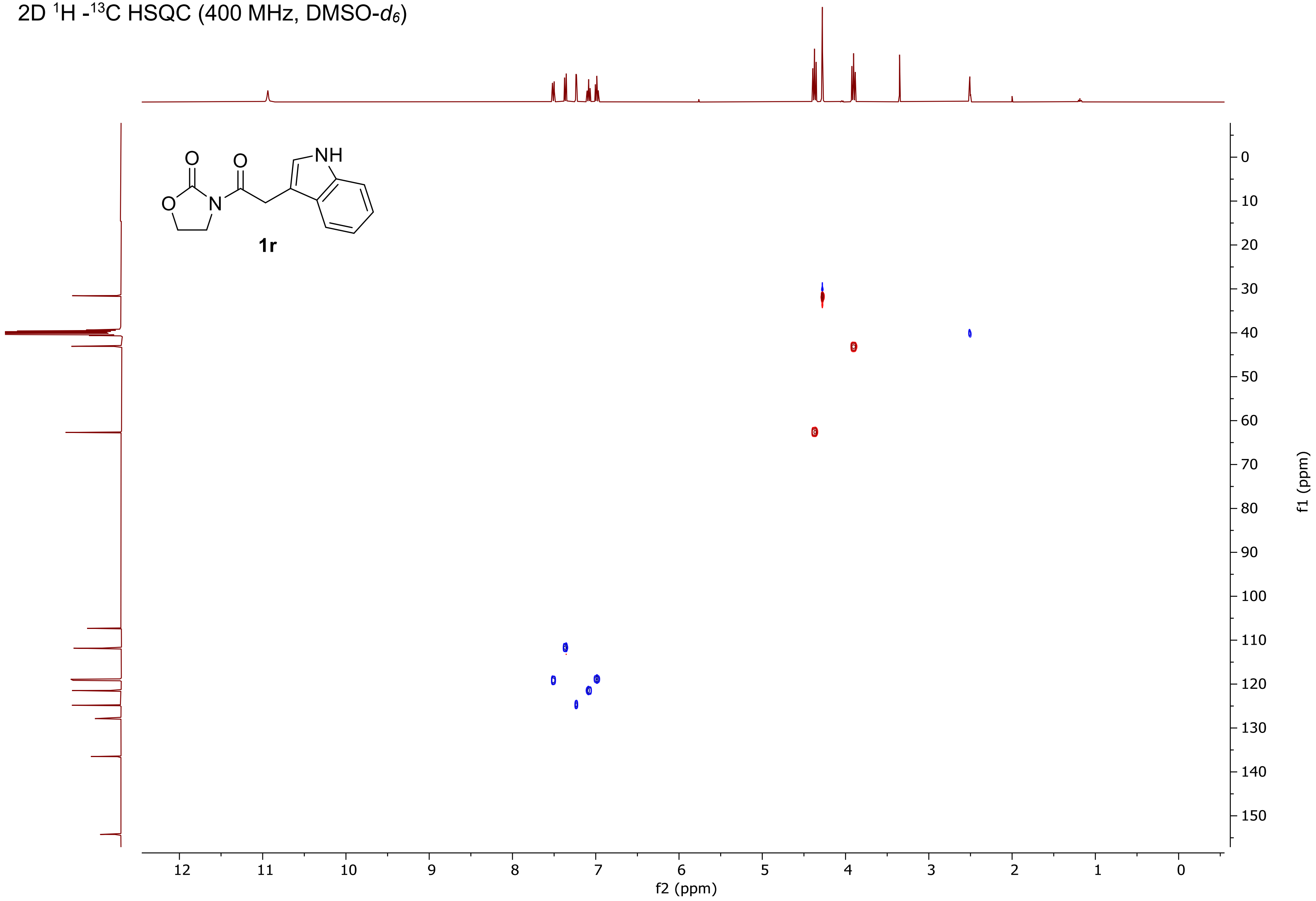

$^{1}H$ NMR (500 MHz, $CDCl_3$)

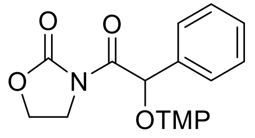


**2a**

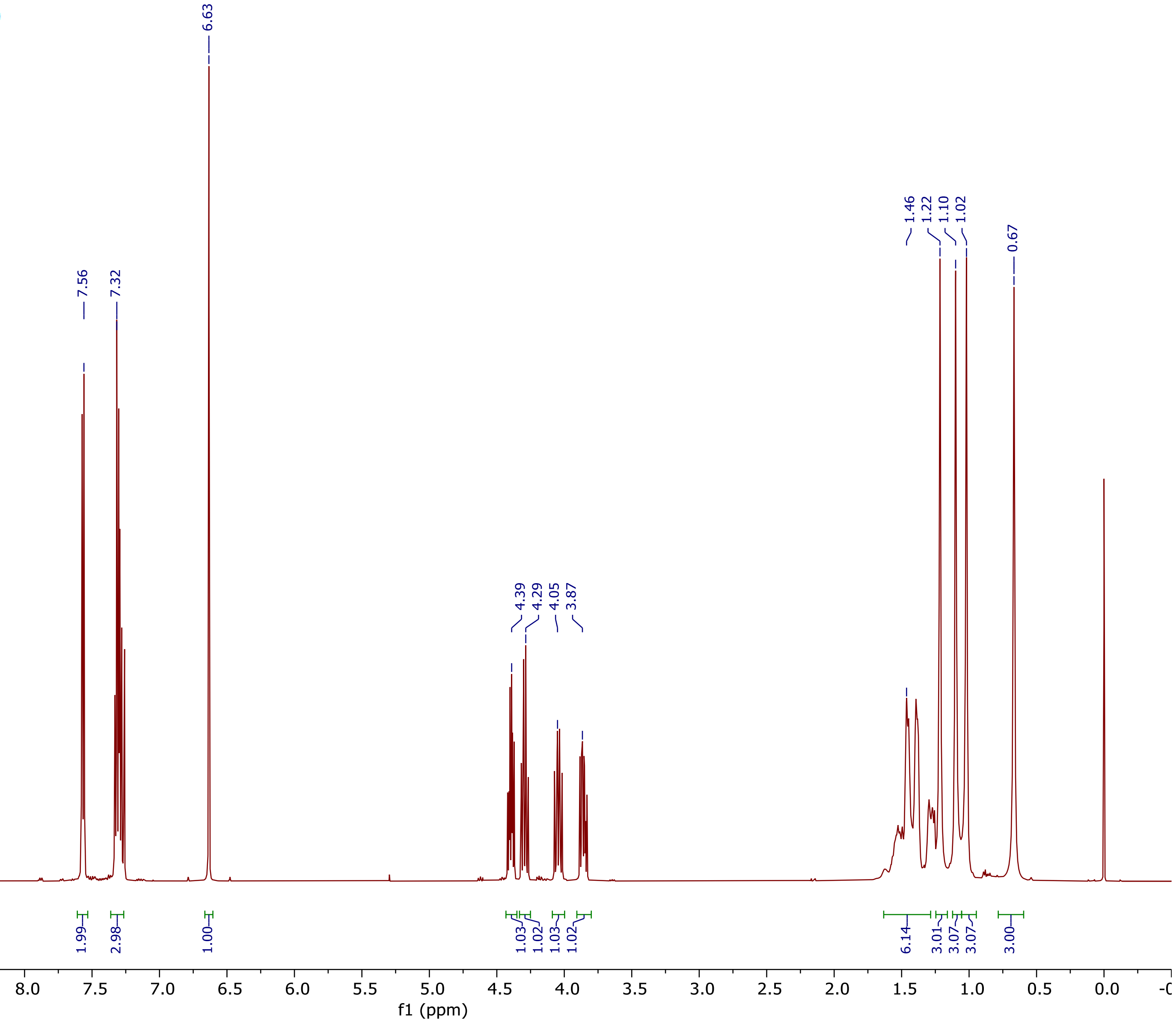

$^{13}C\{^{1}H\}$ NMR (126 MHz, $CDCl_3$)

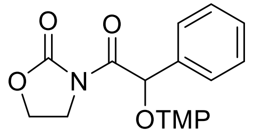


**2a**

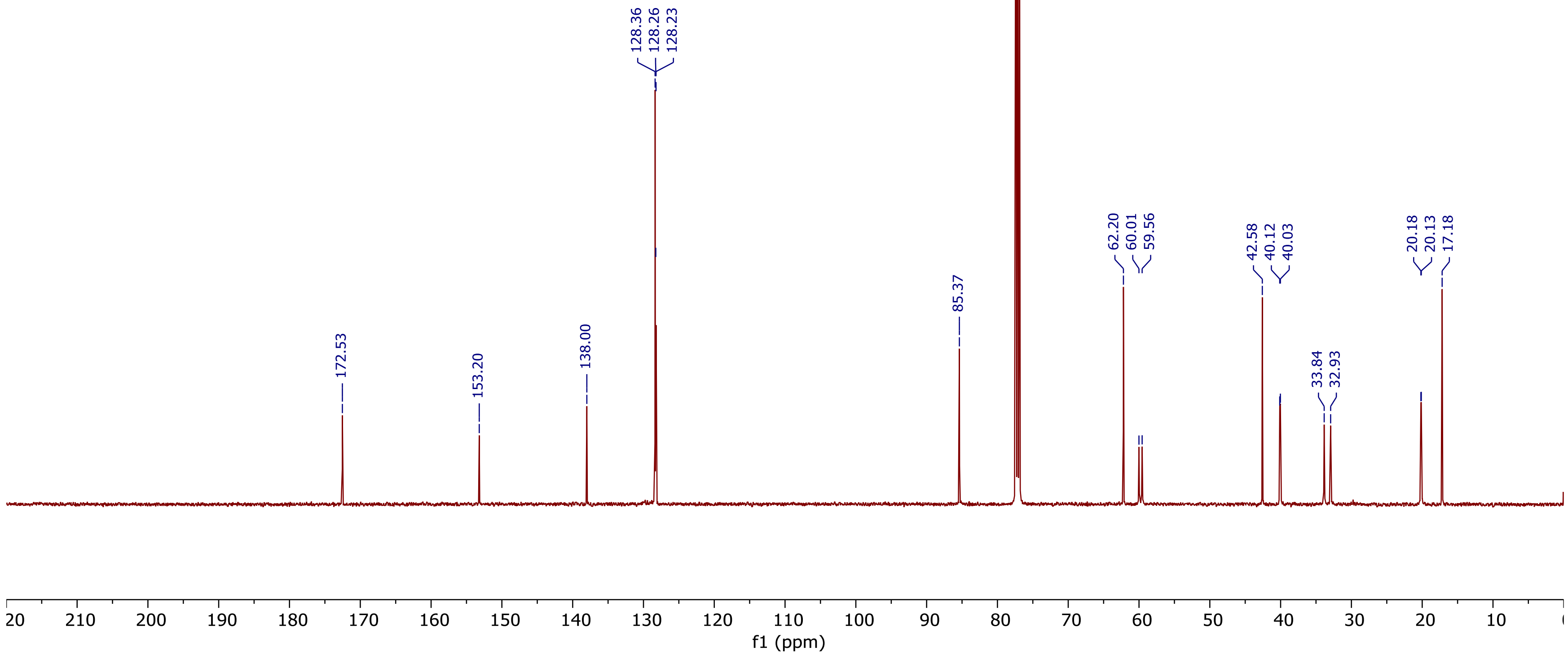

2D $^{1}H$ -$^{1}H$ COSY (500 MHz, $CDCl_3$)

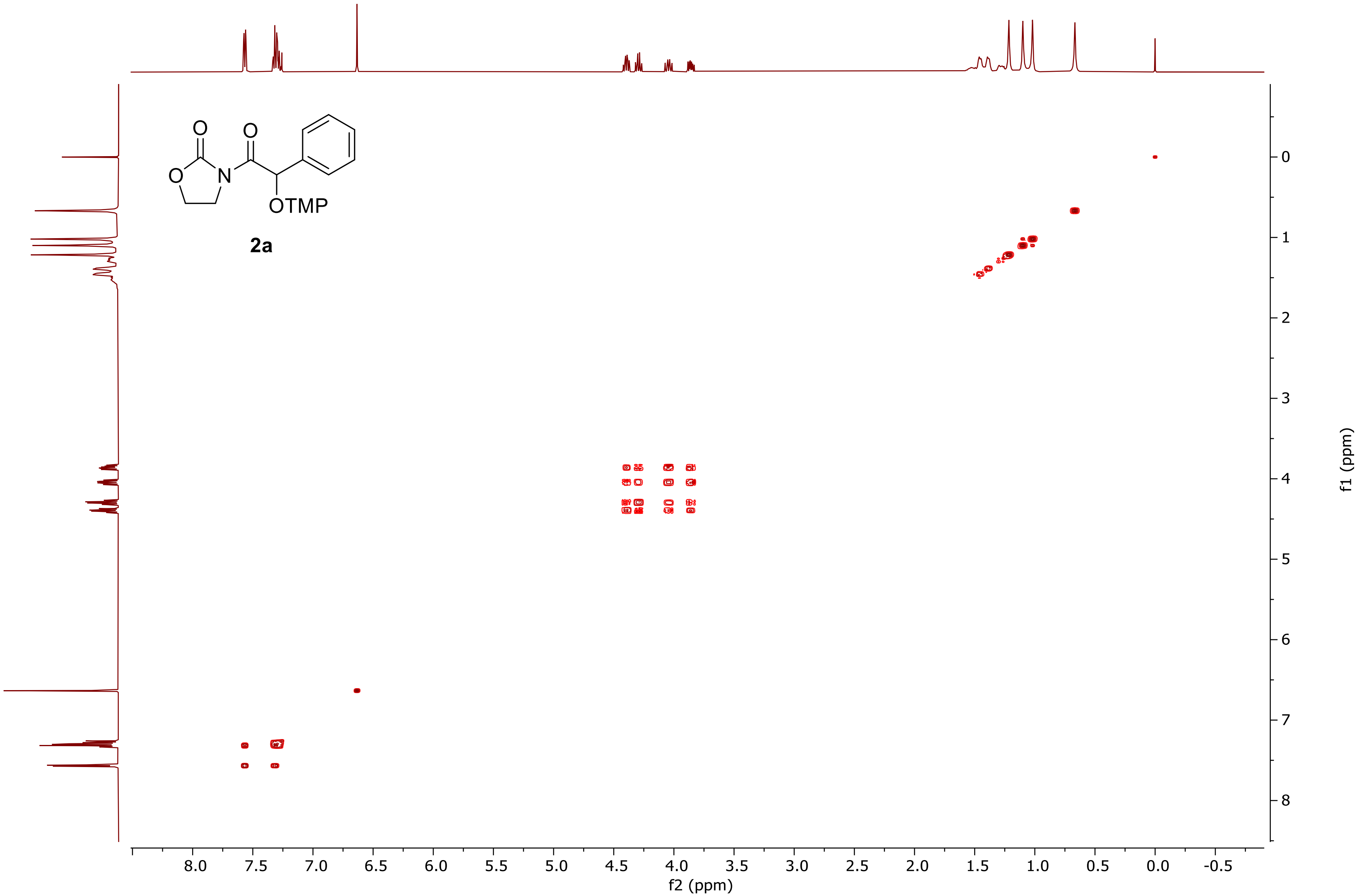

2D $^1$H -$^{13}$C HSQC (500 MHz, $CDCl_3$)

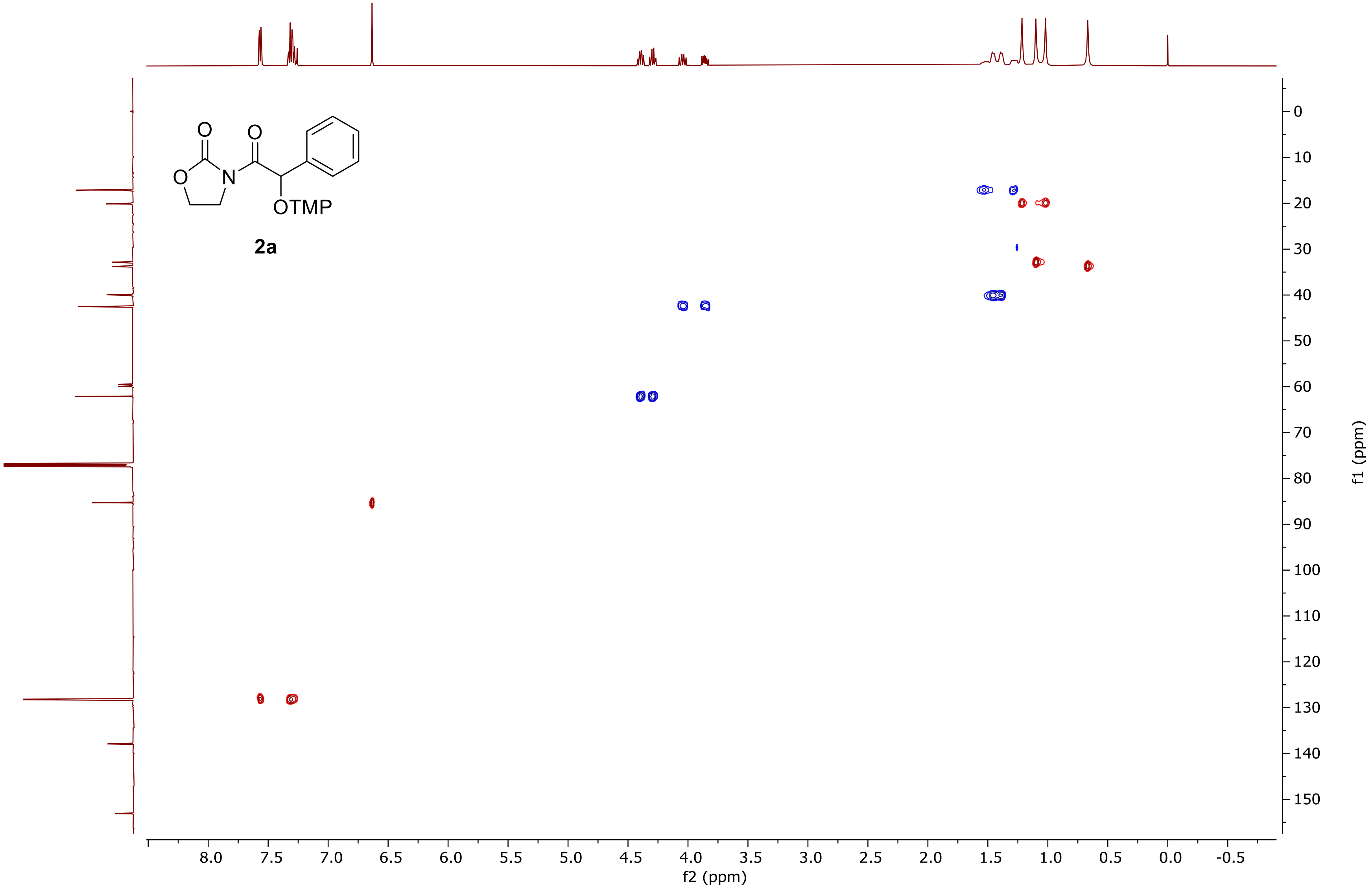

$^{1}H$ NMR (400 MHz, $CDCl_3$)

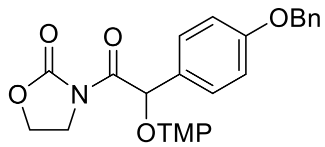


**2b**

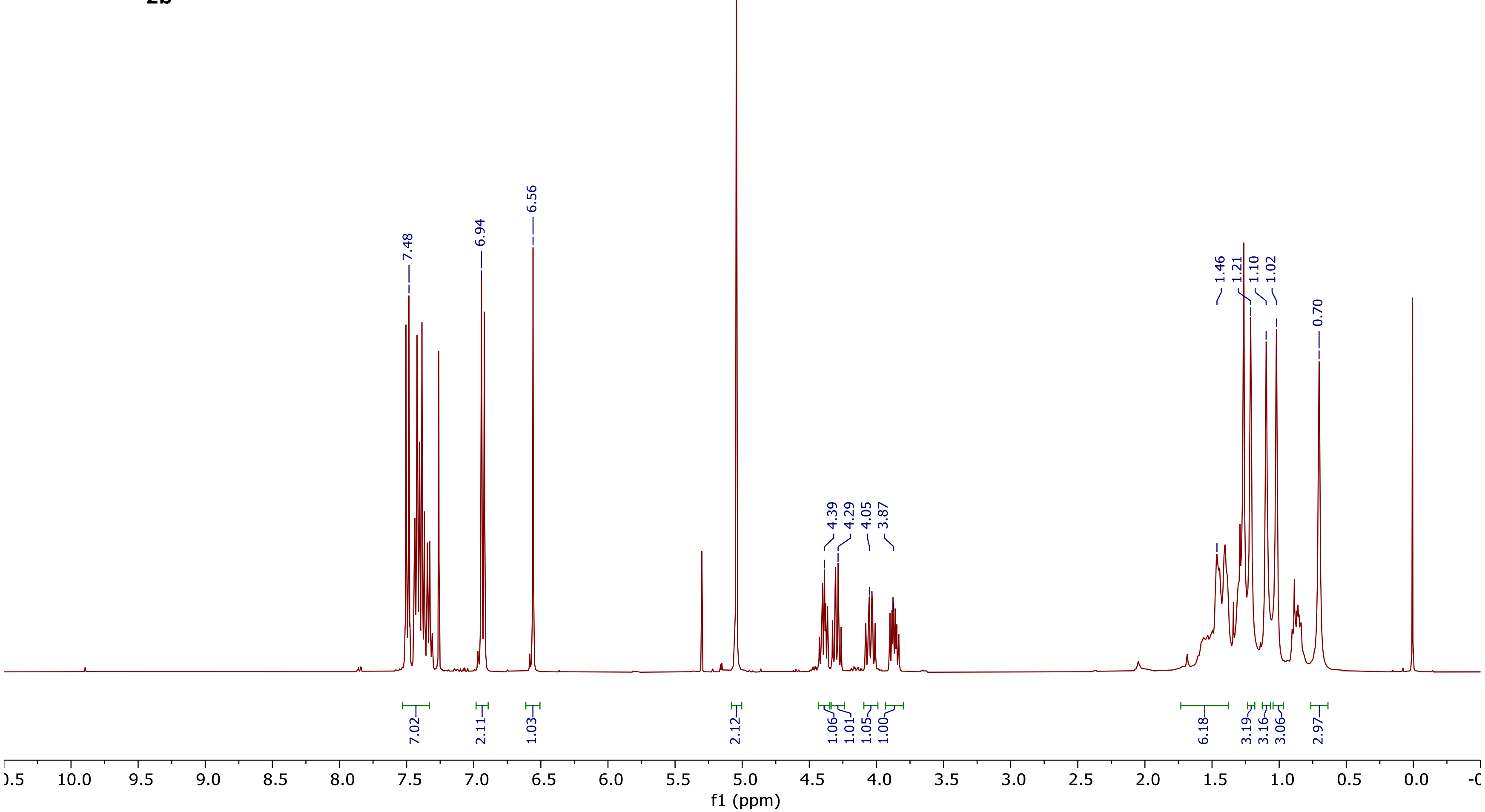

$^{13}C\{^{1}H\}$ NMR (101 MHz, $CDCl_3$)

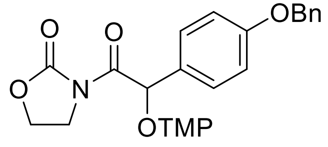


**2b**

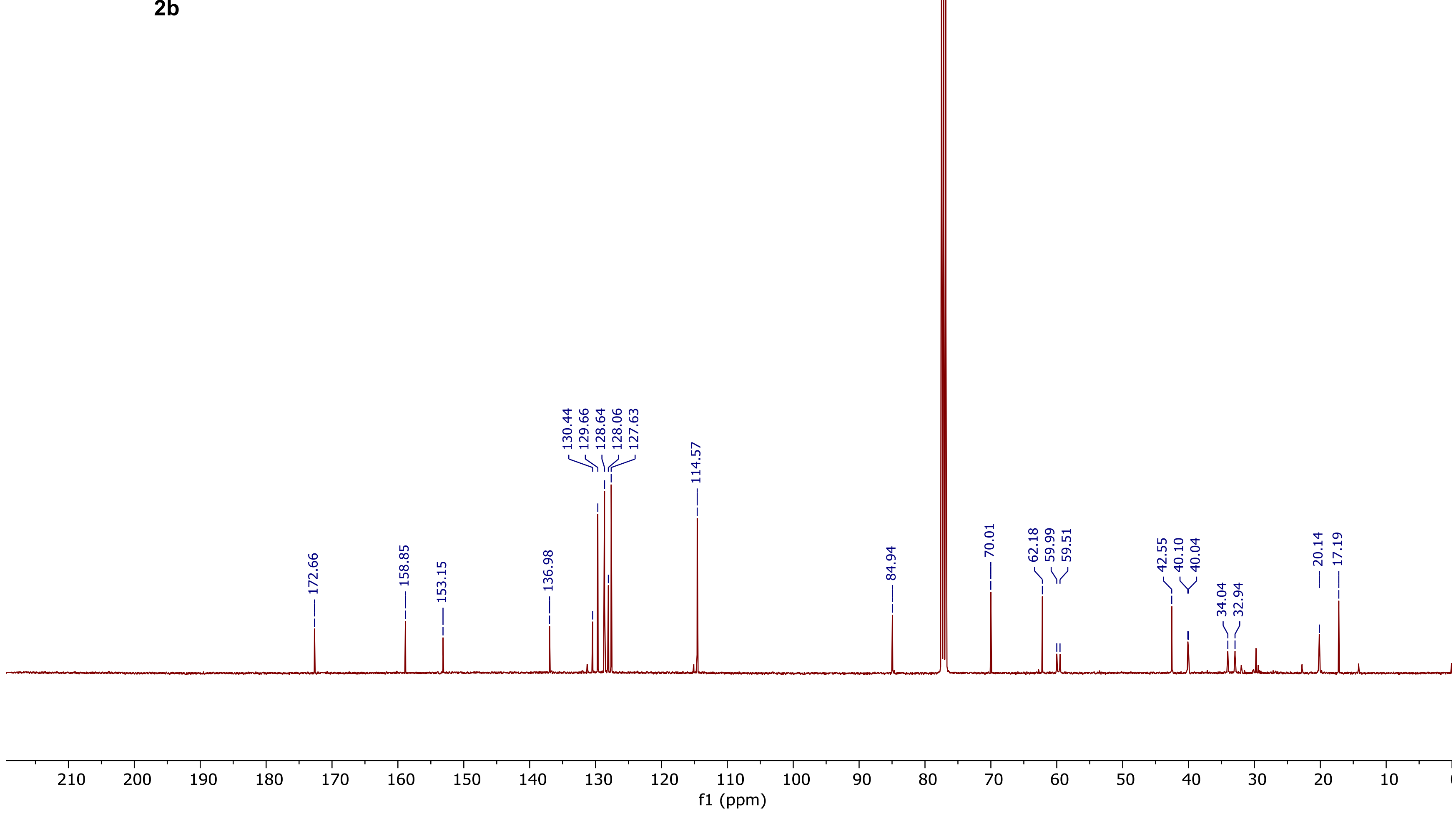

2D $^{1}H$ -$^{1}H$ COSY (400 MHz, $CDCl_3$)

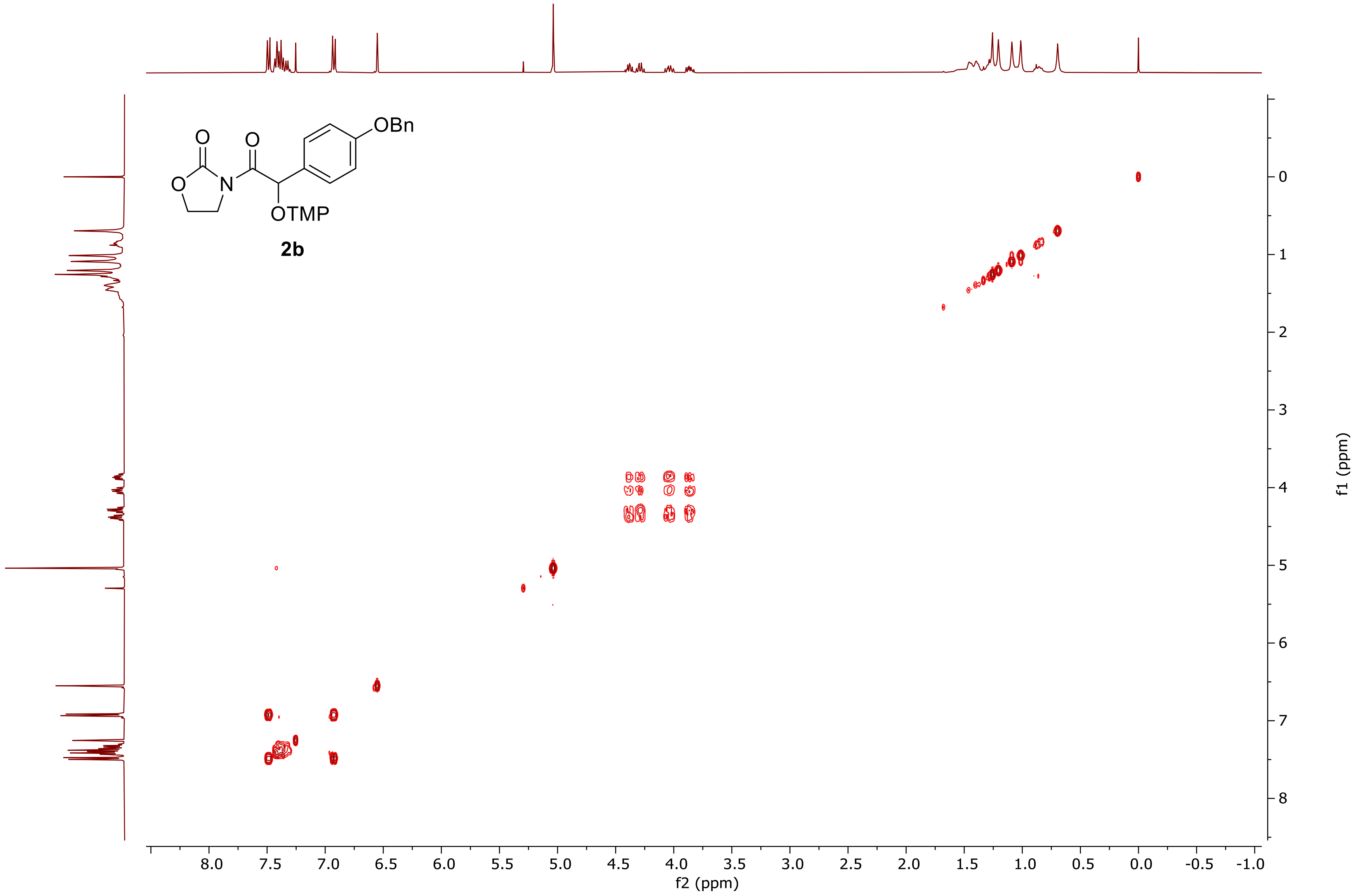

2D $^{1}$H -$^{13}$C HSQC (400 MHz, $CDCl_3$)

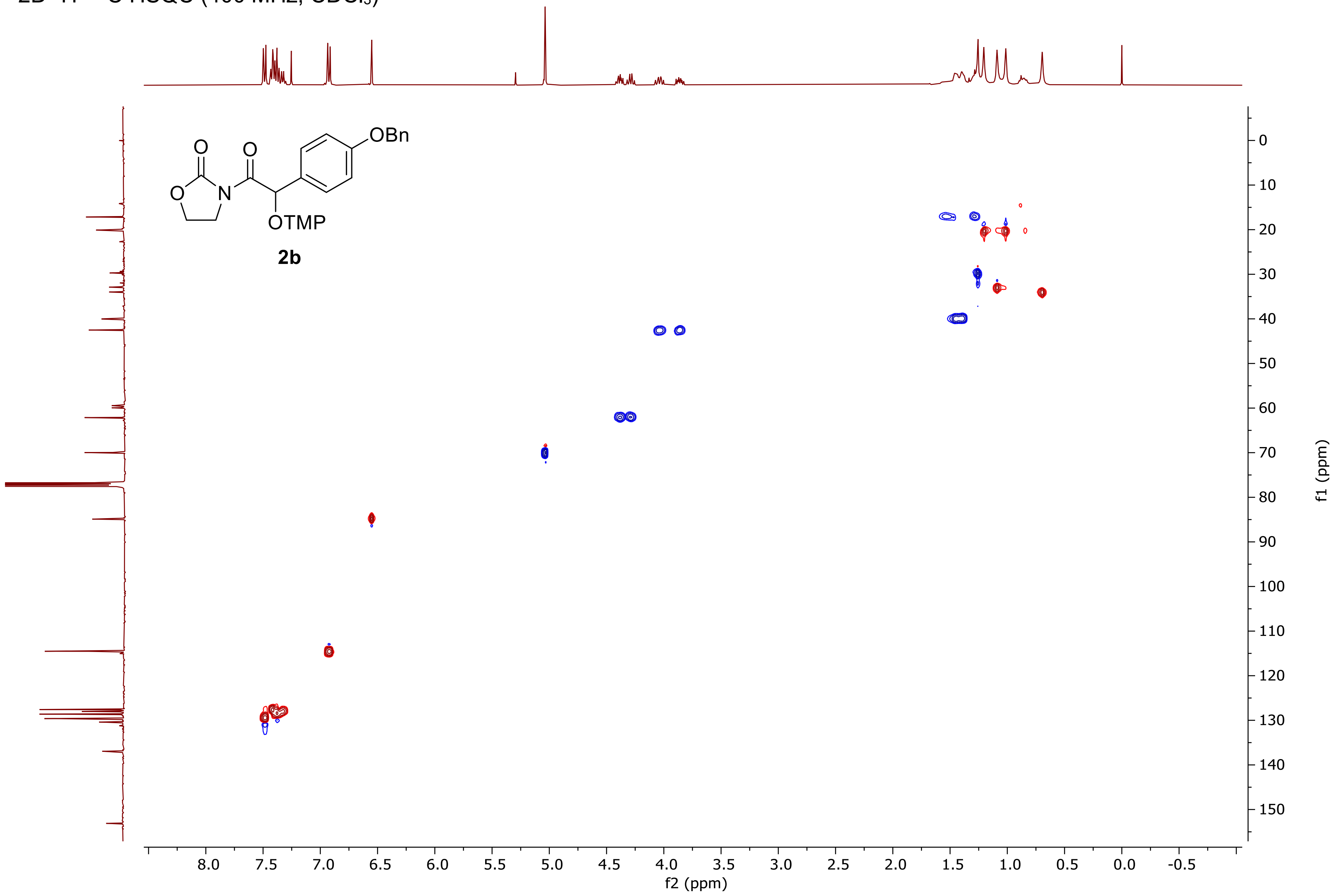

[1]H NMR (400 MHz, $CDCl_3$)

**2c**

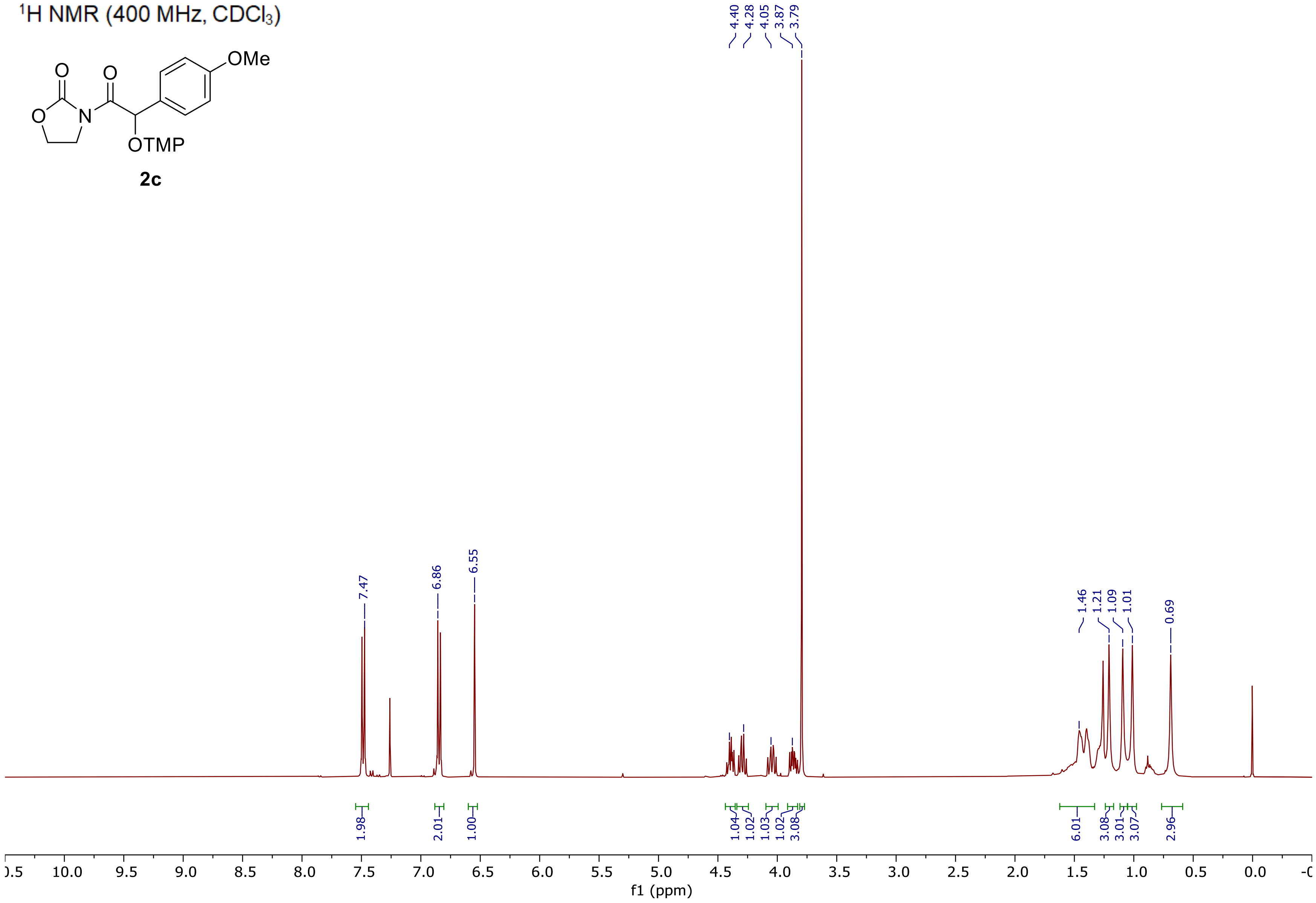

$^{13}C\{^1H\}$ NMR (101 MHz, $CDCl_3$)

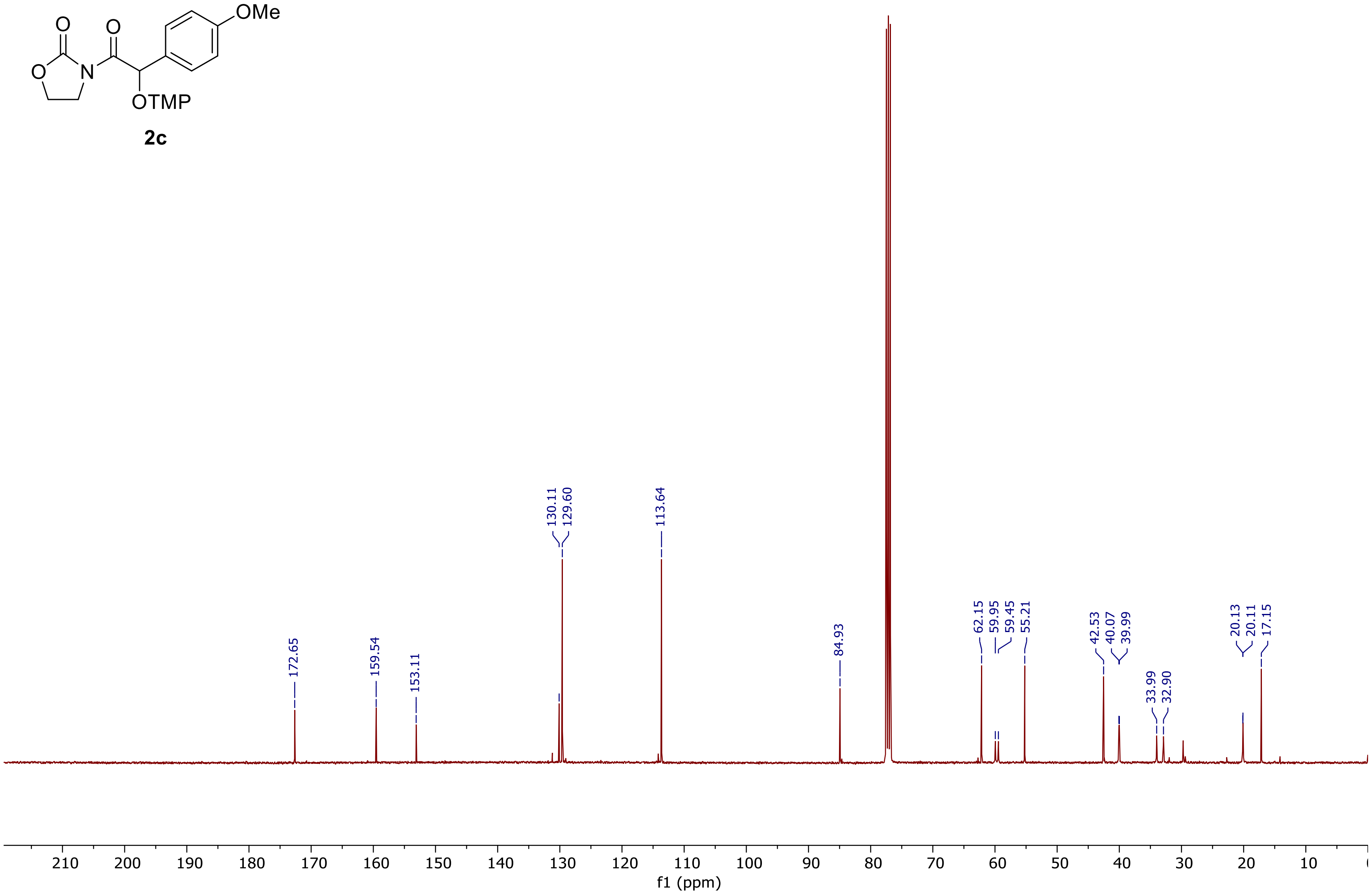


**2c**

2D $^{1}H$ -$^{1}H$ COSY (400 MHz, $CDCl_3$)

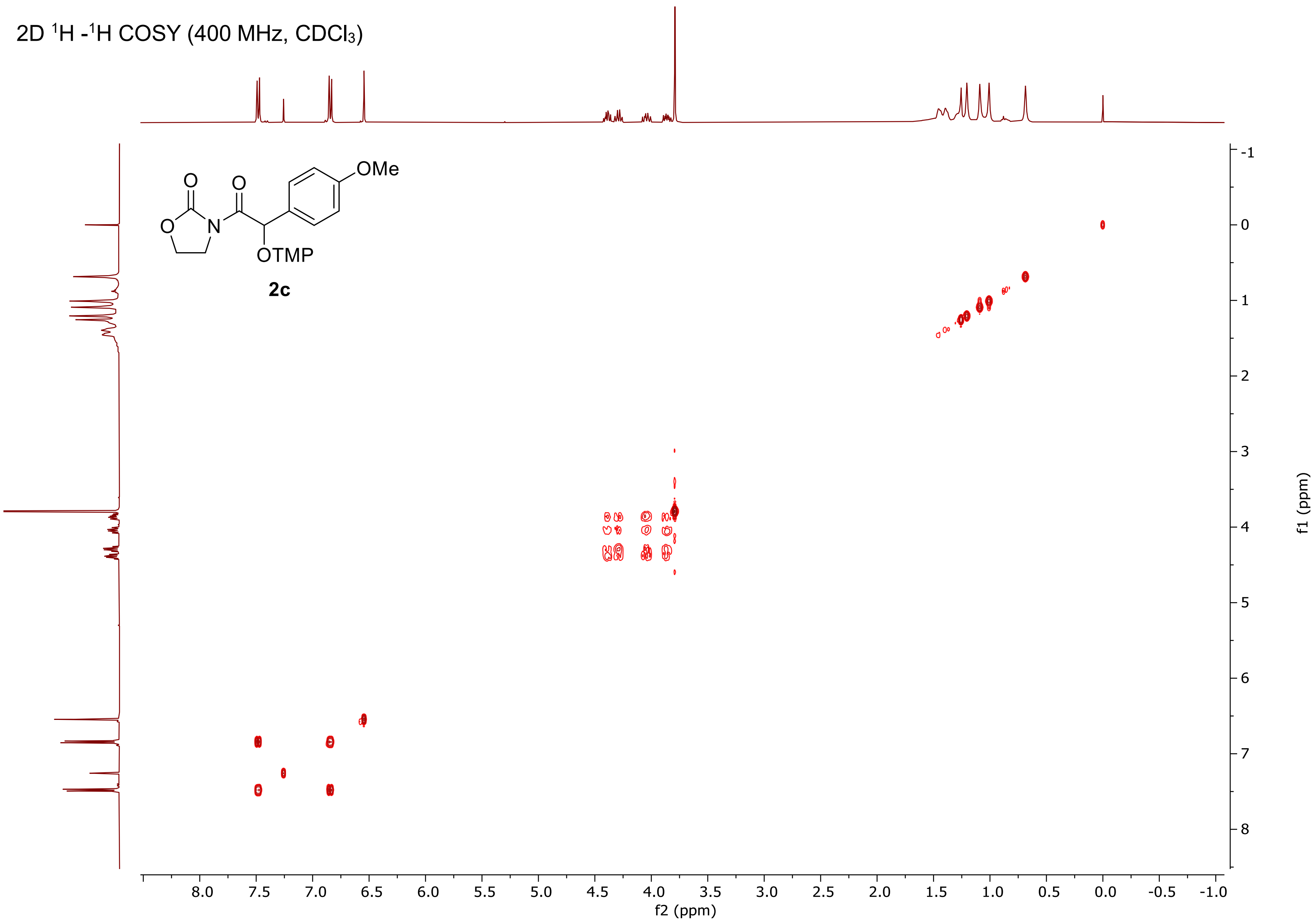

2D $^1$H -$^{13}$C HSQC (400 MHz, $CDCl_3$)

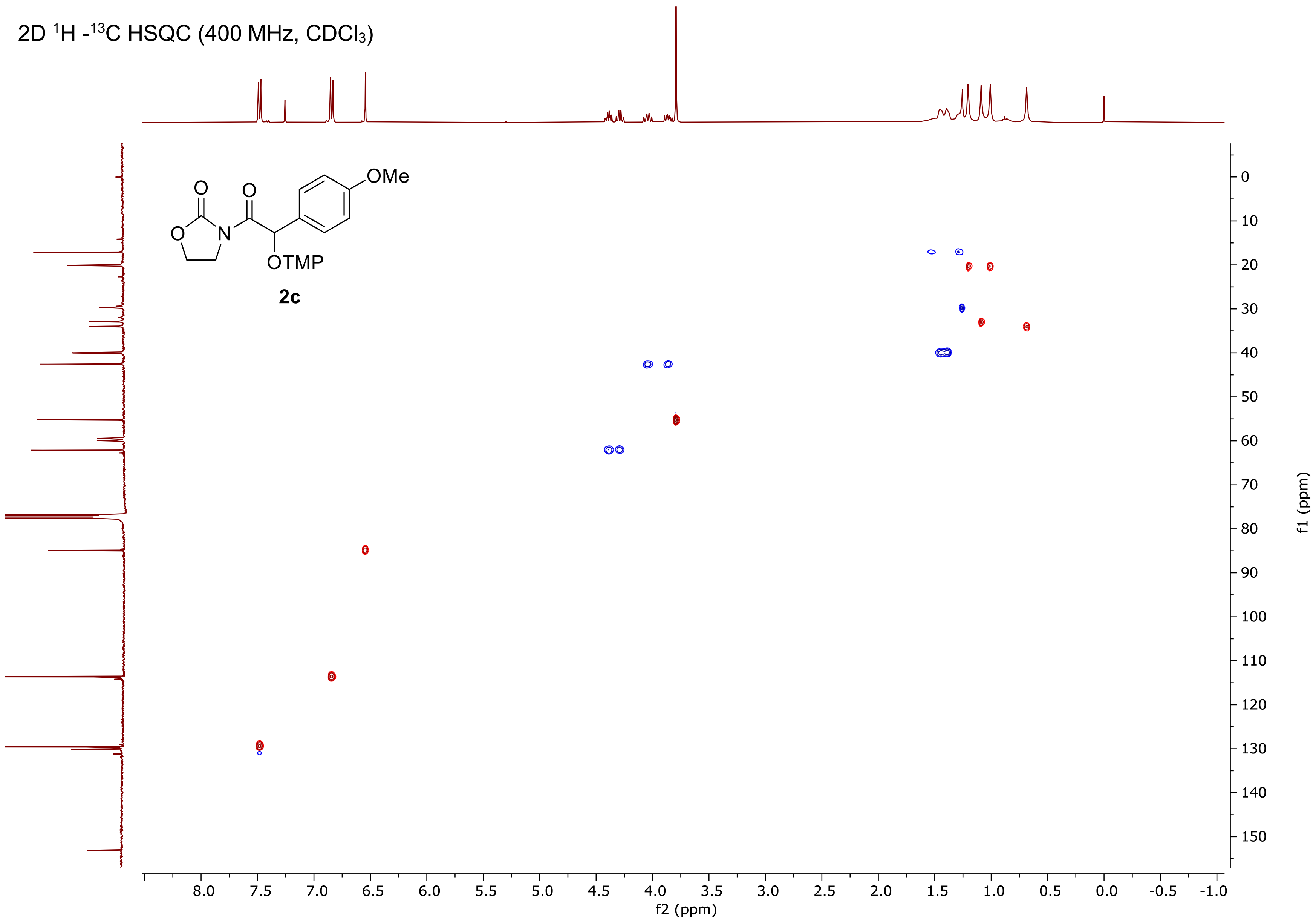

$^1H$ NMR (500 MHz, $CDCl_3$)

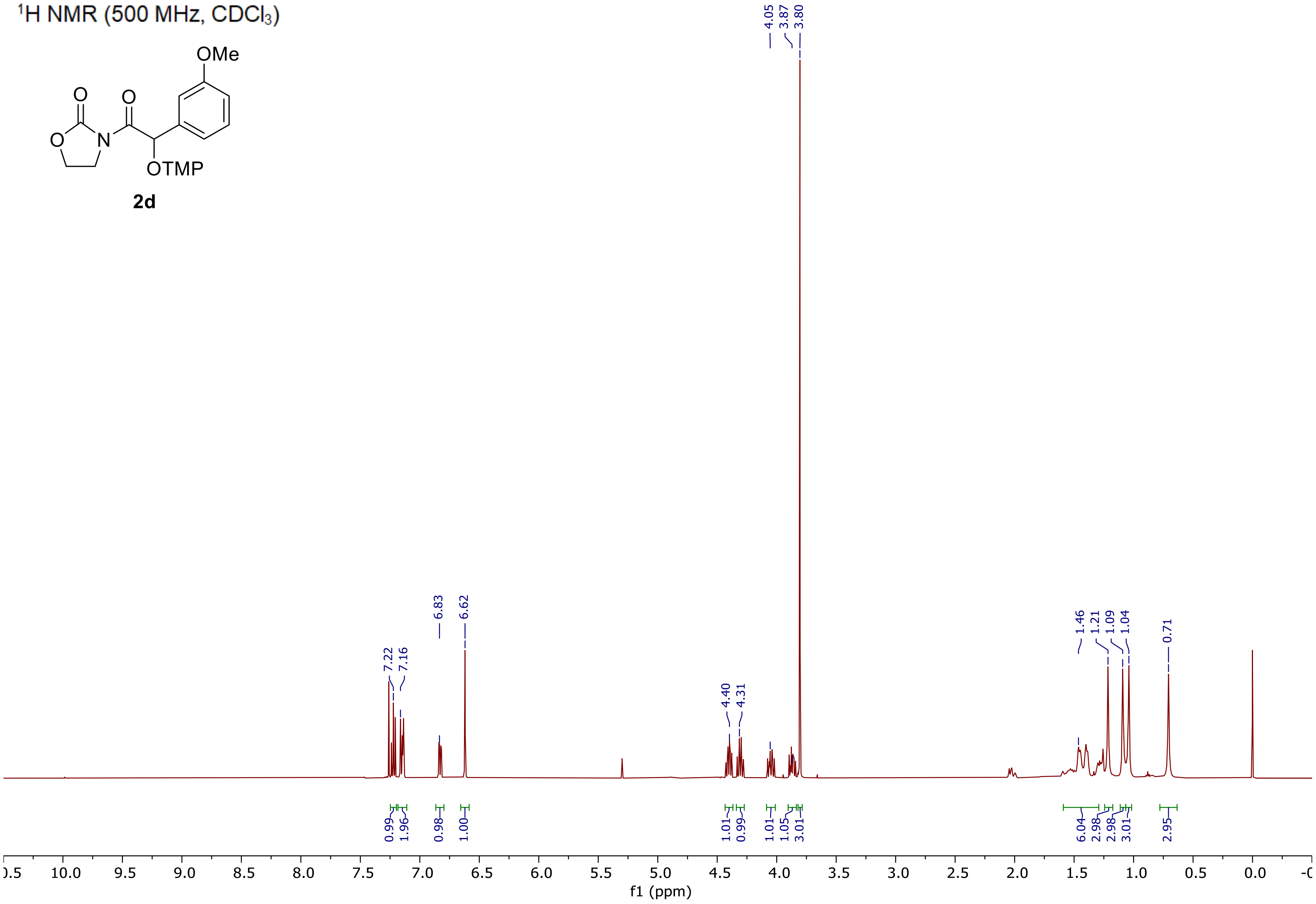

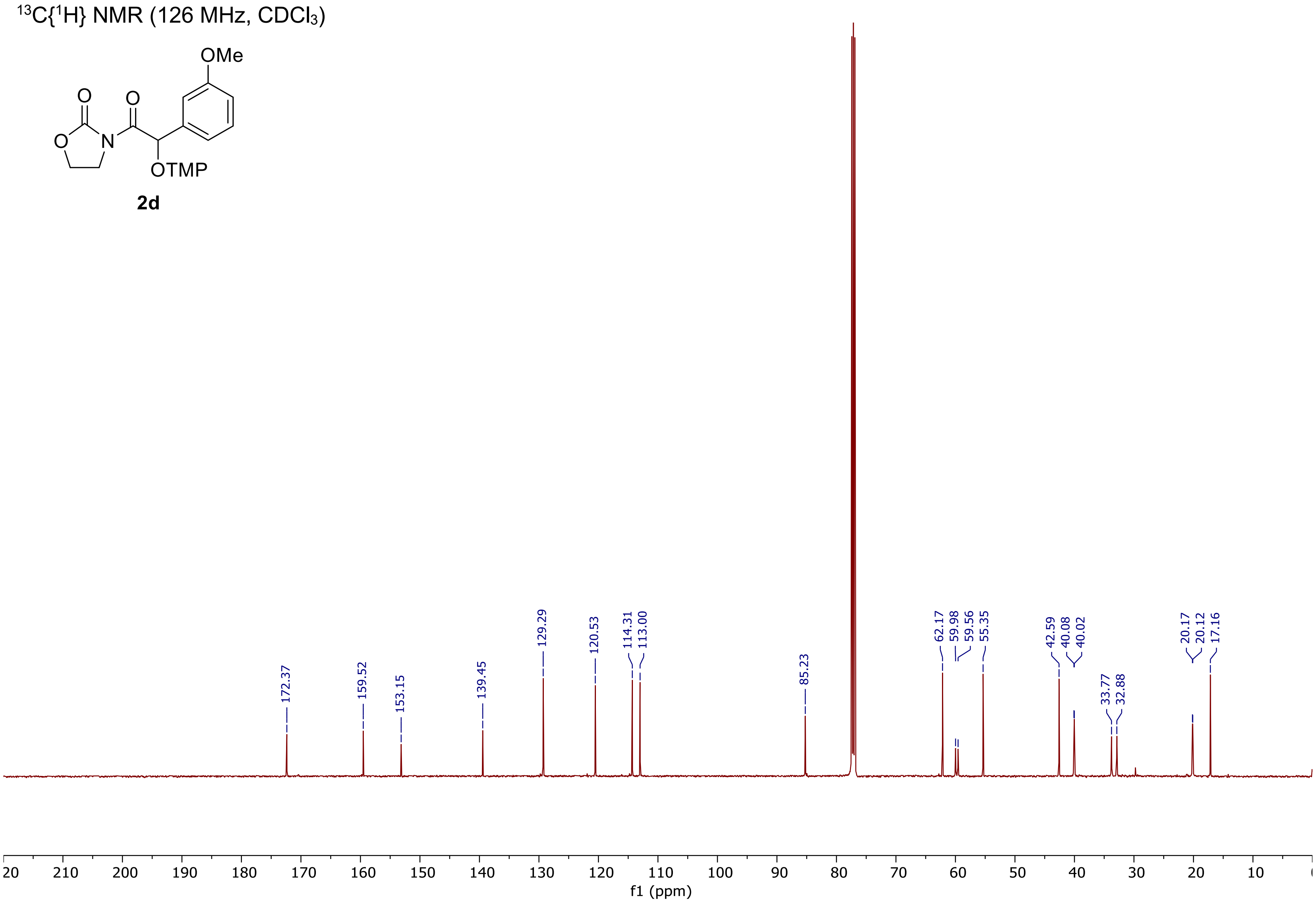

$^{13}C\{^1H\}$ NMR (126 MHz, $CDCl_3$)
OMe
O
O
O
N
OTMP
2d
172.37
159.52
153.15
139.45
129.29
120.53
114.31
113.00
85.23
62.17
59.98
59.56
55.35
42.59
40.08
40.02
33.77
32.88
20.17
20.12
17.16
f1 (ppm)

2D $^{1}H$ -$^{1}H$ COSY (500 MHz, $CDCl_3$)

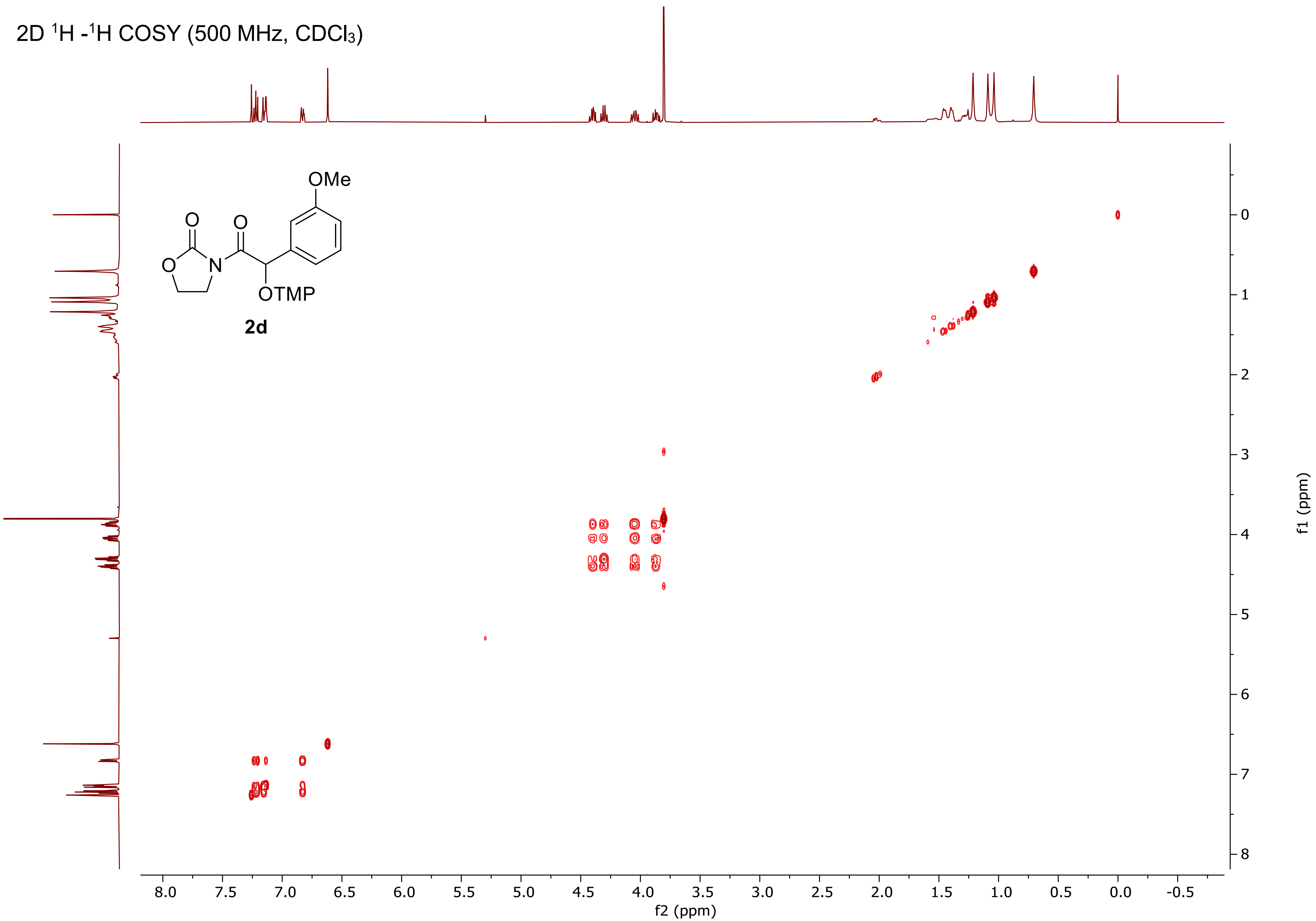

2D $^{1}H$ -$^{13}C$ HSQC (500 MHz, $CDCl_3$)

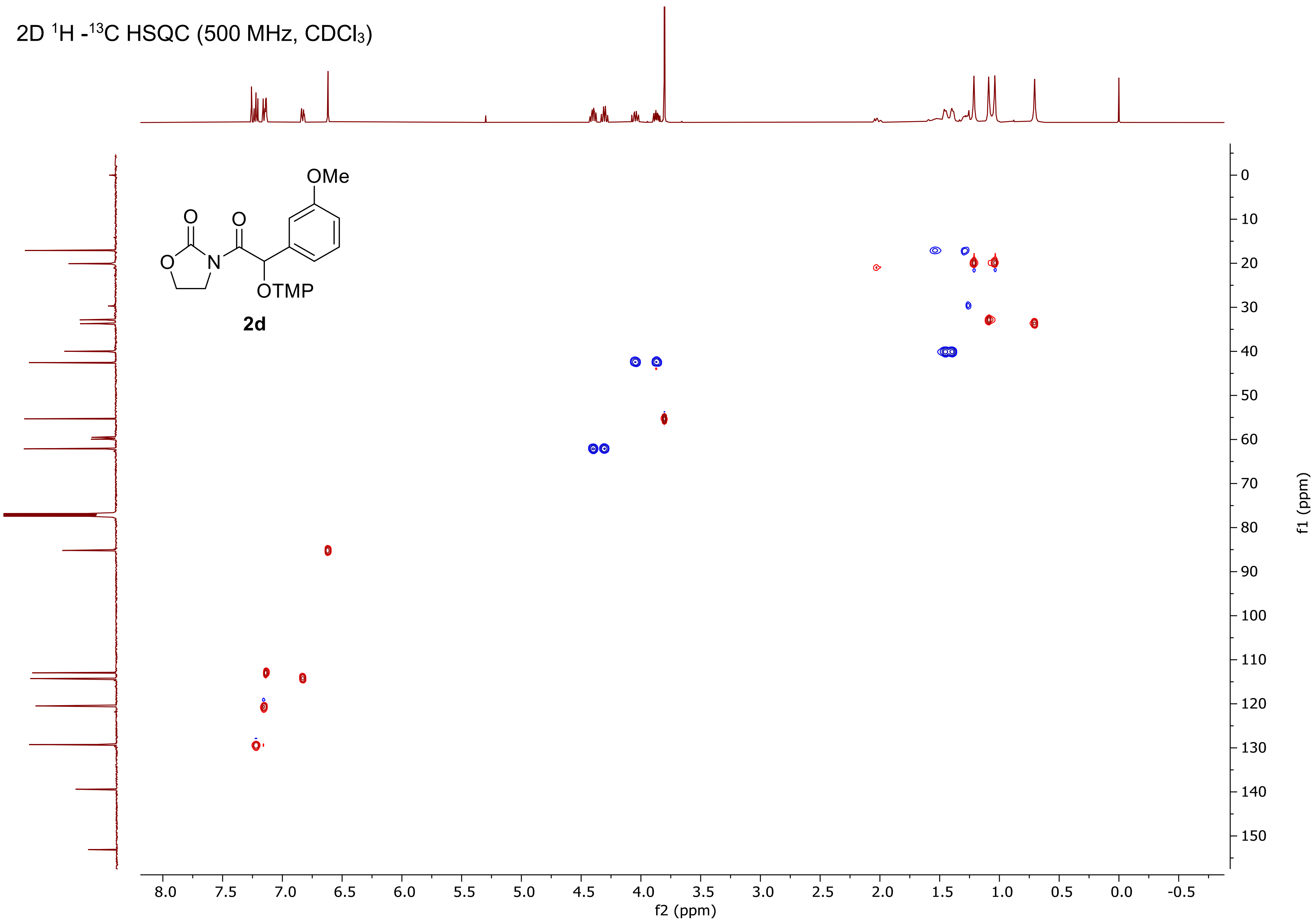

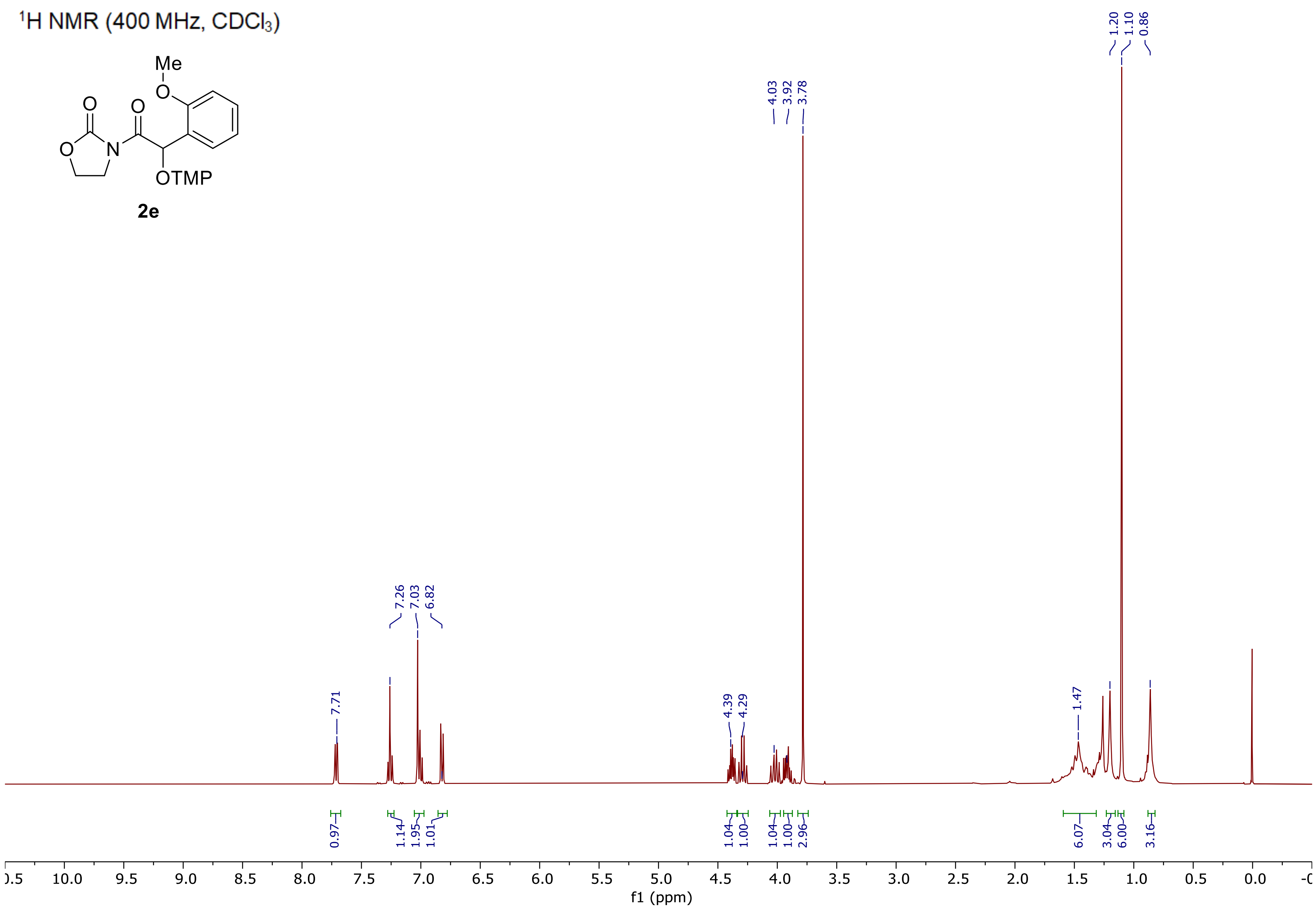

$^1$H NMR (400 MHz, CDCl$_3$)
Me
O
O
O
O
N
OTMP
2e
7.71
7.26
7.03
6.82
4.39
4.29
4.03
3.92
3.78
1.47
1.20
1.10
0.86
0.97
1.14
1.95
1.01
1.04
1.00
1.04
1.00
2.96
6.07
3.04
6.00
3.16
f1 (ppm)

$^{13}C\{^1H\}$ NMR (101 MHz, $CDCl_3$)

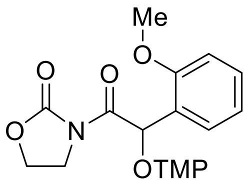

**2e**

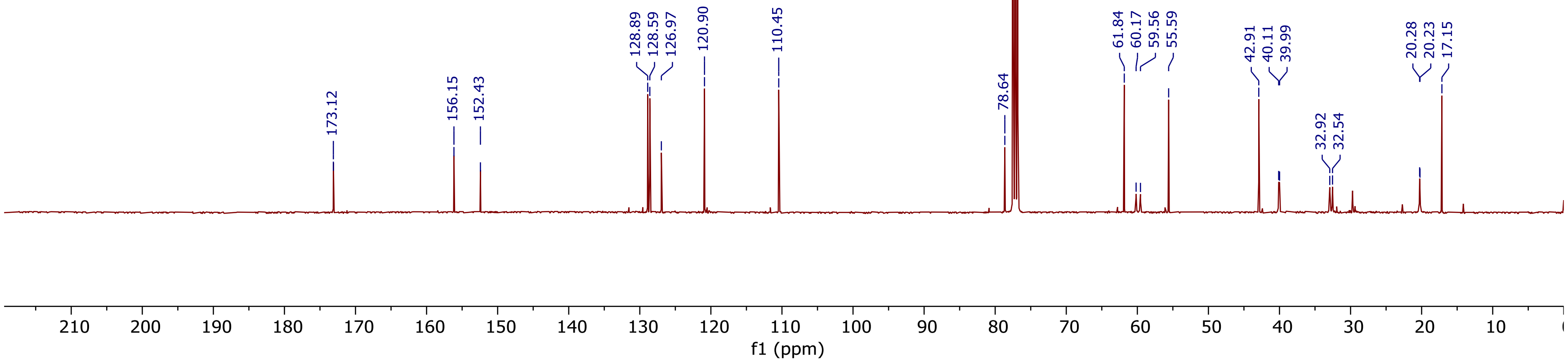

2D $^{1}H$ -$^{1}H$ COSY (400 MHz, $CDCl_3$)

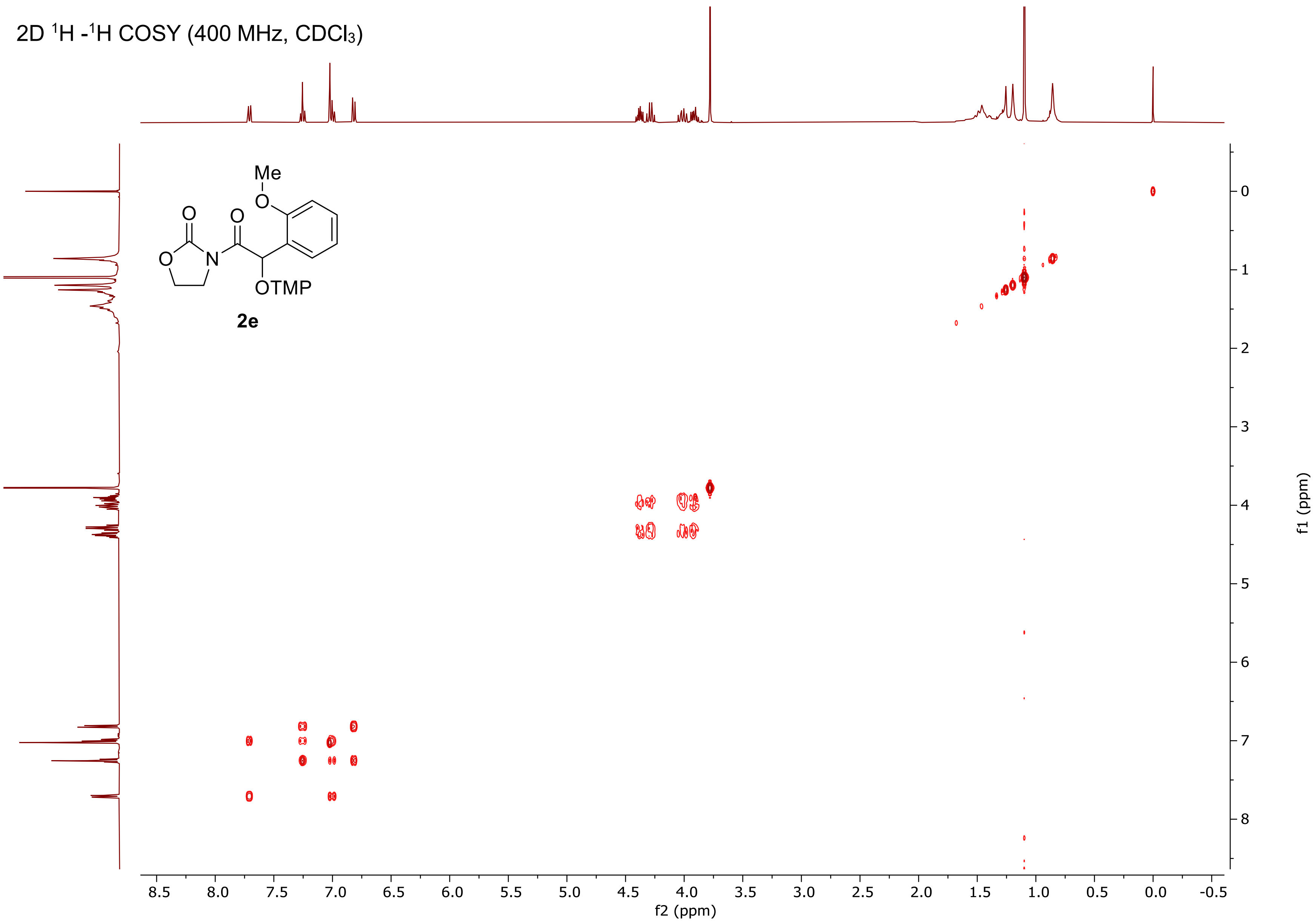

2D $^{1}H$ -$^{13}C$ HSQC (400 MHz, $CDCl_3$)

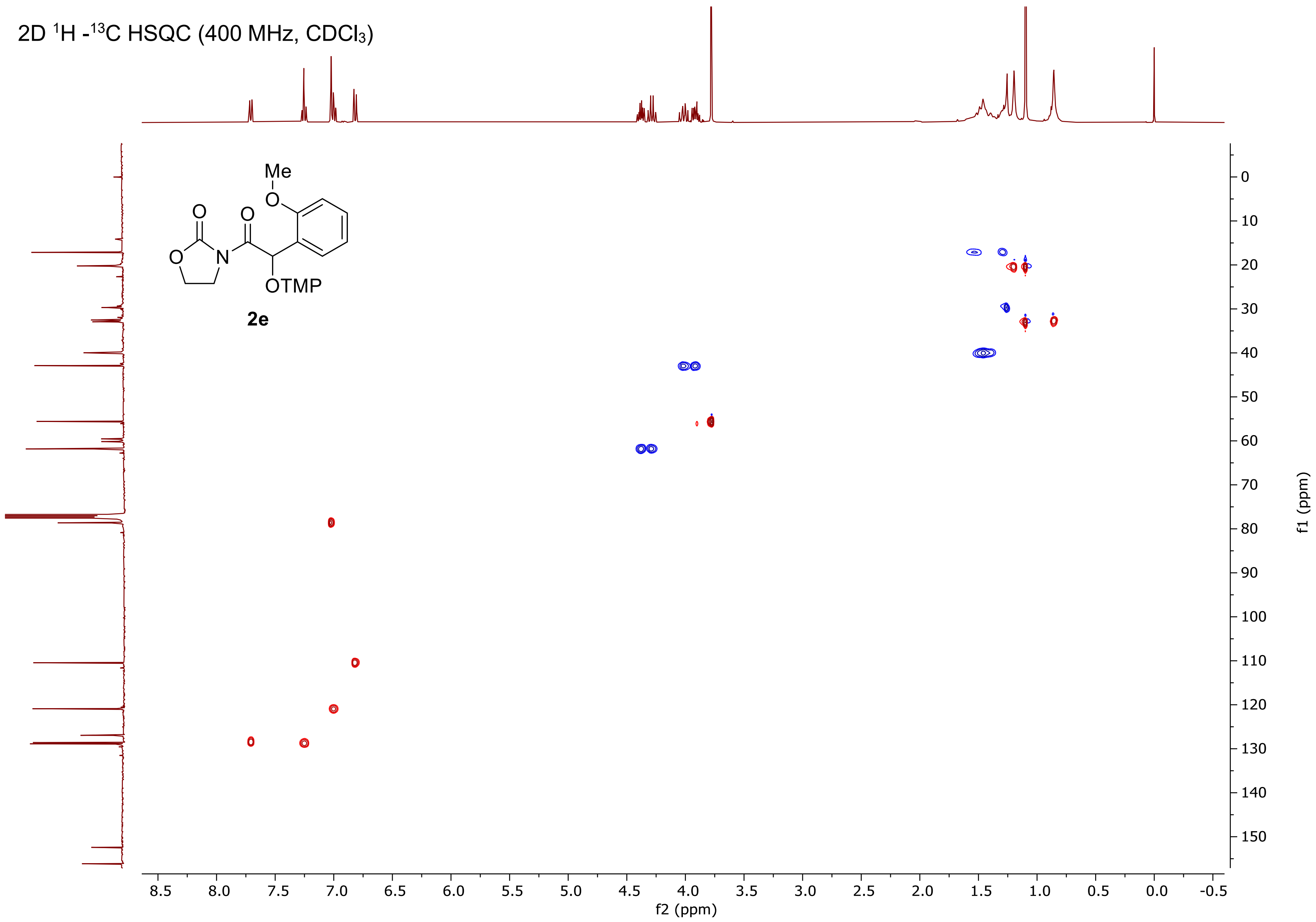

[1]H NMR (400 MHz, $CDCl_3$)

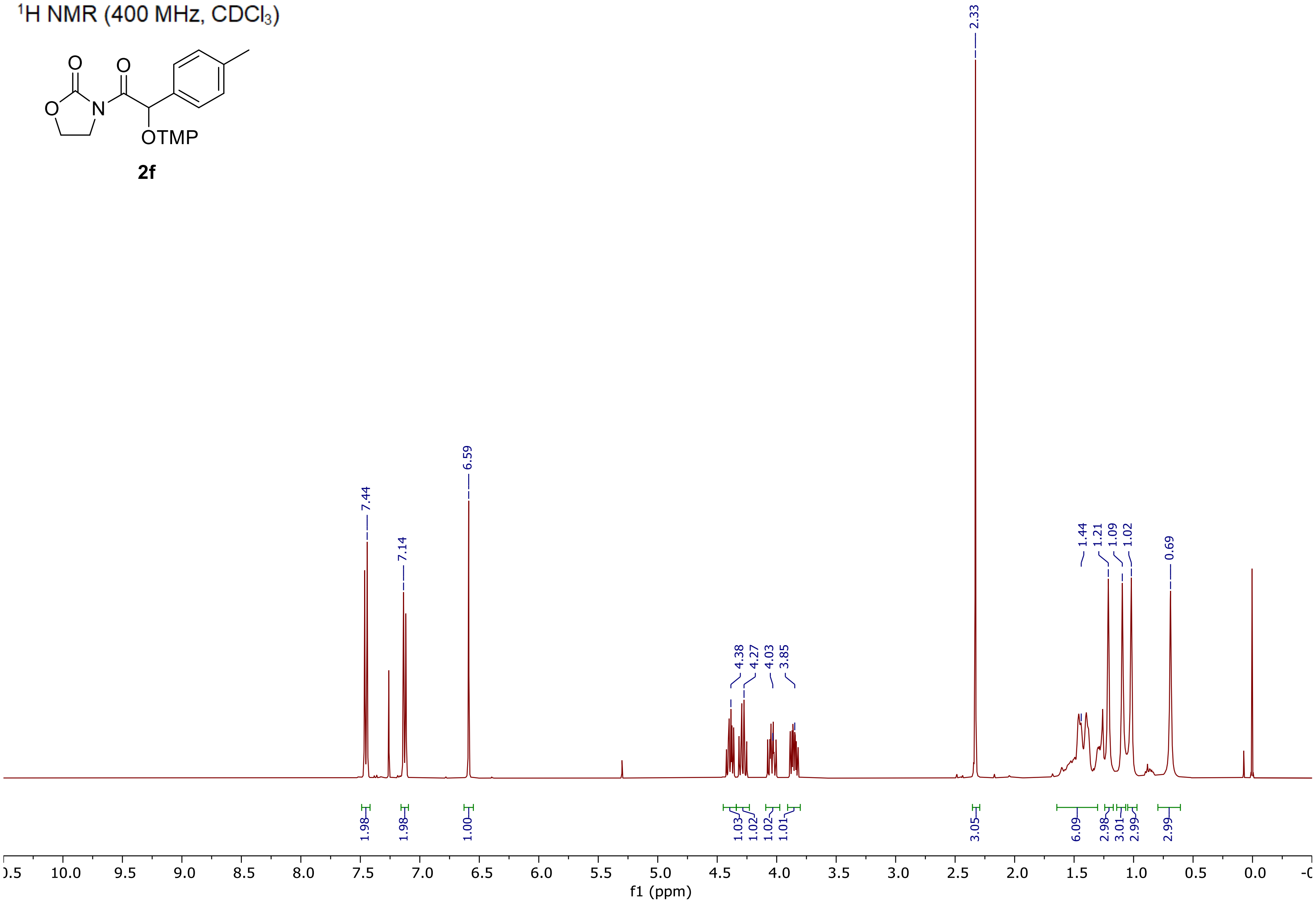


**2f**

$^{13}C\{^1H\}$ NMR (101 MHz, $CDCl_3$)

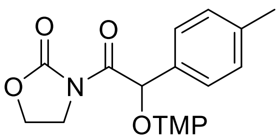

**2f**

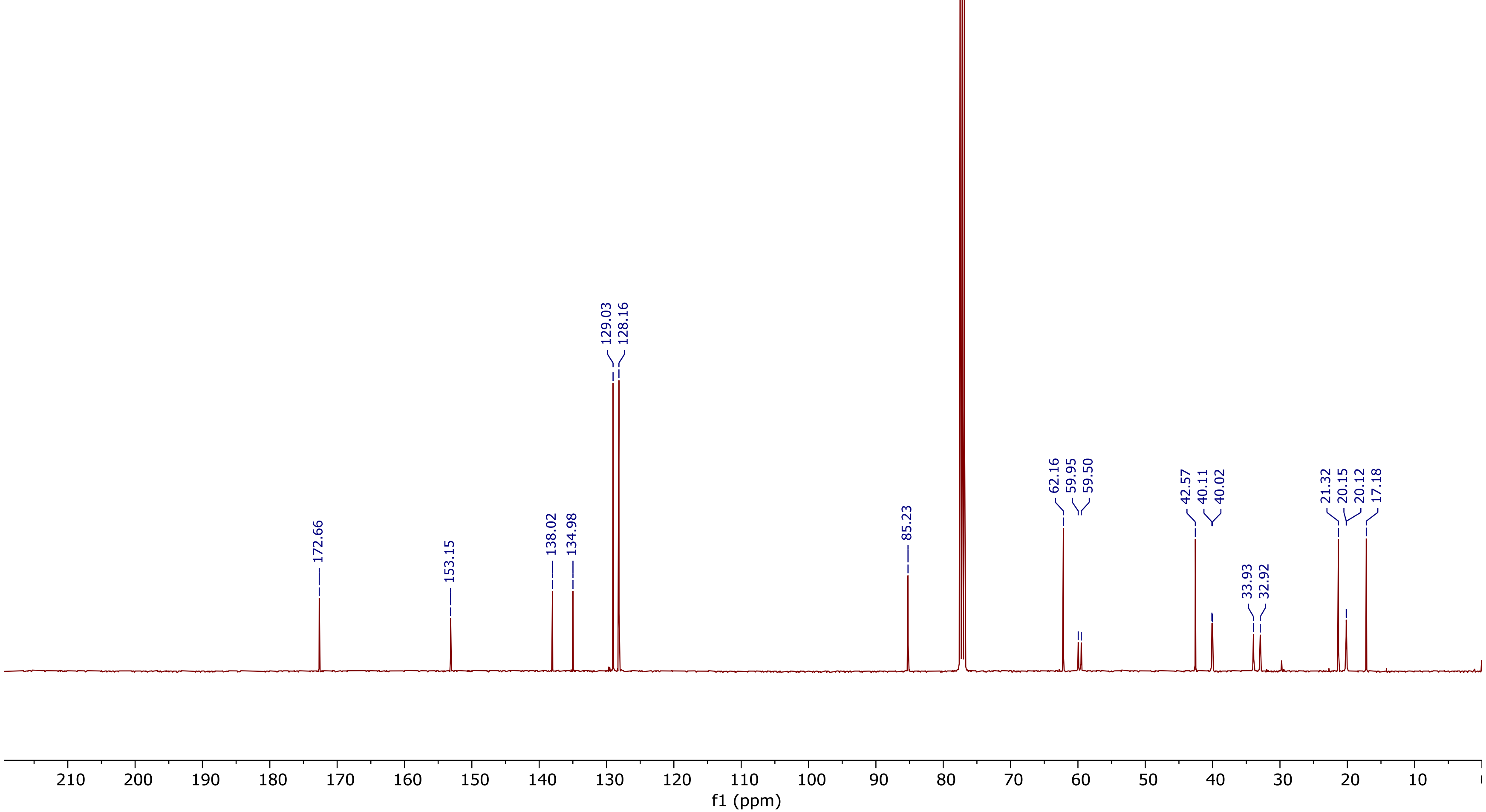

2D $^{1}H$ -$^{1}H$ COSY (400 MHz, $CDCl_3$)

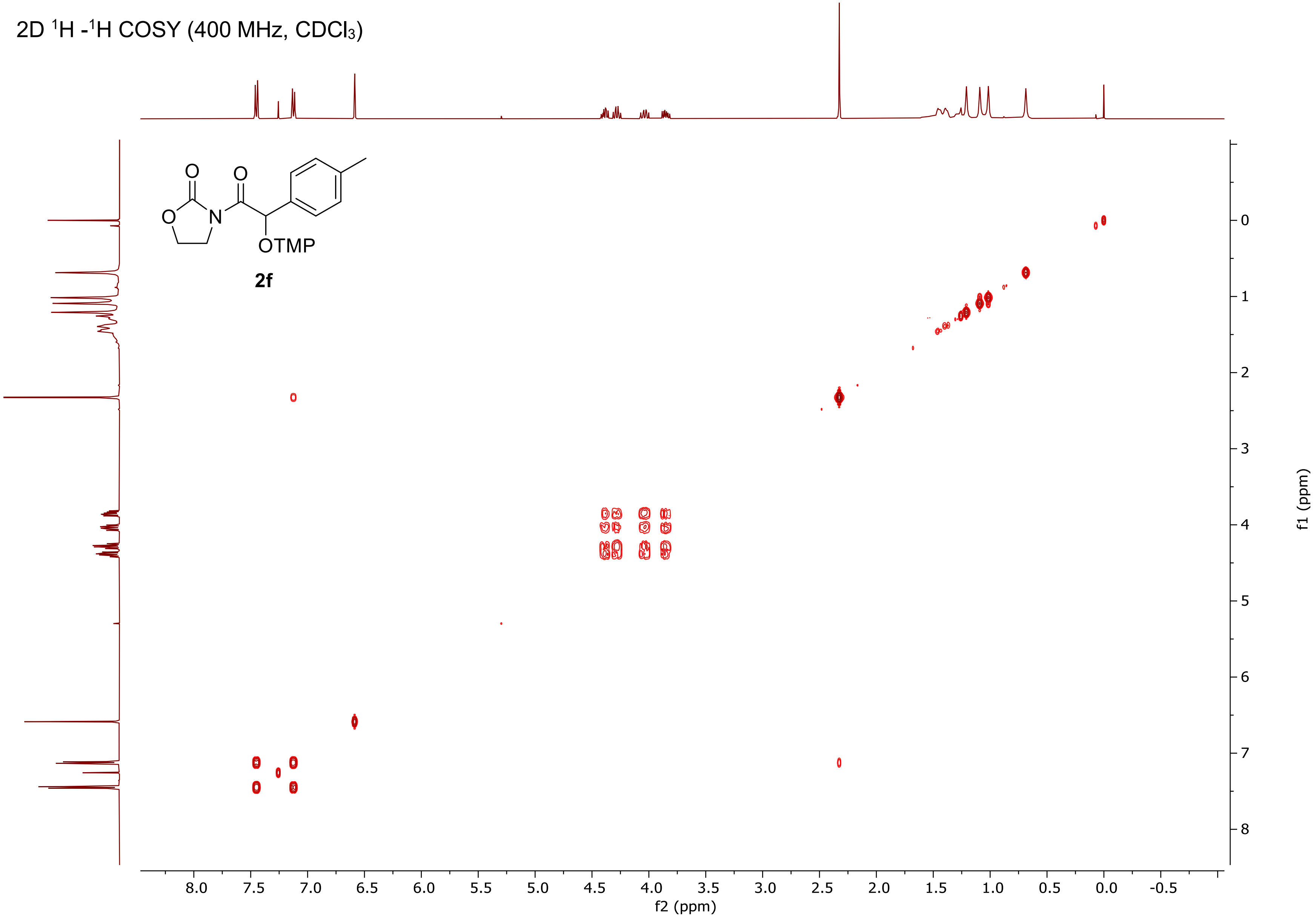

2D $^{1}H$ -$^{13}C$ HSQC (400 MHz, $CDCl_3$)

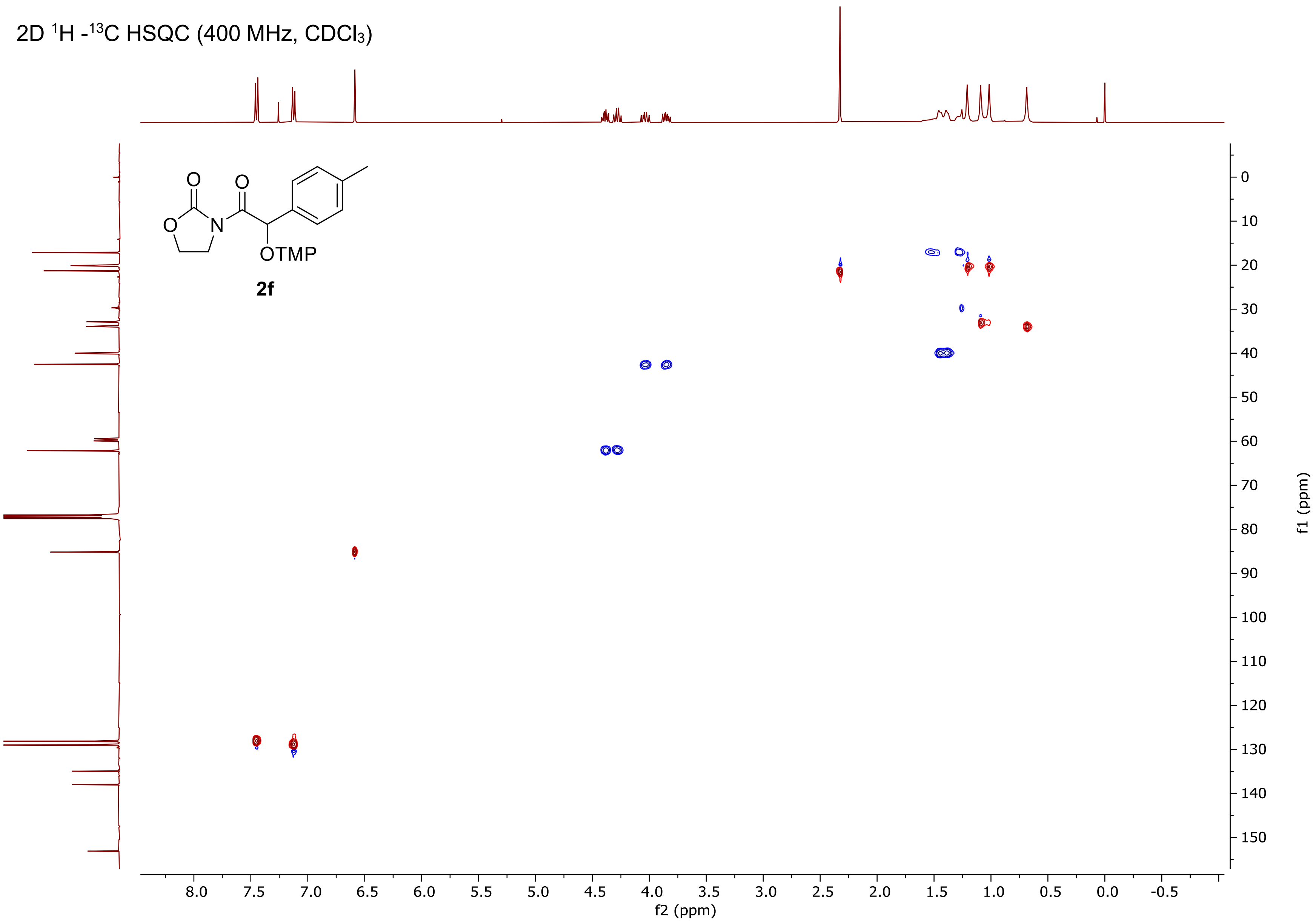

$^{1}H$ NMR (500 MHz, $CDCl_3$)

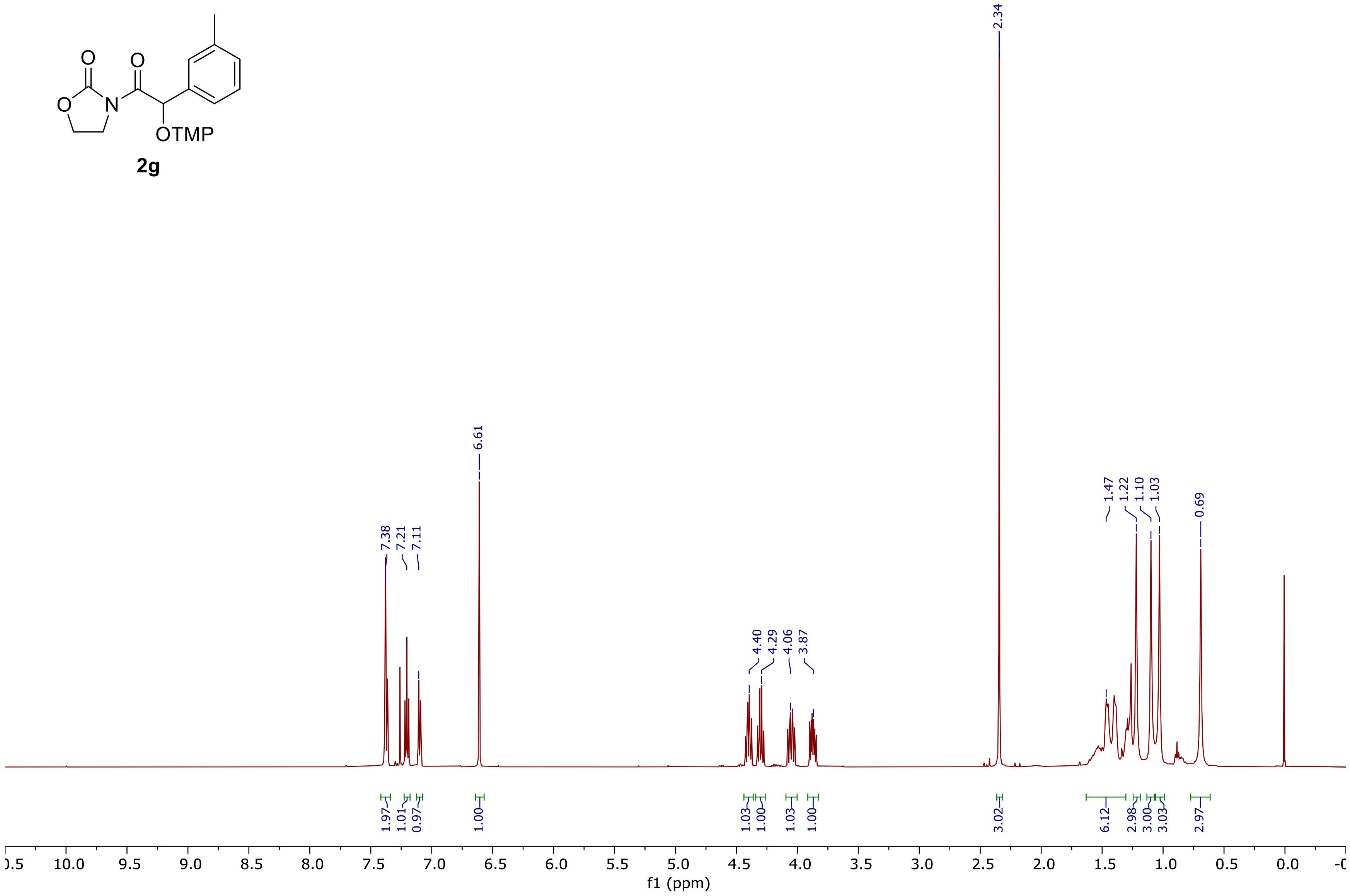

$^{13}C\{^{1}H\}$ NMR (126 MHz, $CDCl_3$)

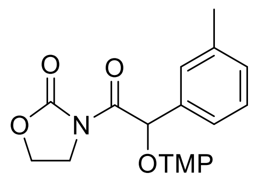


**2g**

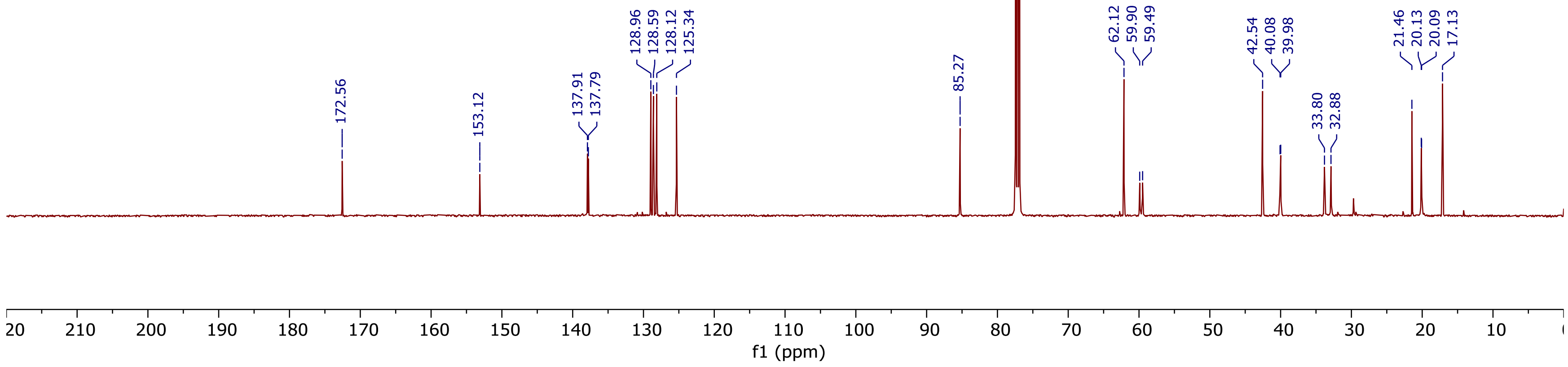

2D $^1H$ -$^1H$ COSY (500 MHz, $CDCl_3$)

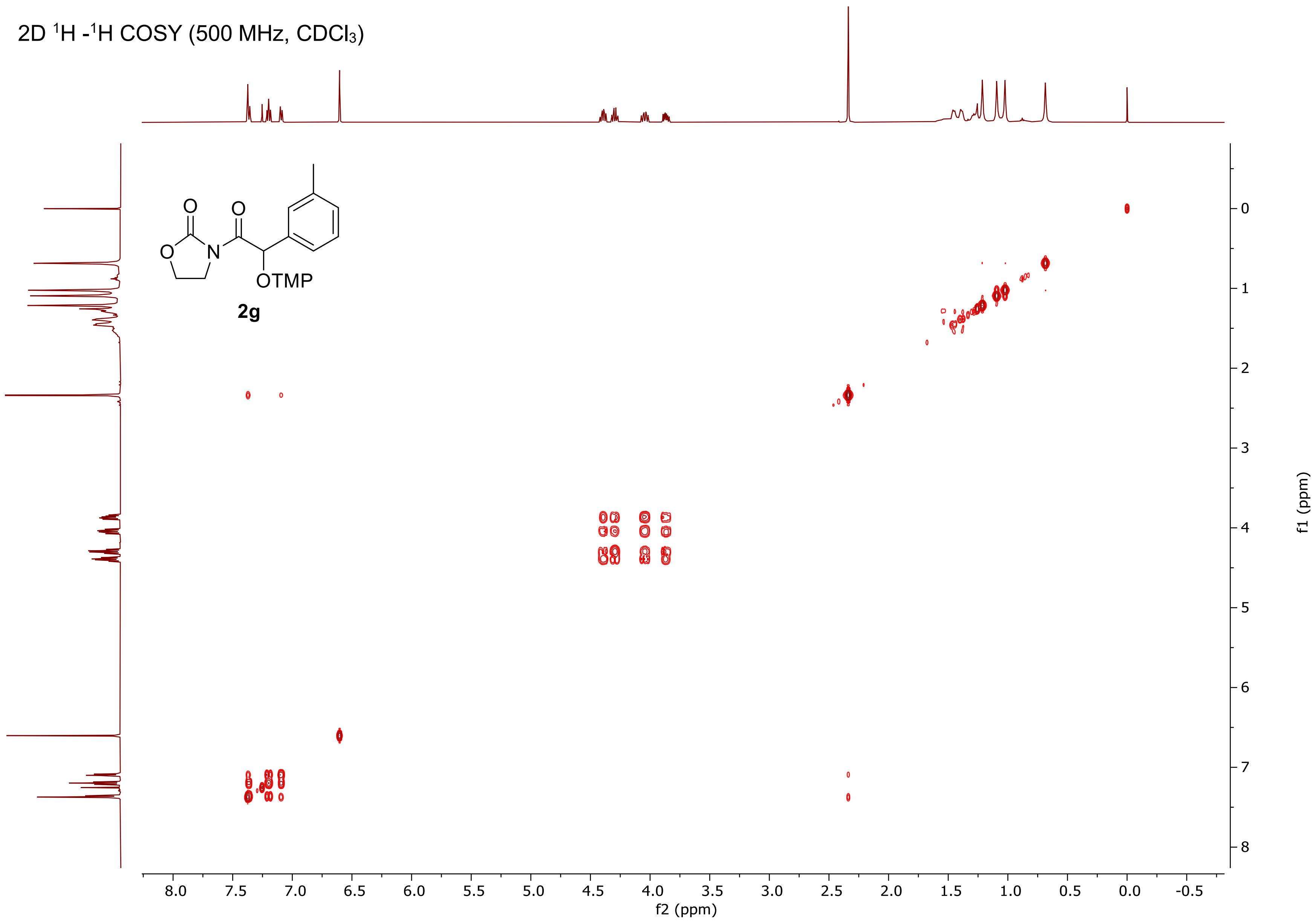

2D $^{1}H$ -$^{13}C$ HSQC (500 MHz, $CDCl_3$)

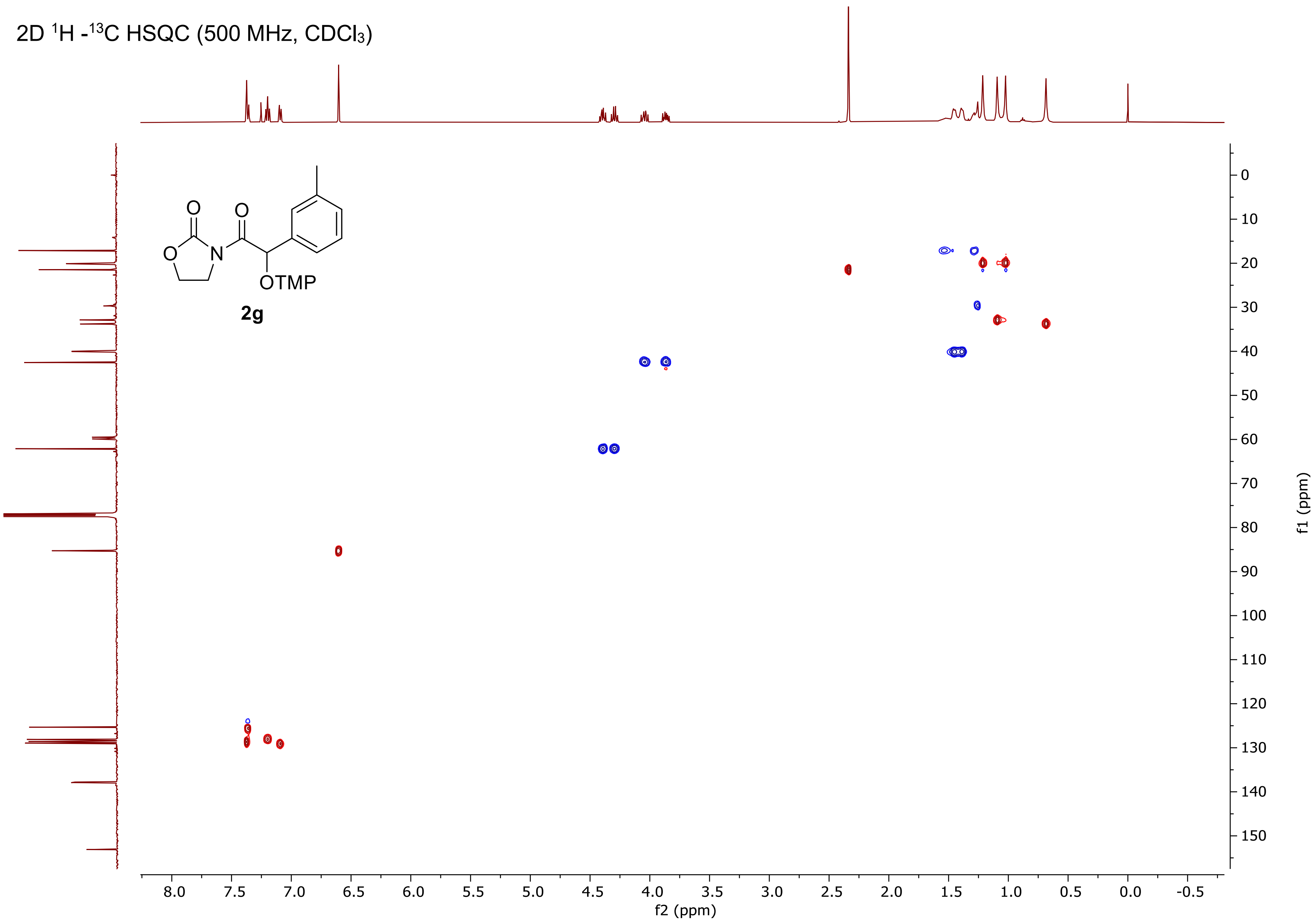

[1]H NMR (400 MHz, $CDCl_3$)

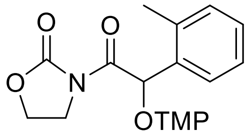


**2h**

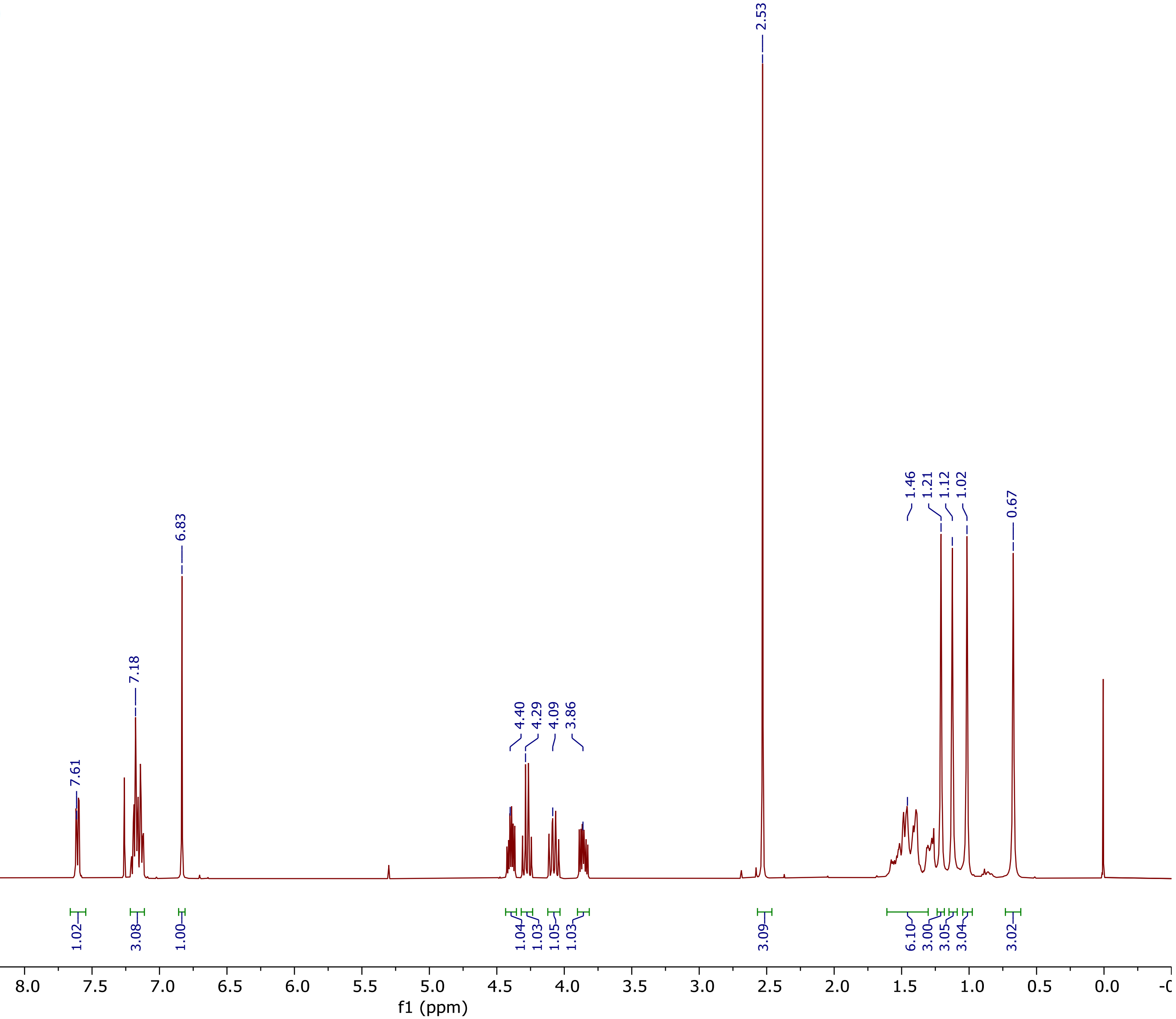

$^{13}C\{^{1}H\}$ NMR (101 MHz, $CDCl_3$)

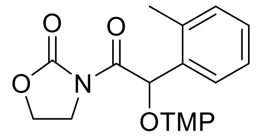


**2h**

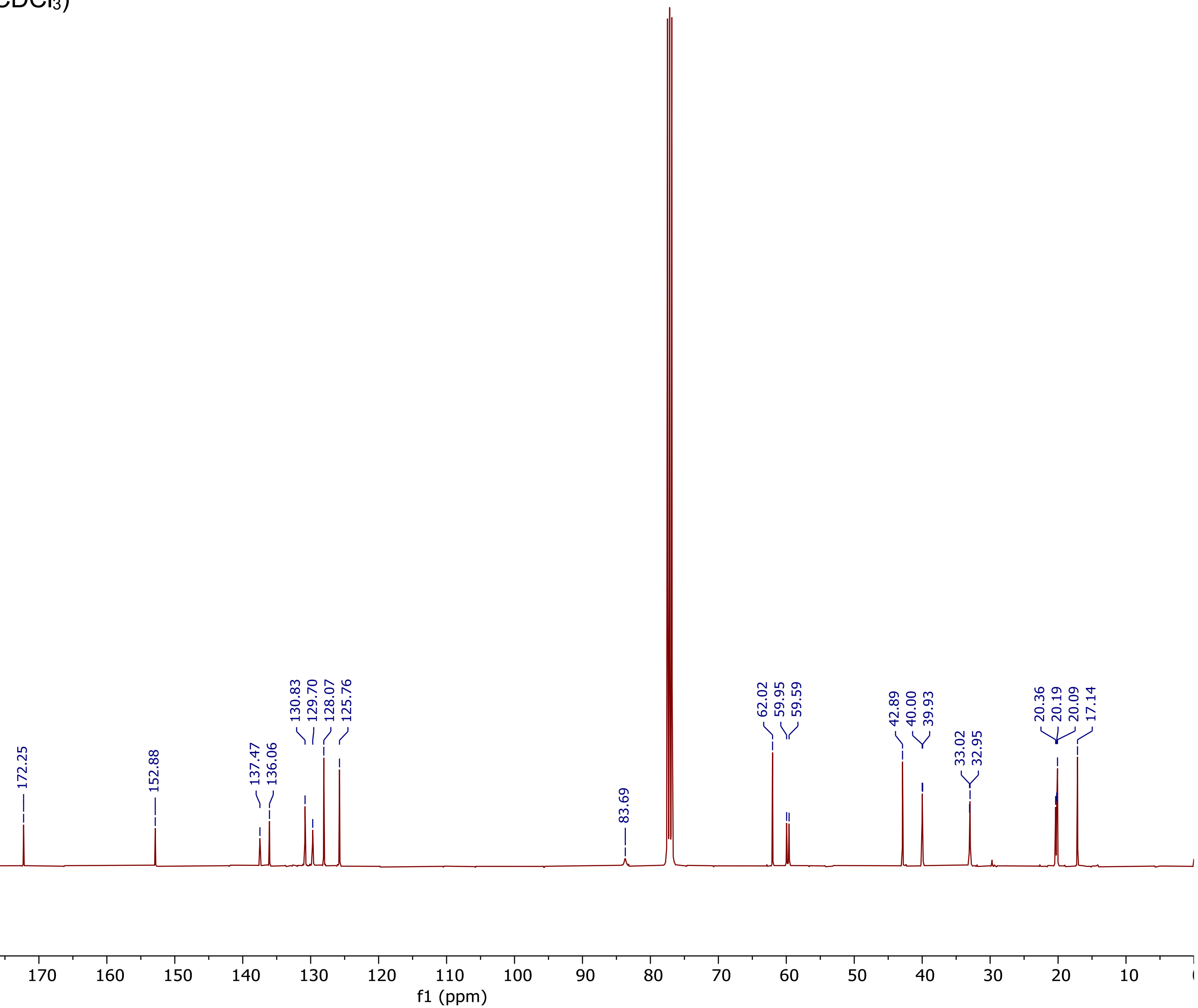

2D $^{1}H$ -$^{1}H$ COSY (400 MHz, $CDCl_3$)

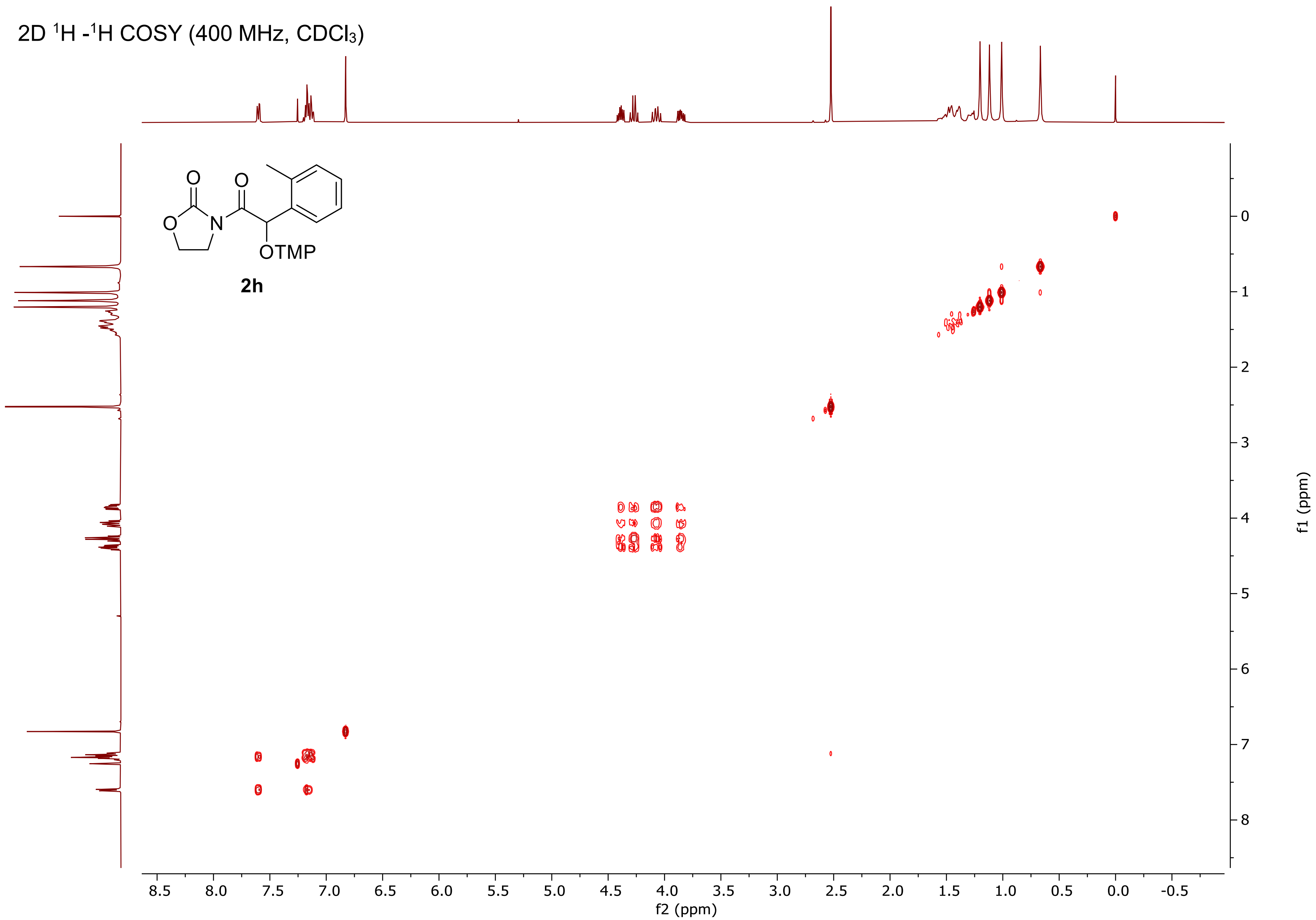

2D $^1$H -$^{13}$C HSQC (400 MHz, $CDCl_3$)

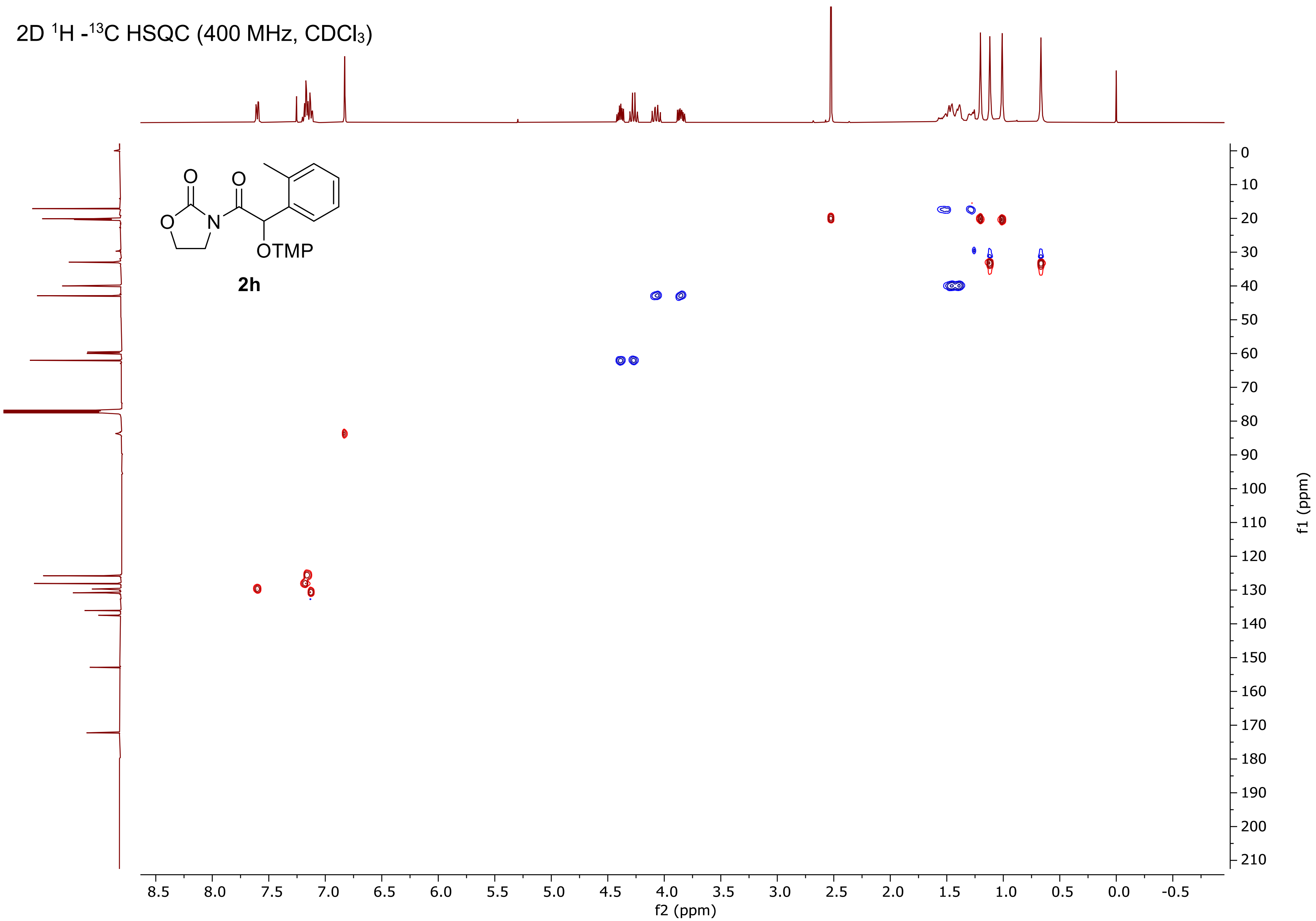

$^{1}H$ NMR (400 MHz, $CDCl_3$)

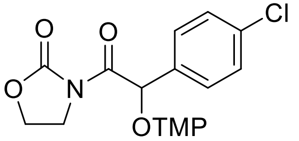


**2i**

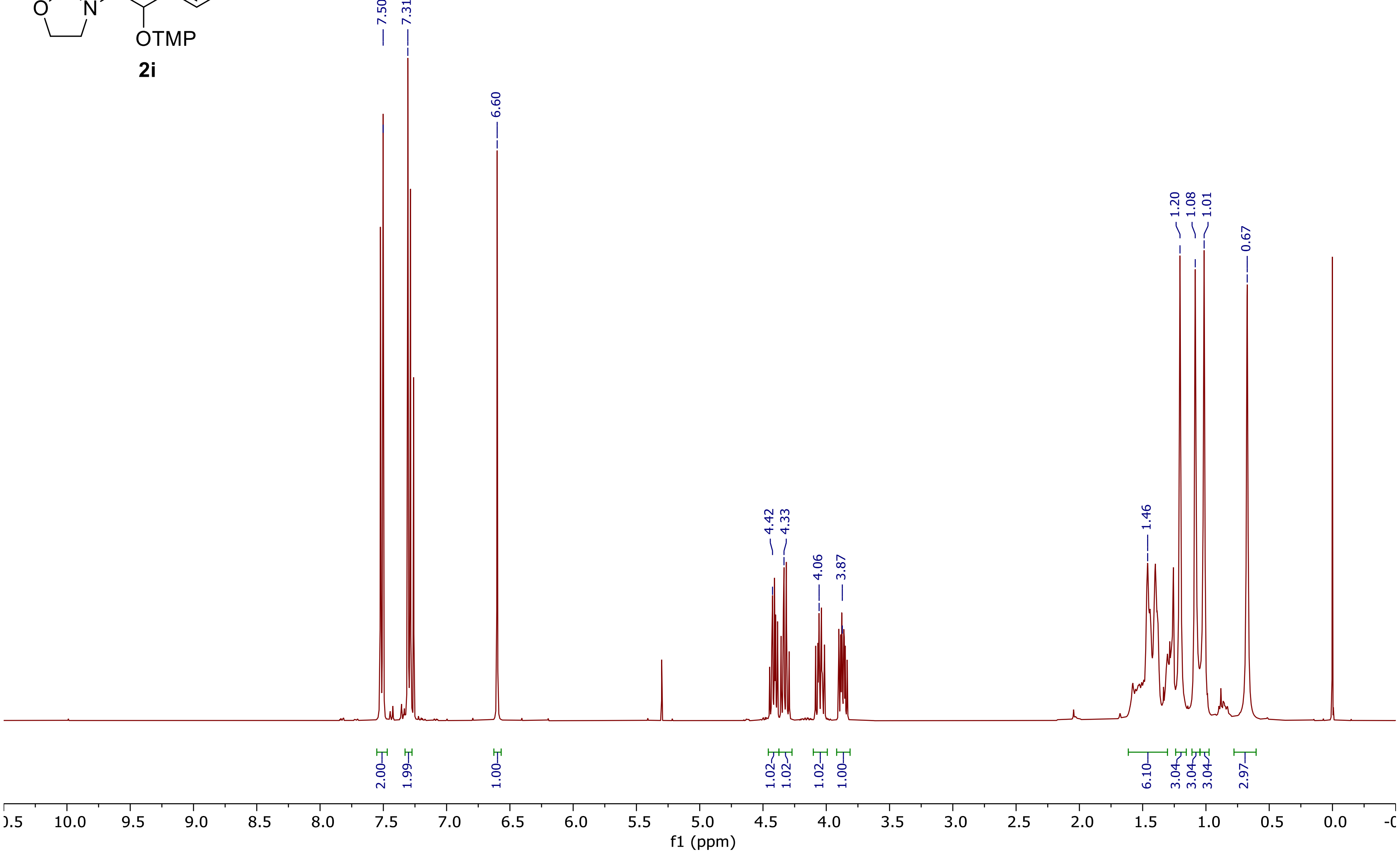

$^{13}C\{^{1}H\}$ NMR (101 MHz, $CDCl_3$)

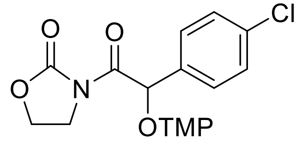

**2i**

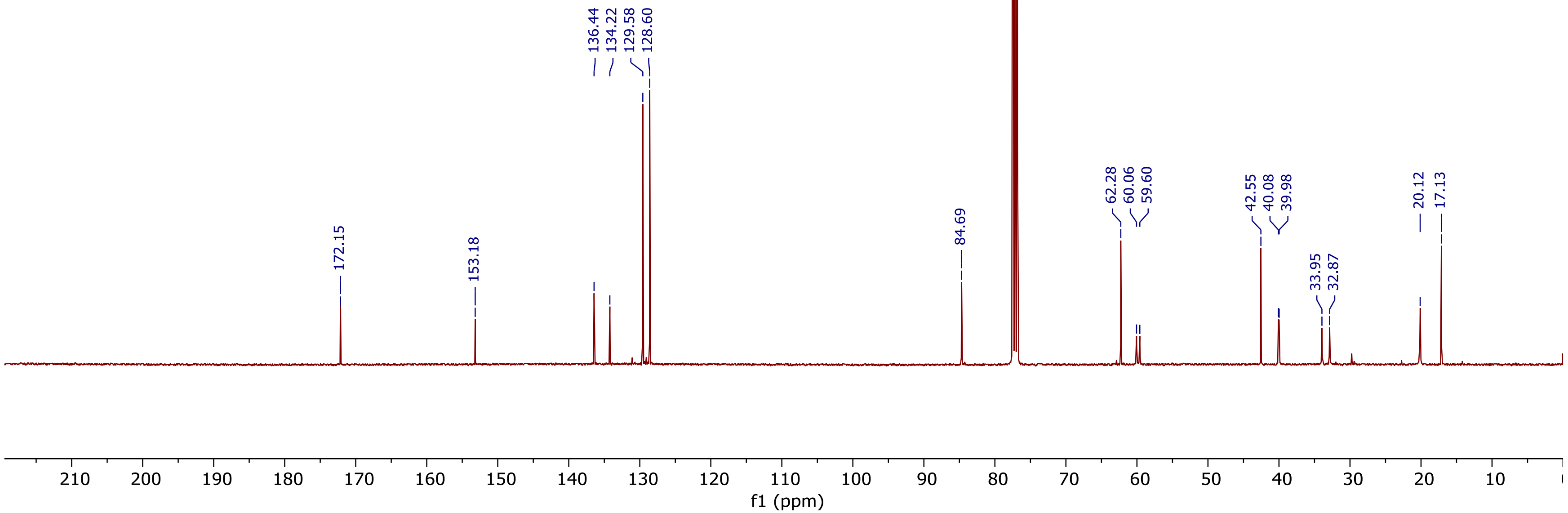

2D $^1H$ -$^1H$ COSY (400 MHz, $CDCl_3$)

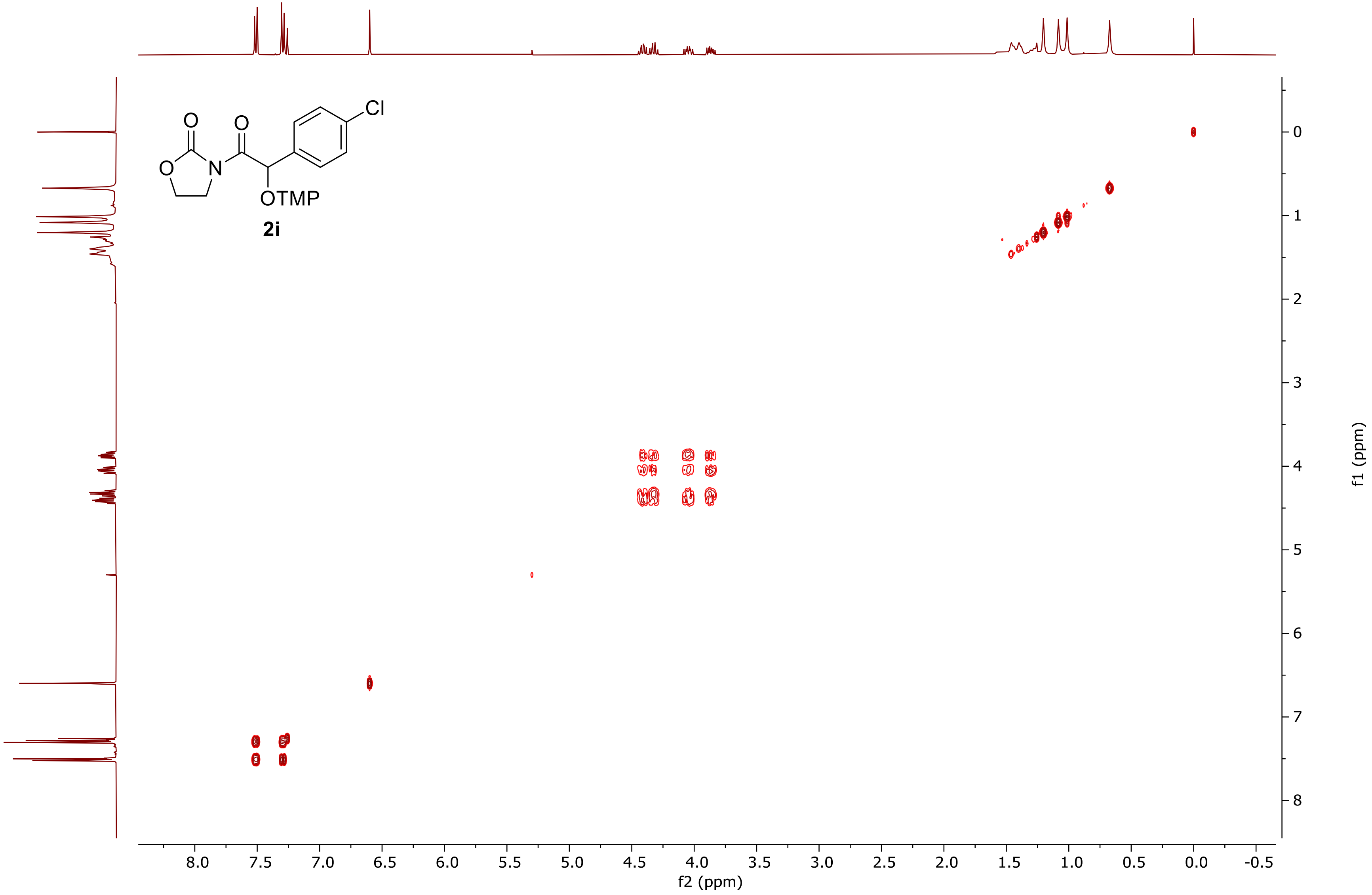

2D $^{1}H$ -$^{13}C$ HSQC (400 MHz, $CDCl_3$)

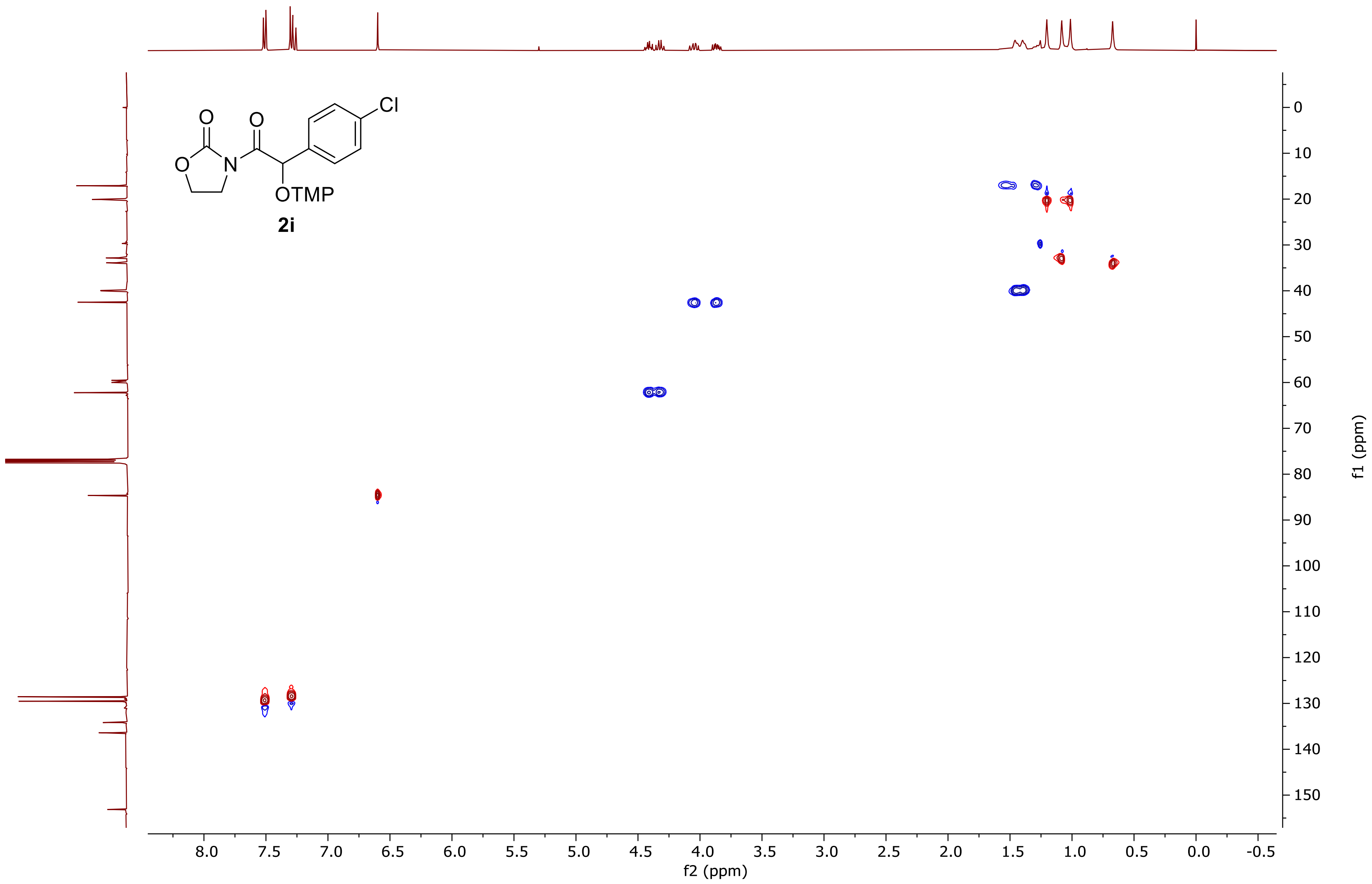

[1]H NMR (400 MHz, $CDCl_3$)

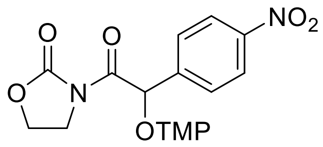

**2j**

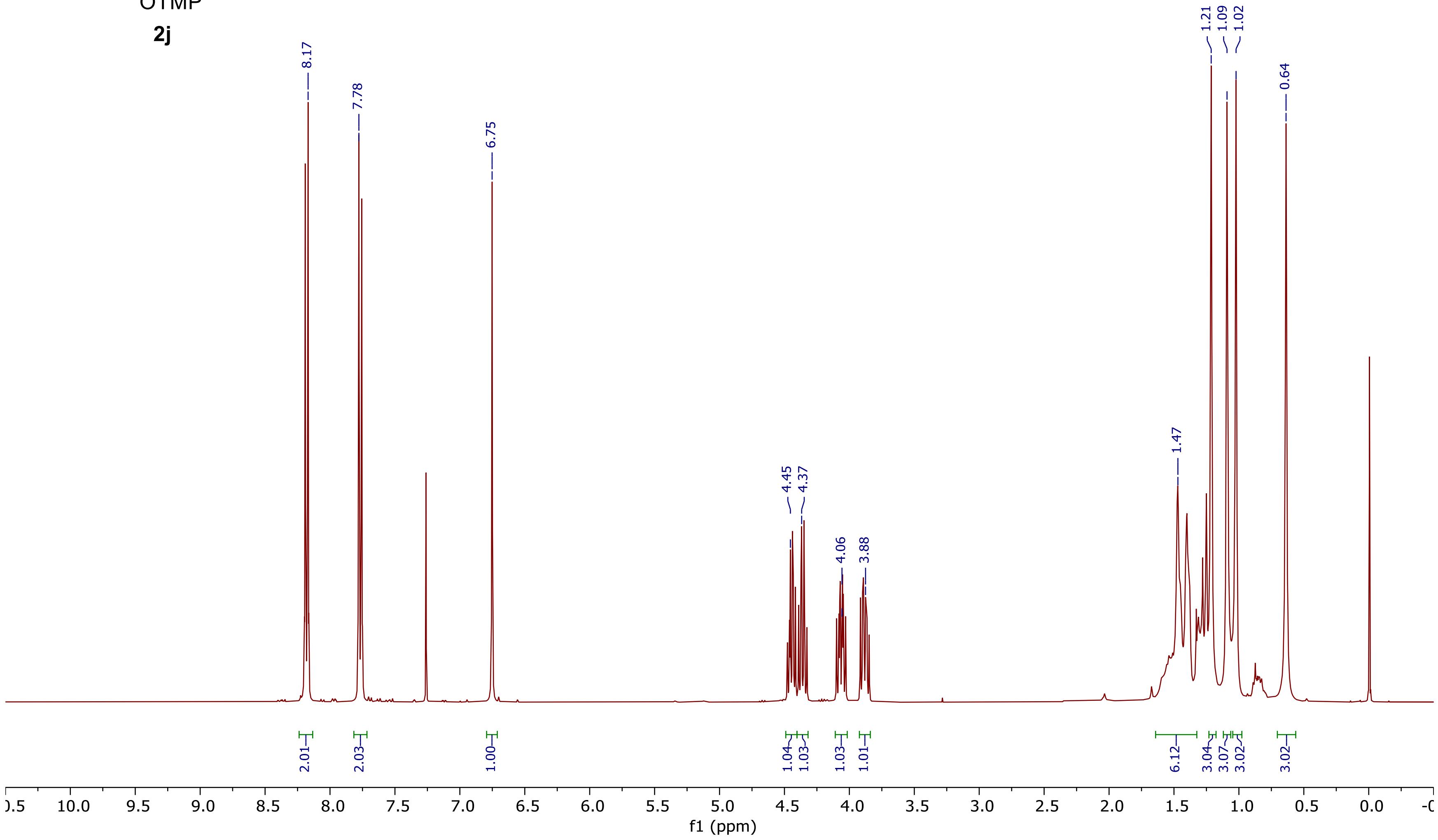

$^{13}C\{^1H\}$ NMR (101 MHz, $CDCl_3$)

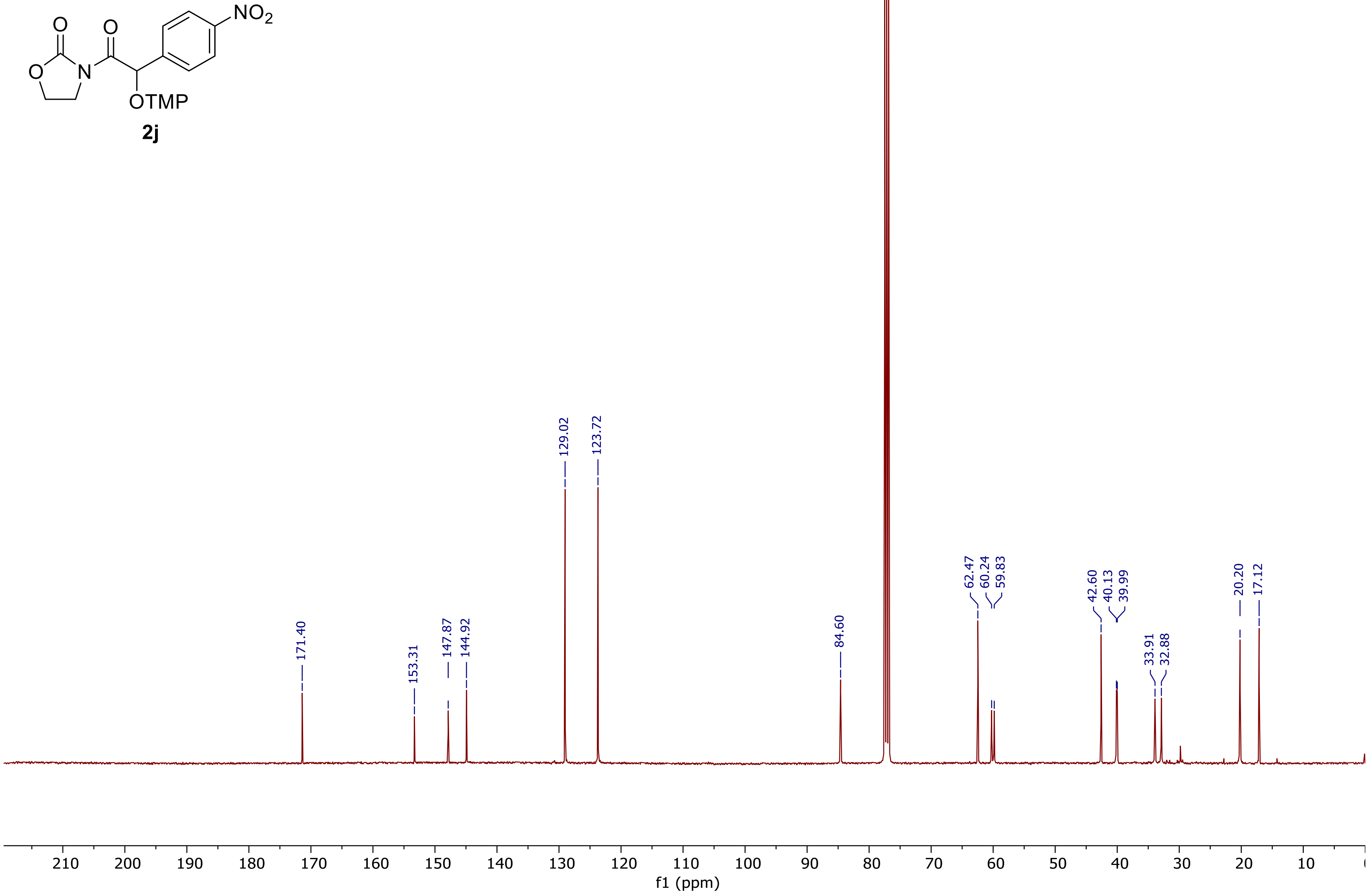

2D $^1$H -$^1$H COSY (400 MHz, $CDCl_3$)

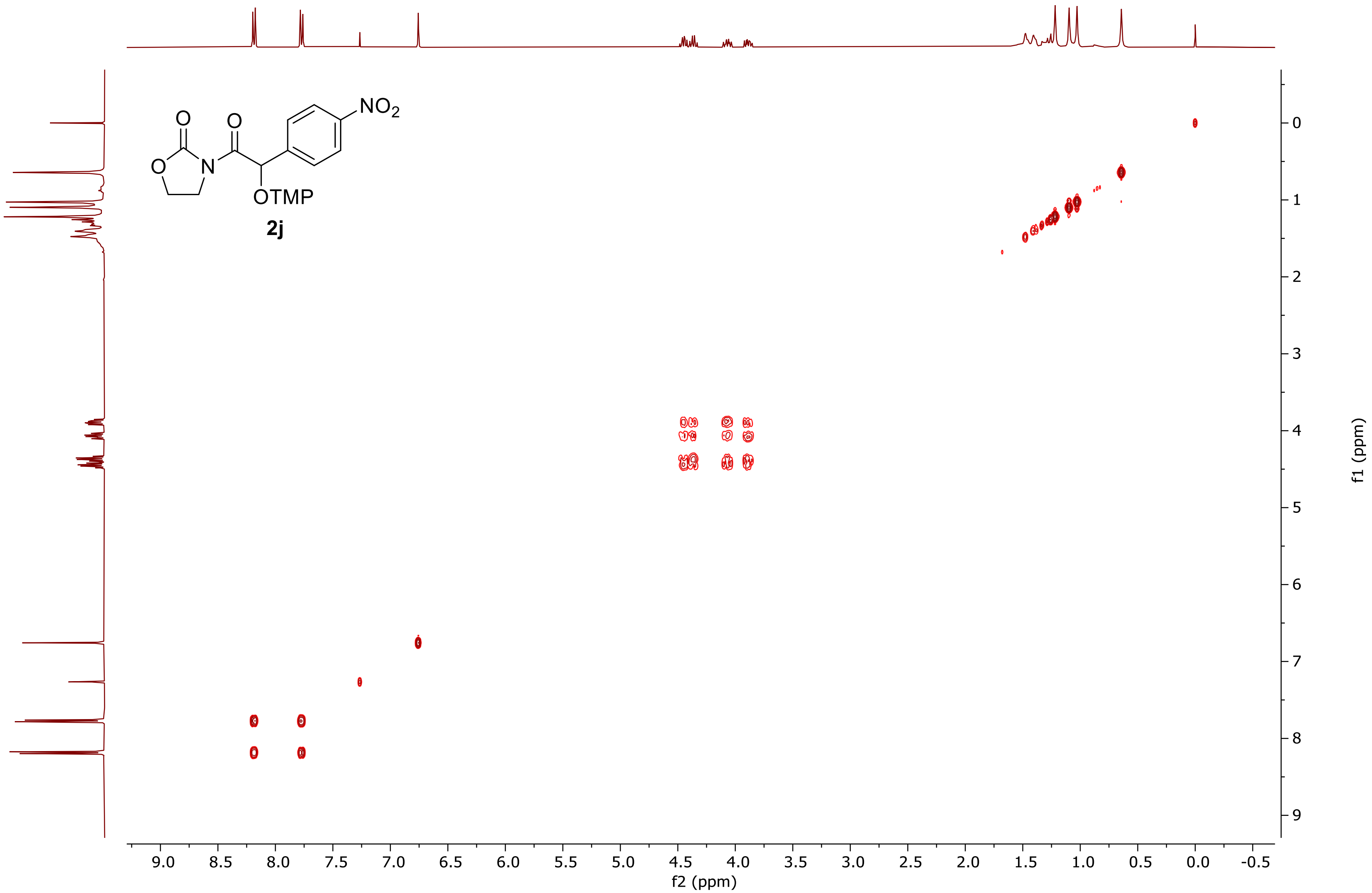

2D $^{1}$H -$^{13}$C HSQC (400 MHz, $CDCl_3$)

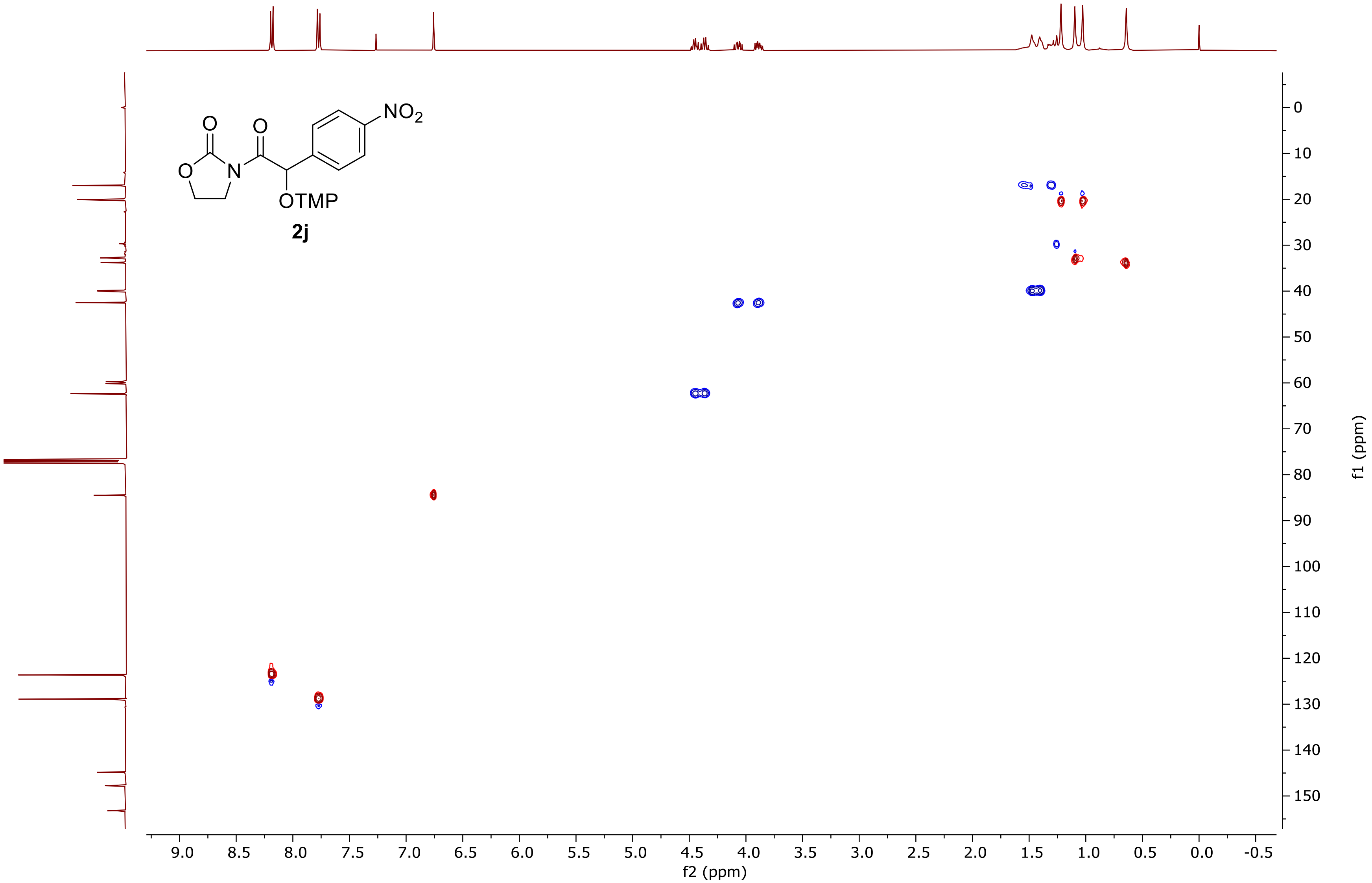

$^1H$ NMR (400 MHz, $CDCl_3$)

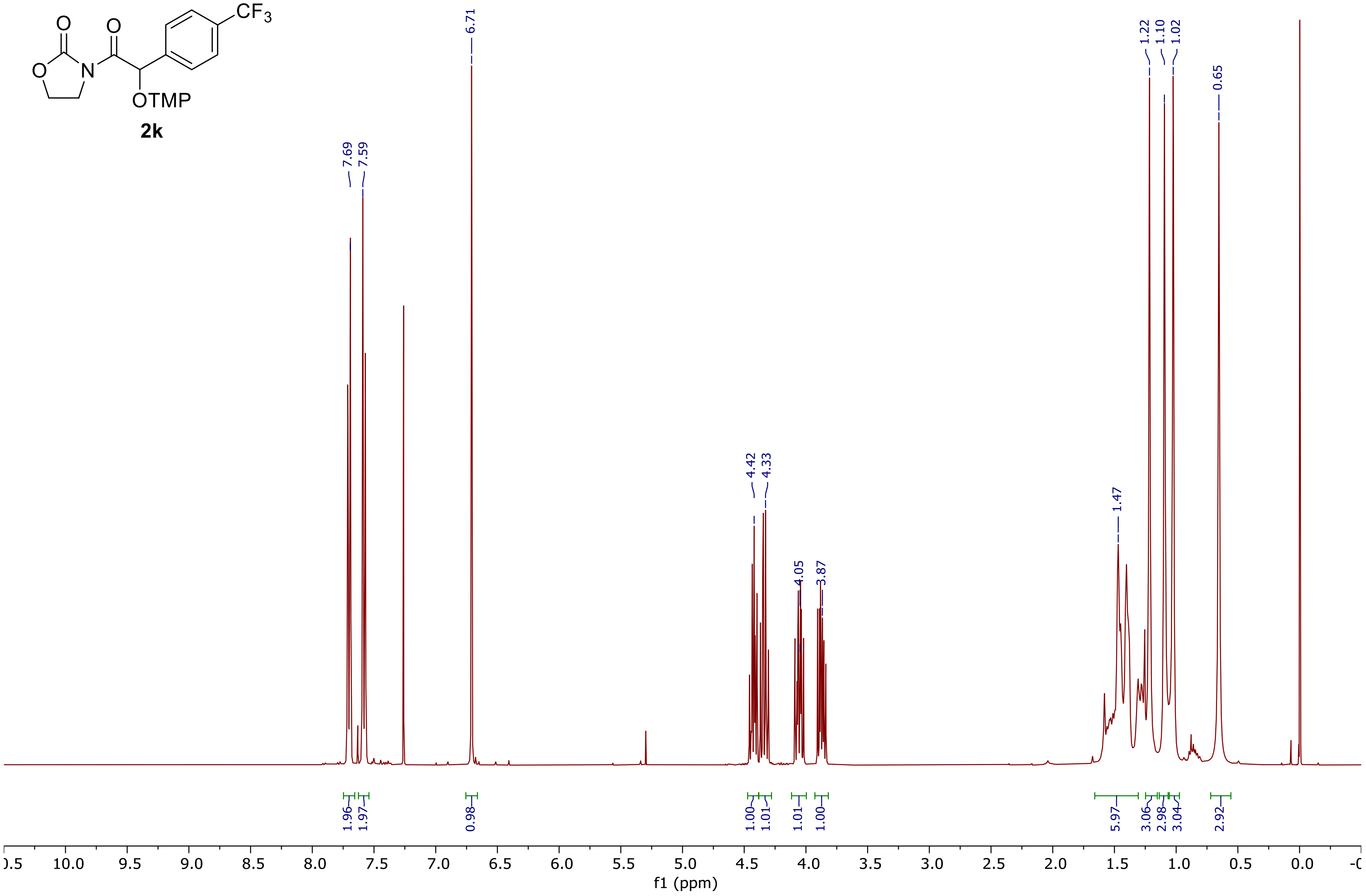

$^{13}C\{^1H\}$ NMR (101 MHz, $CDCl_3$)

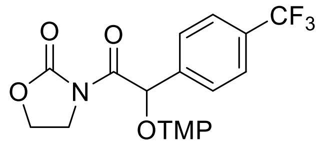


**2k**

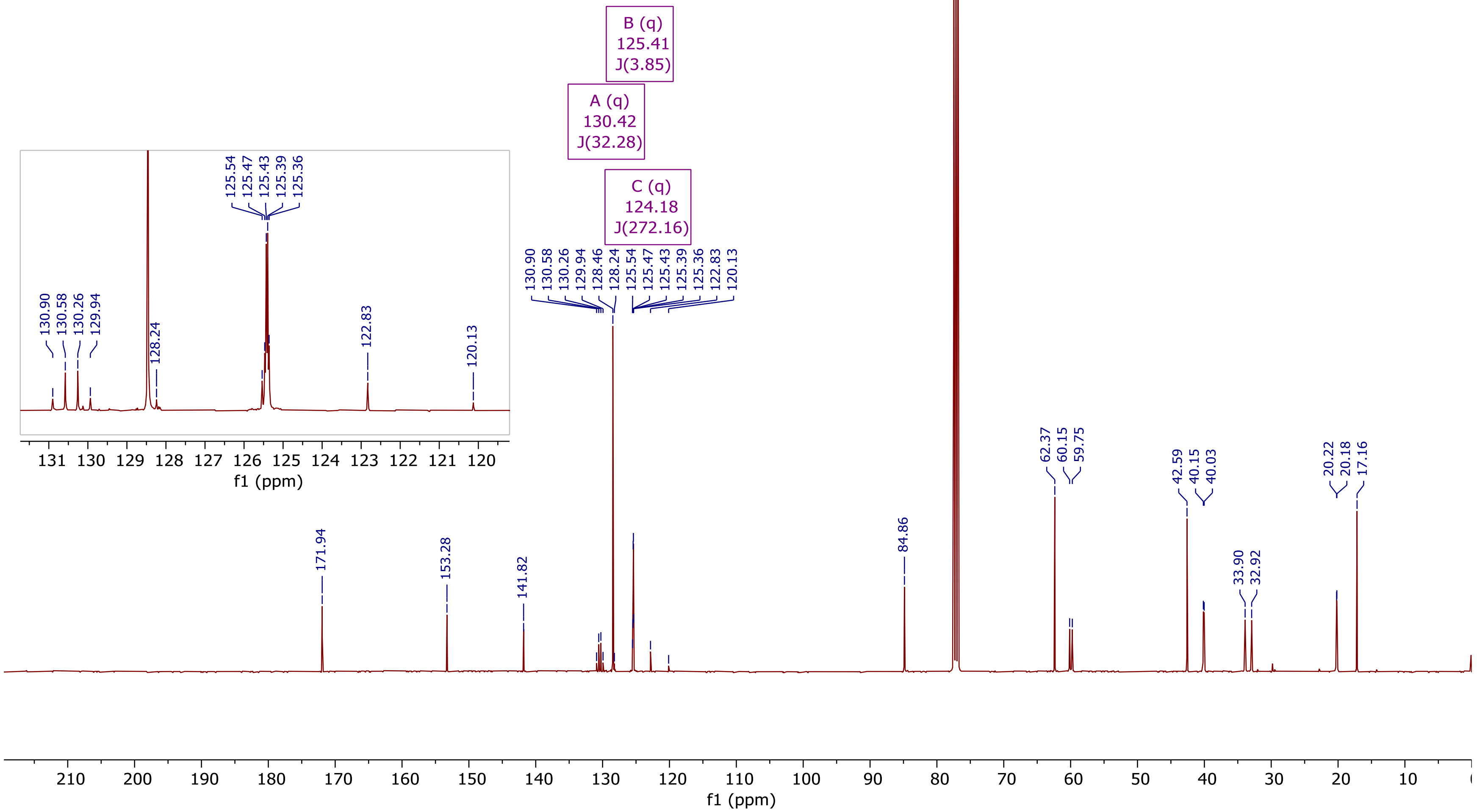

2D $^{1}H$ -$^{1}H$ COSY (400 MHz, $CDCl_3$)

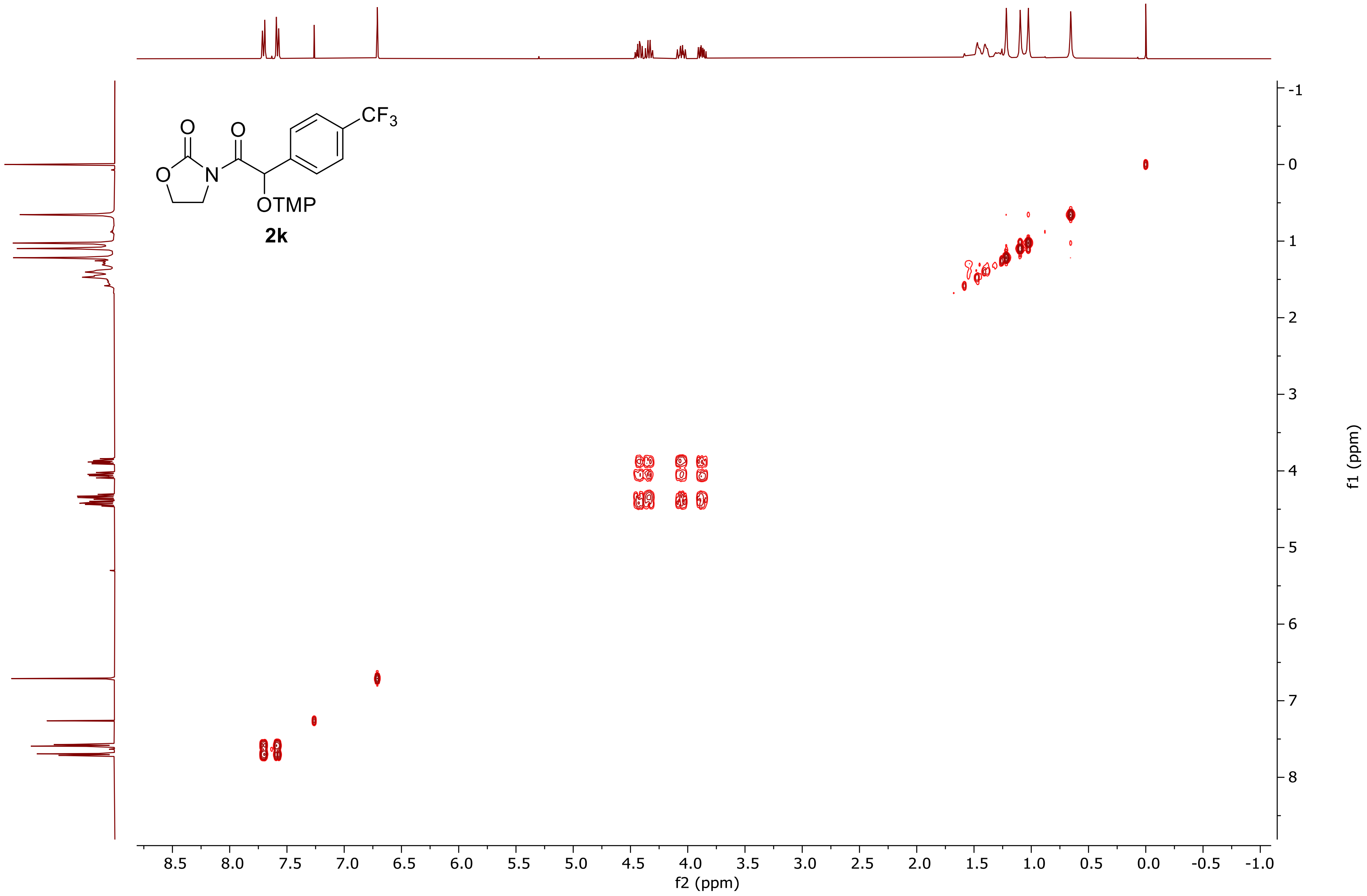

2D $^{1}H$ -$^{13}C$ HSQC (400 MHz, $CDCl_3$)

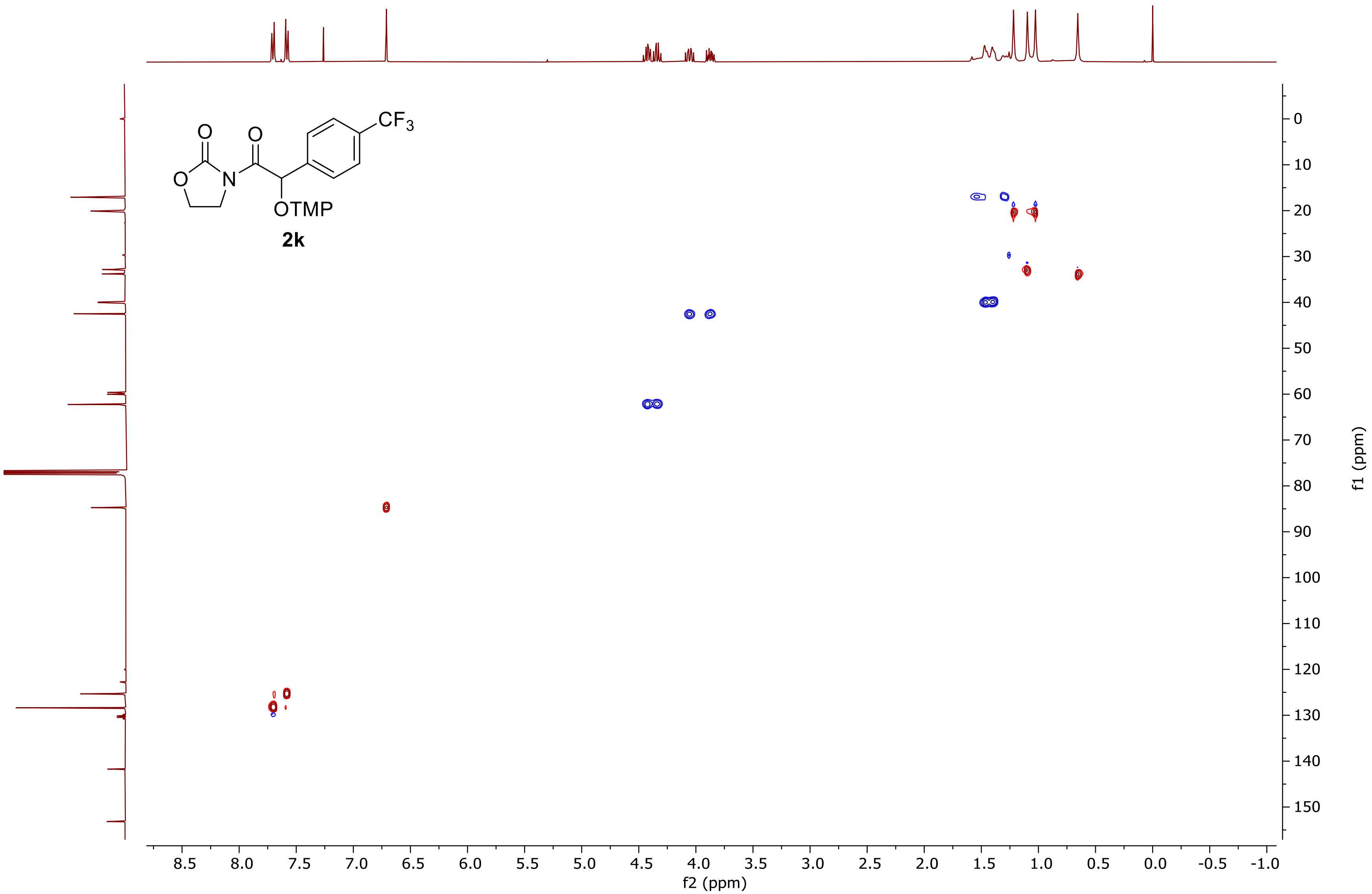

$^{1}H$ NMR (400 MHz, $CDCl_3$)

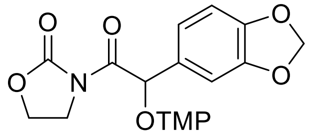

2l

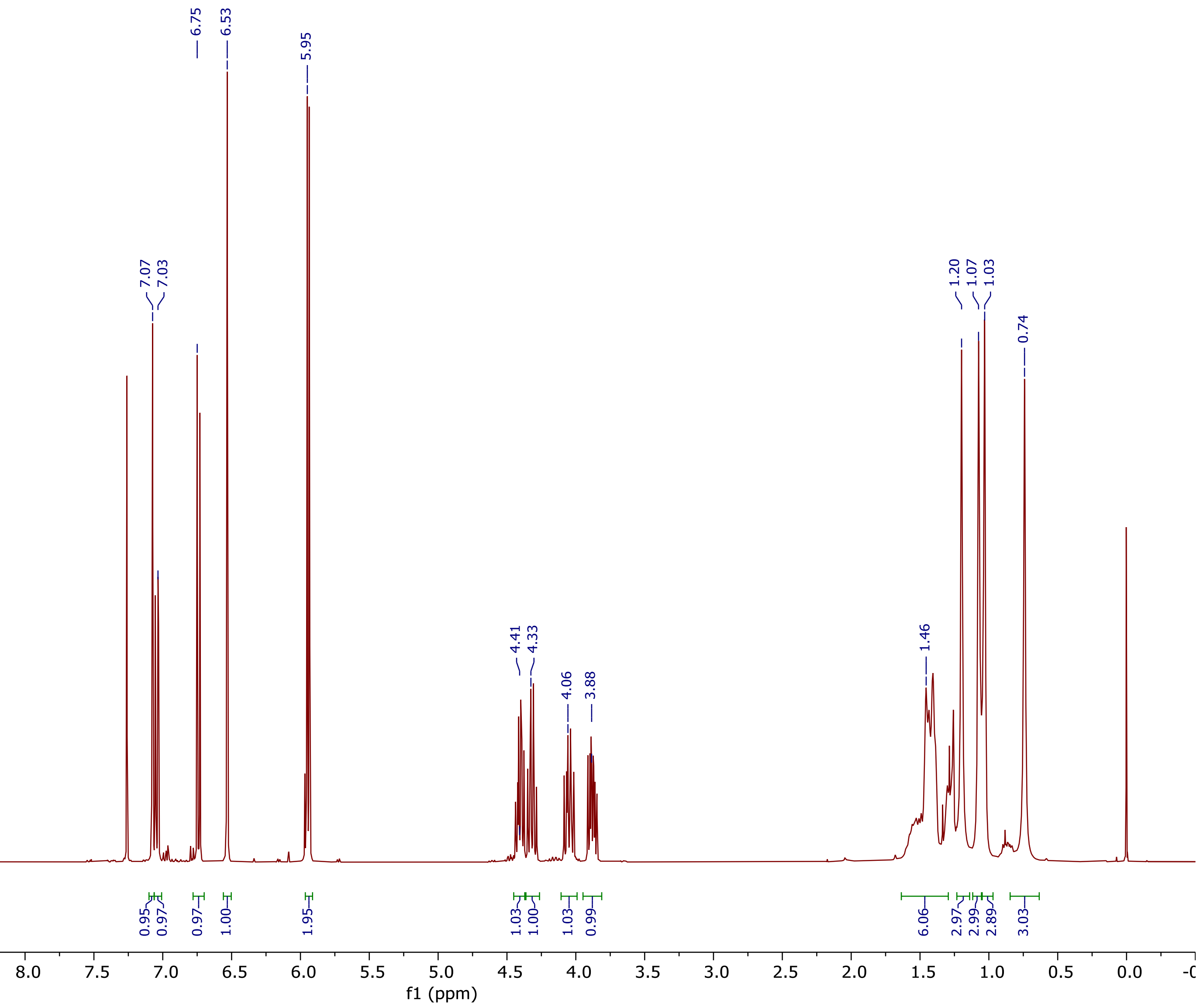

$^{13}C\{^{1}H\}$ NMR (101 MHz, $CDCl_3$)

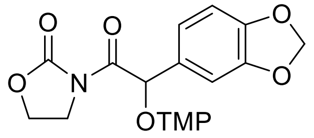


**2l**

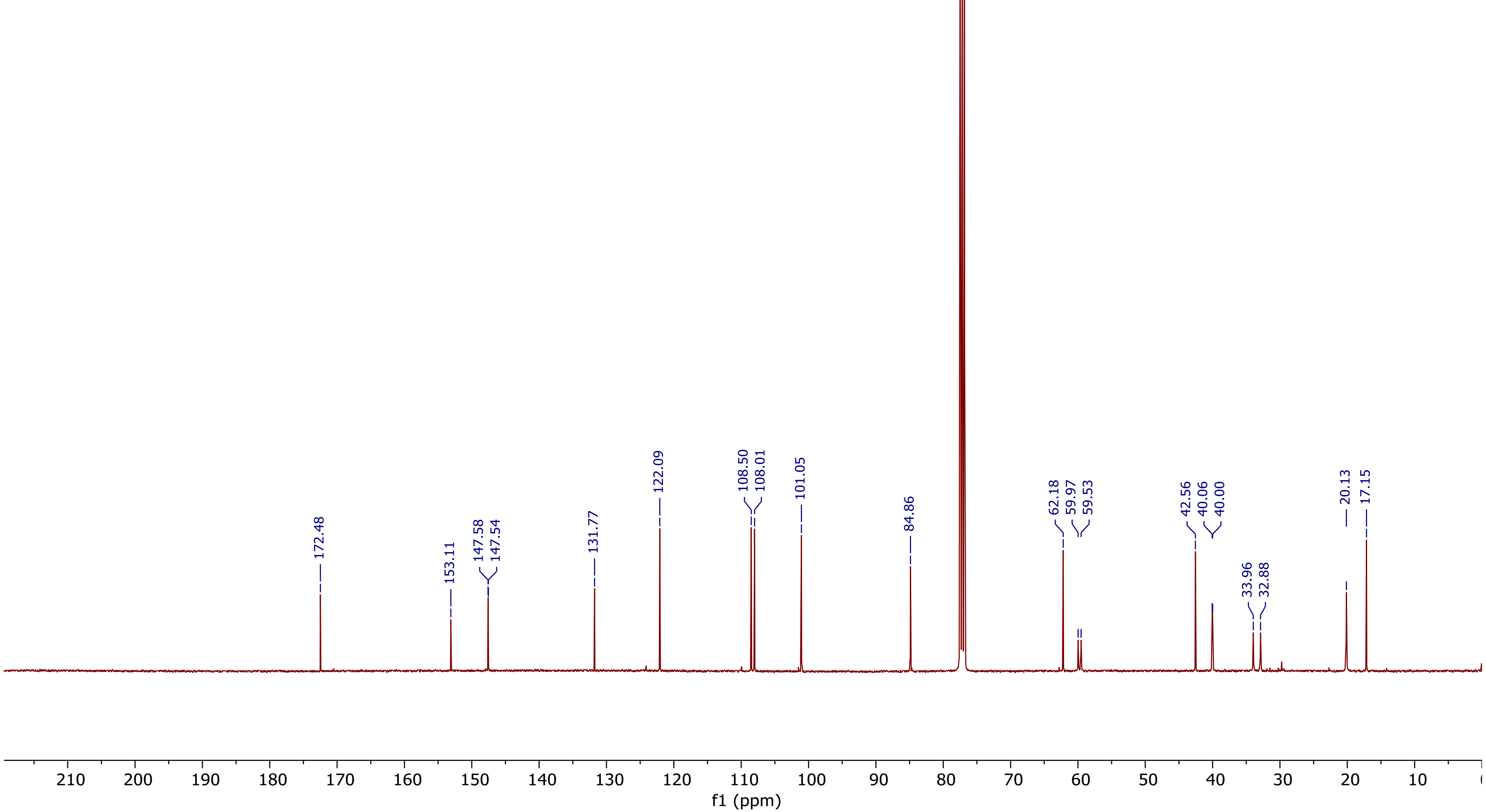

2D $^1H$ -$^1H$ COSY (400 MHz, $CDCl_3$)

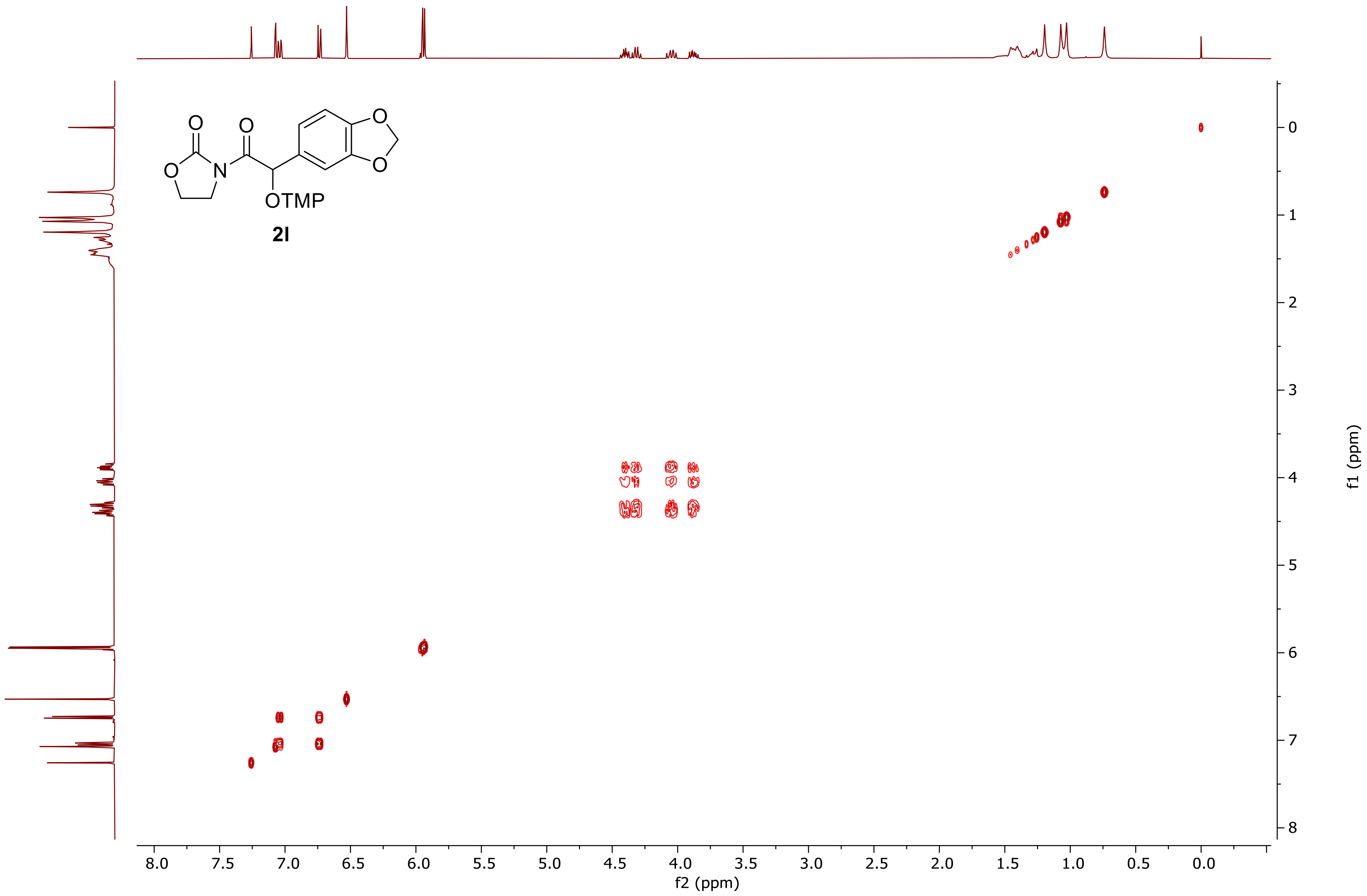

2D $^{1}H$ -$^{13}C$ HSQC (400 MHz, $CDCl_3$)

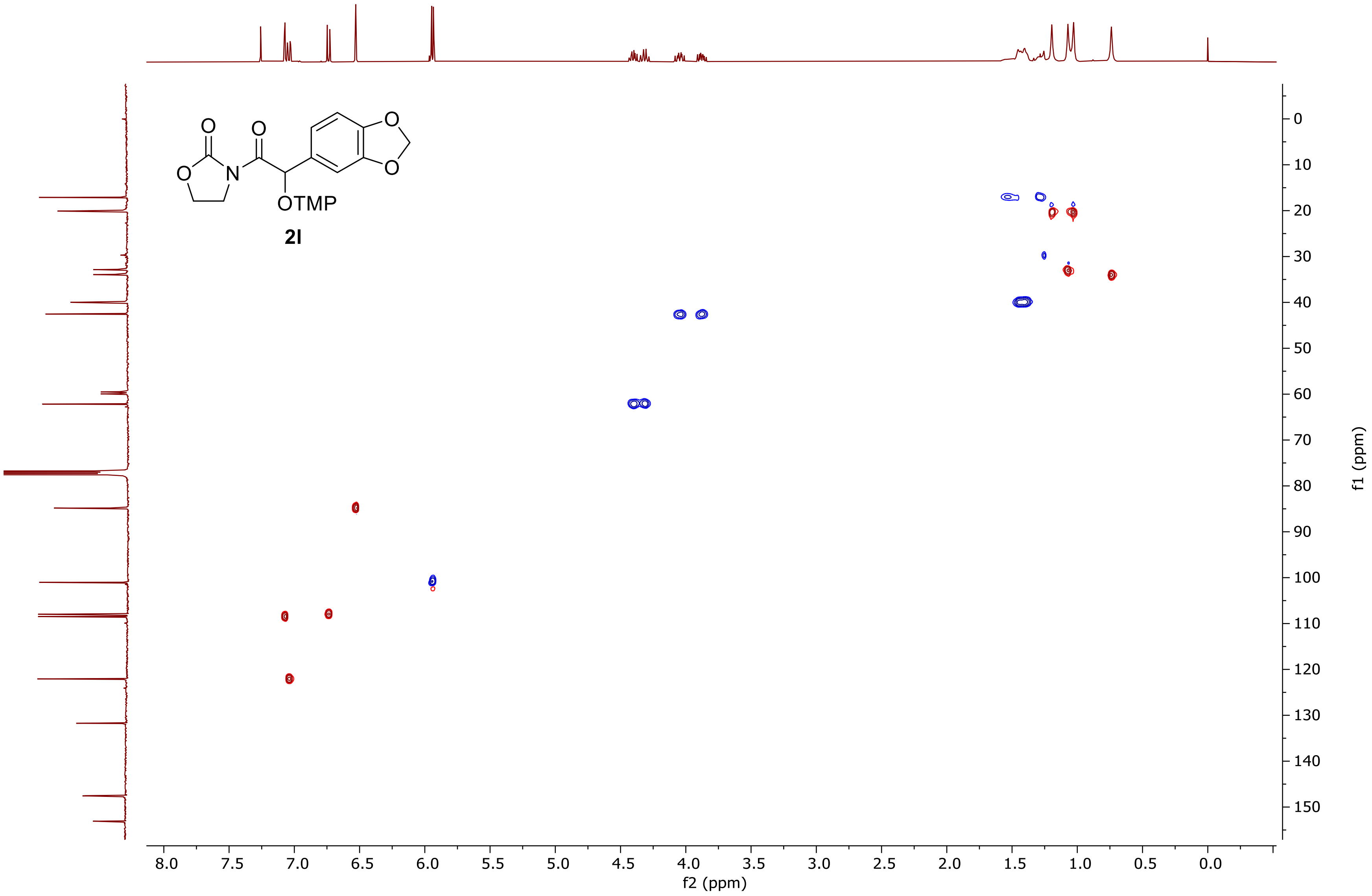

$^1$H NMR (400 MHz, $CDCl_3$)

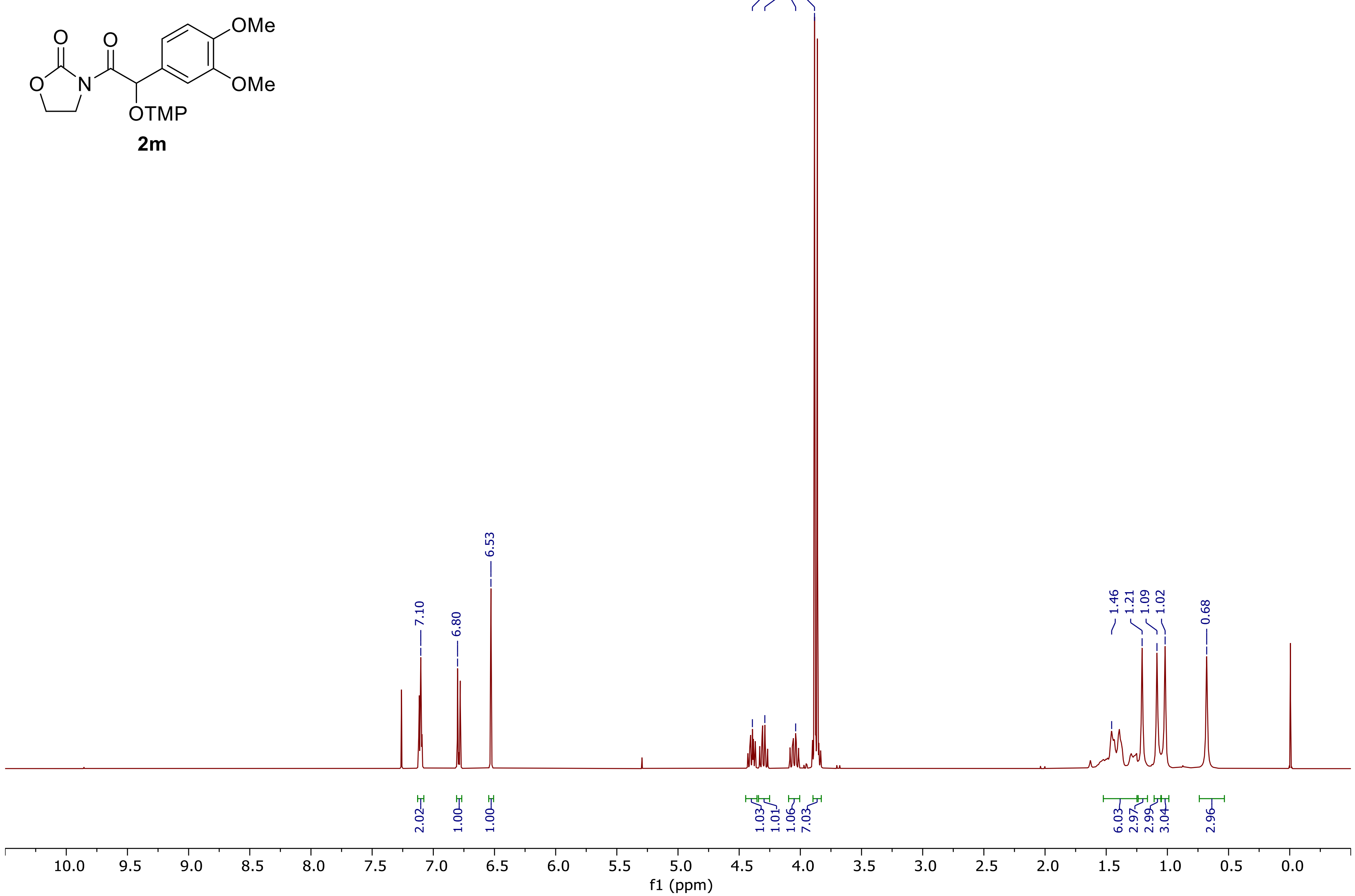

$^{13}C\{^{1}H\}$ NMR (101 MHz, $CDCl_3$)

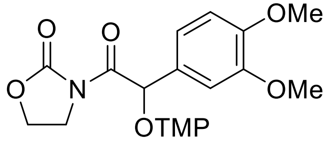


**2m**

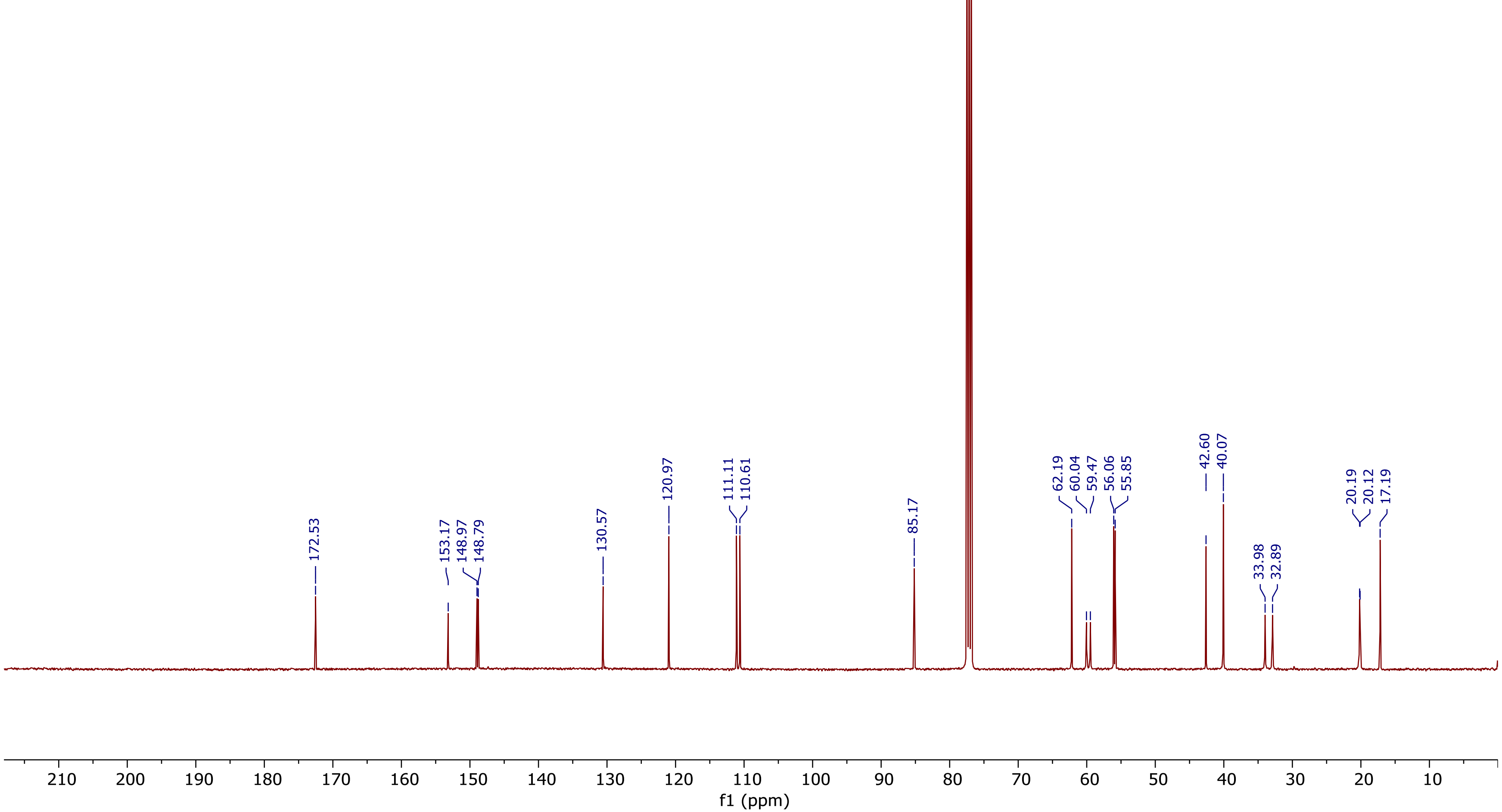

2D $^1$H -$^1$H COSY (400 MHz, $CDCl_3$)

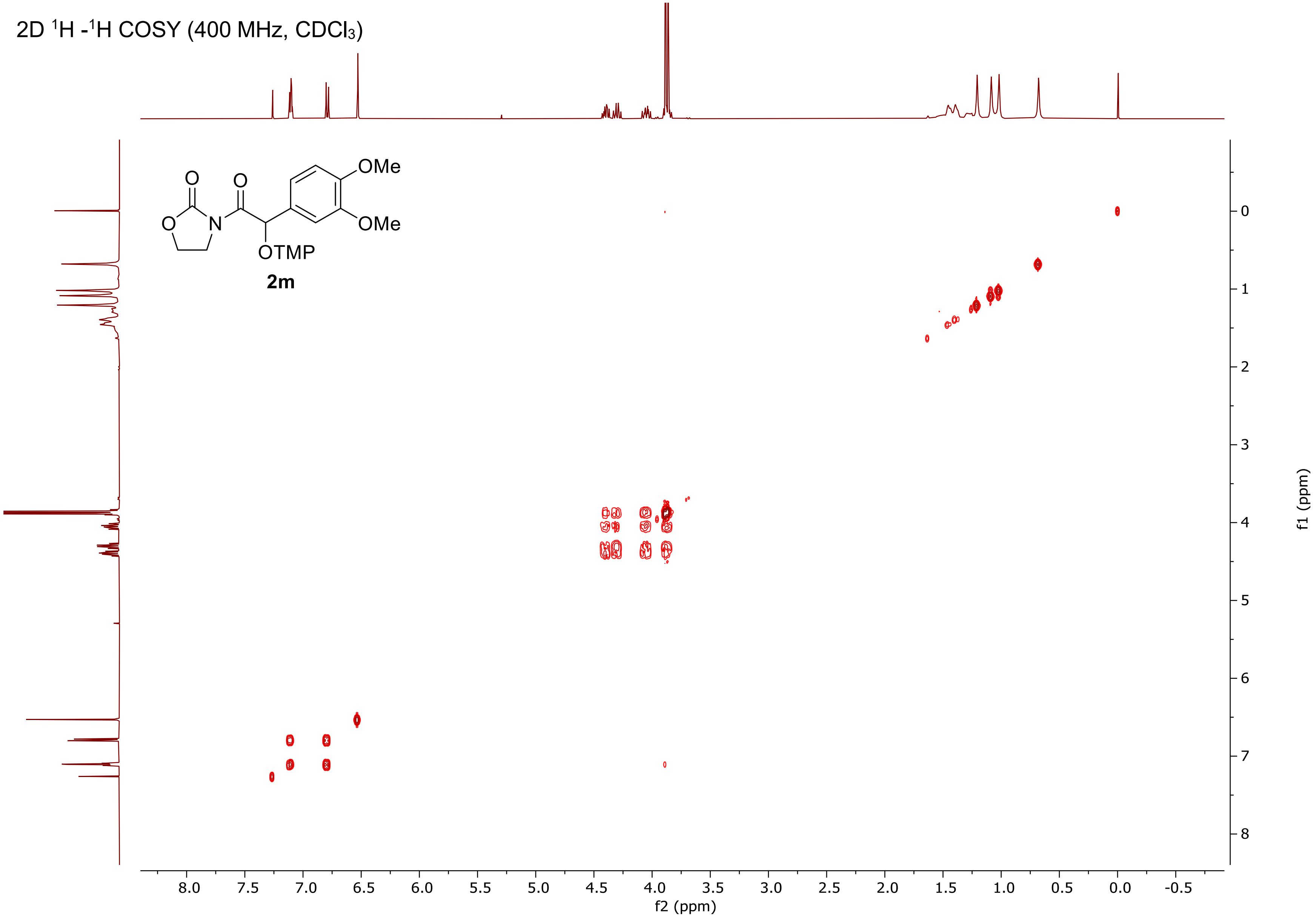

2D $^1H$ -$^{13}C$ HSQC (400 MHz, $CDCl_3$)

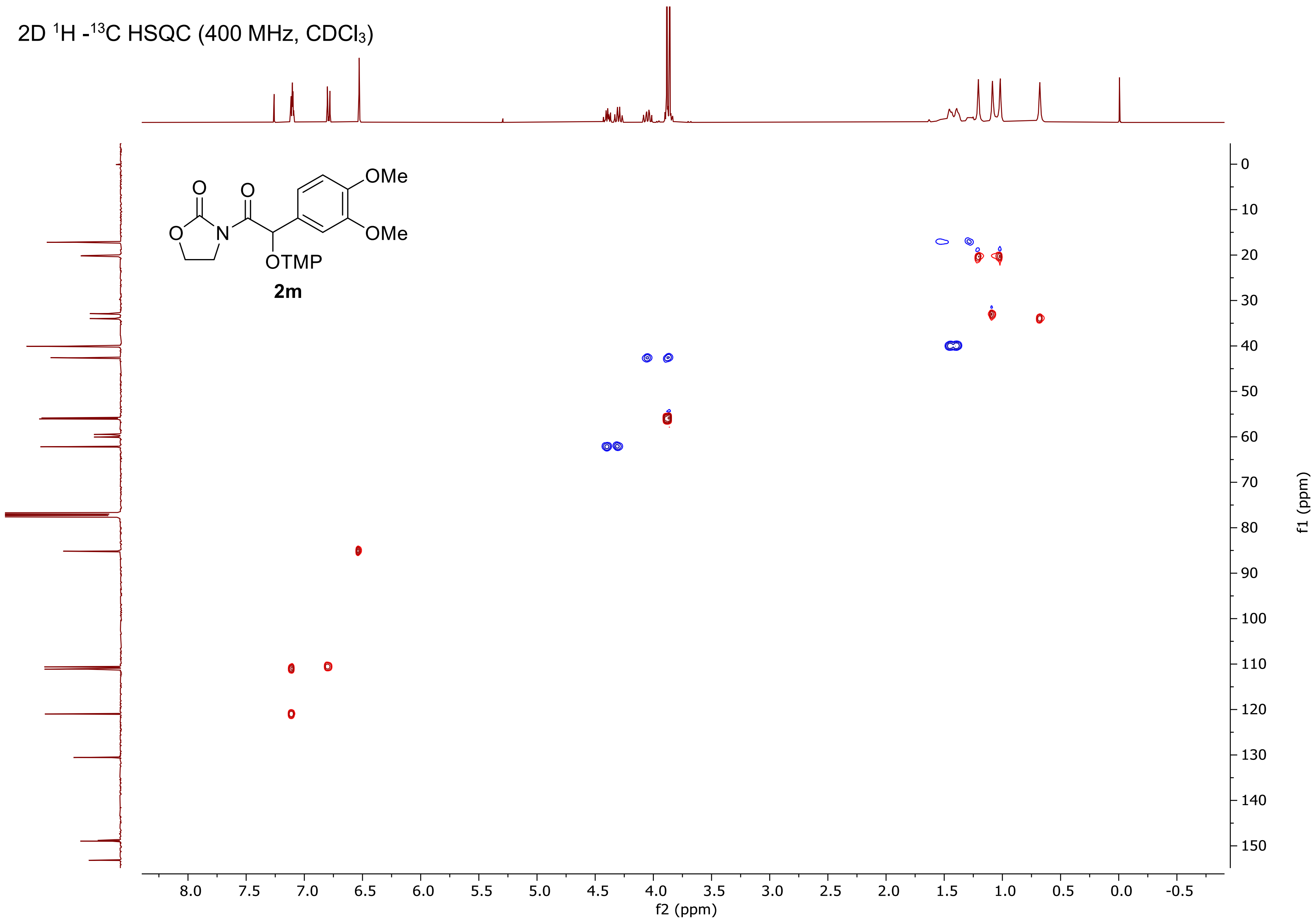

$^{1}H$ NMR (500 MHz, $CDCl_3$)

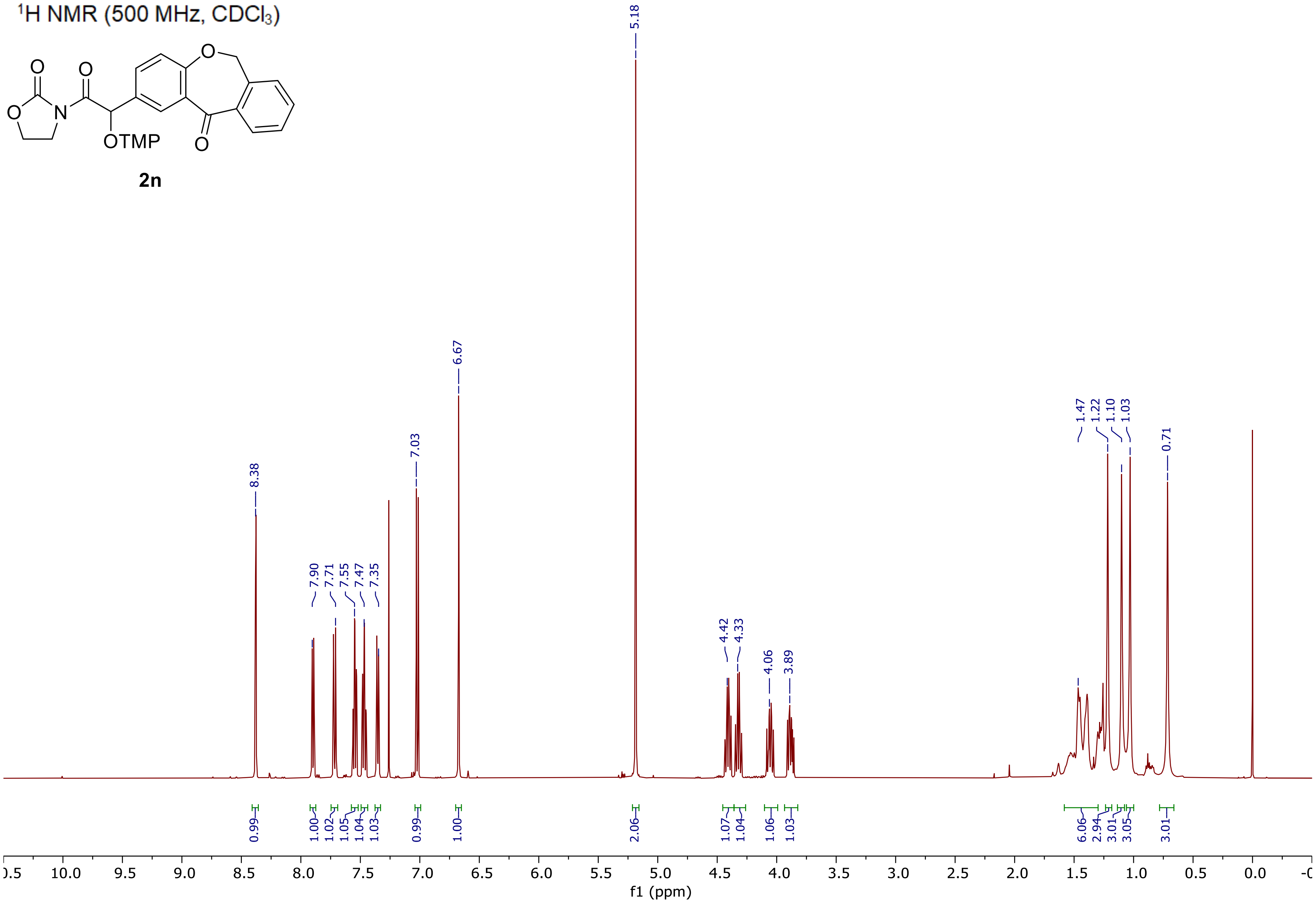


**2n**

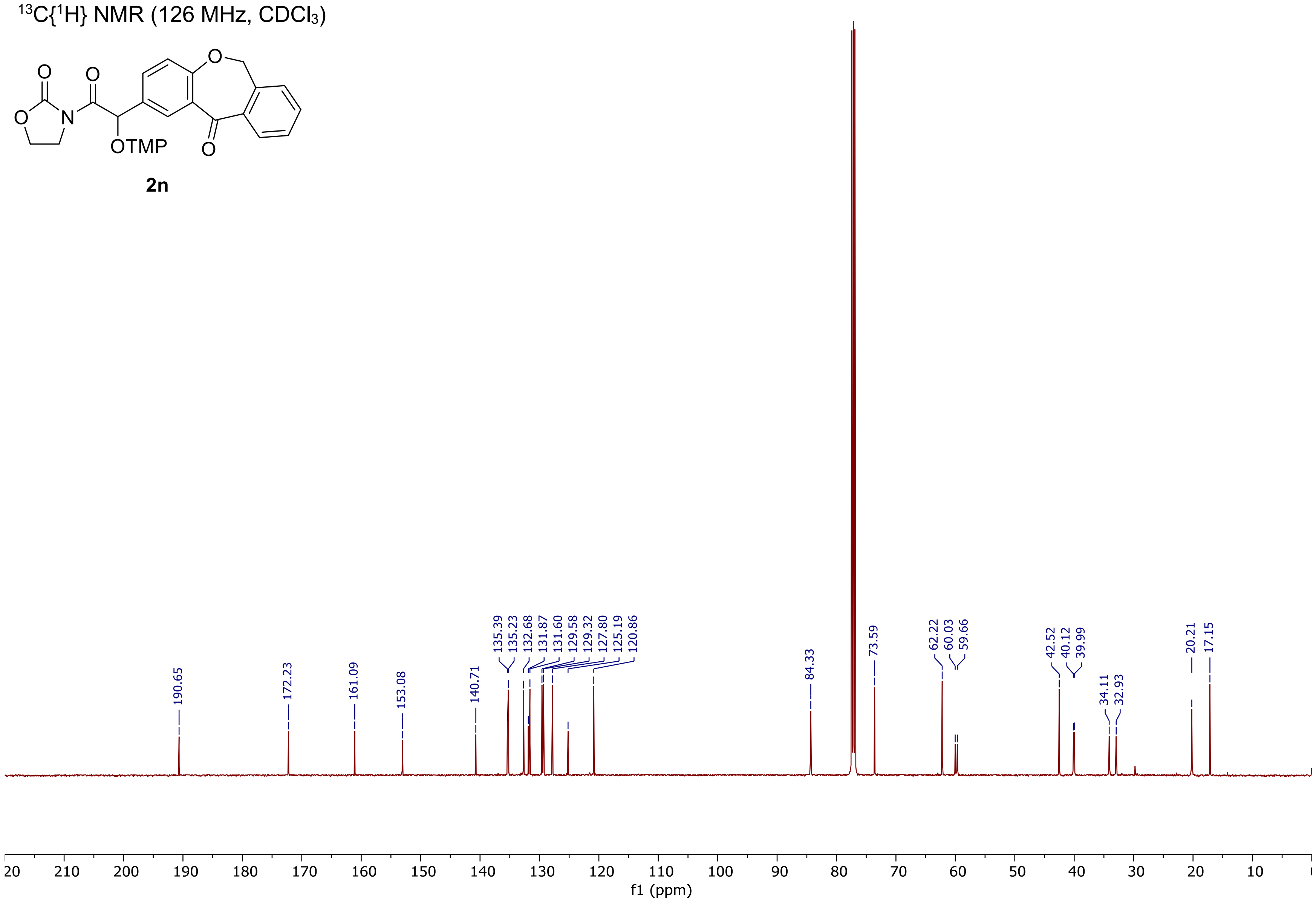

$^{13}C\{^1H\}$ NMR (126 MHz, CDCl$_3$)
OTMP
2n
190.65
172.23
161.09
153.08
140.71
135.39
135.23
132.68
131.87
131.60
129.58
129.32
127.80
125.19
120.86
84.33
73.59
62.22
60.03
59.66
42.52
40.12
39.99
34.11
32.93
20.21
17.15
f1 (ppm)

2D $^1H$ -$^1H$ COSY (500 MHz, $CDCl_3$)

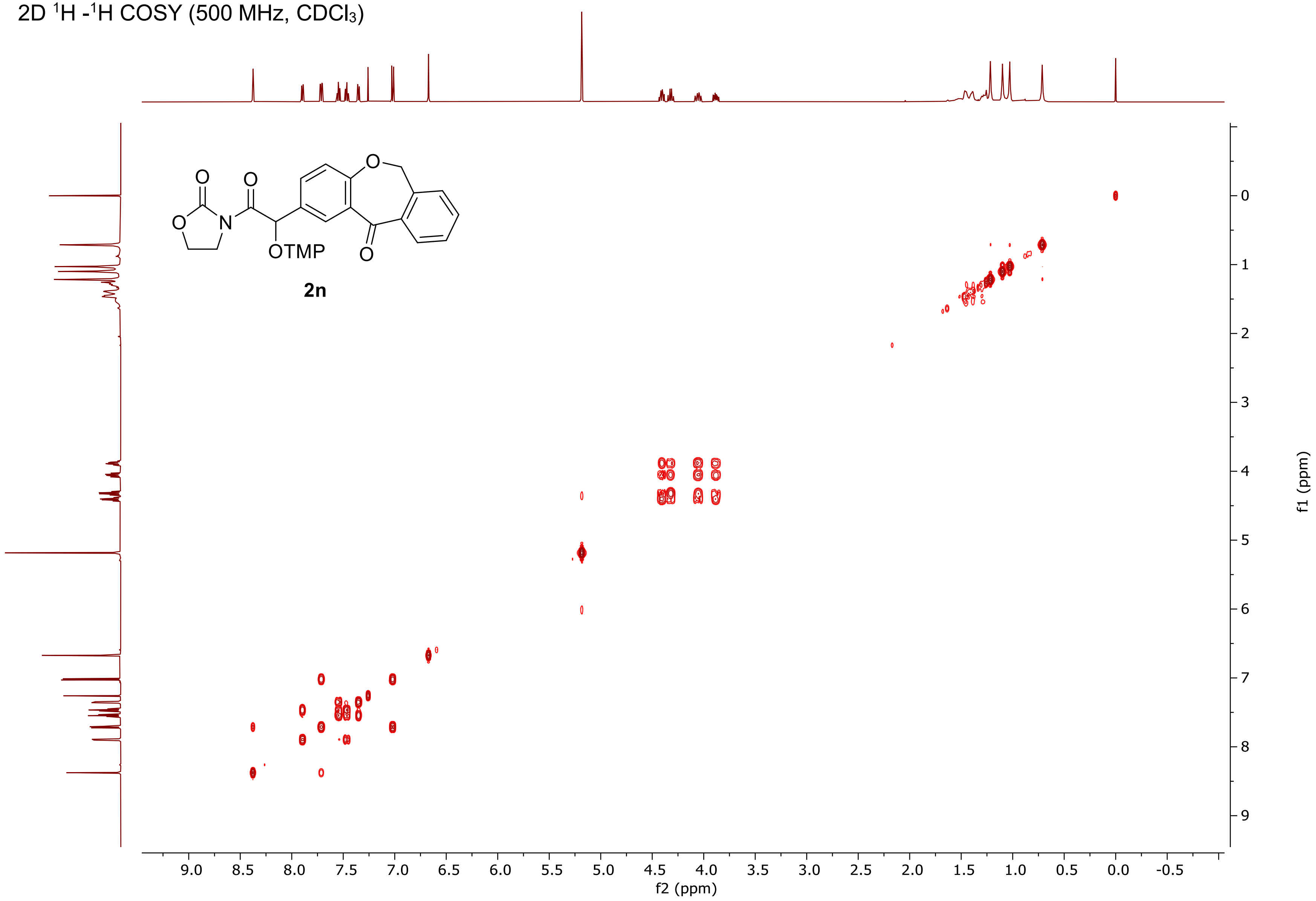

2D $^1$H -$^{13}$C HSQC (500 MHz, $CDCl_3$)

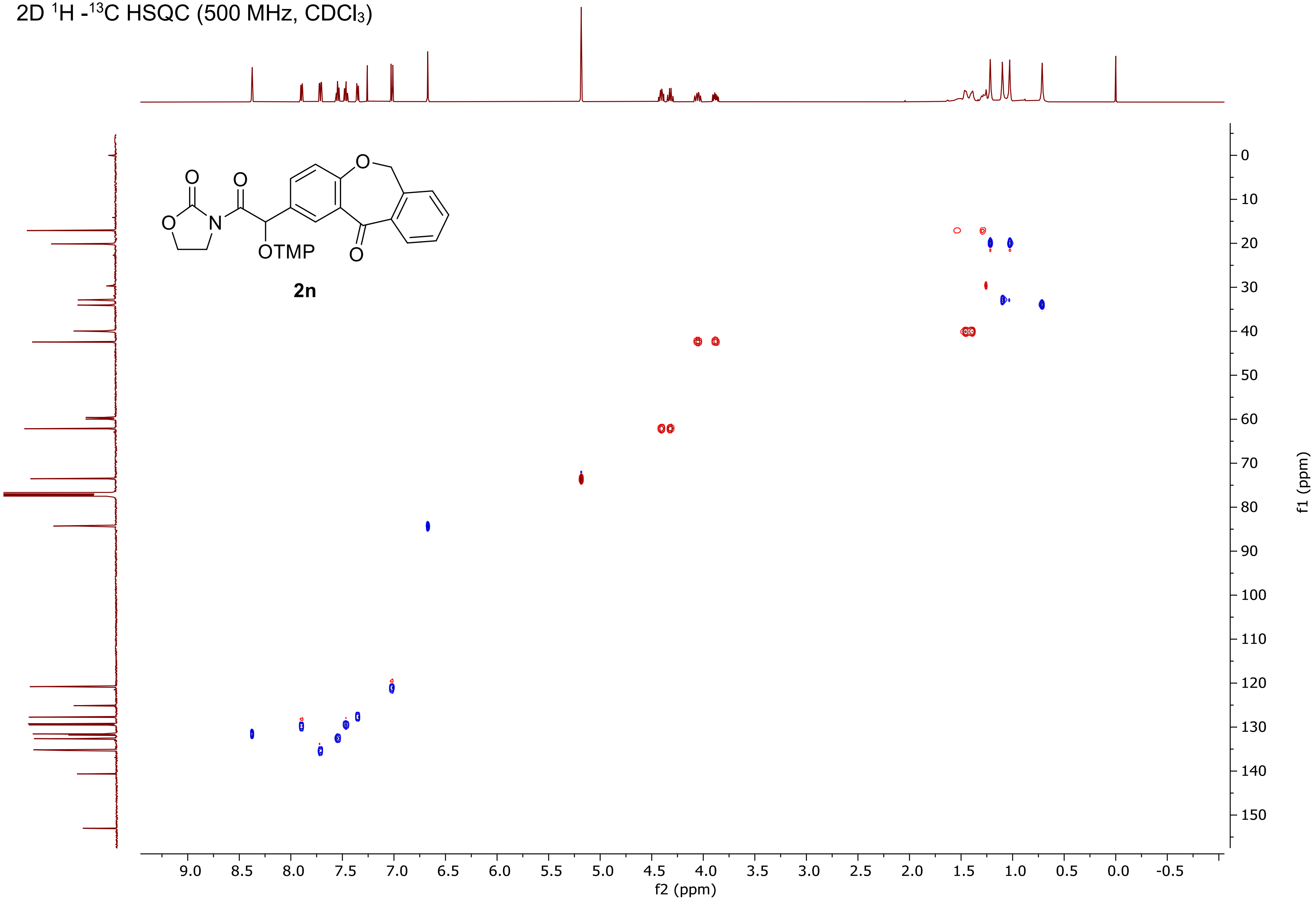

$^{1}$H NMR (500 MHz, $CDCl_3$)

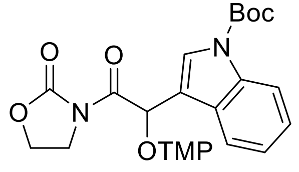


**2o**

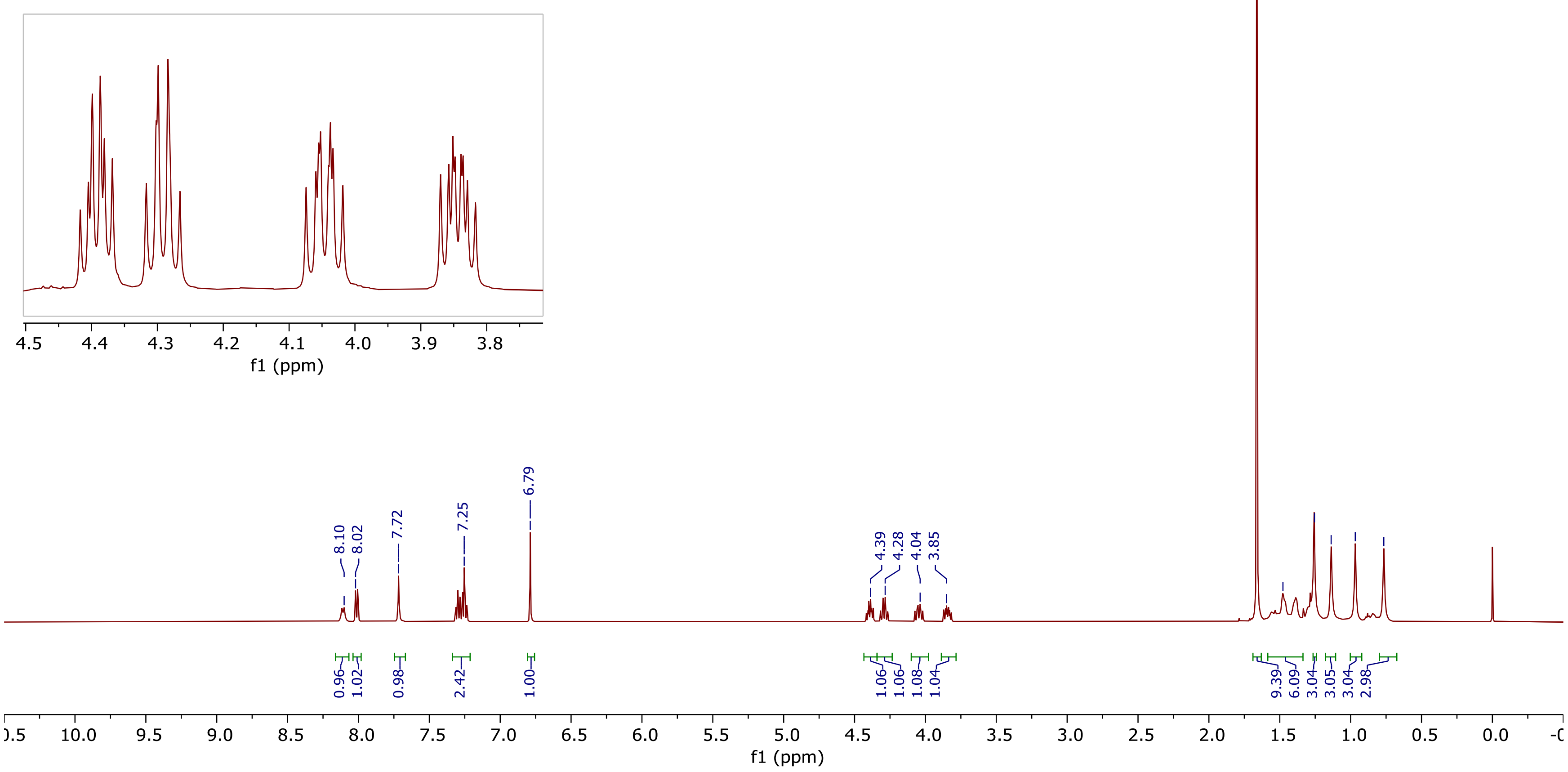

$^{13}C\{^{1}H\}$ NMR (126 MHz, $CDCl_3$)

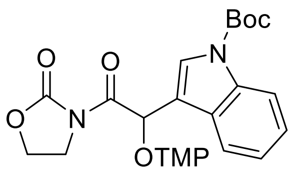


**2o**

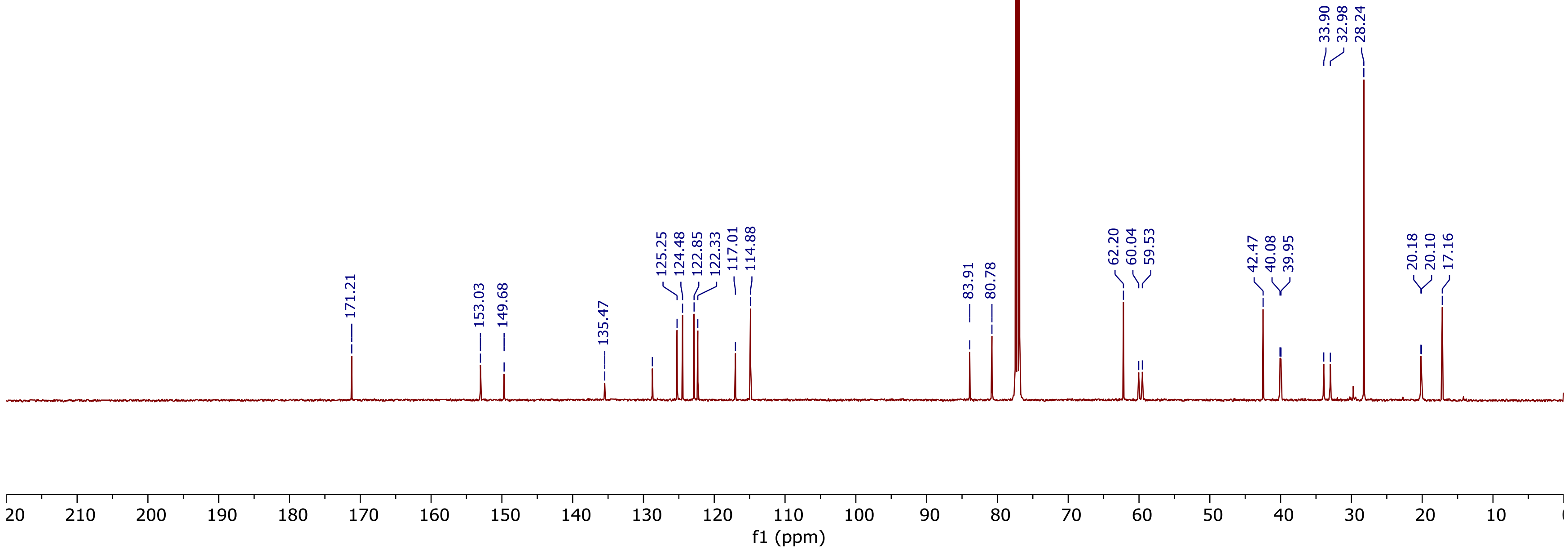

2D $^1$H -$^1$H COSY (500 MHz, $CDCl_3$)

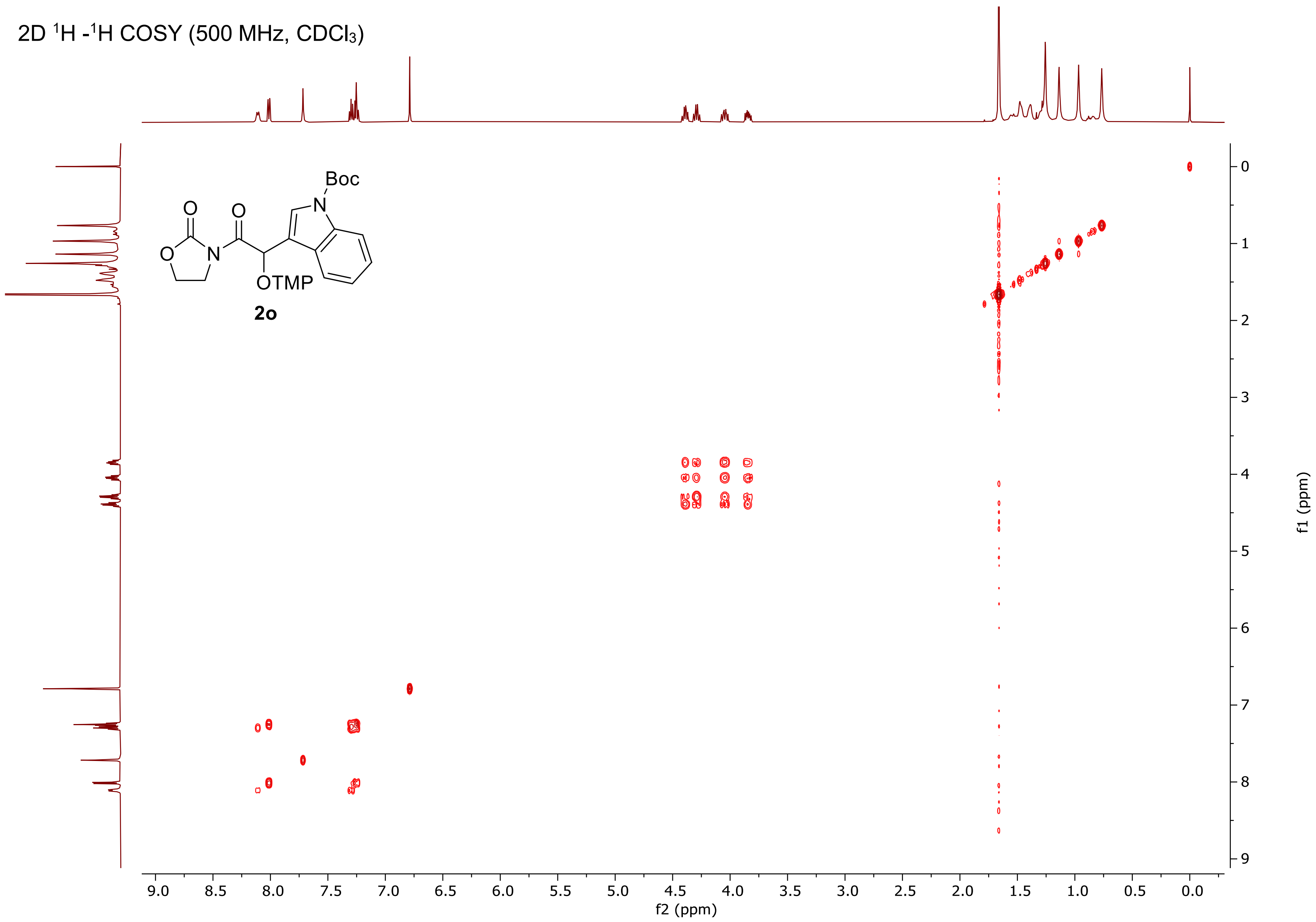

2D $^{1}H$ -$^{13}C$ HSQC (500 MHz, $CDCl_3$)

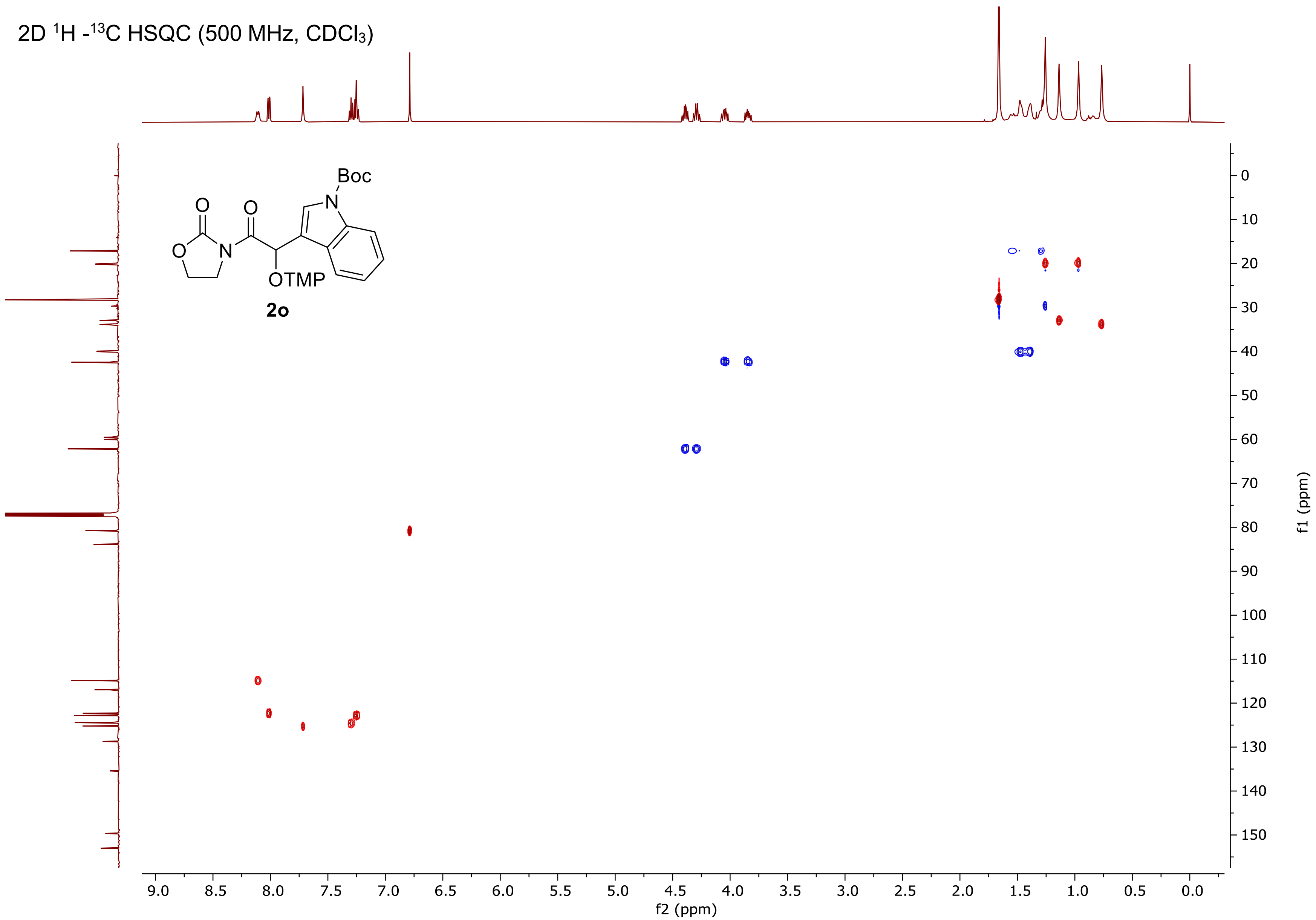

$^1$H NMR (500 MHz, $CDCl_3$)

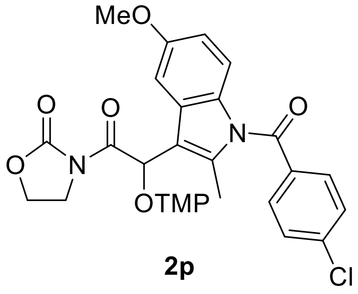


**2p**

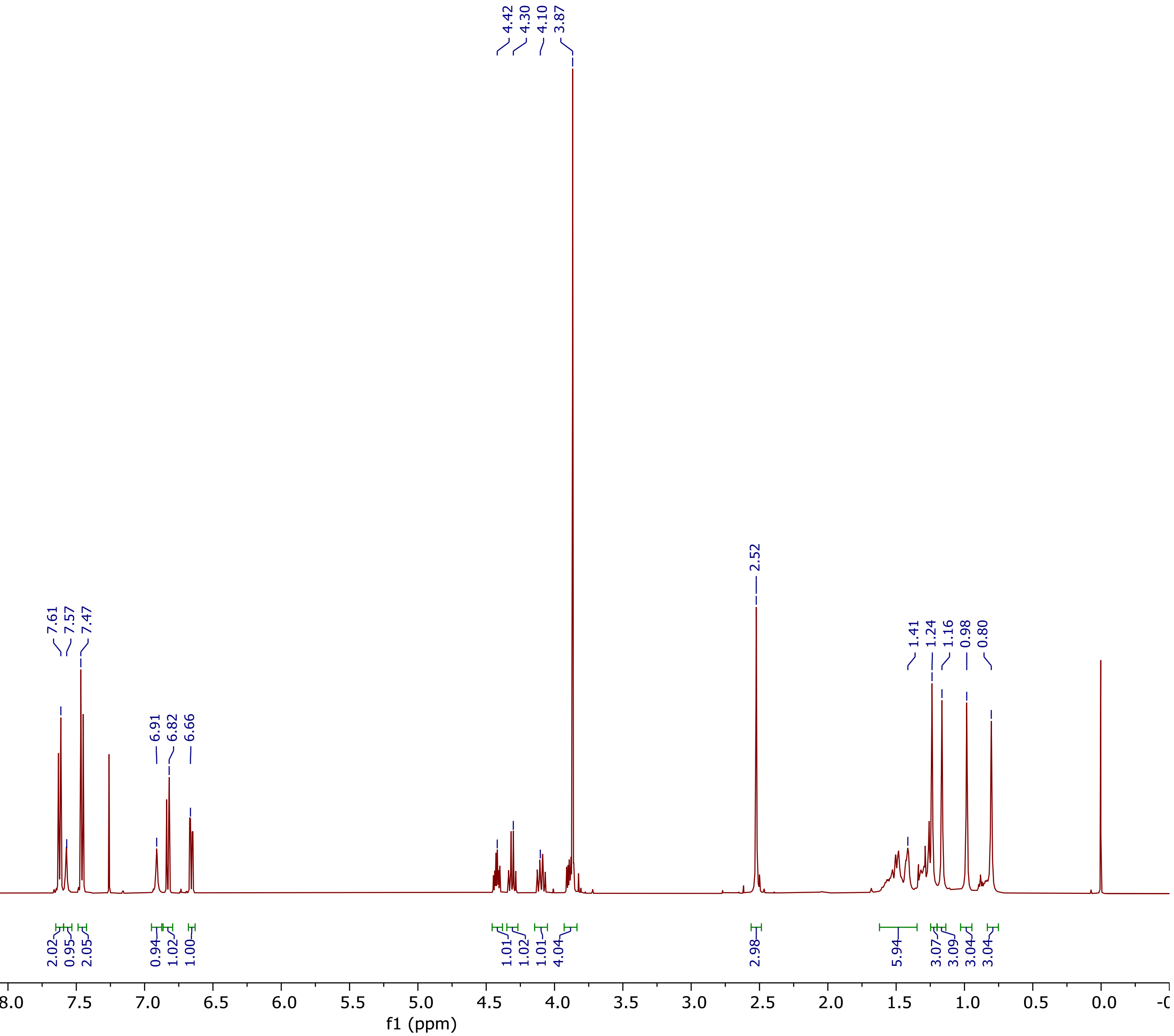

$^{13}C\{^{1}H\}$ NMR (126 MHz, $CDCl_3$)

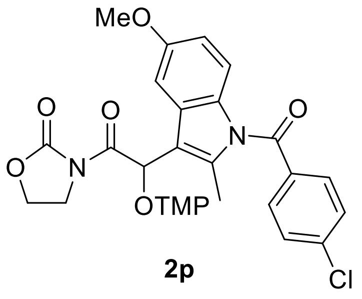

**2p**

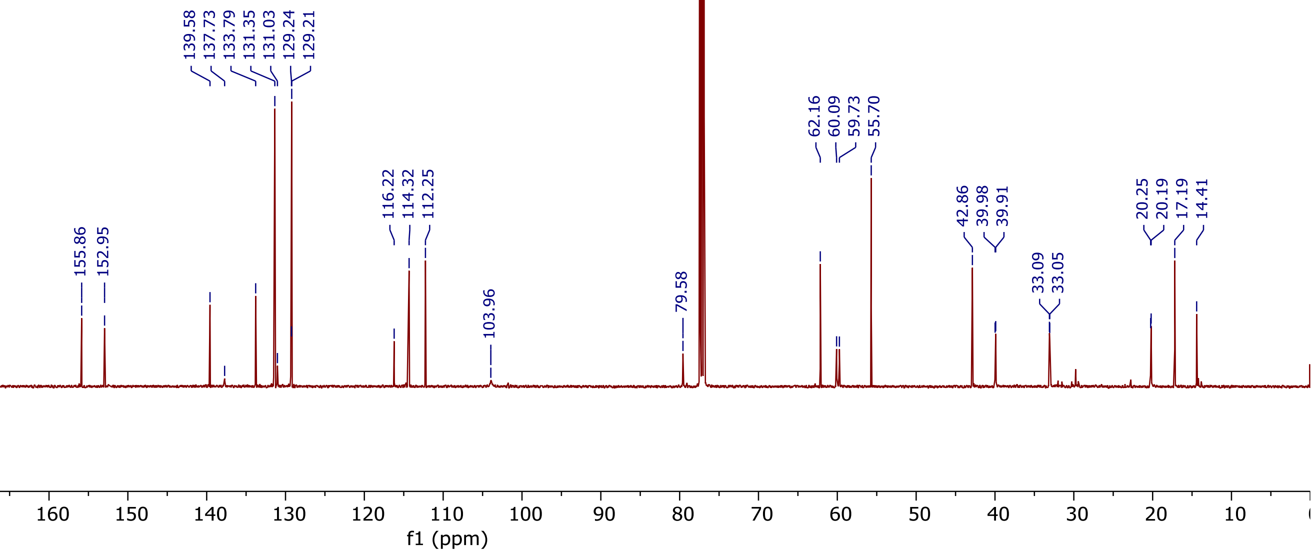

2D $^1$H -$^1$H COSY (500 MHz, $CDCl_3$)

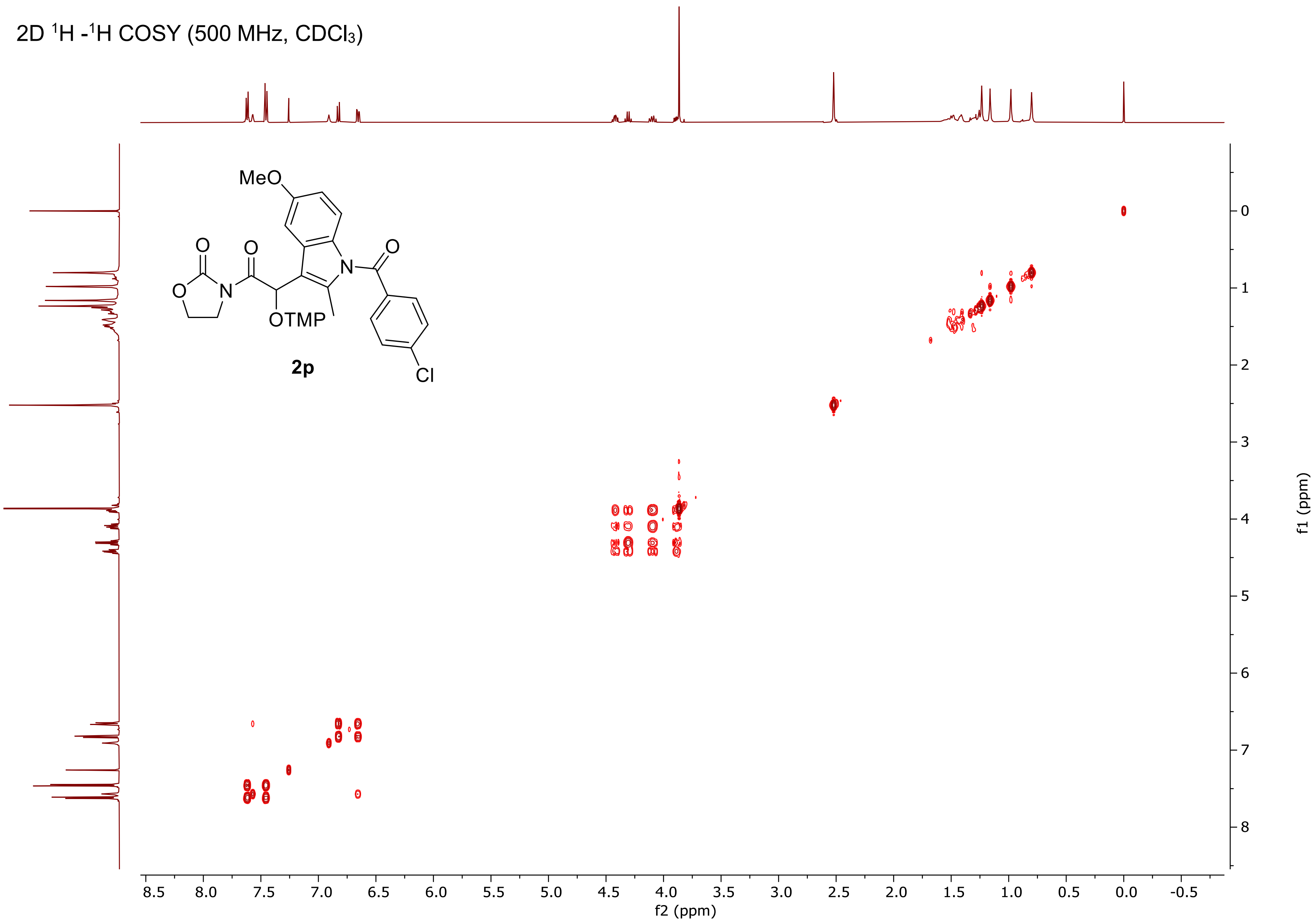

2D $^{1}H$ -$^{13}C$ HSQC (500 MHz, $CDCl_3$)

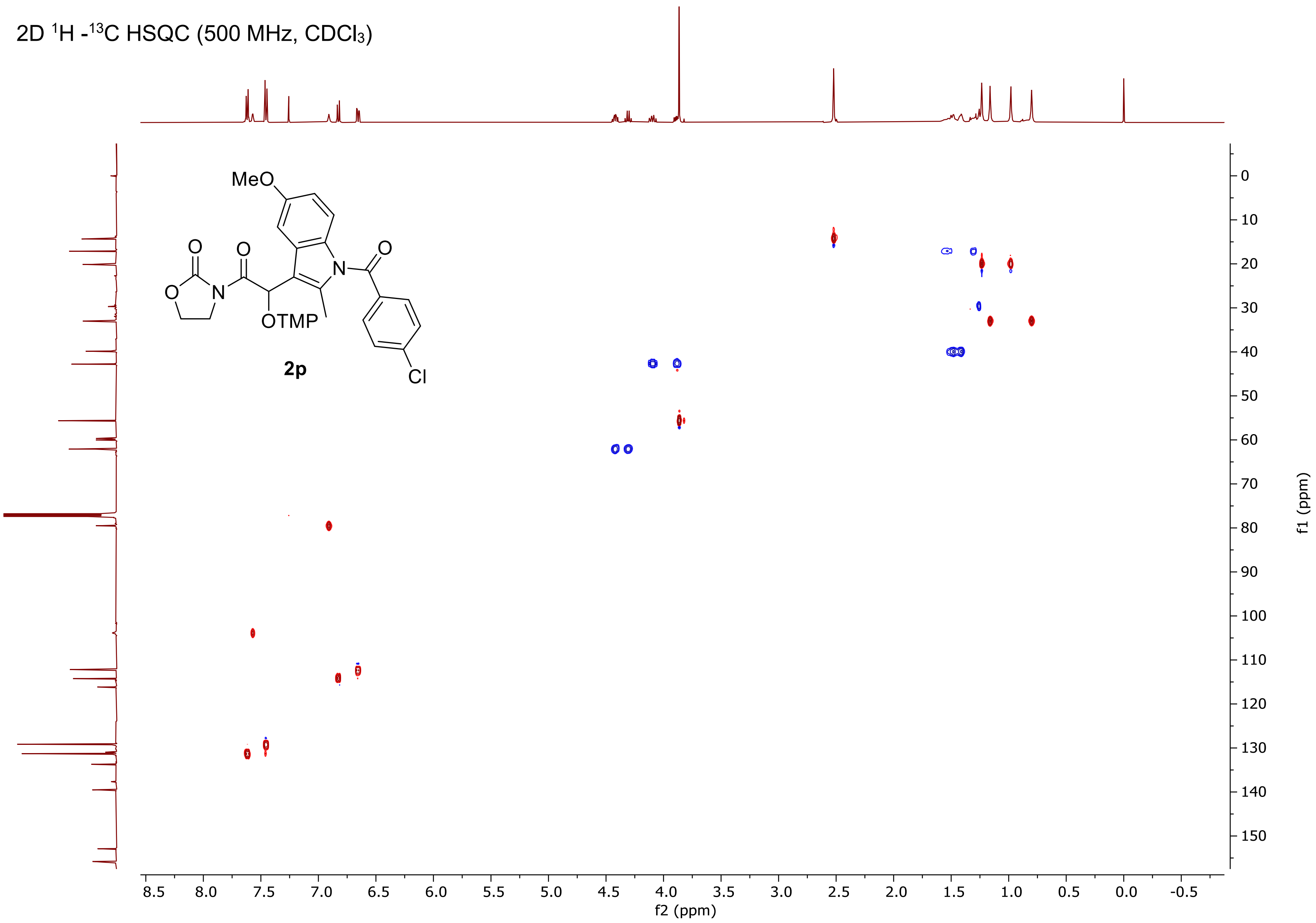

$^{1}H$ NMR (400 MHz, $CDCl_3$)

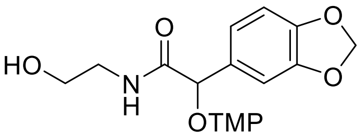


**3l**

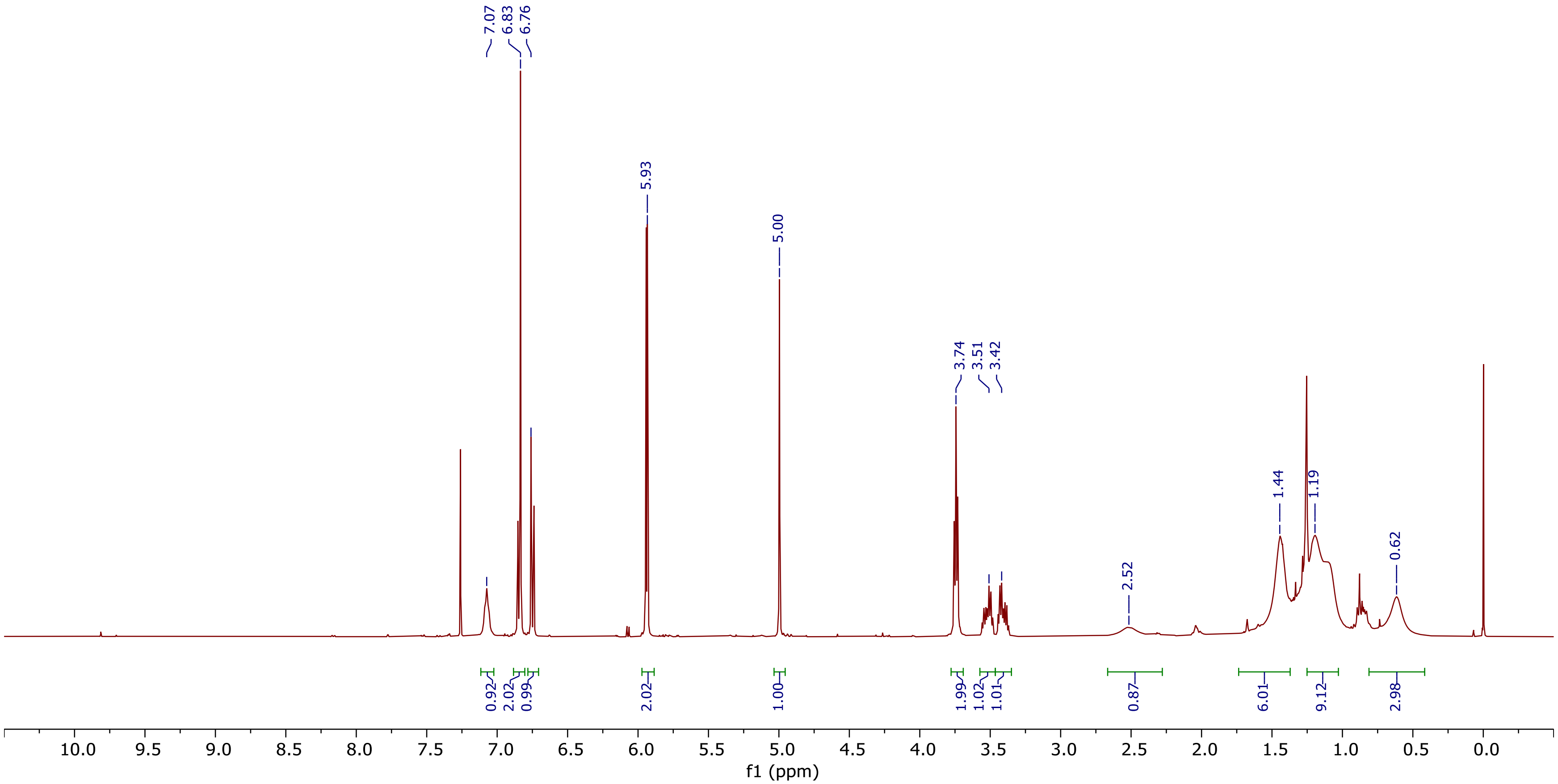

$^{13}C\{^{1}H\}$ NMR (101 MHz, $CDCl_3$)

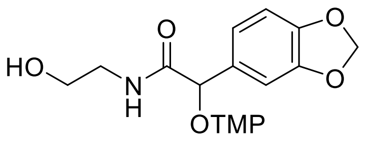


**3l**

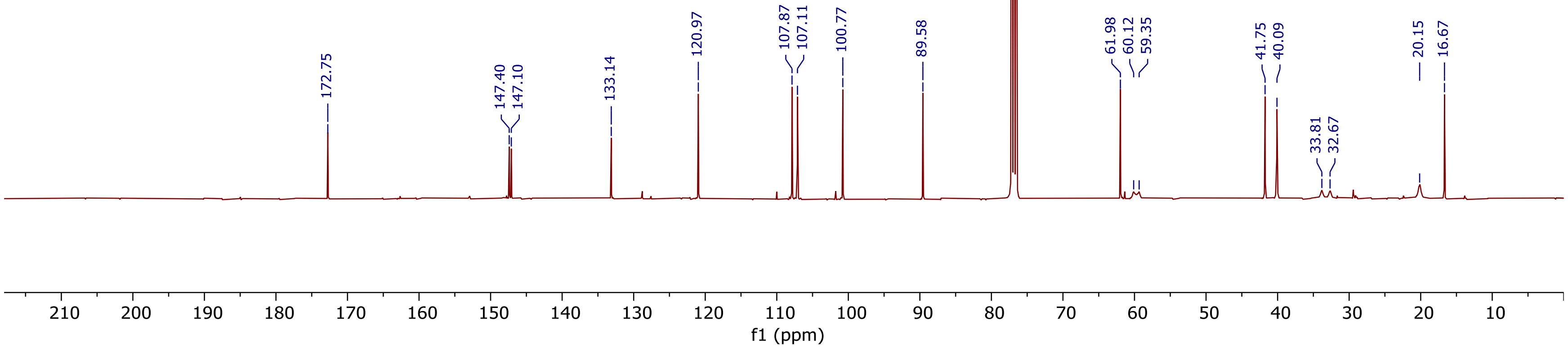

2D $^1$H -$^1$H COSY (400 MHz, $CDCl_3$)

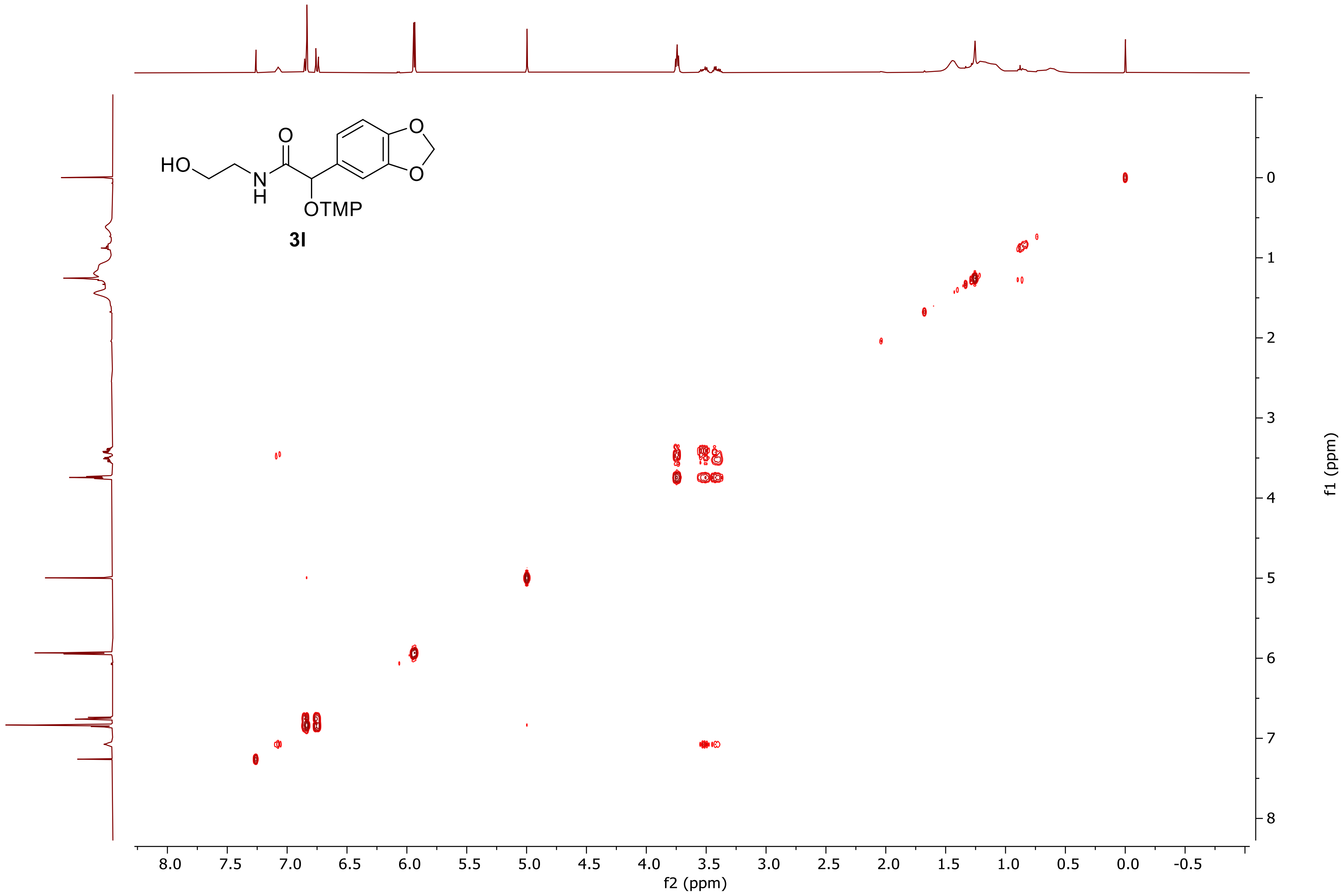

2D $^1H$ -$^{13}C$ HSQC (400 MHz, $CDCl_3$)

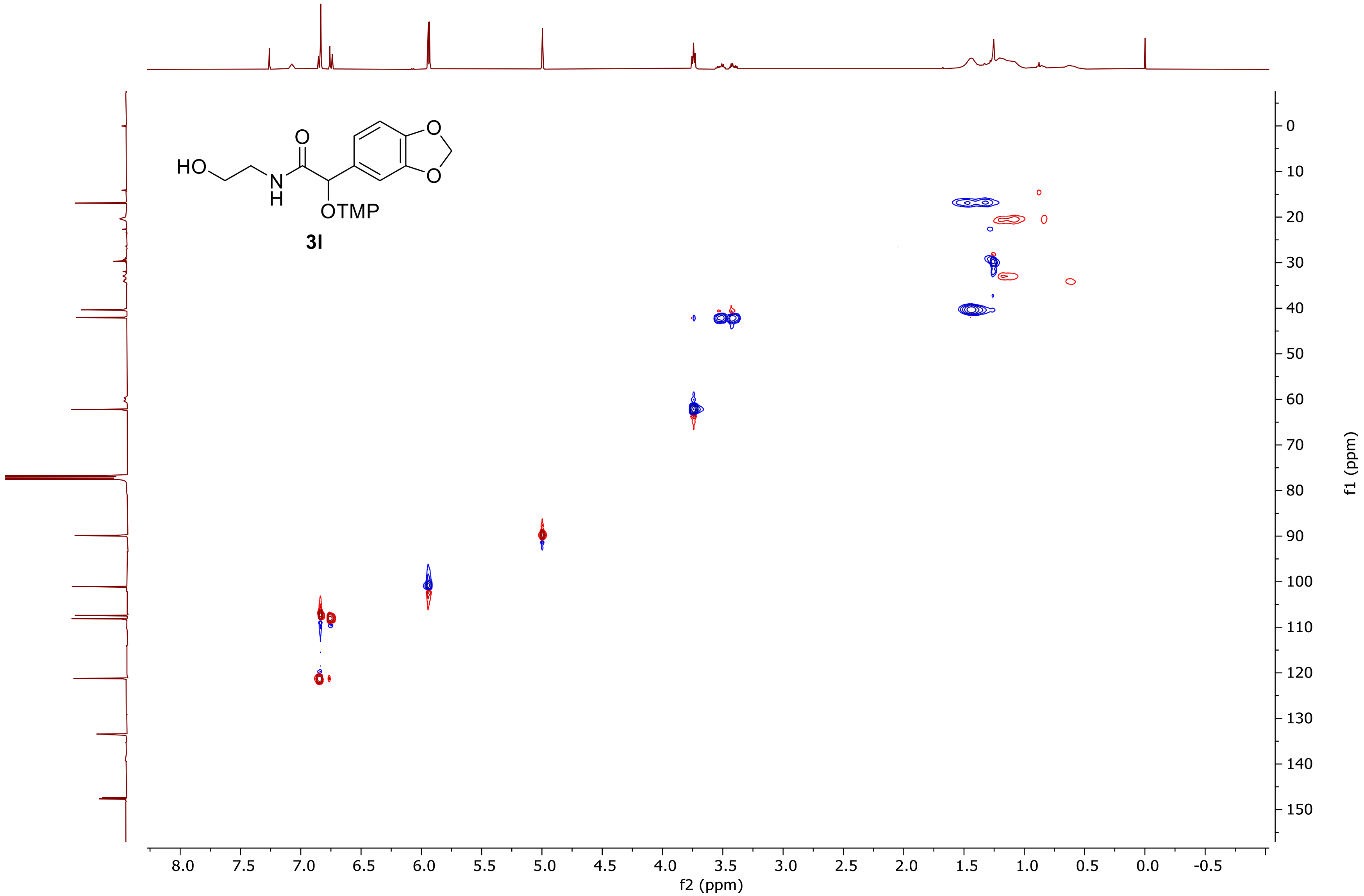

¹H NMR (500 MHz, $CDCl_3$)

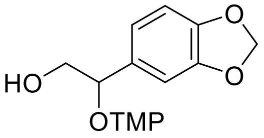

**4l**

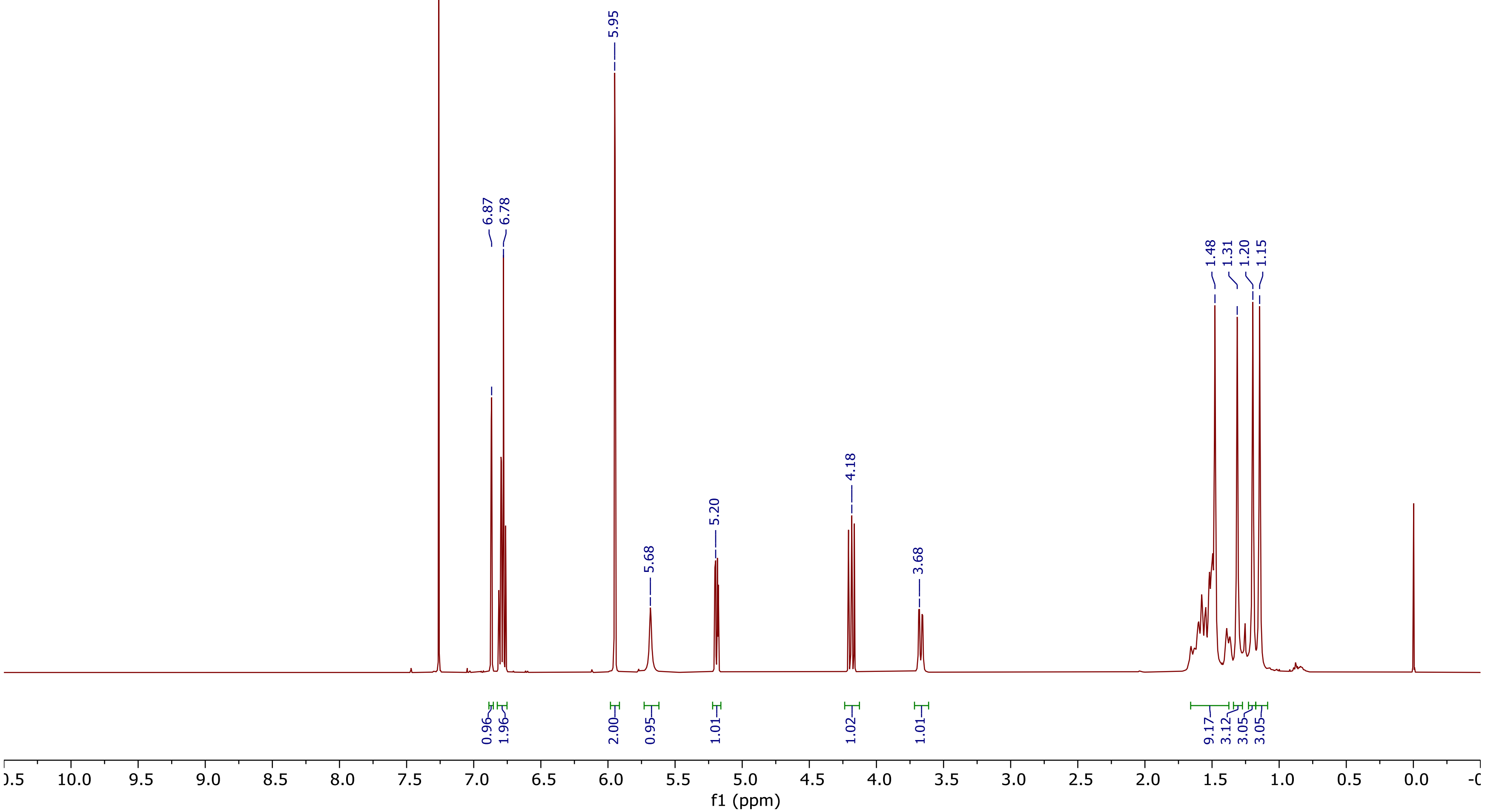

$^{13}C\{^{1}H\}$ NMR (126 MHz, $CDCl_3$)

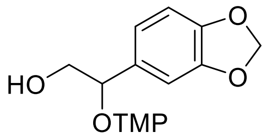

**4l**

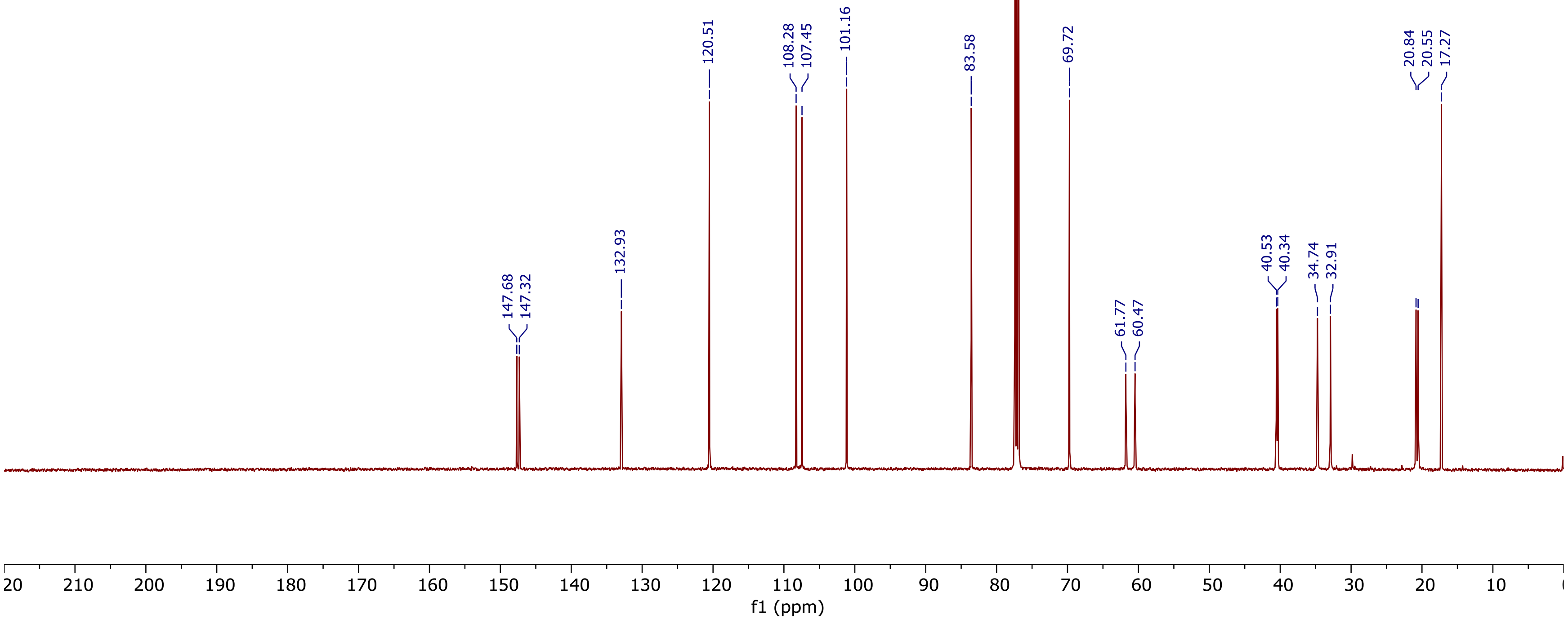

2D $^{1}H$ -$^{1}H$ COSY (500 MHz, $CDCl_3$)

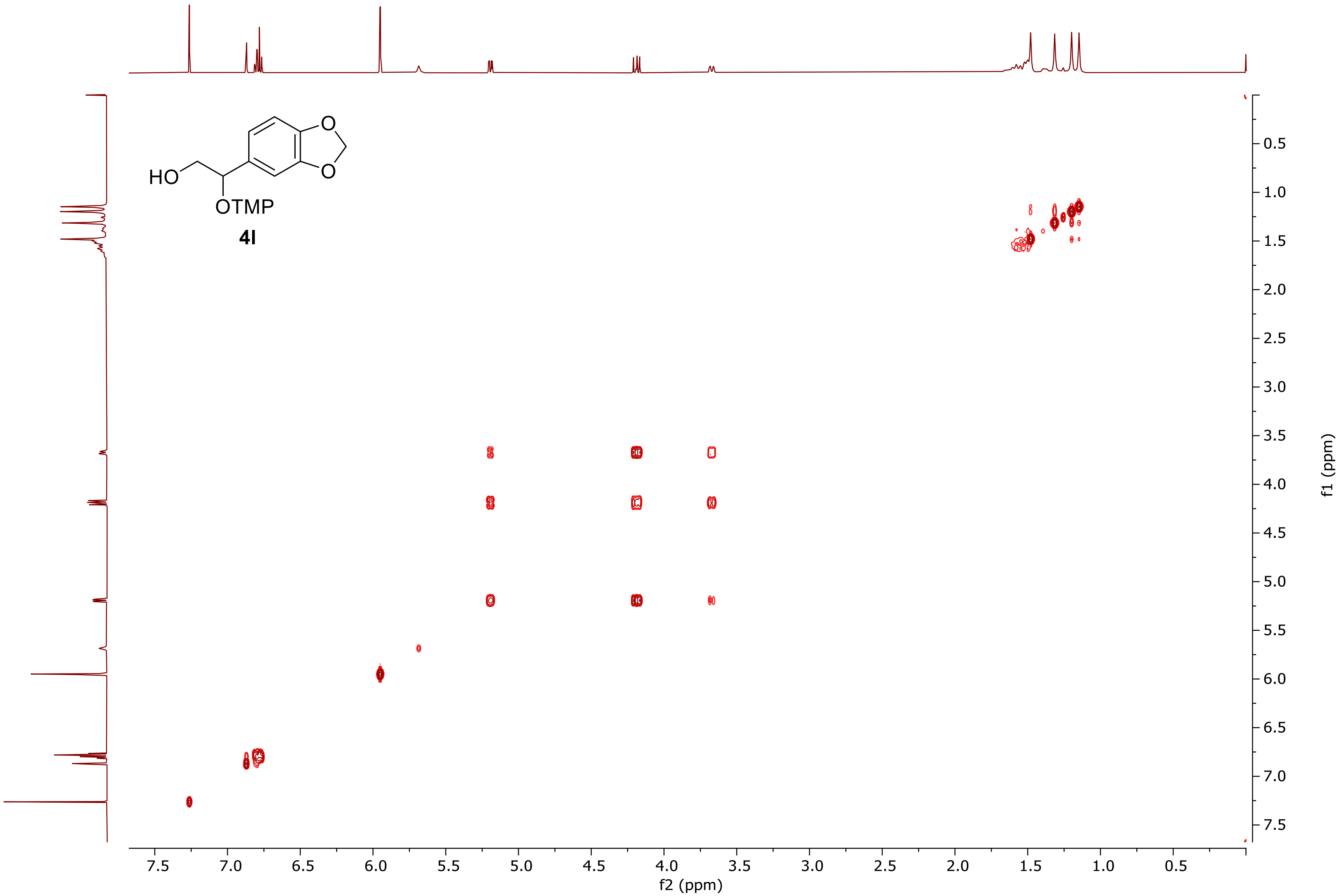

2D $^1$H -$^{13}$C HSQC (500 MHz, $CDCl_3$)

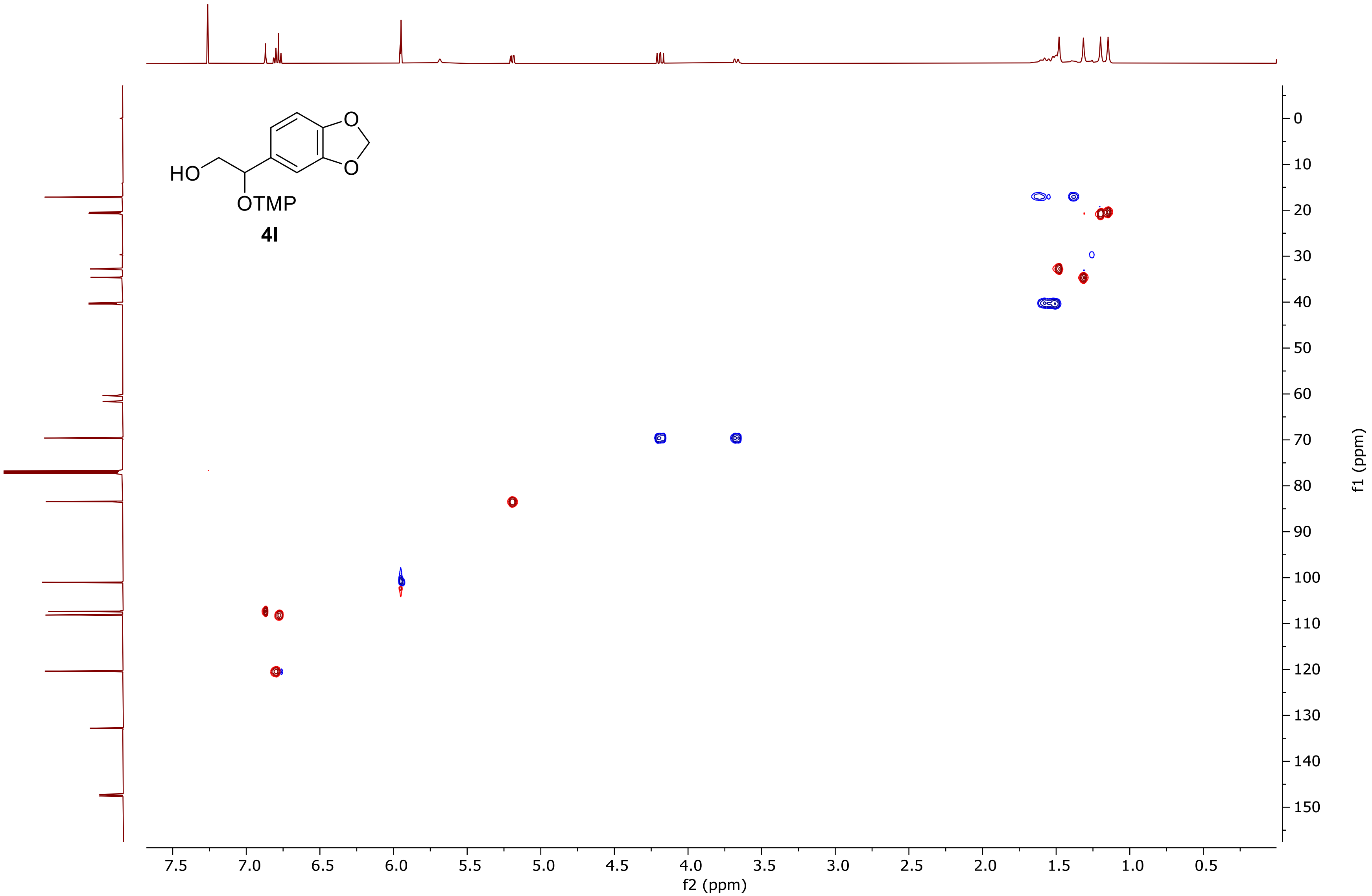

$^1$H NMR (400 MHz, $CDCl_3$)

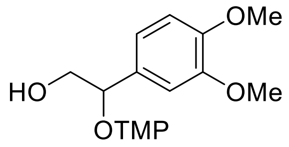


**4m**

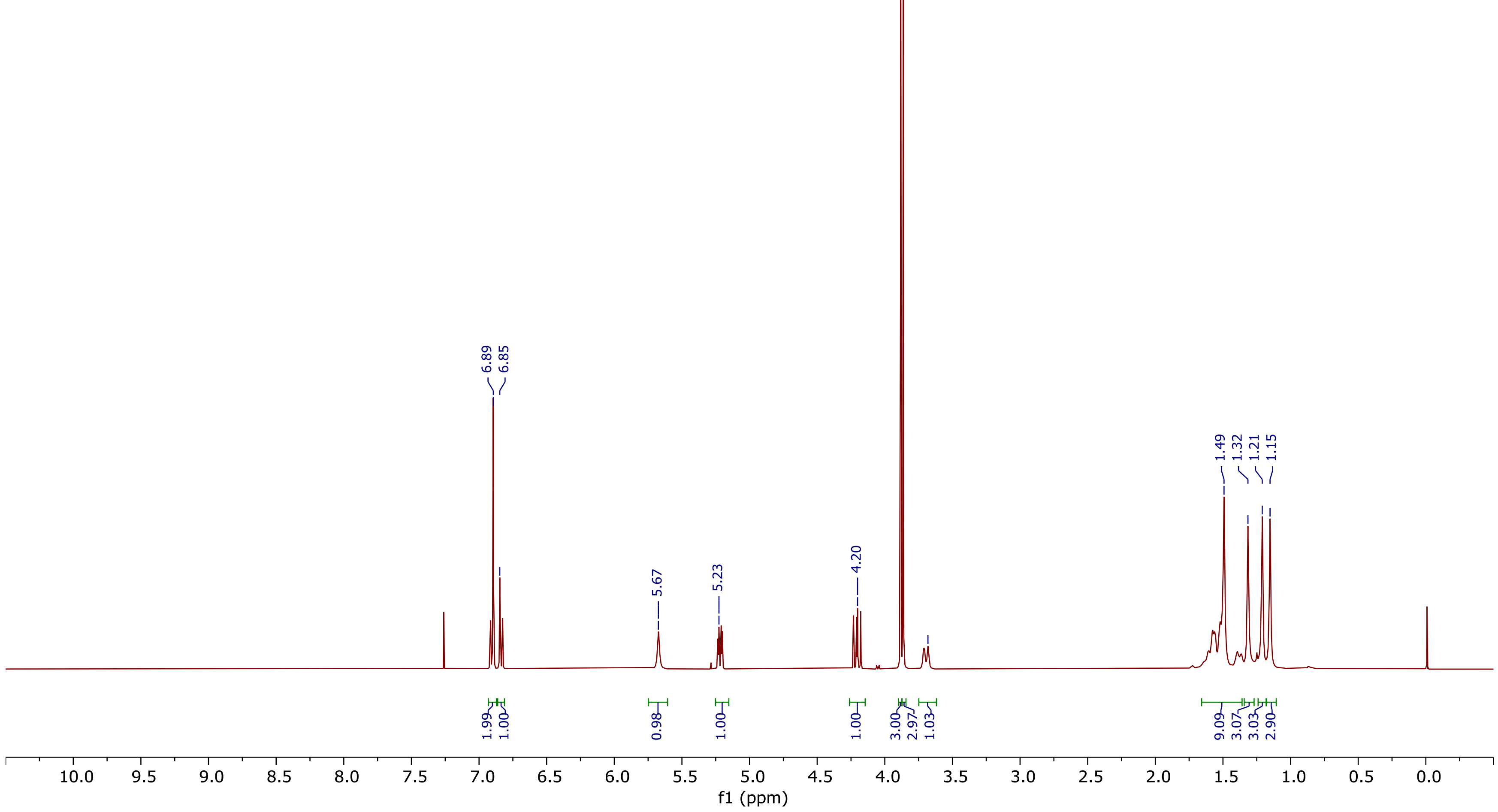

$^{13}C\{^{1}H\}$ NMR (101 MHz, $CDCl_3$)

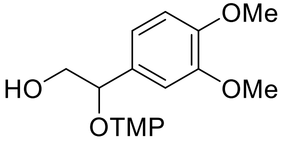


**4m**

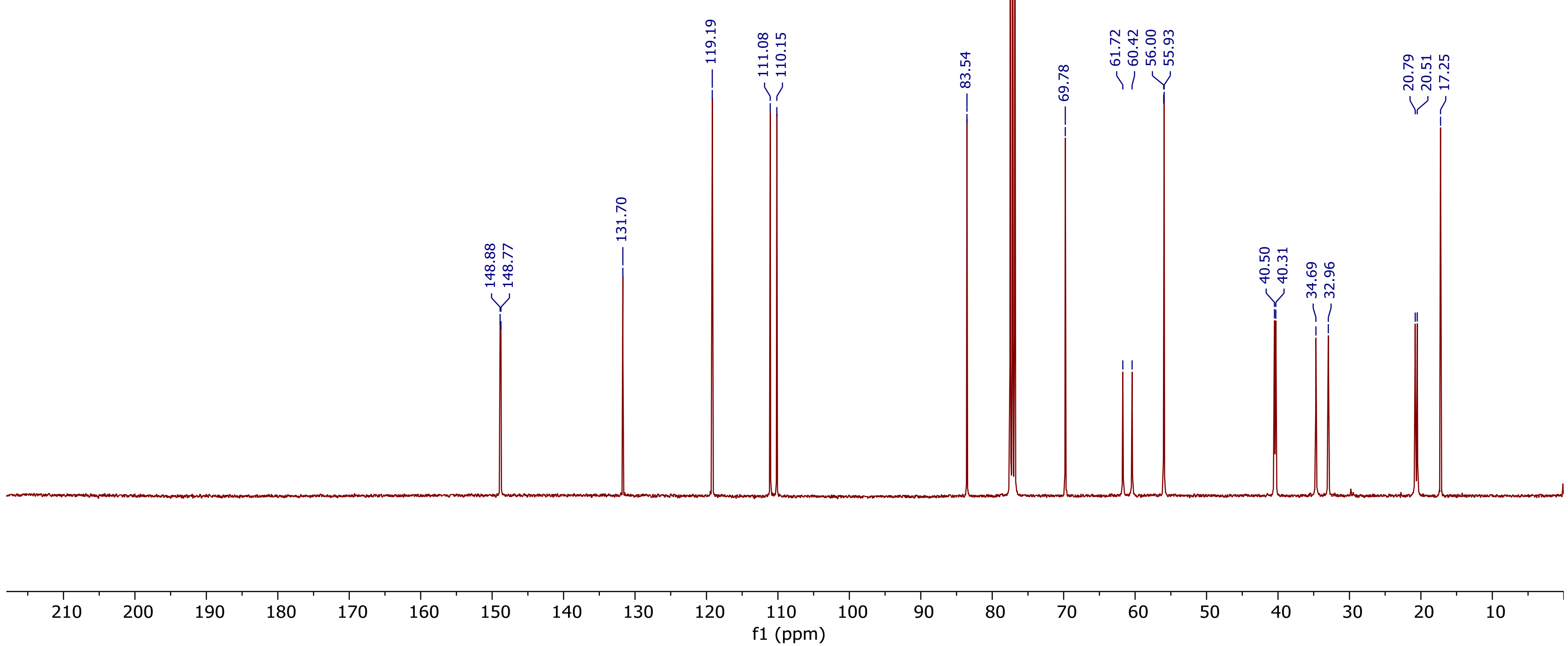

2D $^{1}H$ -$^{1}H$ COSY (400 MHz, $CDCl_3$)

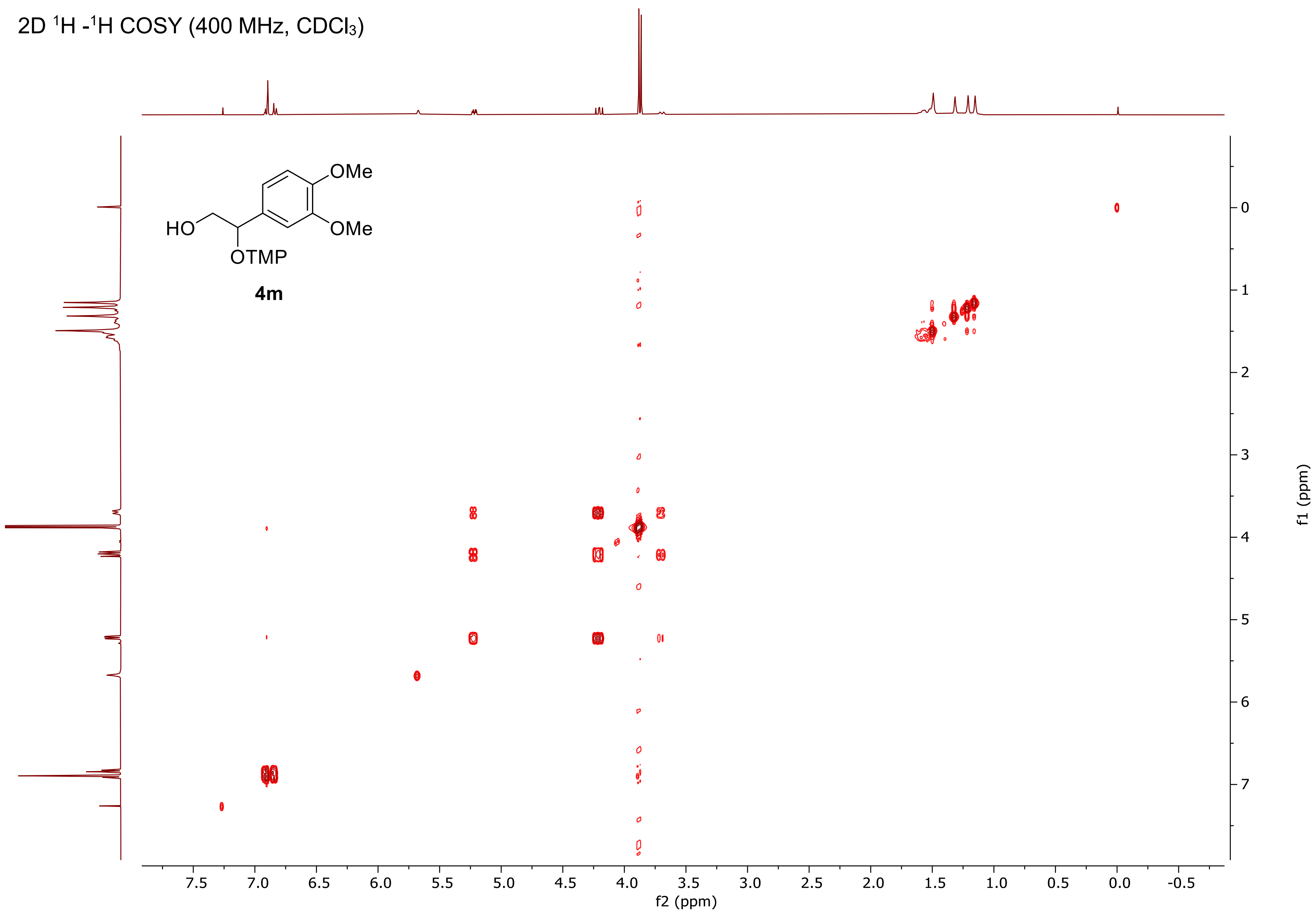

2D $^{1}H$ -$^{13}C$ HSQC (400 MHz, $CDCl_3$)

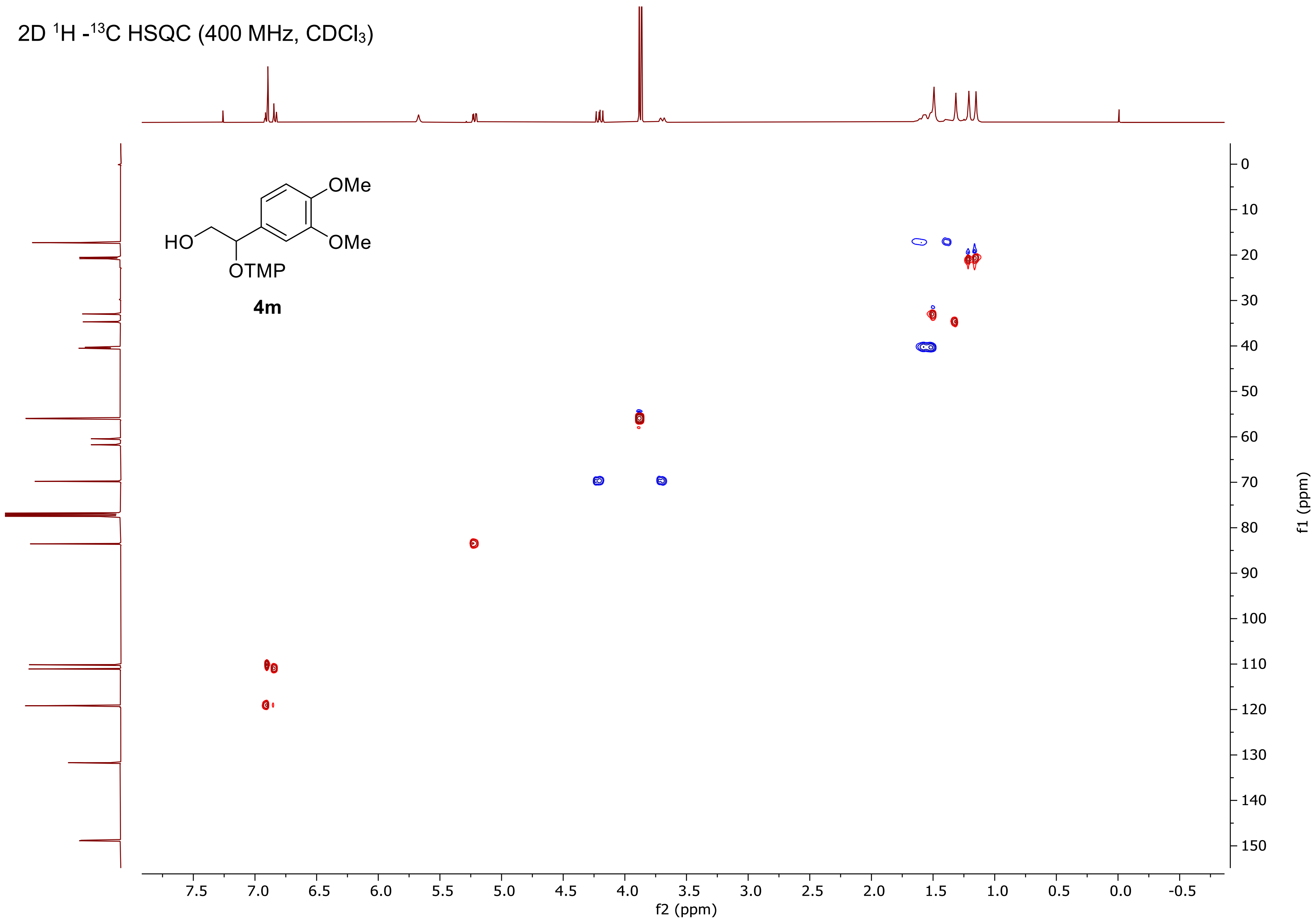

$^{1}H$ NMR (400 MHz, $CDCl_3$)

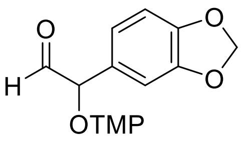


**5l**

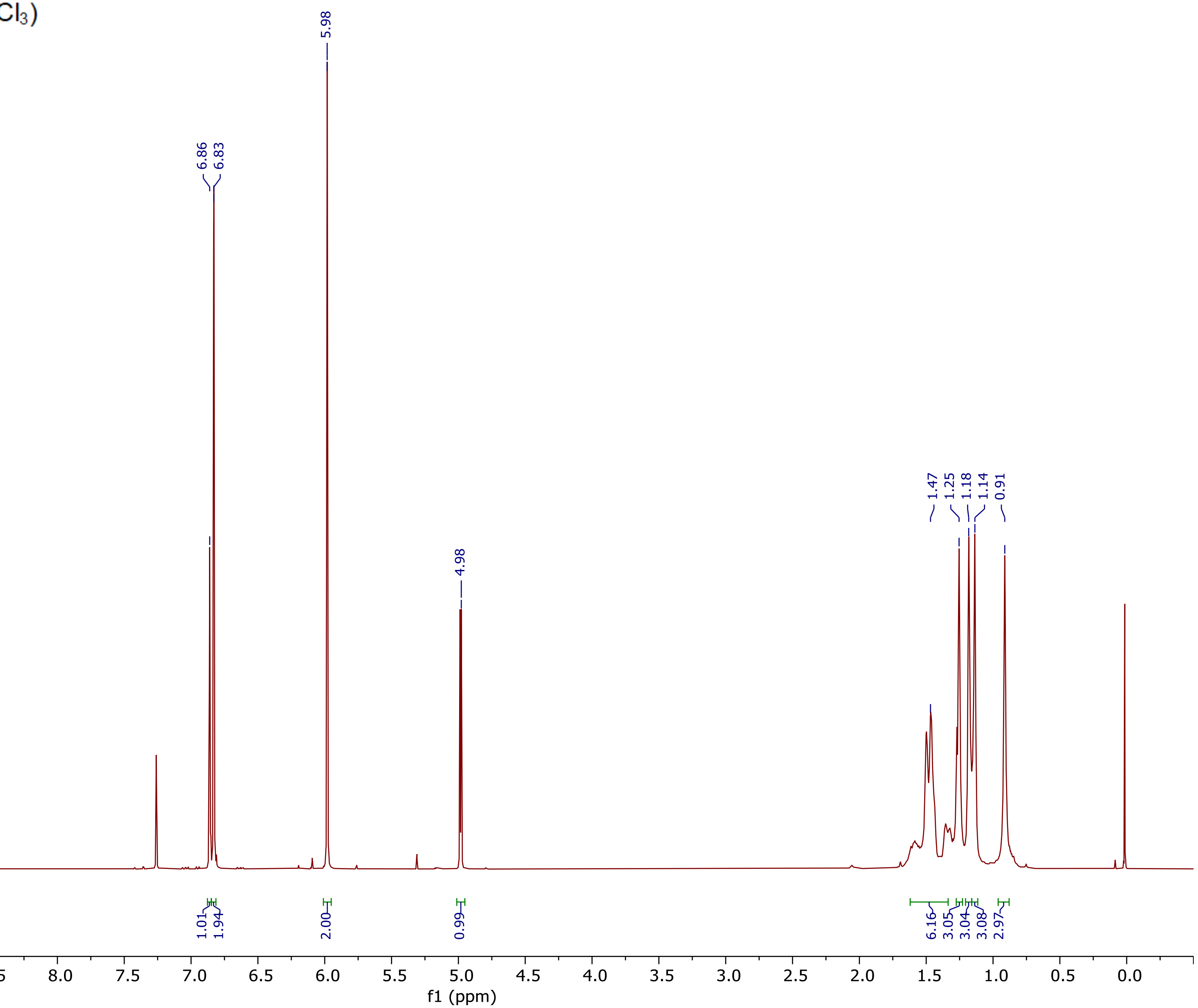

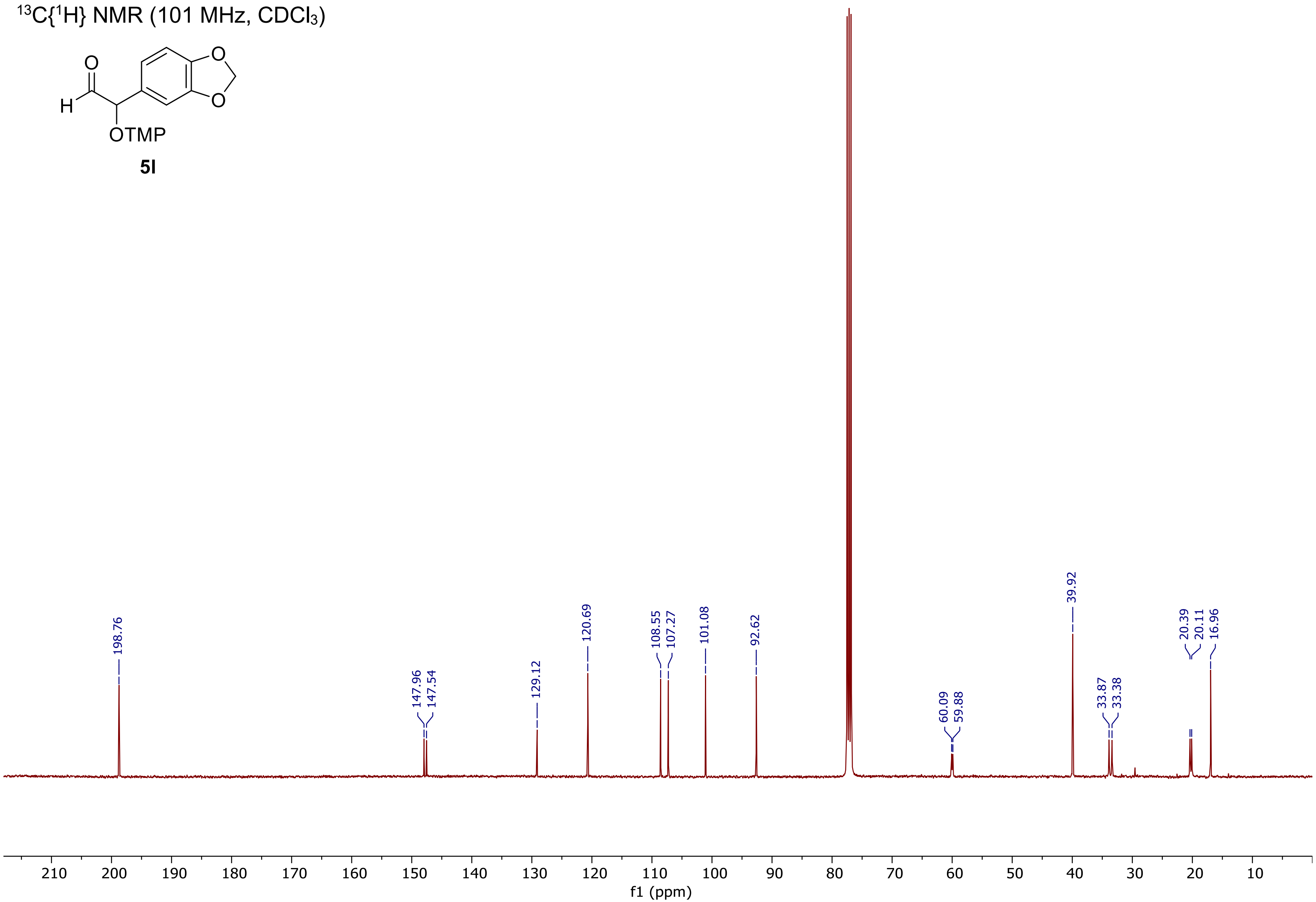

$^{13}C\{^{1}H\}$ NMR (101 MHz, $CDCl_3$)
O
H
O
O
OTMP
5l
198.76
147.96
147.54
129.12
120.69
108.55
107.27
101.08
92.62
60.09
59.88
39.92
33.87
33.38
20.39
20.11
16.96
210
200
190
180
170
160
150
140
130
120
110
100
90
80
70
60
50
40
30
20
10
f1 (ppm)

2D $^{1}H$ -$^{1}H$ COSY (400 MHz, $CDCl_3$)

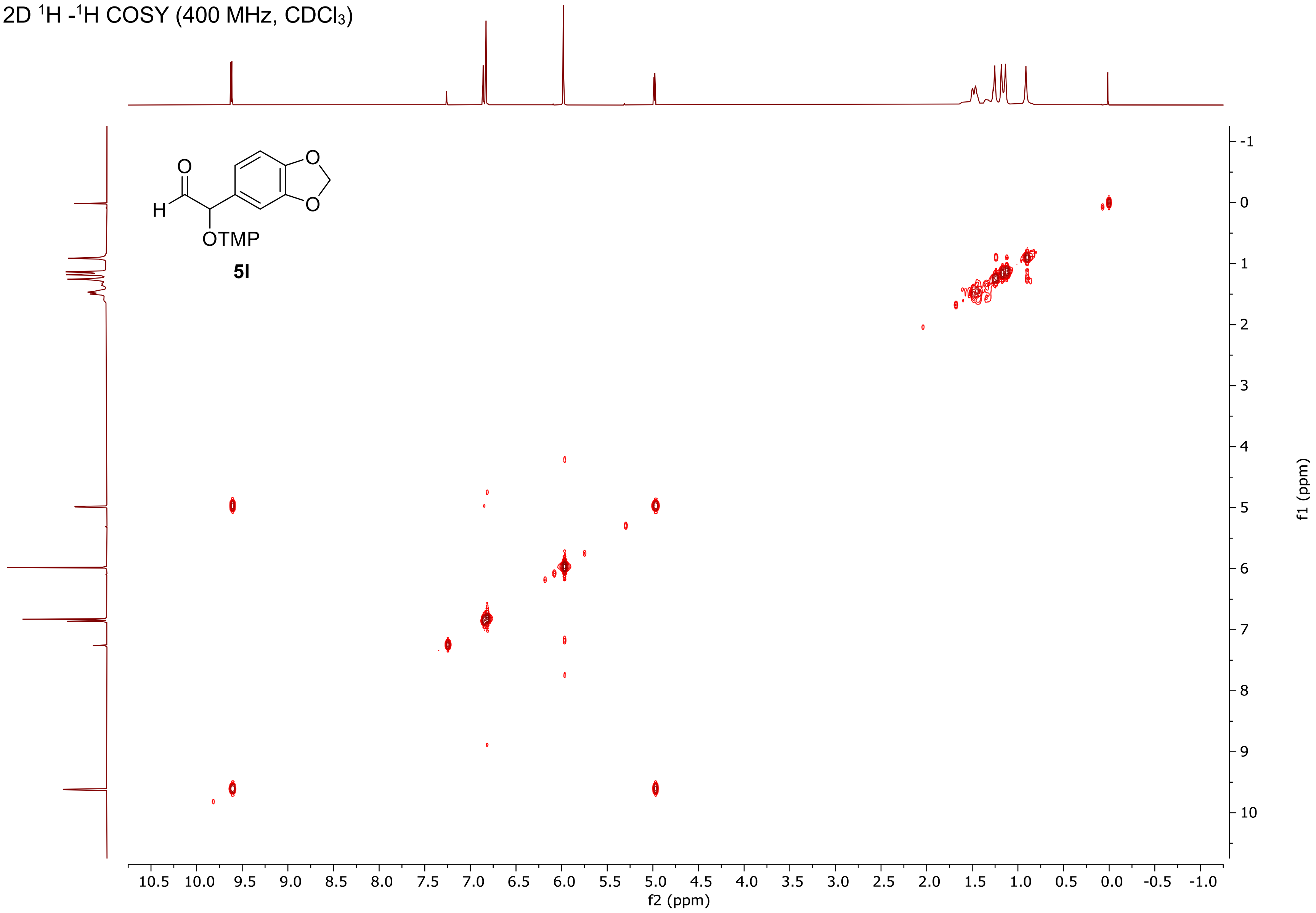

2D $^{1}H$ -$^{13}C$ HSQC (400 MHz, $CDCl_3$)

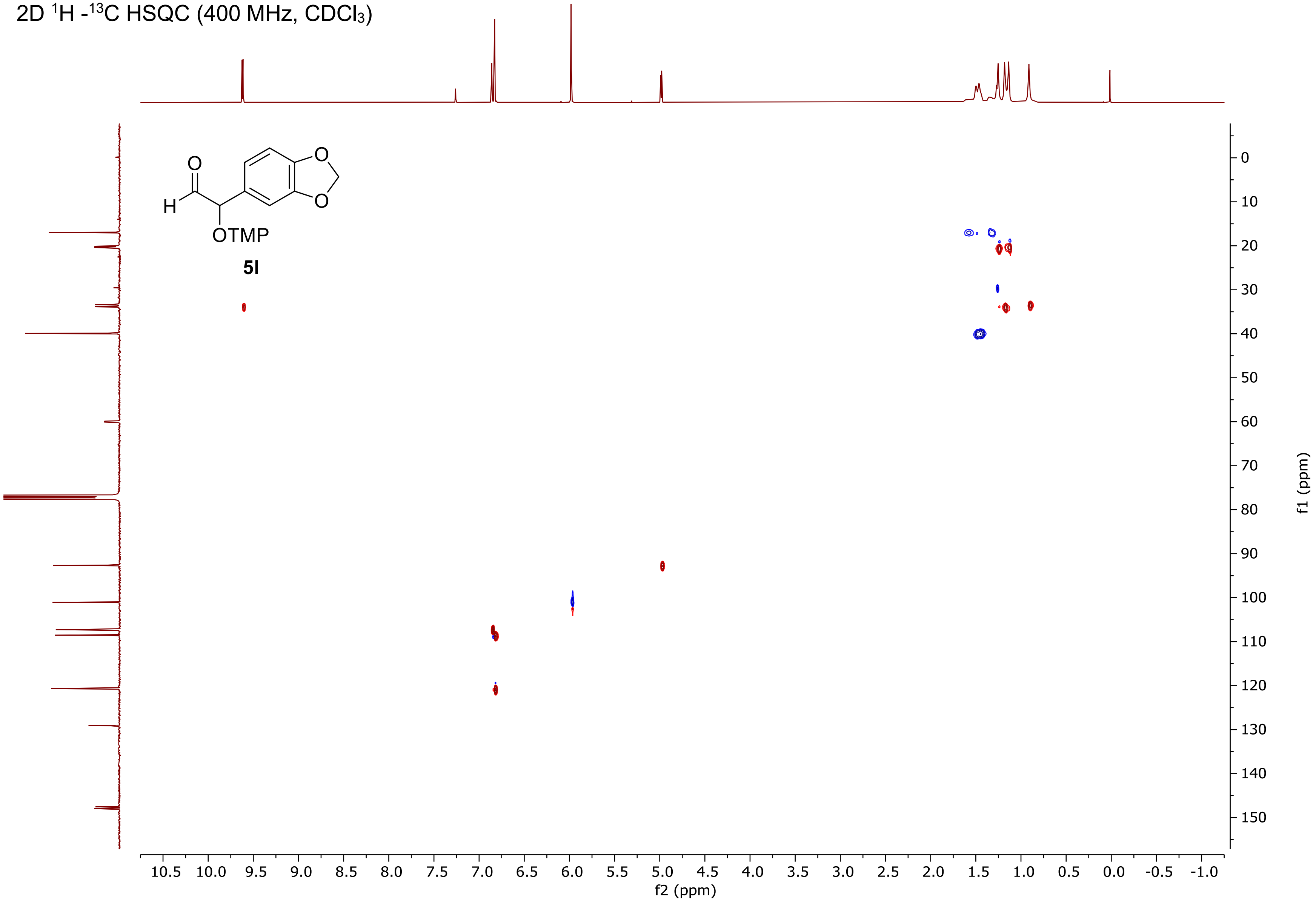

$^{1}H$ NMR (400 MHz, $CDCl_3$)

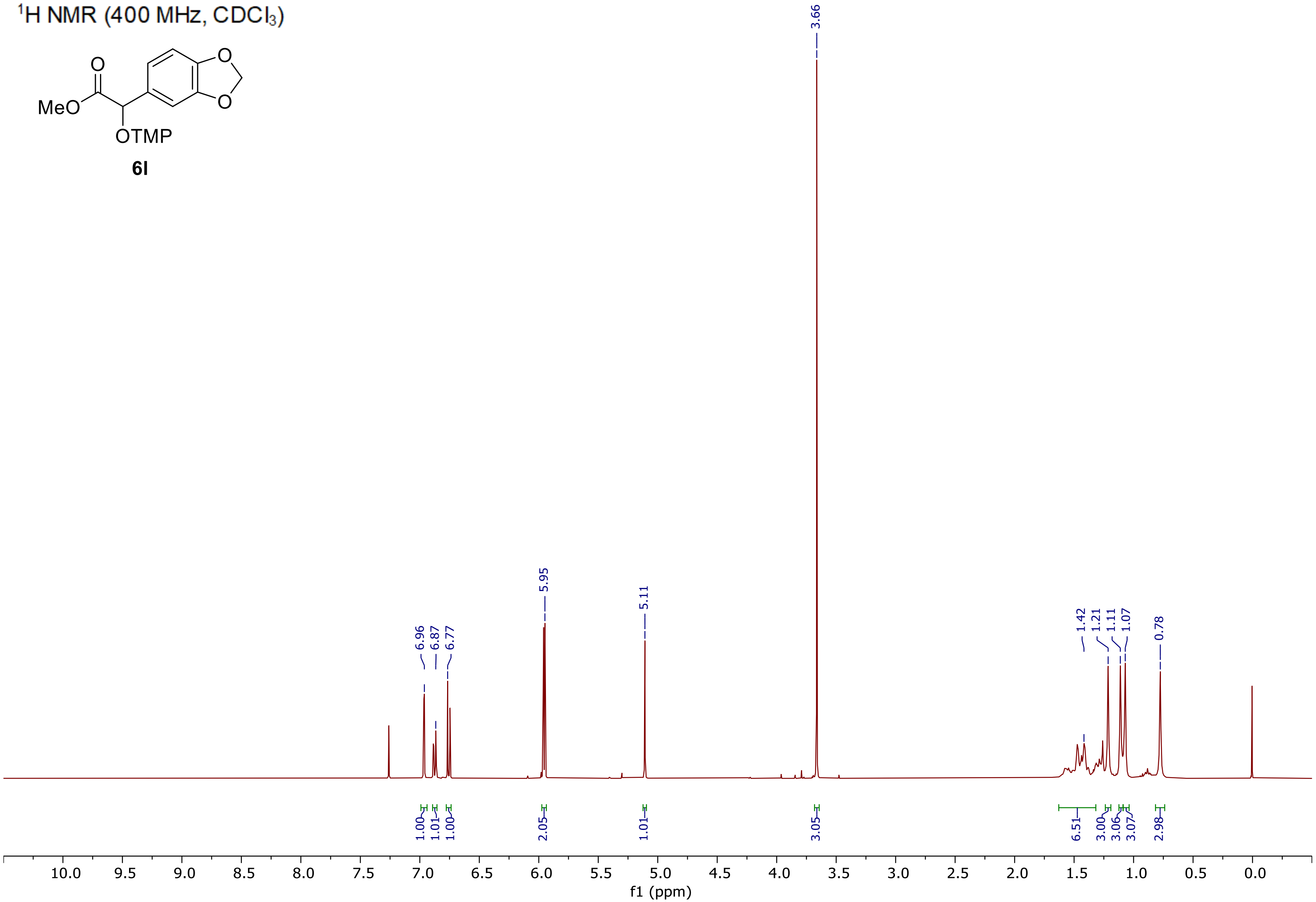

$^{13}C\{^{1}H\}$ NMR (101 MHz, $CDCl_3$)

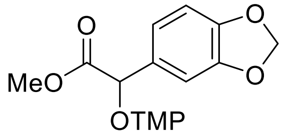


**6l**

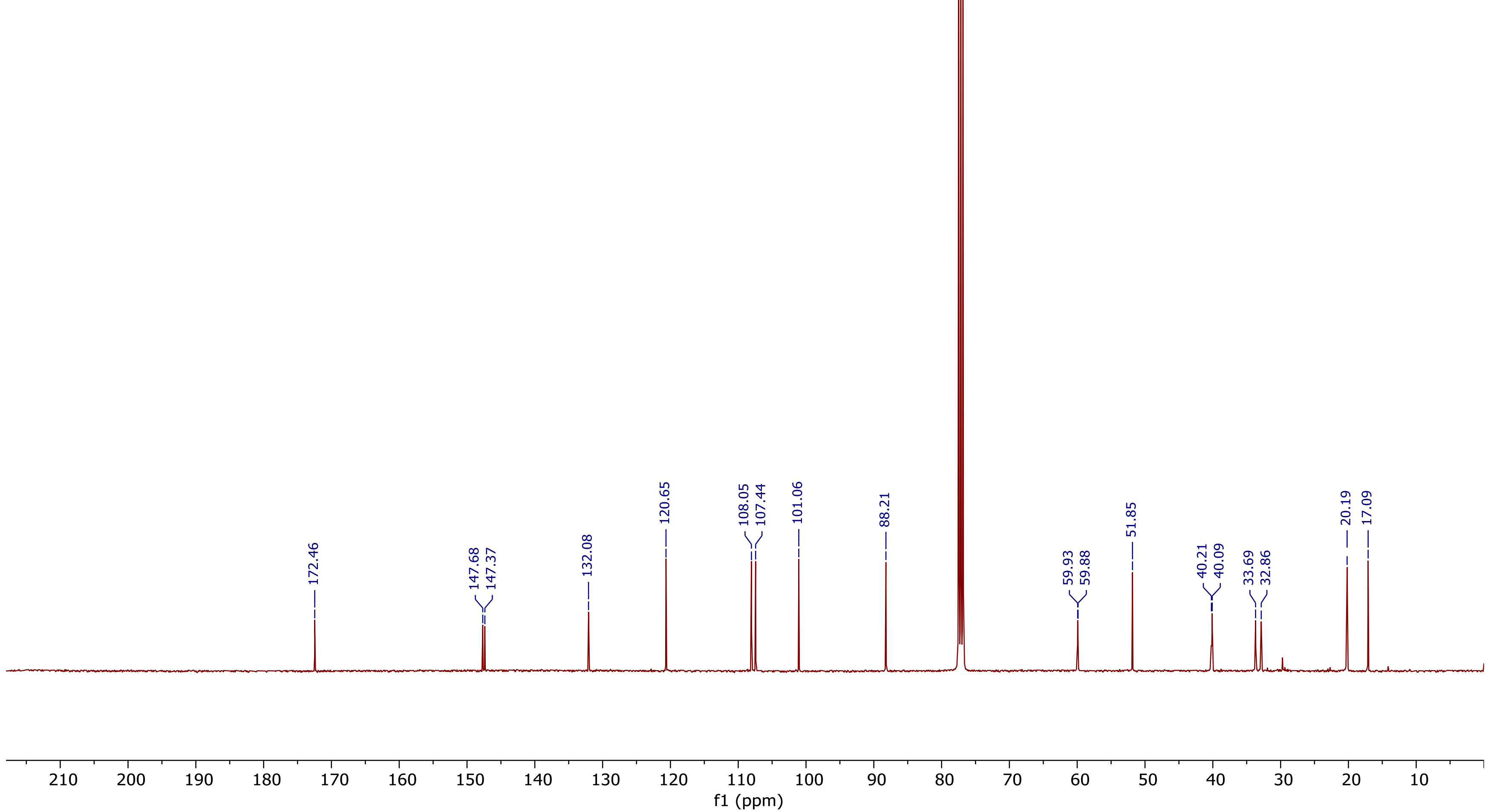

2D $^1$H -$^1$H COSY (400 MHz, $CDCl_3$)

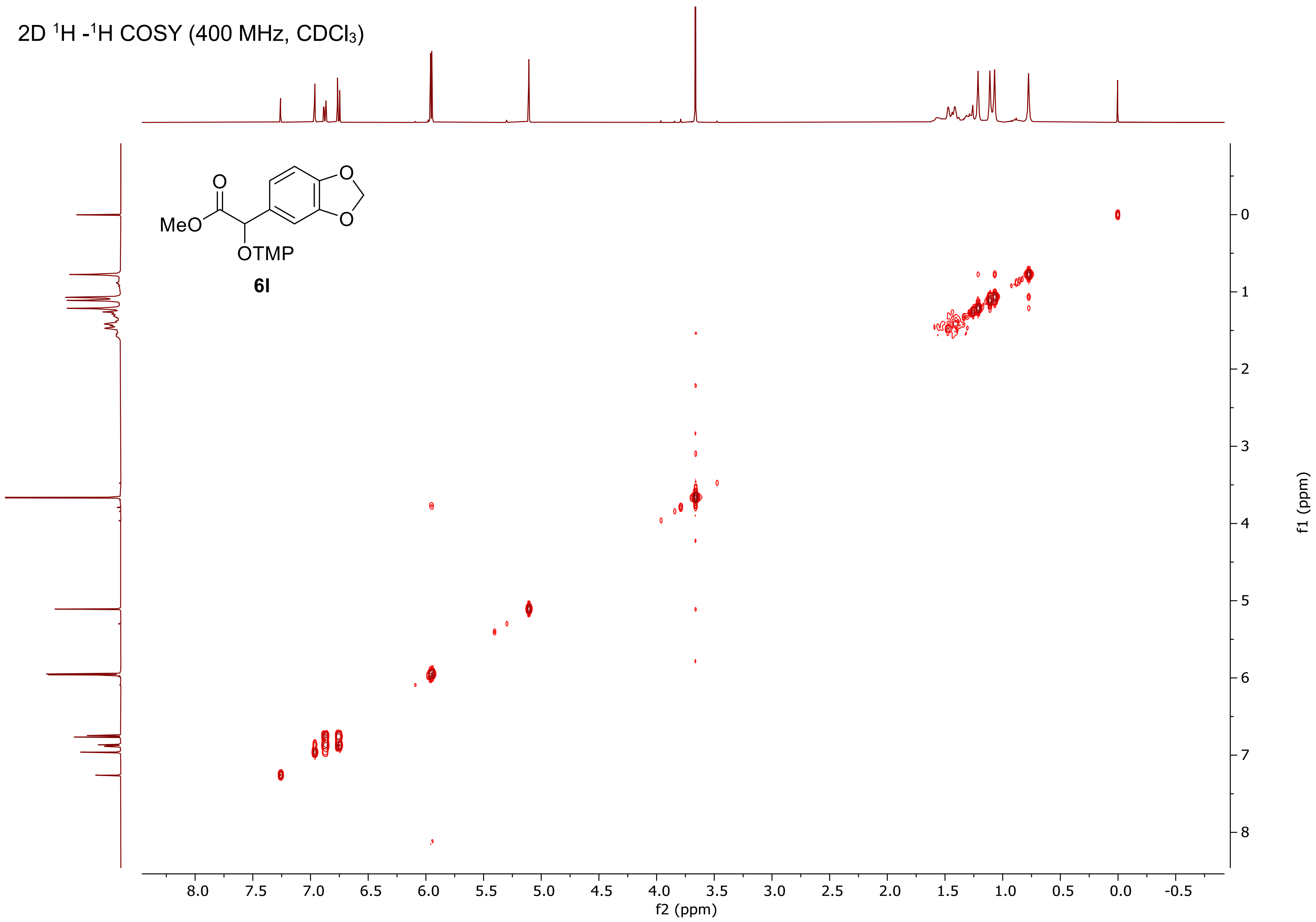

2D $^{1}$H -$^{13}$C HSQC (400 MHz, $CDCl_3$)

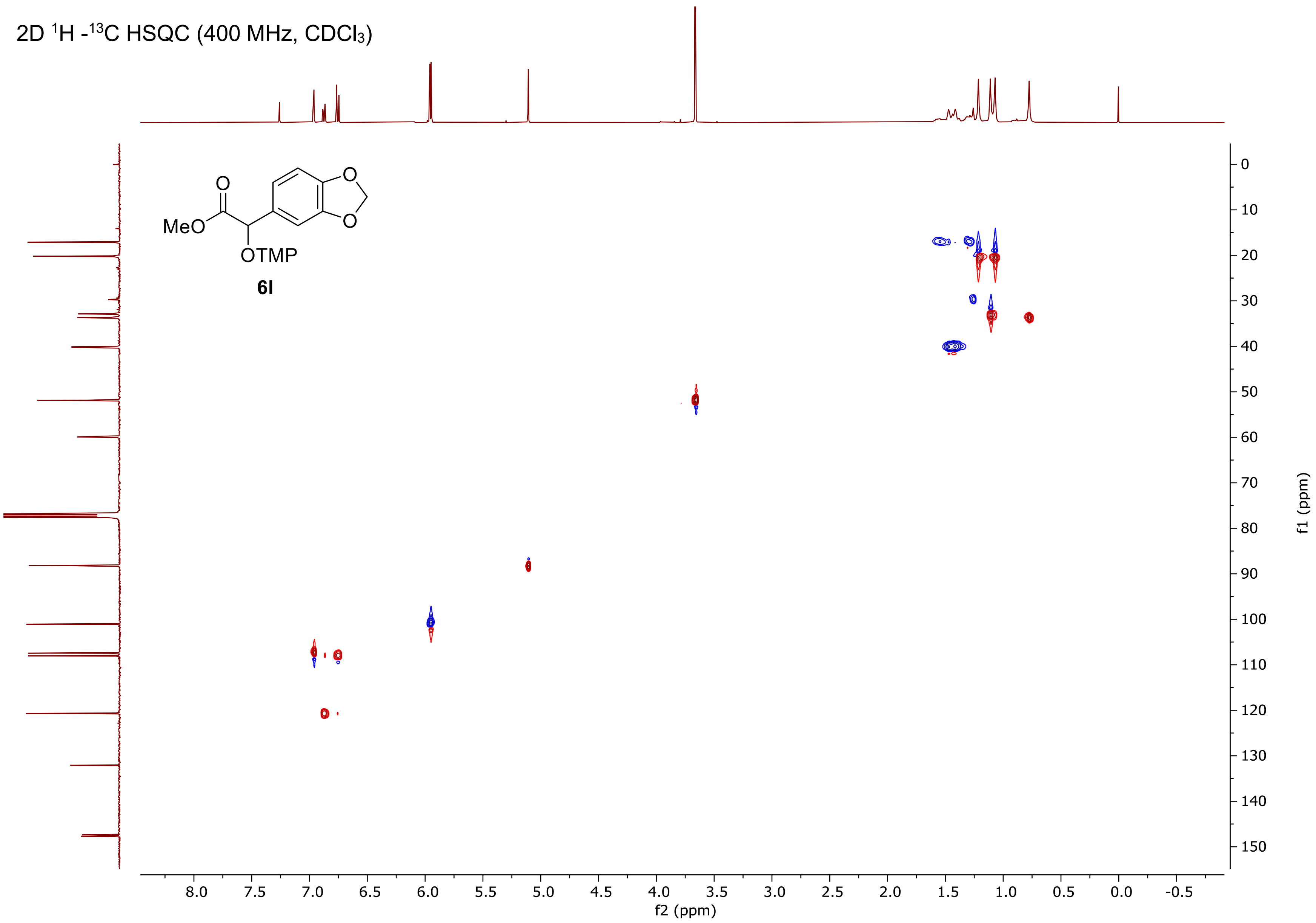

[1]H NMR (400 MHz, $CDCl_3$)

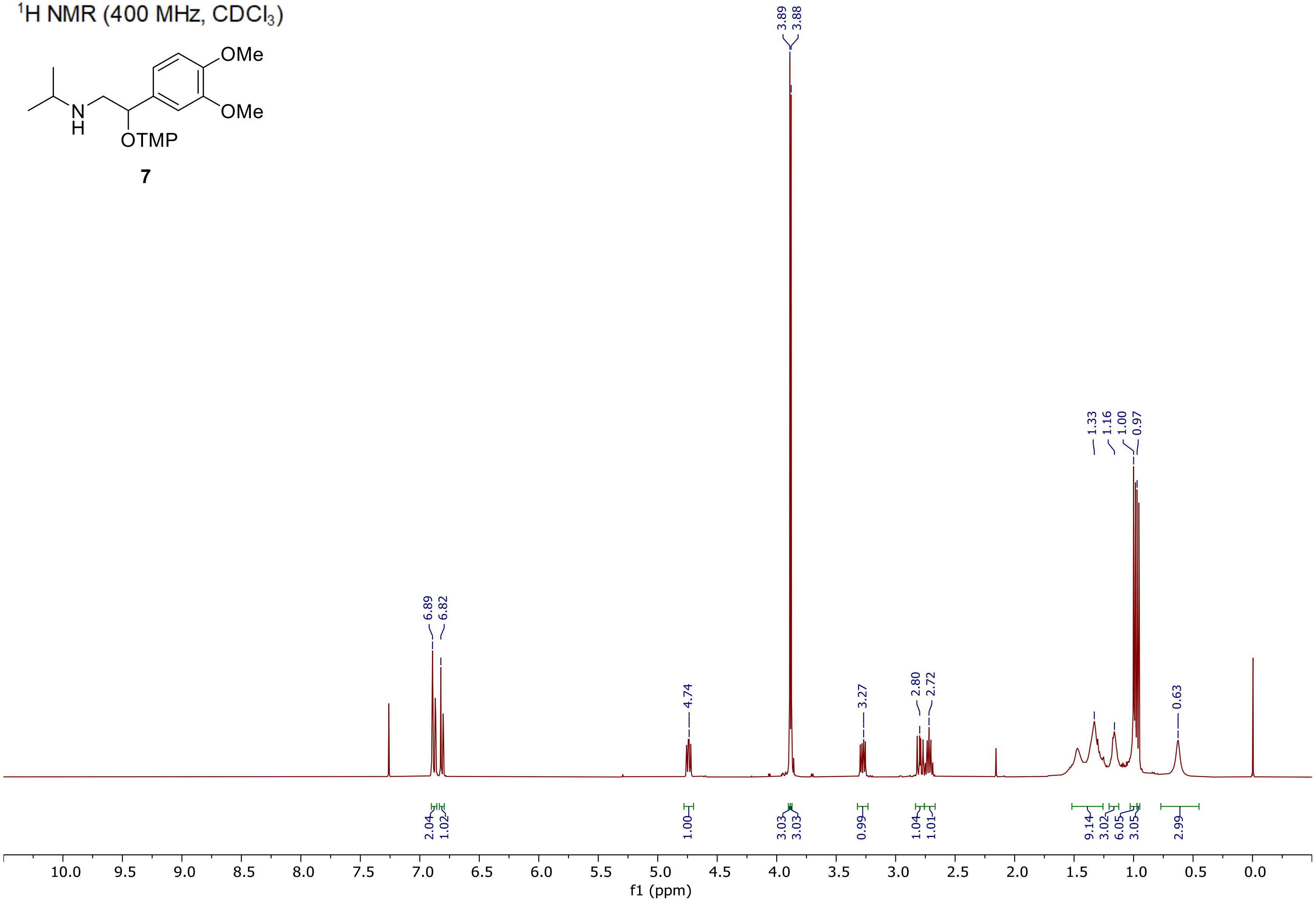

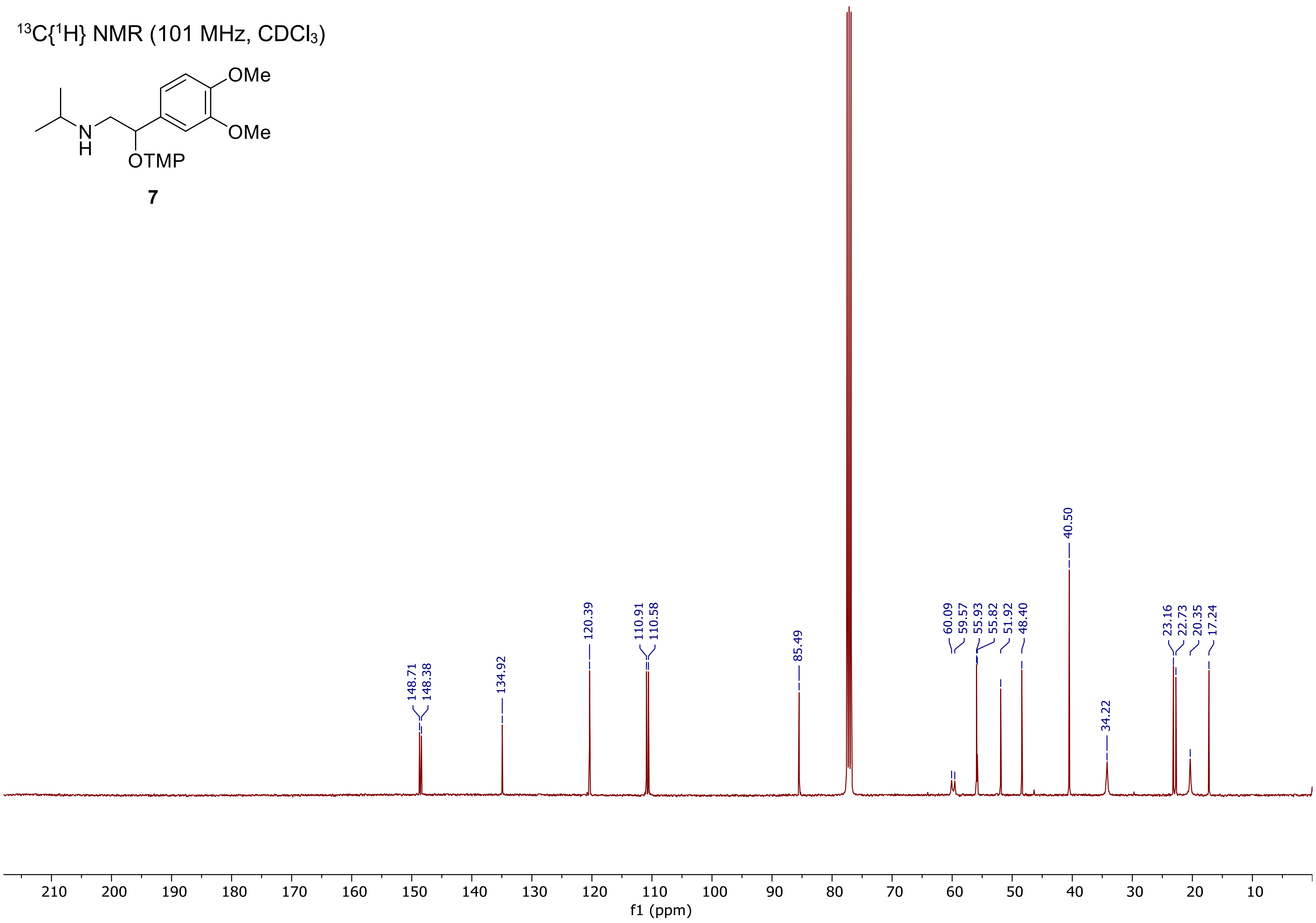

13C{1H} NMR (101 MHz, CDCl3)
OMe
OMe
N
H
OTMP
7
148.71
148.38
134.92
120.39
110.91
110.58
85.49
60.09
59.57
55.93
55.82
51.92
48.40
40.50
34.22
23.16
22.73
20.35
17.24
210
200
190
180
170
160
150
140
130
120
110
100
90
80
70
60
50
40
30
20
10
f1 (ppm)

2D $^1H$ -$^1H$ COSY (400 MHz, $CDCl_3$)

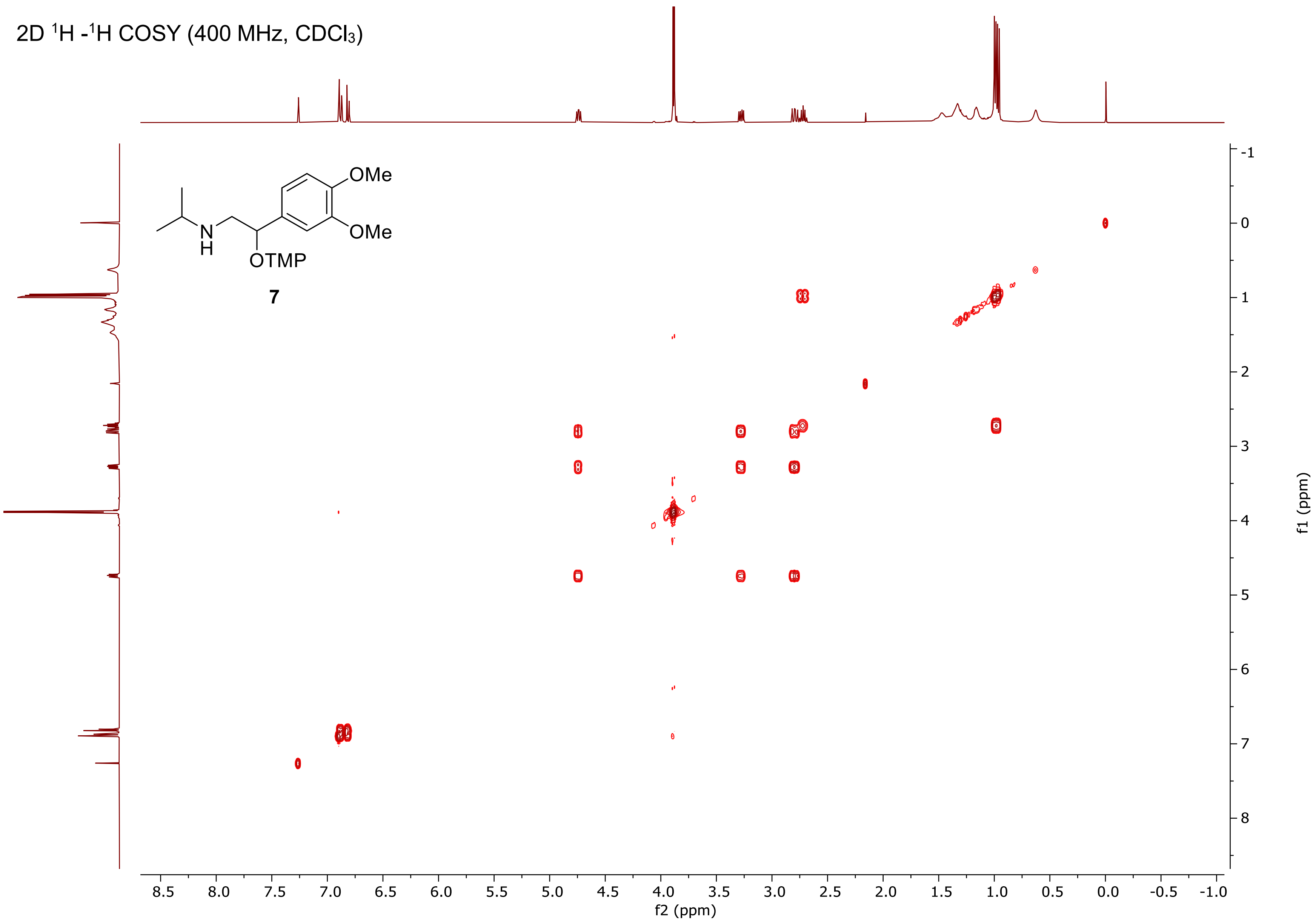

2D $^1$H -$^{13}$C HSQC (400 MHz, $CDCl_3$)

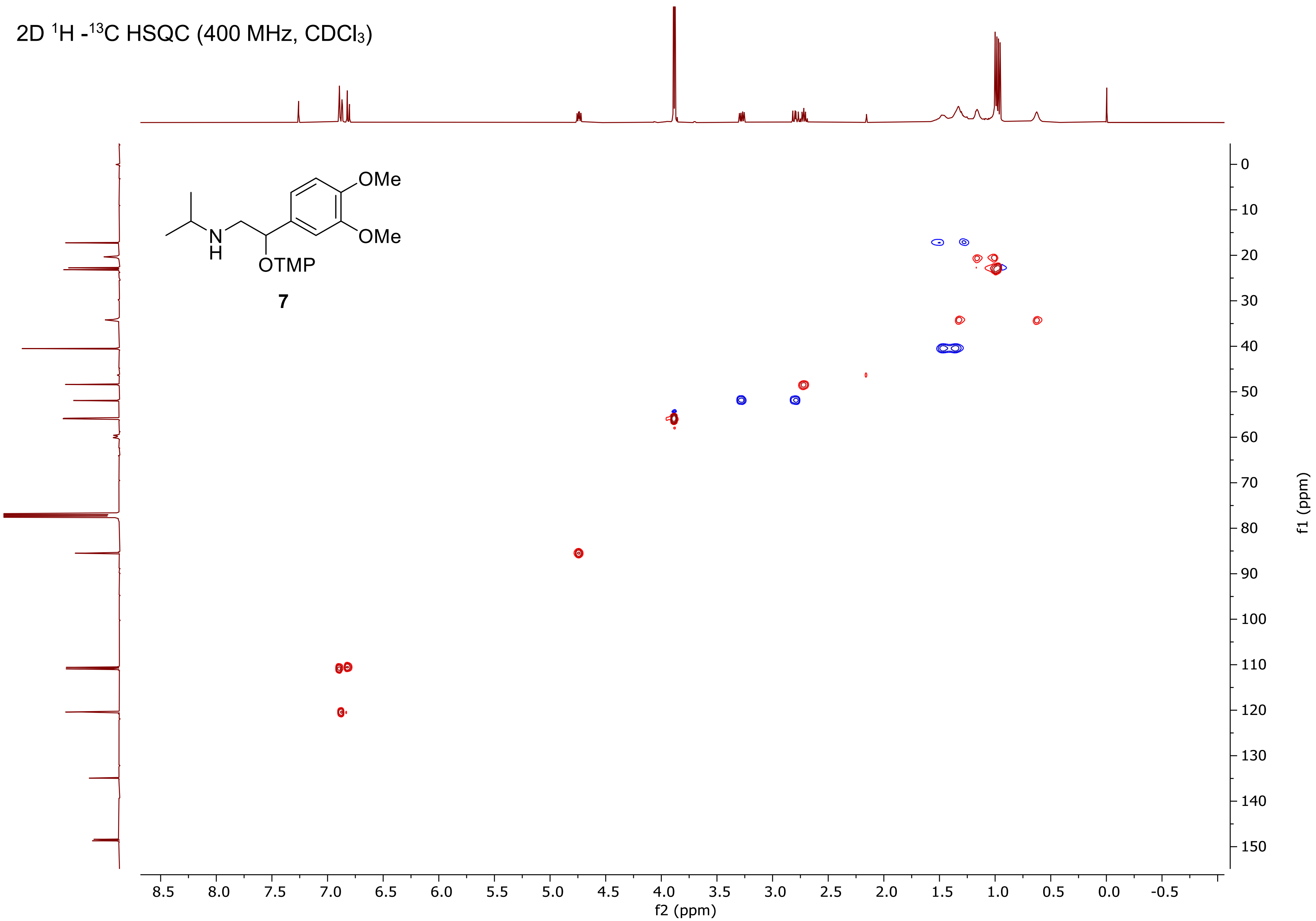

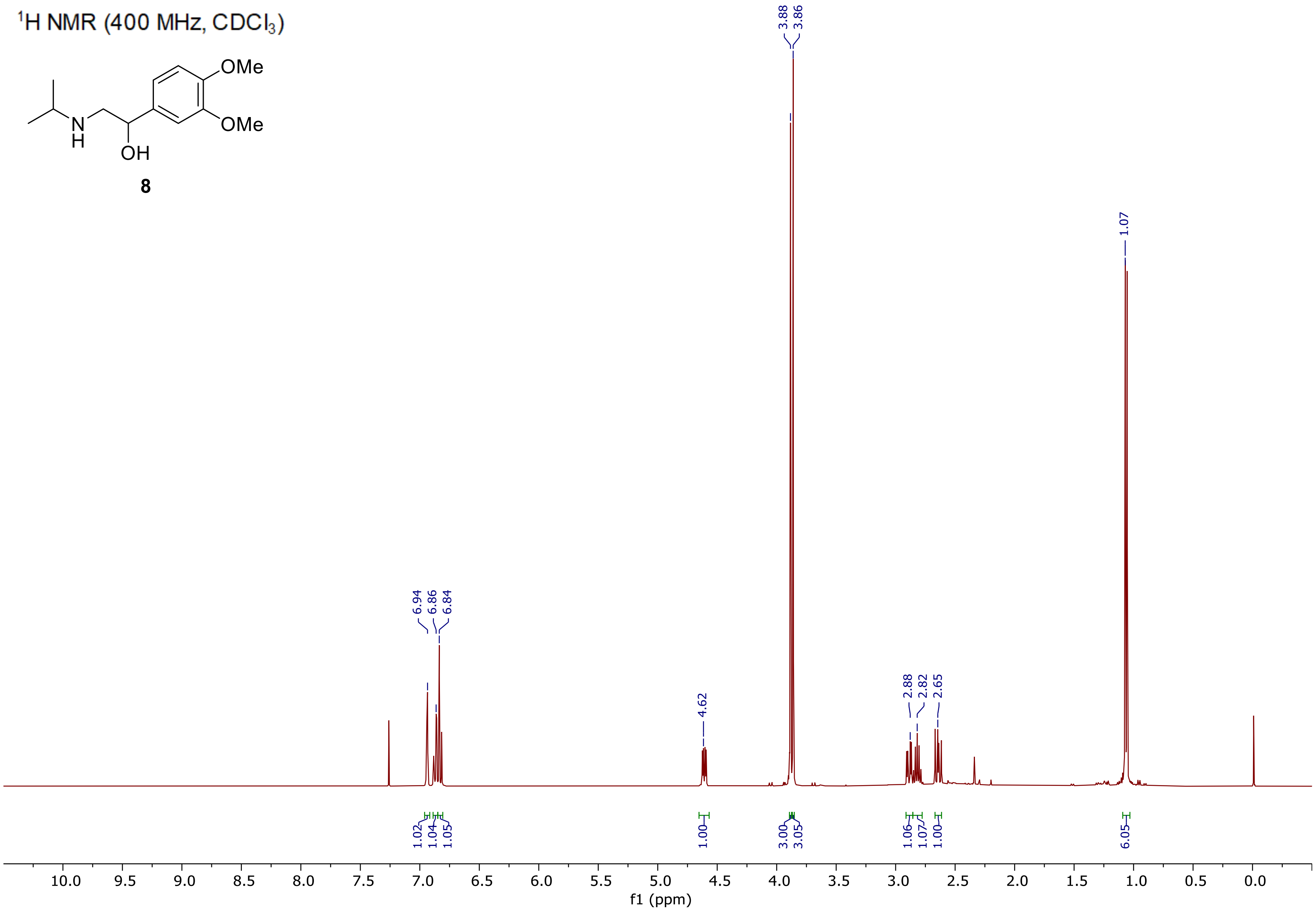

[1]H NMR (400 MHz, CDCl3)
OMe
OMe
N
H
OH
8
3.88
3.86
1.07
6.94
6.86
6.84
4.62
2.88
2.82
2.65
1.02
1.04
1.05
1.00
3.00
3.05
1.06
1.07
1.00
6.05
10.0
9.5
9.0
8.5
8.0
7.5
7.0
6.5
6.0
5.5
5.0
4.5
4.0
3.5
3.0
2.5
2.0
1.5
1.0
0.5
0.0
f1 (ppm)

$^{13}C\{^{1}H\}$ NMR (101 MHz, $CDCl_3$)

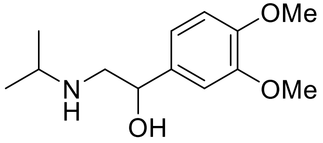


**8**

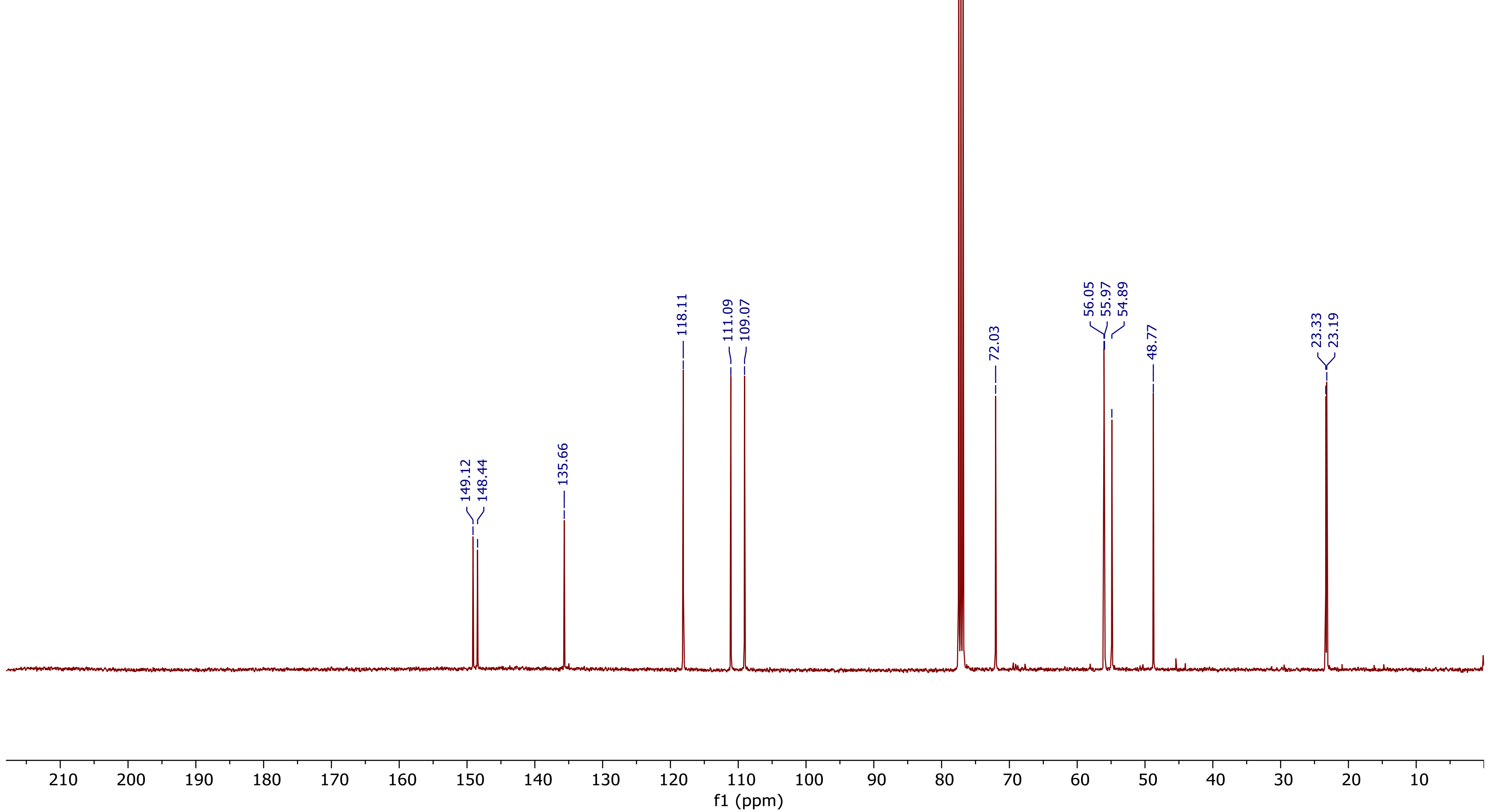

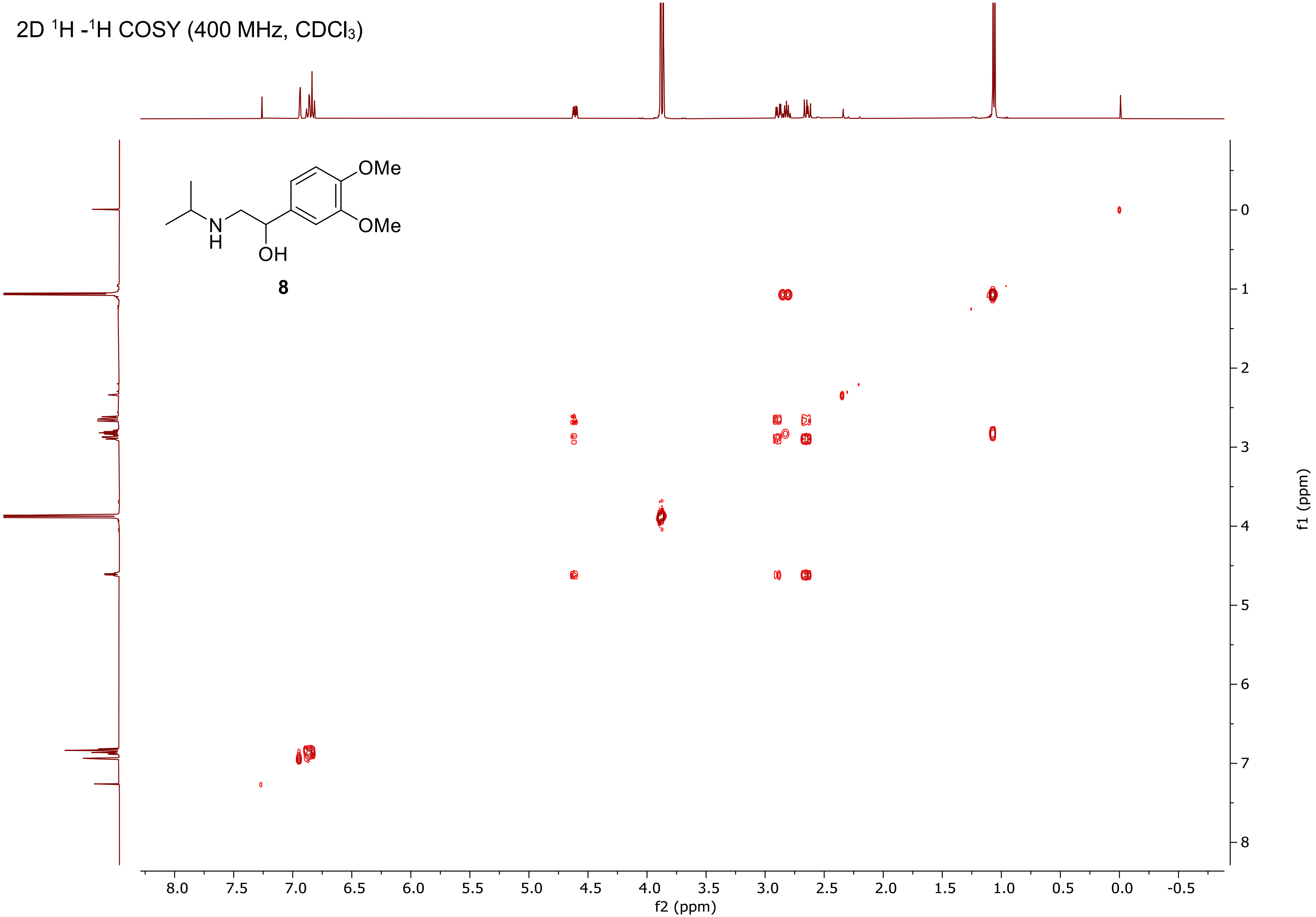
2D ¹H -¹H COSY (400 MHz, CDCl₃)
OMe
OMe
N
H
OH
8
f2 (ppm)
f1 (ppm)

2D $^{1}$H -$^{13}$C HSQC (400 MHz, $CDCl_3$)

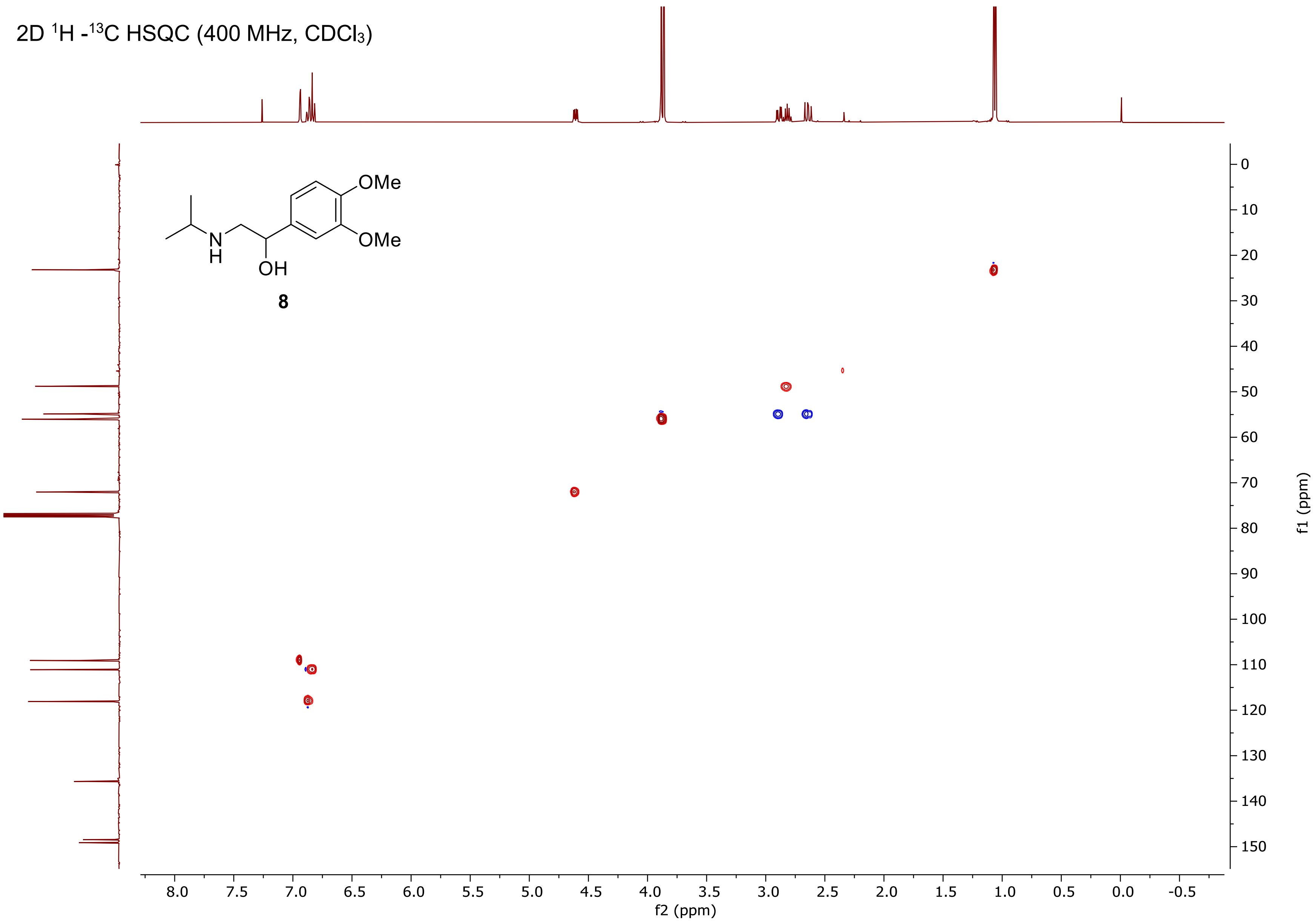

[1]H NMR (400 MHz, $CDCl_3$)

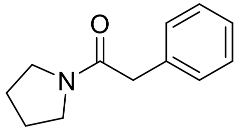

**9a**

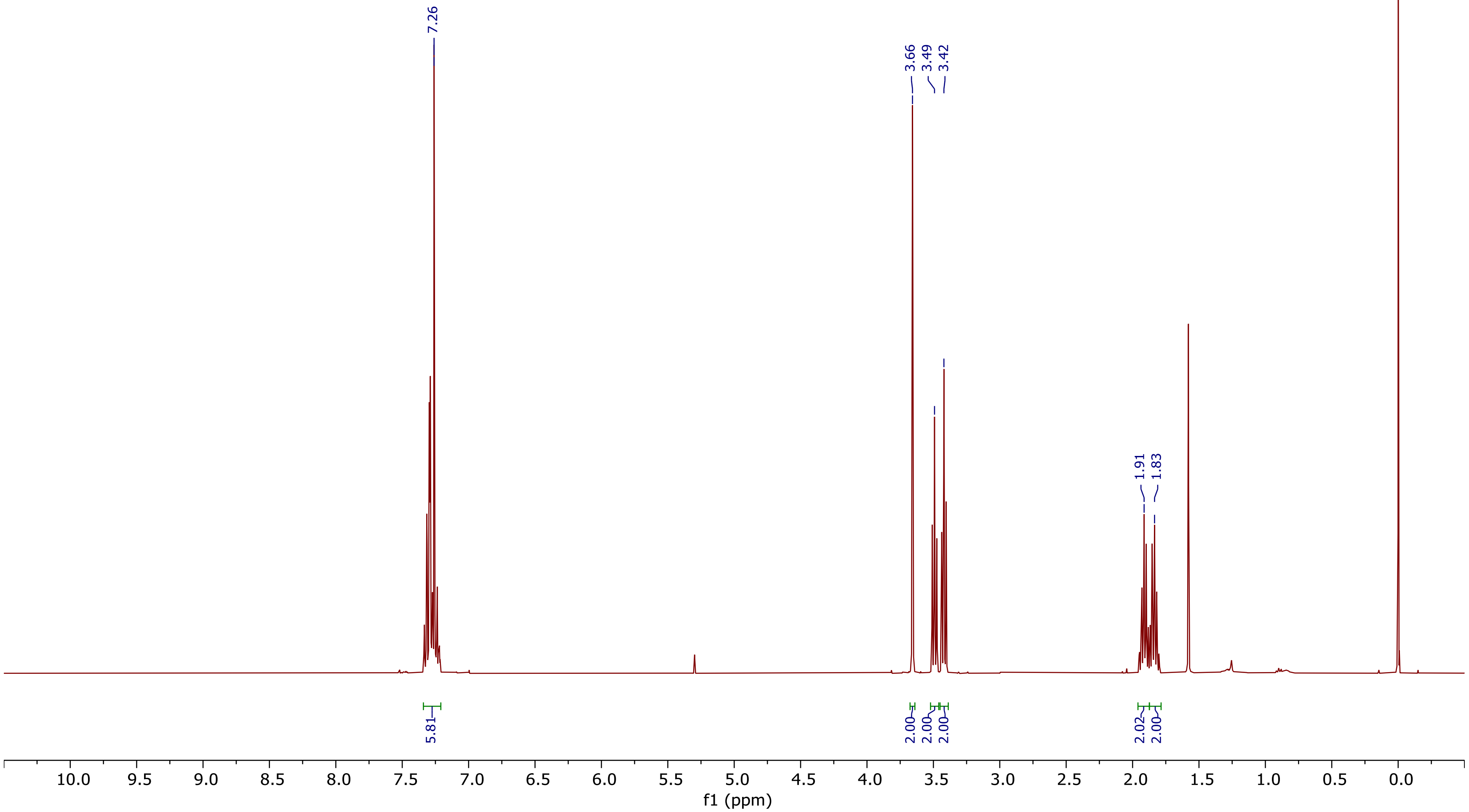

$^{13}C\{^1H\}$ NMR (101 MHz, $CDCl_3$)

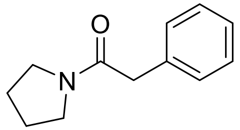

**9a**

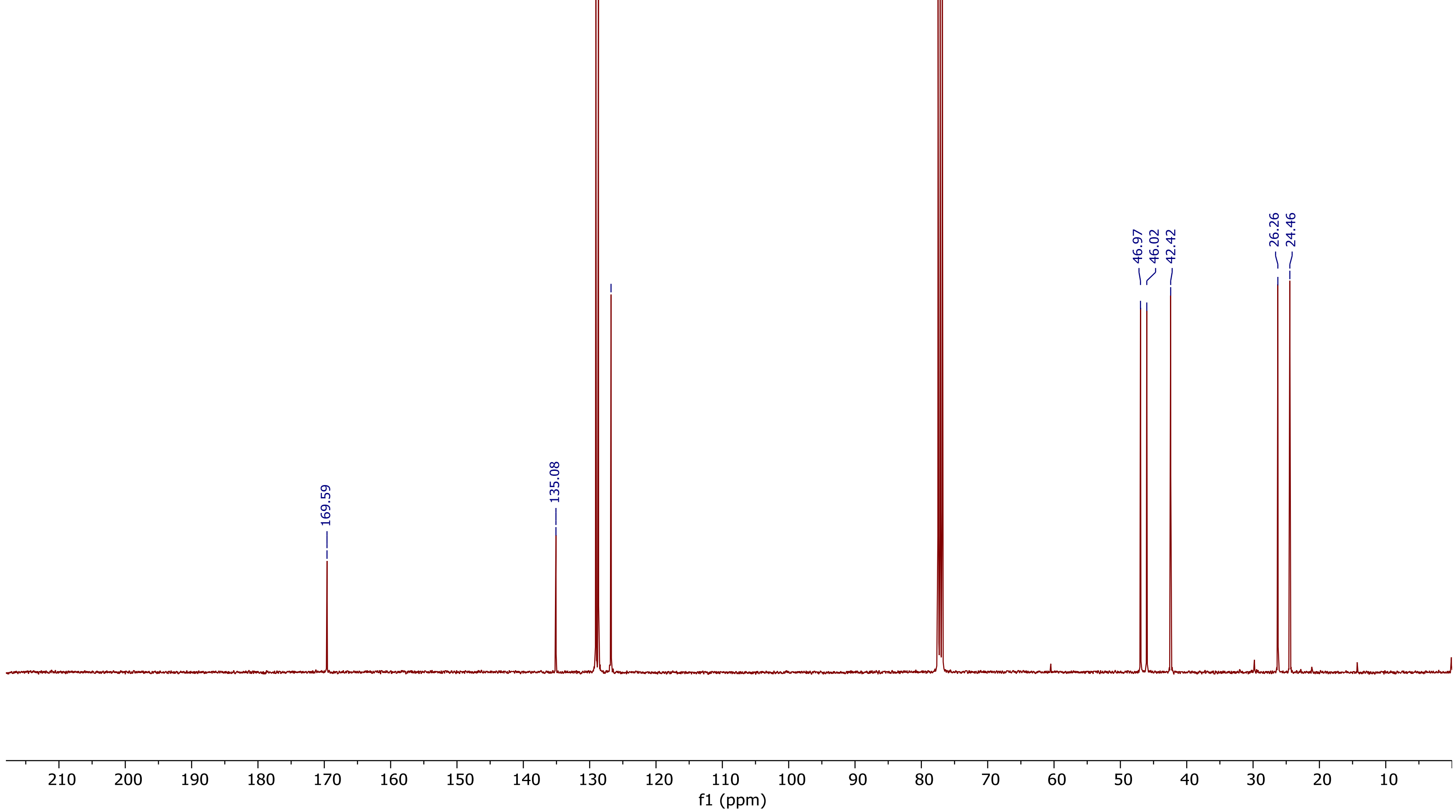

2D $^1$H -$^1$H COSY (400 MHz, $CDCl_3$)

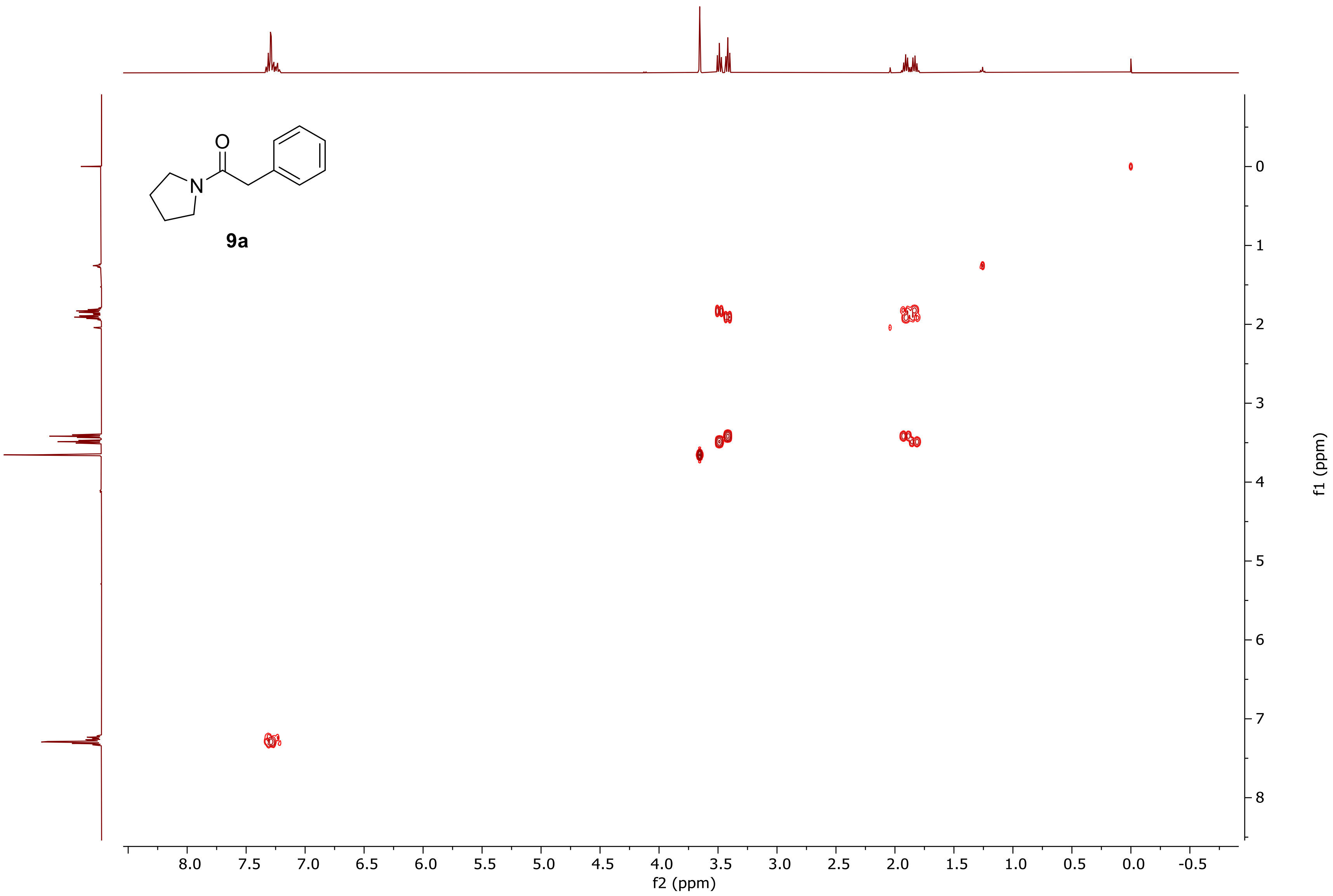


**9a**

2D $^{1}H$ -$^{13}C$ HSQC (400 MHz, $CDCl_3$)

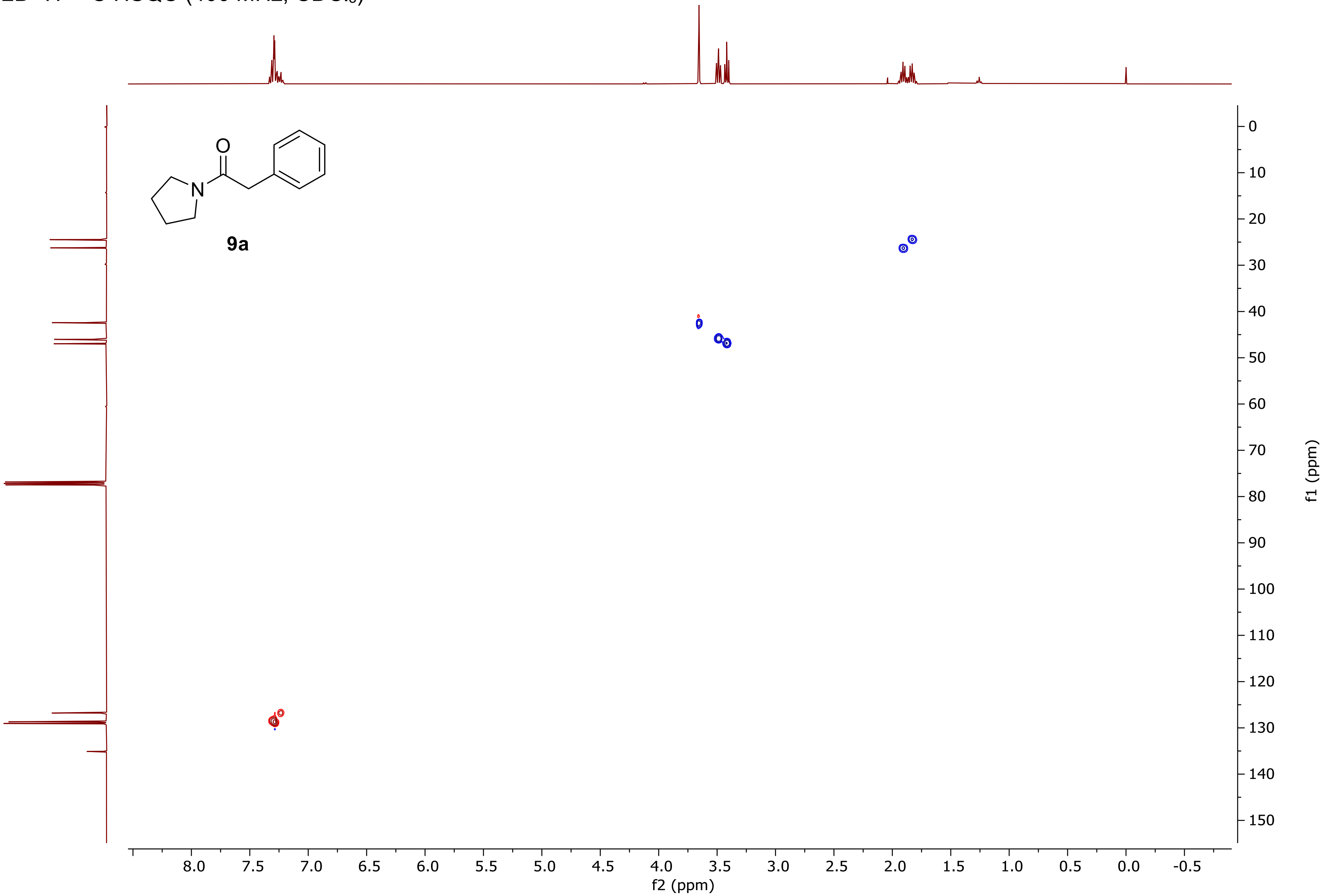

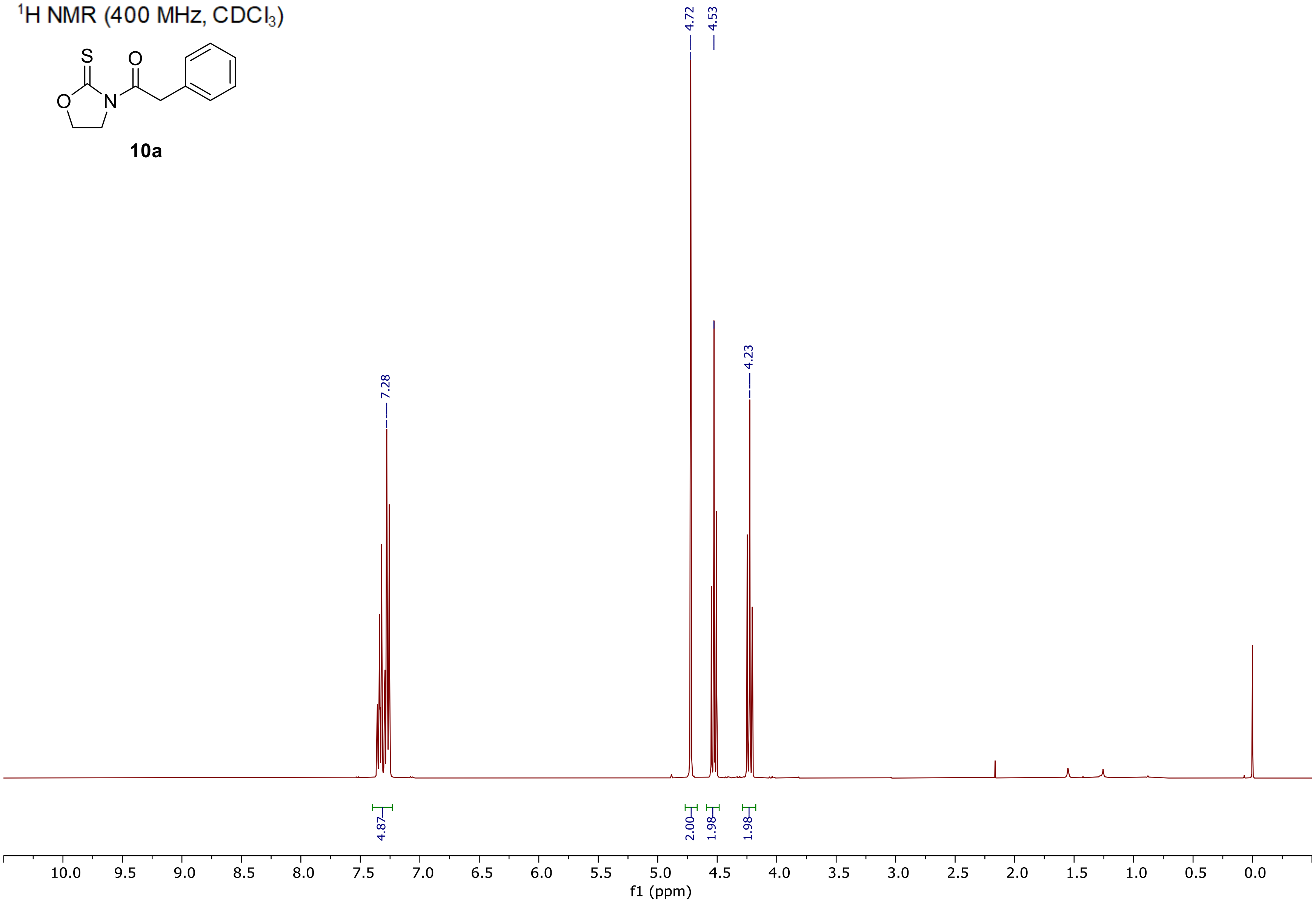
1H NMR (400 MHz, CDCl3)
S
O
O
N
10a
7.28
4.72
4.53
4.23
4.87
2.00
1.98
1.98
10.0
9.5
9.0
8.5
8.0
7.5
7.0
6.5
6.0
5.5
5.0
4.5
4.0
3.5
3.0
2.5
2.0
1.5
1.0
0.5
0.0
f1 (ppm)

$^{13}C\{^{1}H\}$ NMR (101 MHz, $CDCl_3$)

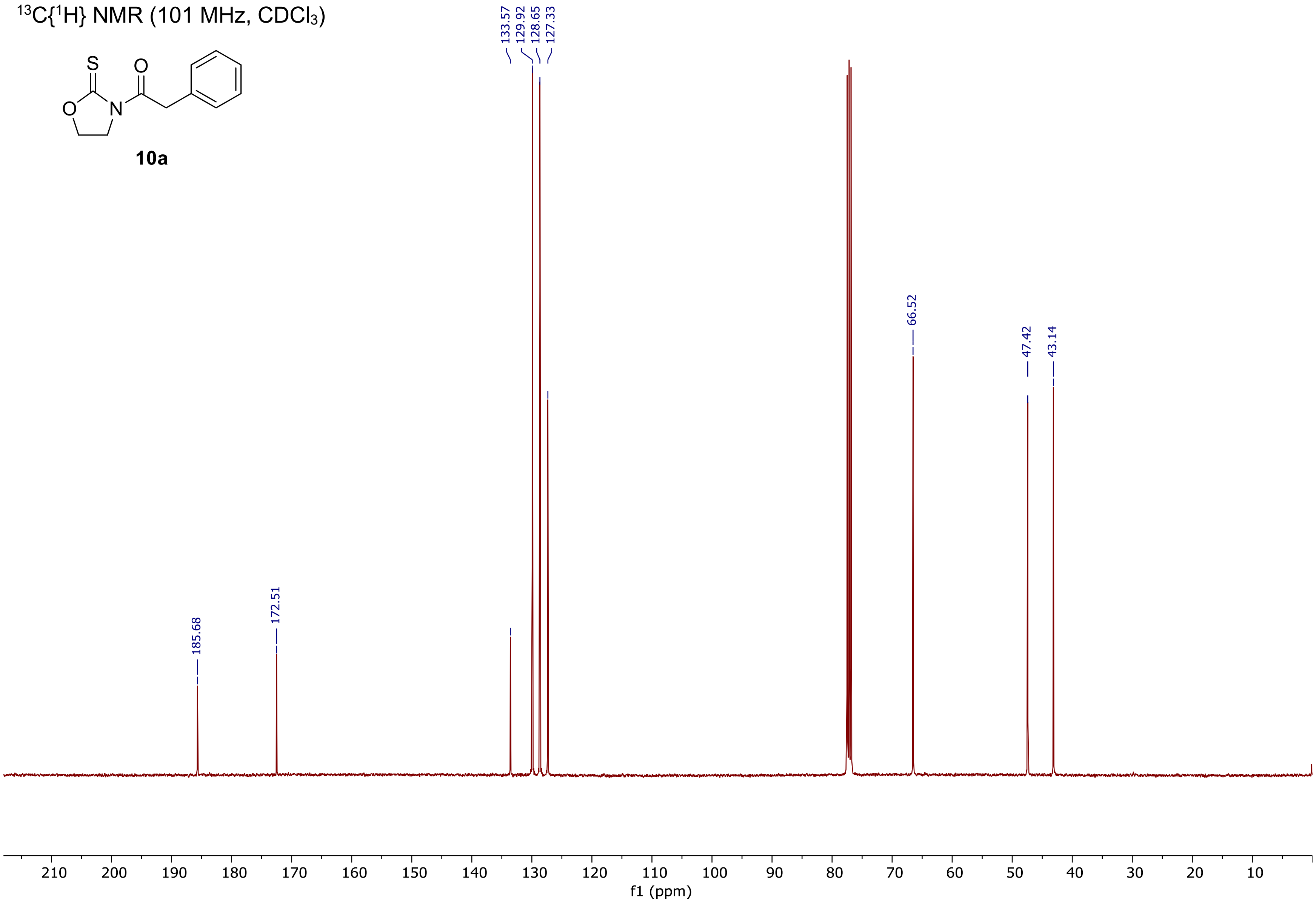

2D $^1$H -$^1$H COSY (400 MHz, $CDCl_3$)

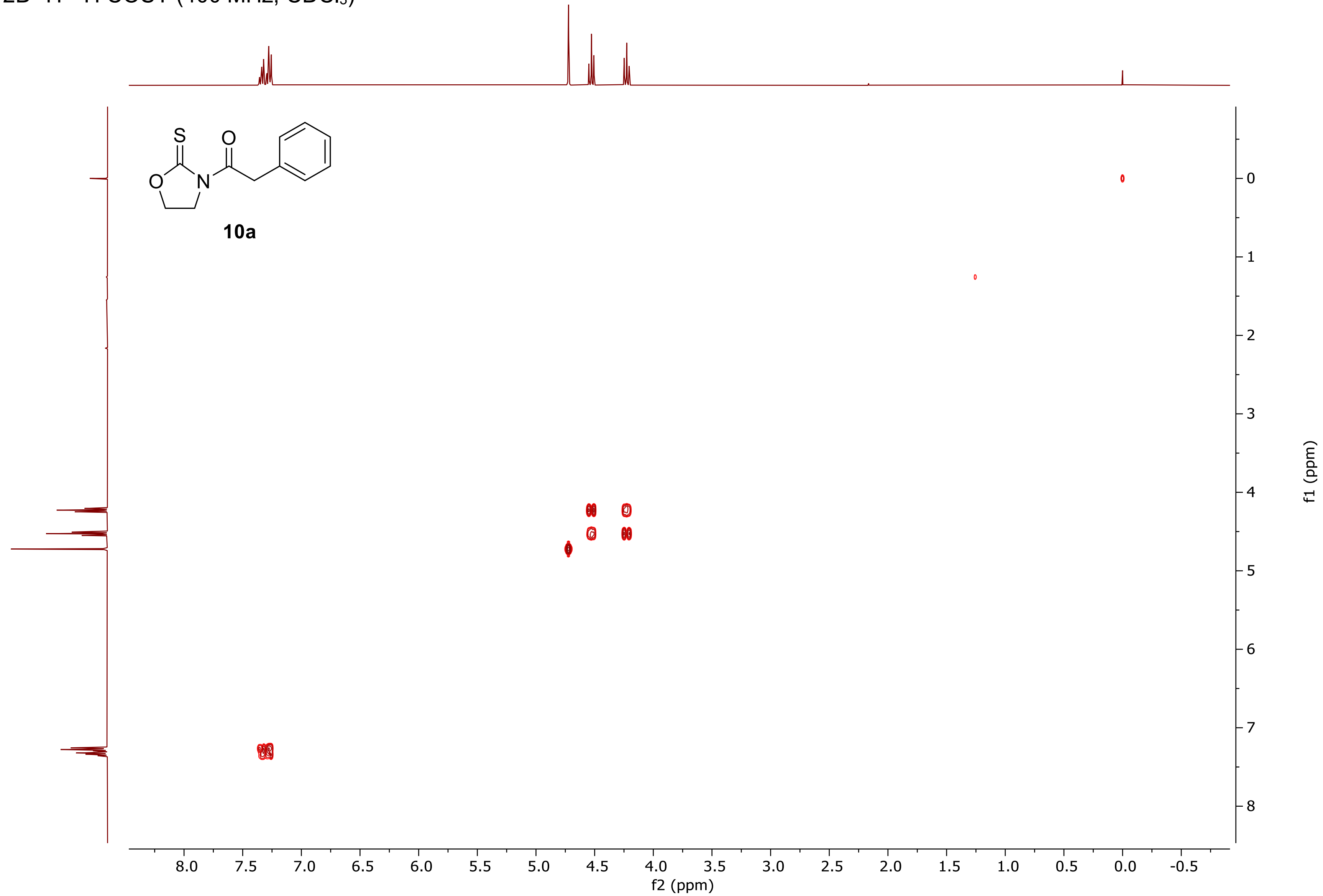

2D $^1$H -$^{13}$C HSQC (400 MHz, $CDCl_3$)

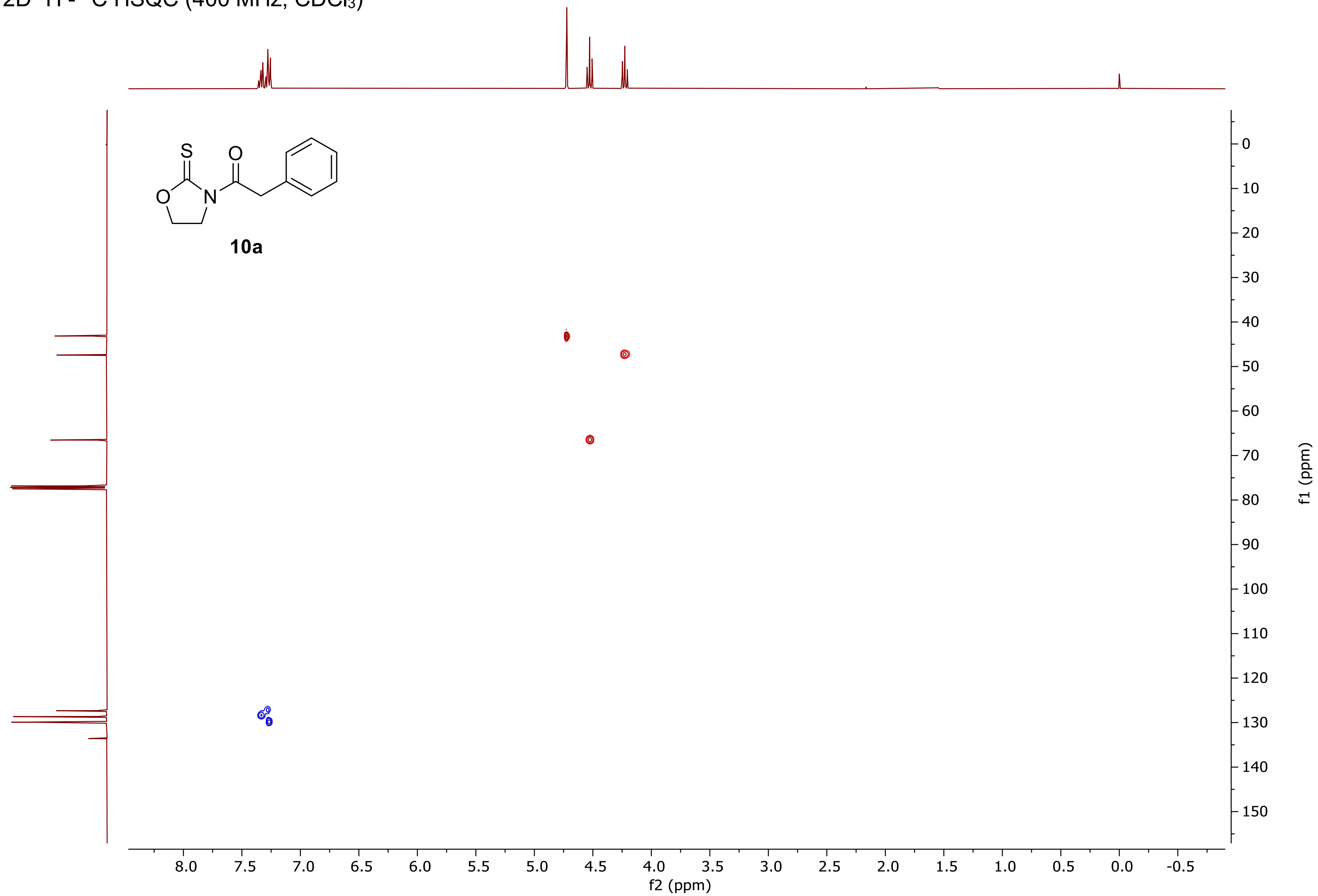

$^1H$ NMR (400 MHz, $CDCl_3$)

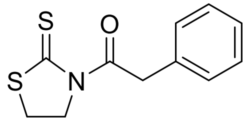

**11a**

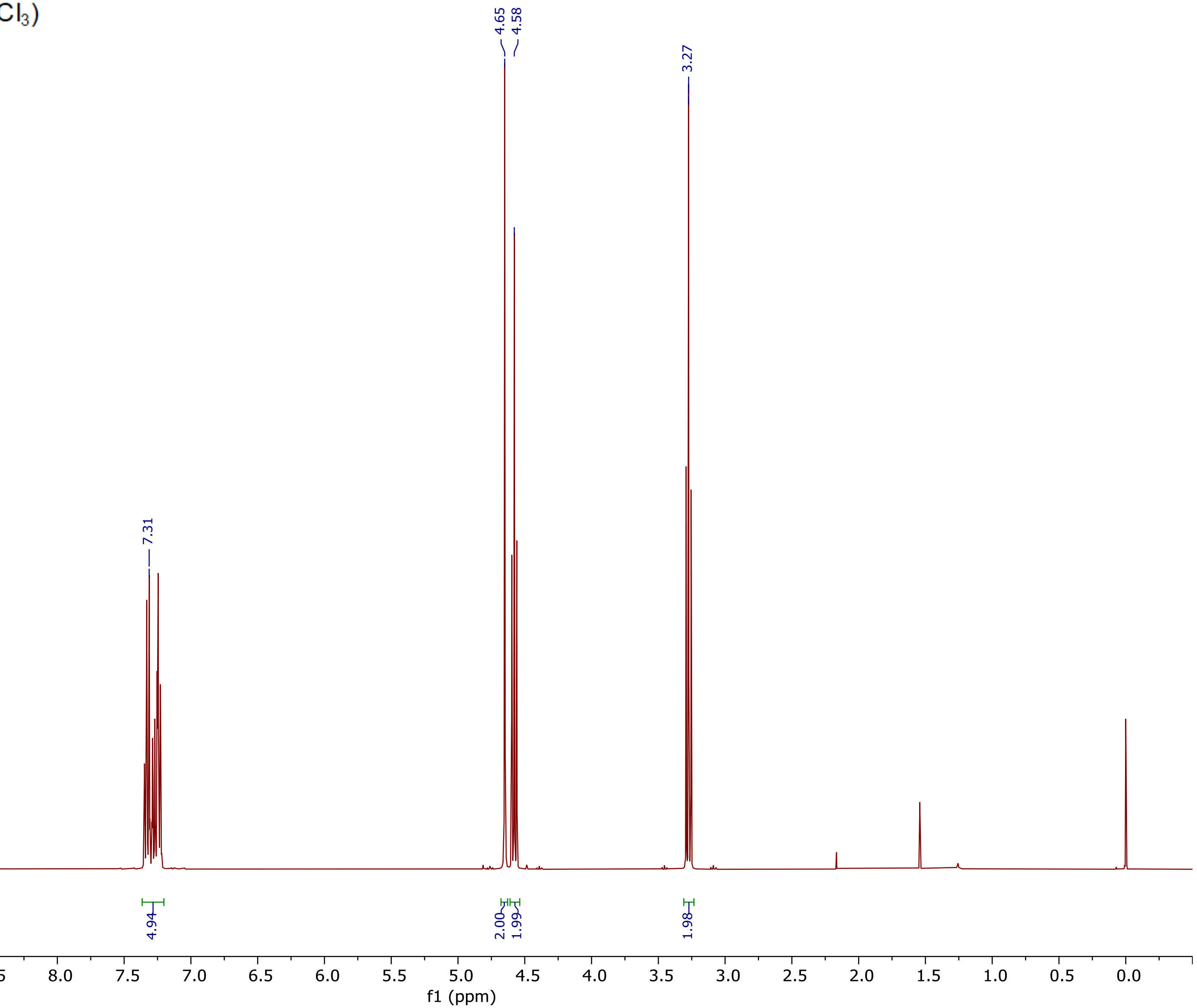

$^{13}C\{^{1}H\}$ NMR (101 MHz, $CDCl_3$)

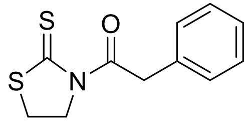


**11a**

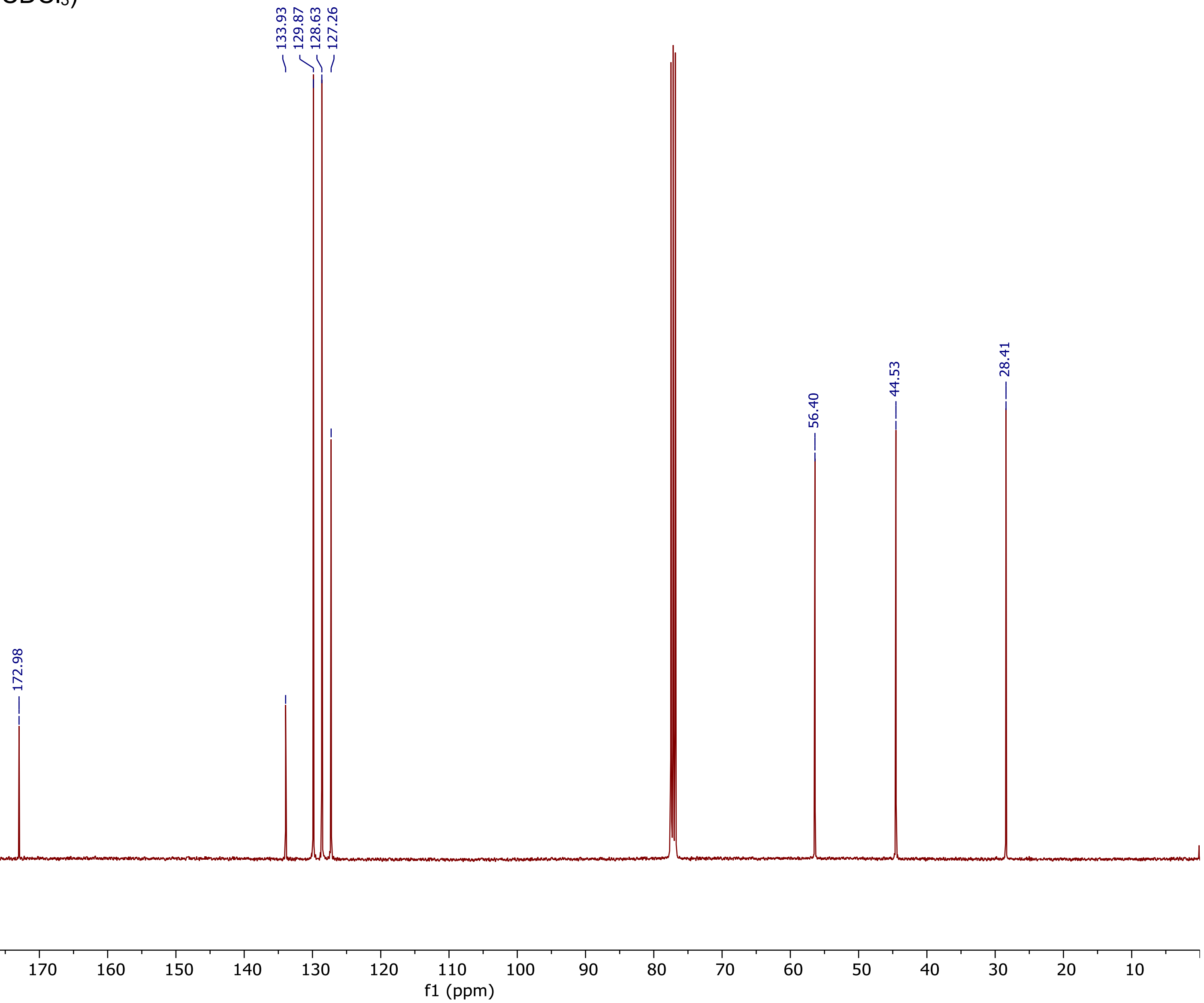

2D $^1H$ -$^1H$ COSY (400 MHz, $CDCl_3$)

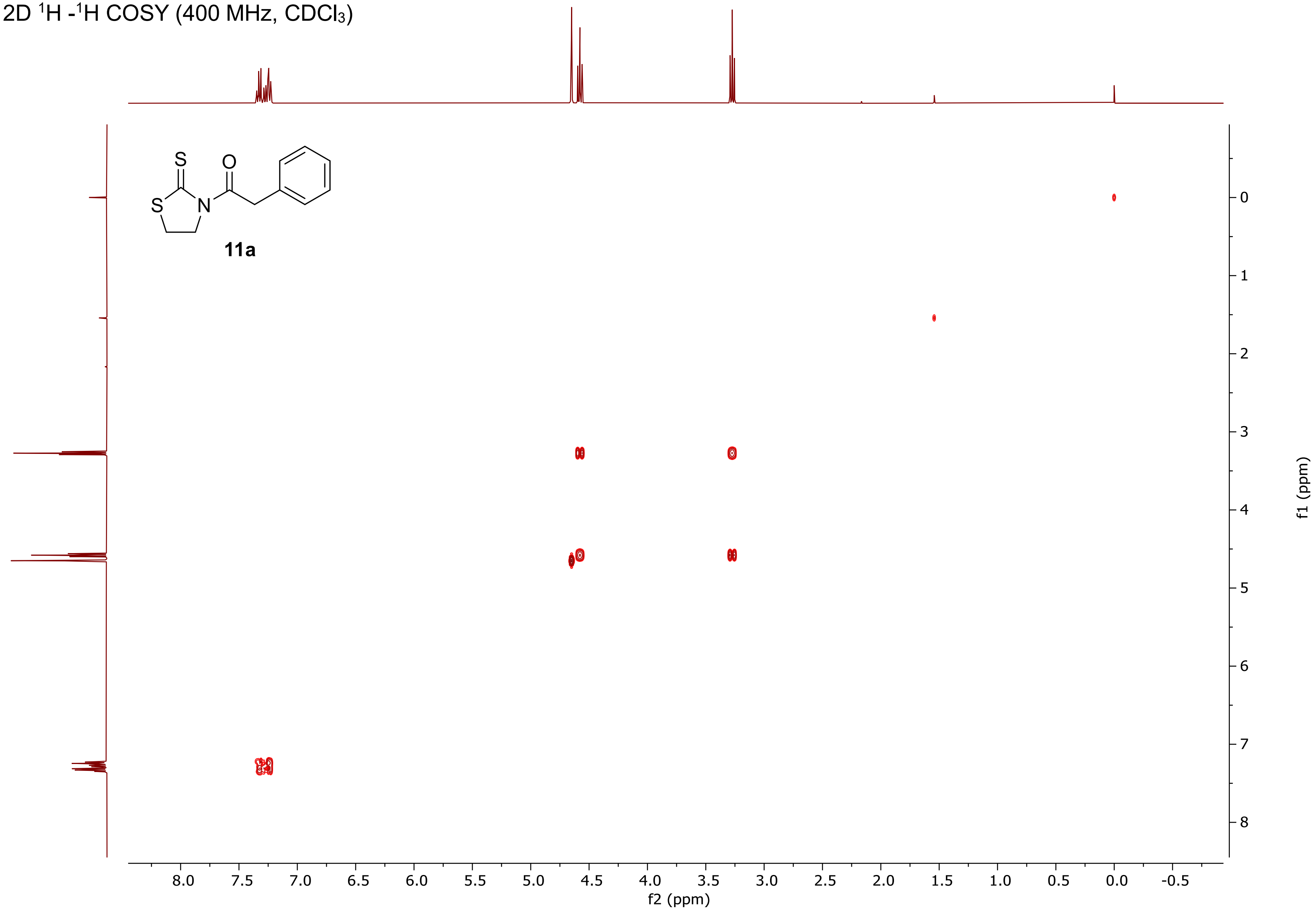

2D $^{1}H$ -$^{13}C$ HSQC (400 MHz, $CDCl_3$)

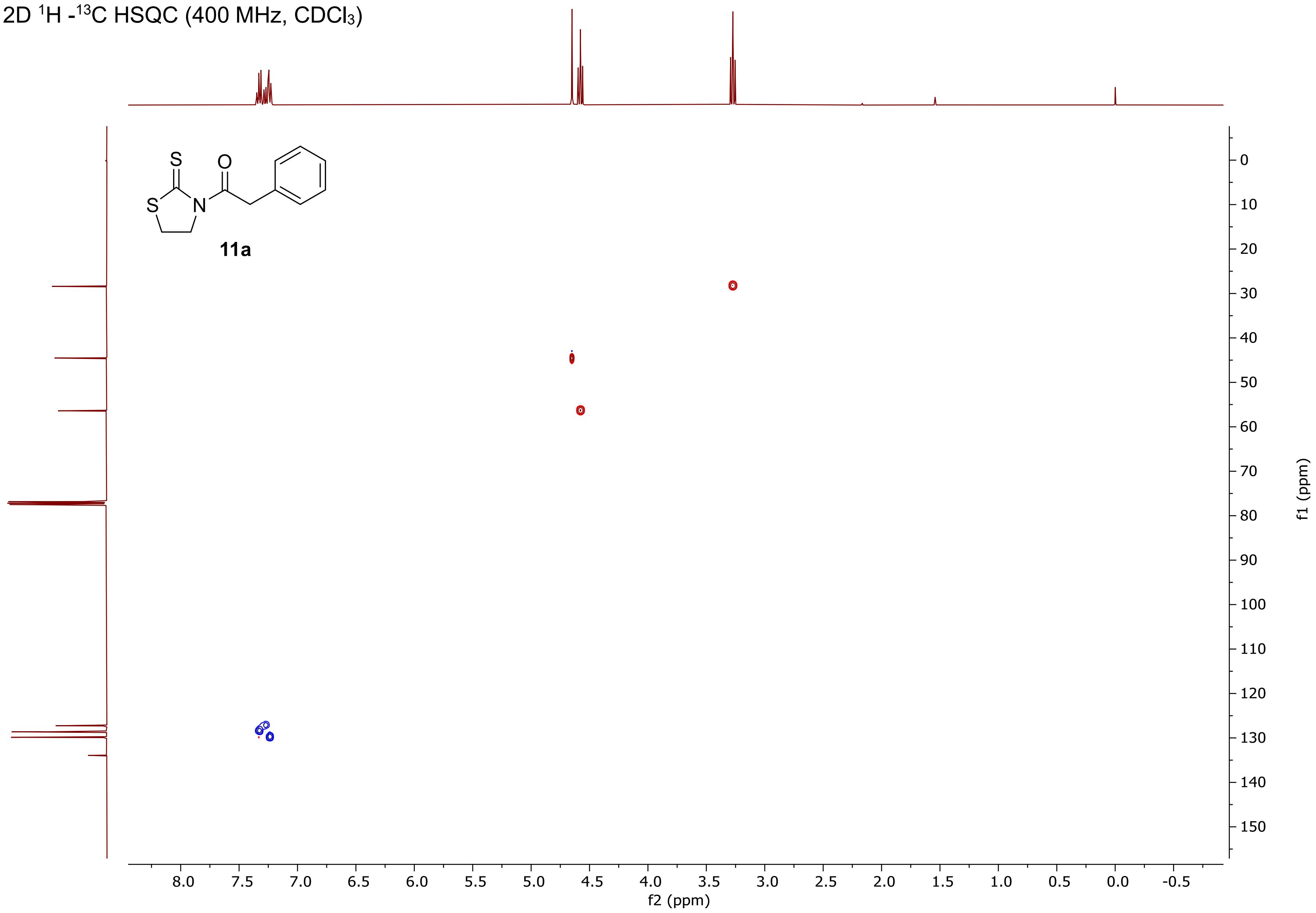

$^{1}H$ NMR (500 MHz, $CDCl_3$)

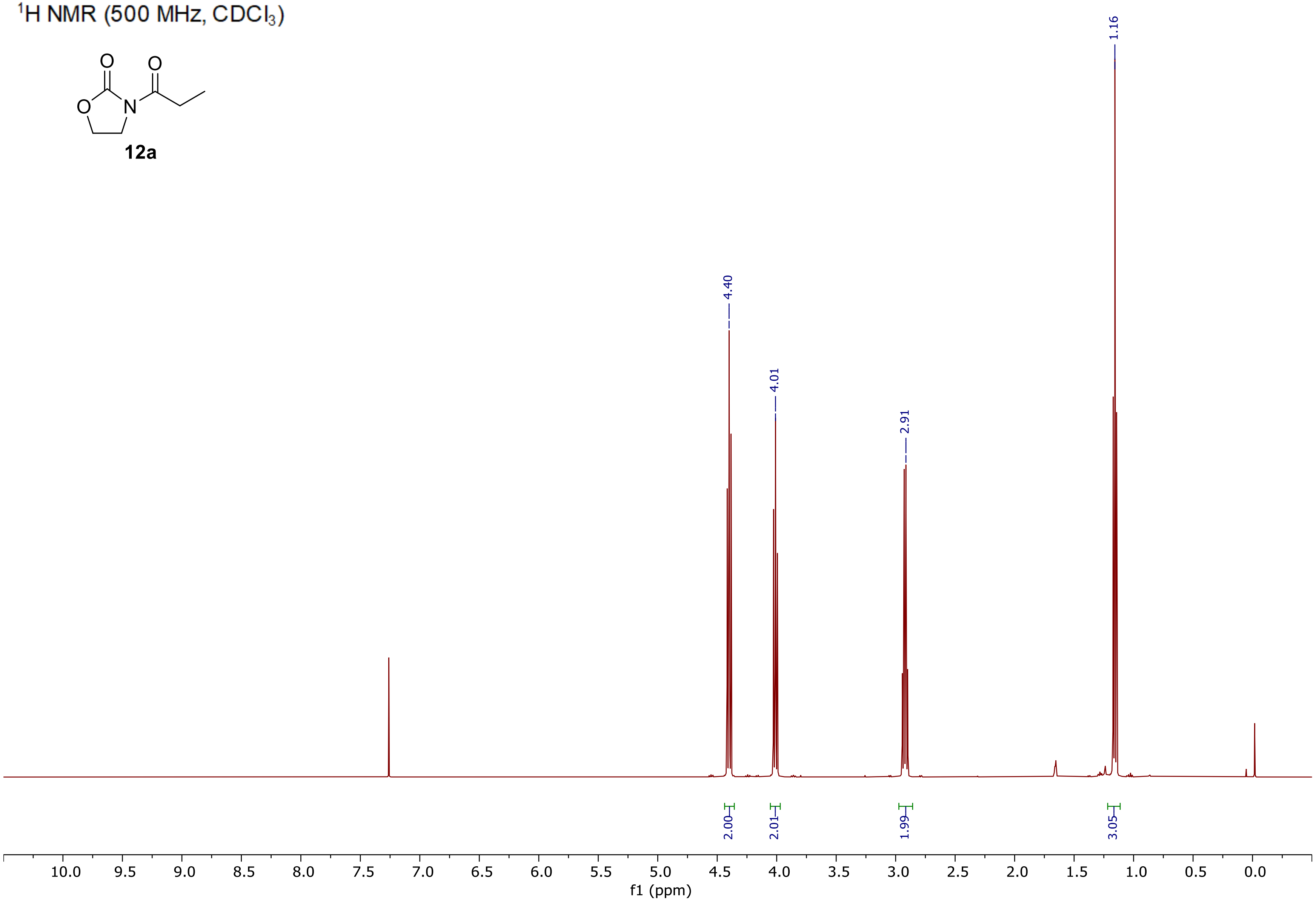

$^{13}C\{^{1}H\}$ NMR (126 MHz, $CDCl_3$)

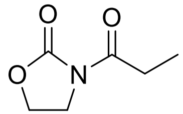


**12a**

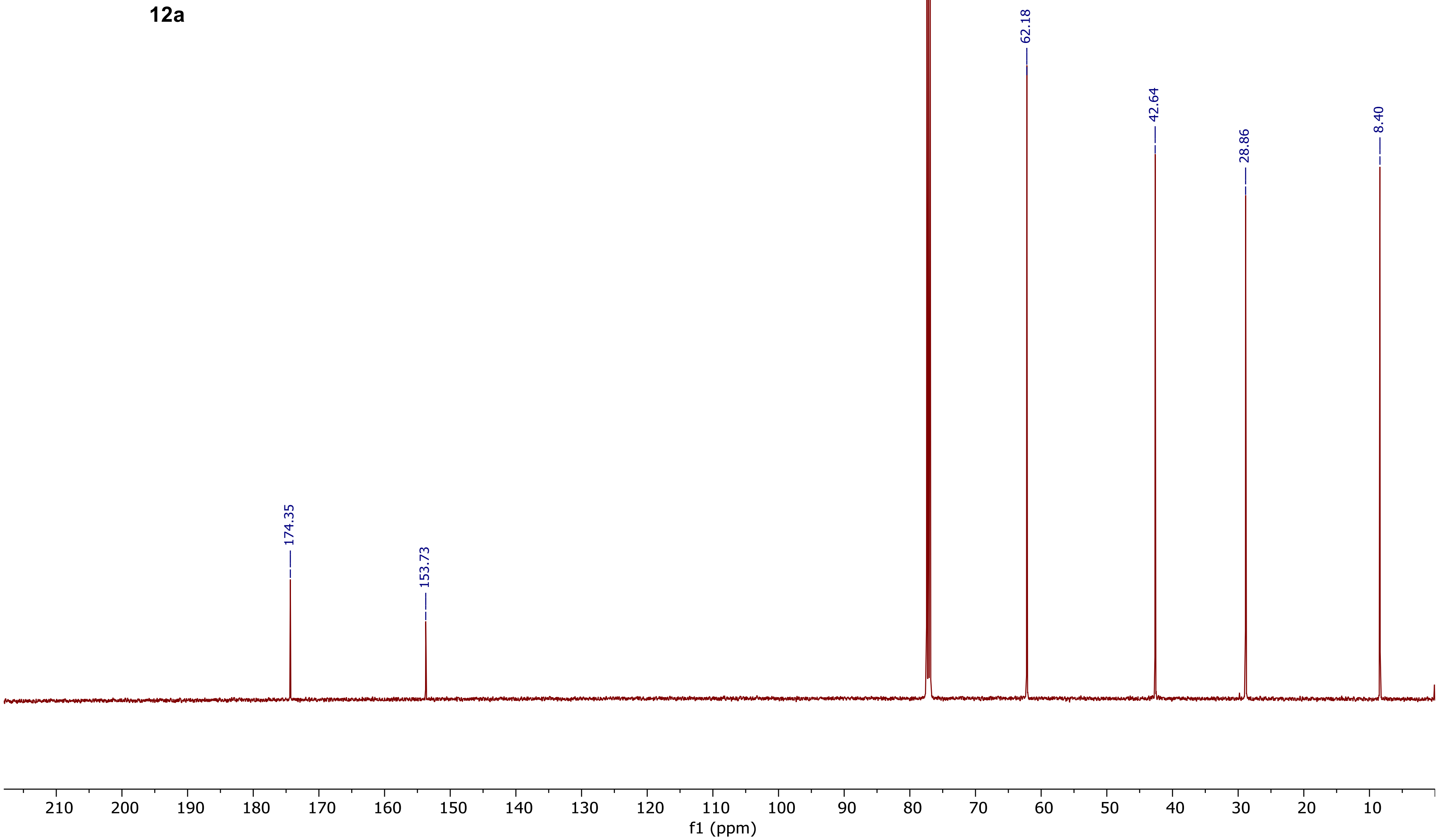

2D $^1H$ -$^1H$ COSY (500 MHz, $CDCl_3$)

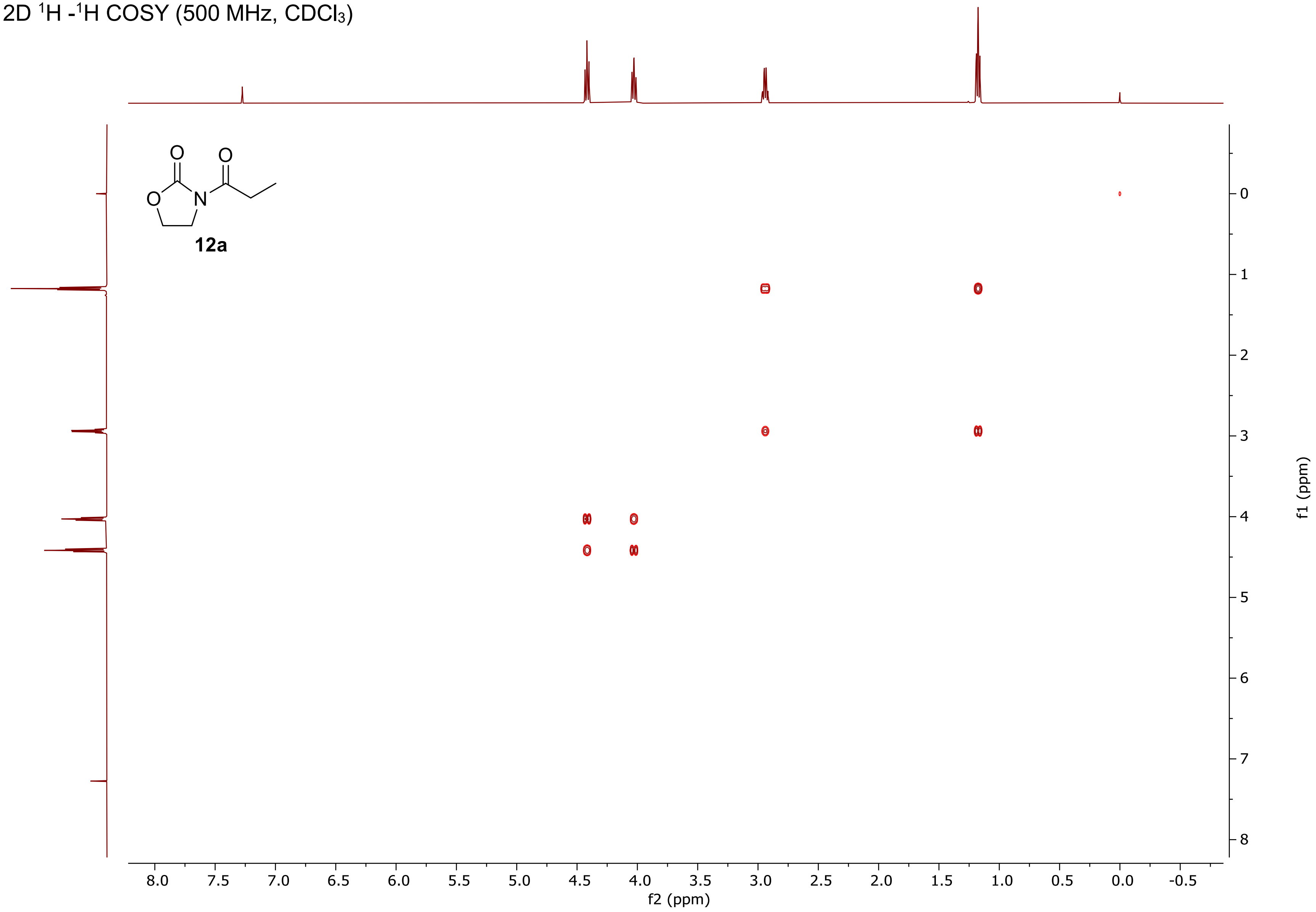

2D $^{1}H$ -$^{13}C$ HSQC (500 MHz, $CDCl_3$)

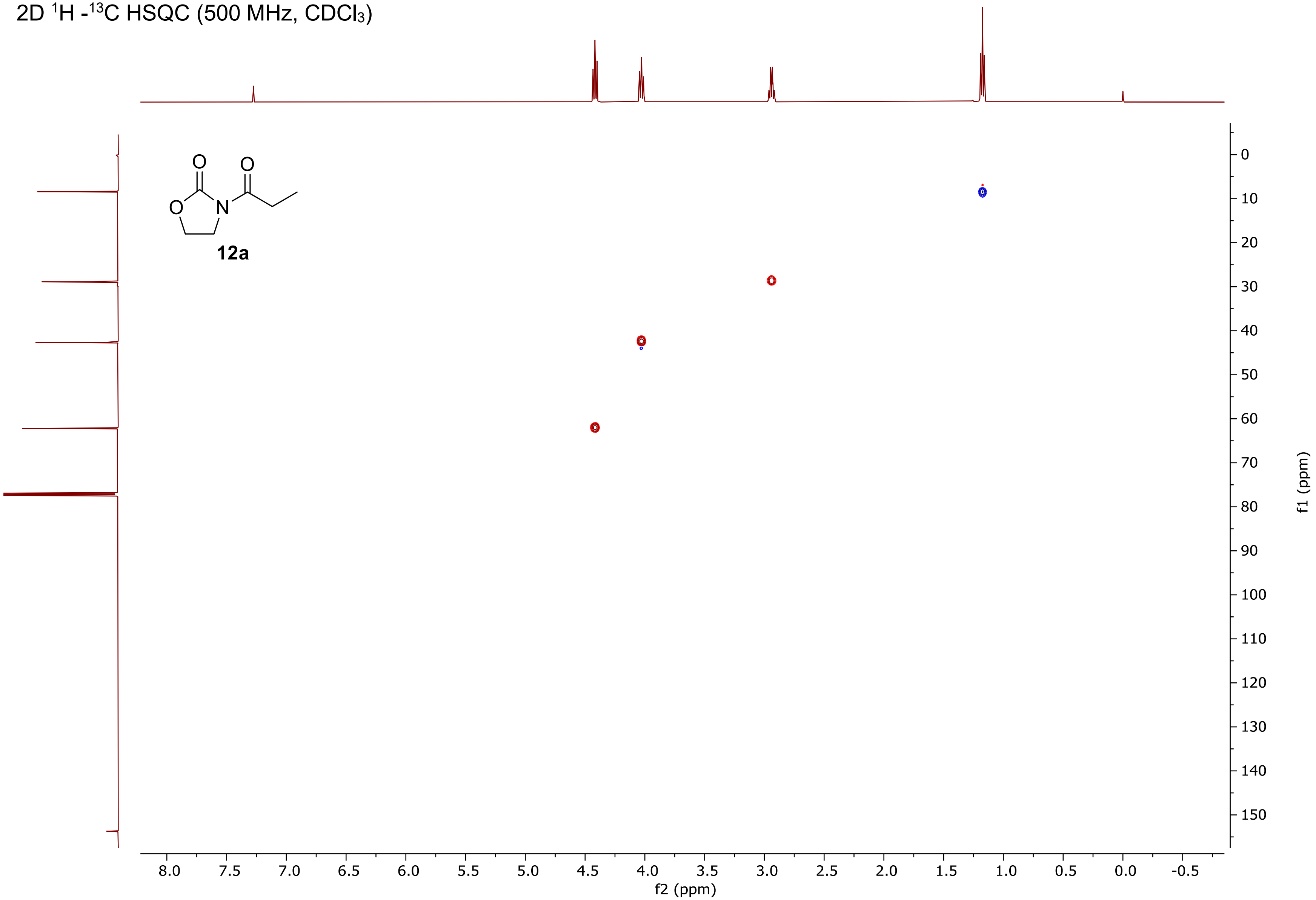

$^{1}H$ NMR (400 MHz, $CDCl_3$)

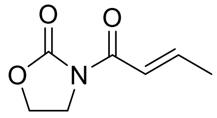

**13a**

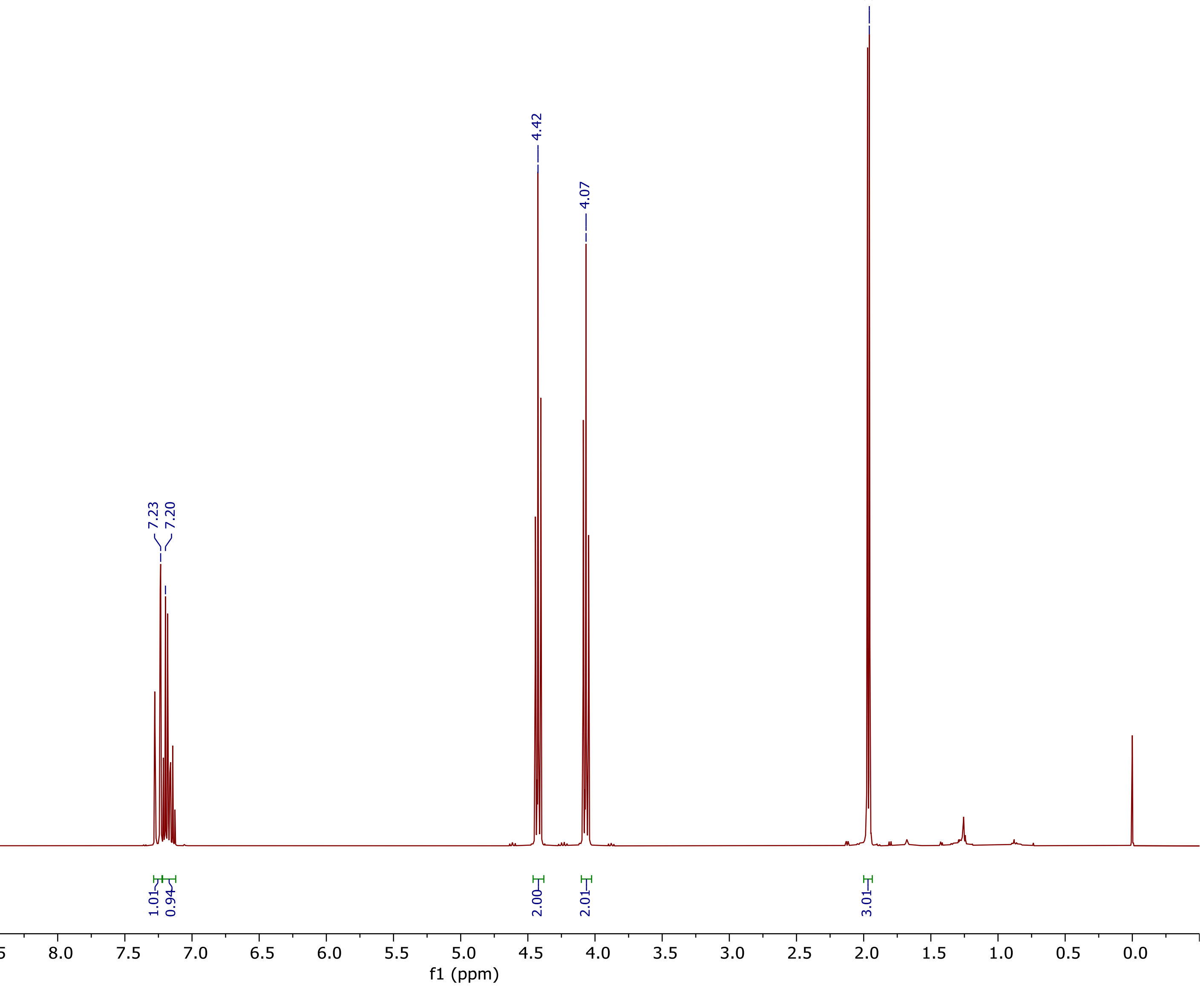

$^{13}C\{^{1}H\}$ NMR (101 MHz, $CDCl_3$)

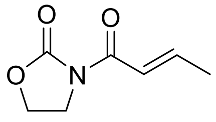

**13a**

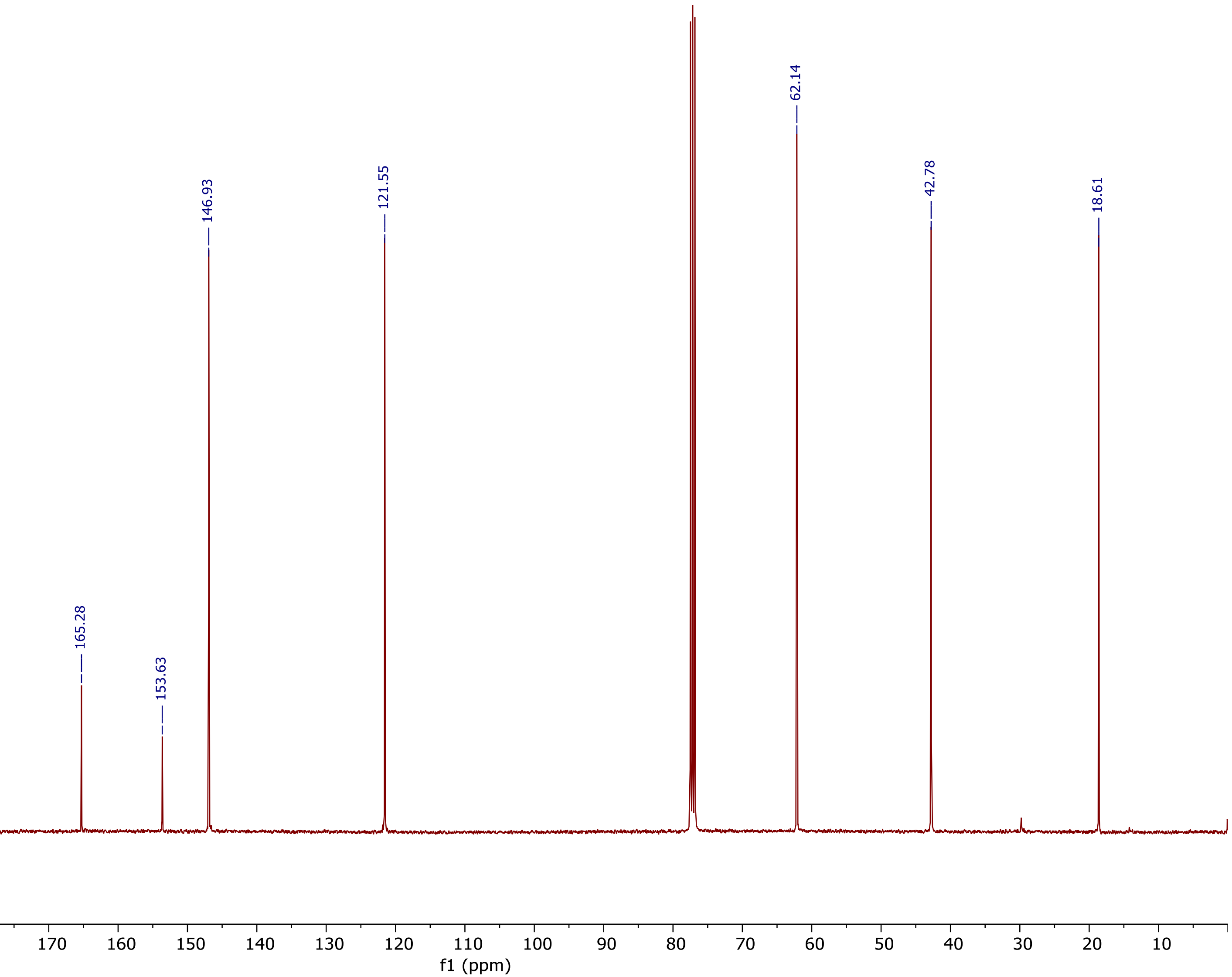

2D $^1$H -$^1$H COSY (400 MHz, $CDCl_3$)

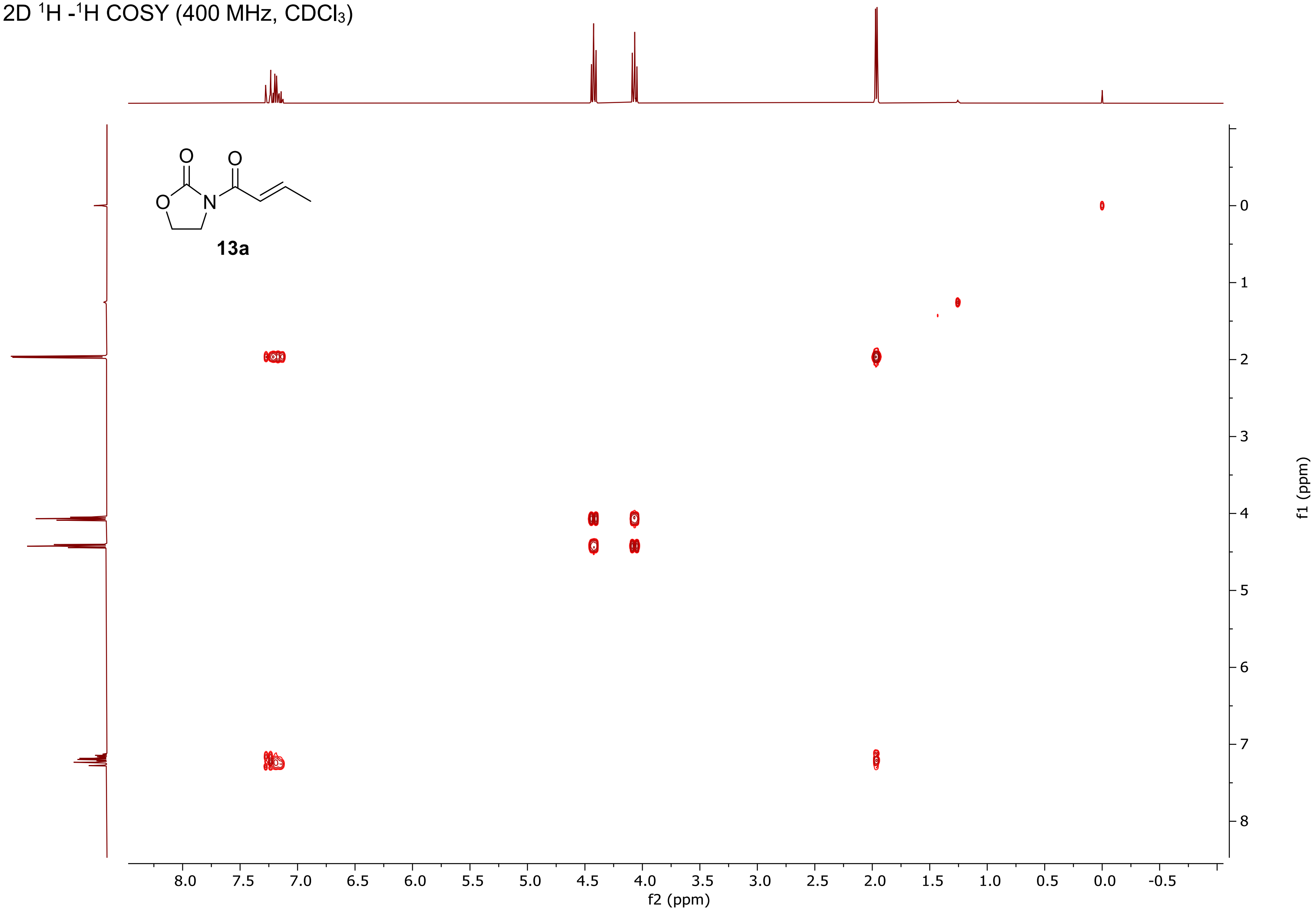

2D $^{1}$H -$^{13}$C HSQC (400 MHz, $CDCl_3$)

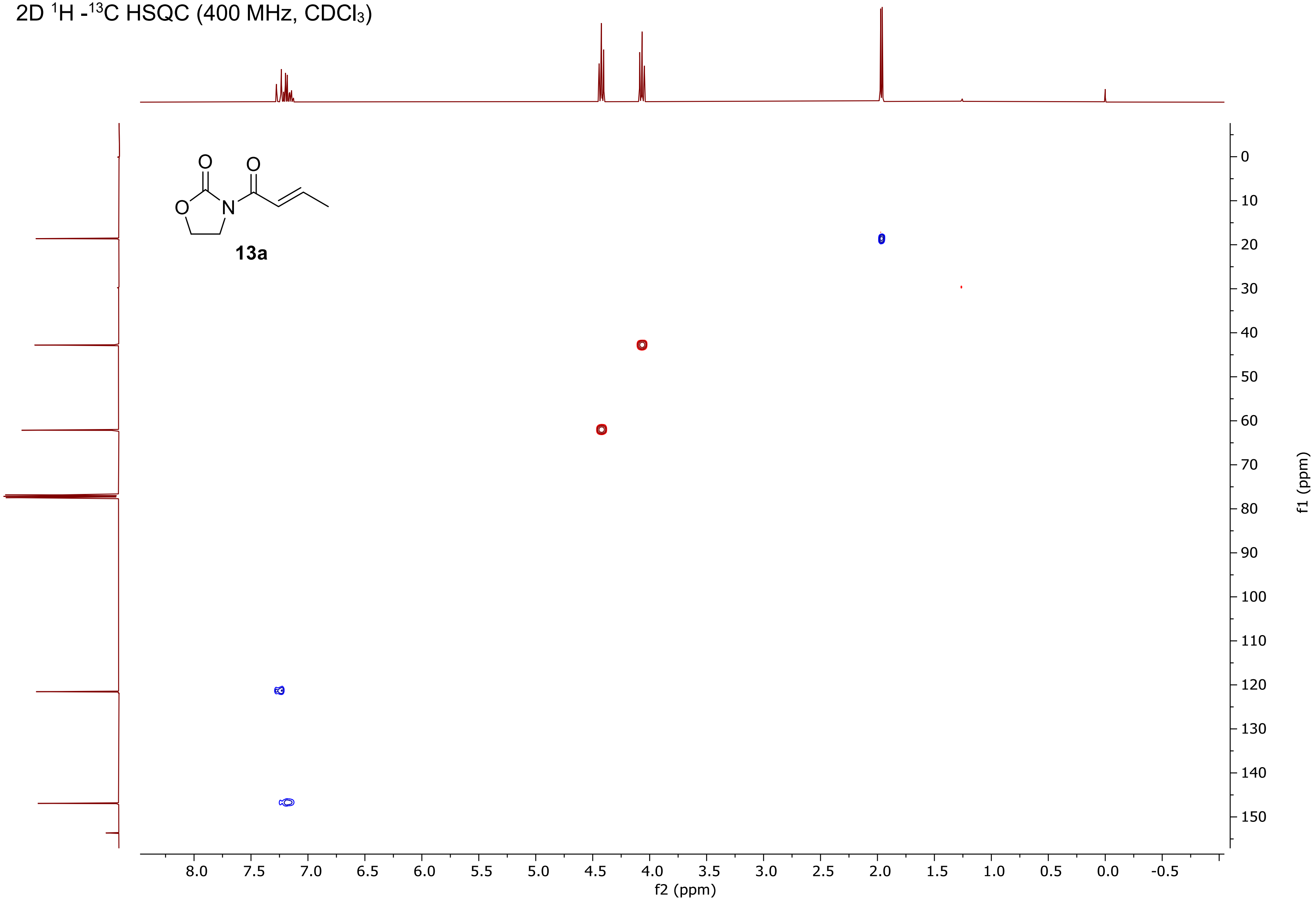

$^{1}H$ NMR (500 MHz, $CDCl_3$)

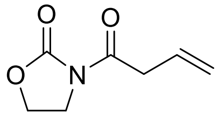

**14a**

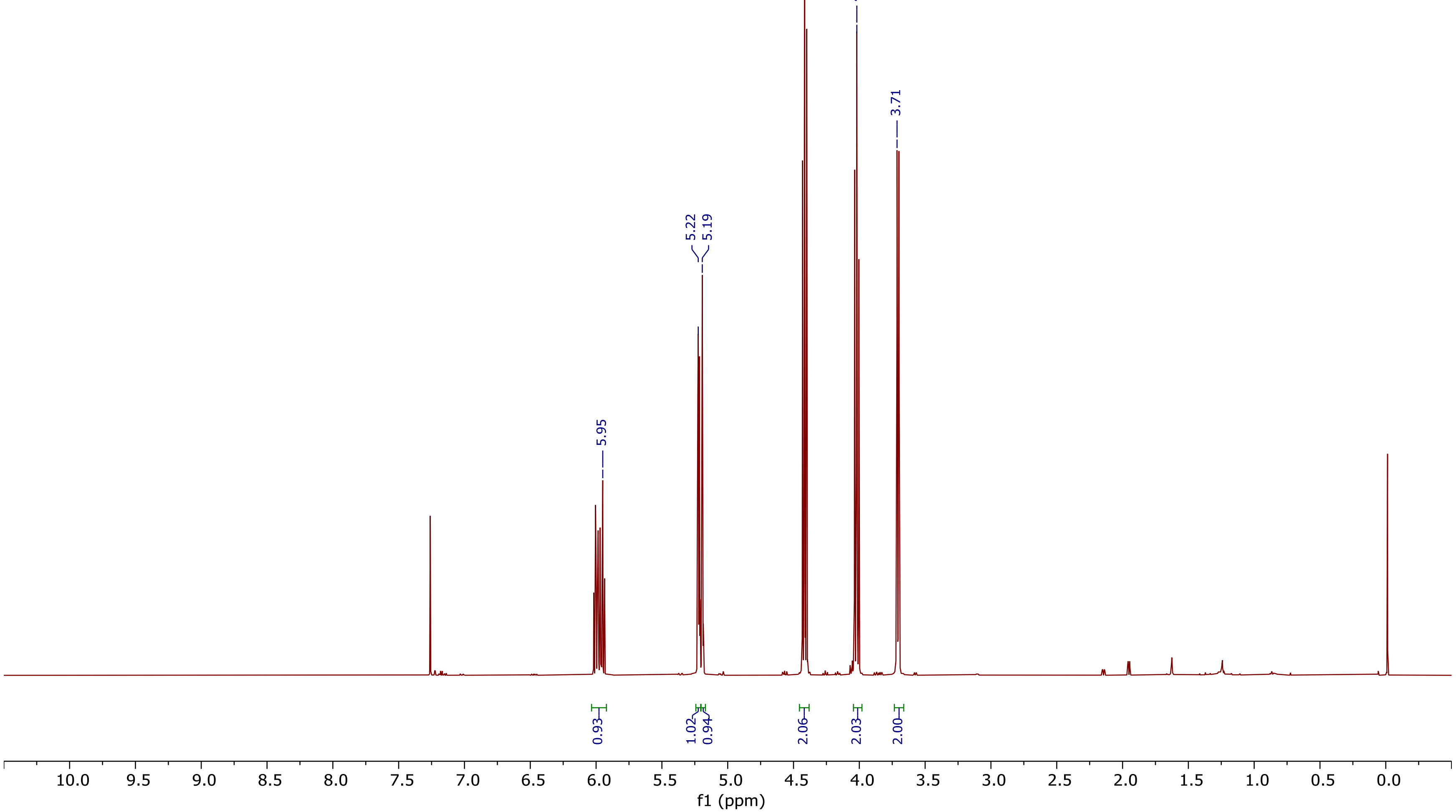

$^{13}C\{^{1}H\}$ NMR (126 MHz, $CDCl_3$)

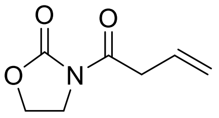

**14a**

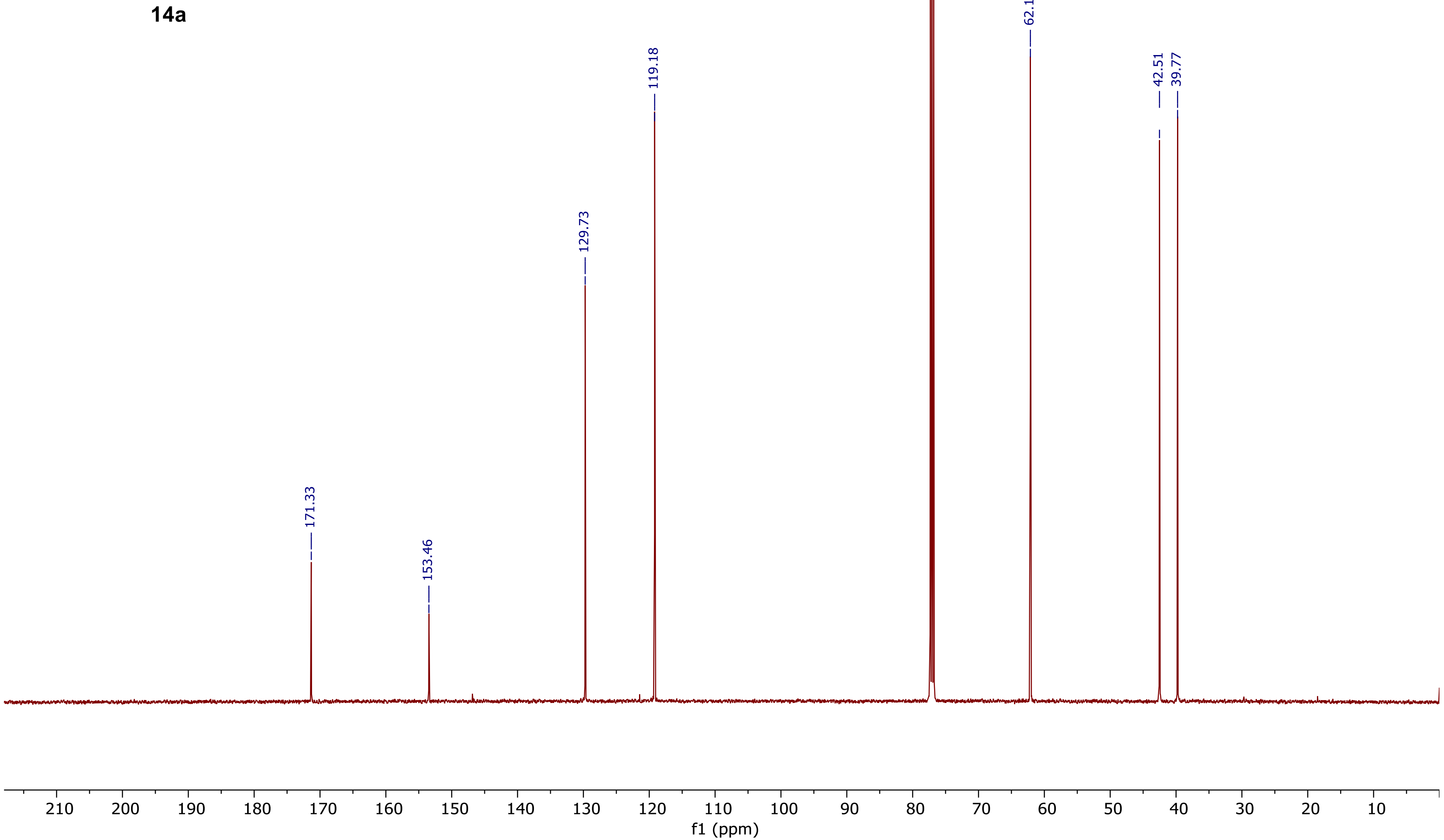

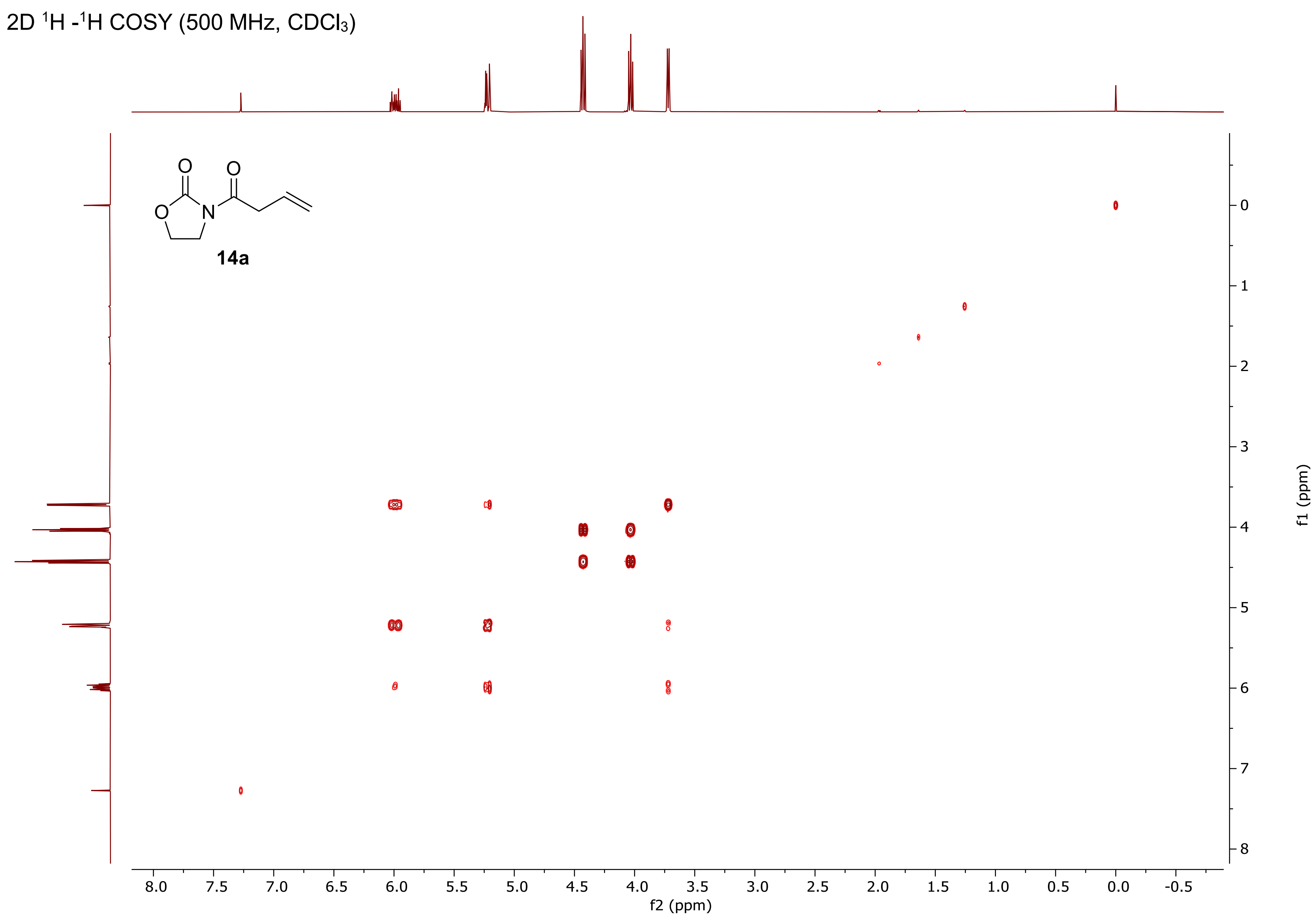
2D ¹H -¹H COSY (500 MHz, CDCl₃)
14a
f2 (ppm)
f1 (ppm)

2D $^{1}H$ -$^{13}C$ HSQC (500 MHz, $CDCl_3$)

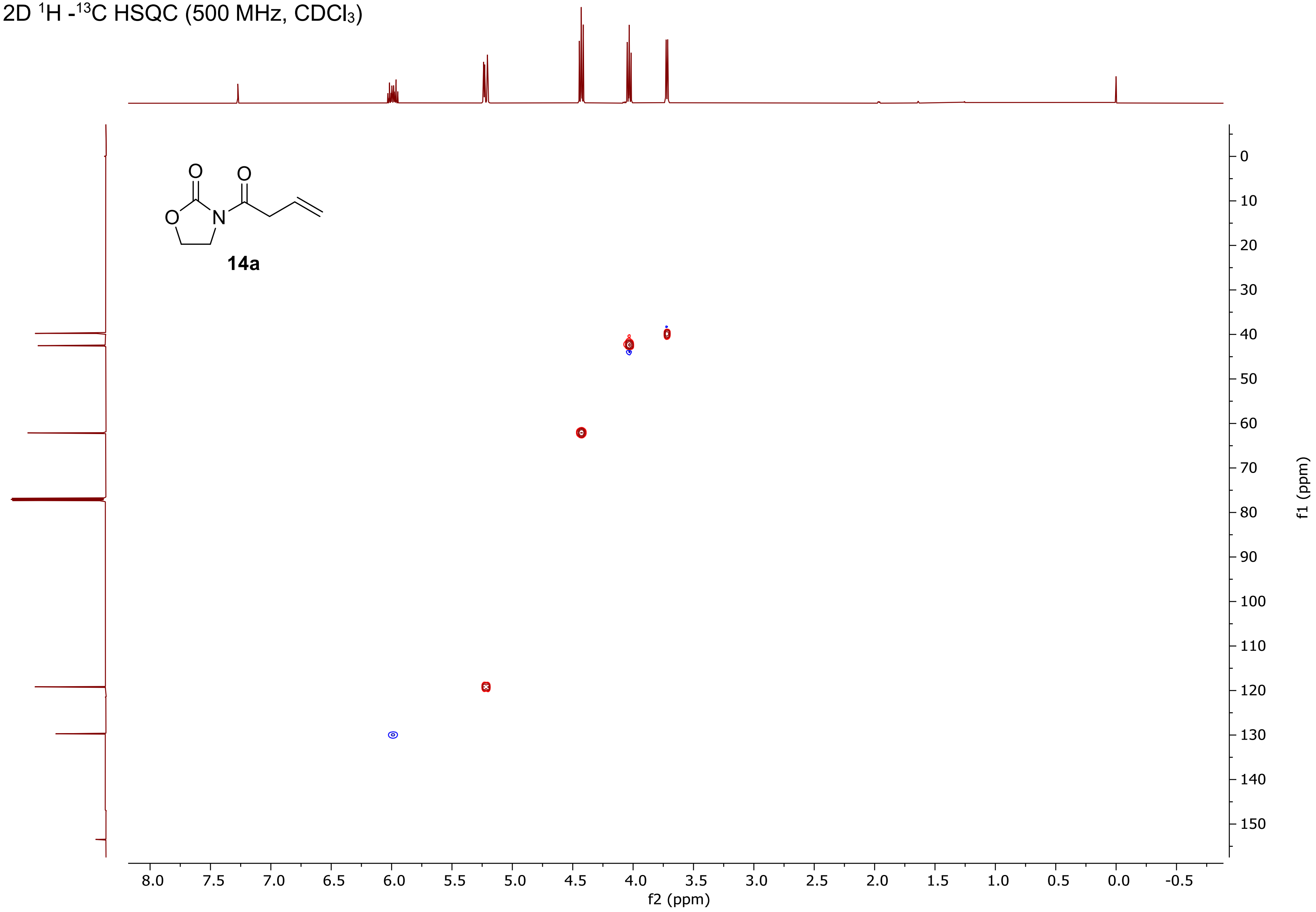

$^1H$ NMR (500 MHz, $CDCl_3$)

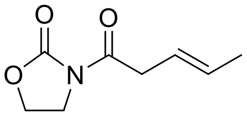

**14b**

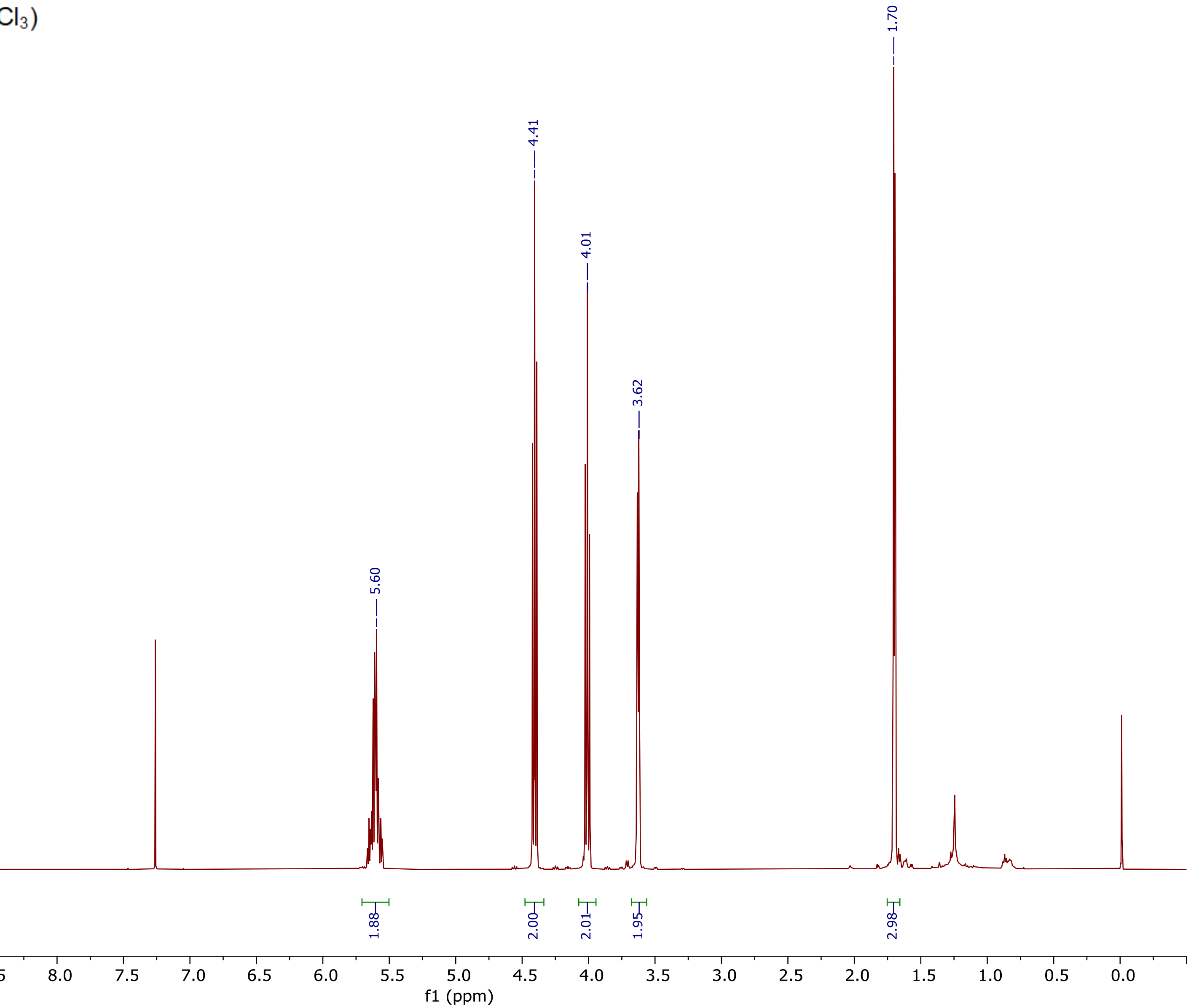

$^{13}C\{^{1}H\}$ NMR (126 MHz, $CDCl_3$)

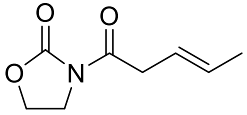

**14b**

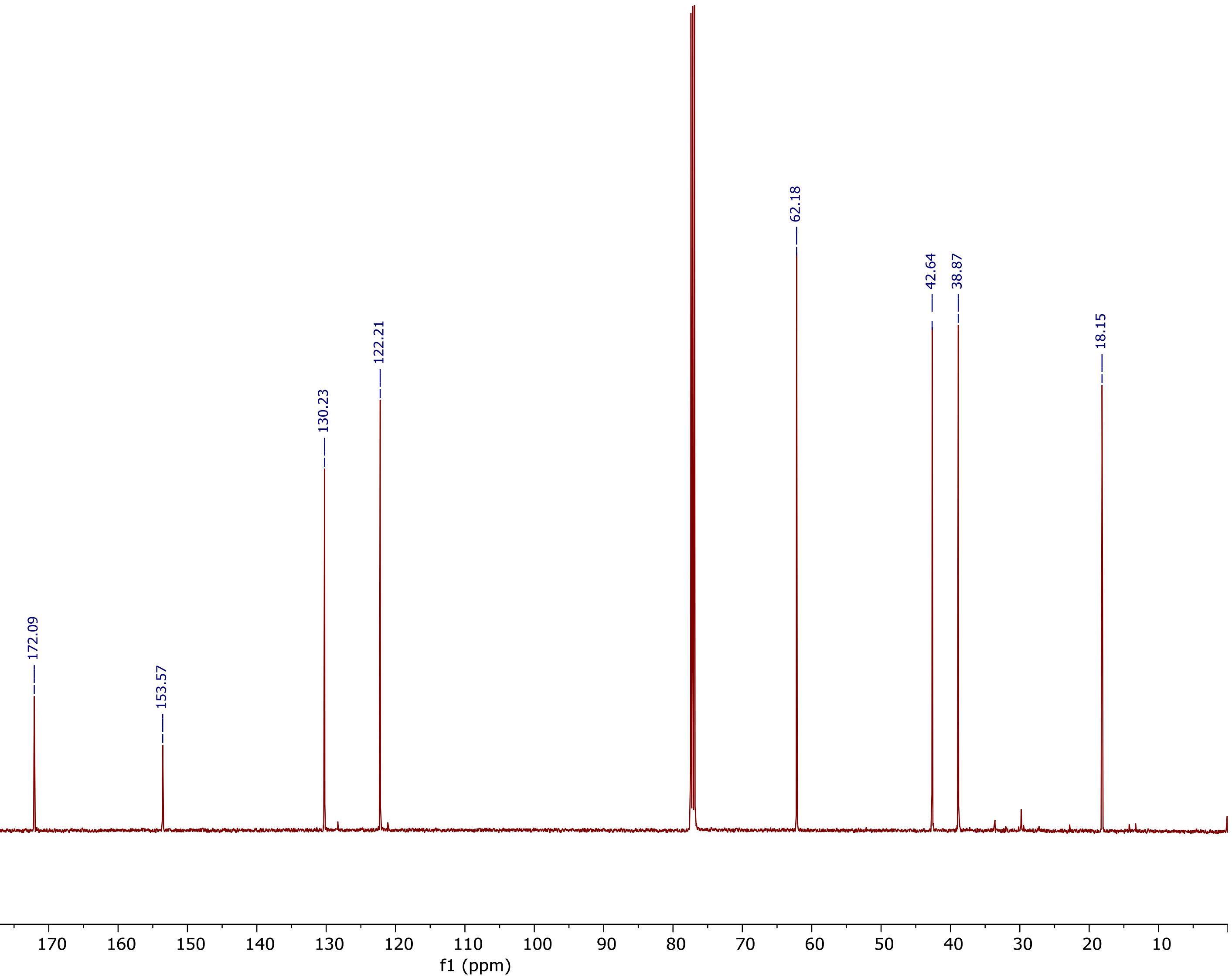

2D $^1H$ -$^1H$ COSY (500 MHz, $CDCl_3$)

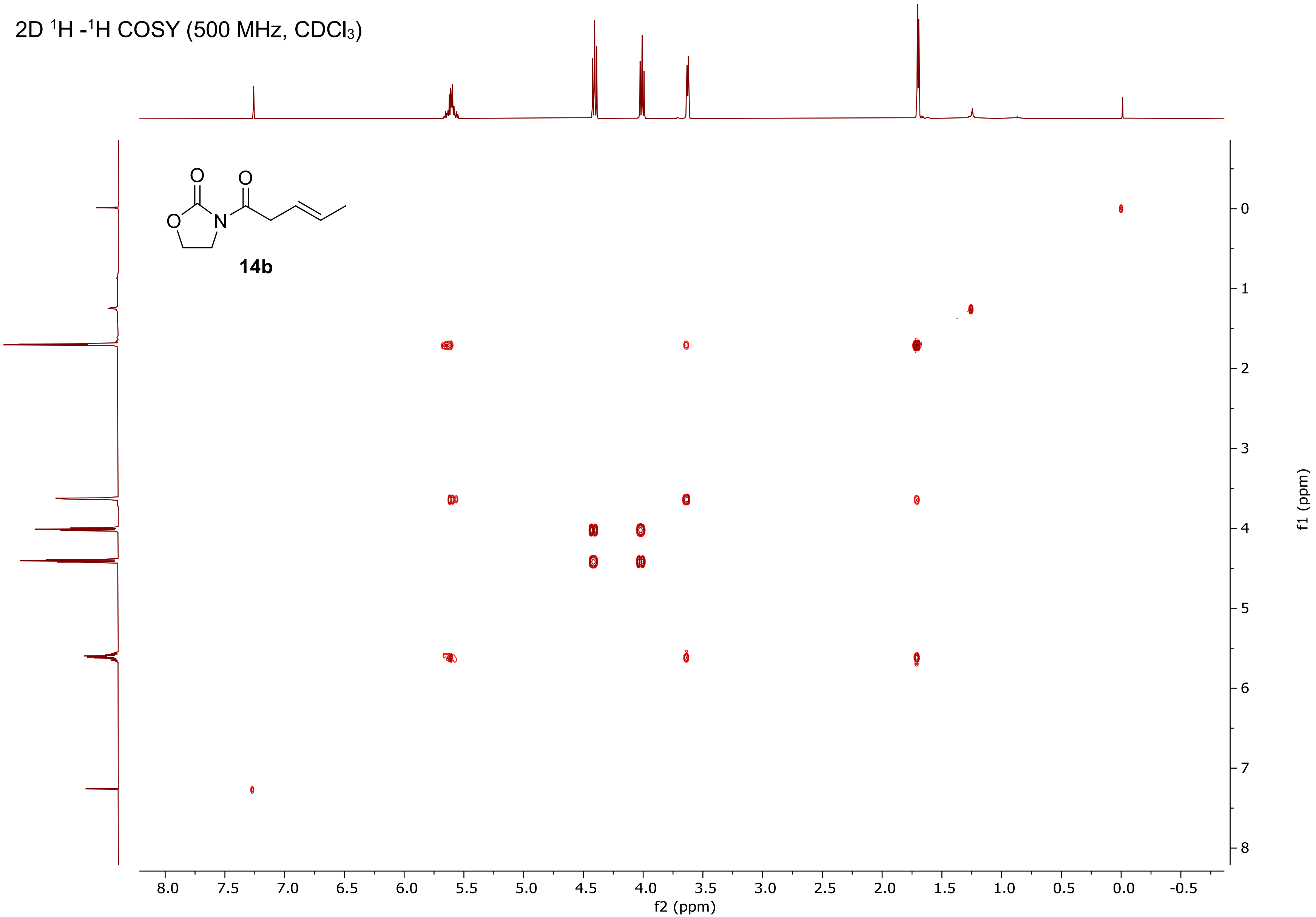

2D $^{1}H$ -$^{13}C$ HSQC (500 MHz, $CDCl_3$)

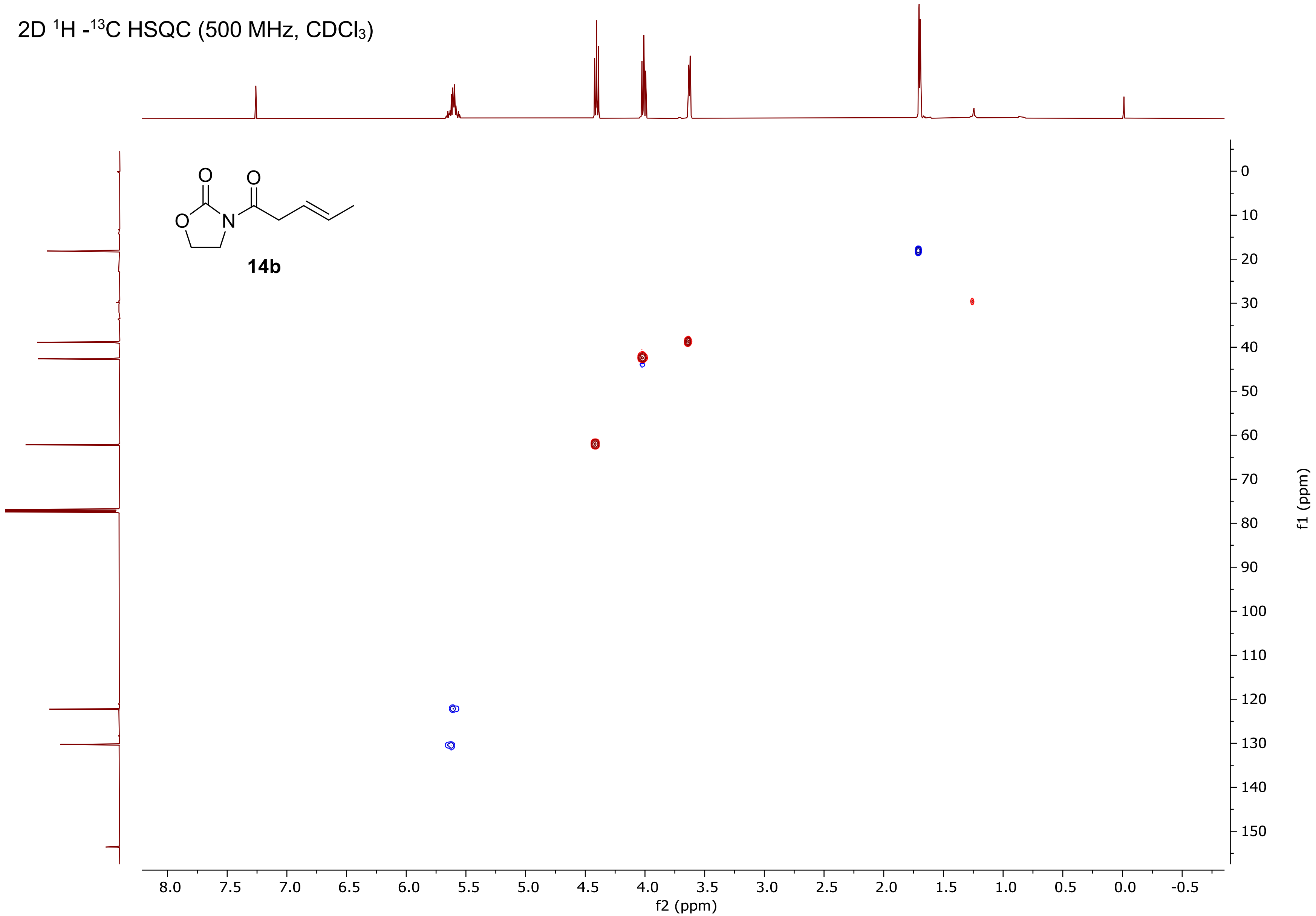

$^{1}$H NMR (500 MHz, CDCl$_3$)

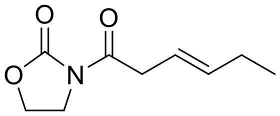

**14c**

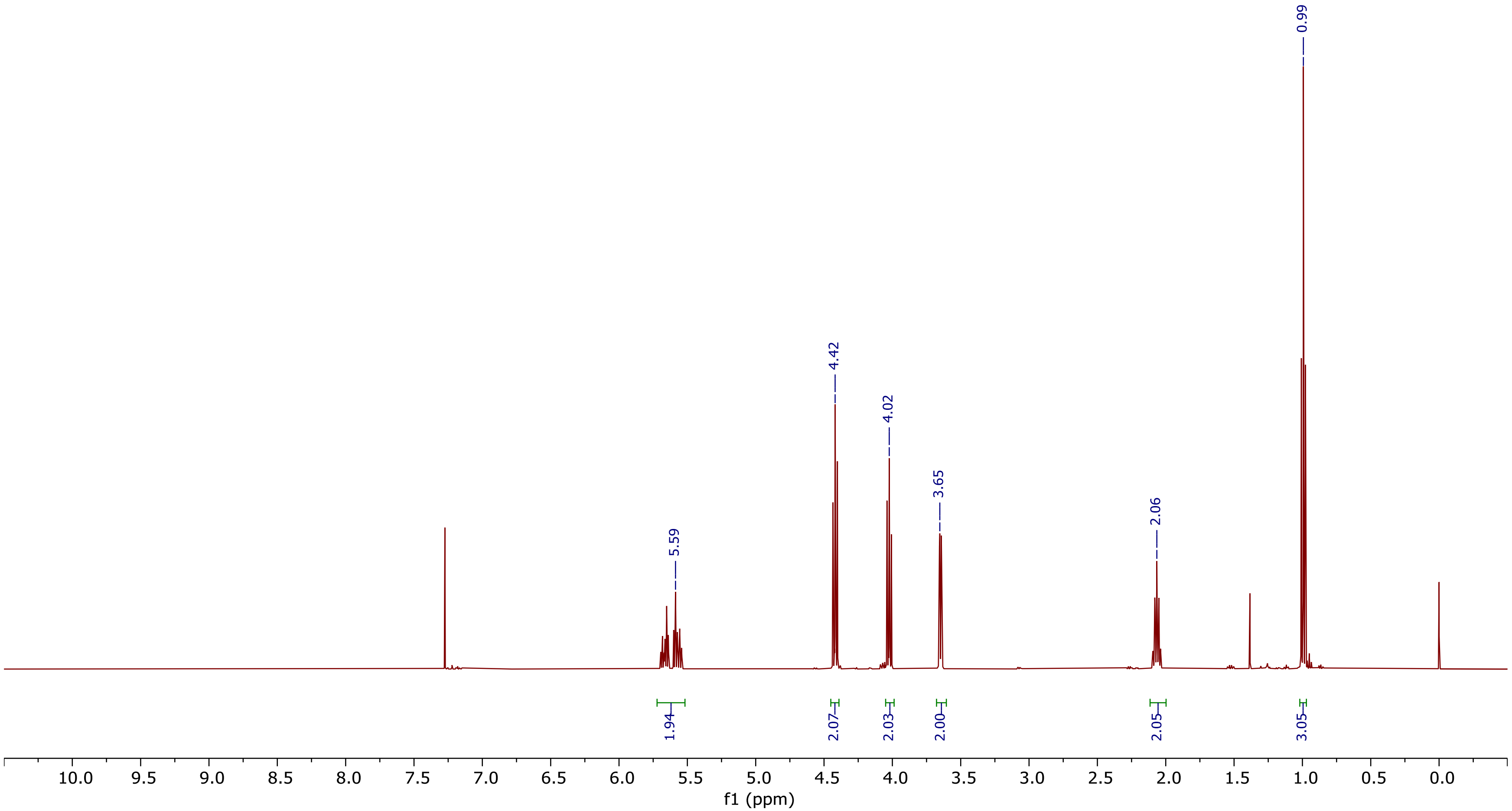

$^{13}C\{^{1}H\}$ NMR (126 MHz, $CDCl_3$)

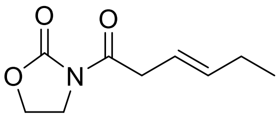

**14c**

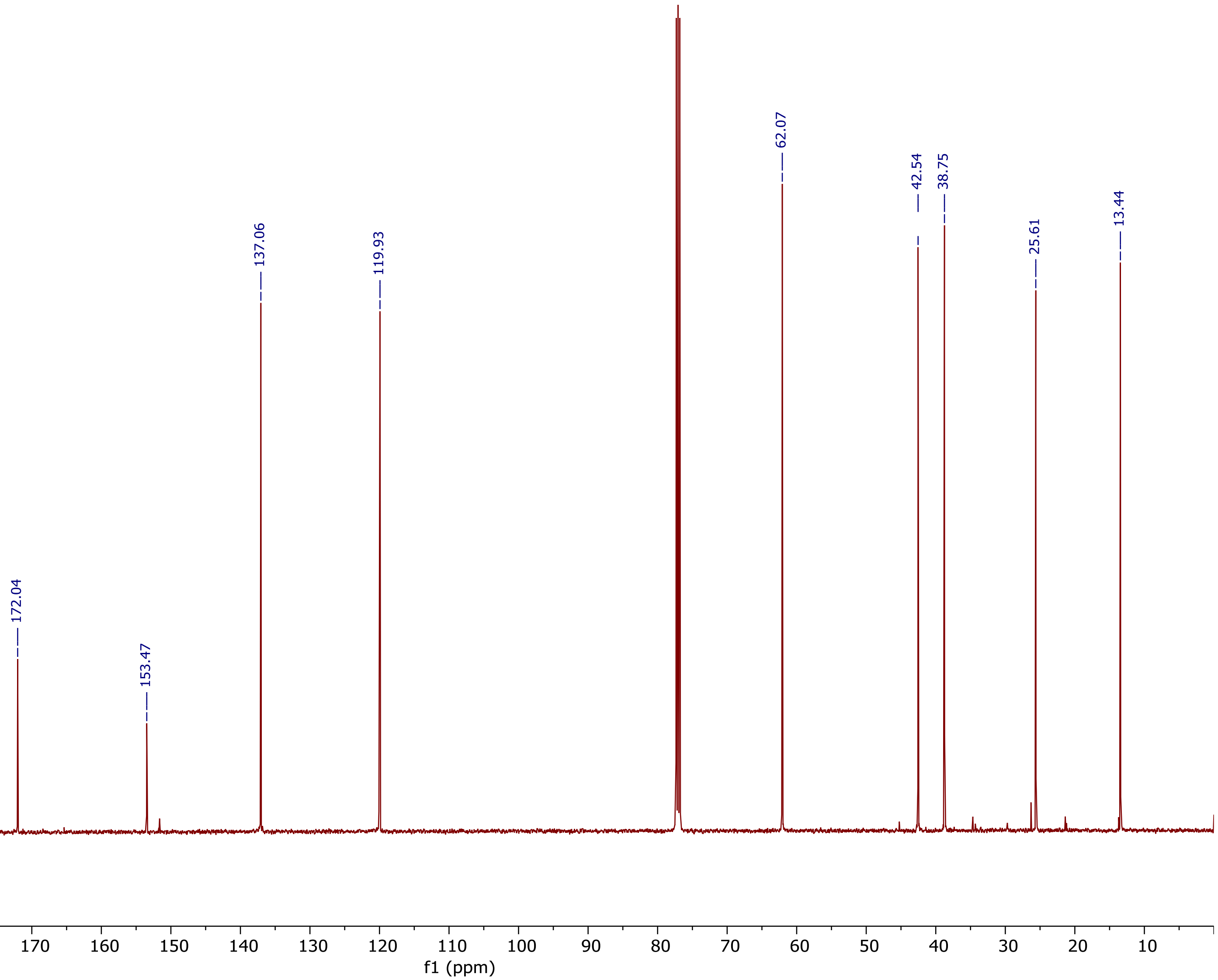

2D $^1H$ -$^1H$ COSY (500 MHz, $CDCl_3$)

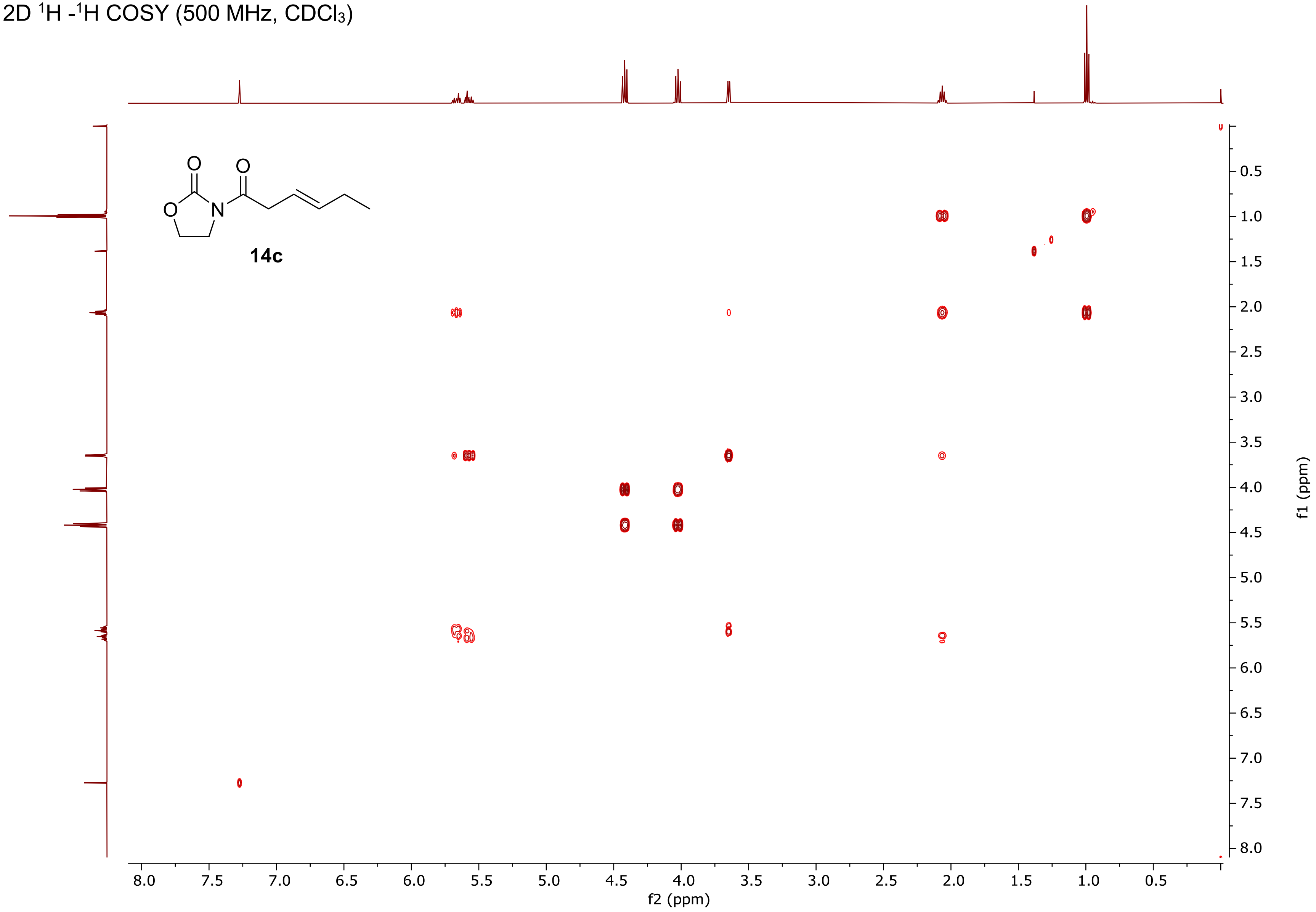

2D $^{1}$H -$^{13}$C HSQC (500 MHz, $CDCl_3$)

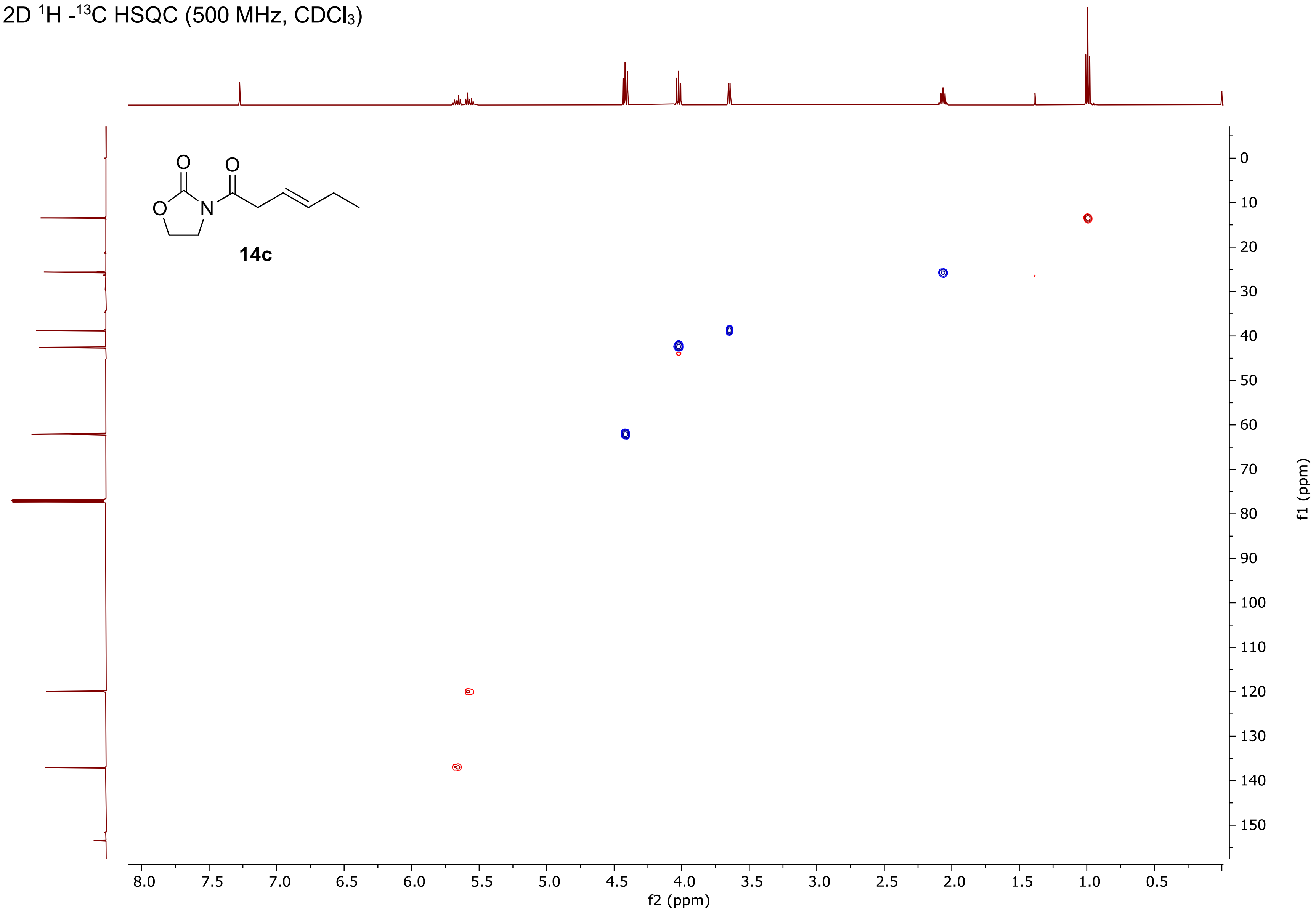

[1]H NMR (500 MHz, $CDCl_3$)

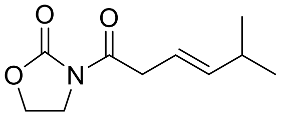

**14d**

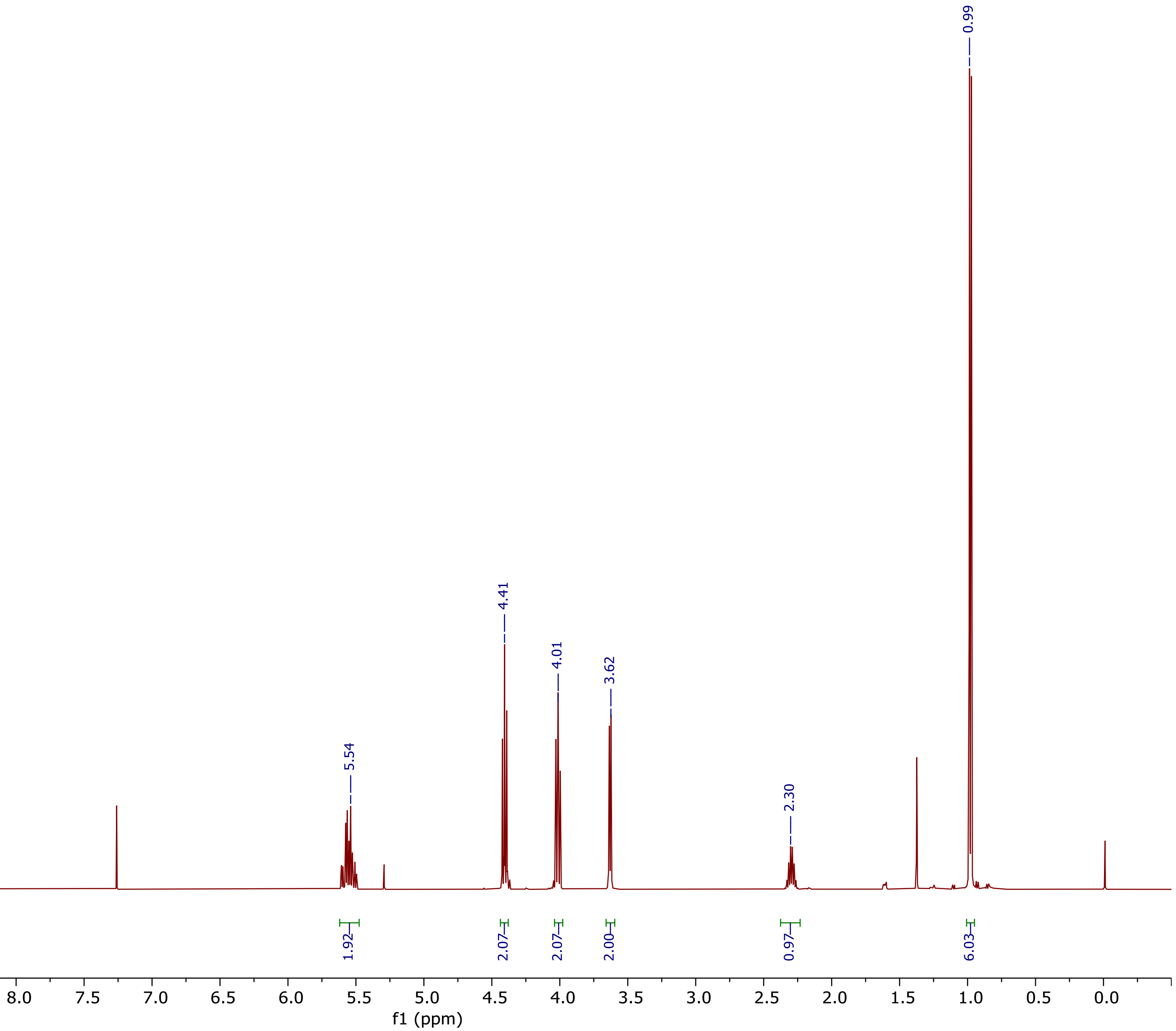

$^{13}C\{^{1}H\}$ NMR (126 MHz, $CDCl_3$)

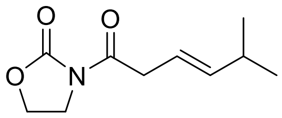

**14d**

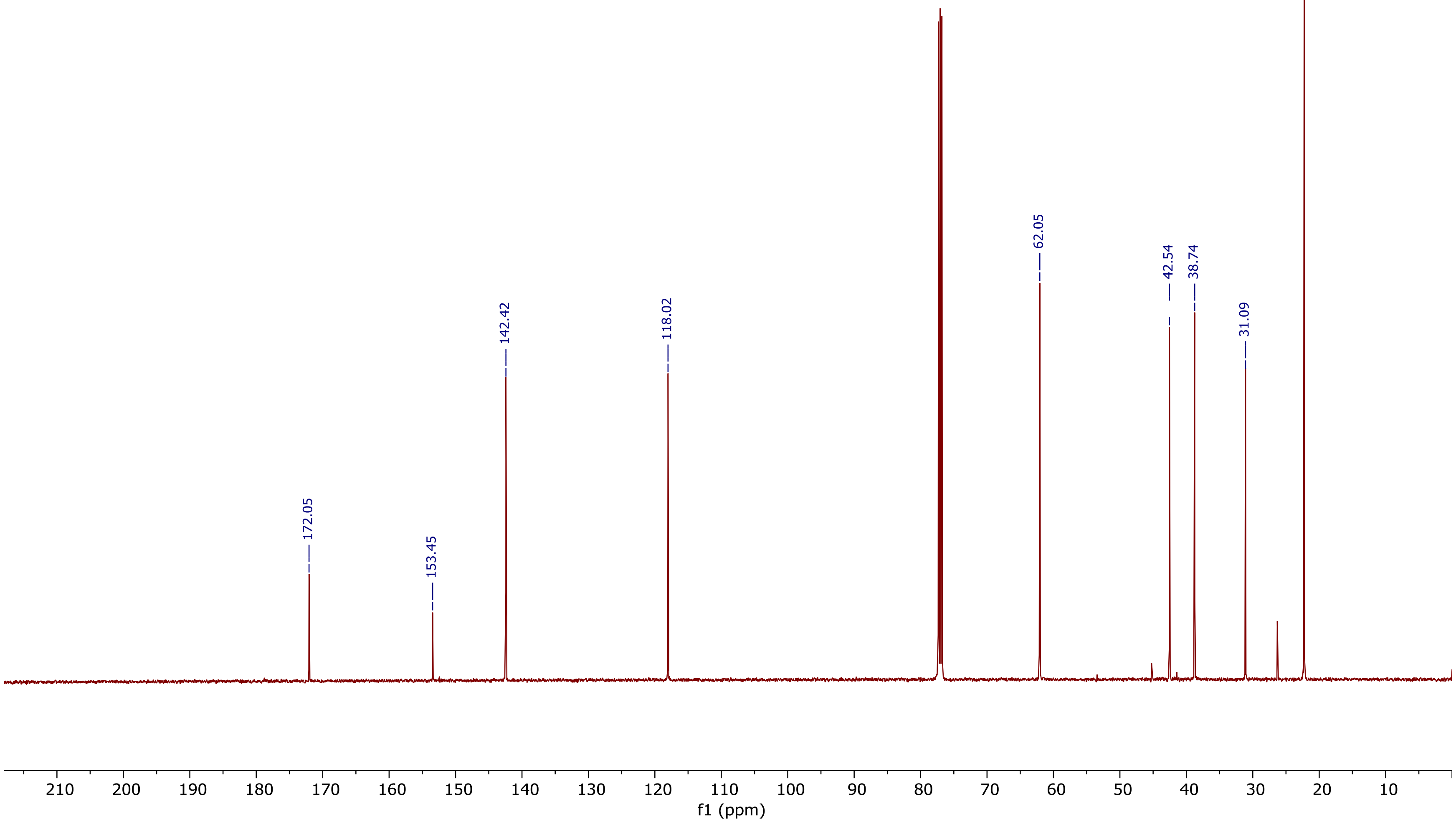

2D $^{1}H$ -$^{1}H$ COSY (500 MHz, $CDCl_3$)

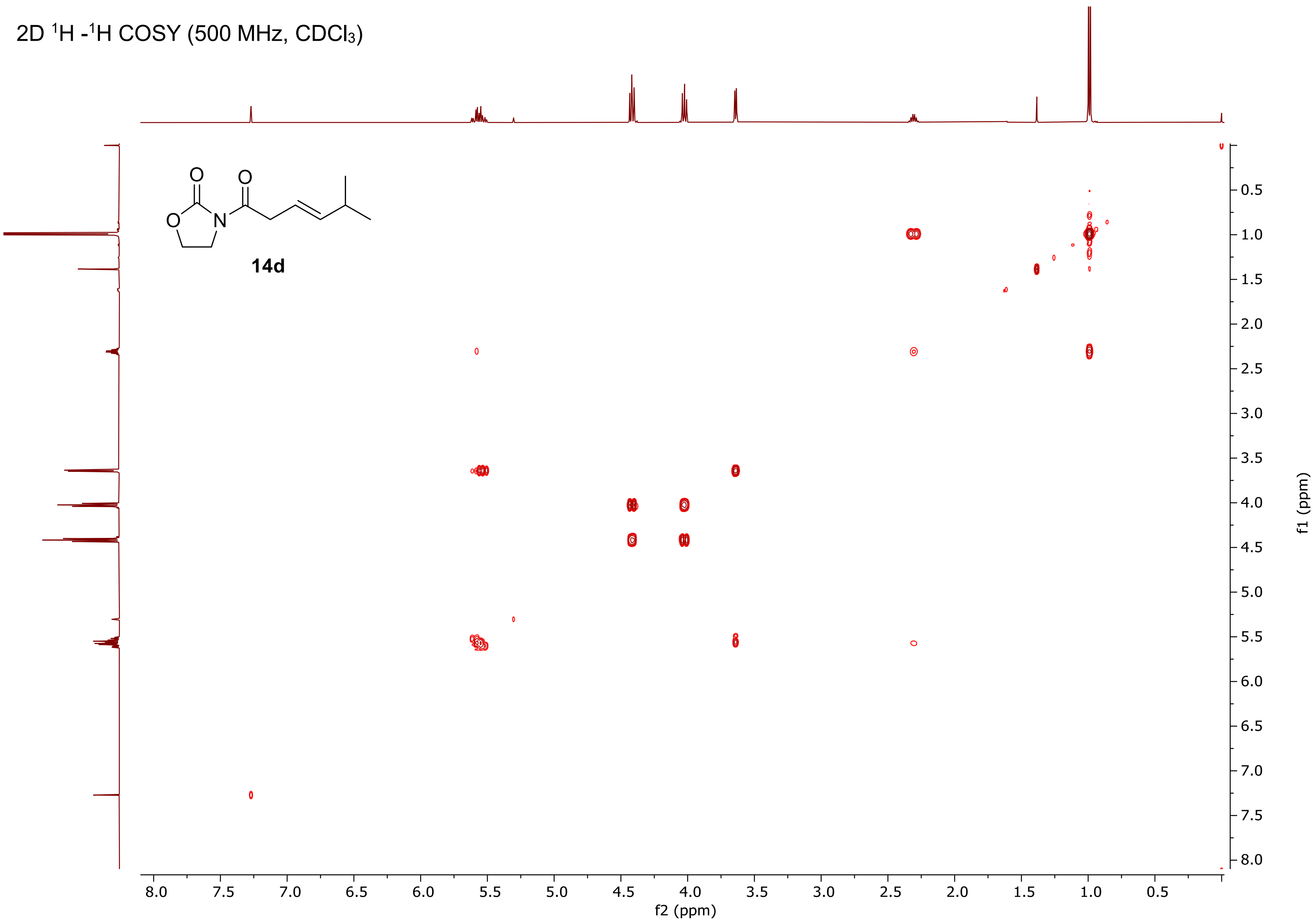

2D $^{1}H$ -$^{13}C$ HSQC (500 MHz, $CDCl_3$)

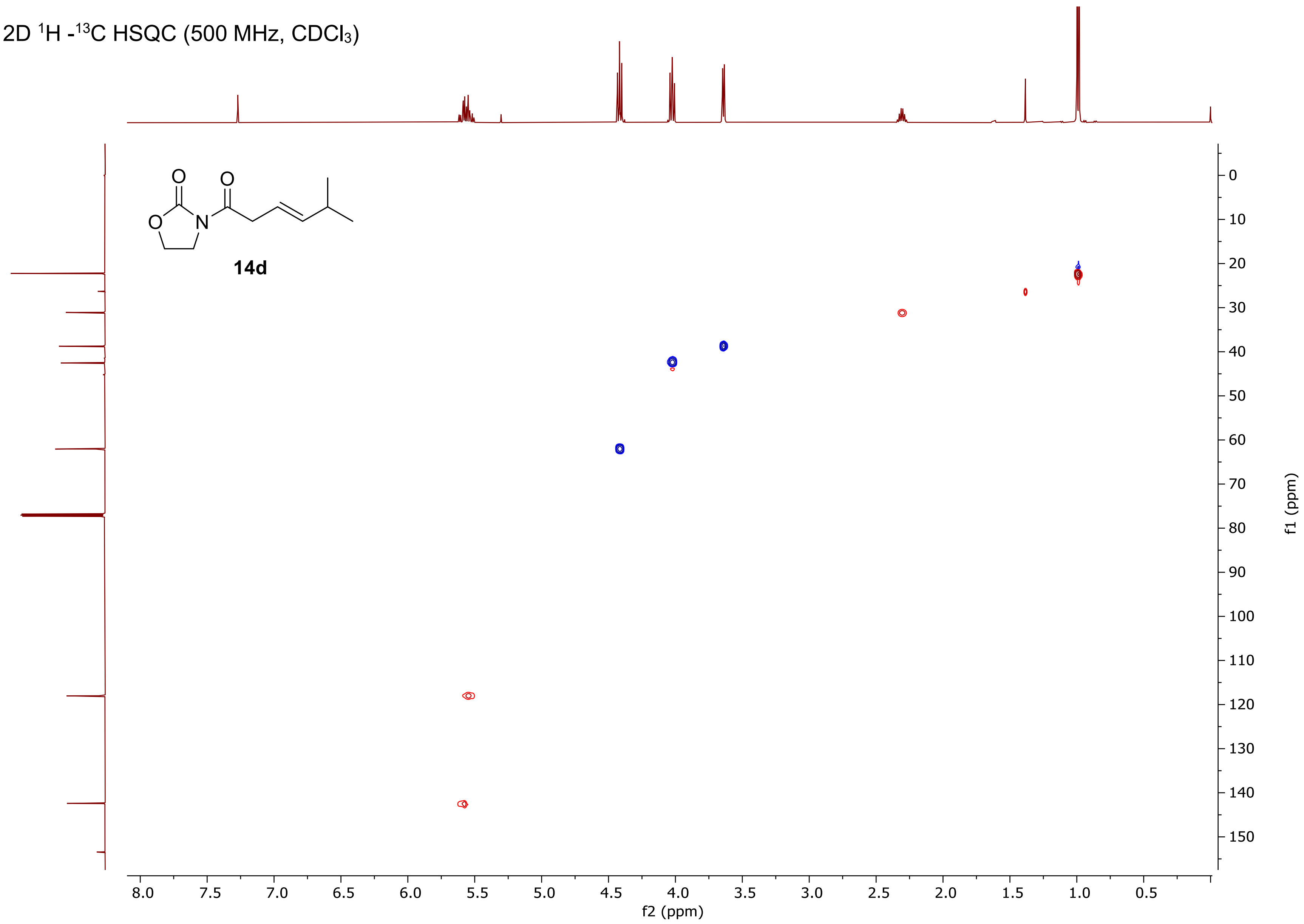

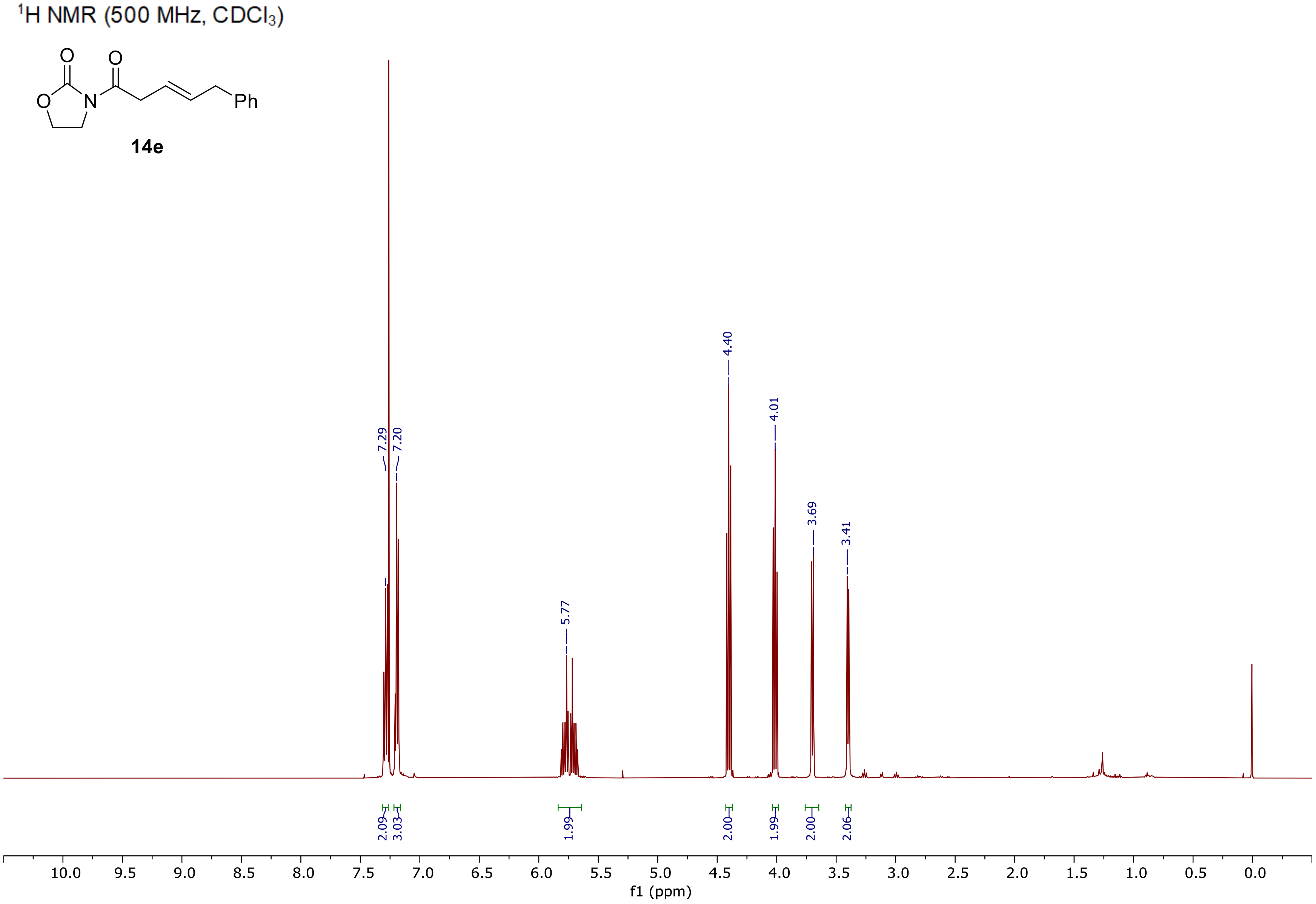

$^{1}$H NMR (500 MHz, $CDCl_3$)
O
O
O
N
Ph
14e
7.29
7.20
5.77
4.40
4.01
3.69
3.41
2.09
3.03
1.99
2.00
1.99
2.00
2.06
10.0 9.5 9.0 8.5 8.0 7.5 7.0 6.5 6.0 5.5 5.0 4.5 4.0 3.5 3.0 2.5 2.0 1.5 1.0 0.5 0.0
f1 (ppm)

$^{13}C\{^1H\}$ NMR (126 MHz, $CDCl_3$)

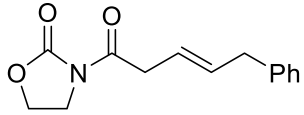

**14e**

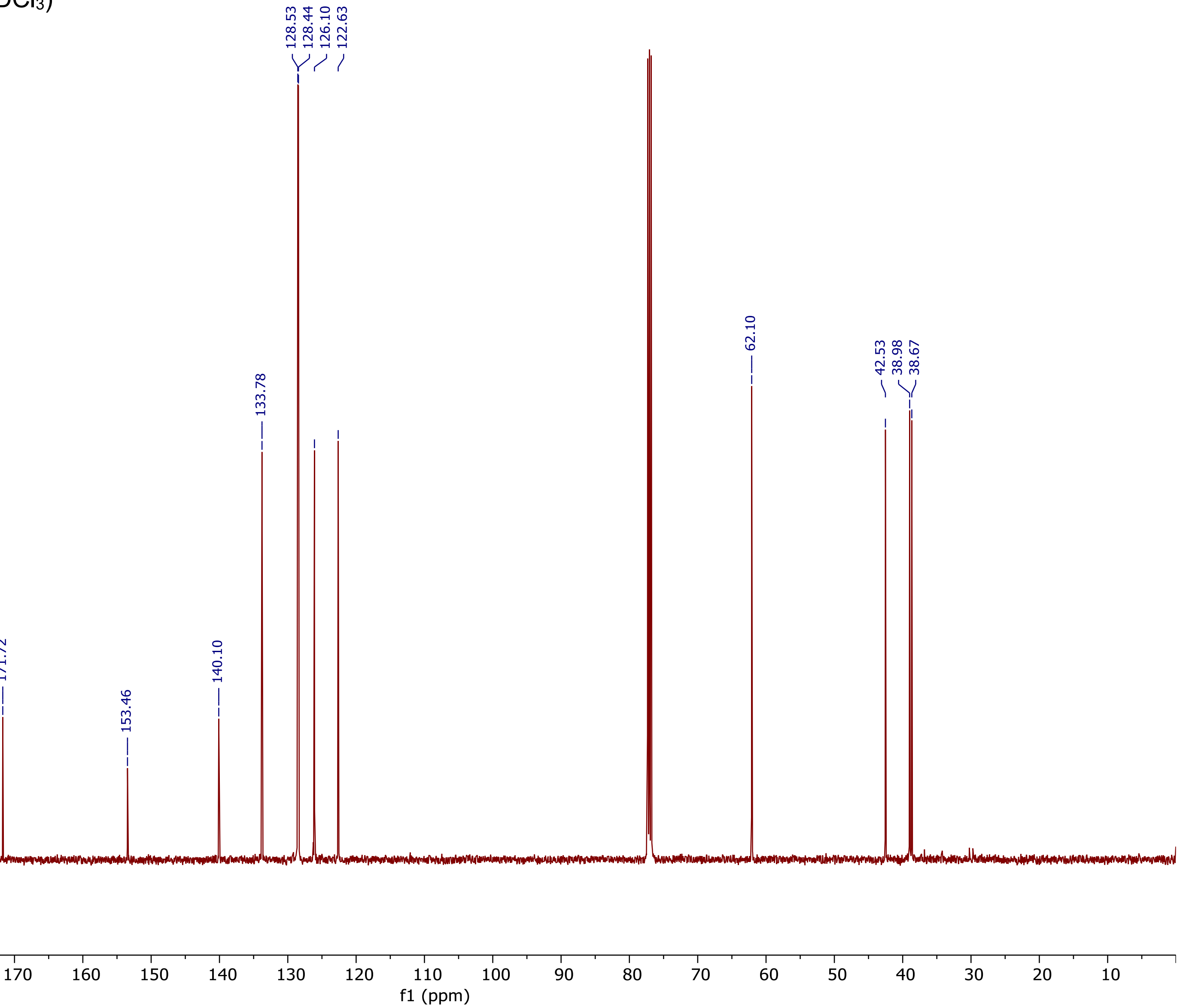

2D $^{1}H$ -$^{1}H$ COSY (500 MHz, $CDCl_3$)

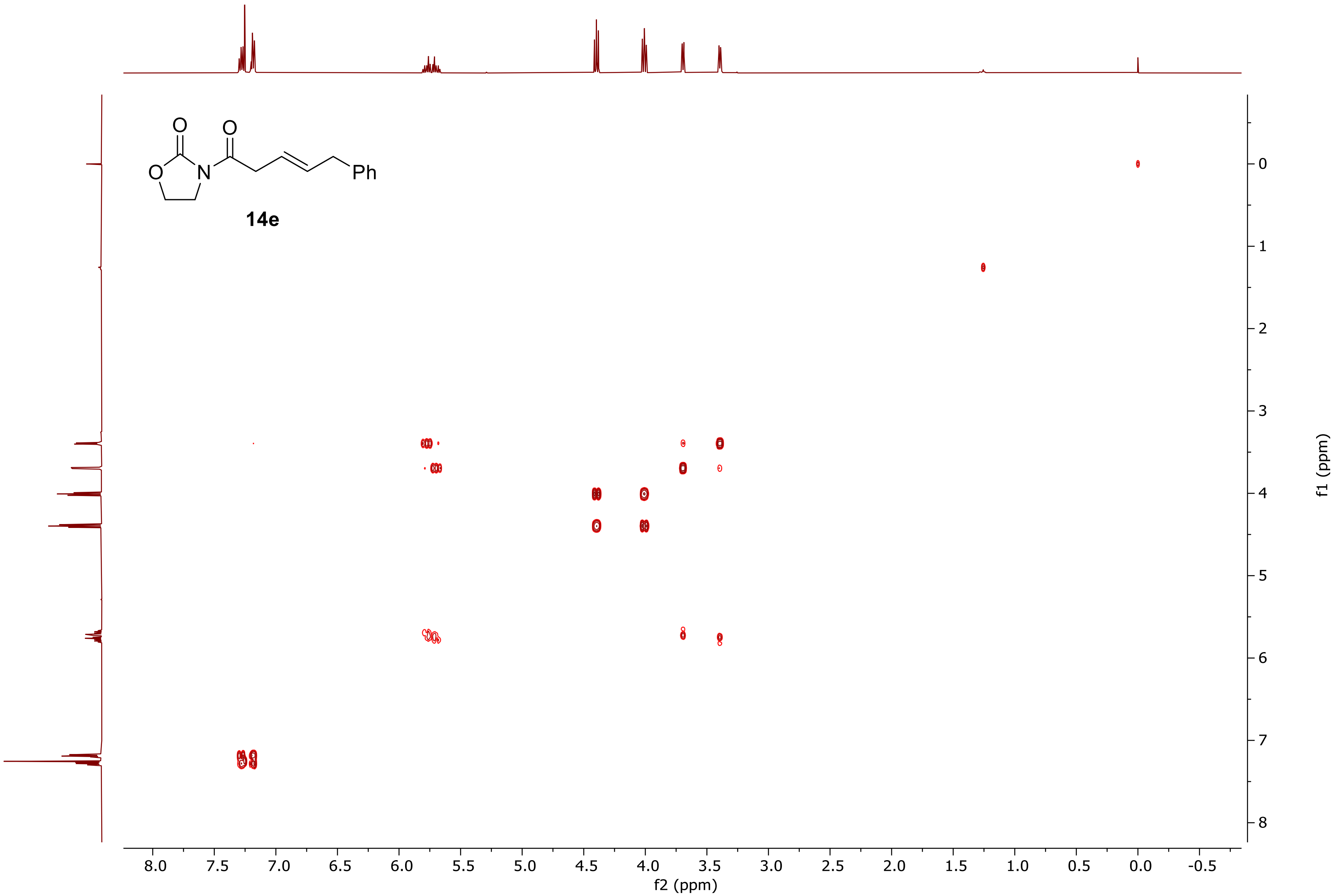

2D $^{1}H$ -$^{13}C$ HSQC (500 MHz, $CDCl_3$)

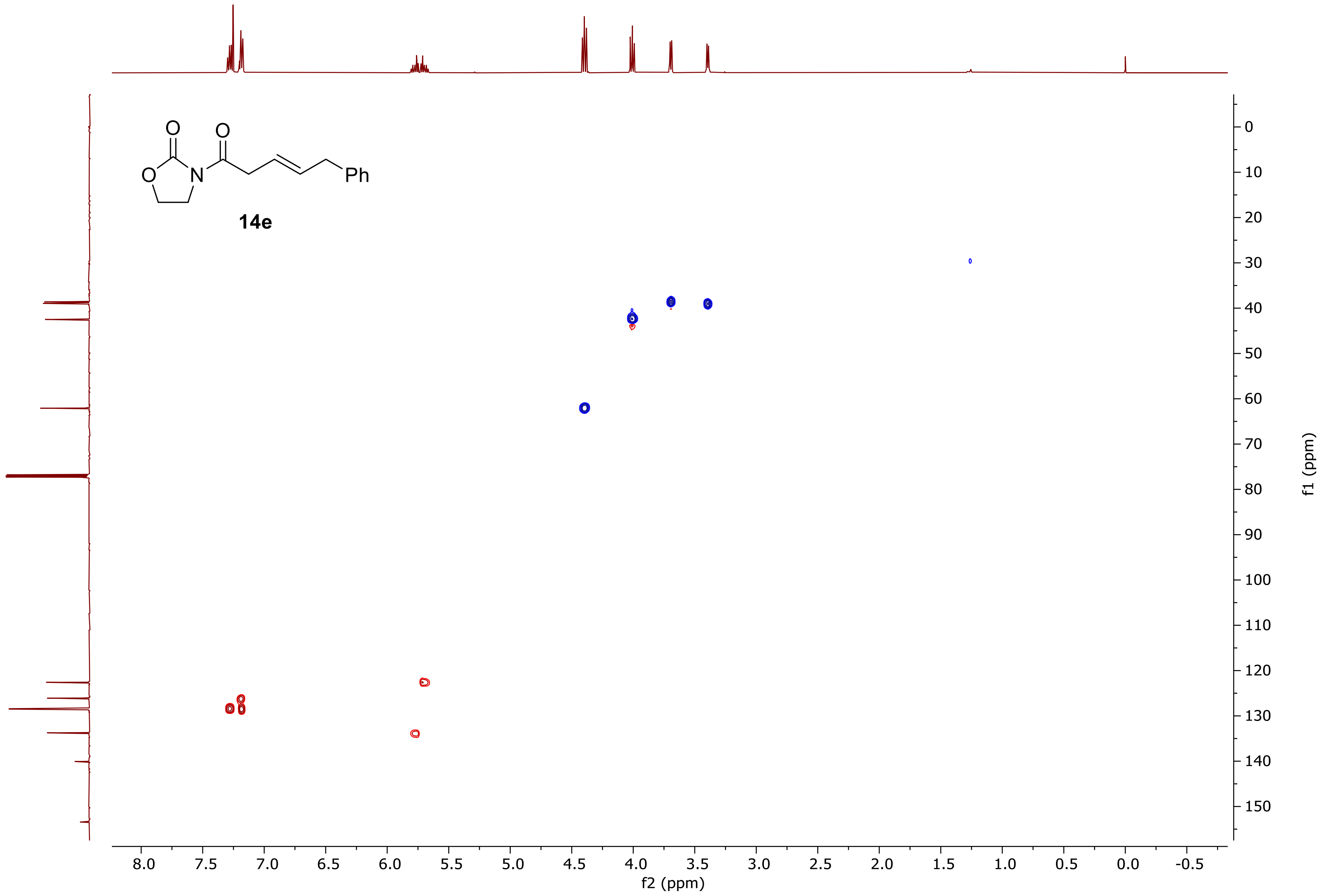

$^{1}H$ NMR (500 MHz, $CDCl_3$)

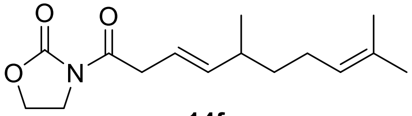


**14f**

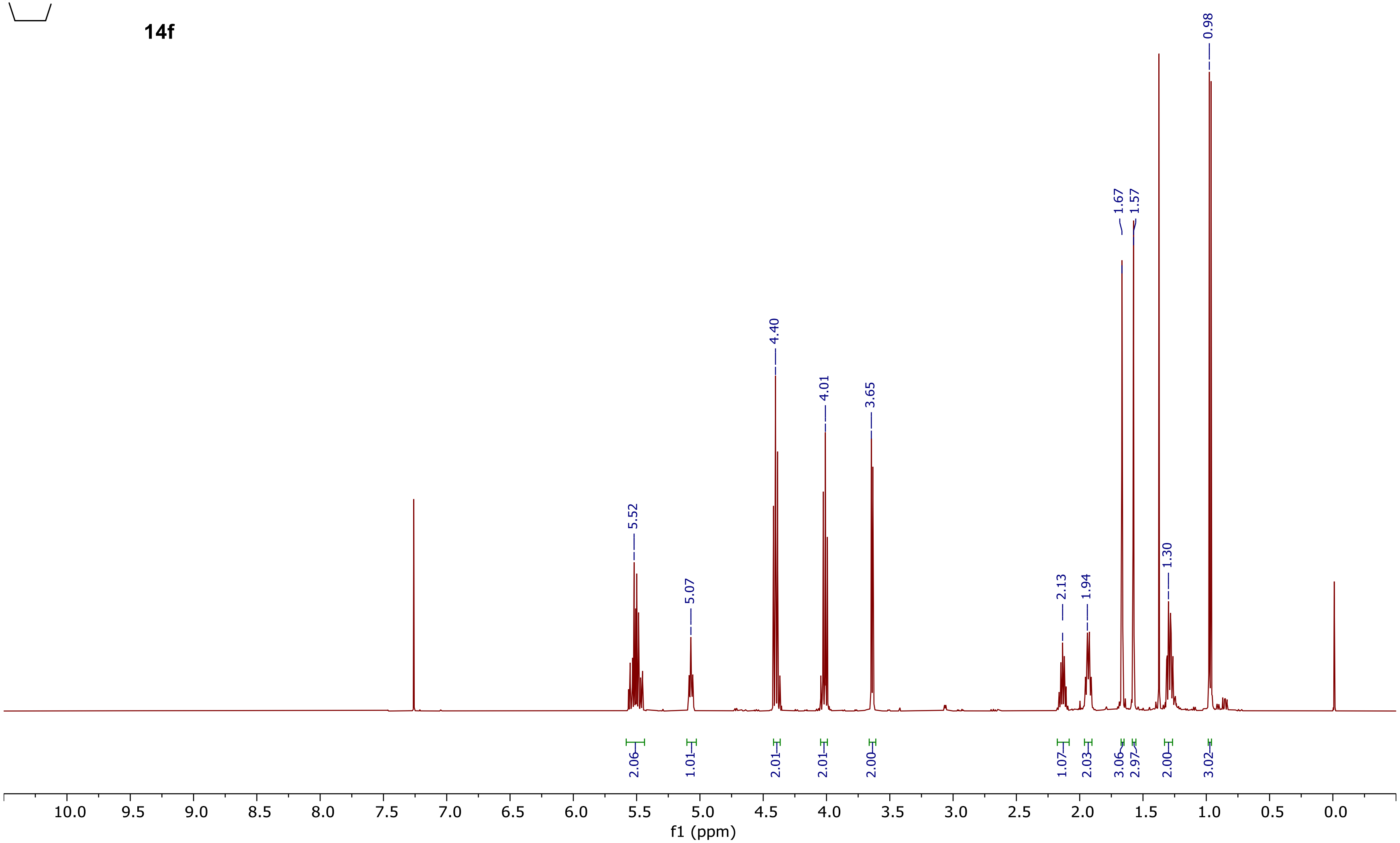

$^{13}C\{^{1}H\}$ NMR (126 MHz, $CDCl_3$)

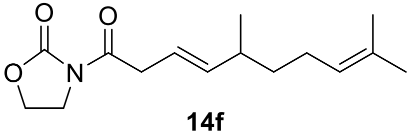

**14f**

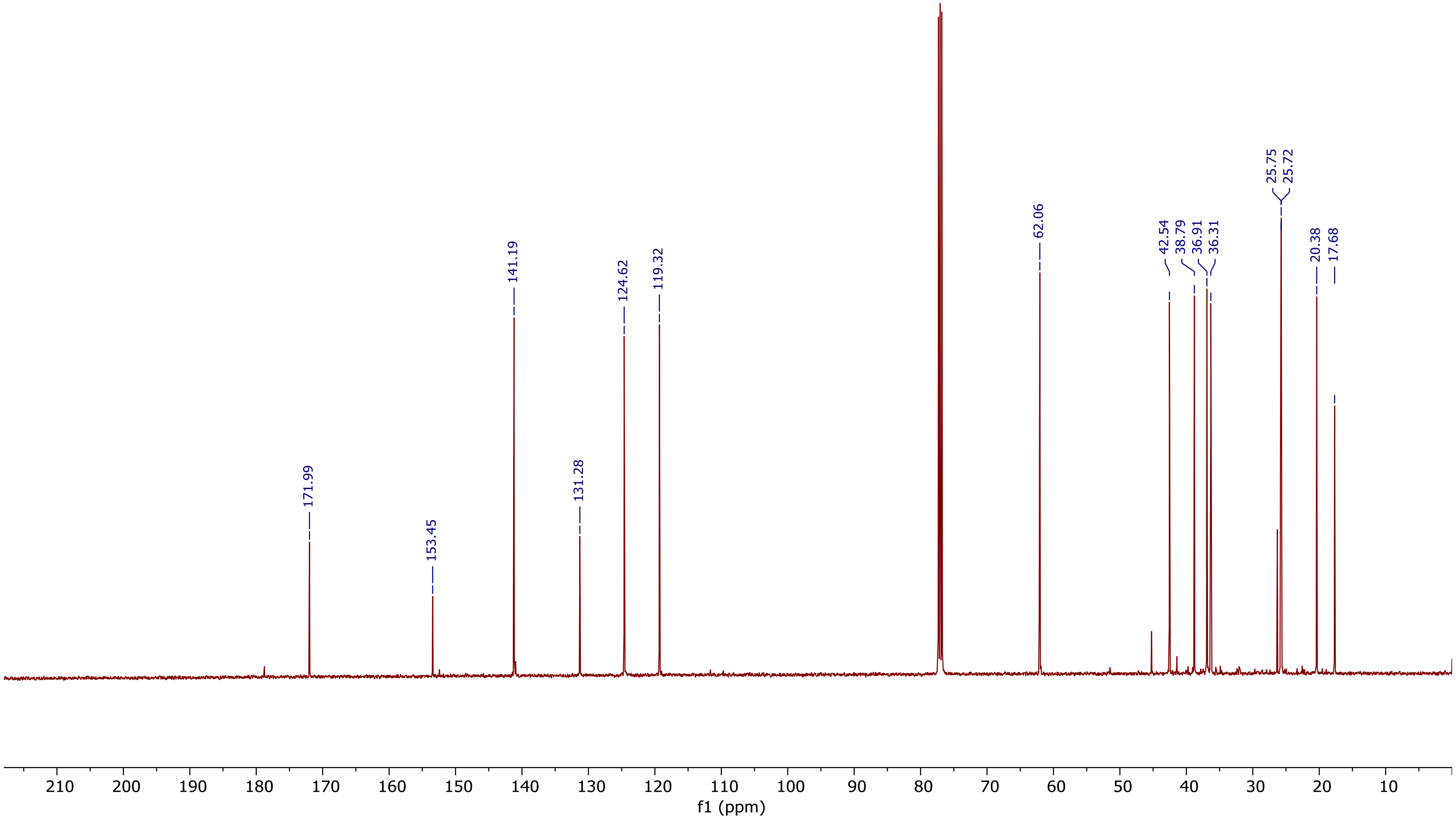

2D $^{1}H$ -$^{1}H$ COSY (500 MHz, $CDCl_3$)

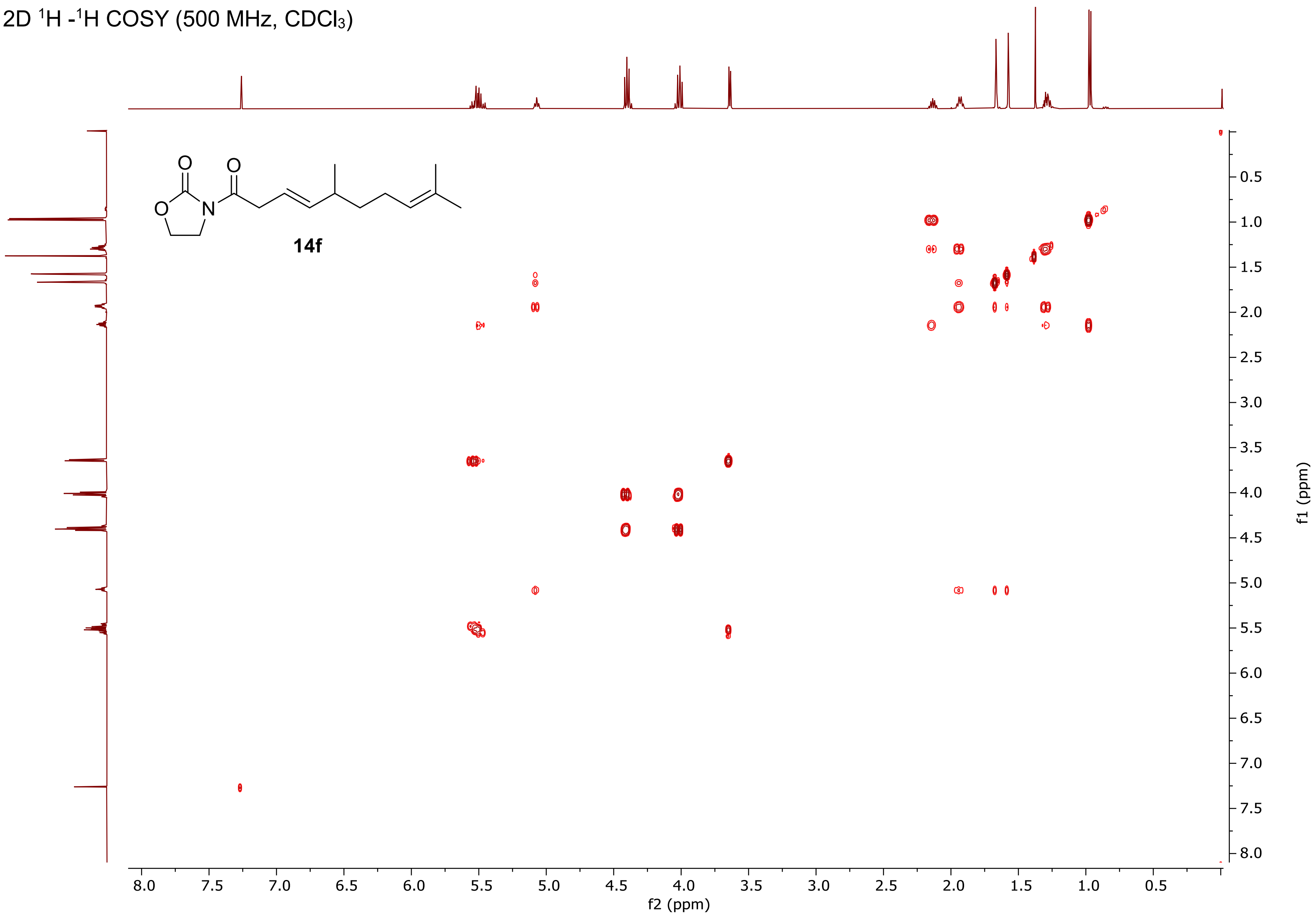

2D $^1$H -$^{13}$C HSQC (500 MHz, $CDCl_3$)

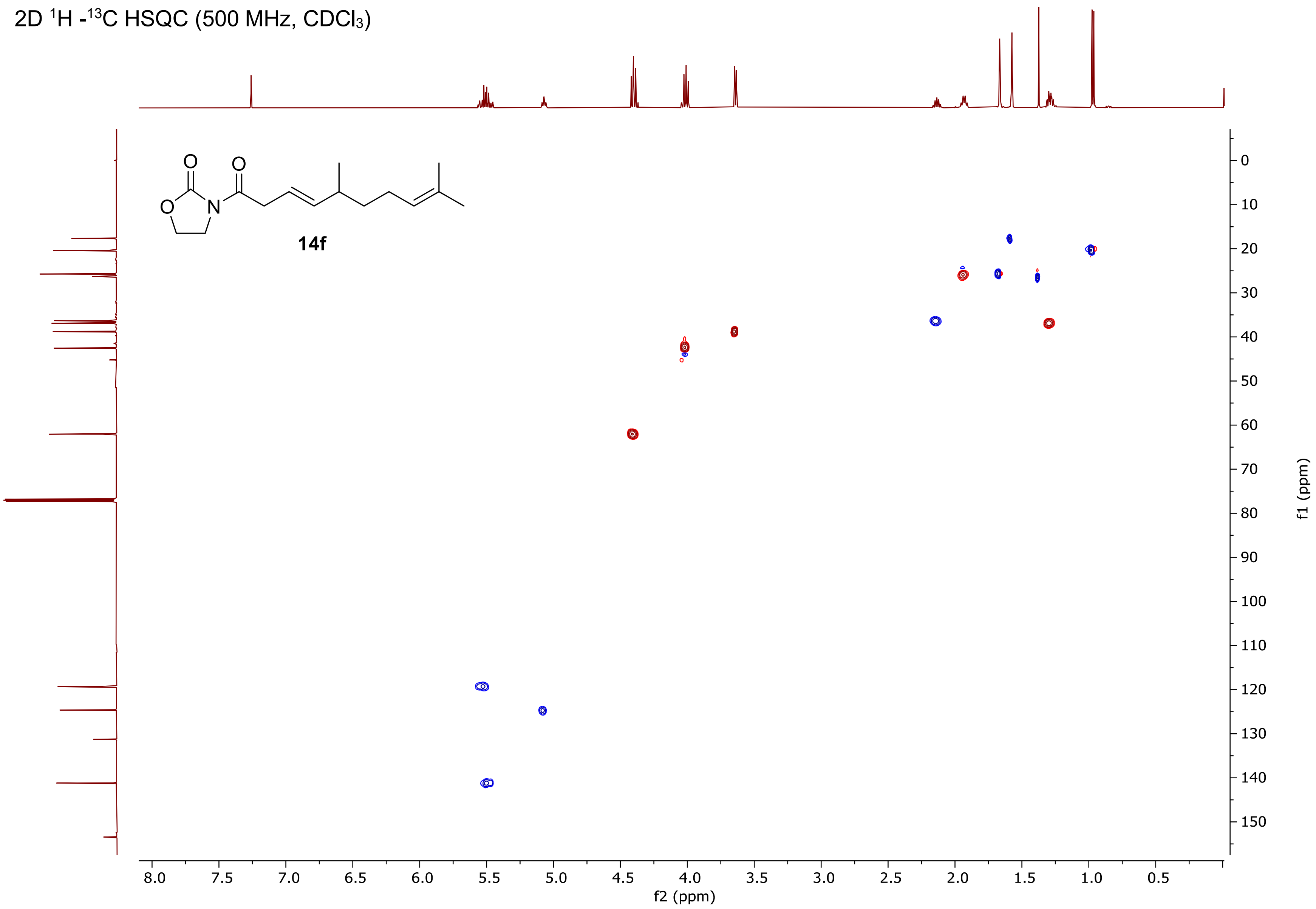

$^1H$ NMR (400 MHz, $CDCl_3$)

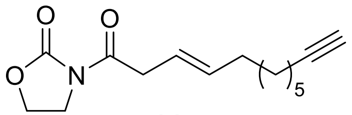

**14g**

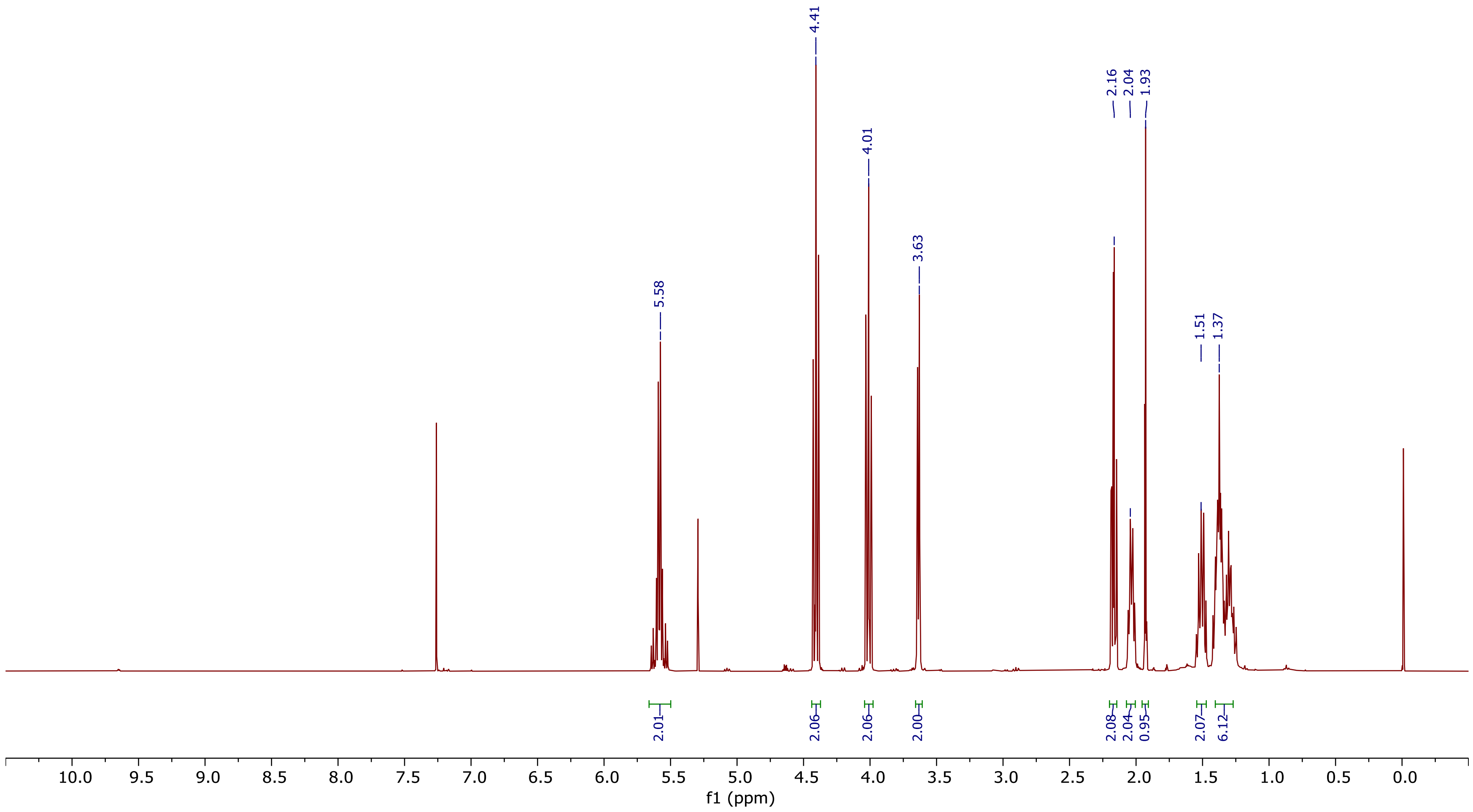

$^{13}C\{^{1}H\}$ NMR (101 MHz, $CDCl_3$)

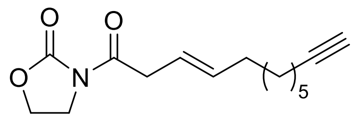

**14g**

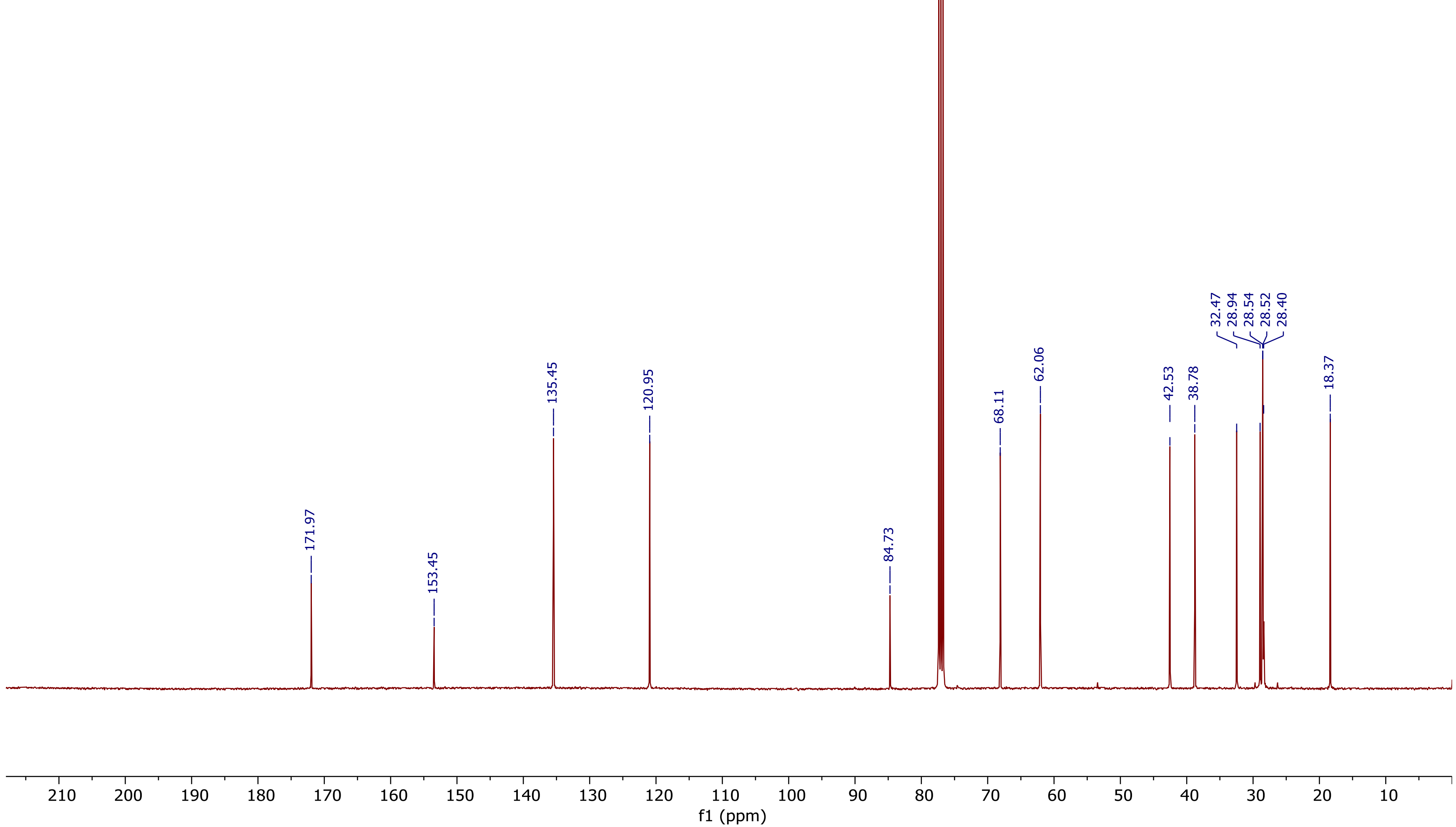

2D $^1$H -$^1$H COSY (400 MHz, $CDCl_3$)

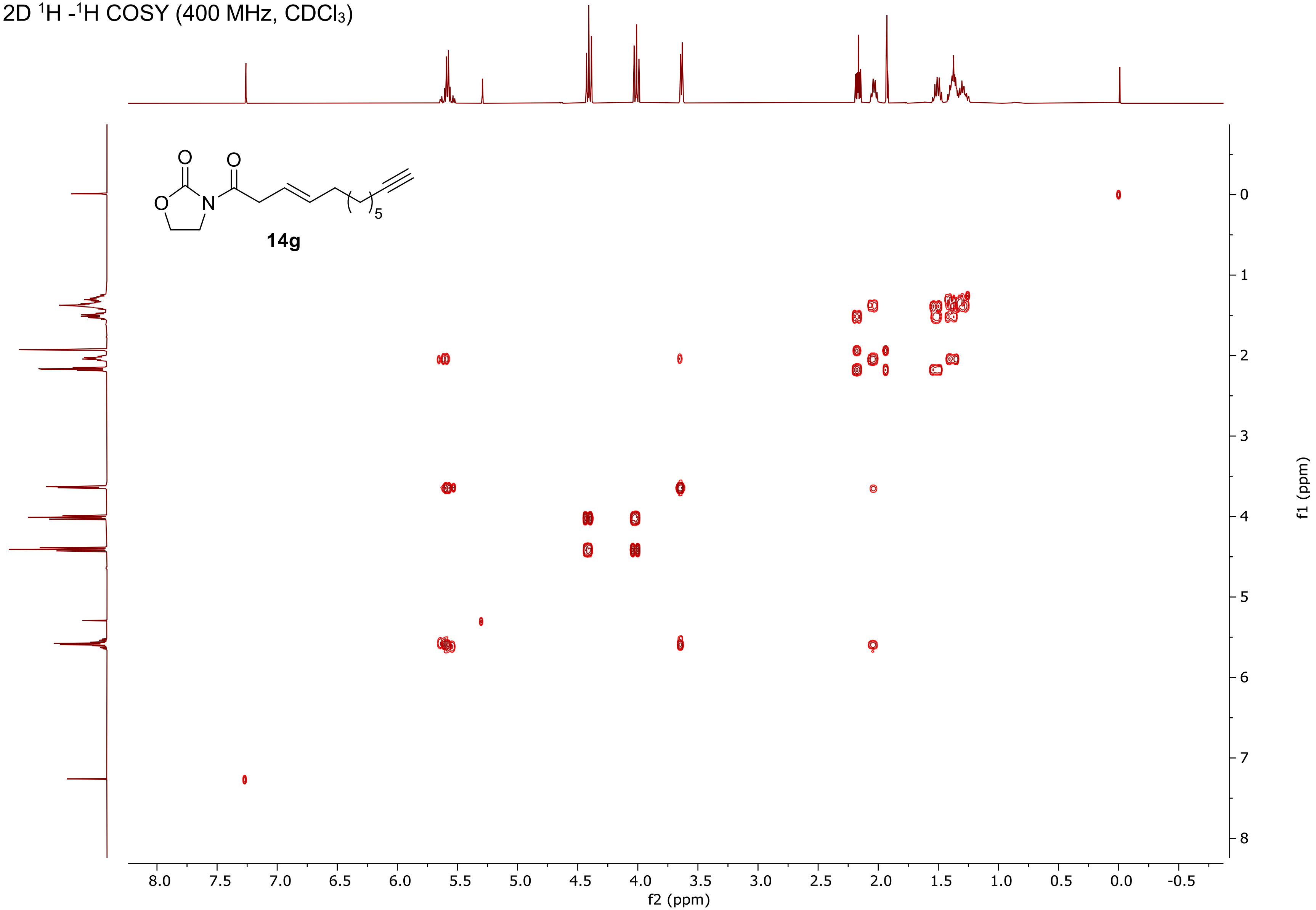

2D $^{1}H$ -$^{13}C$ HSQC (400 MHz, $CDCl_3$)

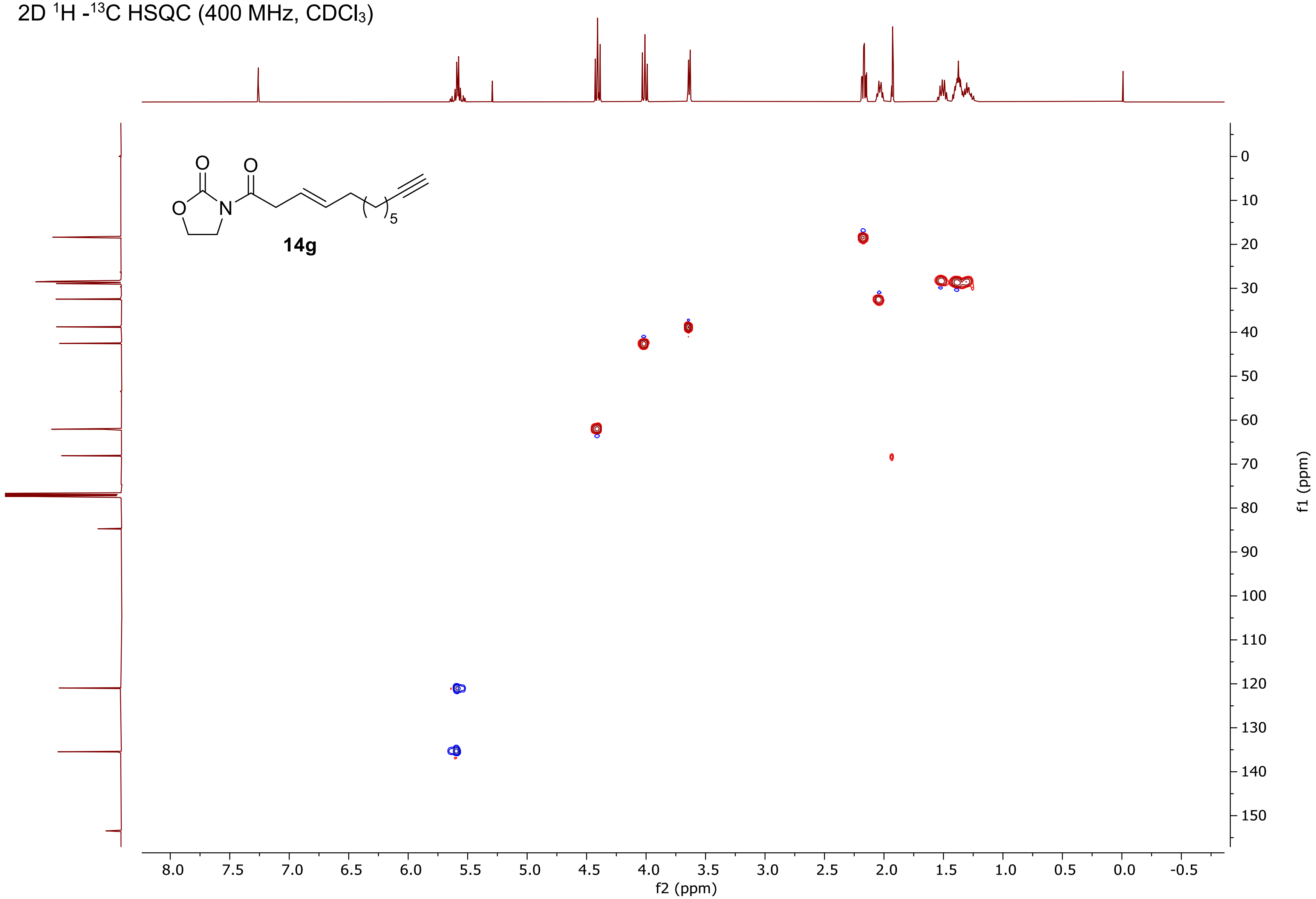

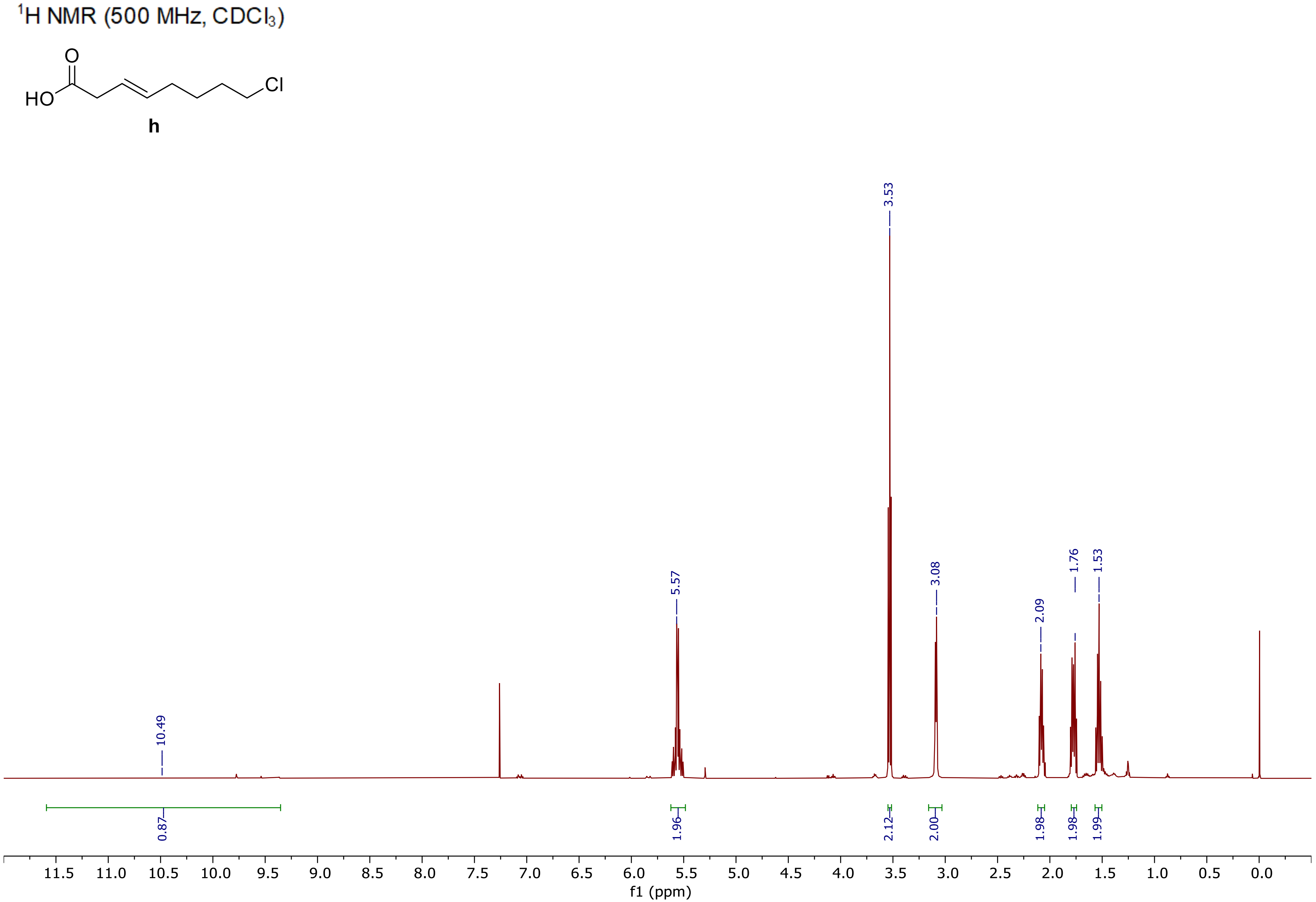

¹H NMR (500 MHz, CDCl₃)
O
HO
Cl
h
10.49
5.57
3.53
3.08
2.09
1.76
1.53
0.87
1.96
2.12
2.00
1.98
1.98
1.99
11.5 11.0 10.5 10.0 9.5 9.0 8.5 8.0 7.5 7.0 6.5 6.0 5.5 5.0 4.5 4.0 3.5 3.0 2.5 2.0 1.5 1.0 0.5 0.0
f1 (ppm)

$^{13}C\{^{1}H\}$ NMR (126 MHz, $CDCl_3$)

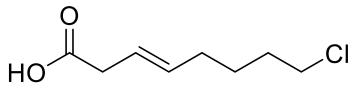


**h**

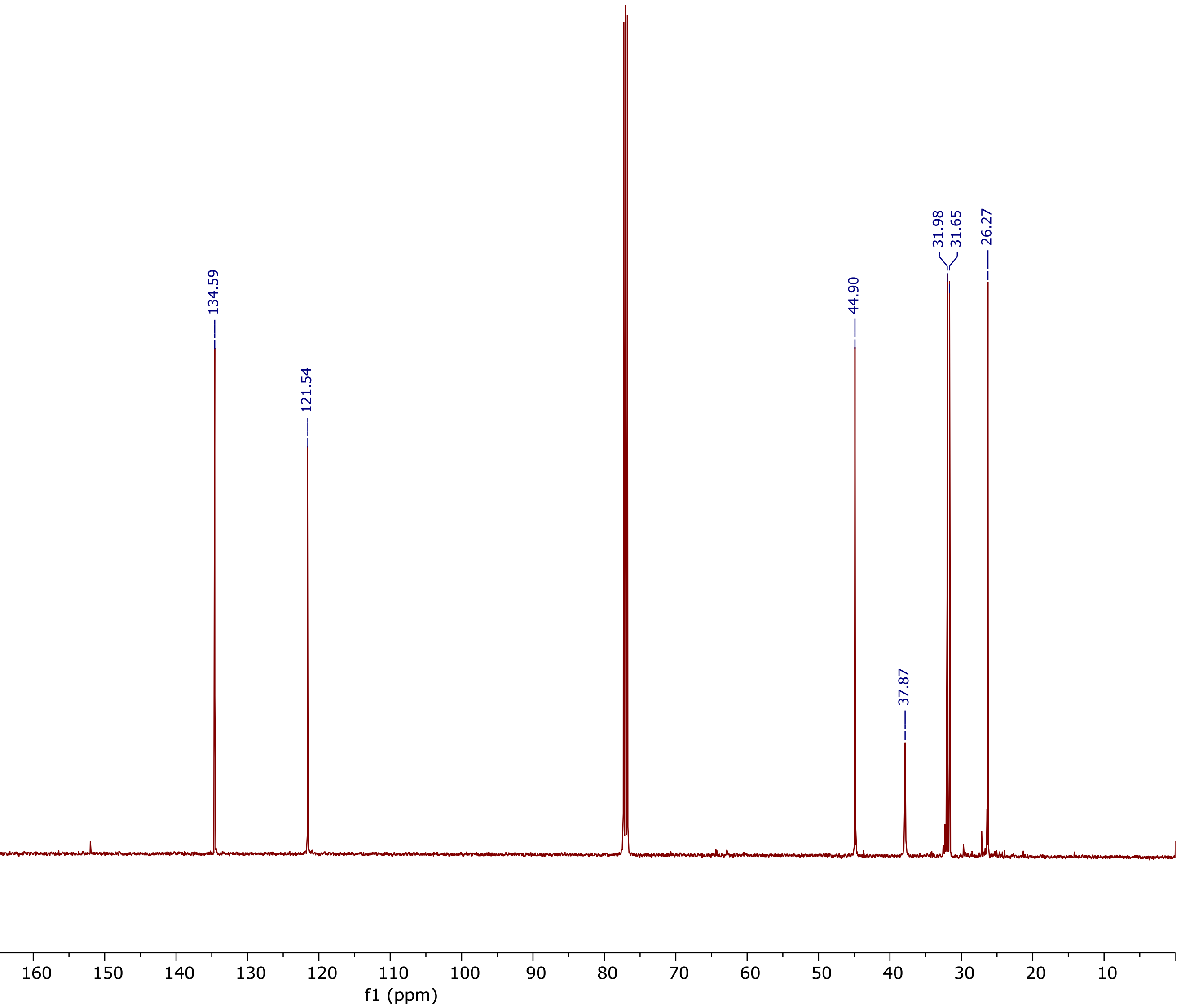

2D $^1$H -$^1$H COSY (500 MHz, $CDCl_3$)

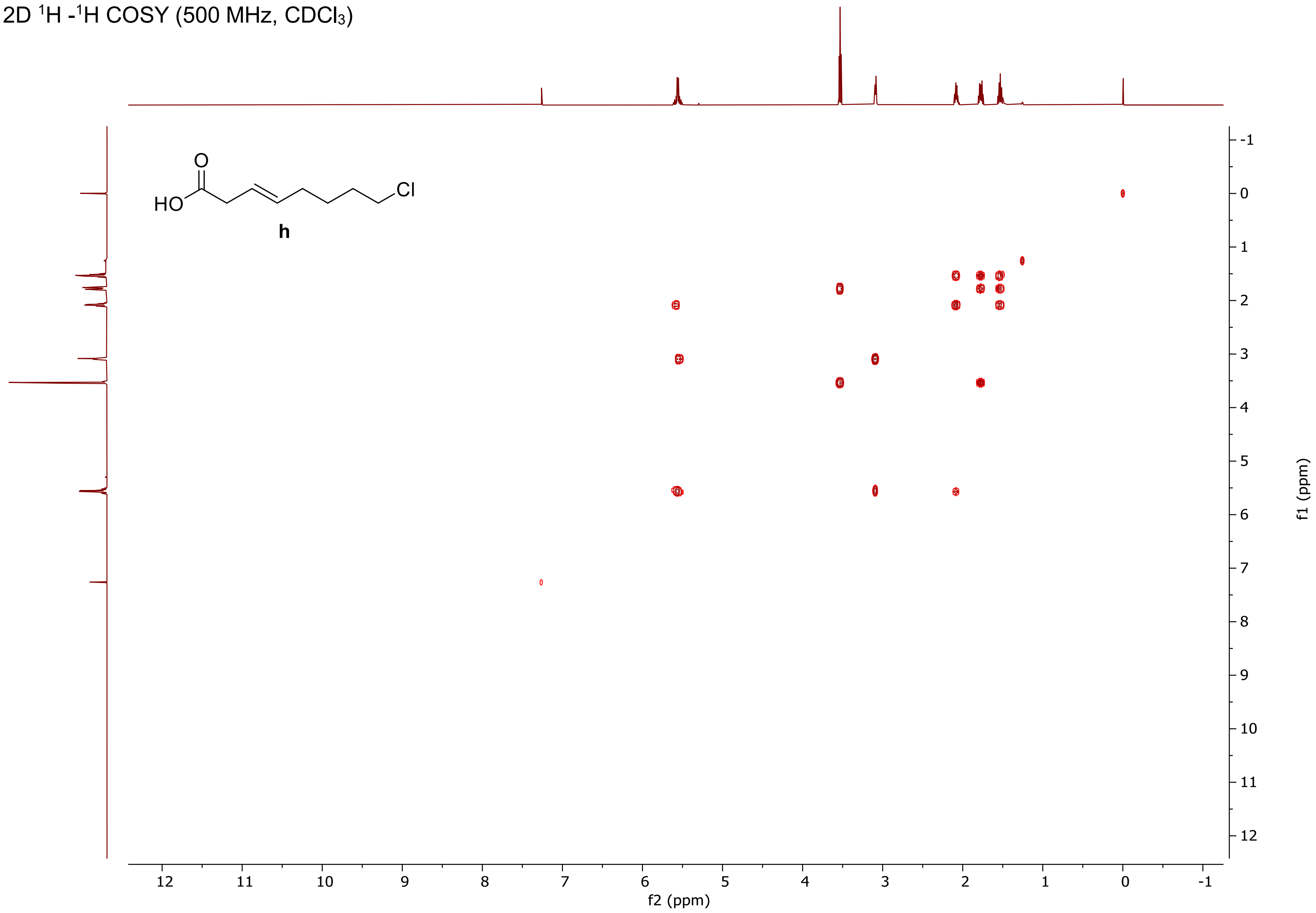

2D $^{1}$H -$^{13}$C HSQC (500 MHz, $CDCl_3$)

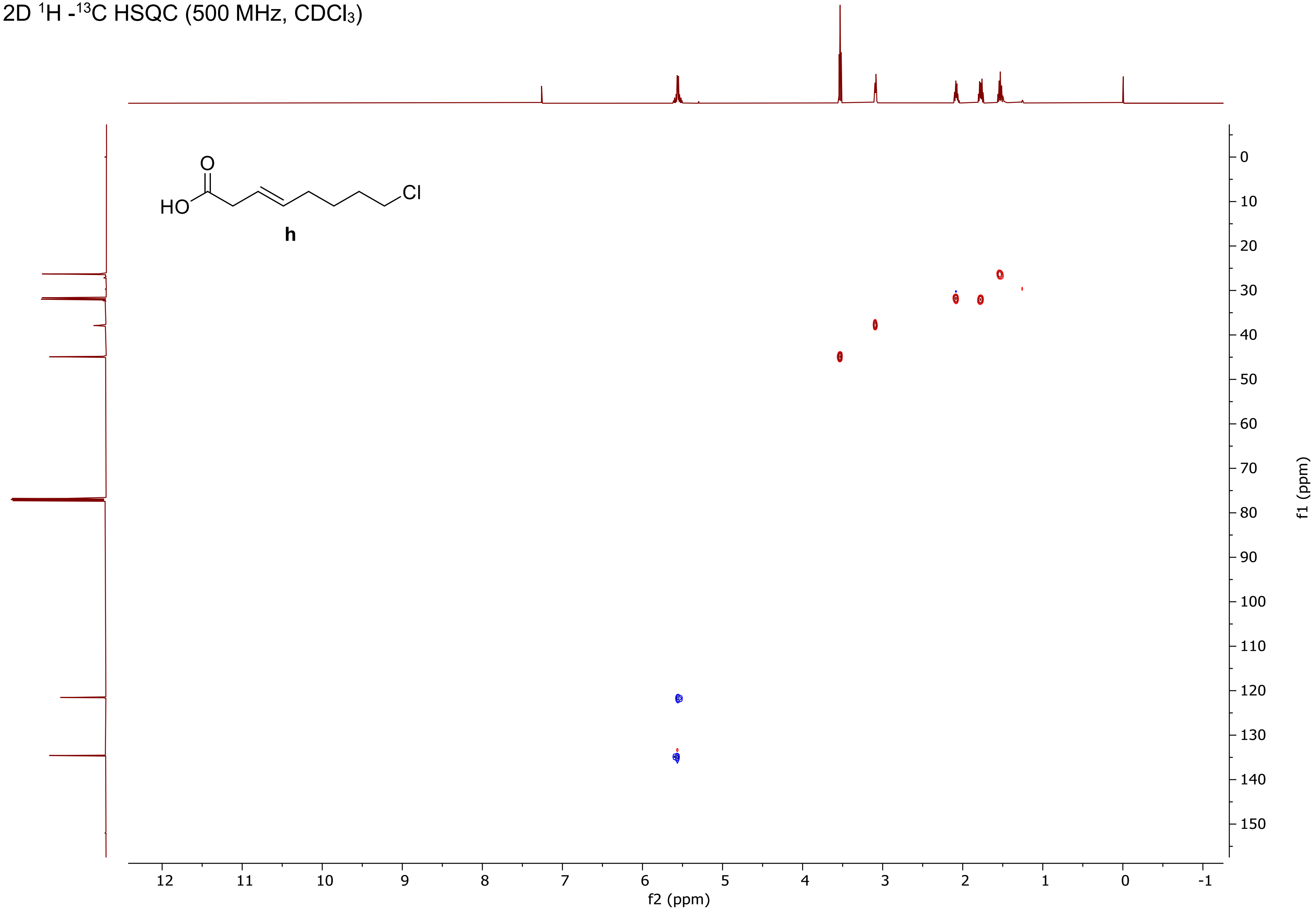

$^{1}H$ NMR (500 MHz, $CDCl_3$)

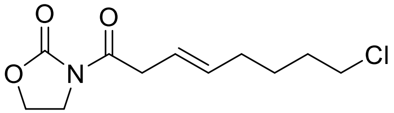


**14h**

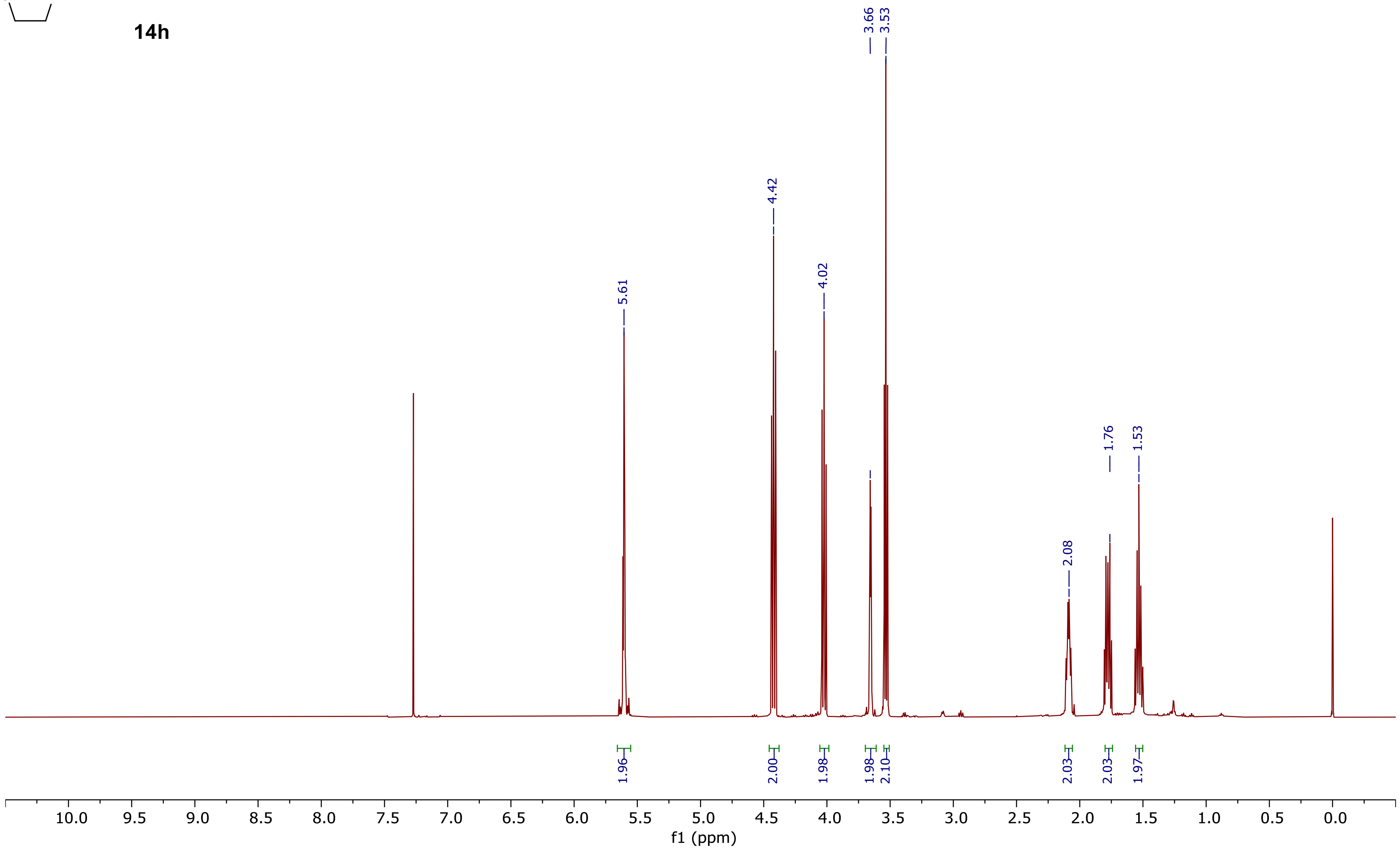

$^{13}C\{^{1}H\}$ NMR (126 MHz, $CDCl_3$)

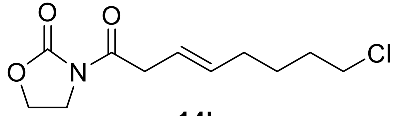

**14h**

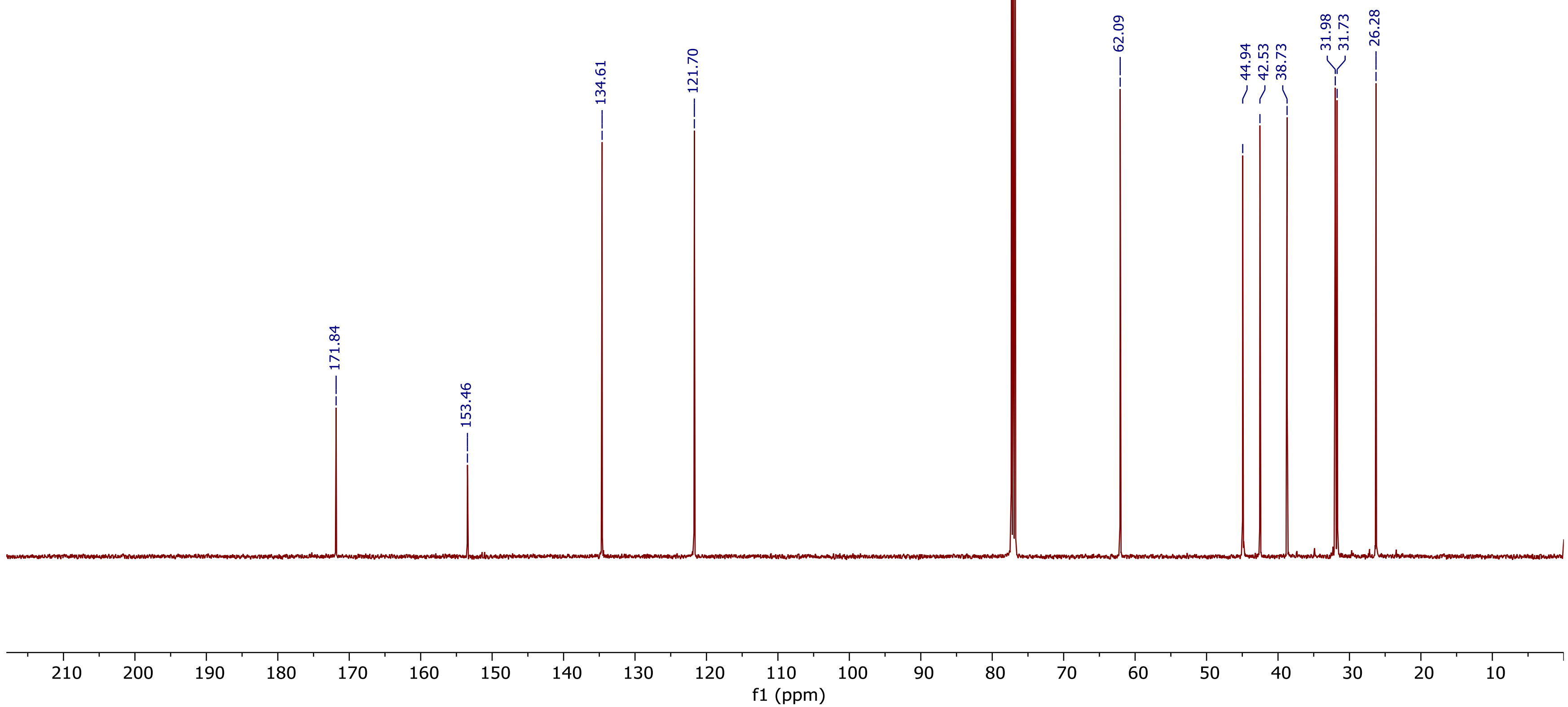

2D $^{1}H$ -$^{1}H$ COSY (500 MHz, $CDCl_3$)

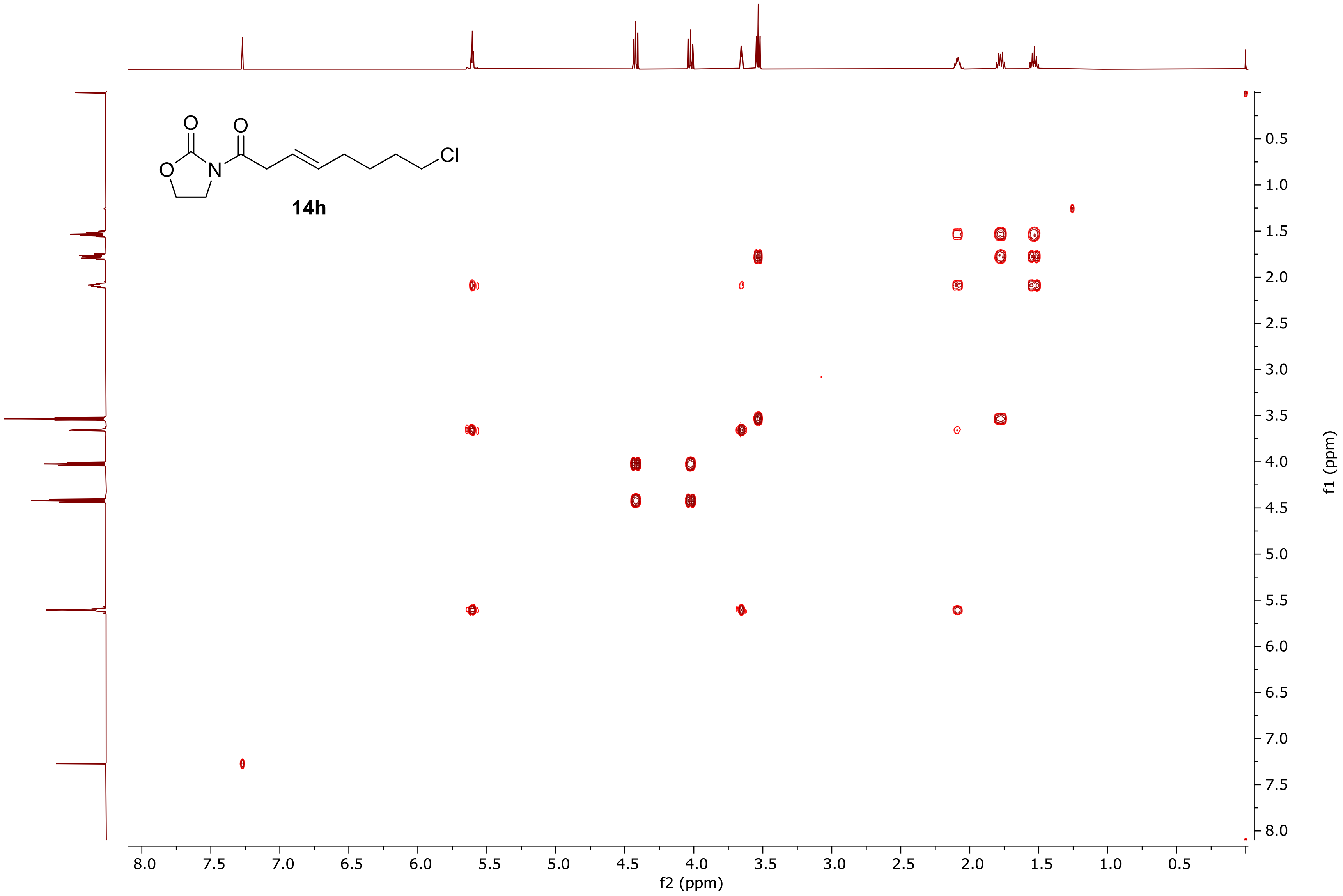

2D $^{1}H$ -$^{13}C$ HSQC (500 MHz, $CDCl_3$)

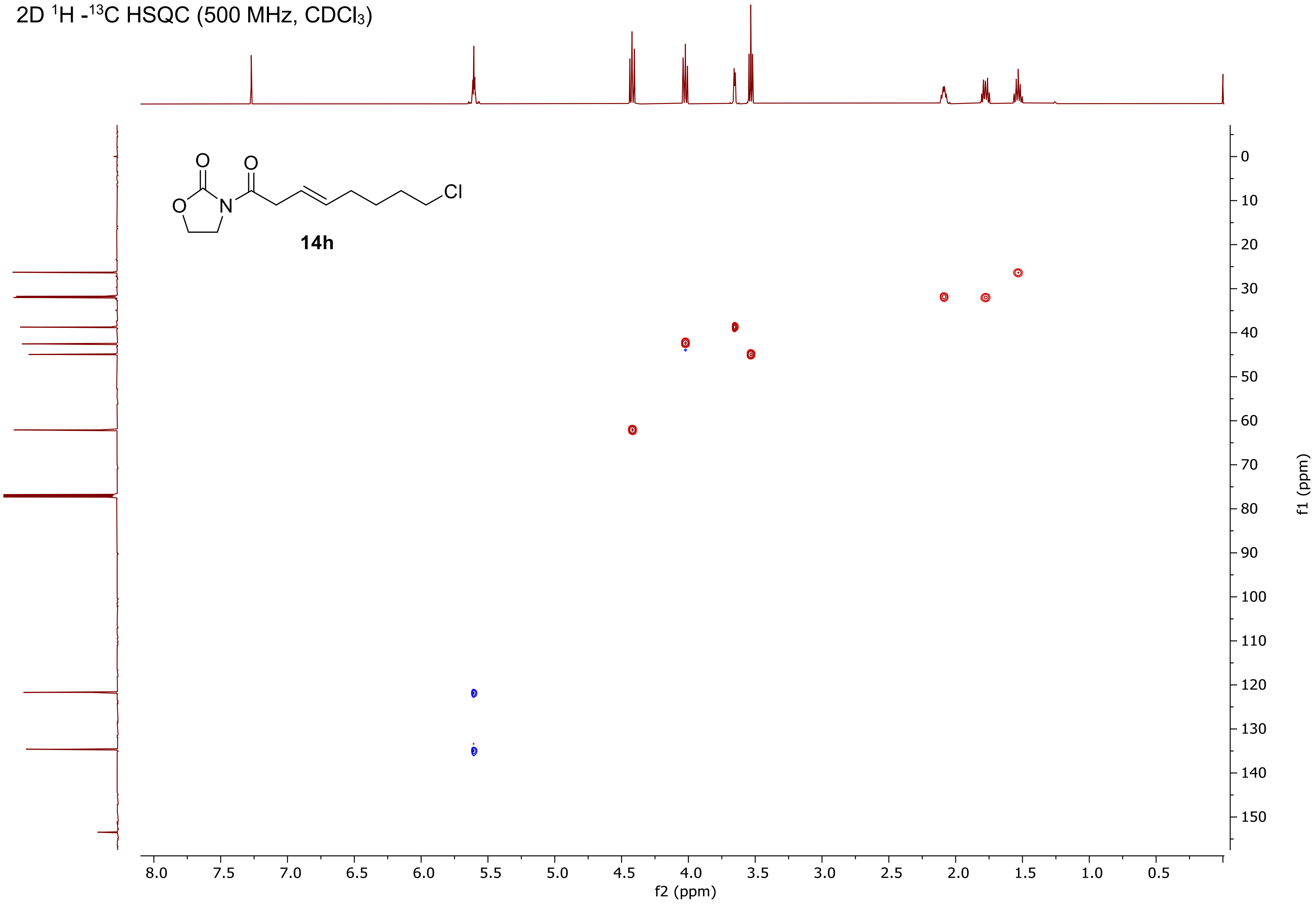

$^1H$ NMR (400 MHz, $CDCl_3$)

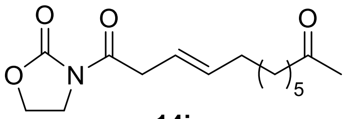

**14i**

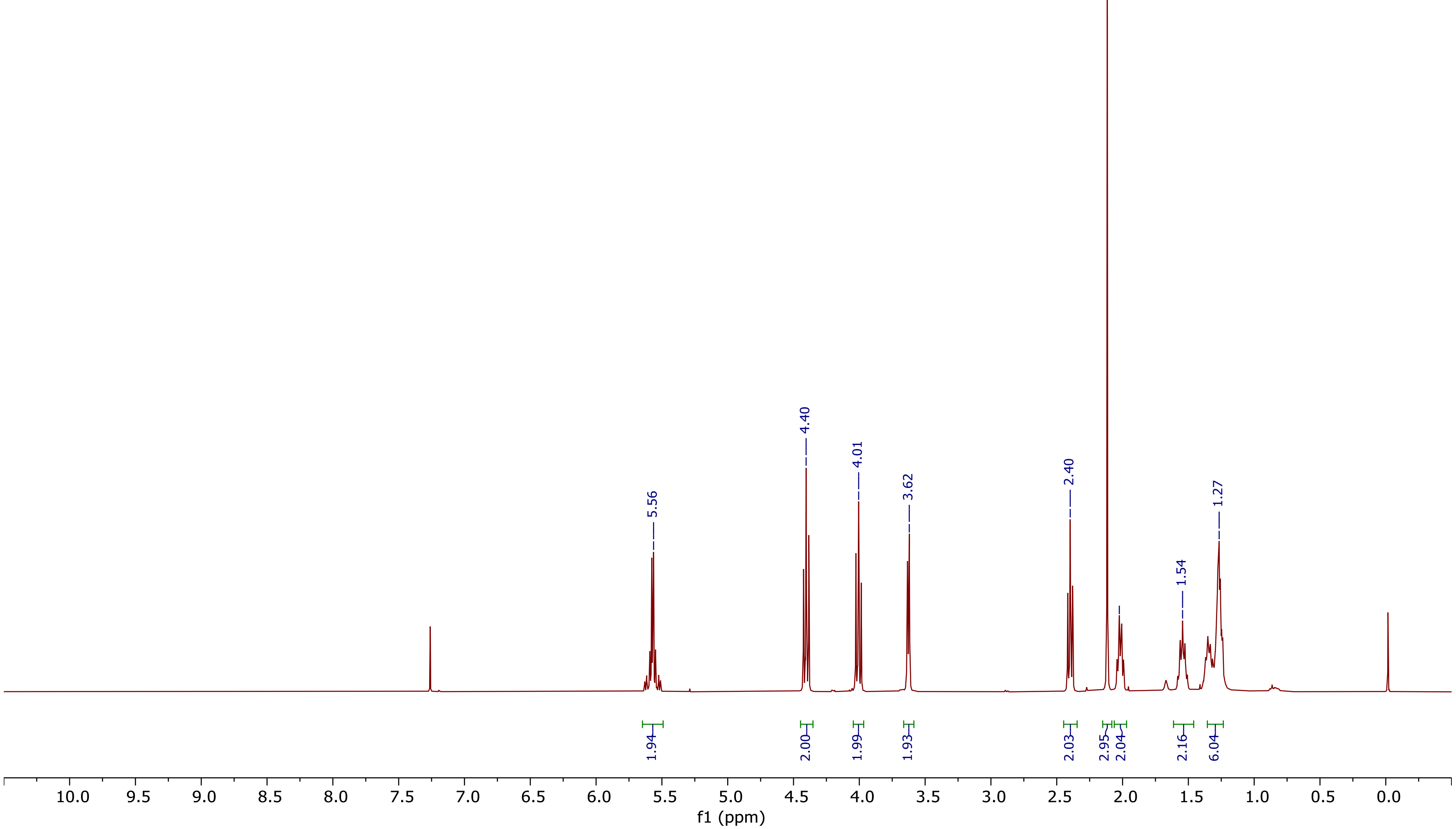

$^{13}C\{^1H\}$ NMR (101 MHz, $CDCl_3$)

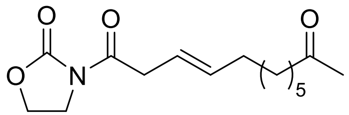

**14i**

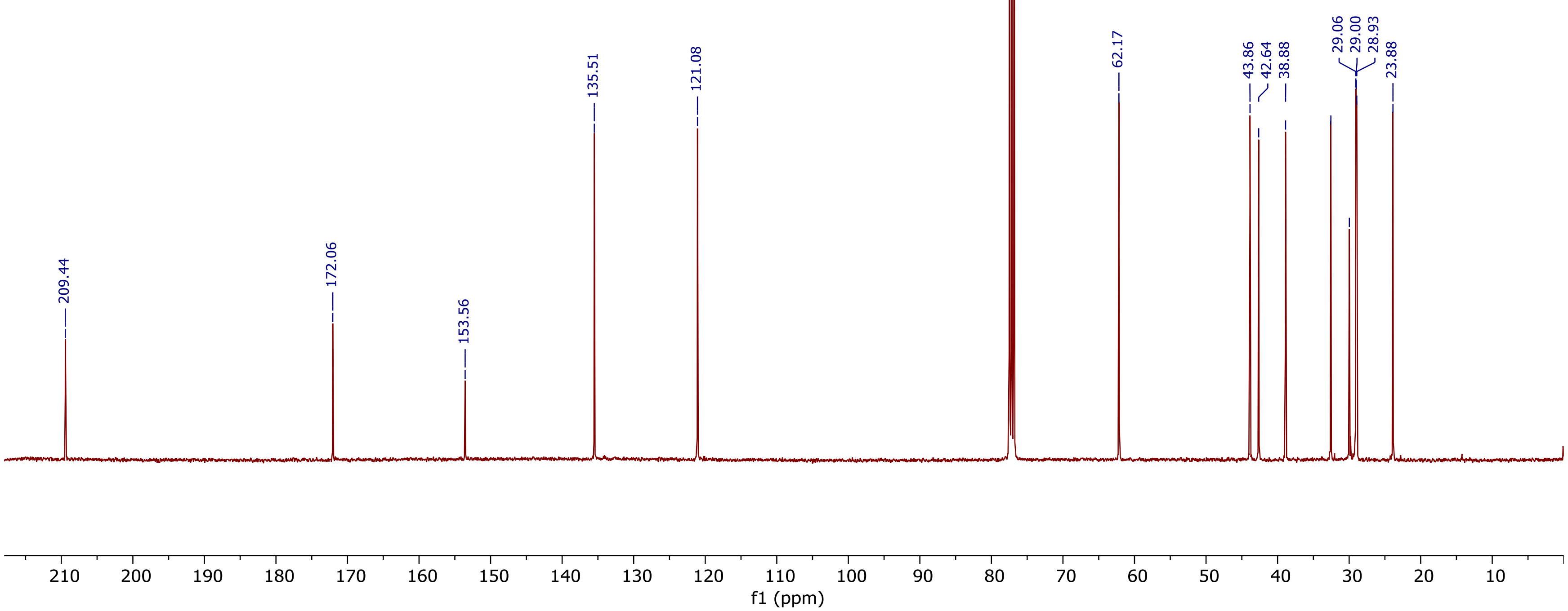

2D $^1H$ -$^1H$ COSY (400 MHz, $CDCl_3$)

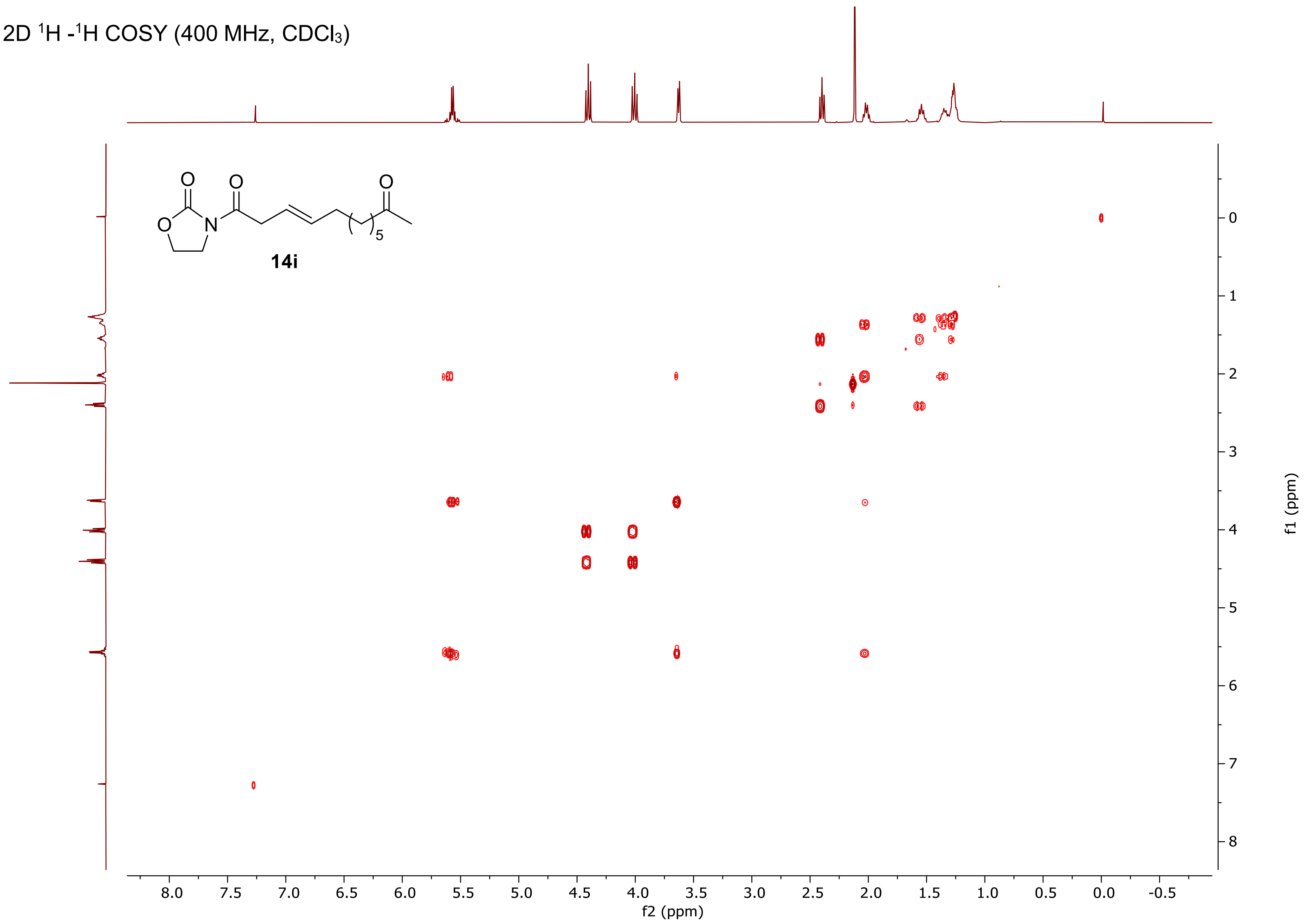

2D $^1H$ -$^{13}C$ HSQC (400 MHz, $CDCl_3$)

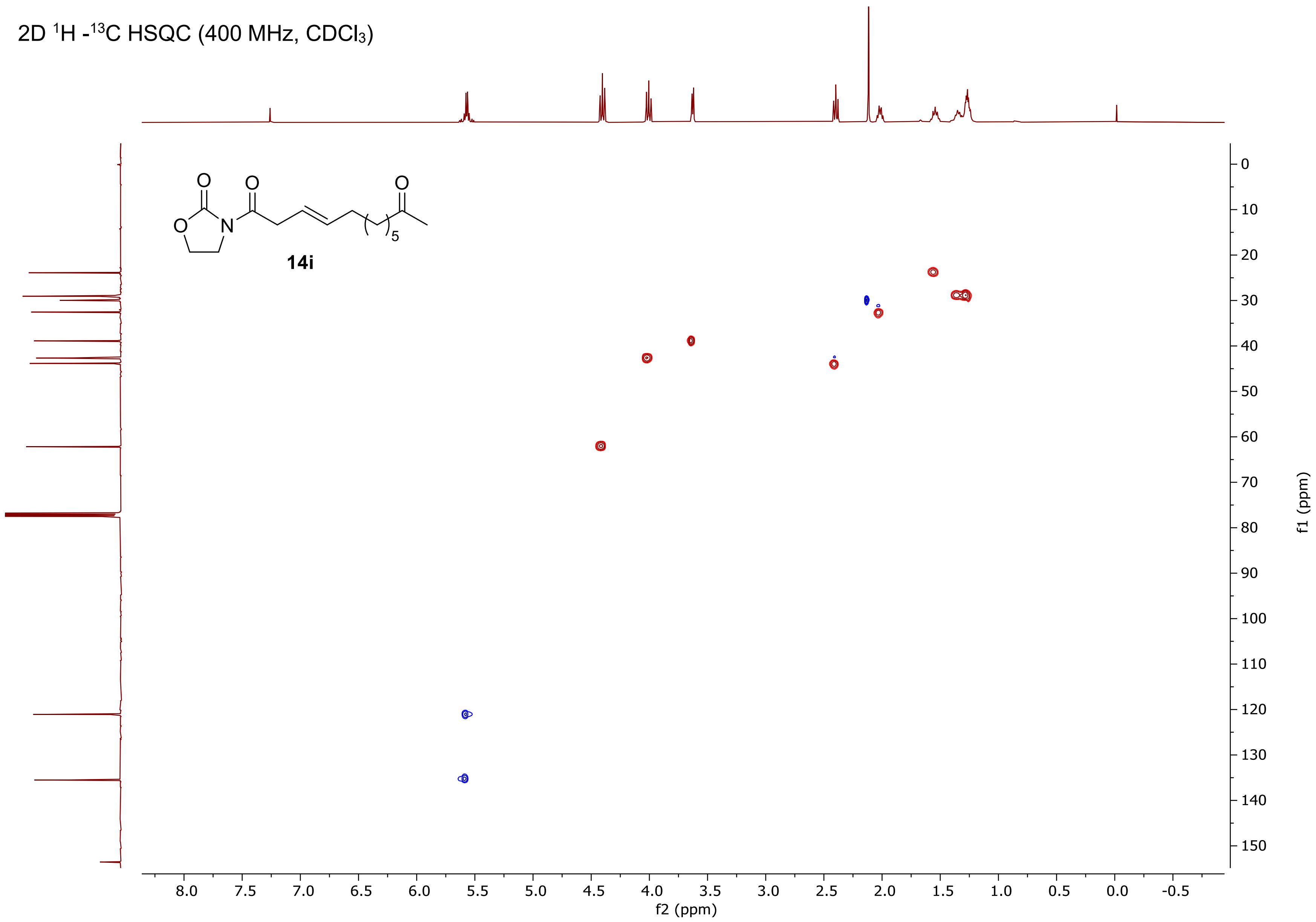

$^1H$ NMR (400 MHz, $CDCl_3$)

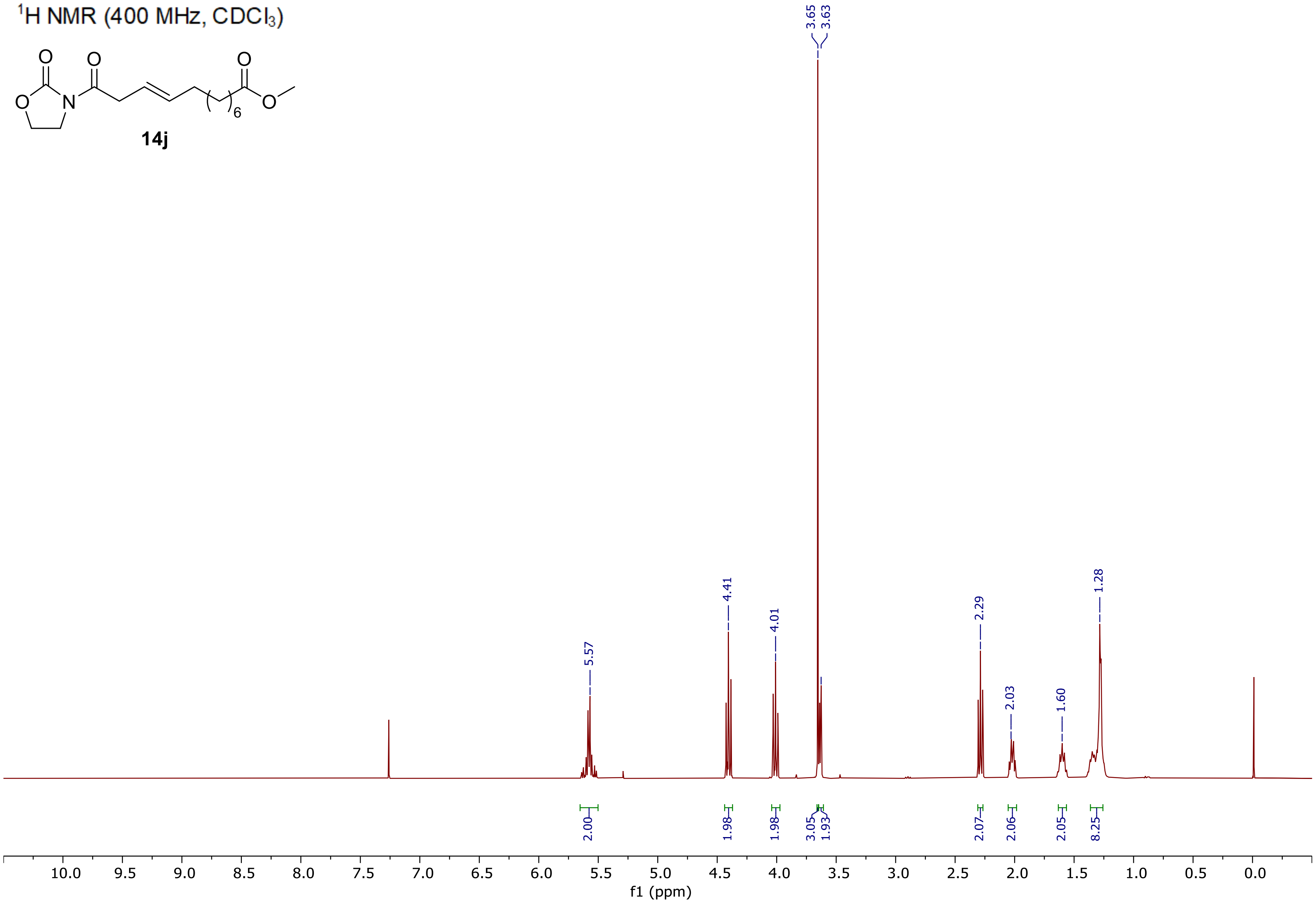

$^{13}C\{^{1}H\}$ NMR (101 MHz, $CDCl_3$)

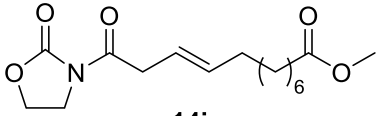

**14j**

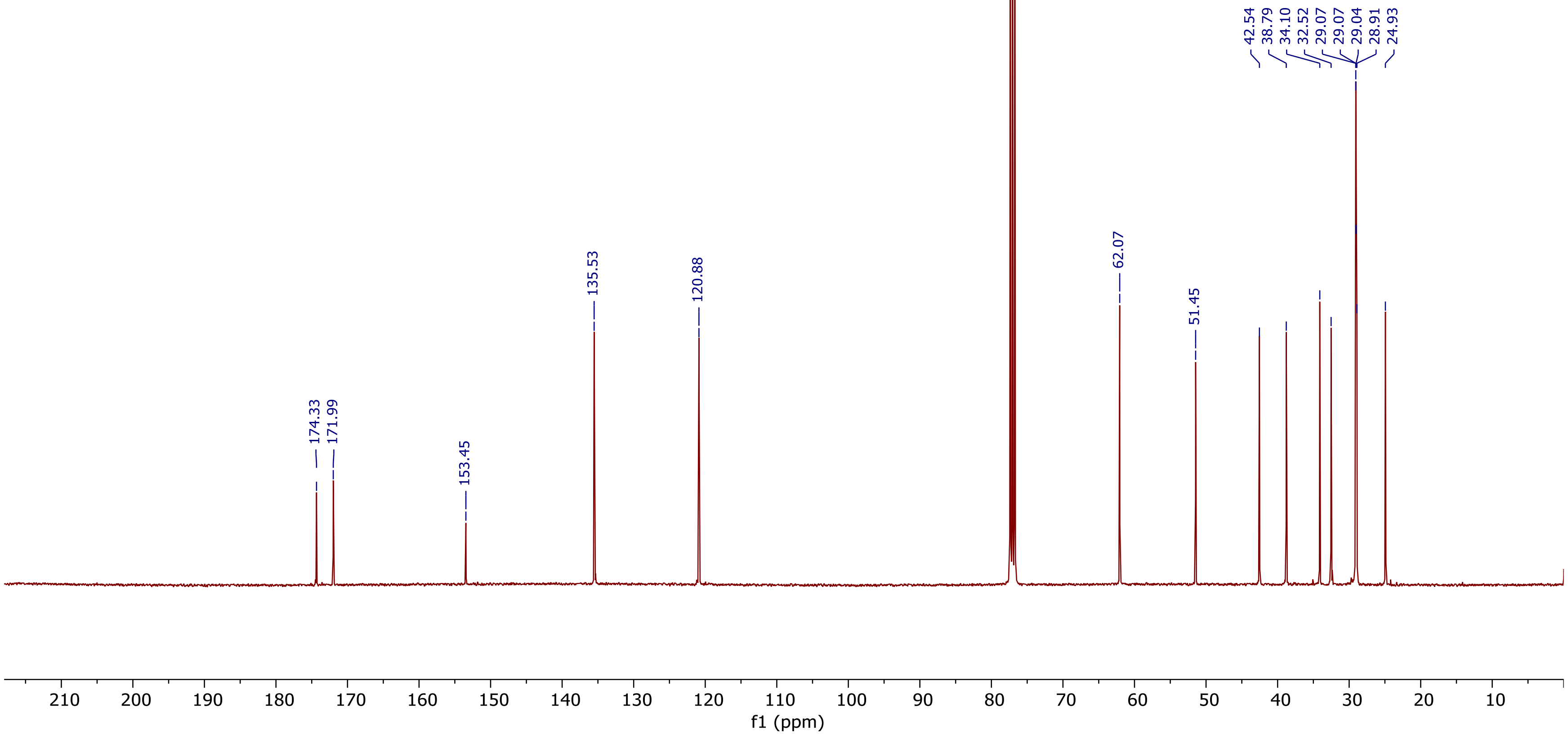

2D $^1$H -$^1$H COSY (400 MHz, $CDCl_3$)

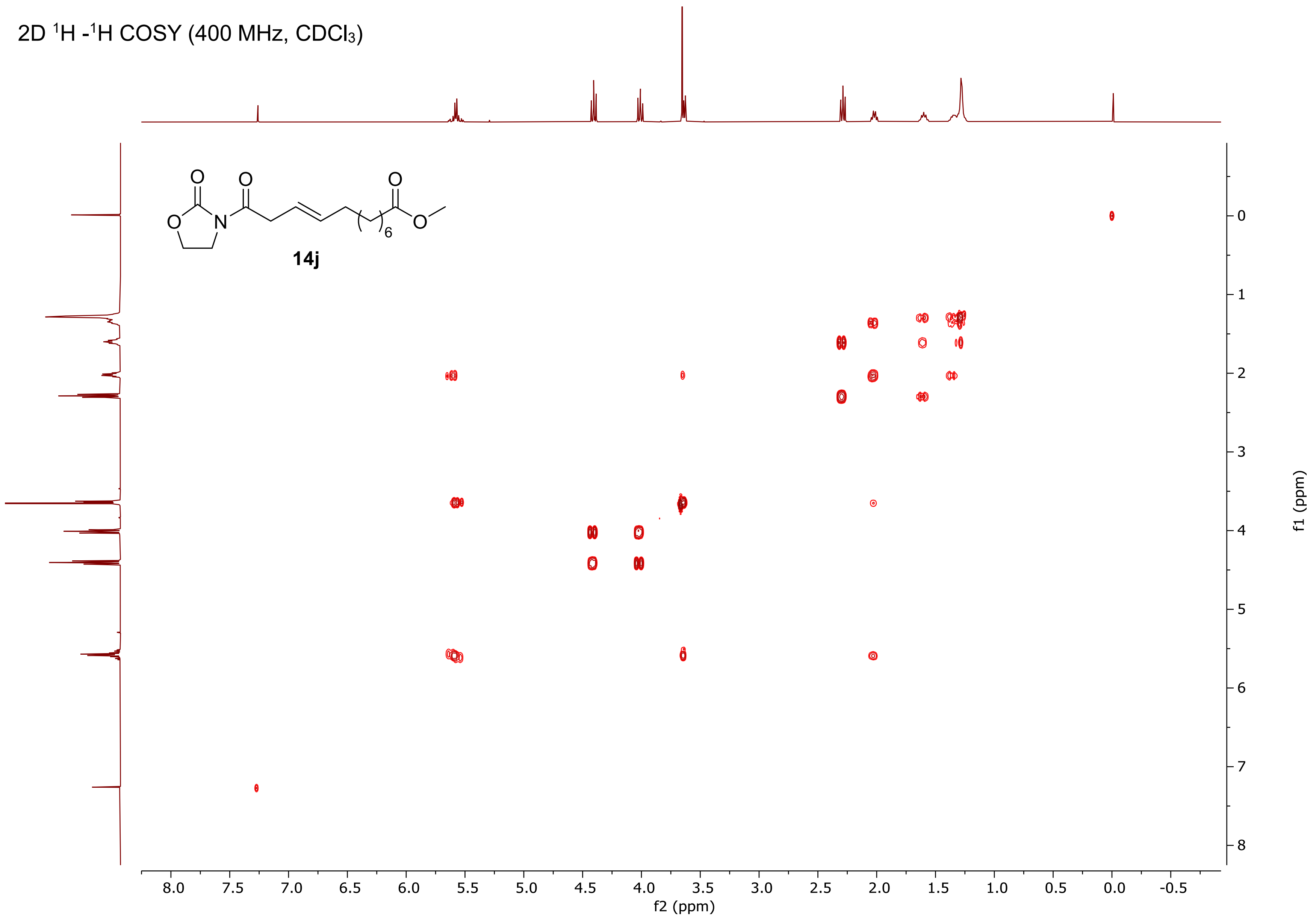

2D $^{1}H$ -$^{13}C$ HSQC (400 MHz, $CDCl_3$)

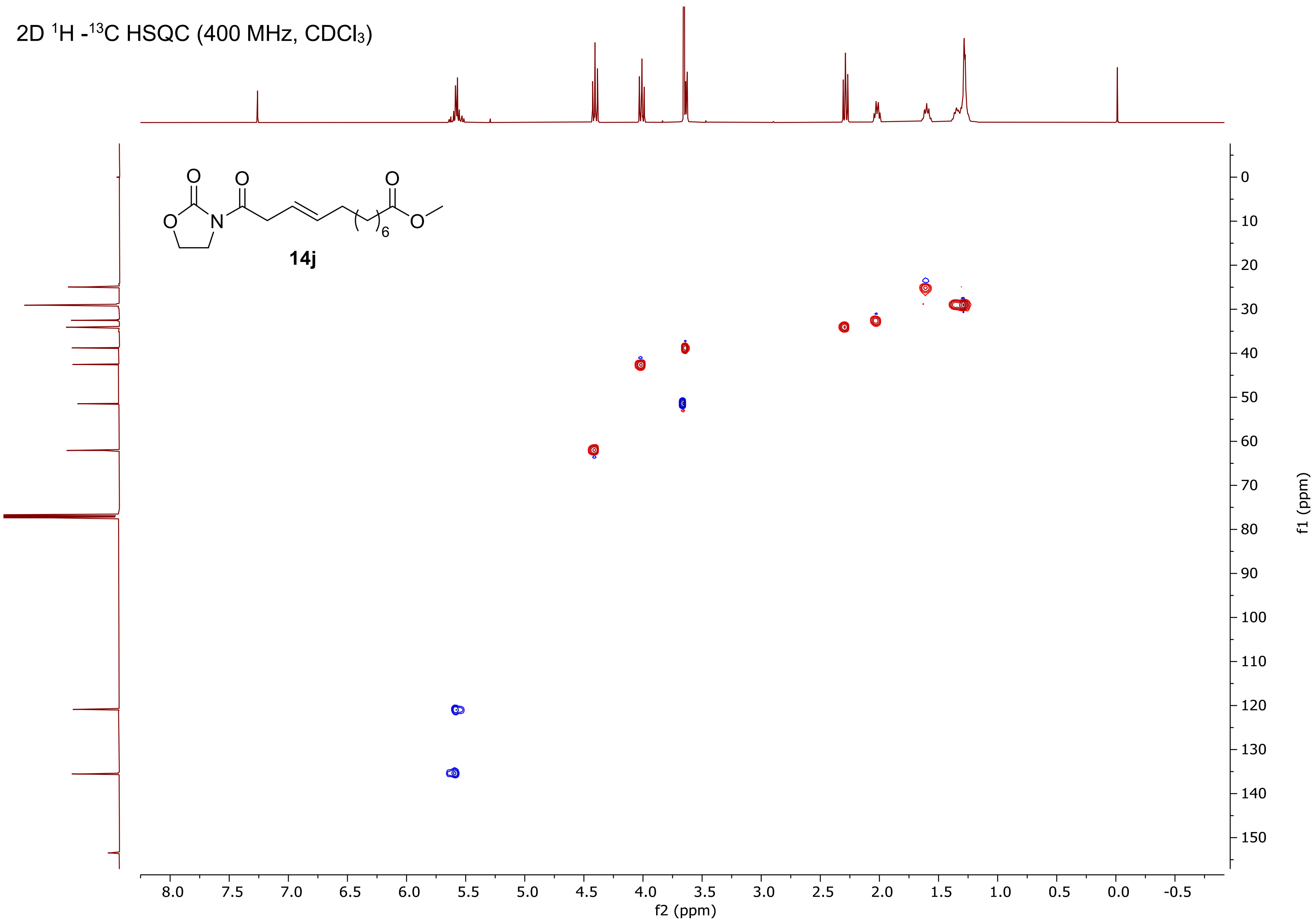

$^1H$ NMR (400 MHz, $CDCl_3$)

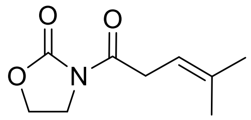

**14k**

10% of α-β unsaturated compound present

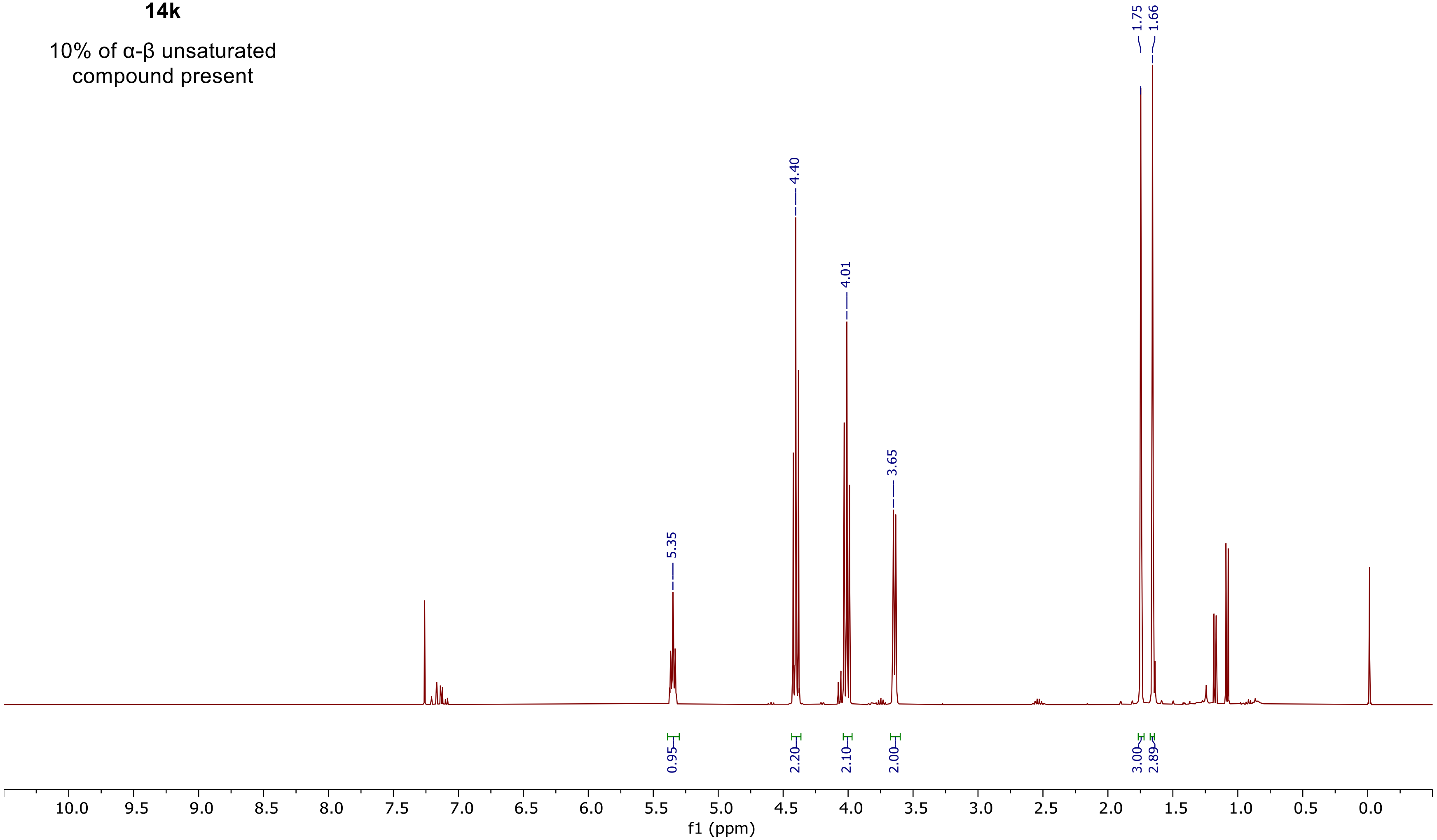

$^{13}C\{^{1}H\}$ NMR (101 MHz, $CDCl_3$)

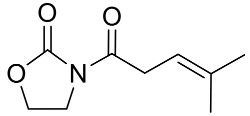

**14k**

10% of α-β unsaturated compound present

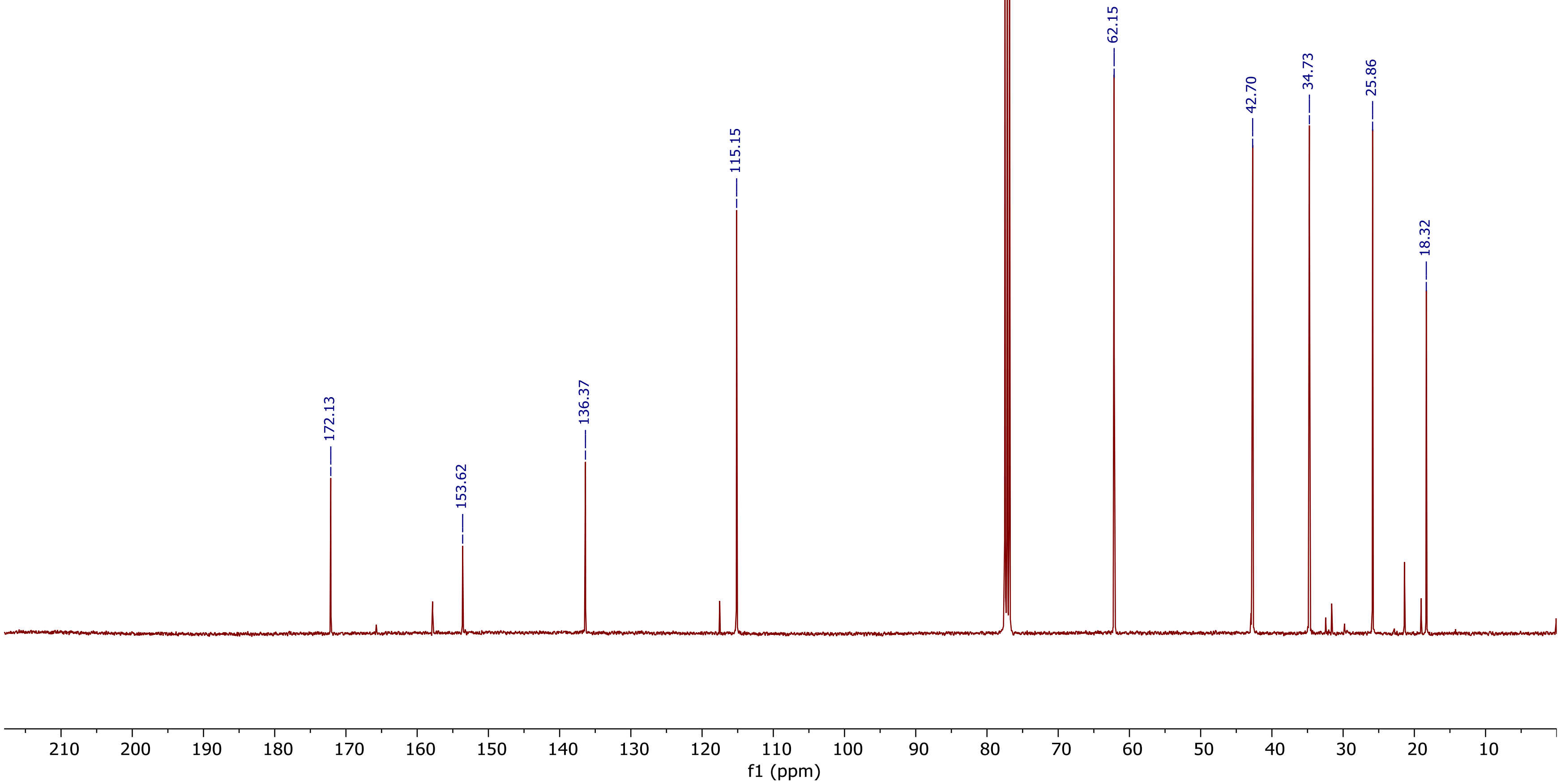

2D $^{1}H$ -$^{1}H$ COSY (400 MHz, $CDCl_3$)

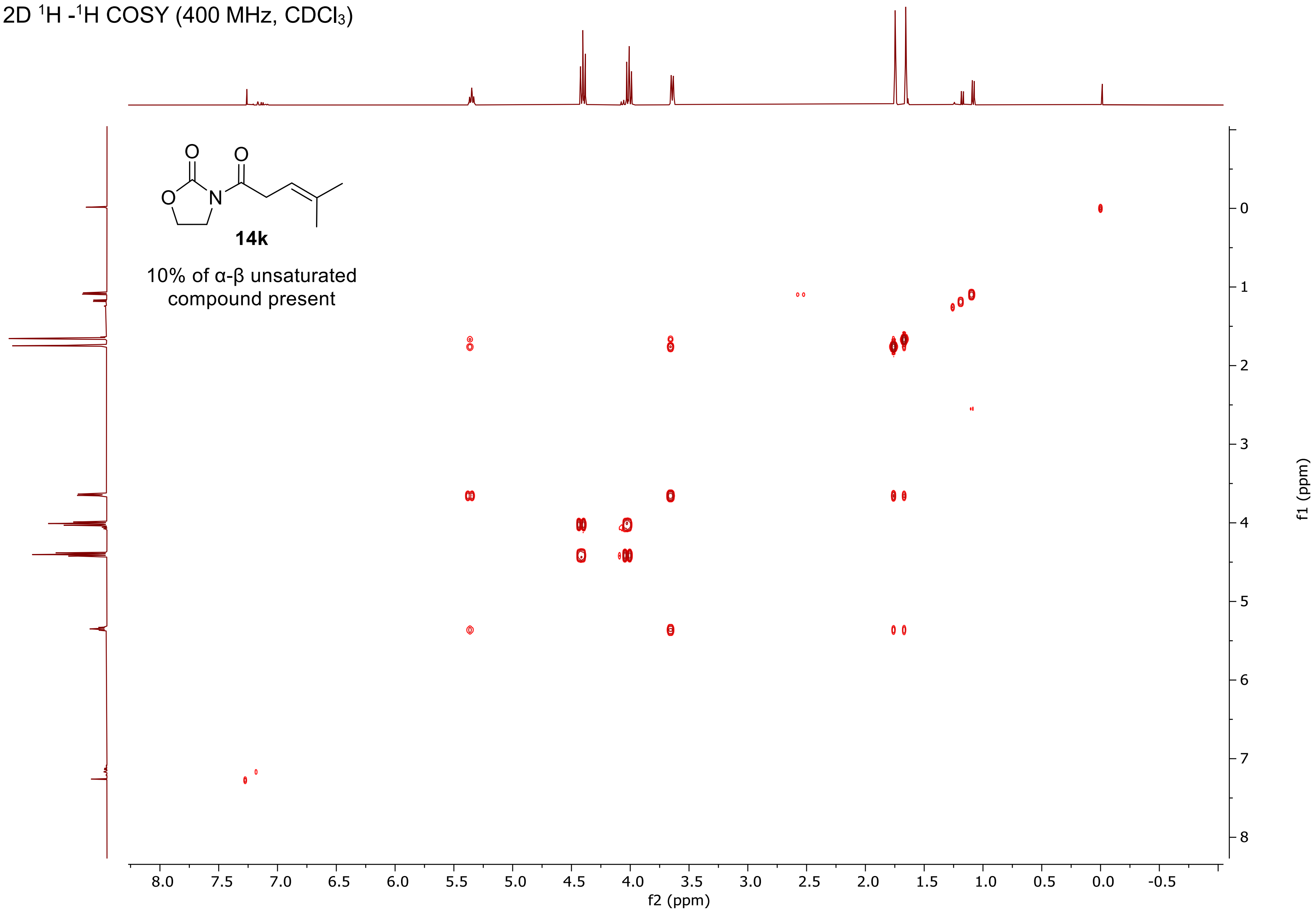

2D $^{1}H$ -$^{13}C$ HSQC (400 MHz, $CDCl_3$)

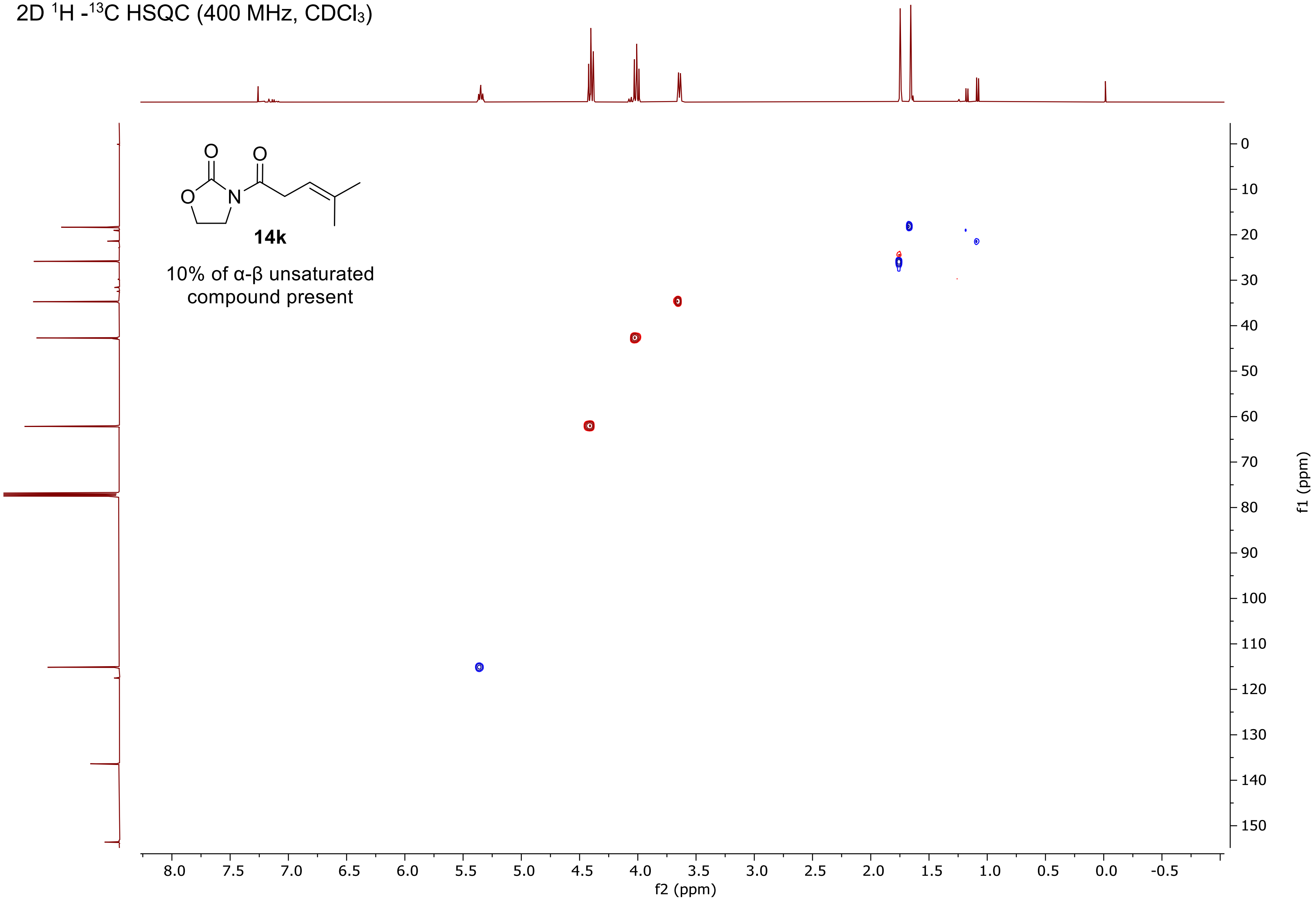

$^1H$ NMR (400 MHz, $CDCl_3$)

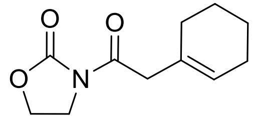

**14l**

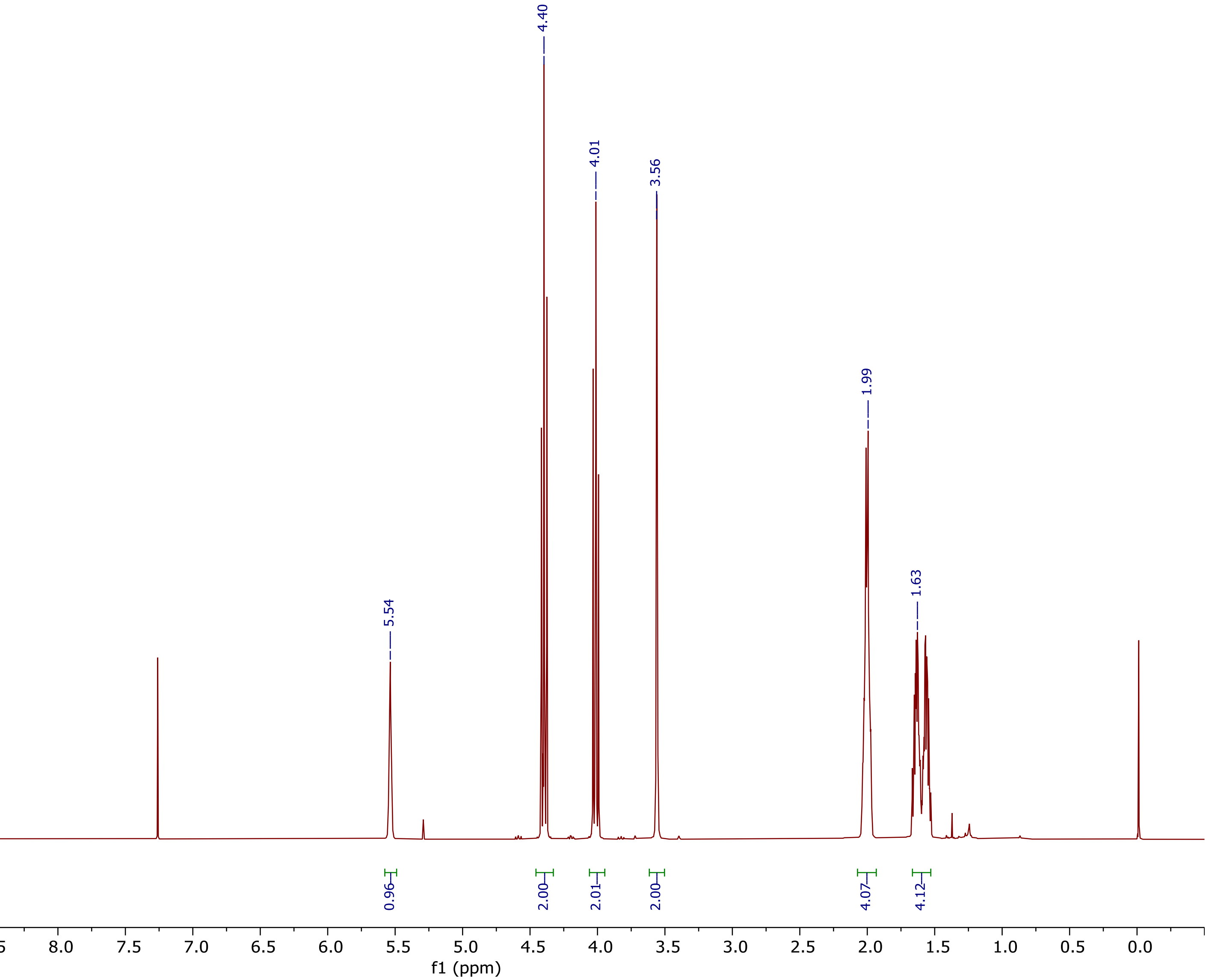

$^{13}C\{^1H\}$ NMR (101 MHz, $CDCl_3$)

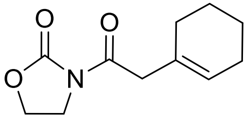


**14l**

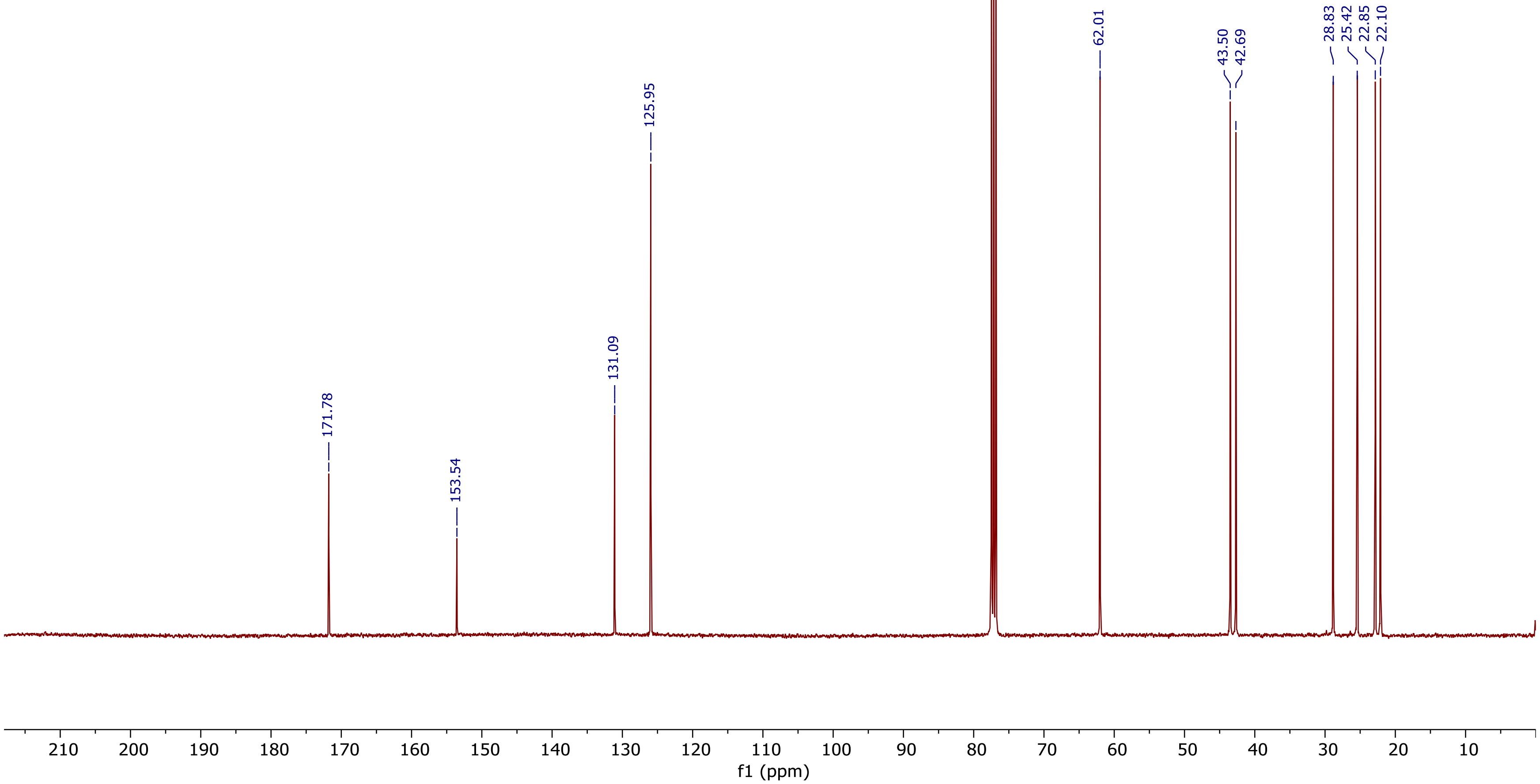

2D $^{1}H$ -$^{1}H$ COSY (400 MHz, $CDCl_3$)

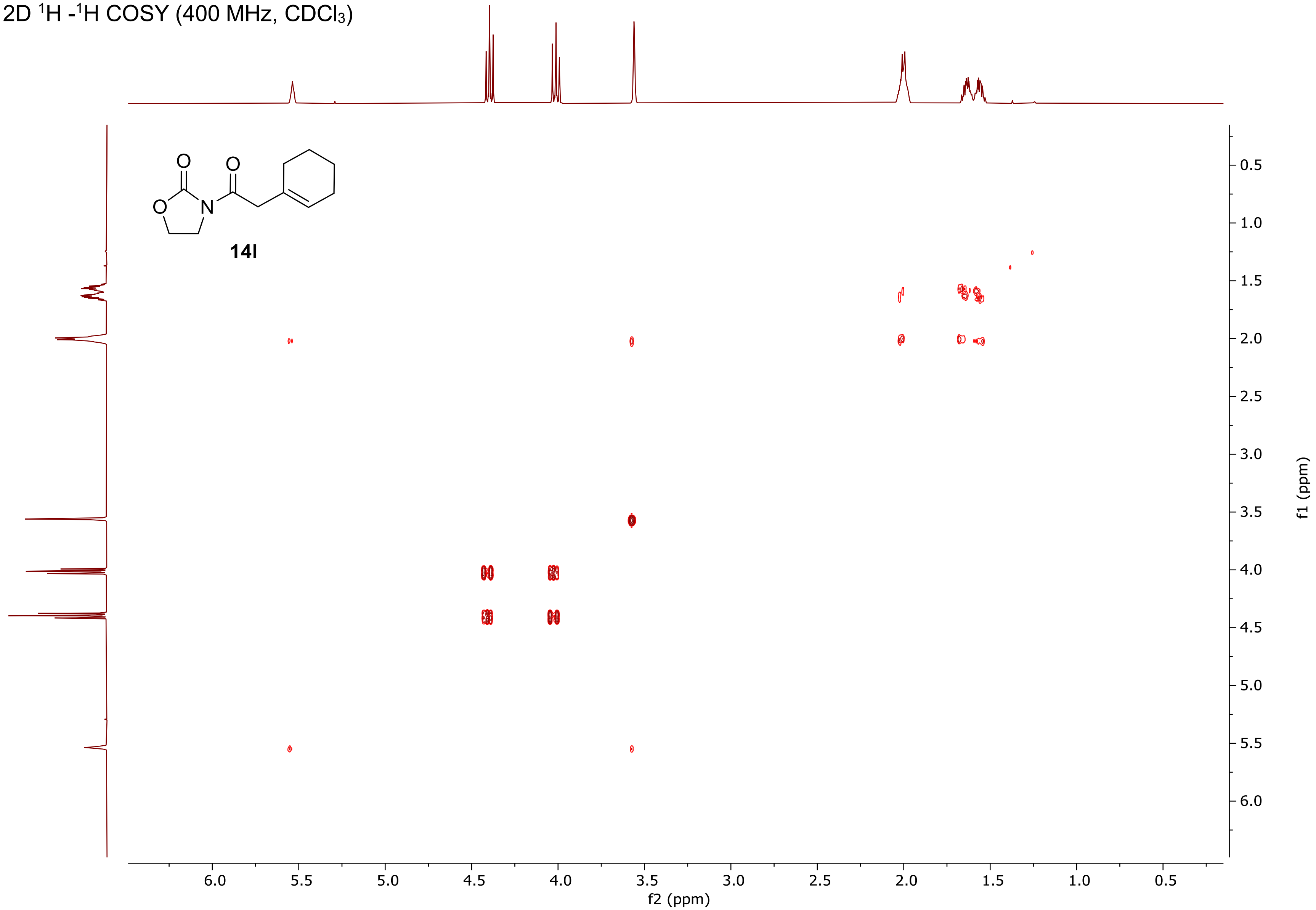

2D $^1H$ -$^{13}C$ HSQC (400 MHz, $CDCl_3$)

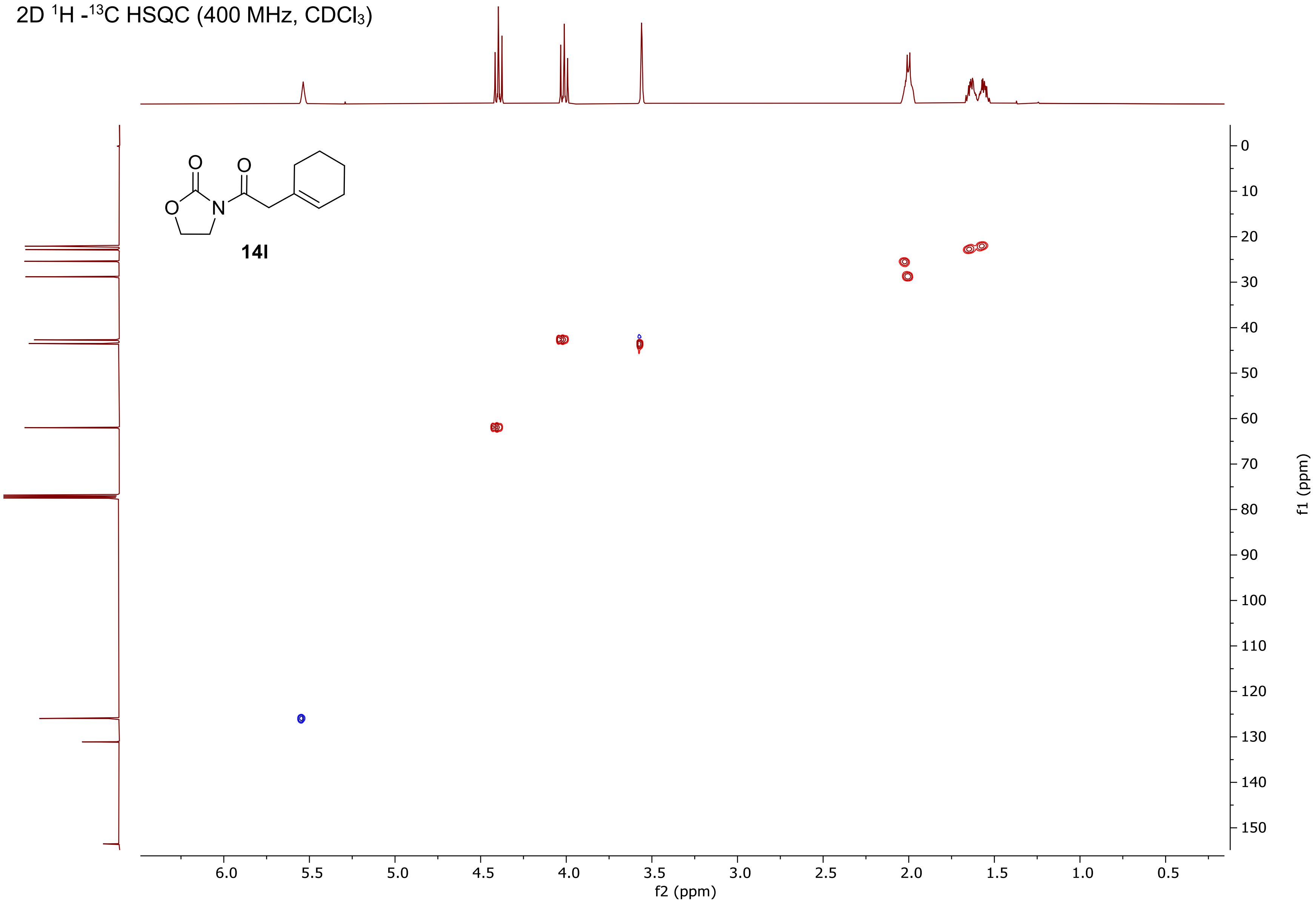

$^{1}H$ NMR (400 MHz, $CDCl_3$)

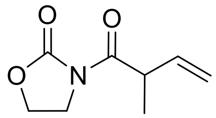

**14m**

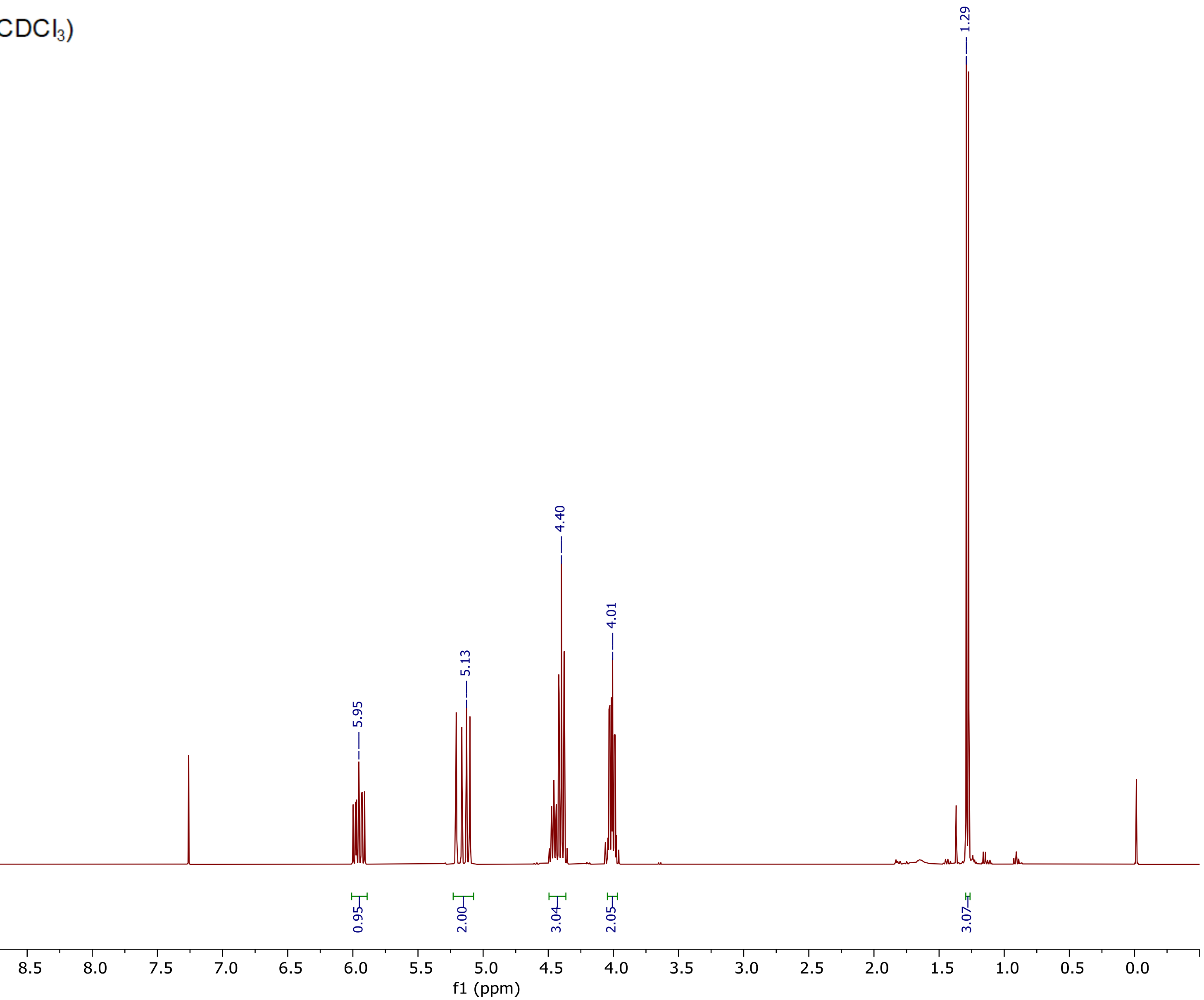

$^{13}C\{^1H\}$ NMR (101 MHz, $CDCl_3$)

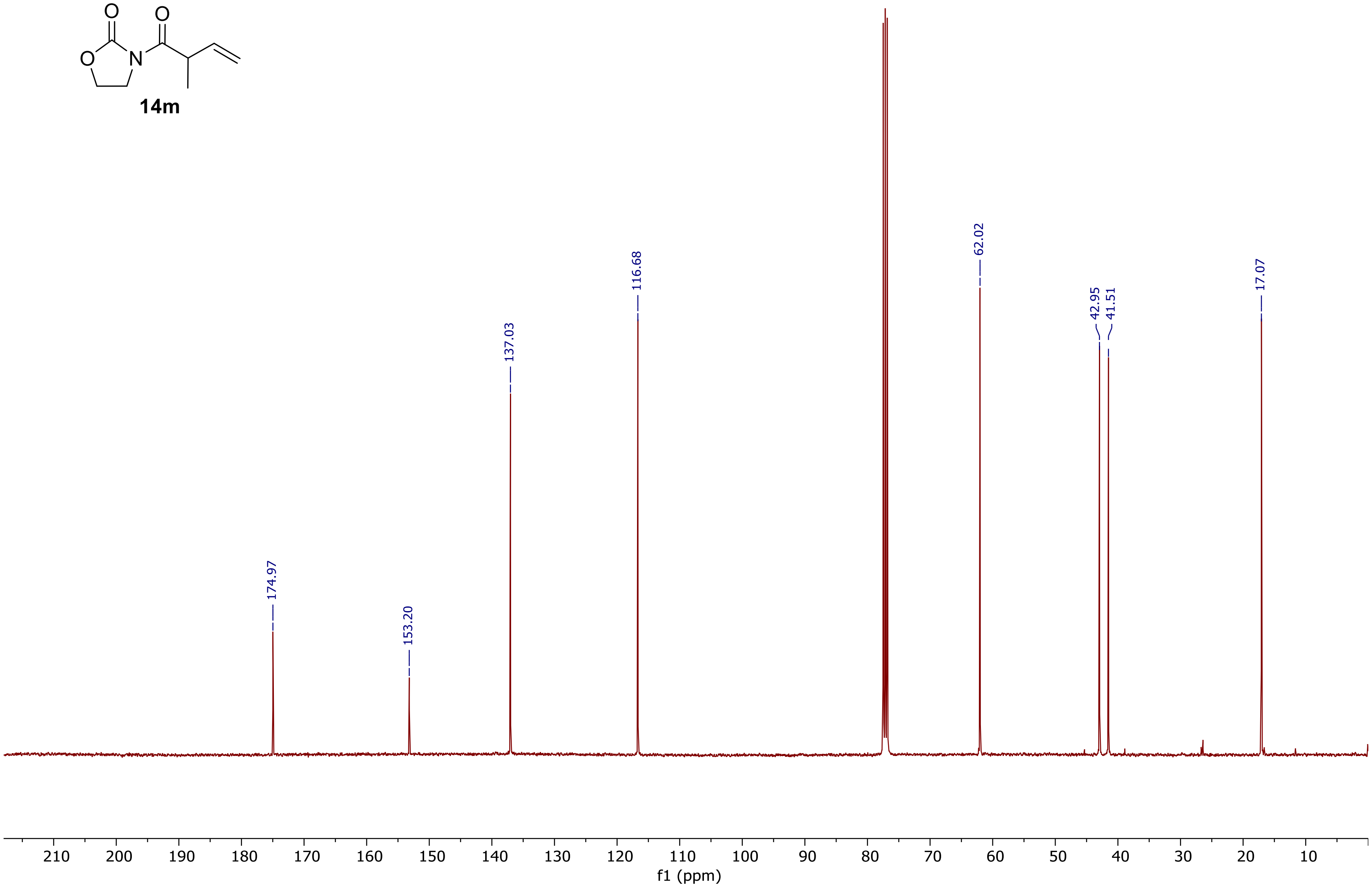

2D $^1$H -$^1$H COSY (400 MHz, $CDCl_3$)

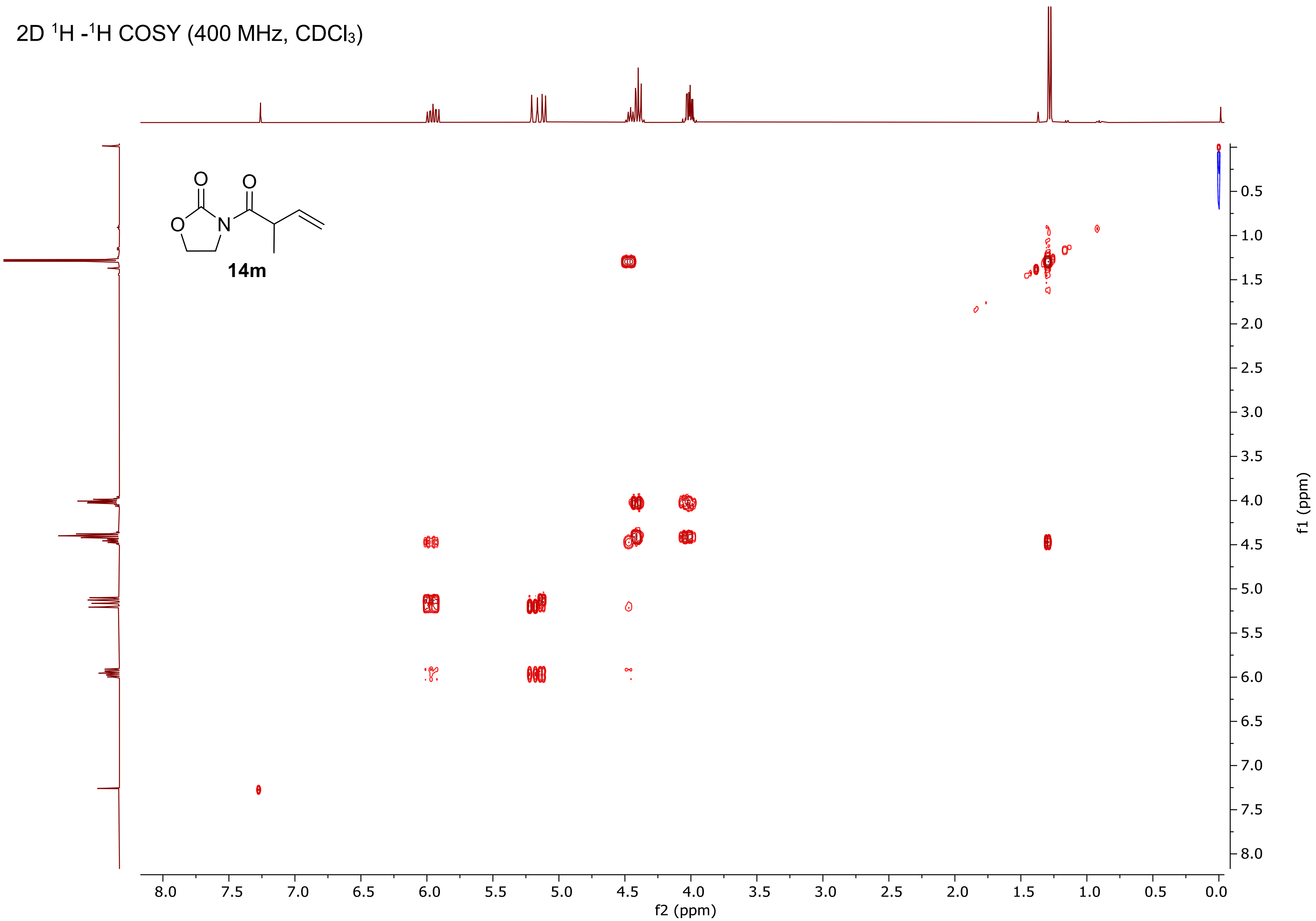

2D $^1$H -$^{13}$C HSQC (400 MHz, $CDCl_3$)

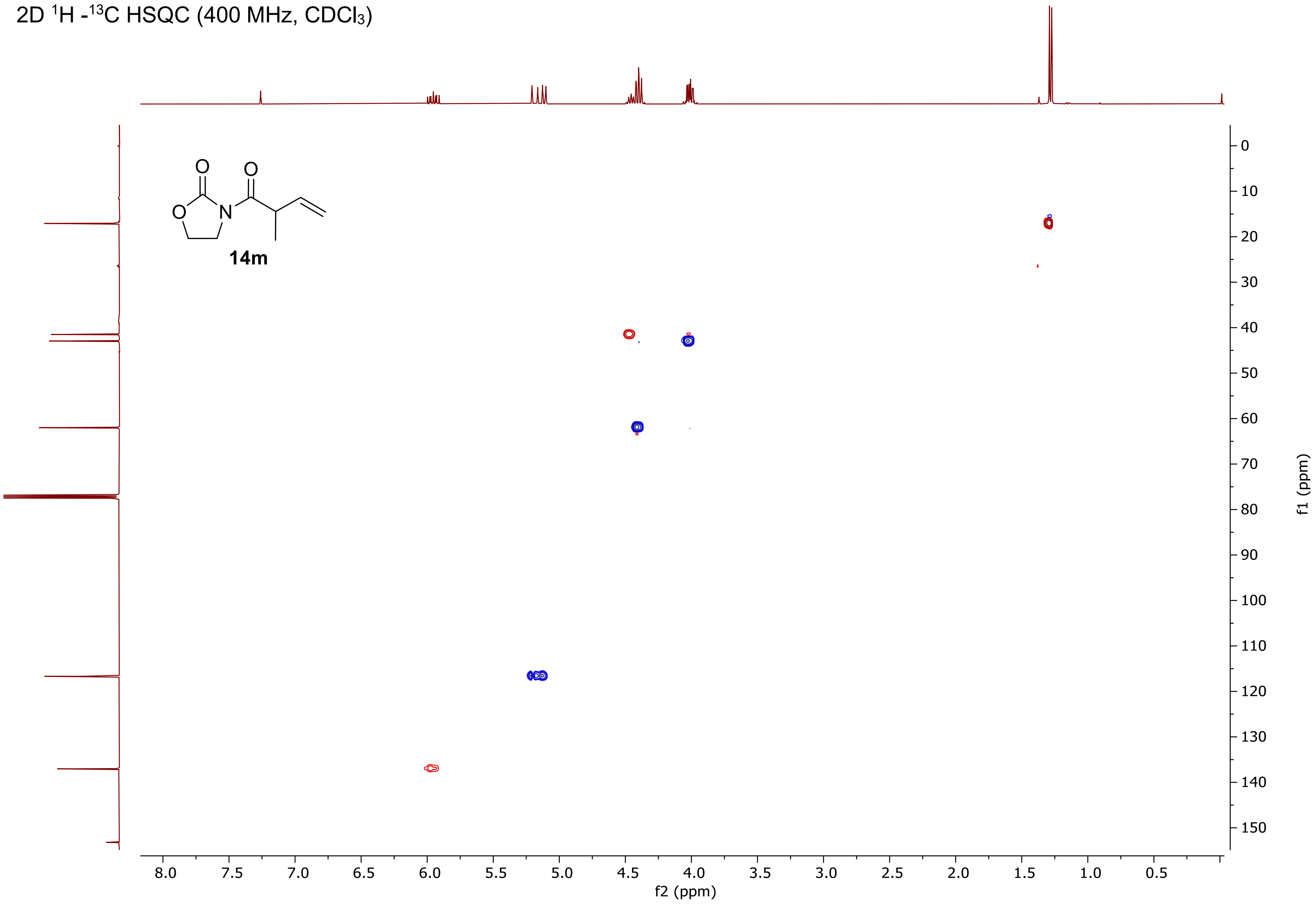

$^{1}$H NMR (500 MHz, $CDCl_3$)

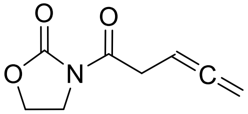


**14n**

38% of α,β-γ,δ unsaturated compound present

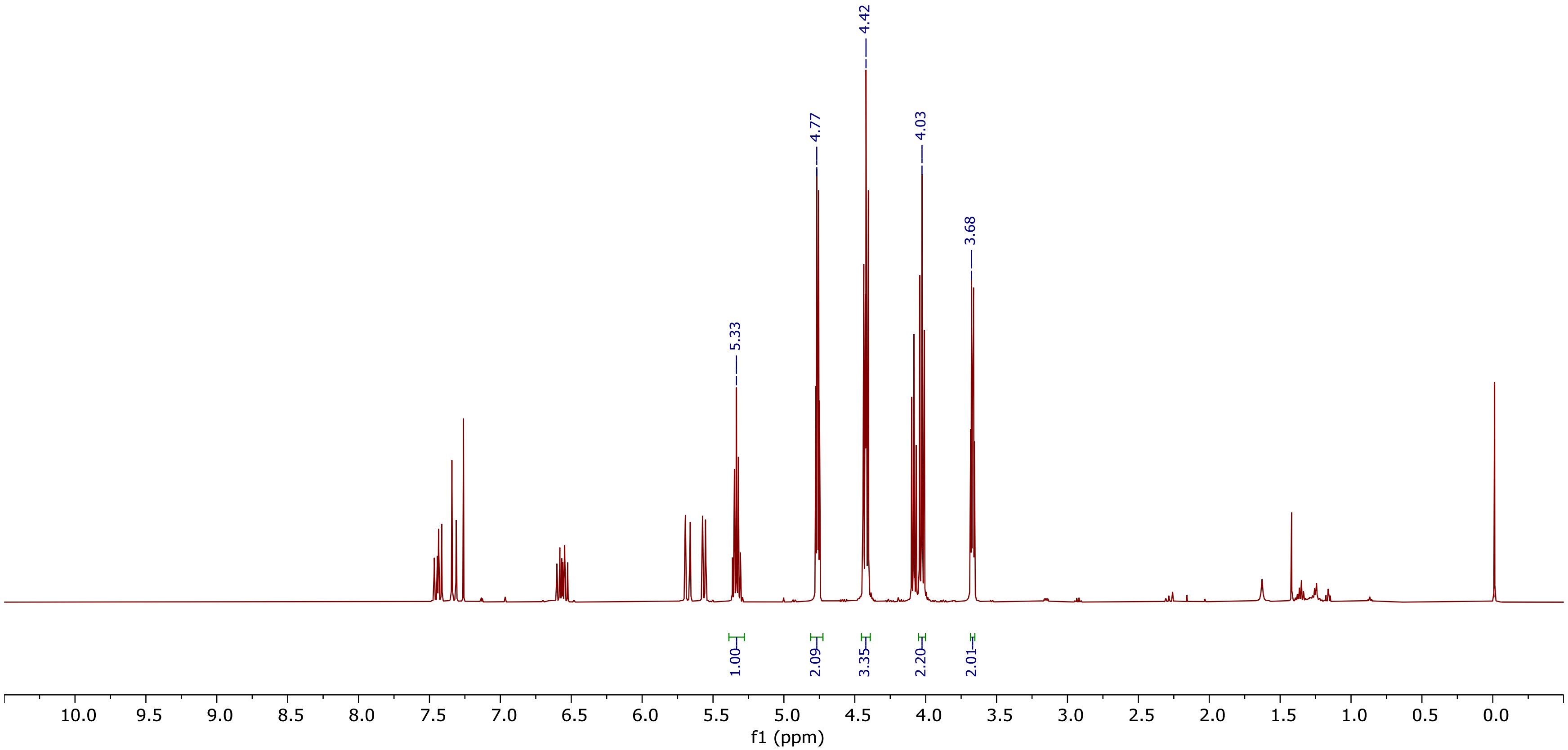

$^{13}C\{^{1}H\}$ NMR (126 MHz, $CDCl_3$)

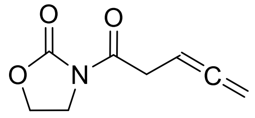

**14n**

38% of α,β-γ,δ unsaturated compound present

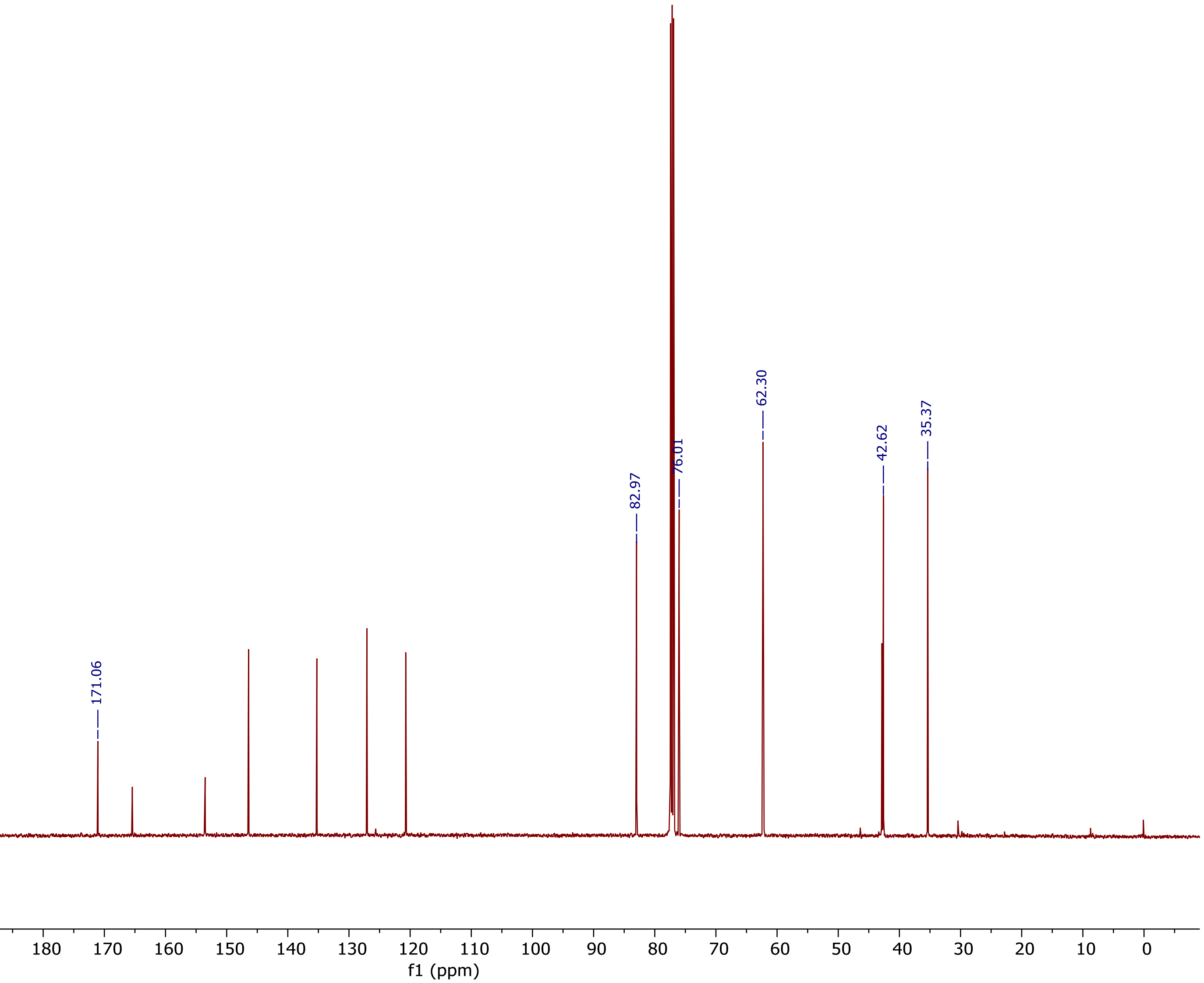

2D $^1H$ -$^1H$ COSY (500 MHz, $CDCl_3$)

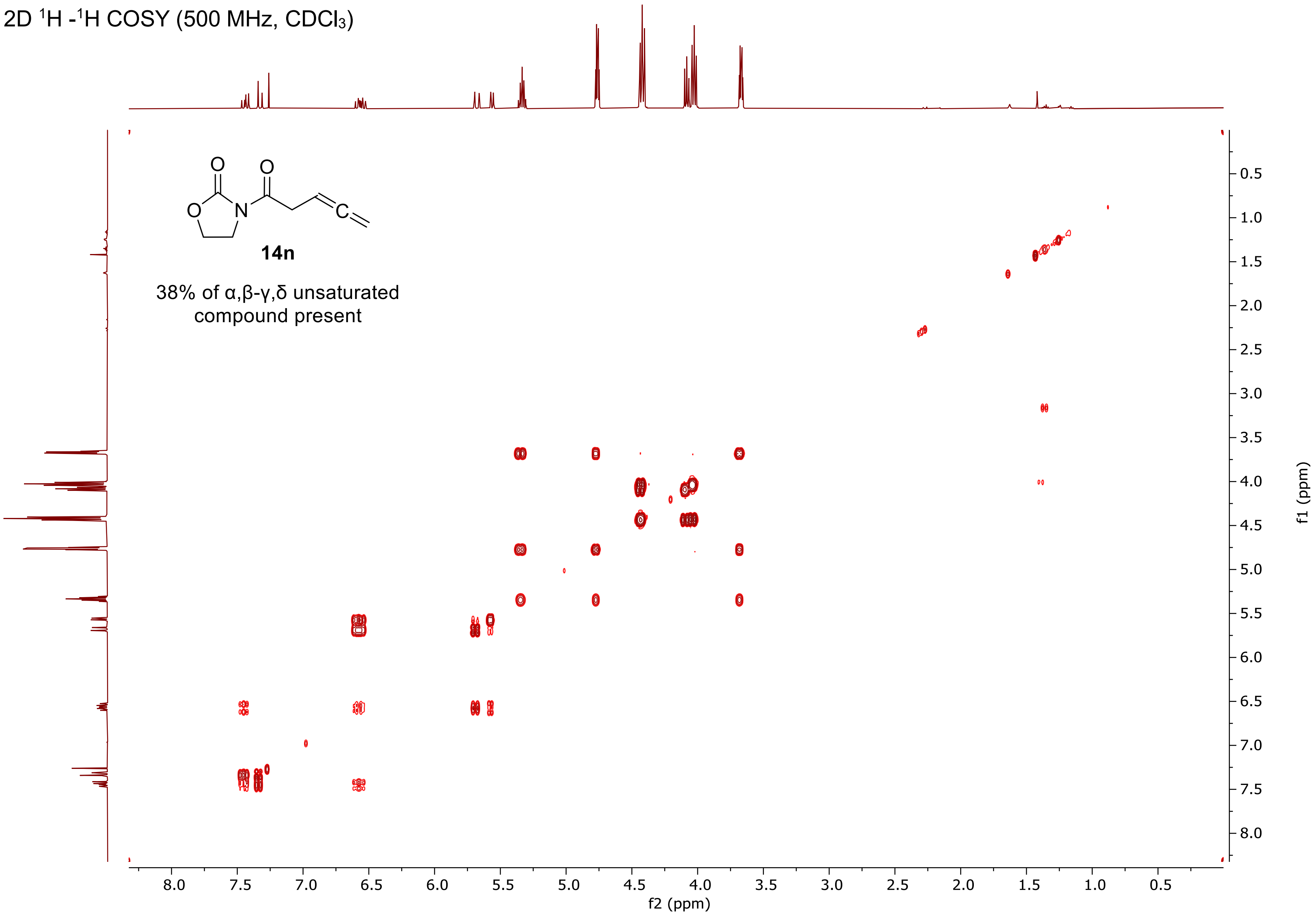

2D $^{1}H$ -$^{13}C$ HSQC (500 MHz, $CDCl_3$)

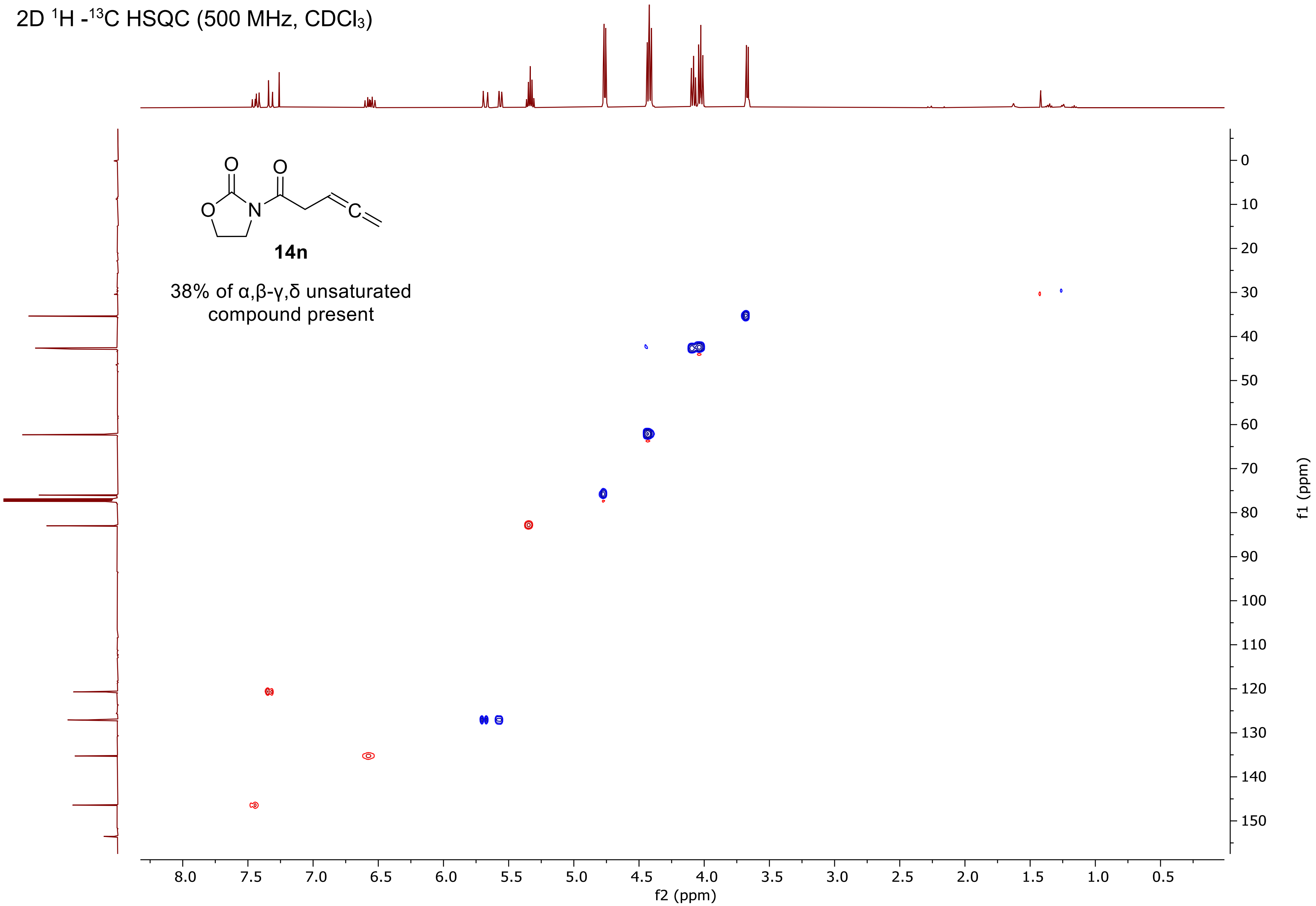

[1]H NMR (400 MHz, $CDCl_3$)

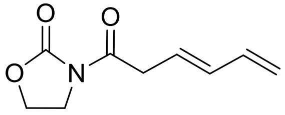

**14o**

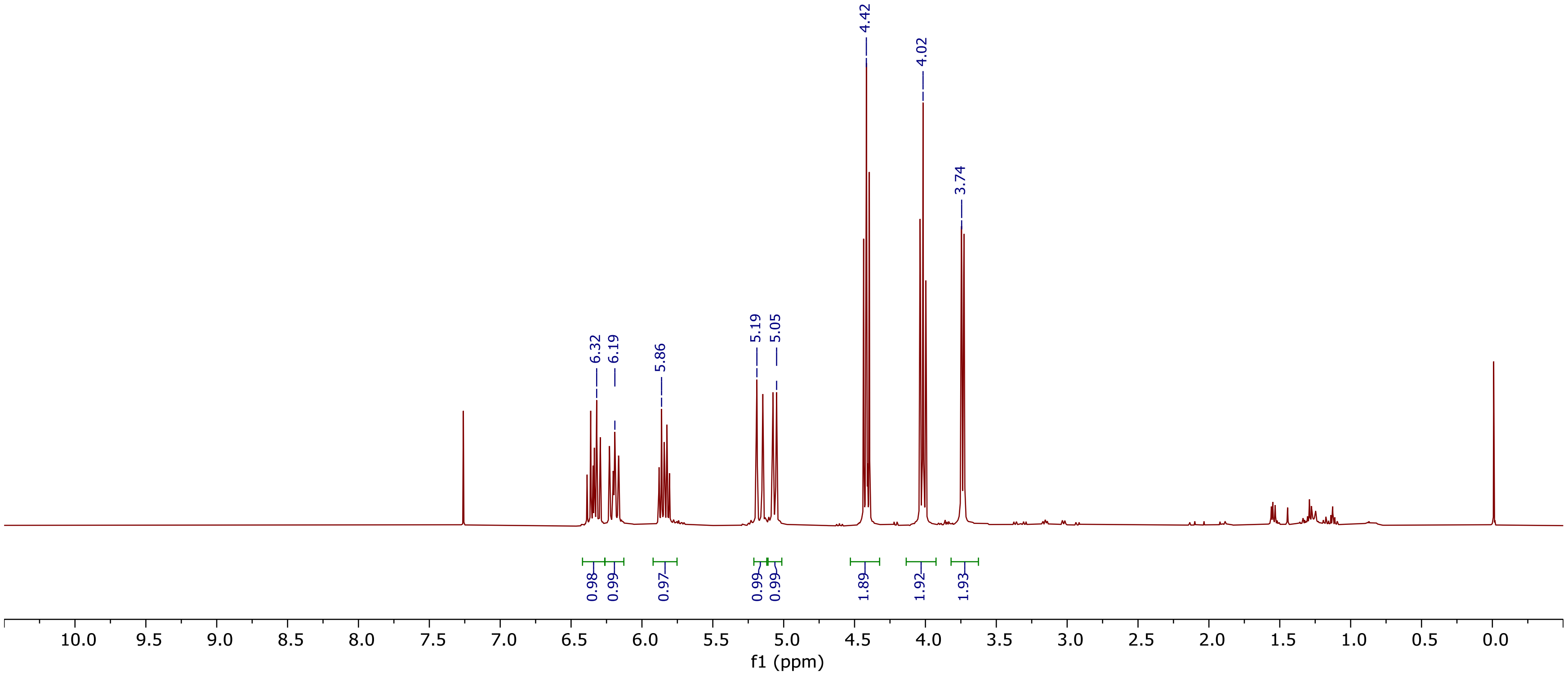

$^{13}C\{^{1}H\}$ NMR (101 MHz, $CDCl_3$)

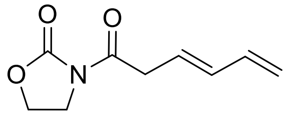

**14o**

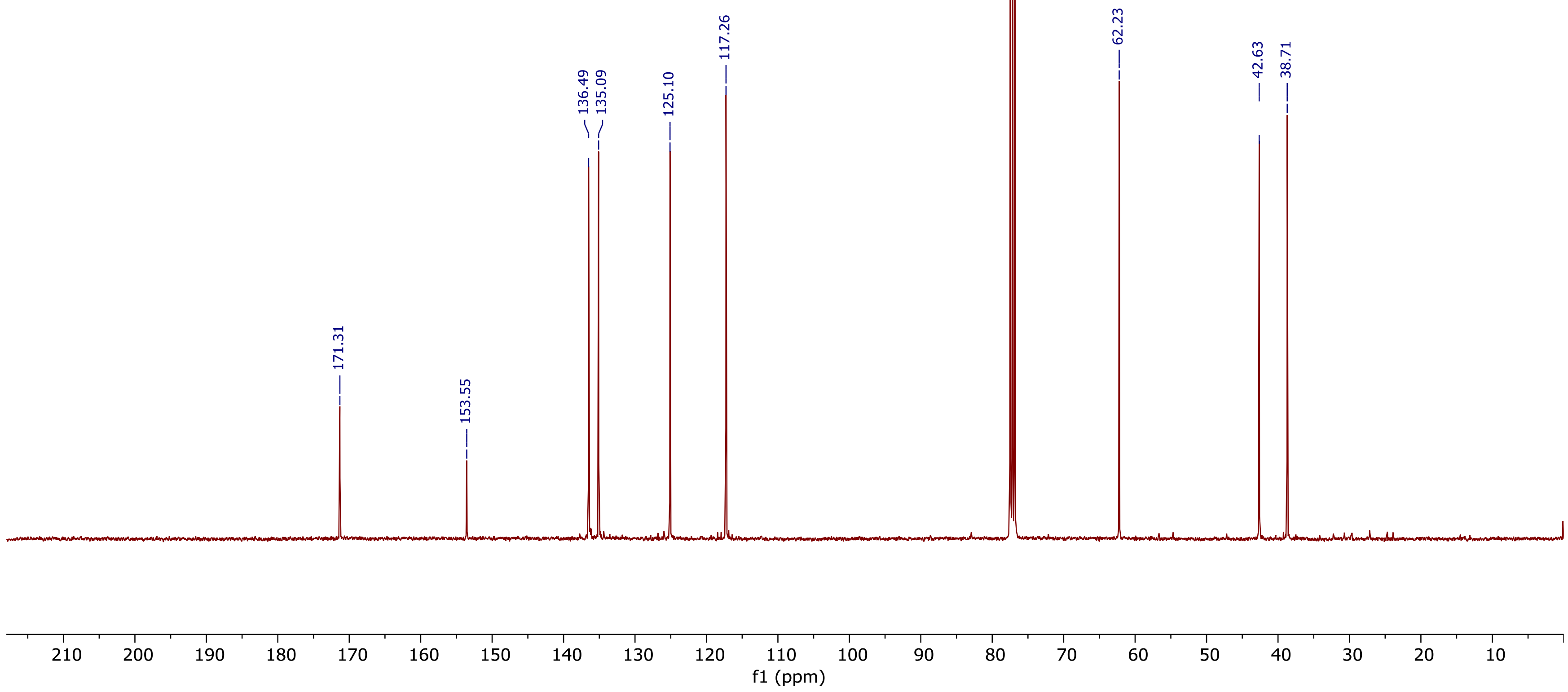

2D $^{1}H$ -$^{1}H$ COSY (400 MHz, $CDCl_3$)

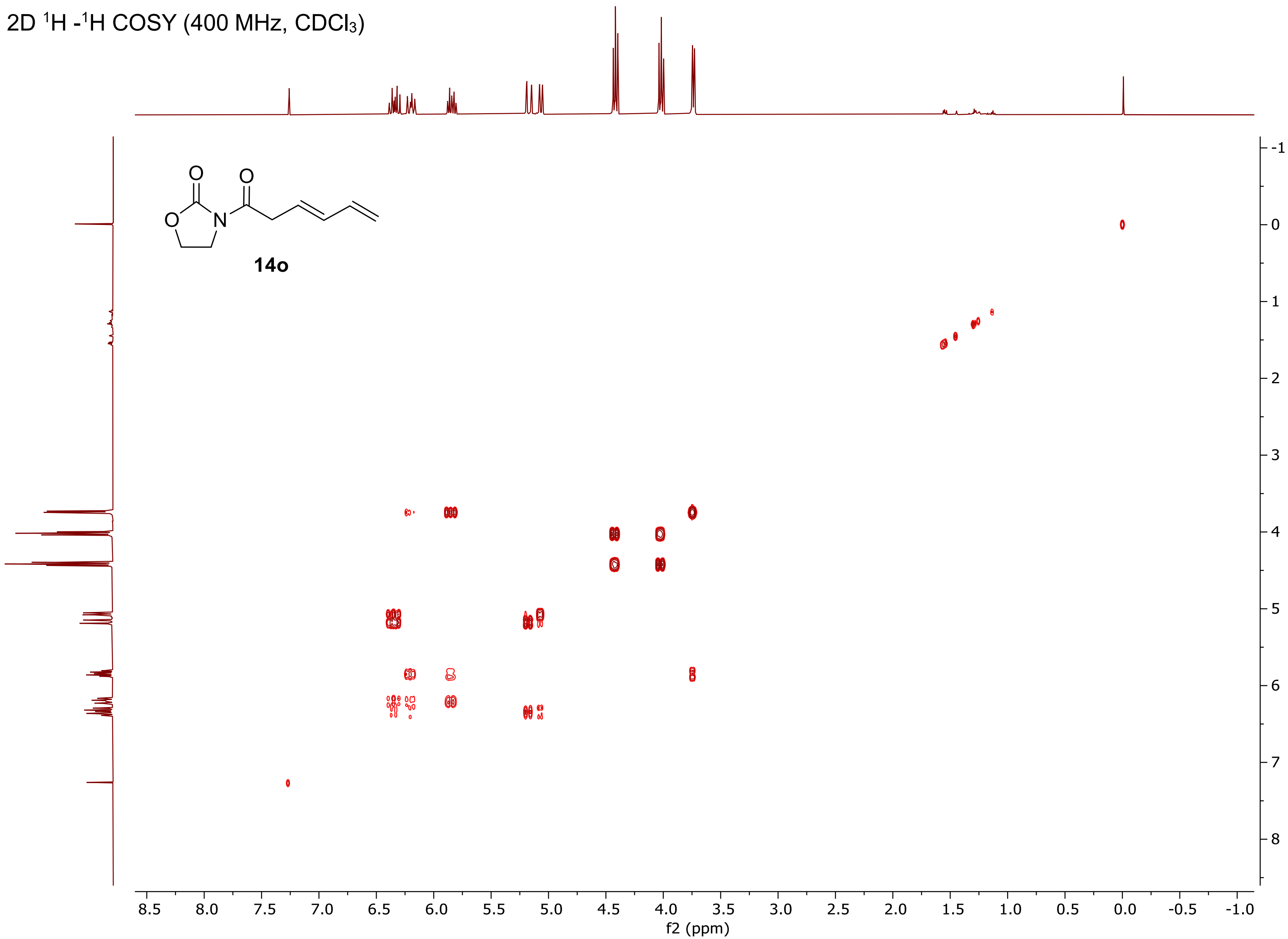

2D ¹H -¹³C HSQC (400 MHz, $CDCl_3$)

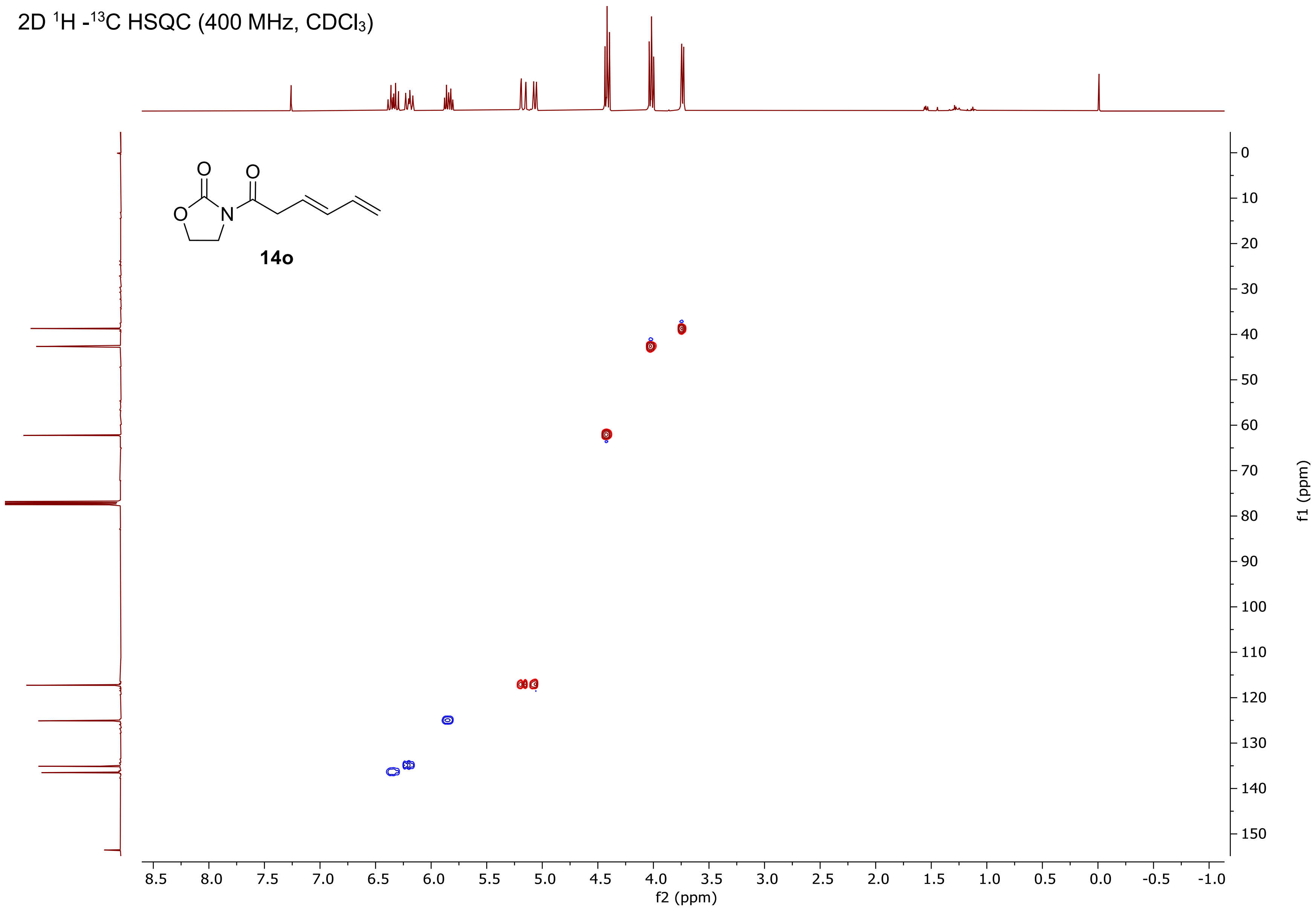

$^{1}H$ NMR (400 MHz, $CDCl_3$)

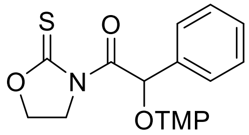


**15a**

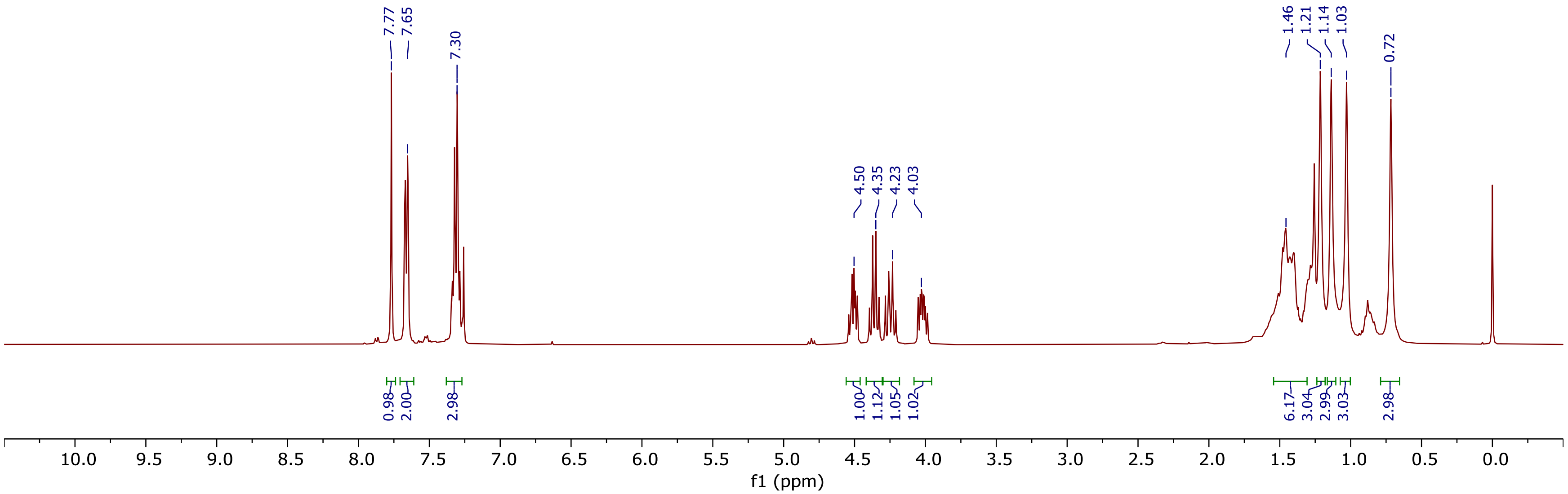

$^{13}C\{^{1}H\}$ NMR (101 MHz, $CDCl_3$)

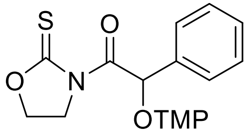


**15a**

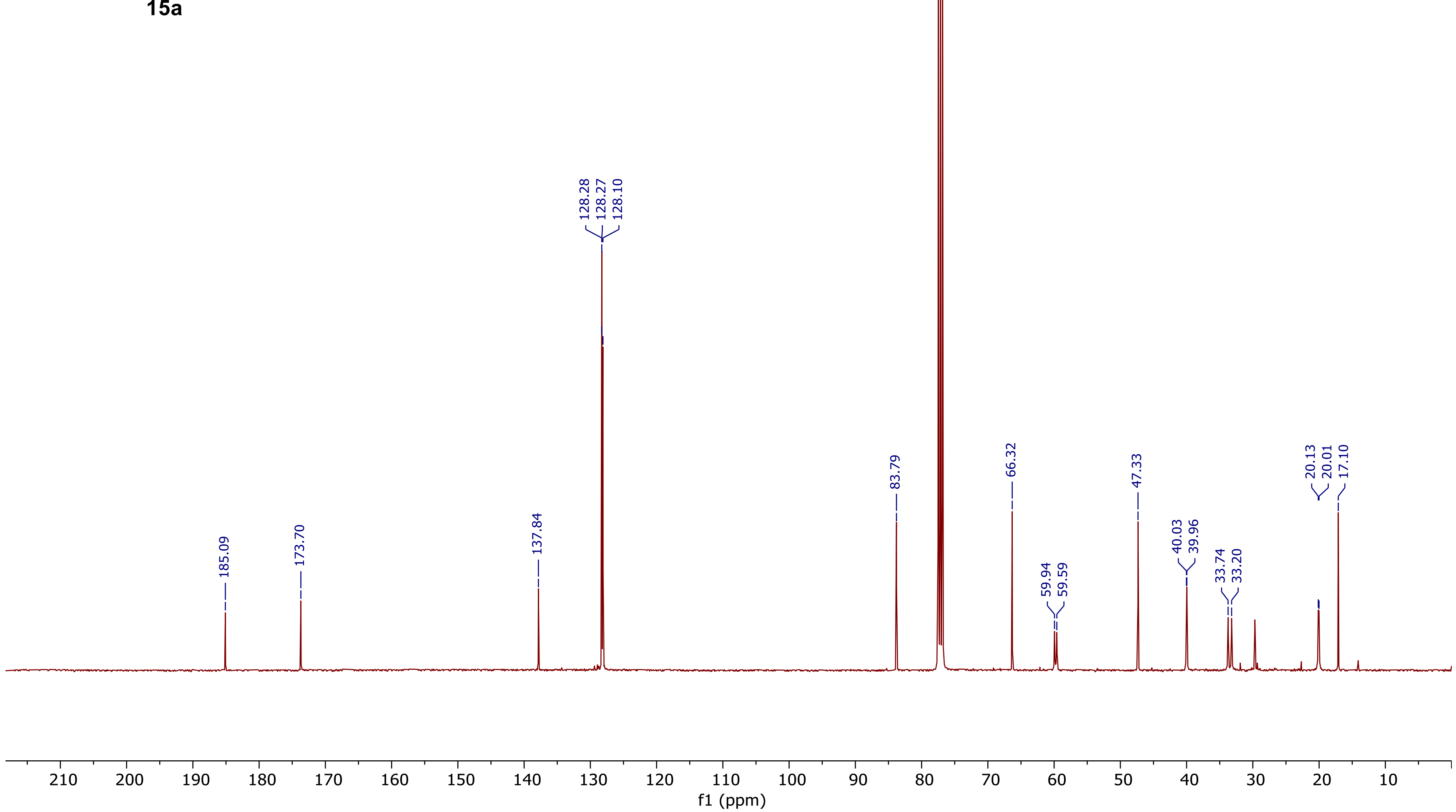

2D $^{1}H$ -$^{1}H$ COSY (400 MHz, $CDCl_3$)

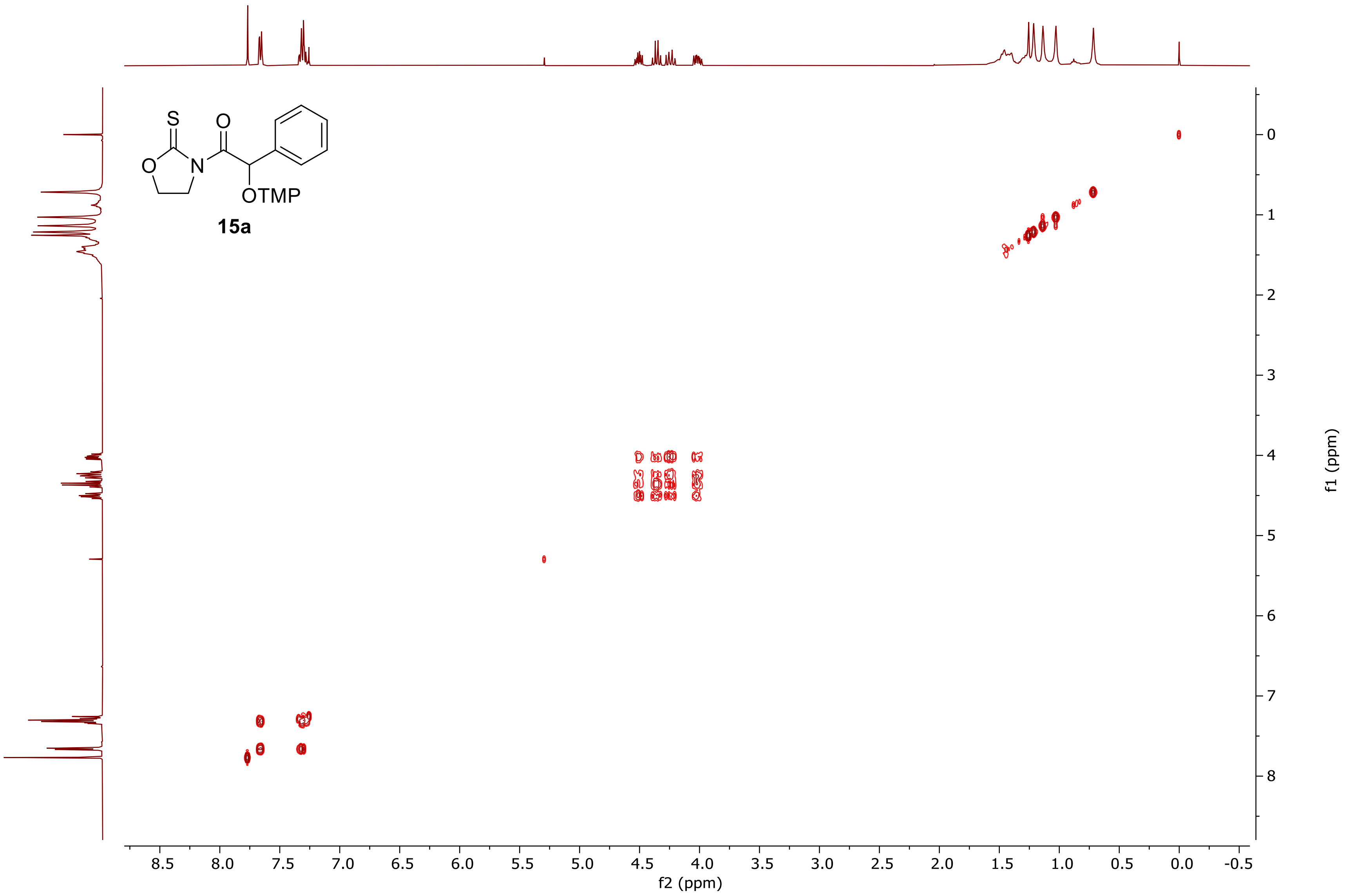

2D $^{1}H$ -$^{13}C$ HSQC (400 MHz, $CDCl_3$)

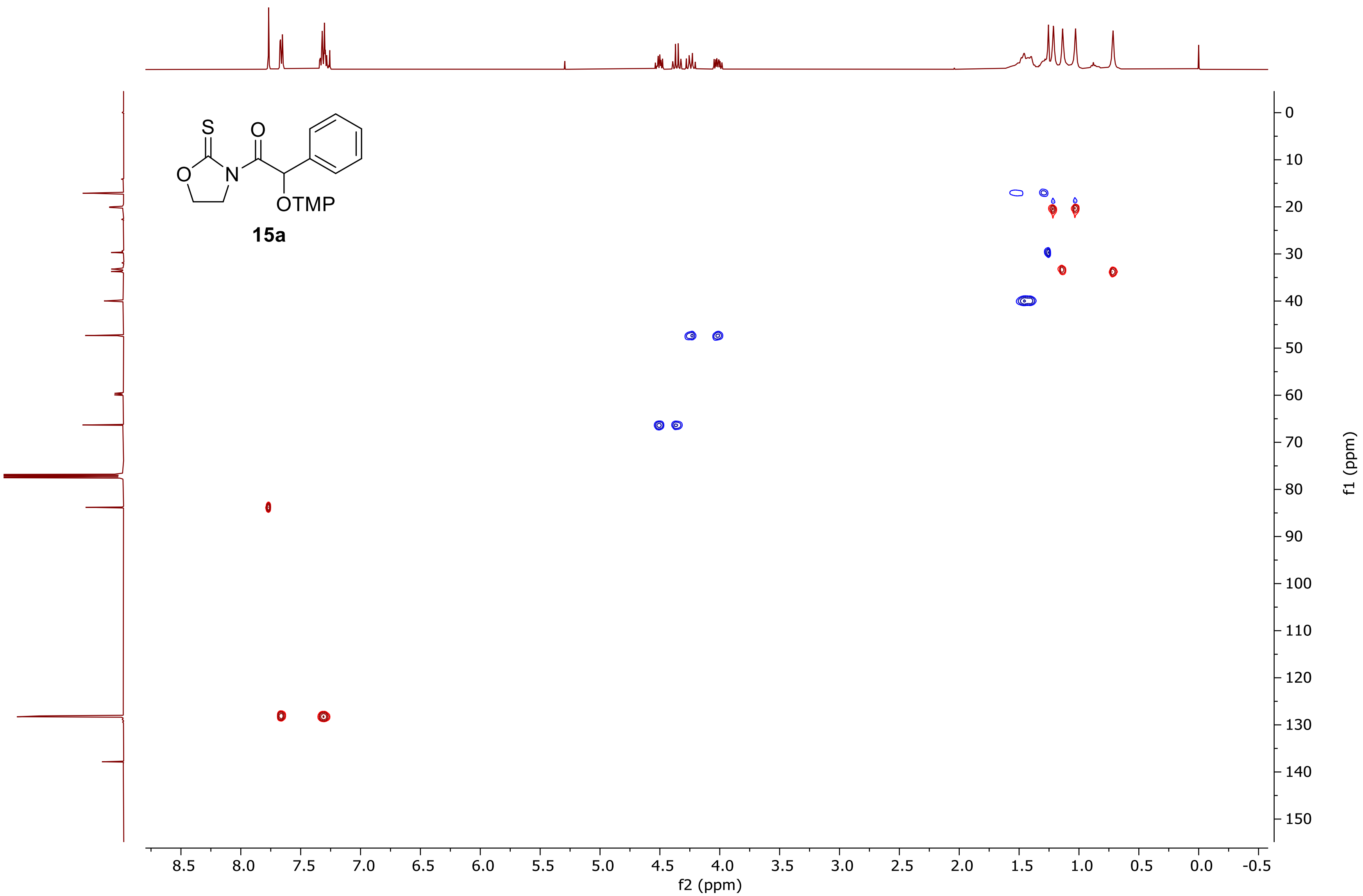

$^{1}$H NMR (400 MHz, $CDCl_3$)

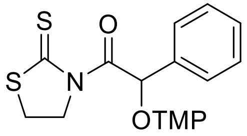


**16a**

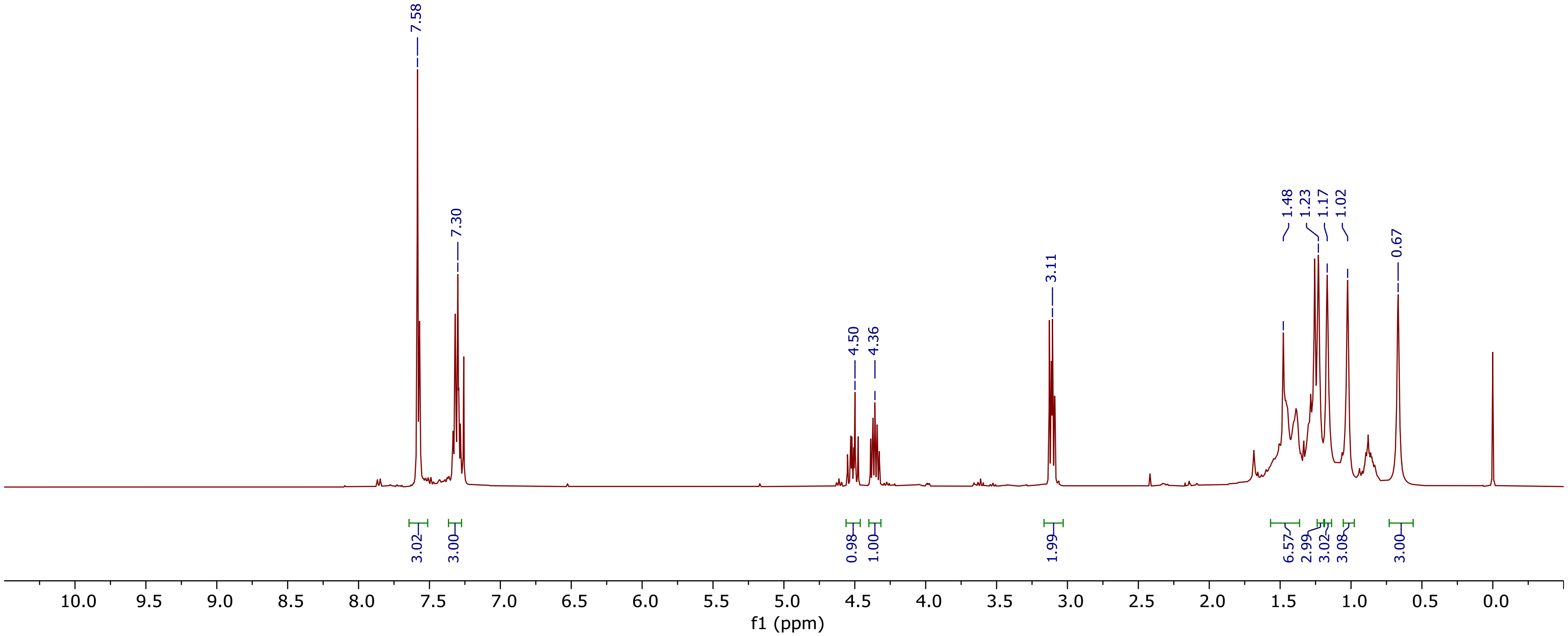

$^{13}C\{^{1}H\}$ NMR (101 MHz, $CDCl_3$)

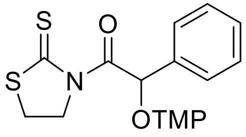

**16a**

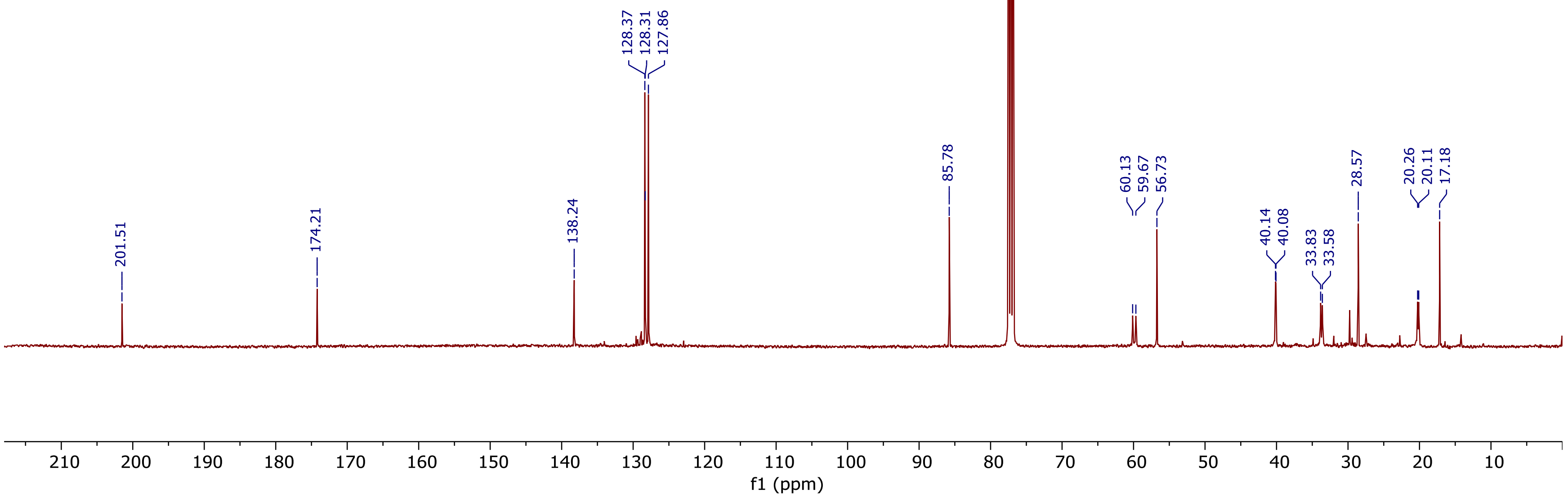

2D $^{1}H$ -$^{1}H$ COSY (400 MHz, $CDCl_3$)

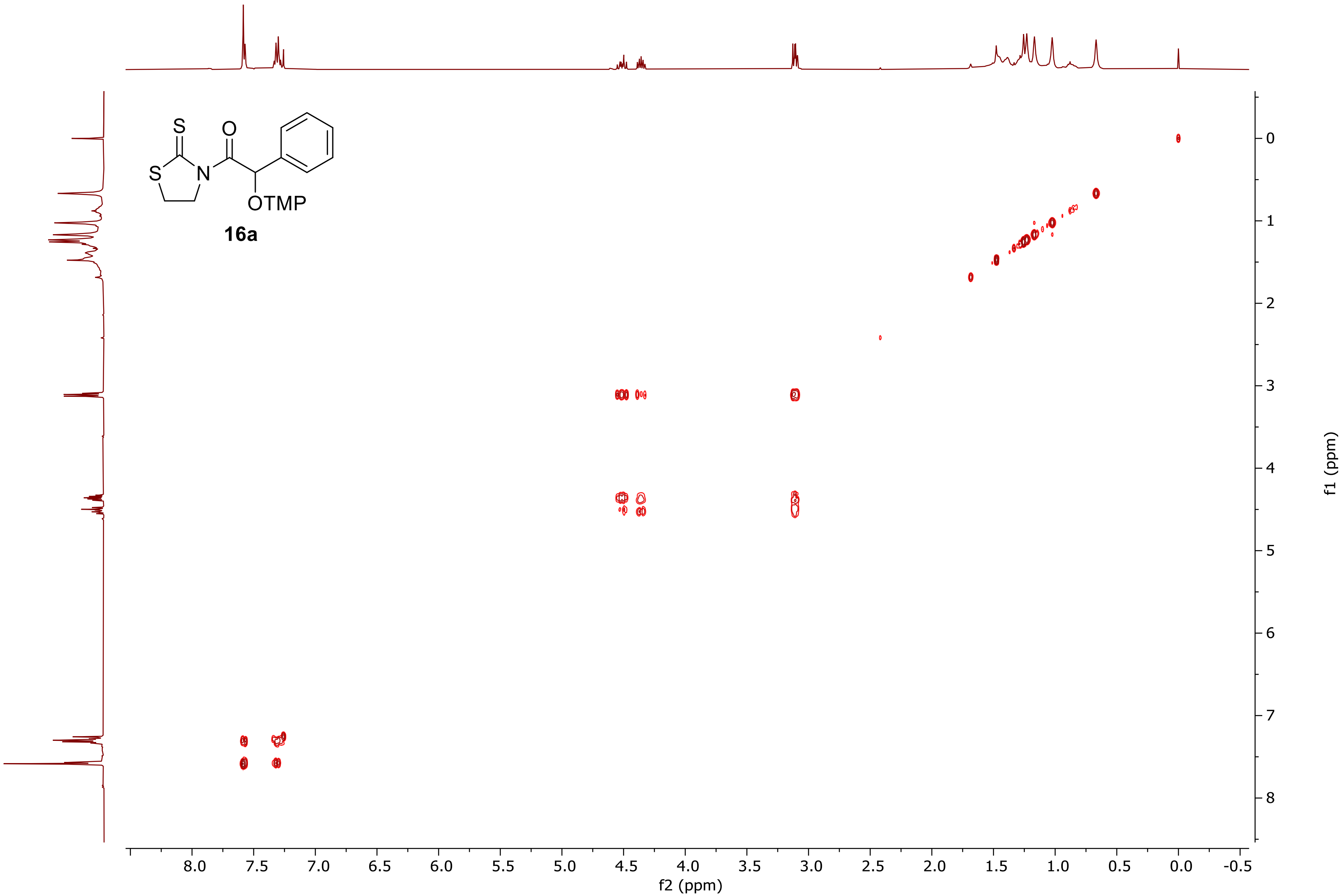

2D $^{1}H$ -$^{13}C$ HSQC (400 MHz, $CDCl_3$)

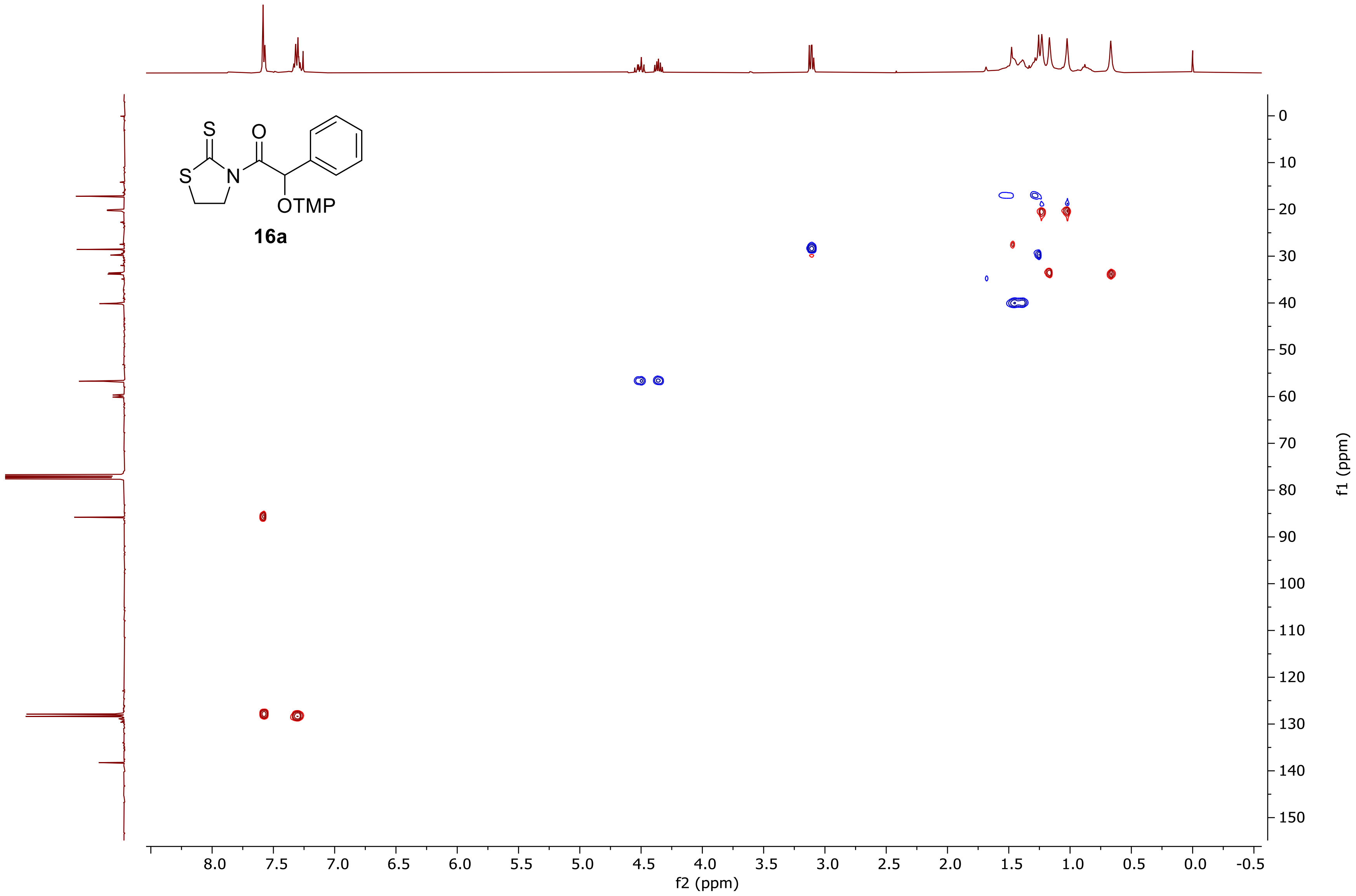

$^1$H NMR (400 MHz, $CDCl_3$)

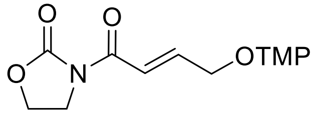


**17a**

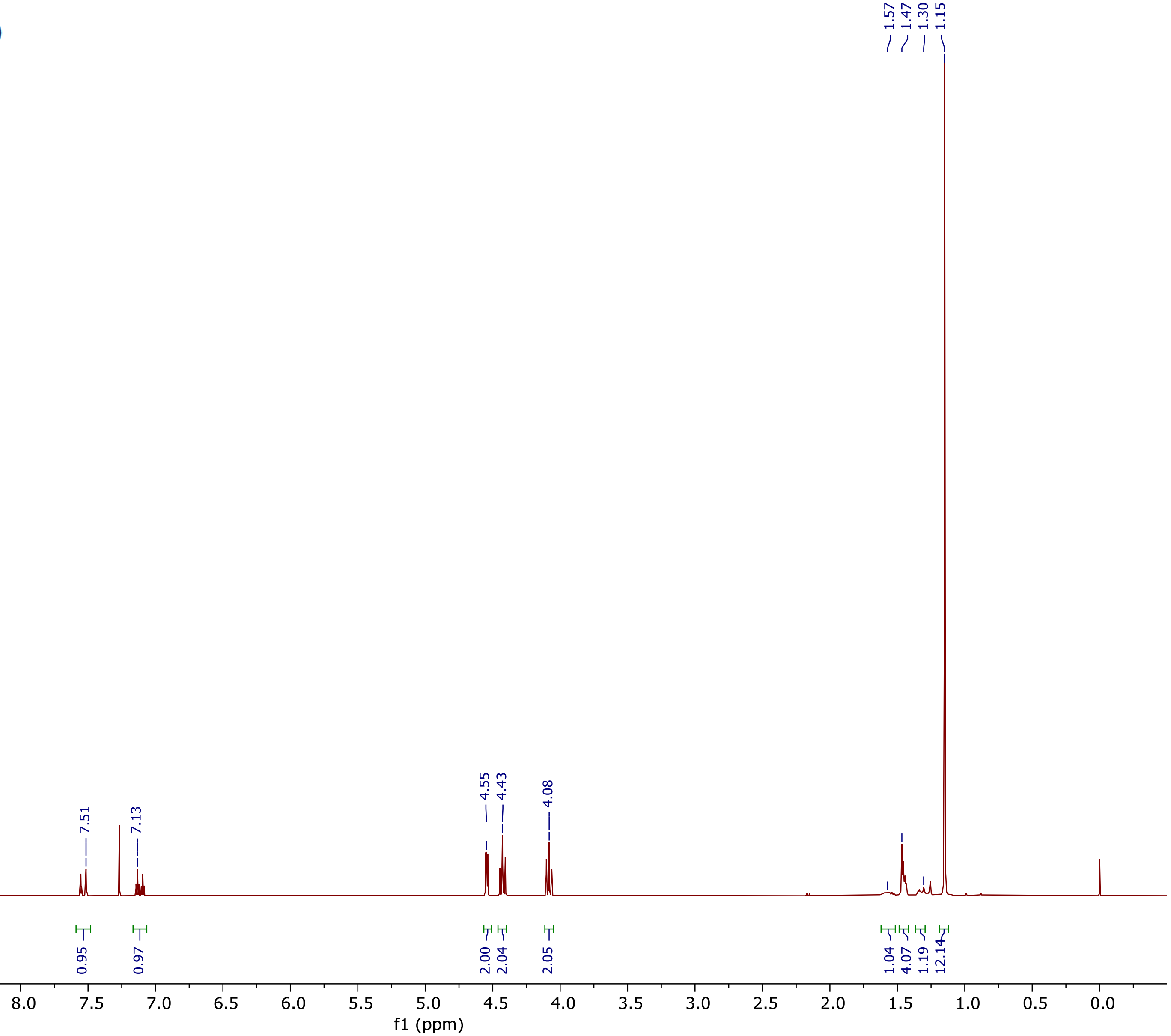

$^{13}C\{^1H\}$ NMR (101 MHz, $CDCl_3$)

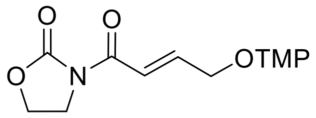


**17a**

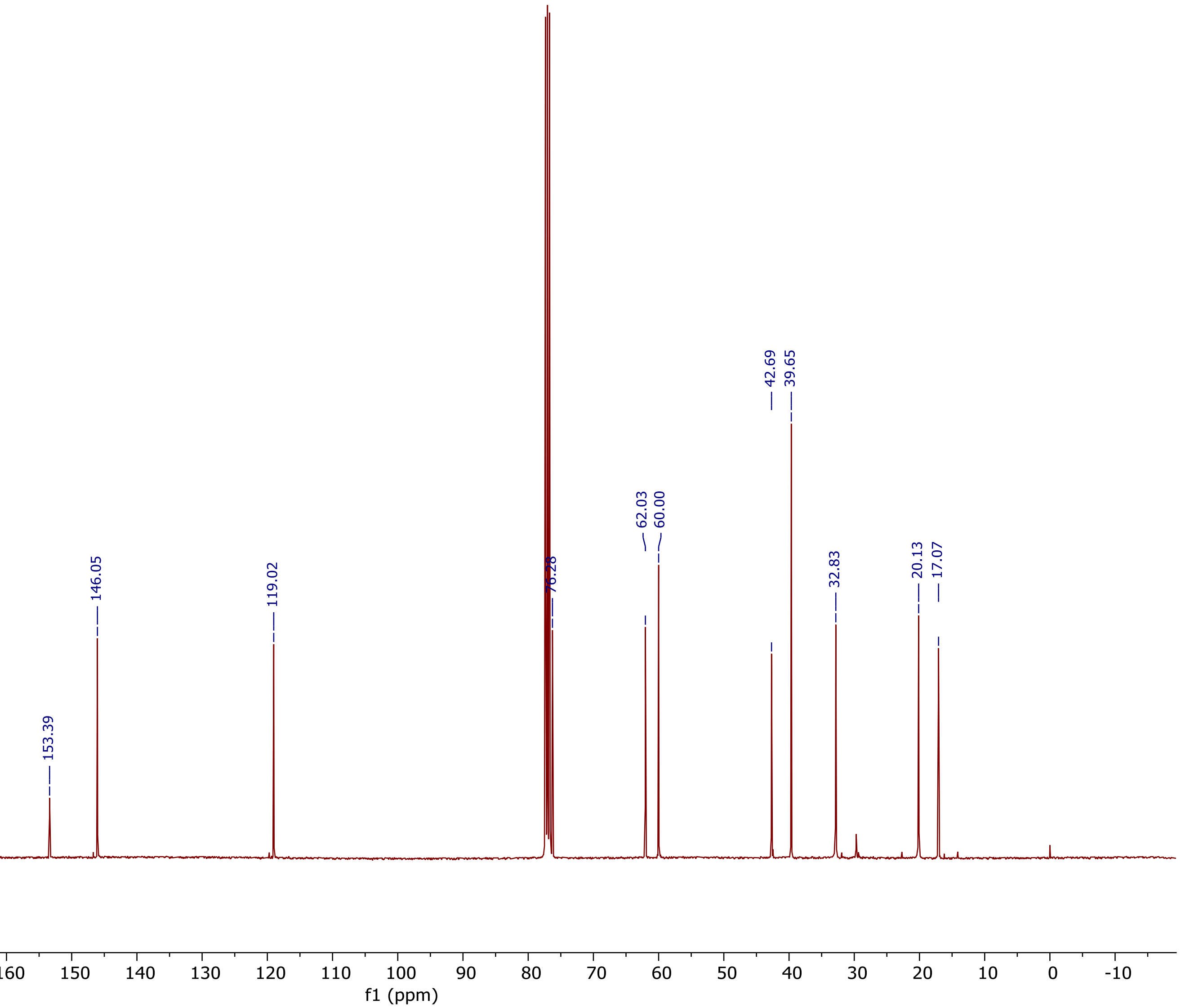

2D $^{1}$H -$^{1}$H COSY (400 MHz, $CDCl_3$)

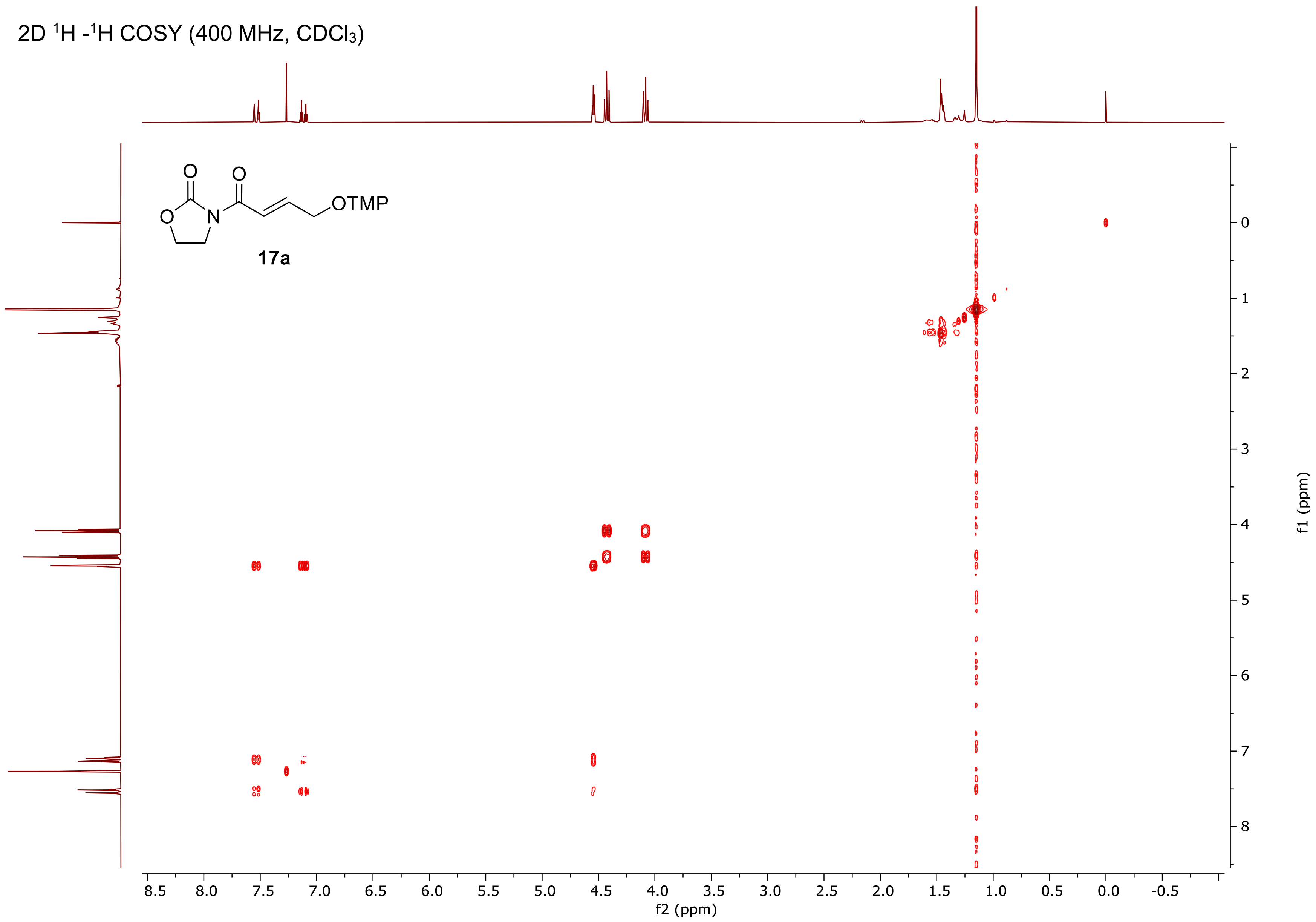

2D $^{1}H$ -$^{13}C$ HSQC (400 MHz, $CDCl_3$)

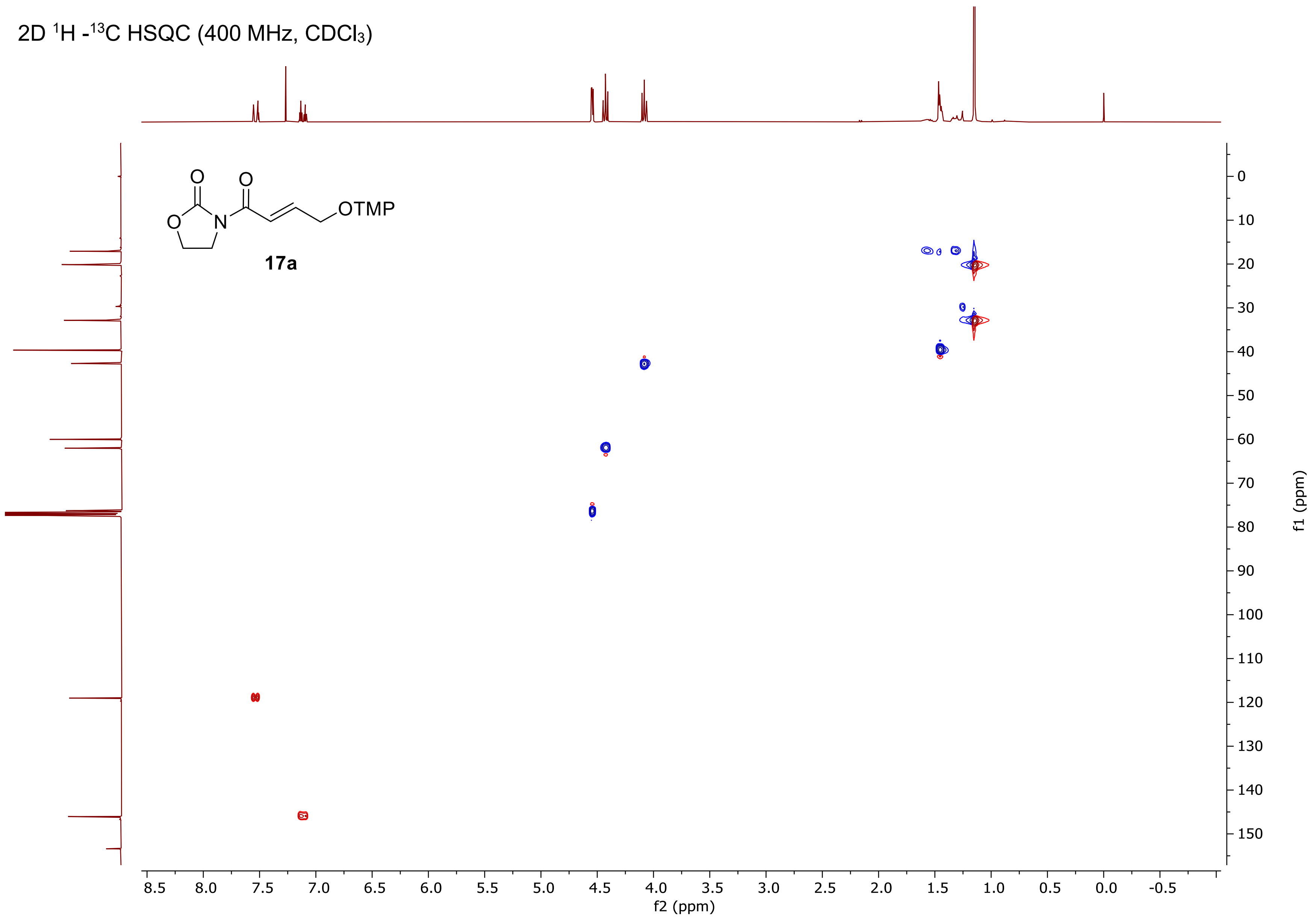

$^1H$ NMR (400 MHz, $CDCl_3$)

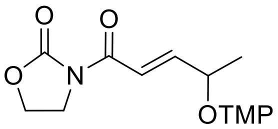


**17b**

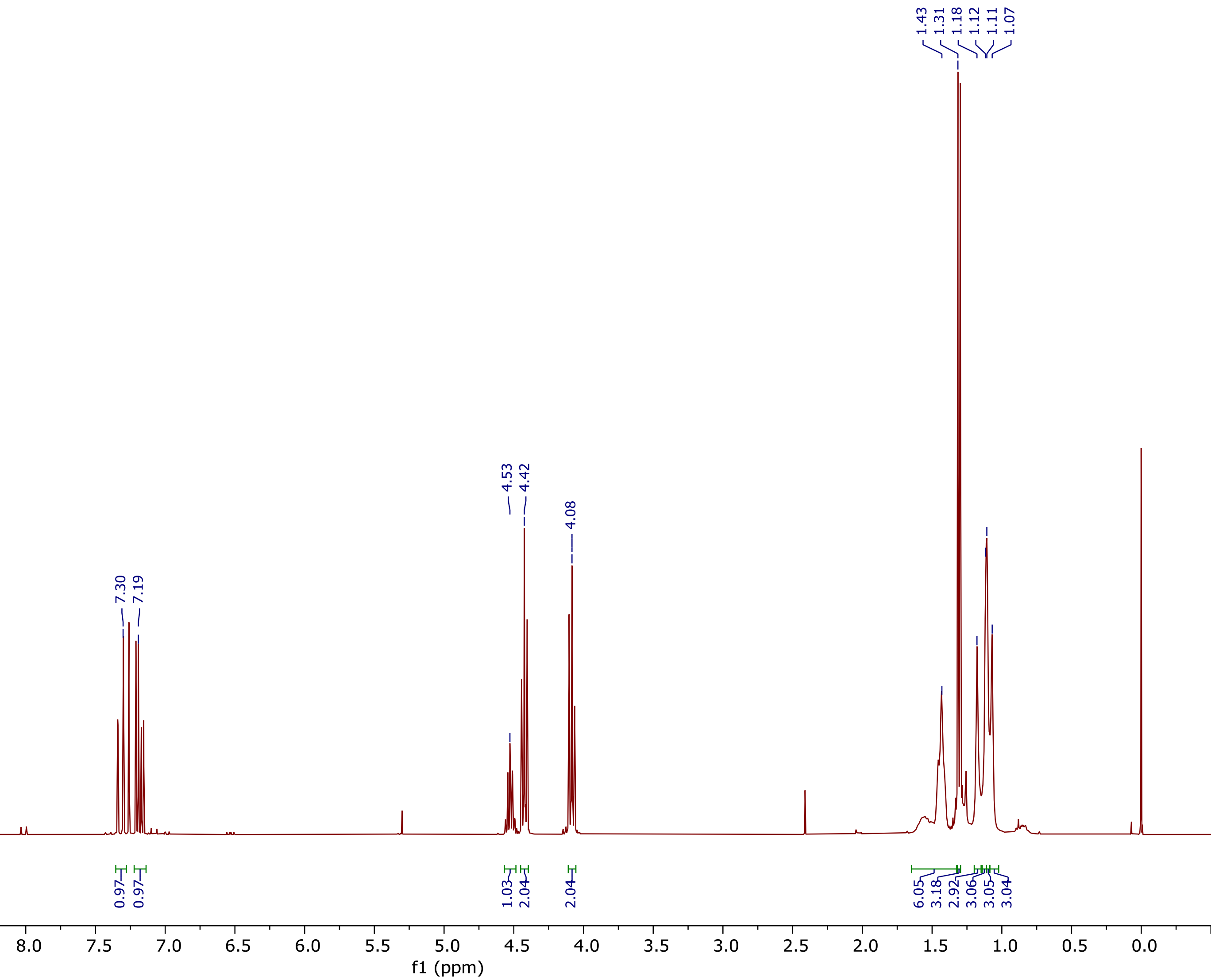

$^{13}C\{^{1}H\}$ NMR (101 MHz, $CDCl_3$)

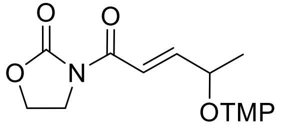


**17b**

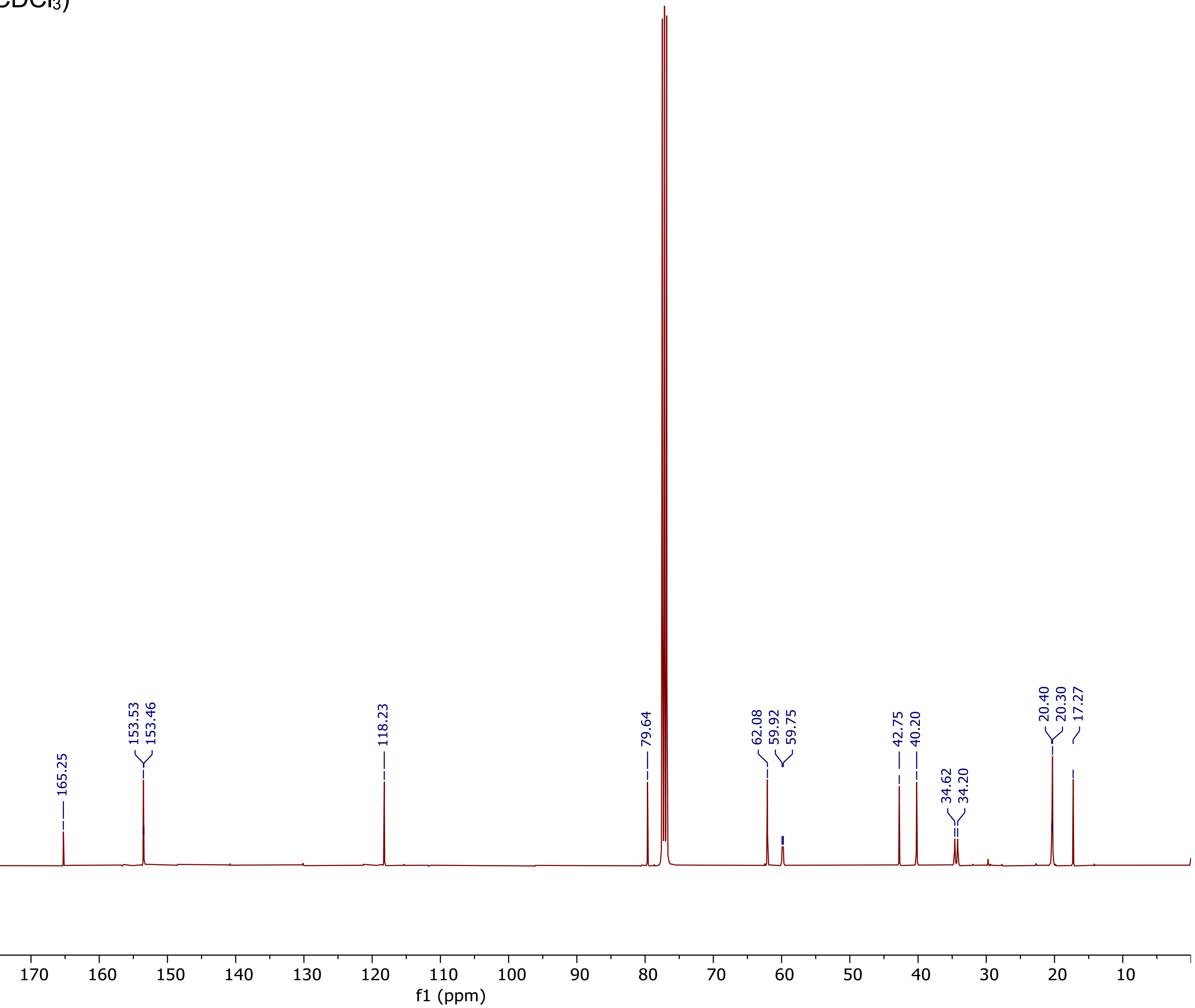

2D $^{1}H$ -$^{1}H$ COSY (400 MHz, $CDCl_3$)

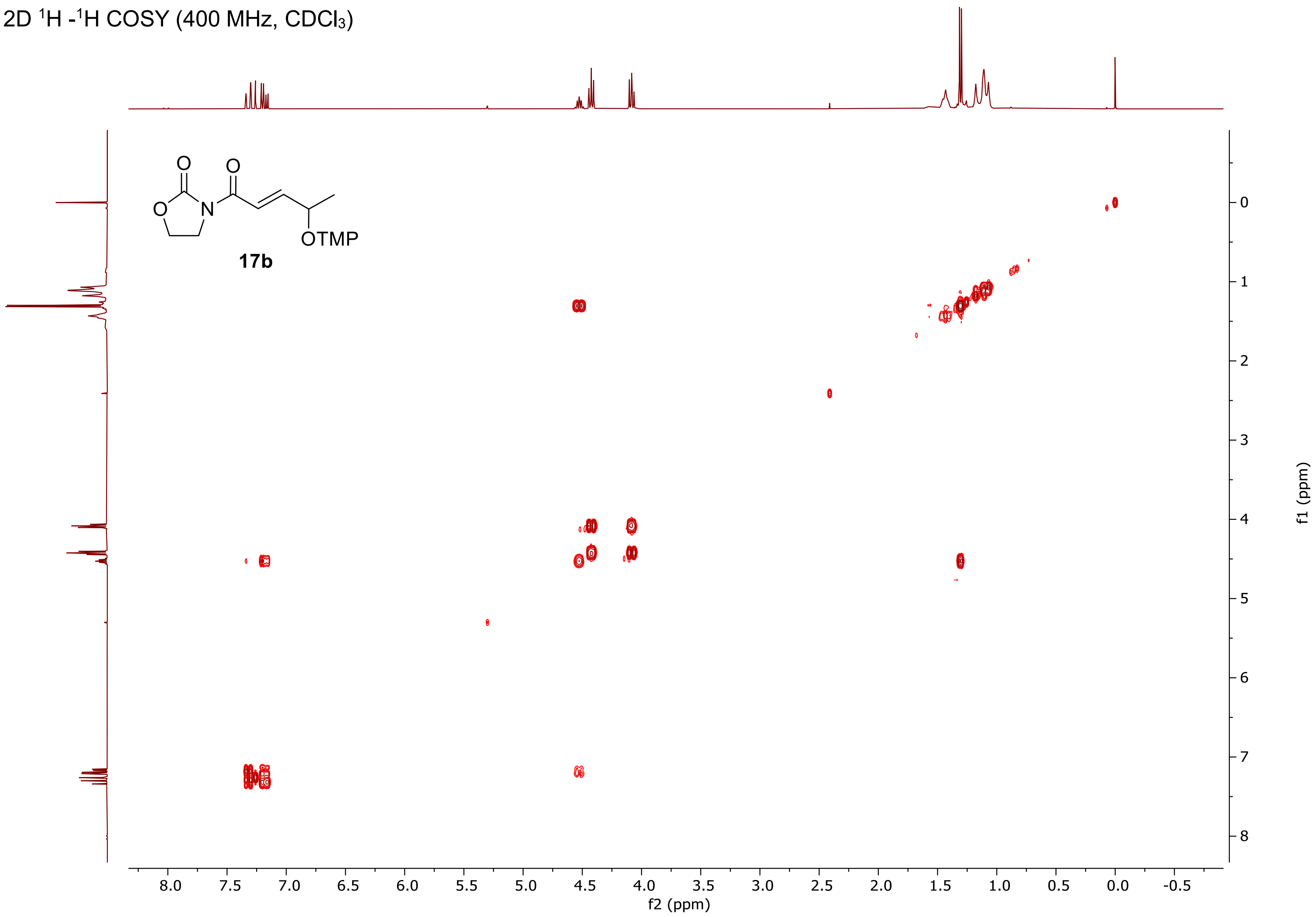

2D $^{1}H$ -$^{13}C$ HSQC (400 MHz, $CDCl_3$)

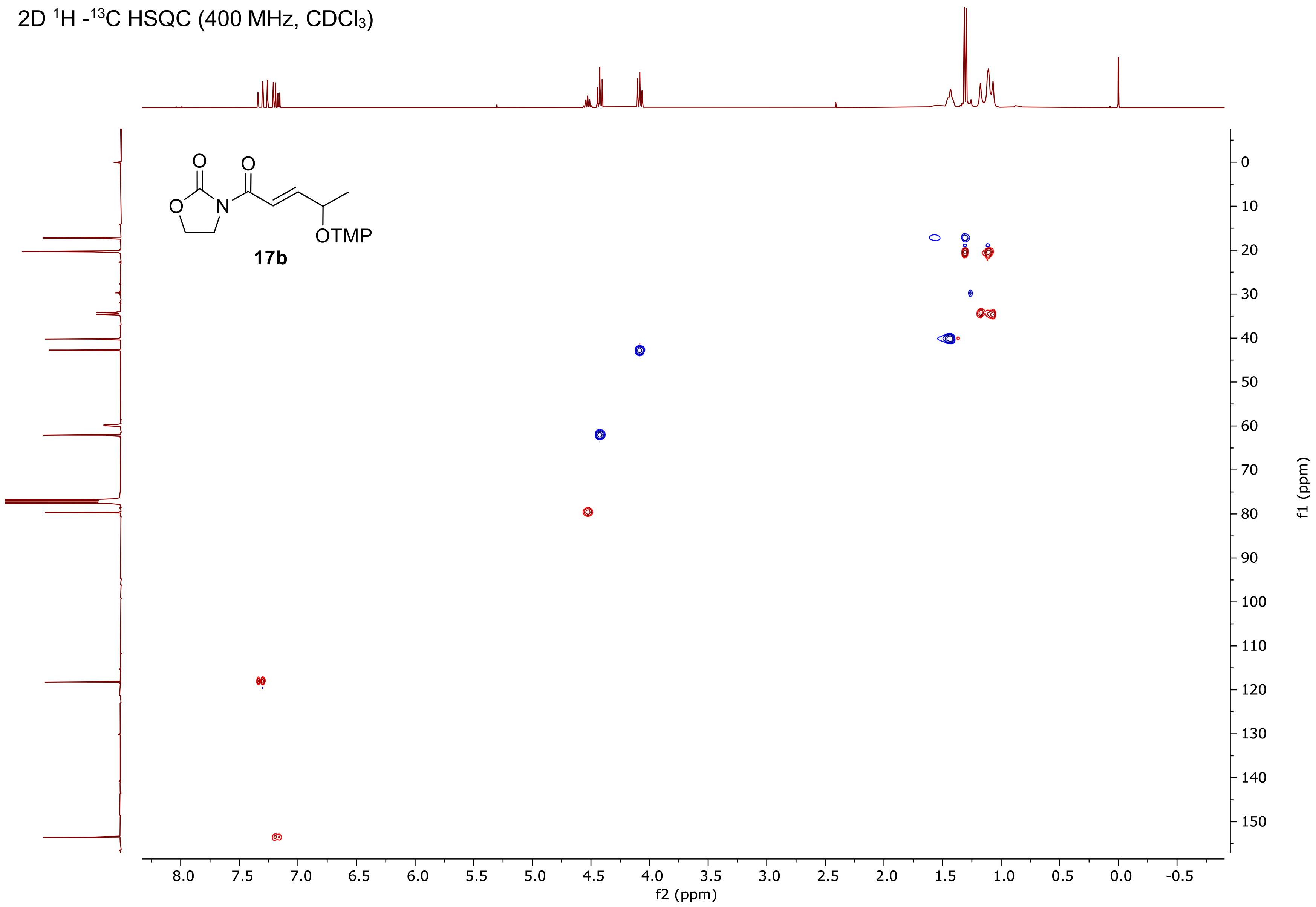

<sup></sup>

[1]H NMR (500 MHz, $CDCl_3$)

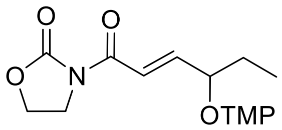


**17c**

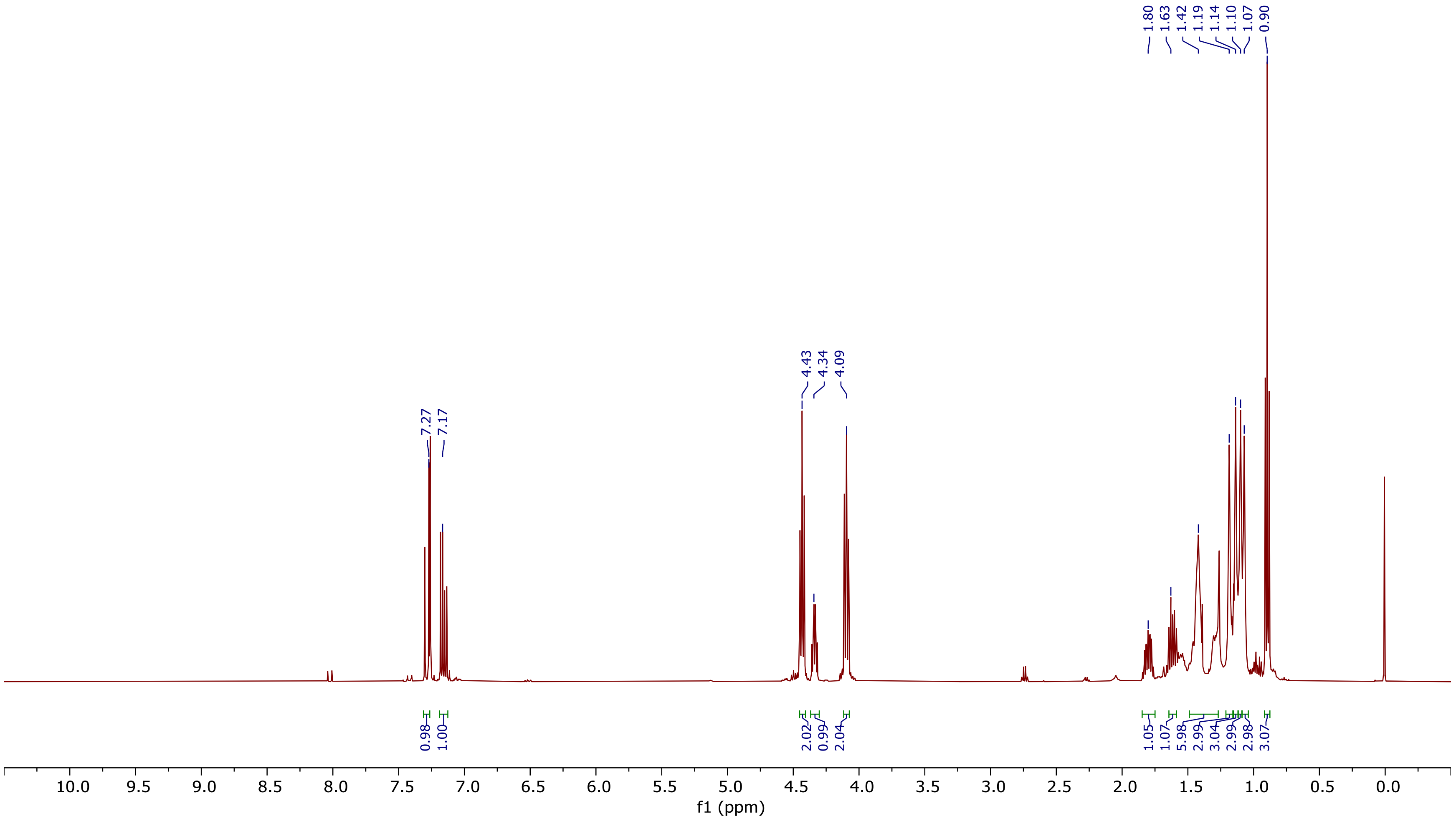

$^{13}C\{^{1}H\}$ NMR (126 MHz, $CDCl_3$)

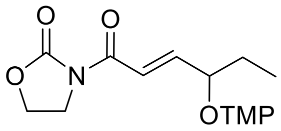

**17c**

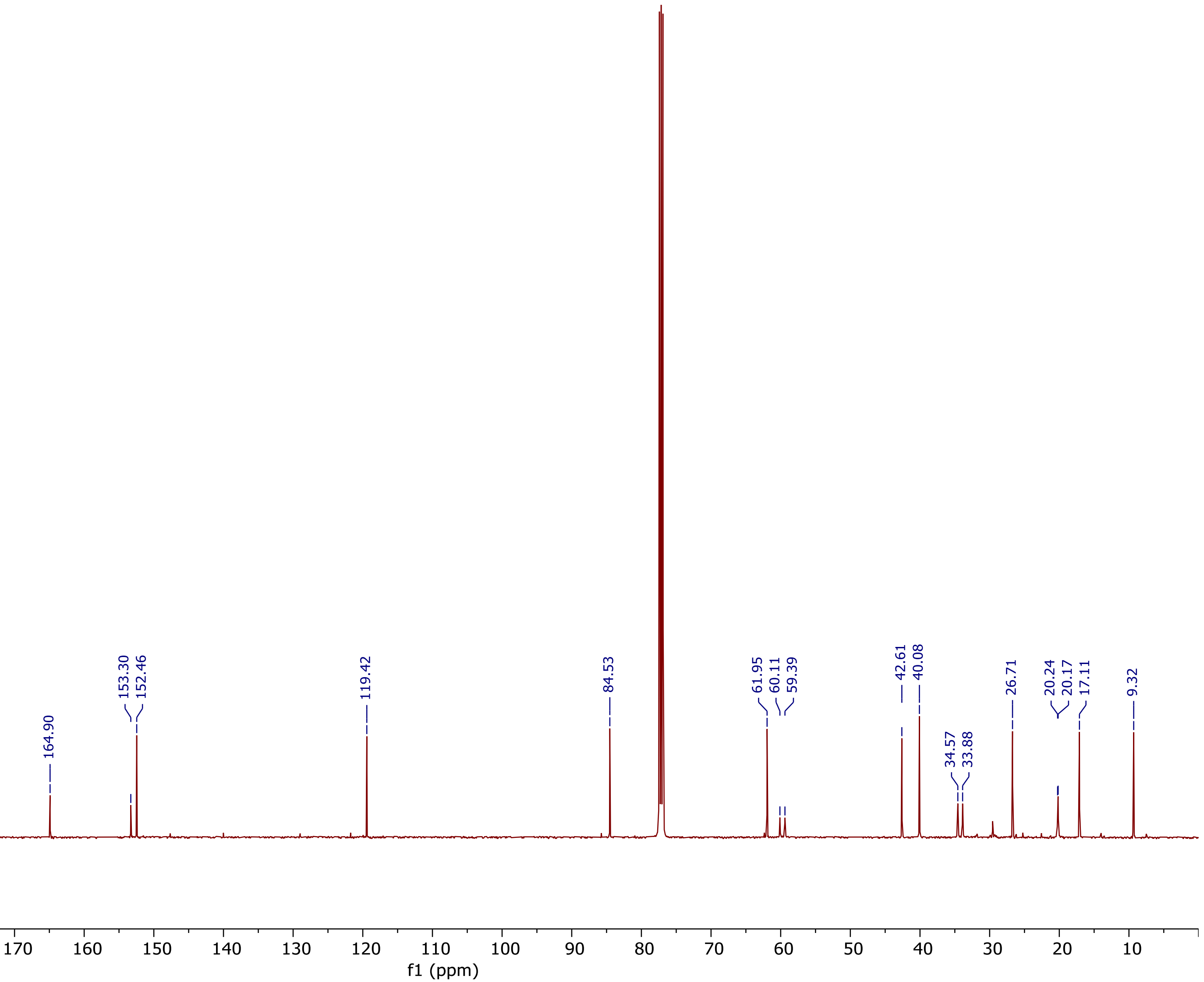

2D ¹H -¹H COSY (500 MHz, $CDCl_3$)

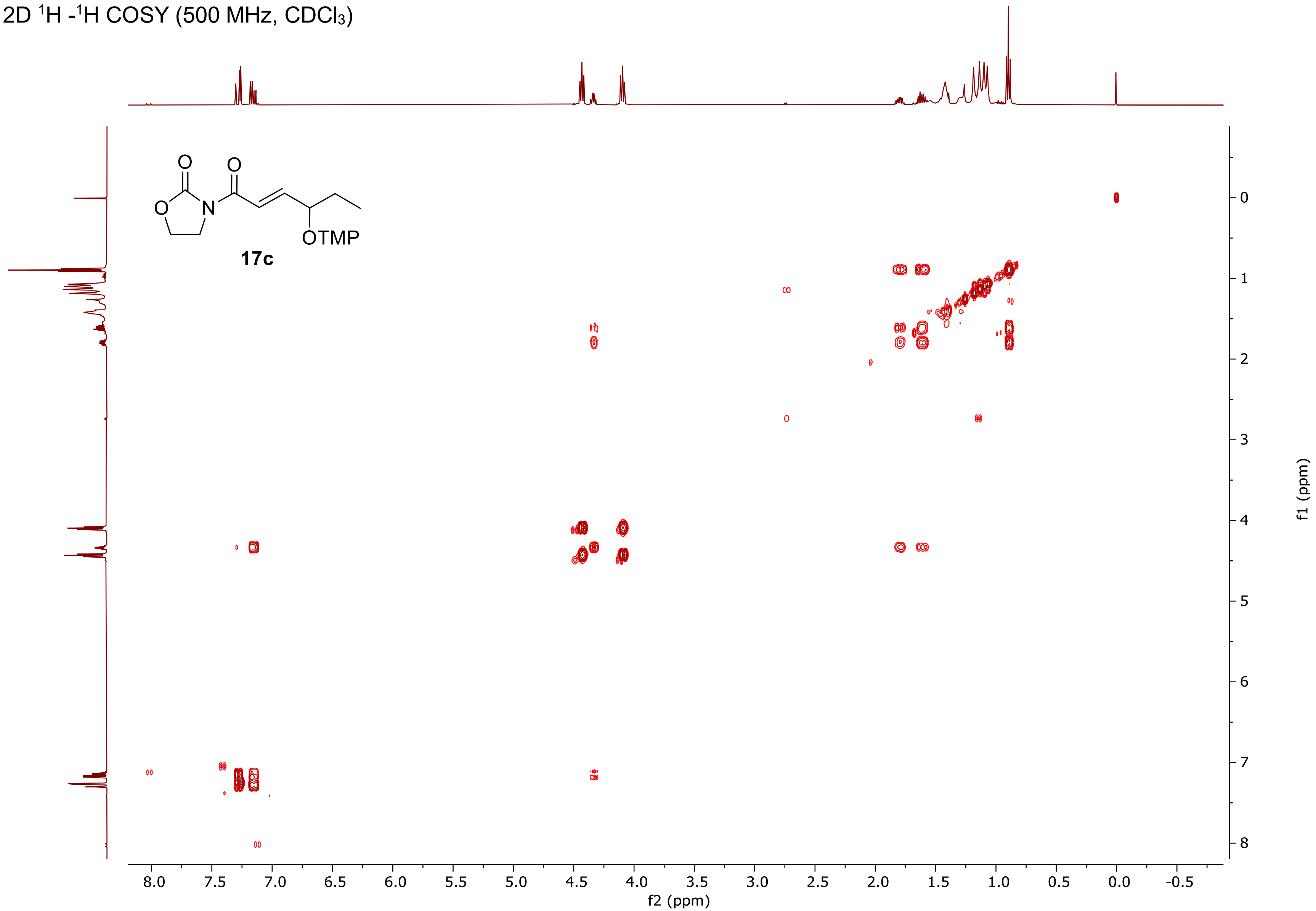

2D $^1$H -$^{13}$C HSQC (500 MHz, $CDCl_3$)

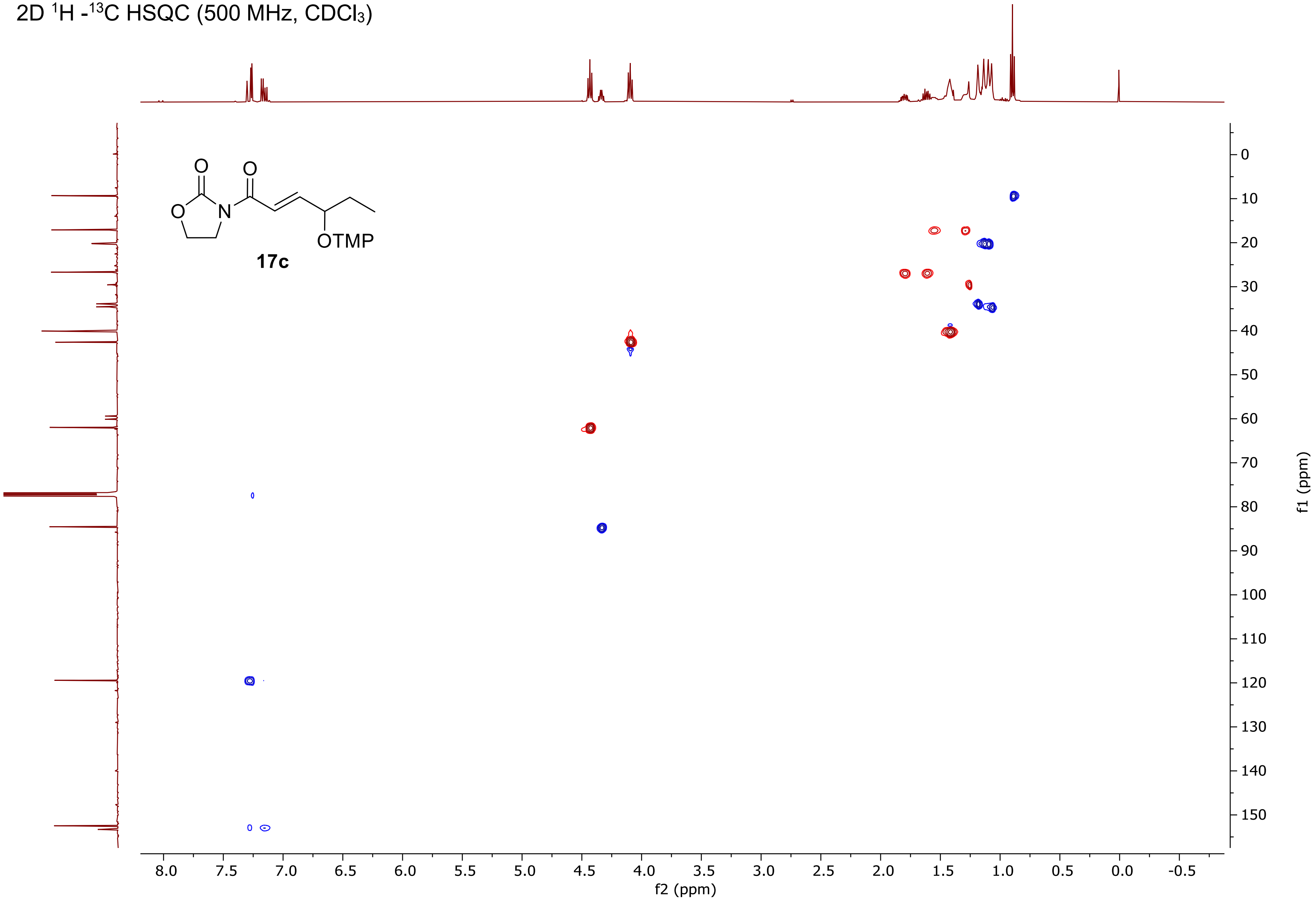

$^1$H NMR (500 MHz, $CDCl_3$)

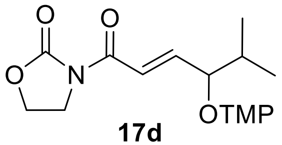


**17d**

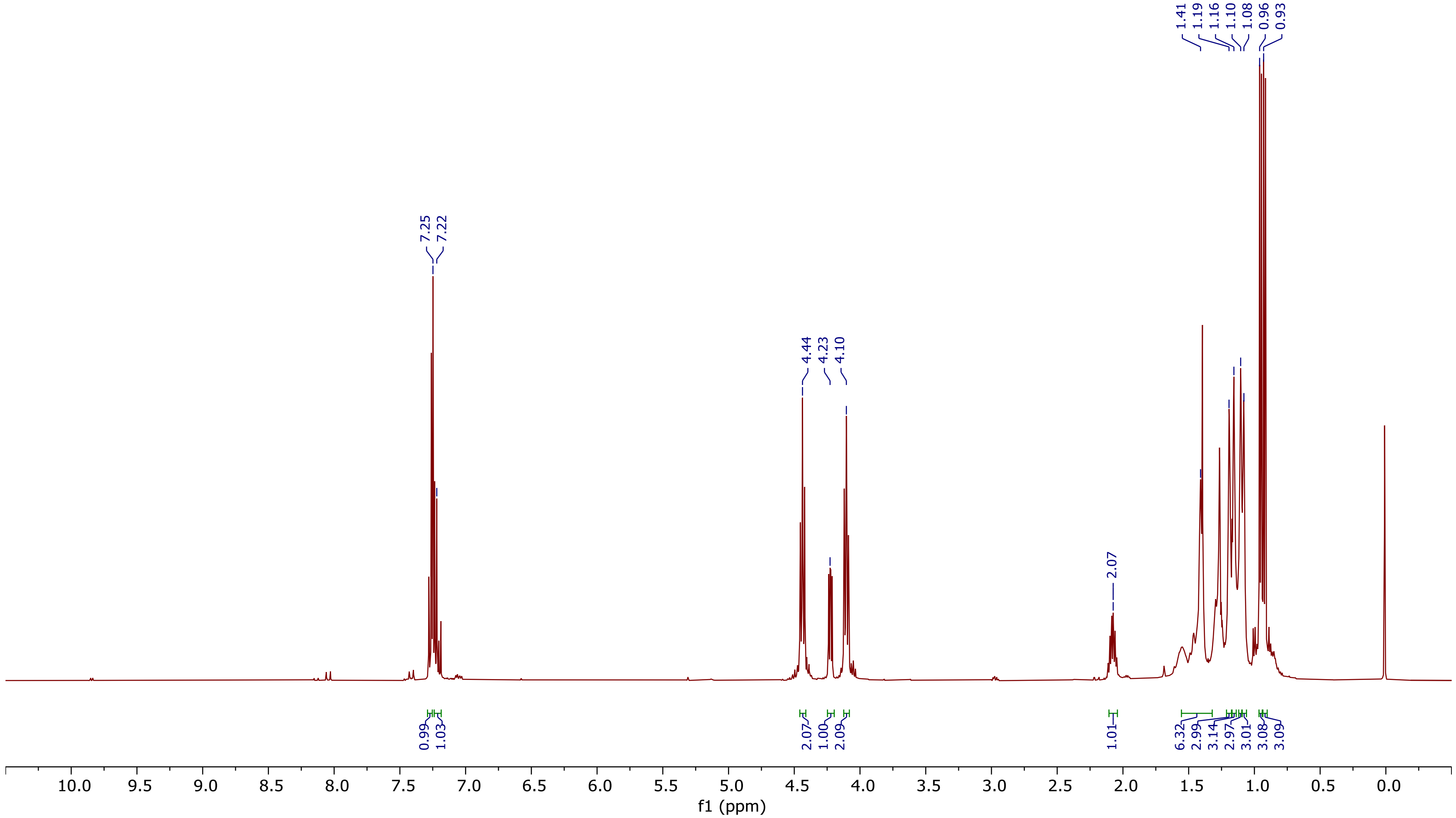

$^{13}C\{^1H\}$ NMR (126 MHz, $CDCl_3$)

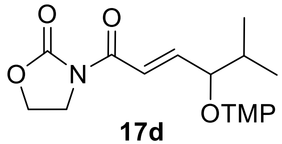


**17d**

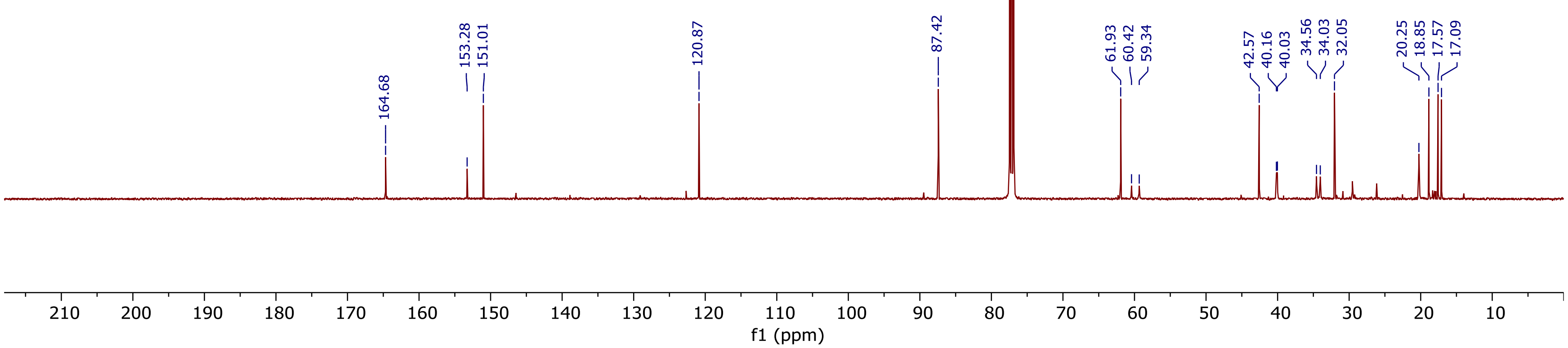

2D $^1H$ -$^1H$ COSY (500 MHz, $CDCl_3$)

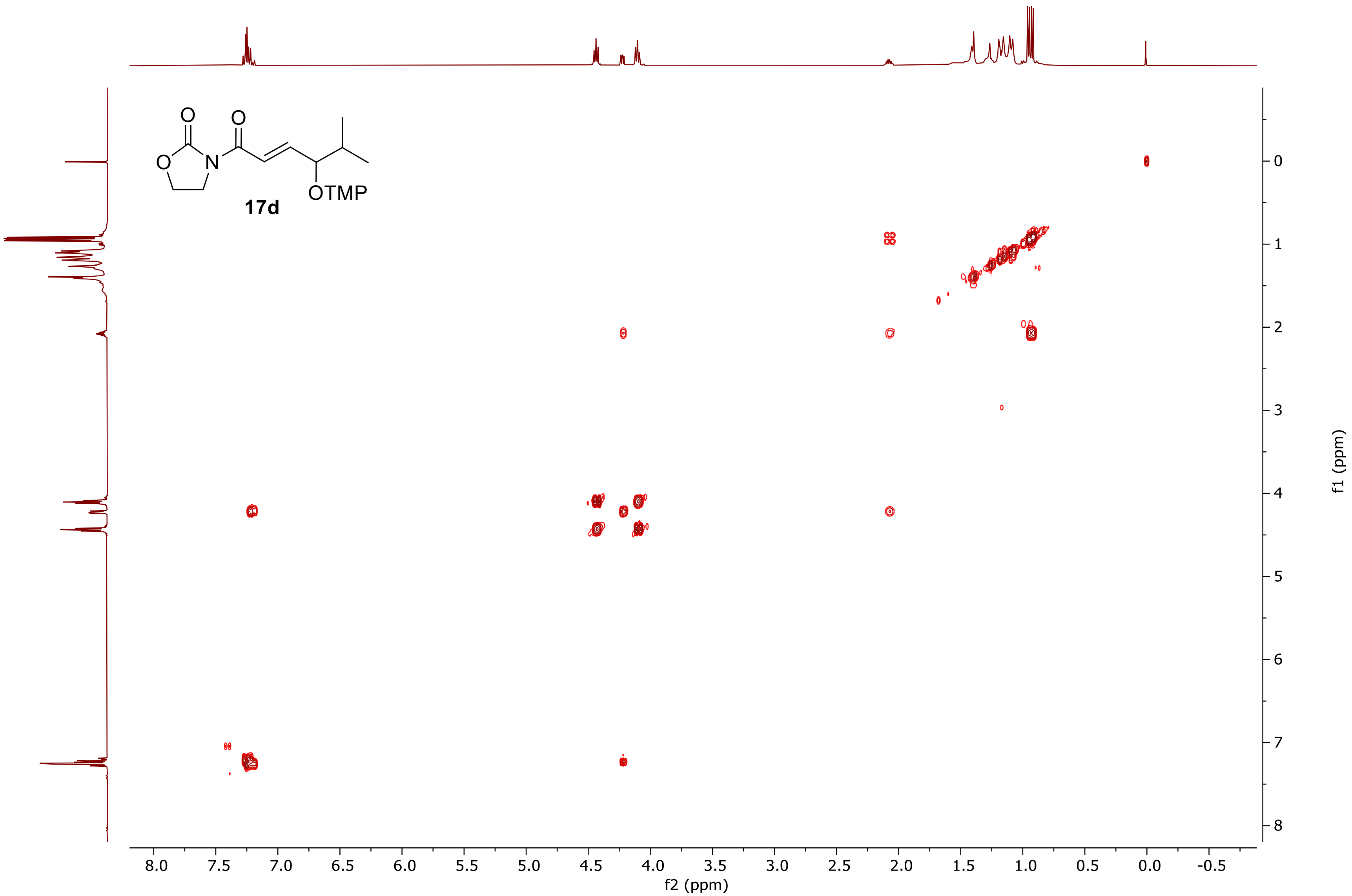

2D $^{1}H$ -$^{13}C$ HSQC (500 MHz, $CDCl_3$)

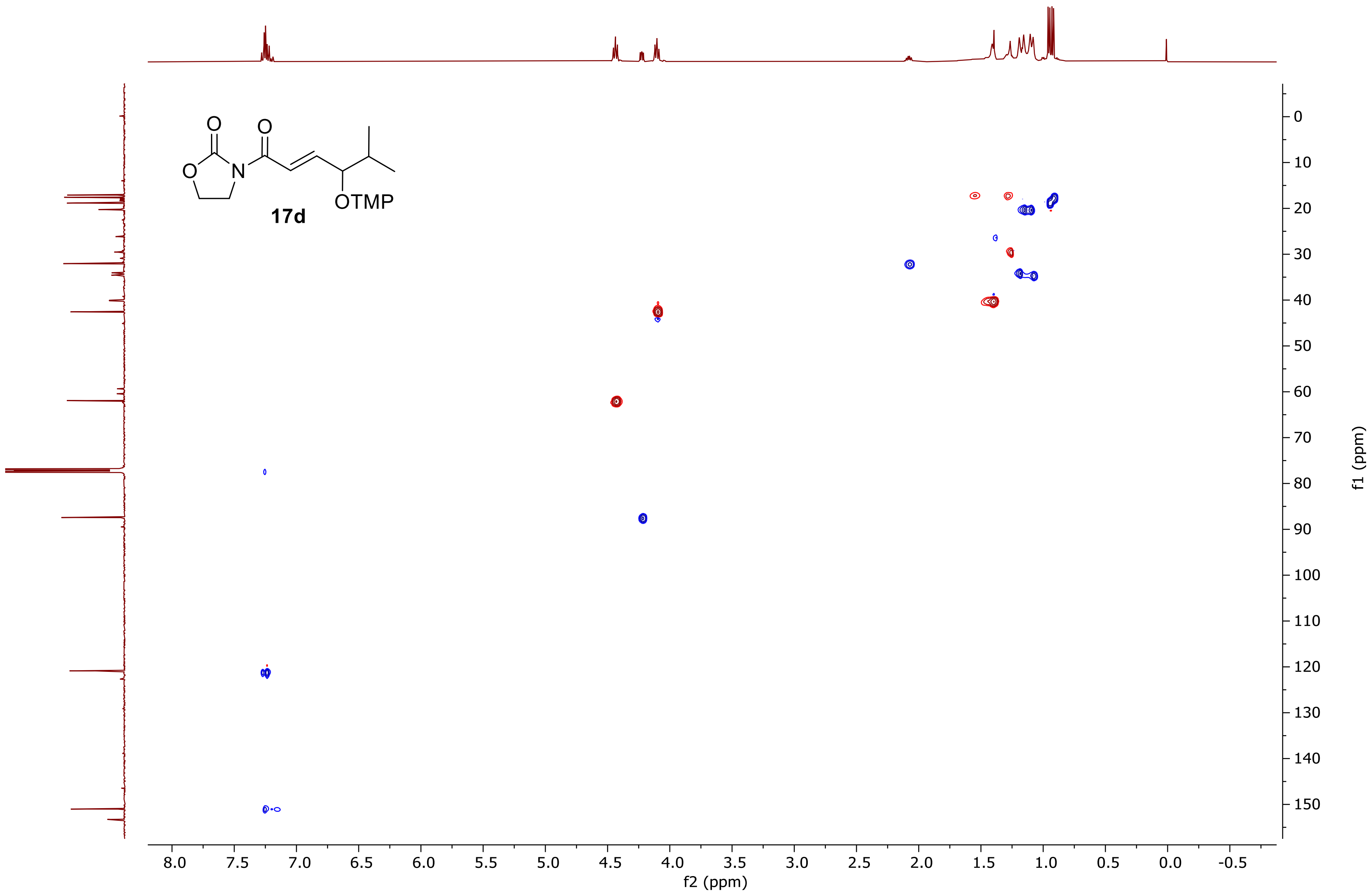

$^1H$ NMR (500 MHz, $CDCl_3$)

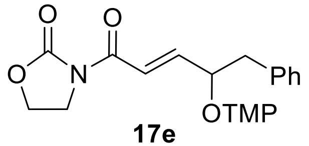


**17e**

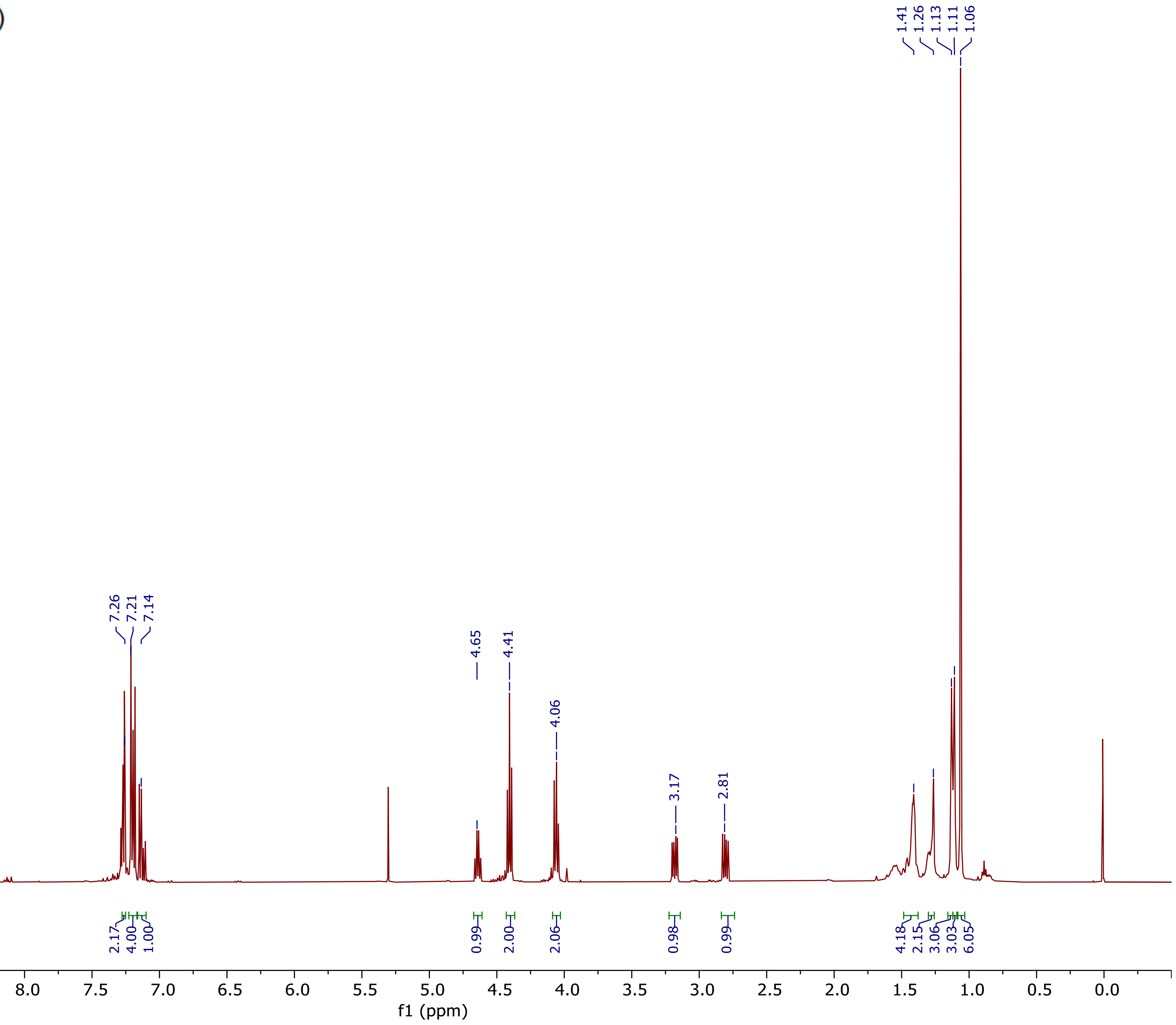

$^{13}C\{^{1}H\}$ NMR (126 MHz, $CDCl_3$)

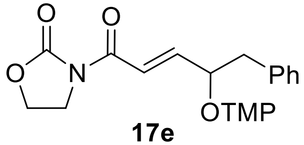


**17e**

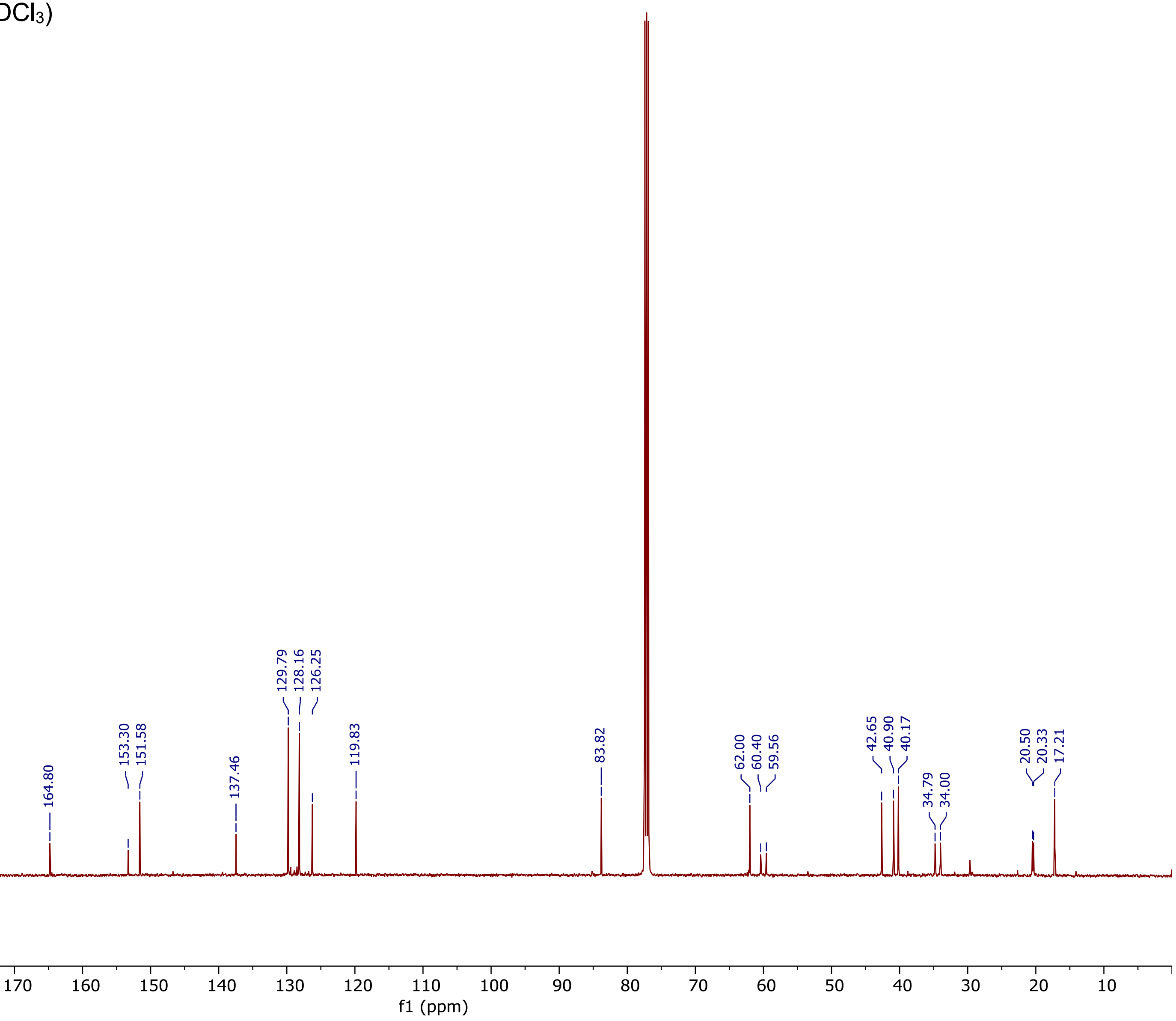

2D $^1$H -$^1$H COSY (500 MHz, $CDCl_3$)

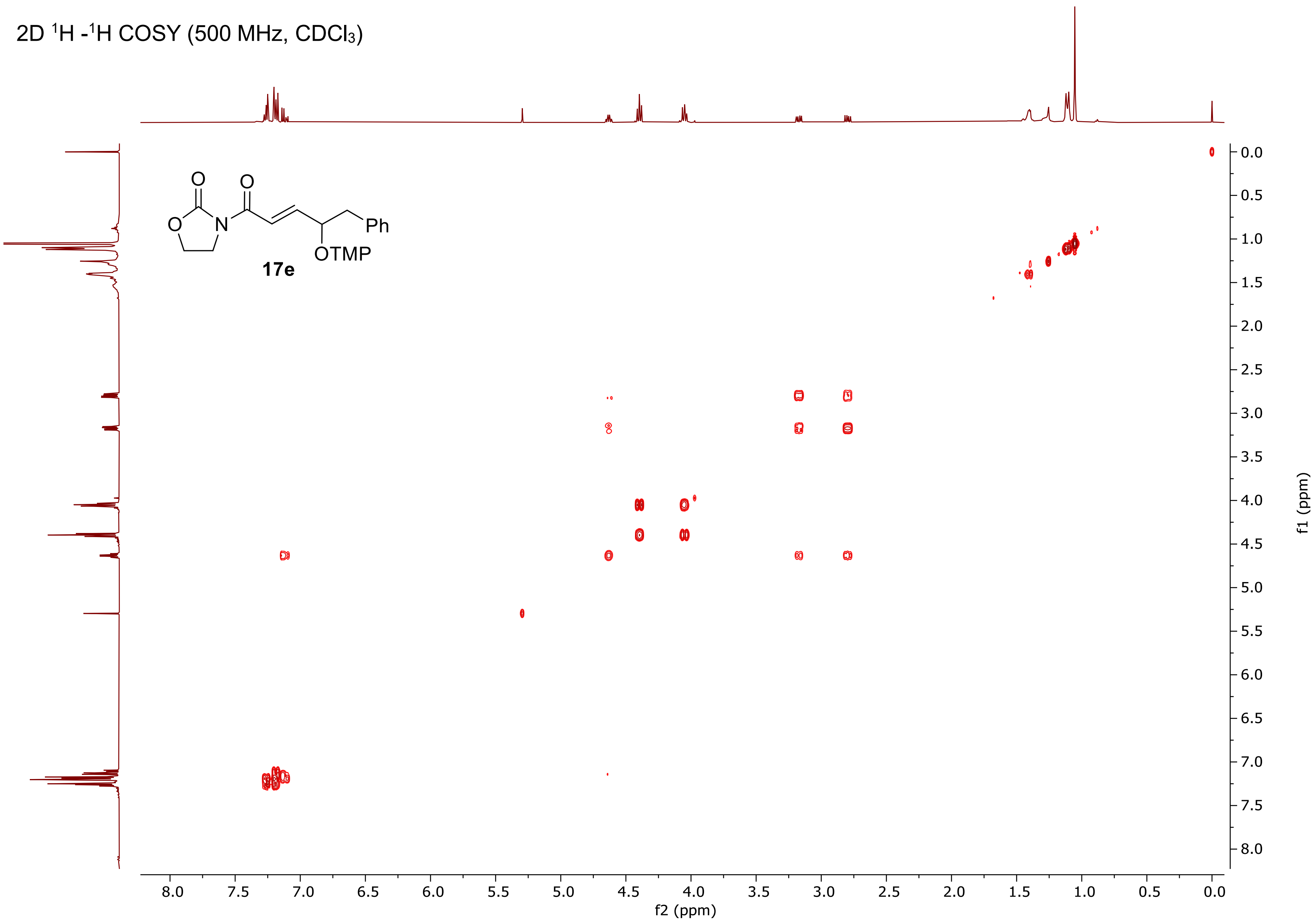

2D $^{1}H$ -$^{13}C$ HSQC (500 MHz, $CDCl_3$)

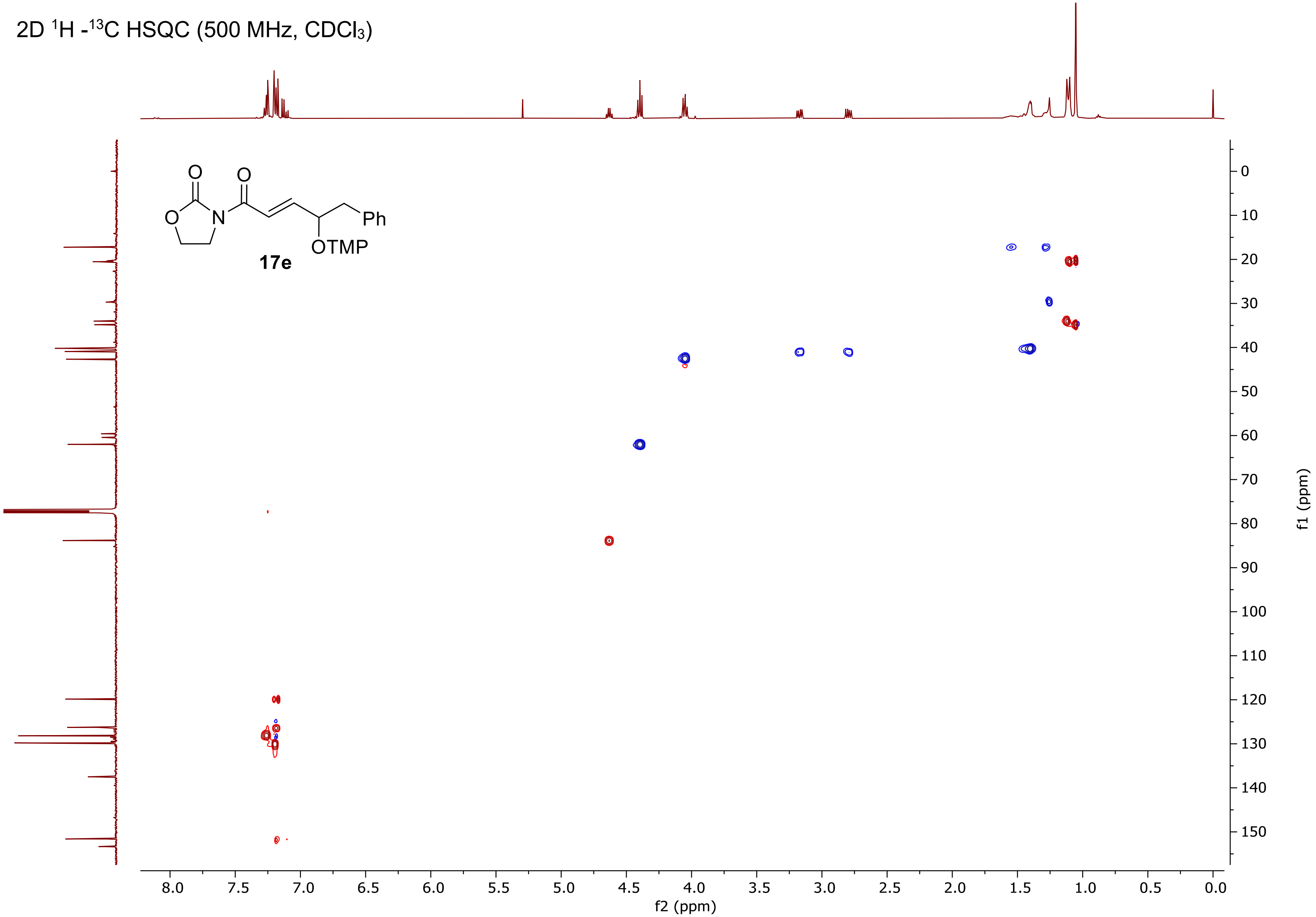

$^{1}$H NMR (400 MHz, $CDCl_3$)

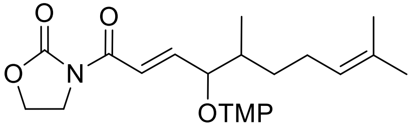


**17f**

A mixture of diastereomers
in a 56:44 ratio

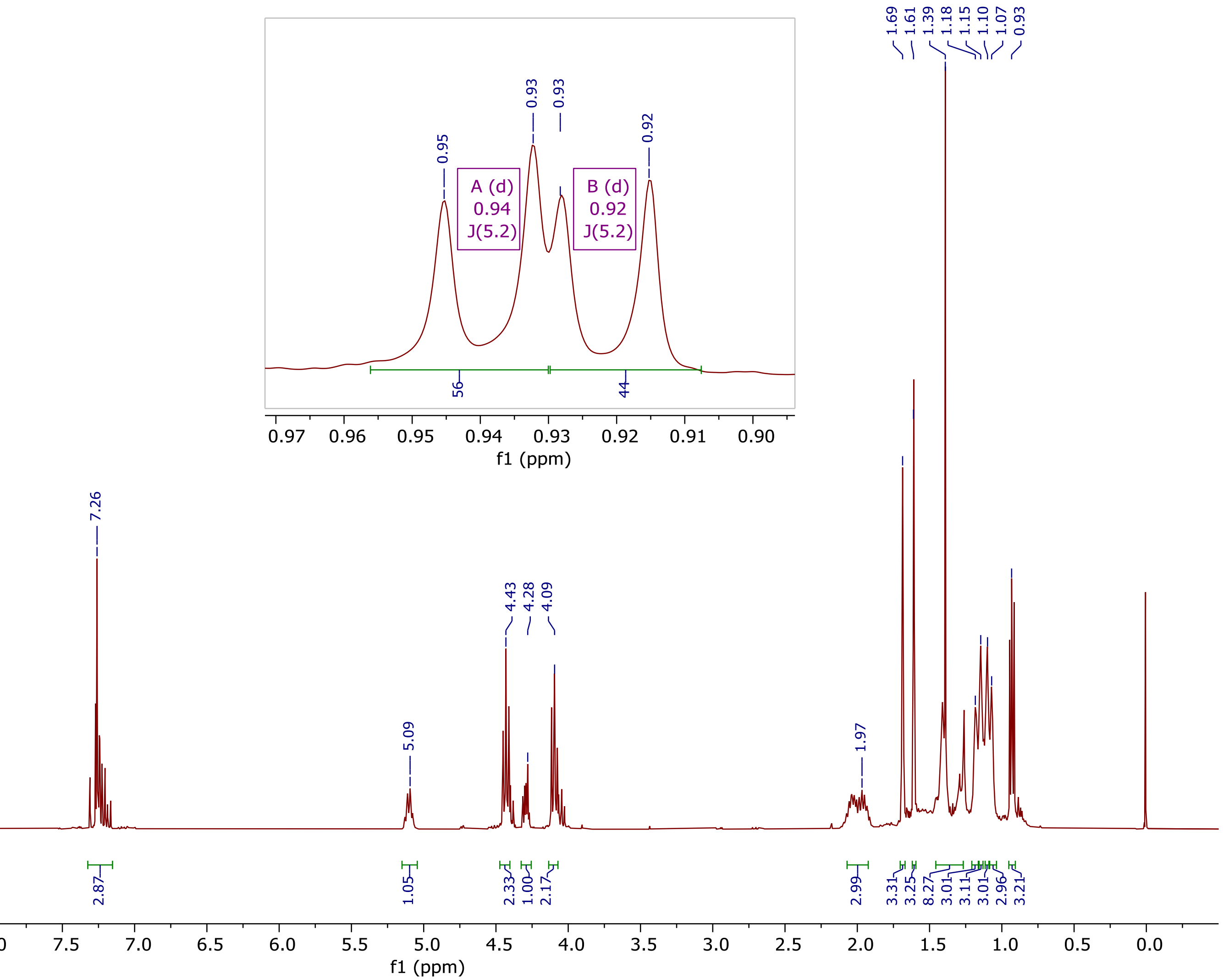

$^{13}C\{^1H\}$ NMR (101 MHz, $CDCl_3$)

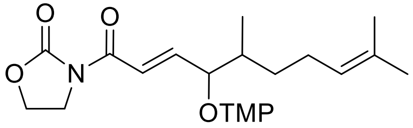


**17f**

A mixture of diastereomers
in a 56:44 ratio

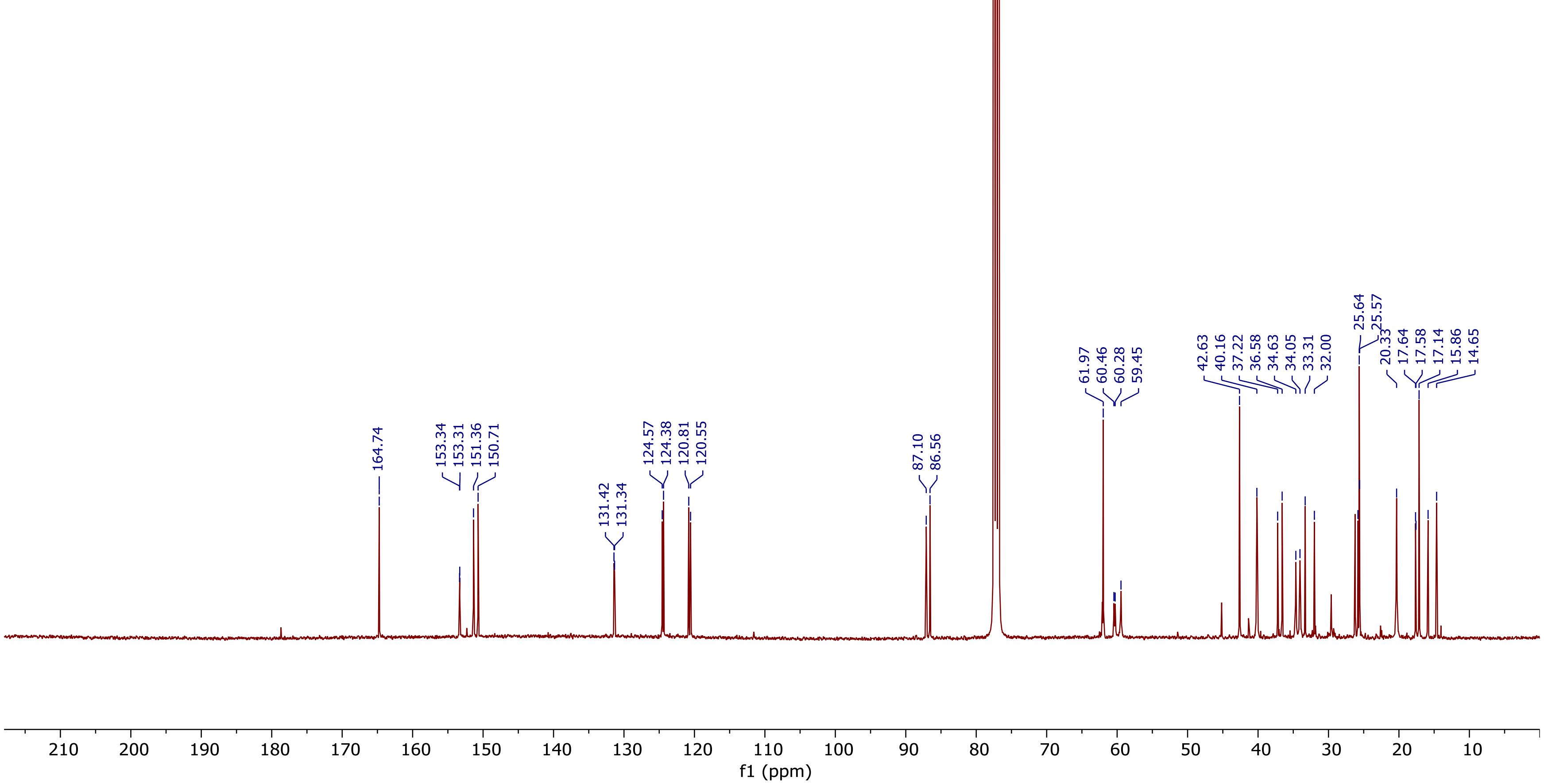

2D $^1H$ -$^1H$ COSY (400 MHz, $CDCl_3$)

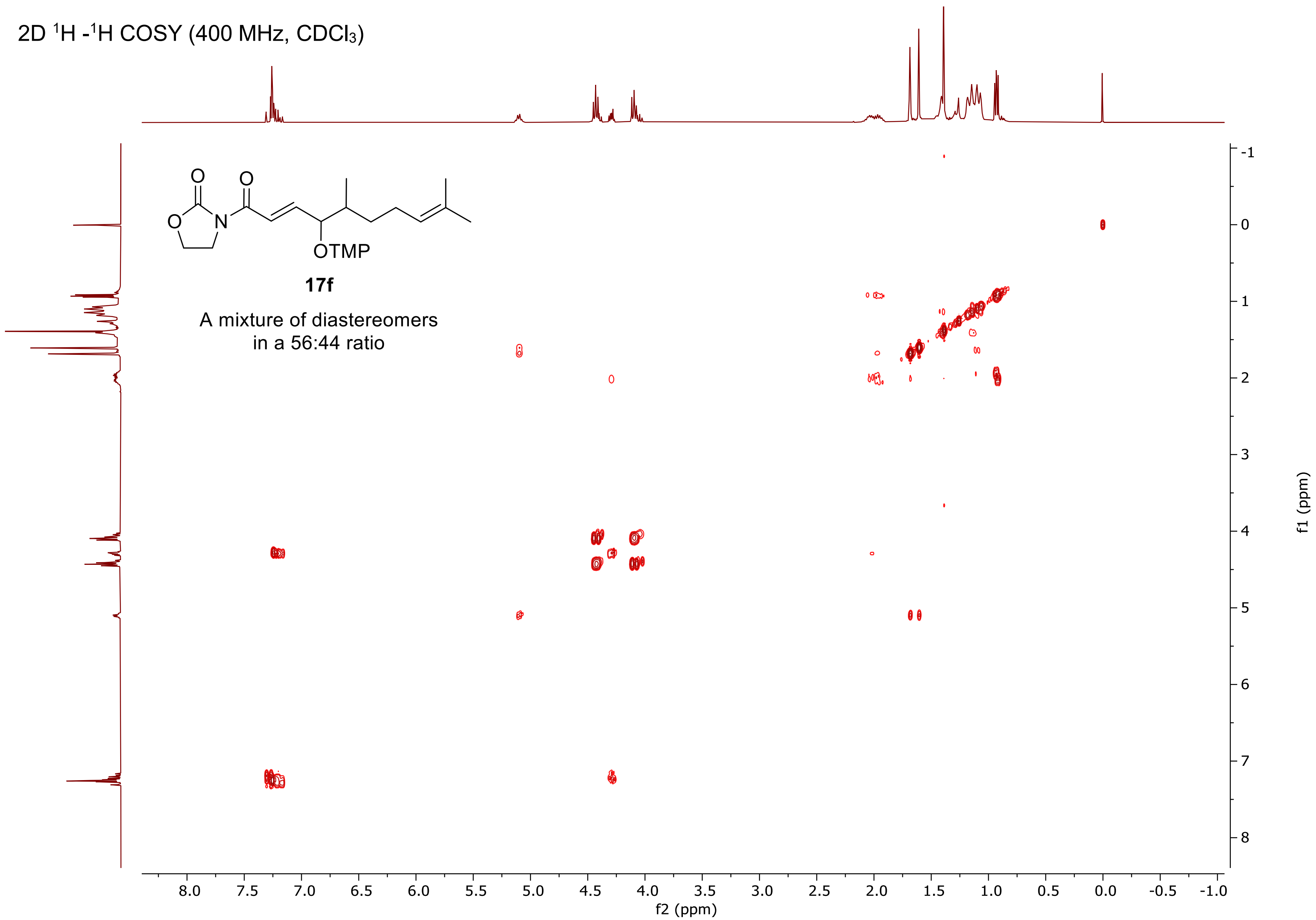

2D $^1H$ -$^{13}C$ HSQC (400 MHz, $CDCl_3$)

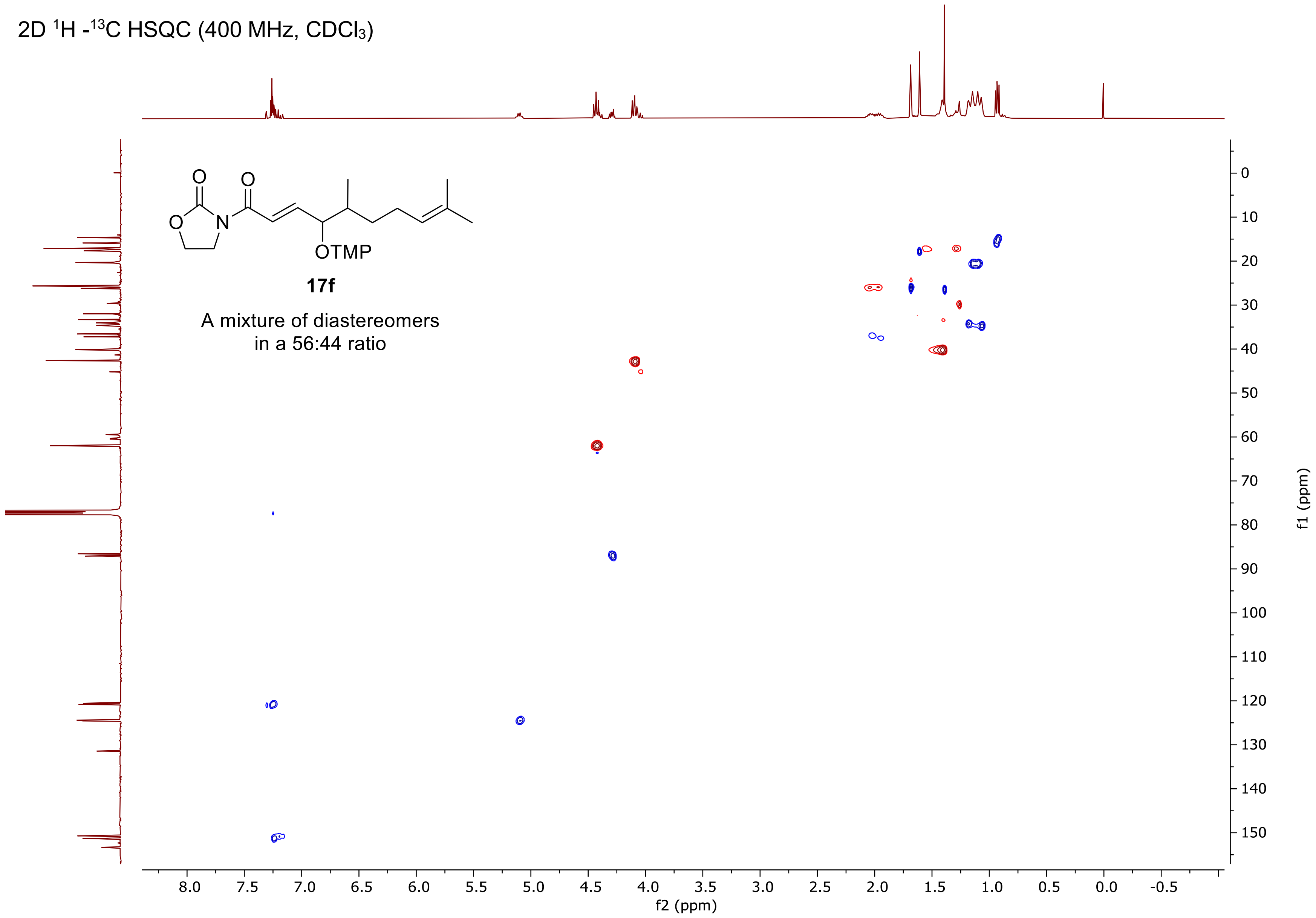

$^1$H NMR (400 MHz, $CDCl_3$)

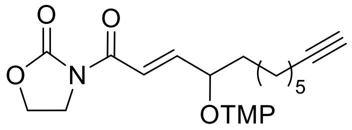


**17g**

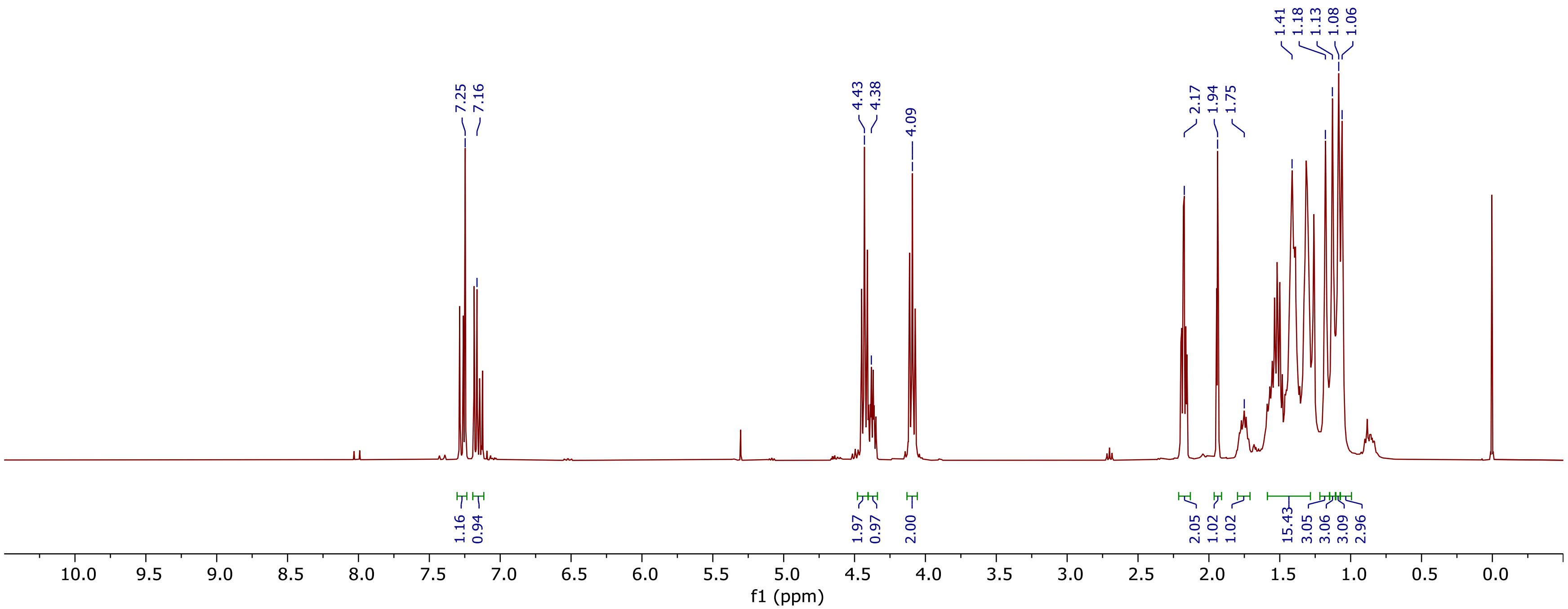

$^{13}C\{^1H\}$ NMR (101 MHz, $CDCl_3$)

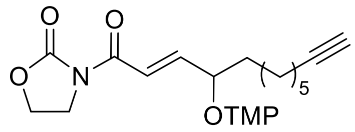


**17g**

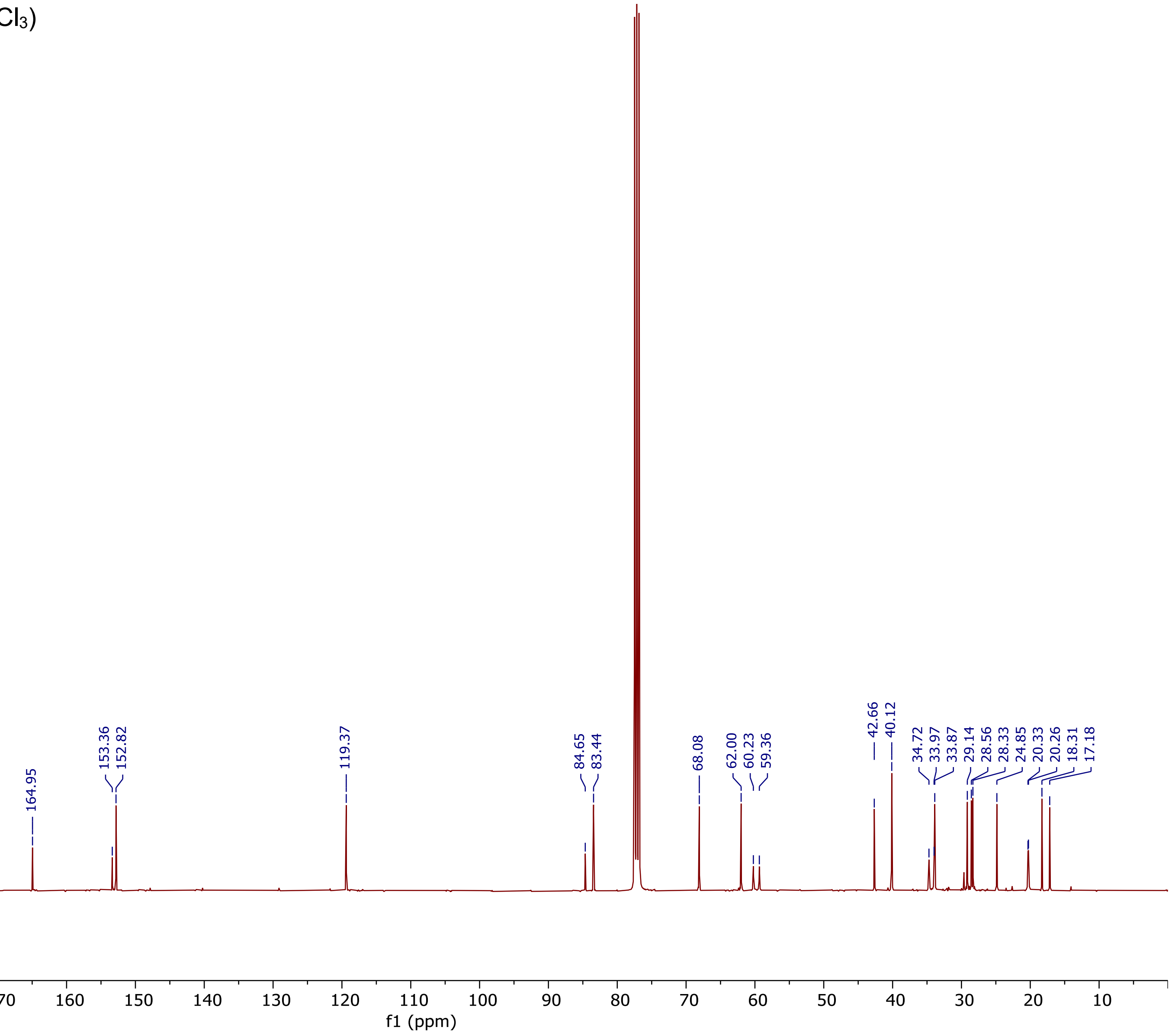

2D ¹H -¹H COSY (400 MHz, CDCl$_3$)

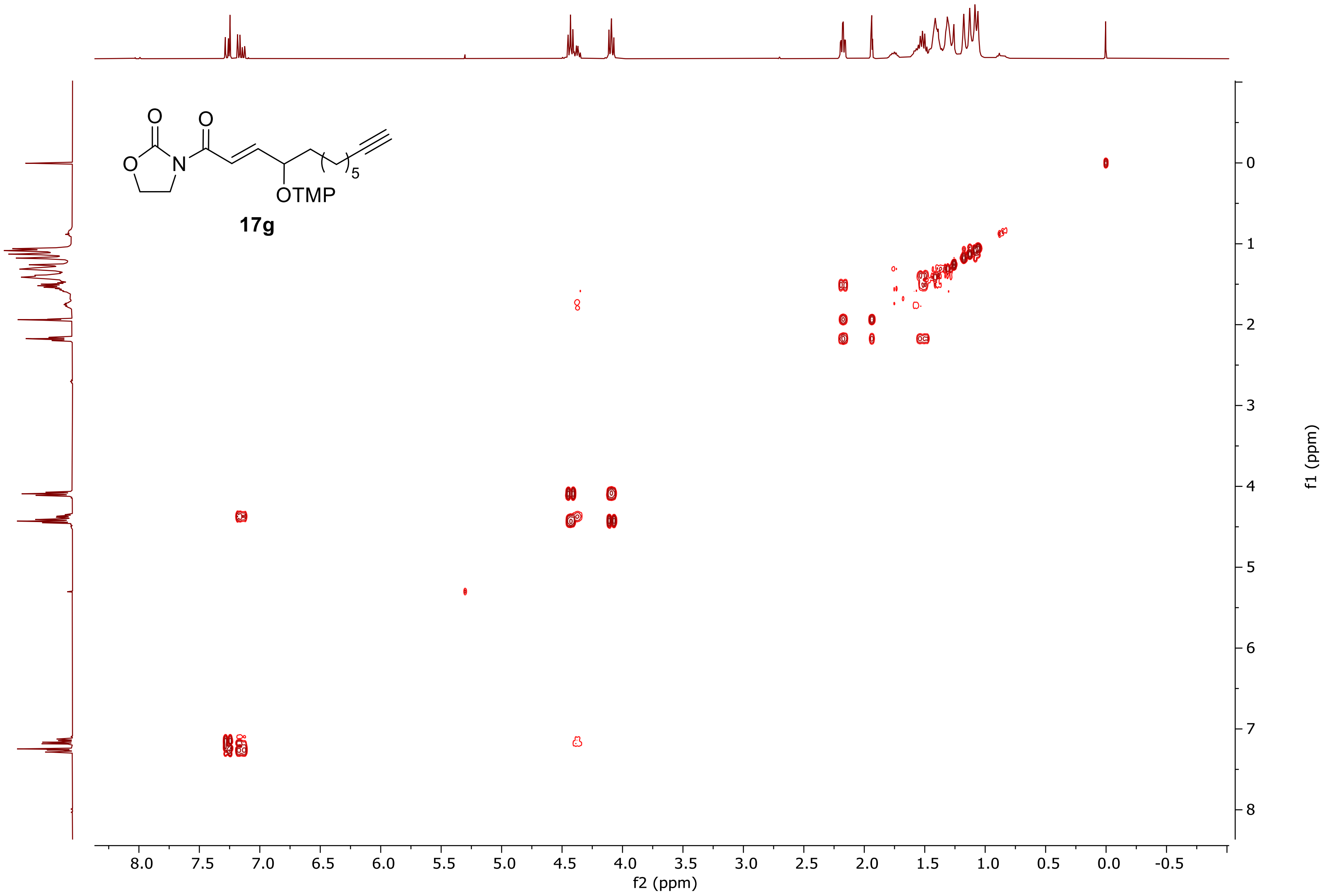

2D $^{1}H$ -$^{13}C$ HSQC (400 MHz, $CDCl_3$)

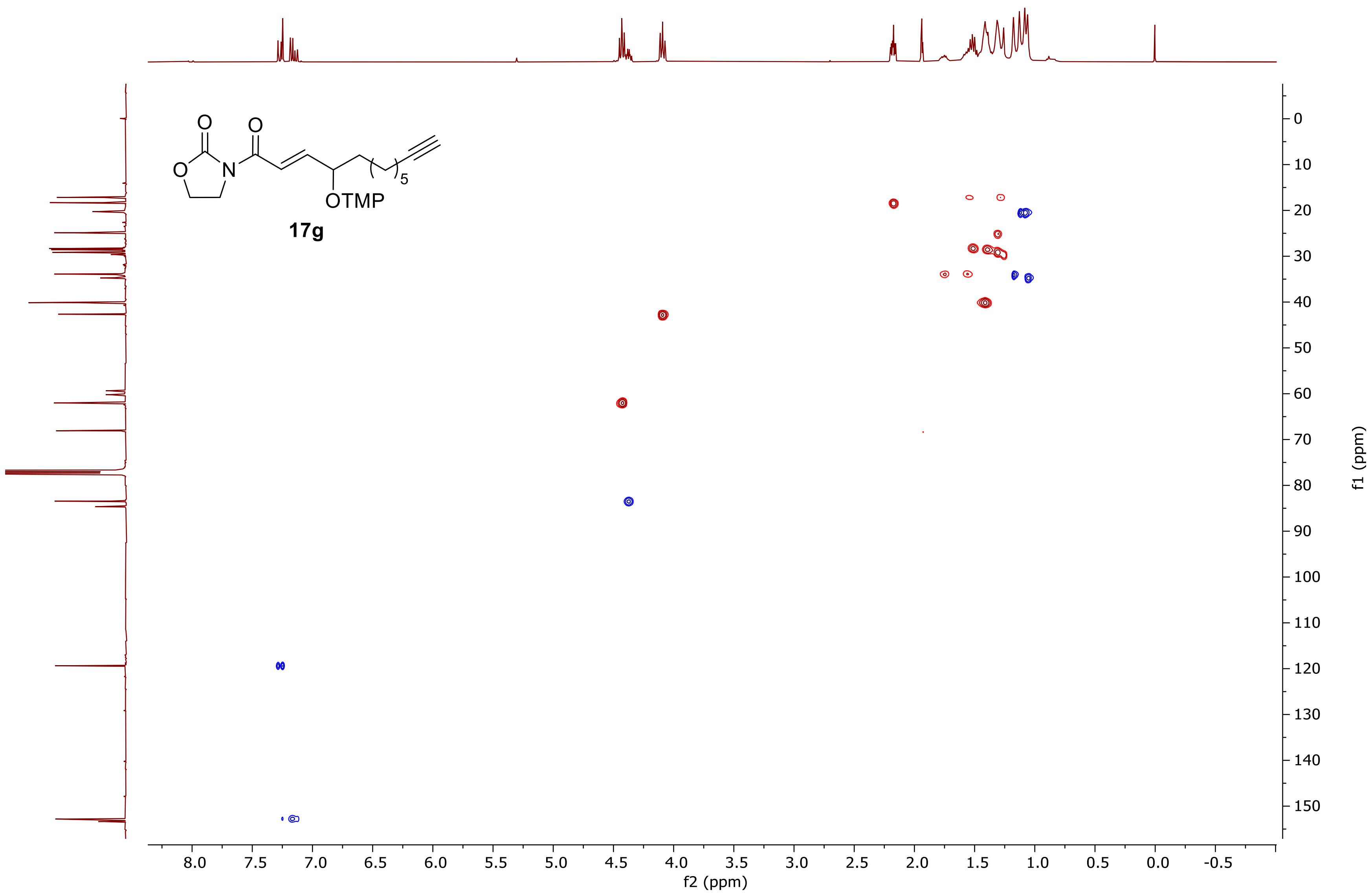

$^{1}H$ NMR (500 MHz, $CDCl_3$)

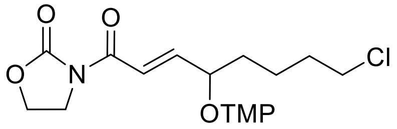


**17h**

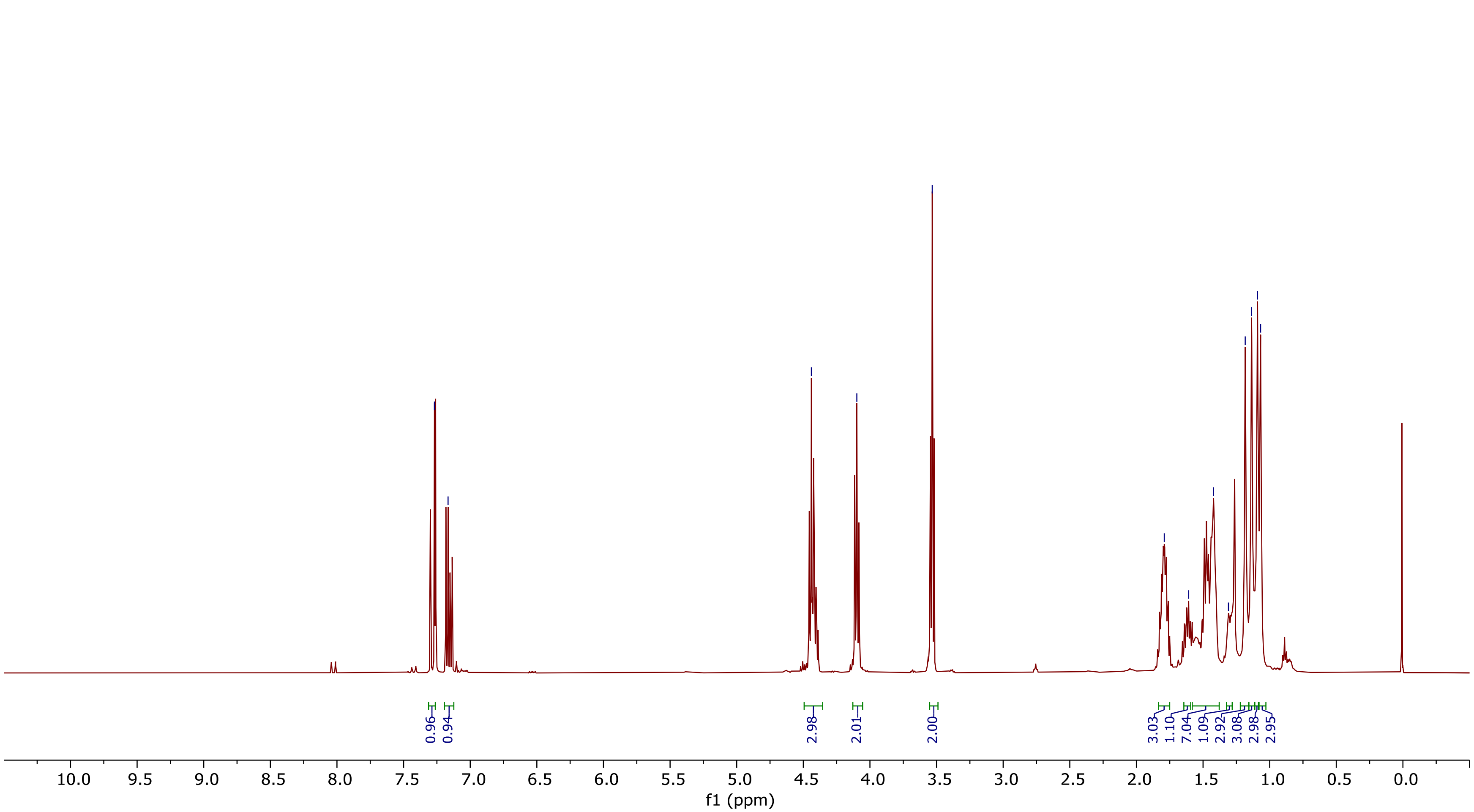

$^{13}C\{^{1}H\}$ NMR (126 MHz, $CDCl_3$)

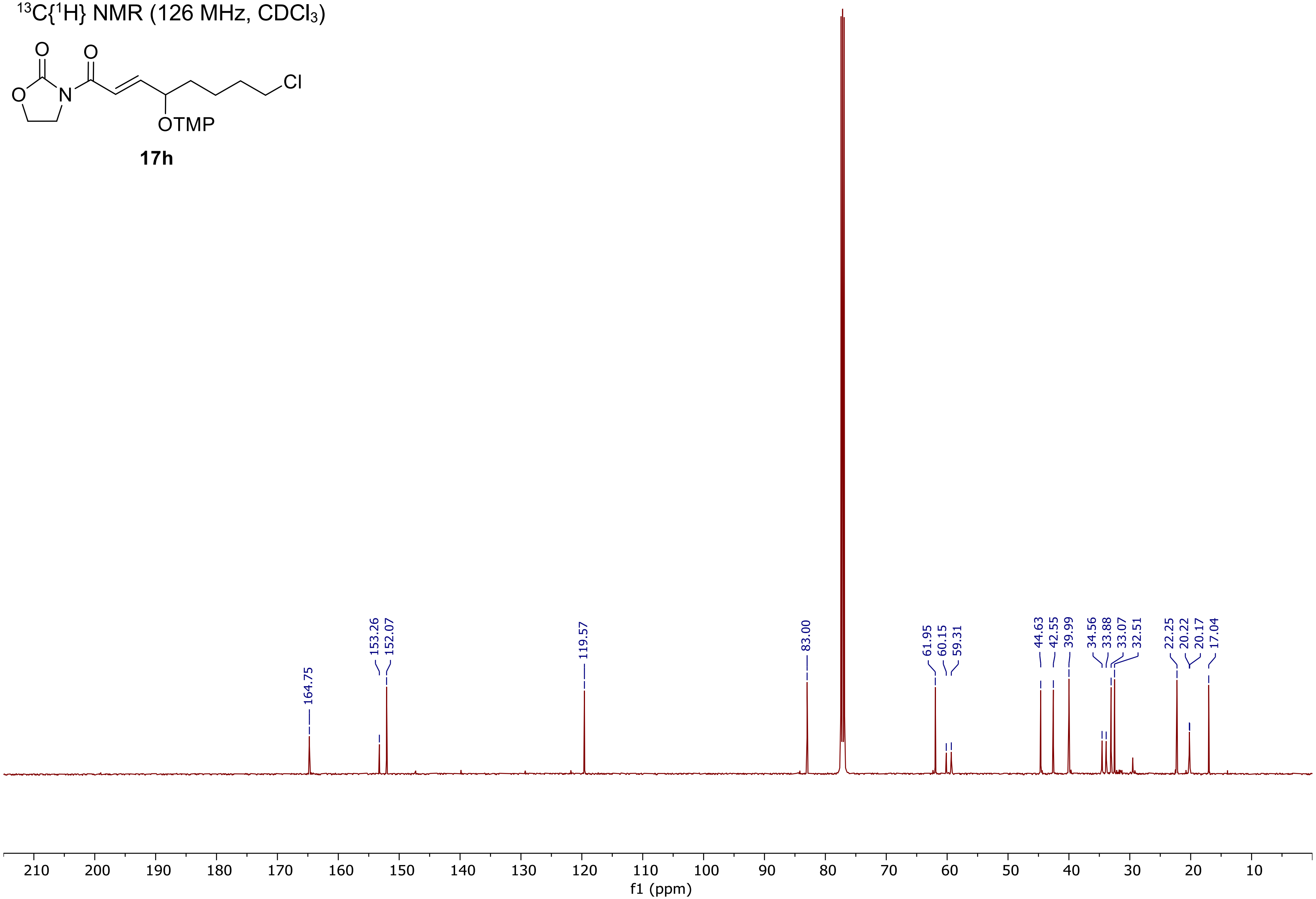


**17h**

2D $^{1}H$ -$^{1}H$ COSY (500 MHz, $CDCl_3$)

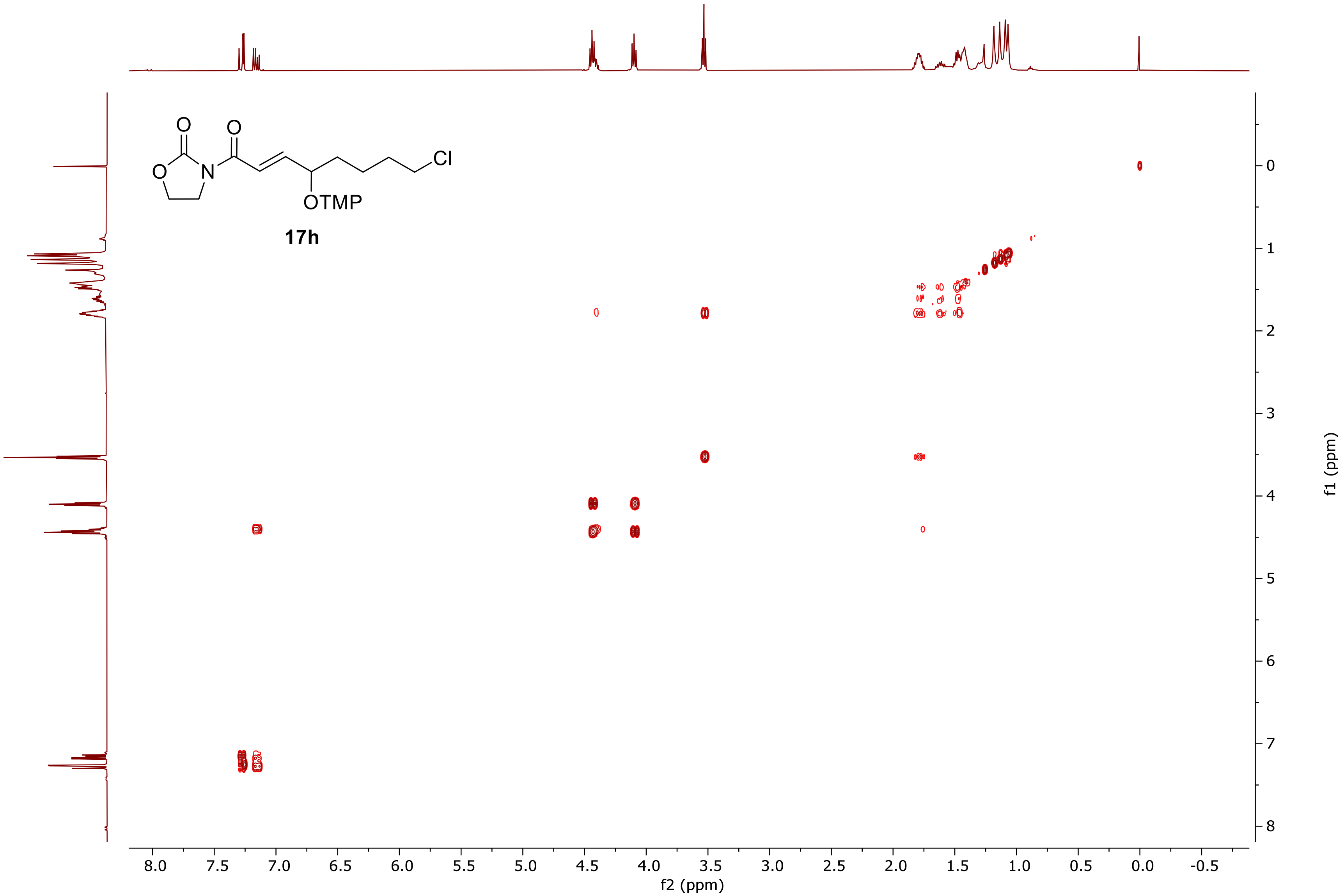

2D $^{1}H$ -$^{13}C$ HSQC (500 MHz, $CDCl_3$)

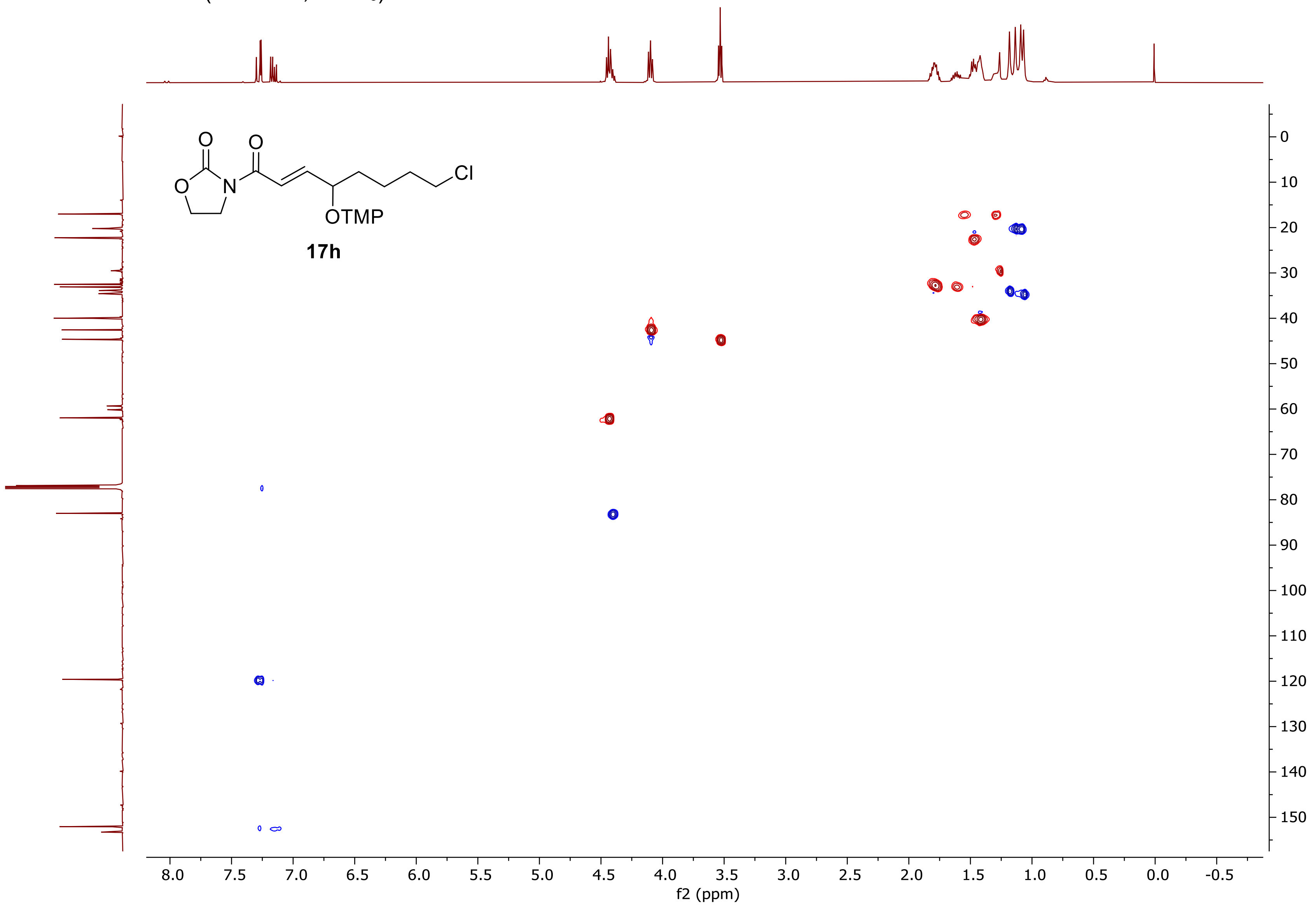

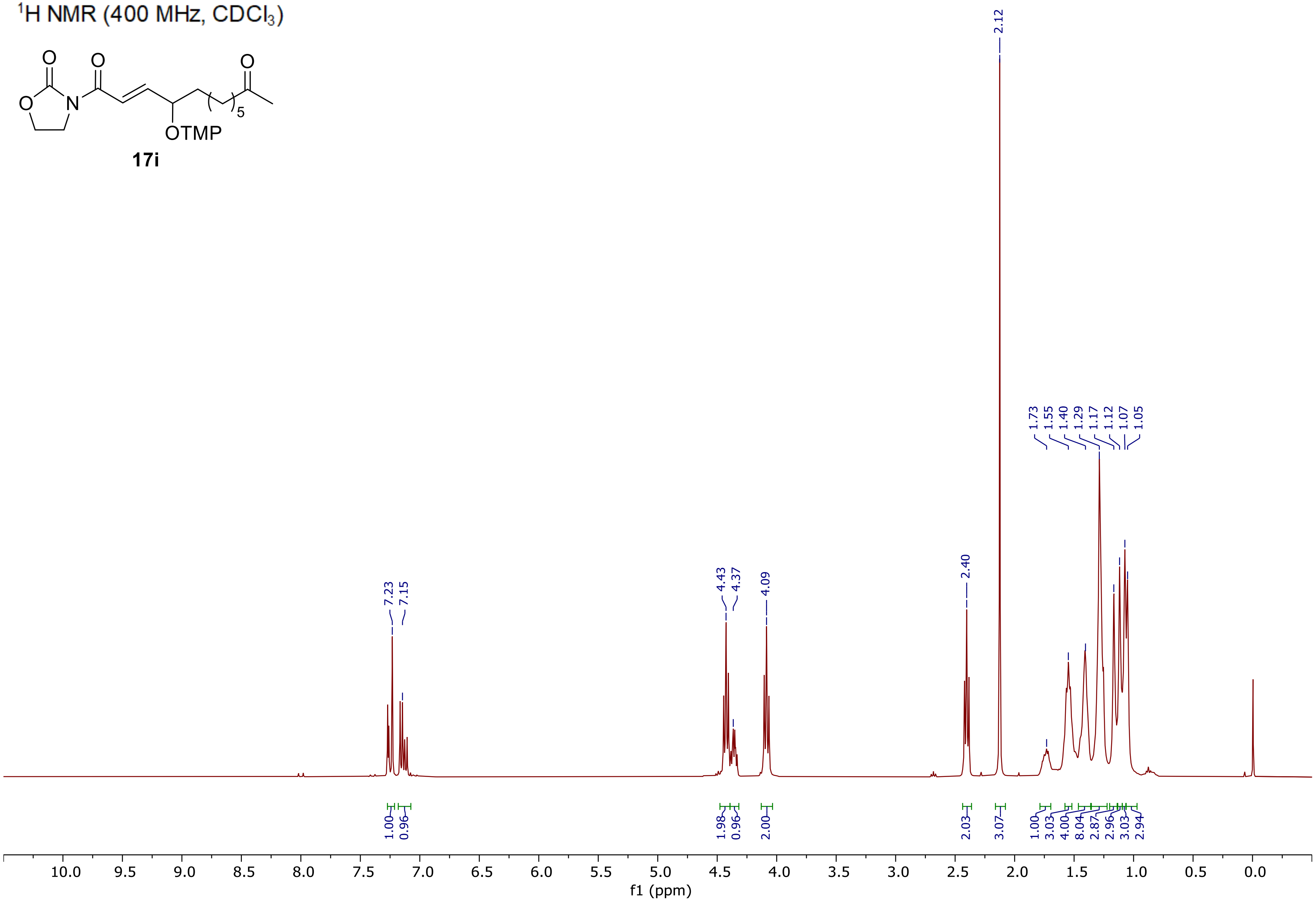

$^1$H NMR (400 MHz, CDCl$_3$)
O
O
O
O
N
5
OTMP
17i
2.12
1.73
1.55
1.40
1.29
1.17
1.12
1.07
1.05
7.23
7.15
4.43
4.37
4.09
2.40
1.00
0.96
1.98
0.96
2.00
2.03
3.07
1.00
3.03
4.00
8.04
2.87
2.96
3.03
2.94
10.0 9.5 9.0 8.5 8.0 7.5 7.0 6.5 6.0 5.5 5.0 4.5 4.0 3.5 3.0 2.5 2.0 1.5 1.0 0.5 0.0
f1 (ppm)

¹³C{¹H} NMR (101 MHz, CDCl₃)

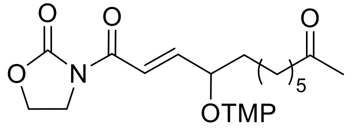


**17i**

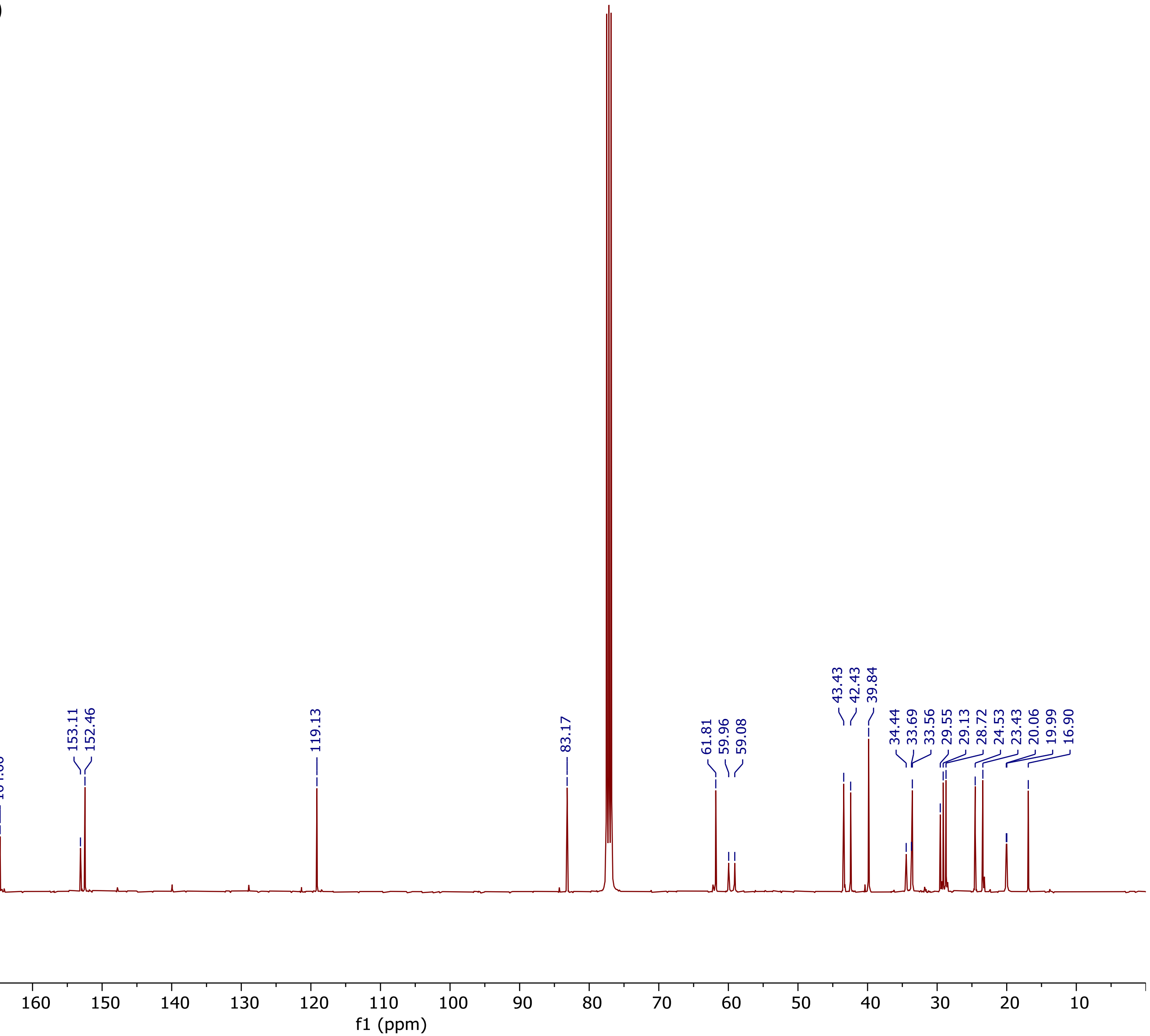

2D $^{1}H$ -$^{1}H$ COSY (400 MHz, $CDCl_3$)

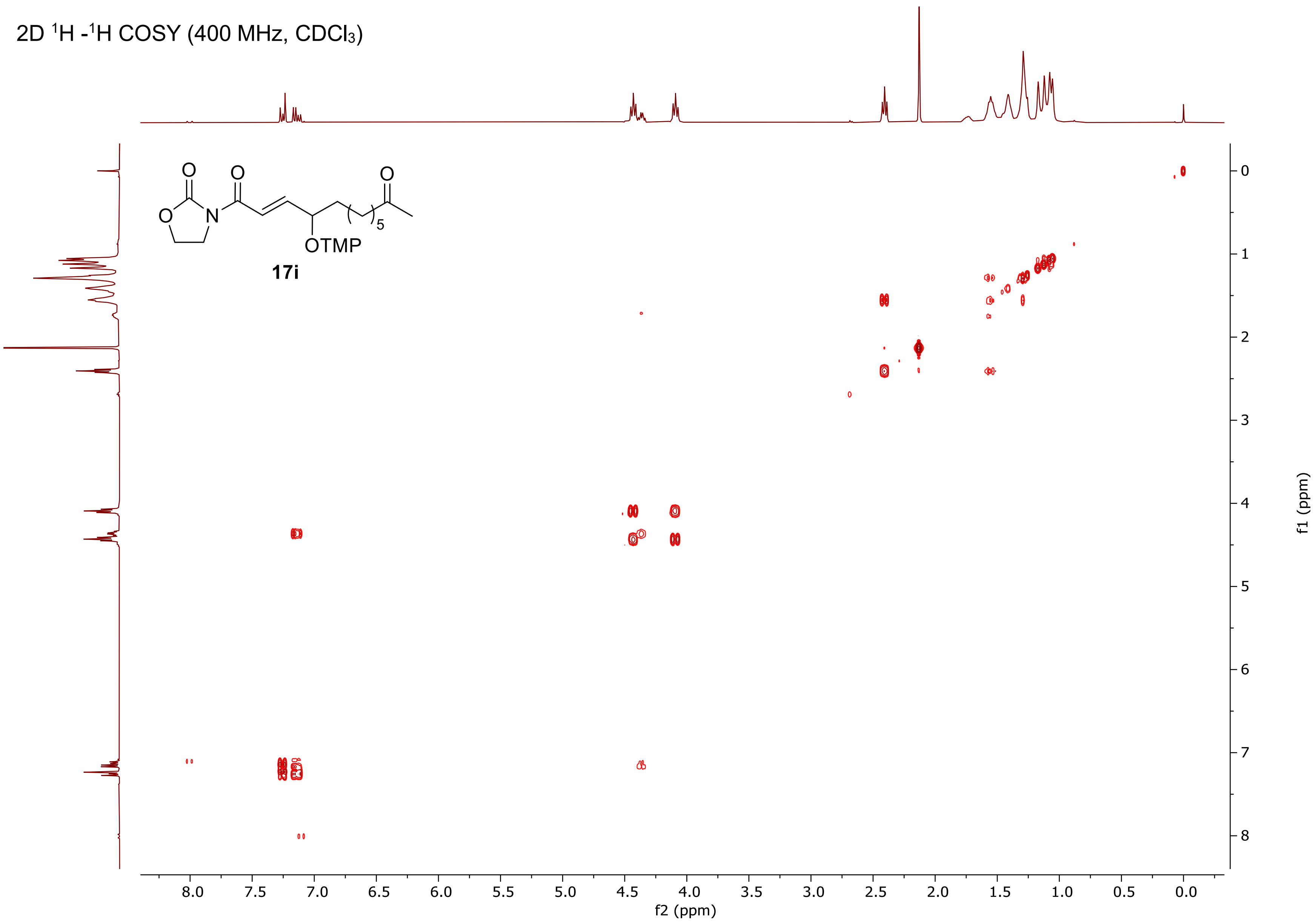

2D $^1$H -$^{13}$C HSQC (400 MHz, $CDCl_3$)

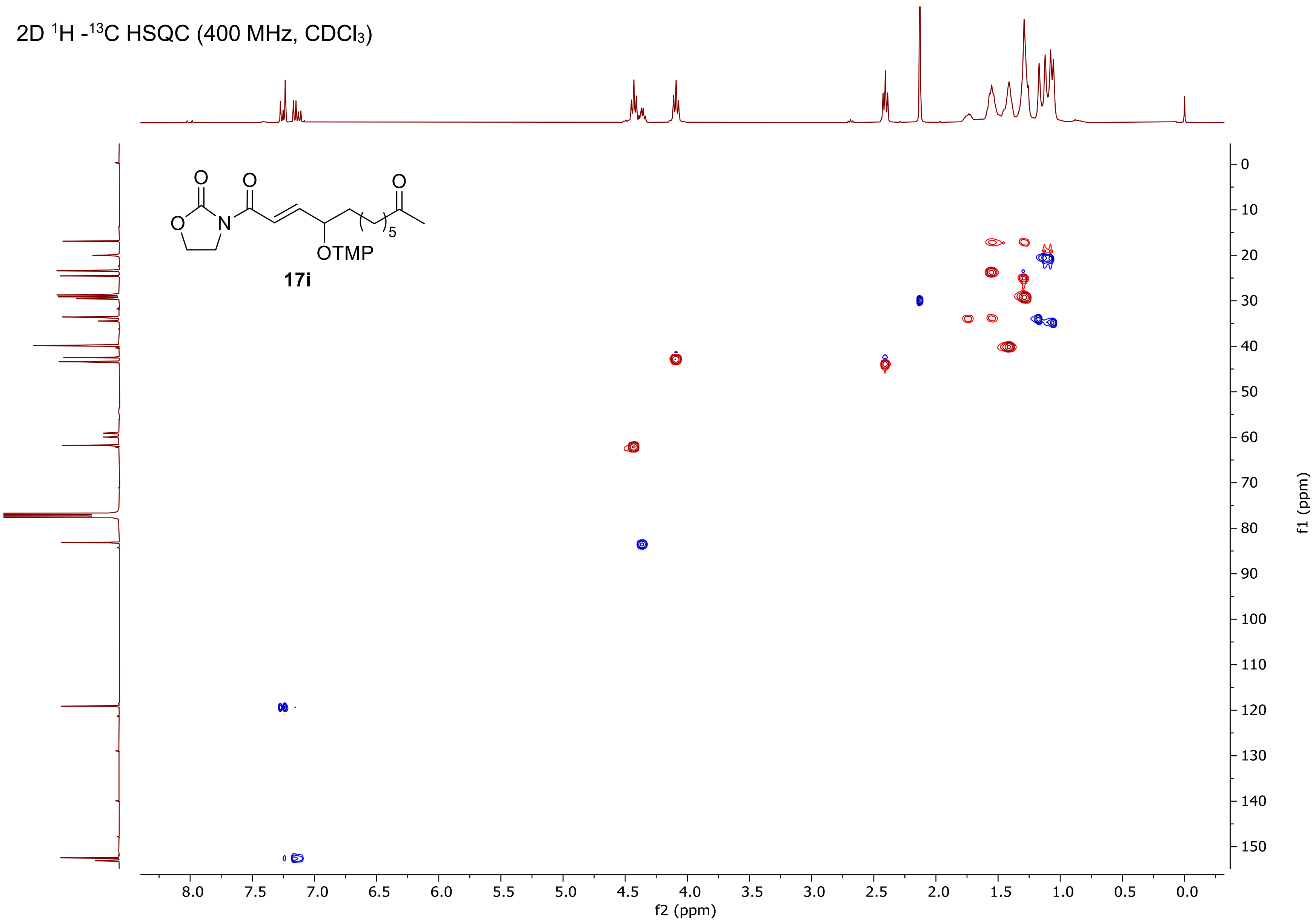

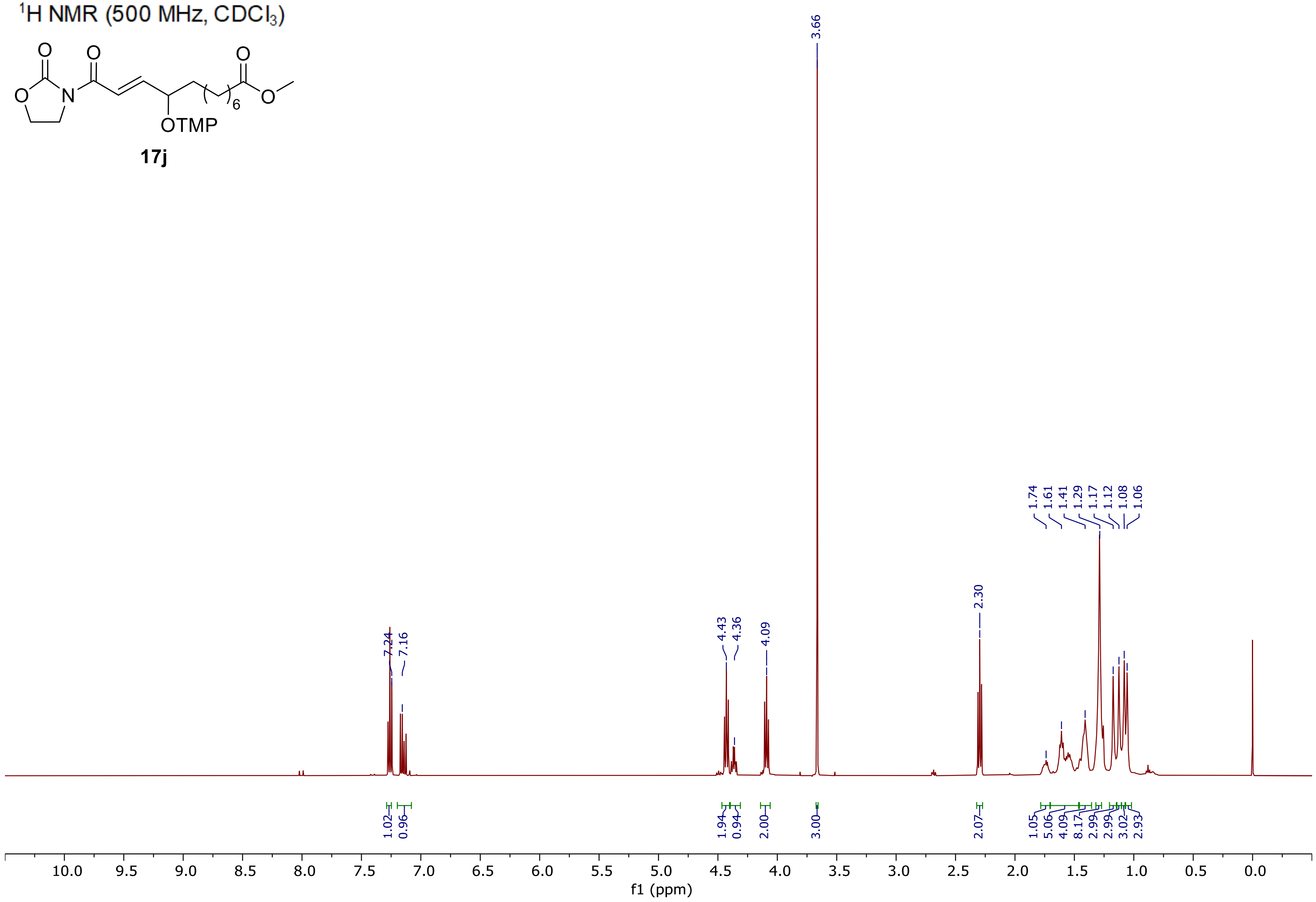

1H NMR (500 MHz, CDCl3)
O
O
O
N
O
O
6
O
OTMP
17j
3.66
1.74
1.61
1.41
1.29
1.17
1.12
1.08
1.06
2.30
4.43
4.36
4.09
7.24
7.16
1.02
0.96
1.94
0.94
2.00
3.00
2.07
1.05
5.06
4.09
8.17
2.99
2.99
3.02
2.93
10.0 9.5 9.0 8.5 8.0 7.5 7.0 6.5 6.0 5.5 5.0 4.5 4.0 3.5 3.0 2.5 2.0 1.5 1.0 0.5 0.0
f1 (ppm)

$^{13}C\{^{1}H\}$ NMR (126 MHz, $CDCl_3$)

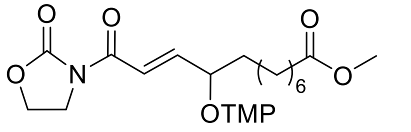


**17j**

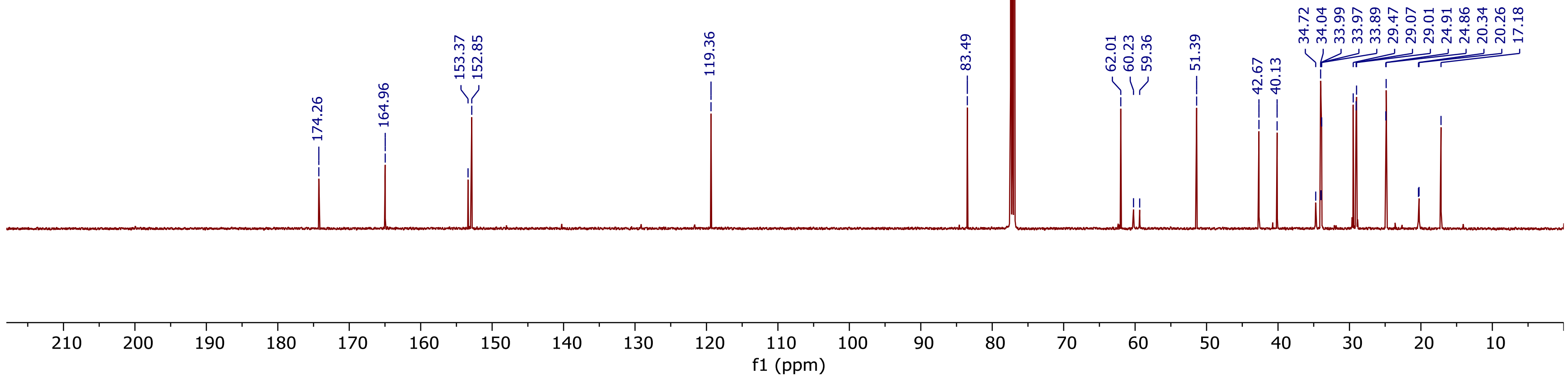

2D $^{1}H$ -$^{1}H$ COSY (500 MHz, $CDCl_3$)

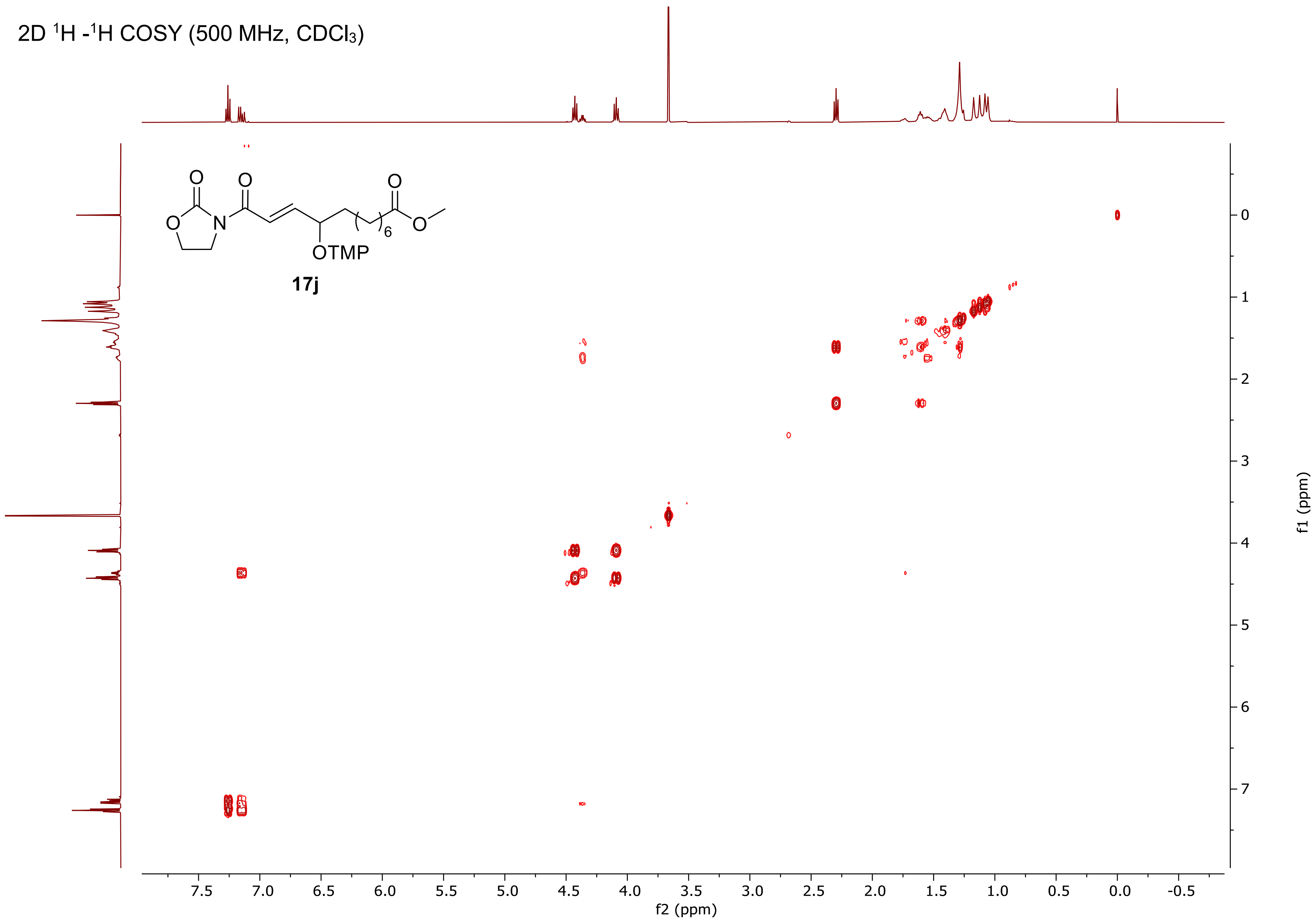

2D $^{1}H$ -$^{13}C$ HSQC (500 MHz, $CDCl_3$)

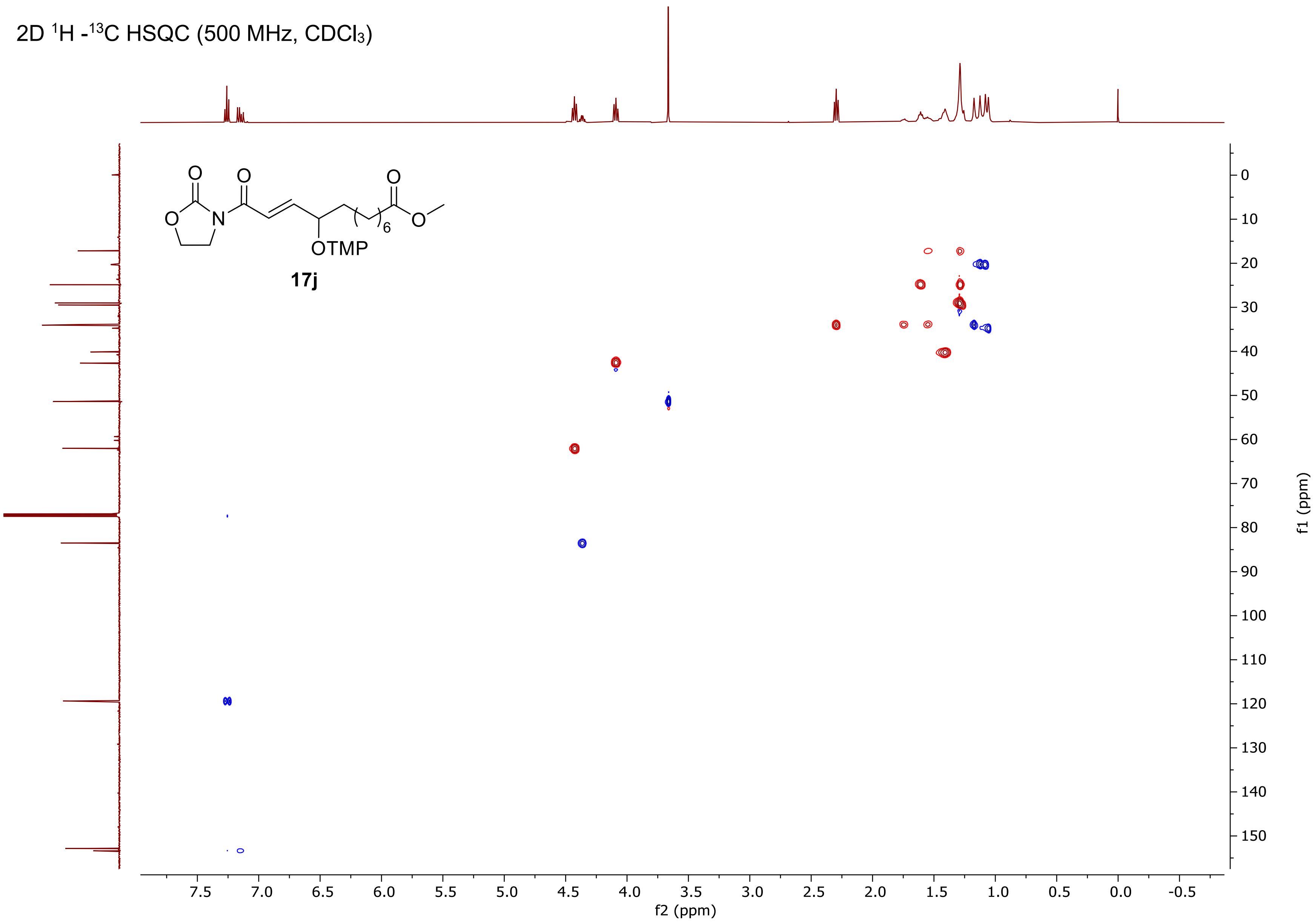

$^{1}$H NMR (400 MHz, $CDCl_3$)

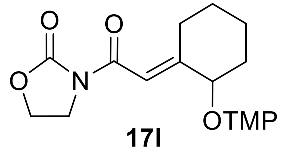

**17l**

22% of *Z* isomer present

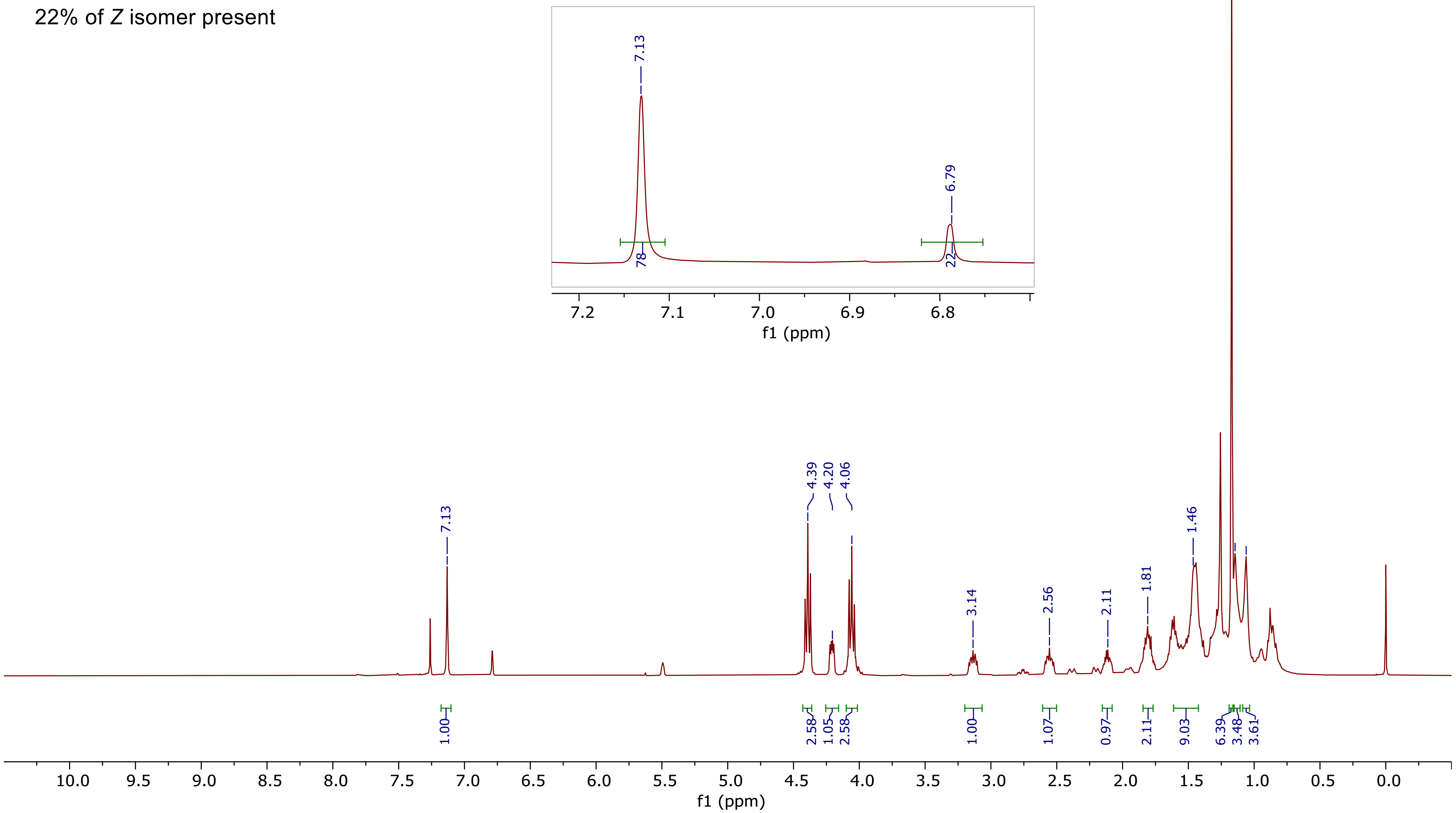

2D $^{1}H$-$^{1}H$ NOESY (400 MHz, $CDCl_3$)

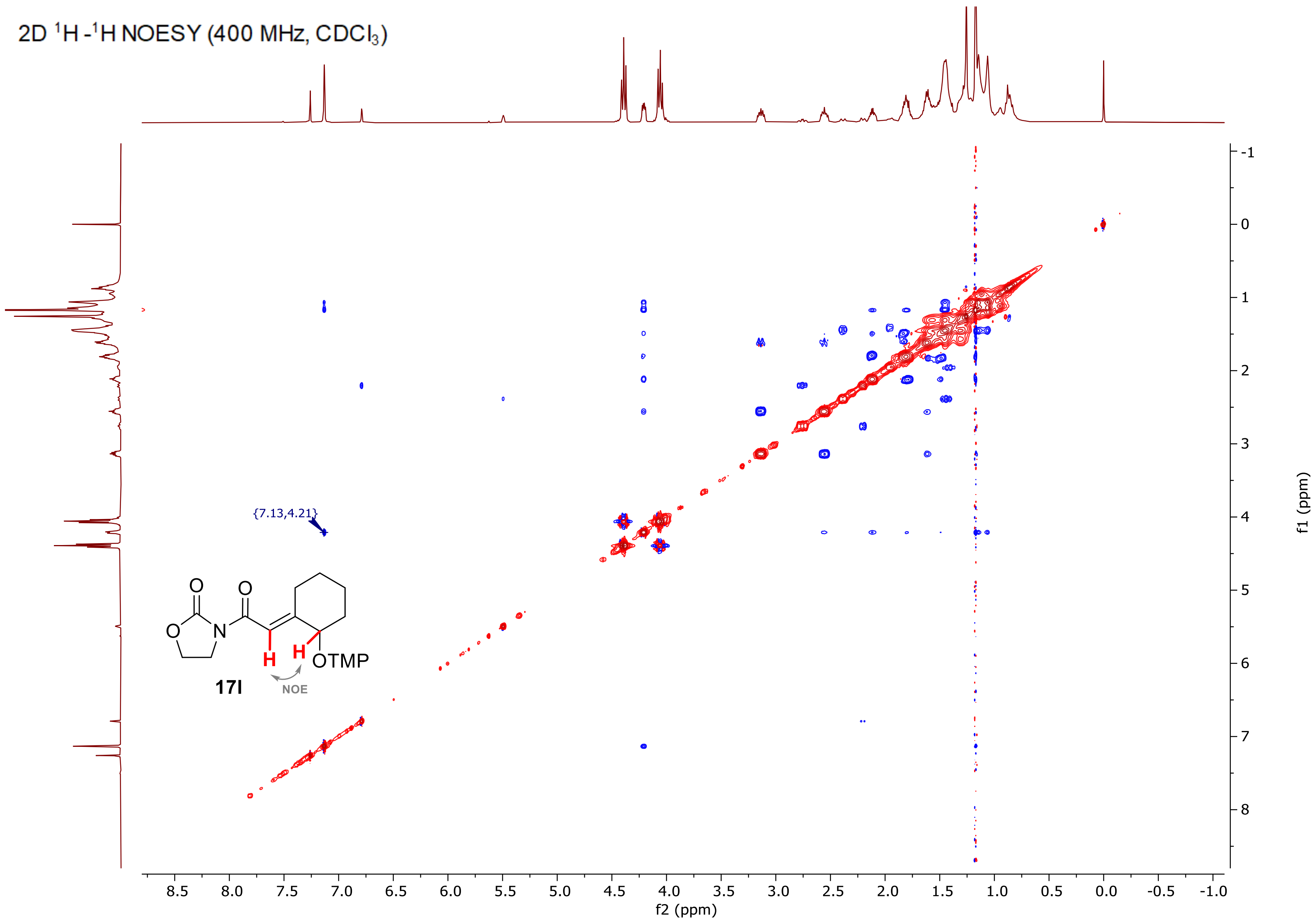

$^{13}C\{^{1}H\}$ NMR (101 MHz, $CDCl_3$)

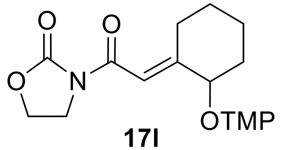


**17l**

22% of *Z* isomer present

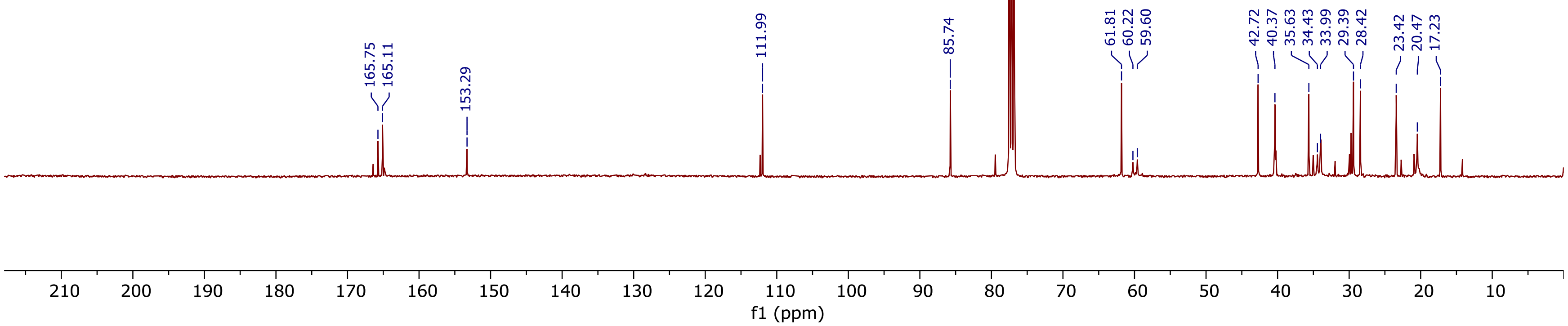

2D $^1$H -$^1$H COSY (400 MHz, $CDCl_3$)

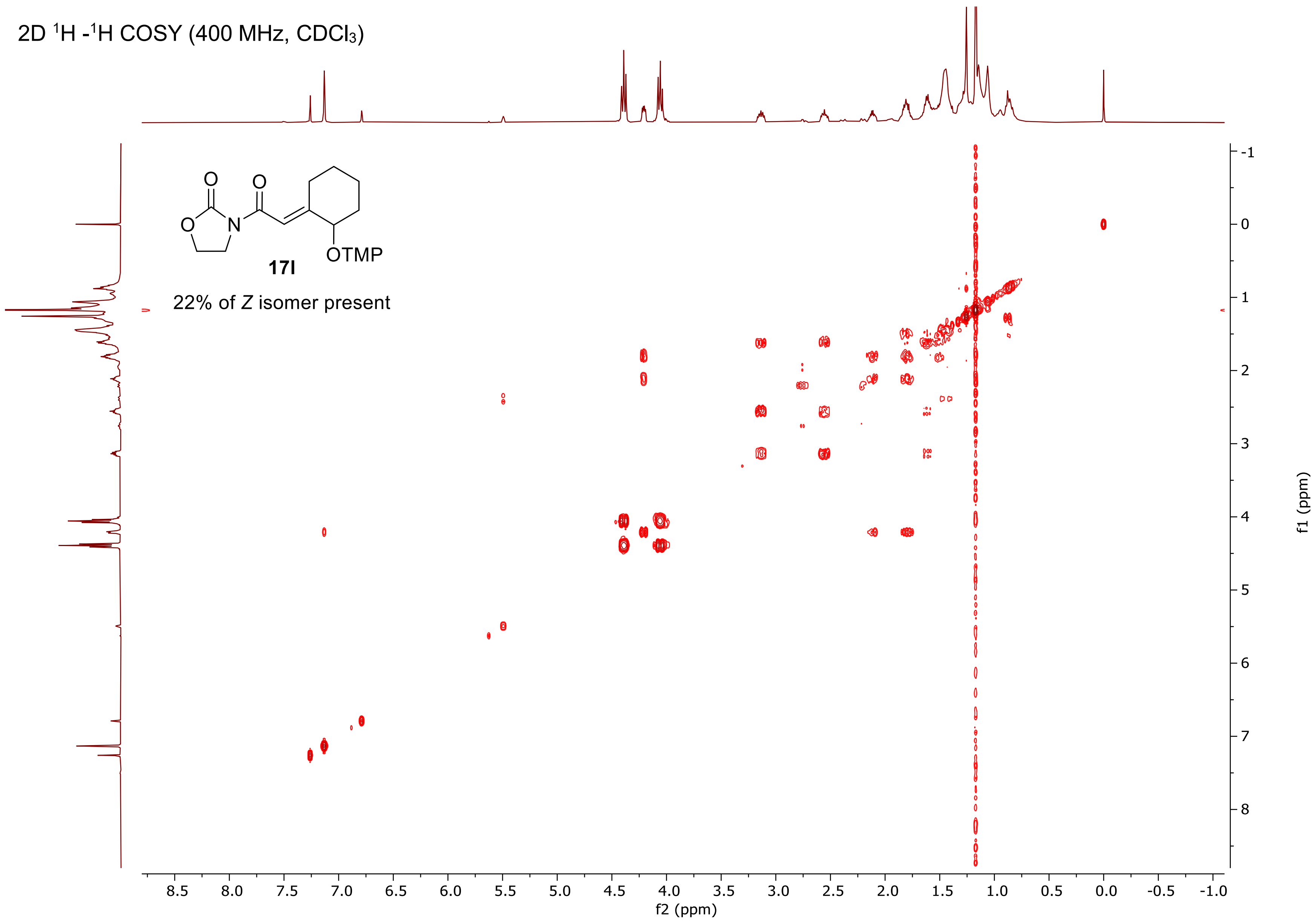

2D $^{1}H$ -$^{13}C$ HSQC (400 MHz, $CDCl_3$)

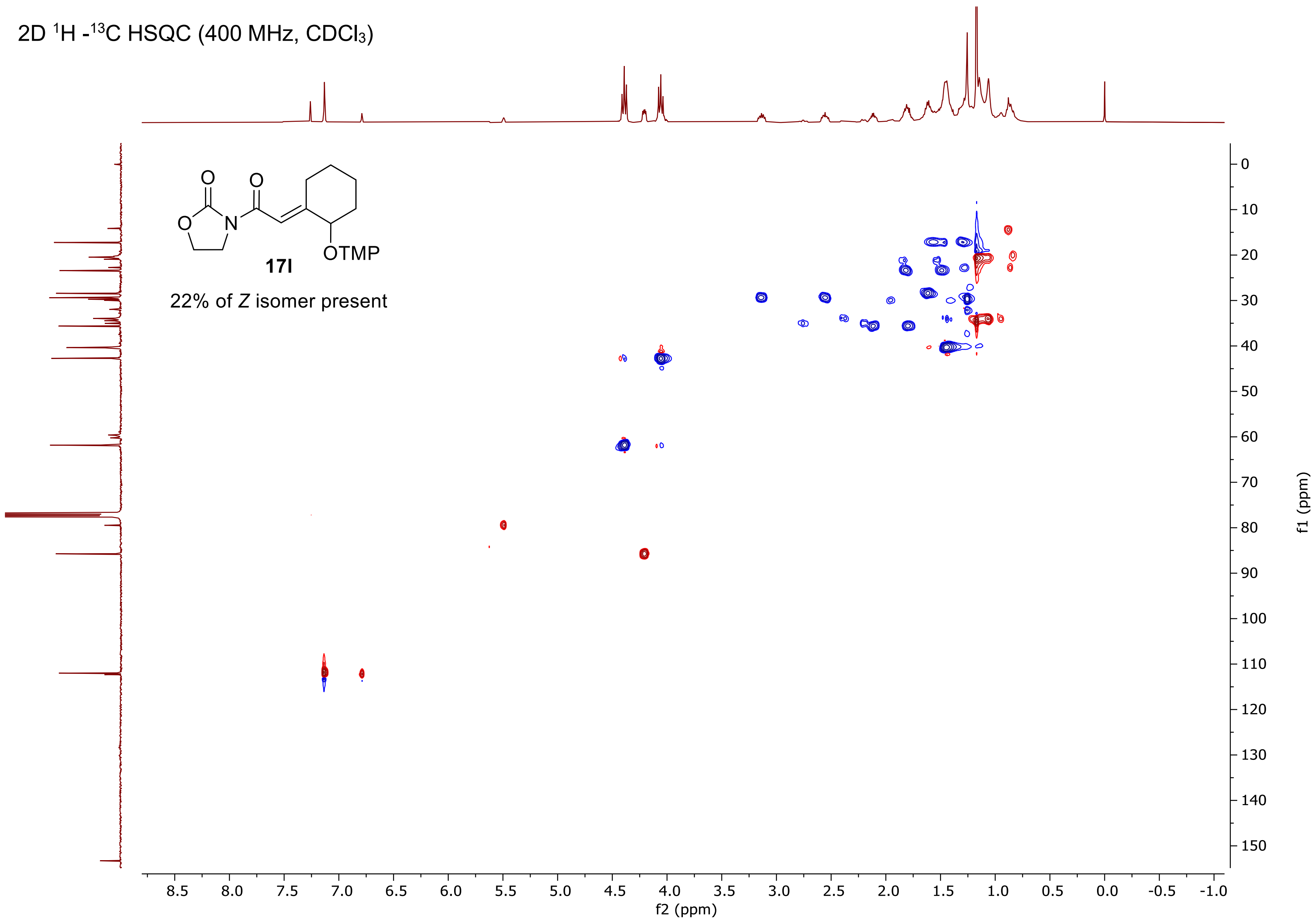

$^{1}H$ NMR (400 MHz, $CDCl_3$)

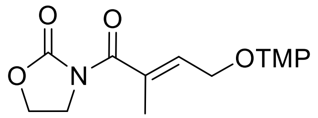


**17m**

8% of *Z* isomer present

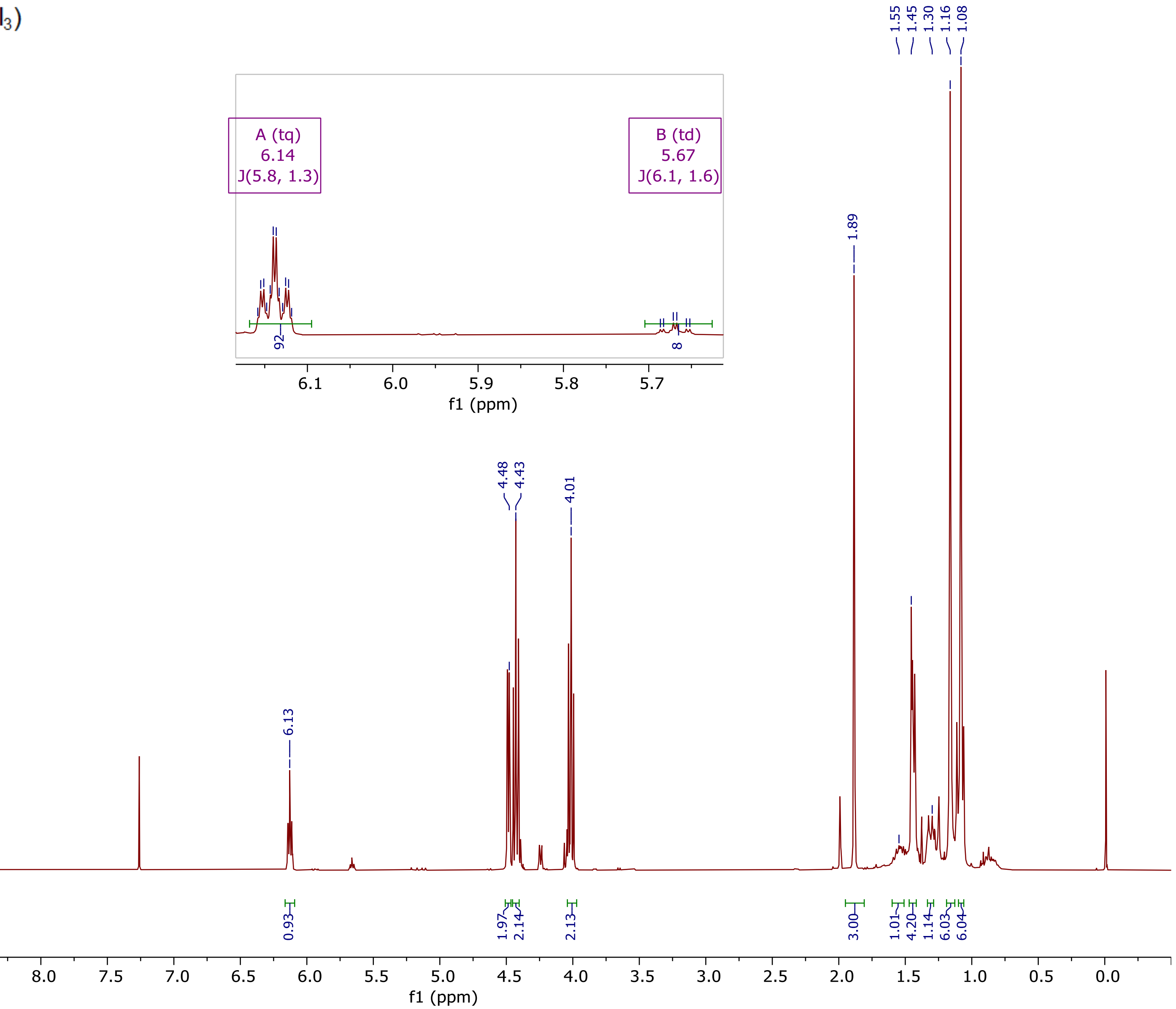

2D $^{1}H$ -$^{1}H$ NOESY (400 MHz, $CDCl_3$)

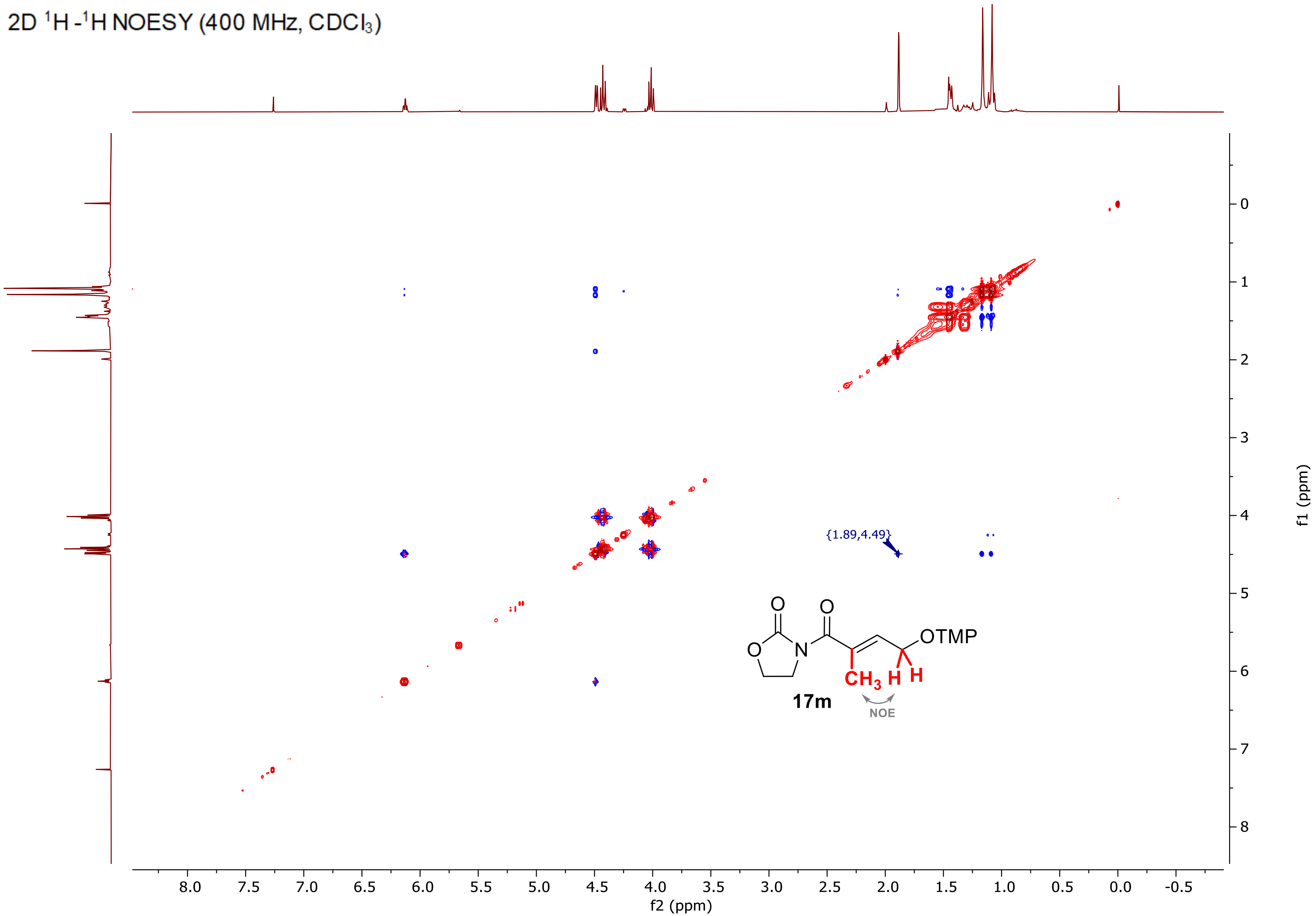

$^{13}C\{^{1}H\}$ NMR (101 MHz, $CDCl_3$)

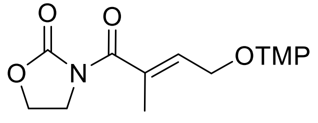

**17m**

8% of *Z* isomer present

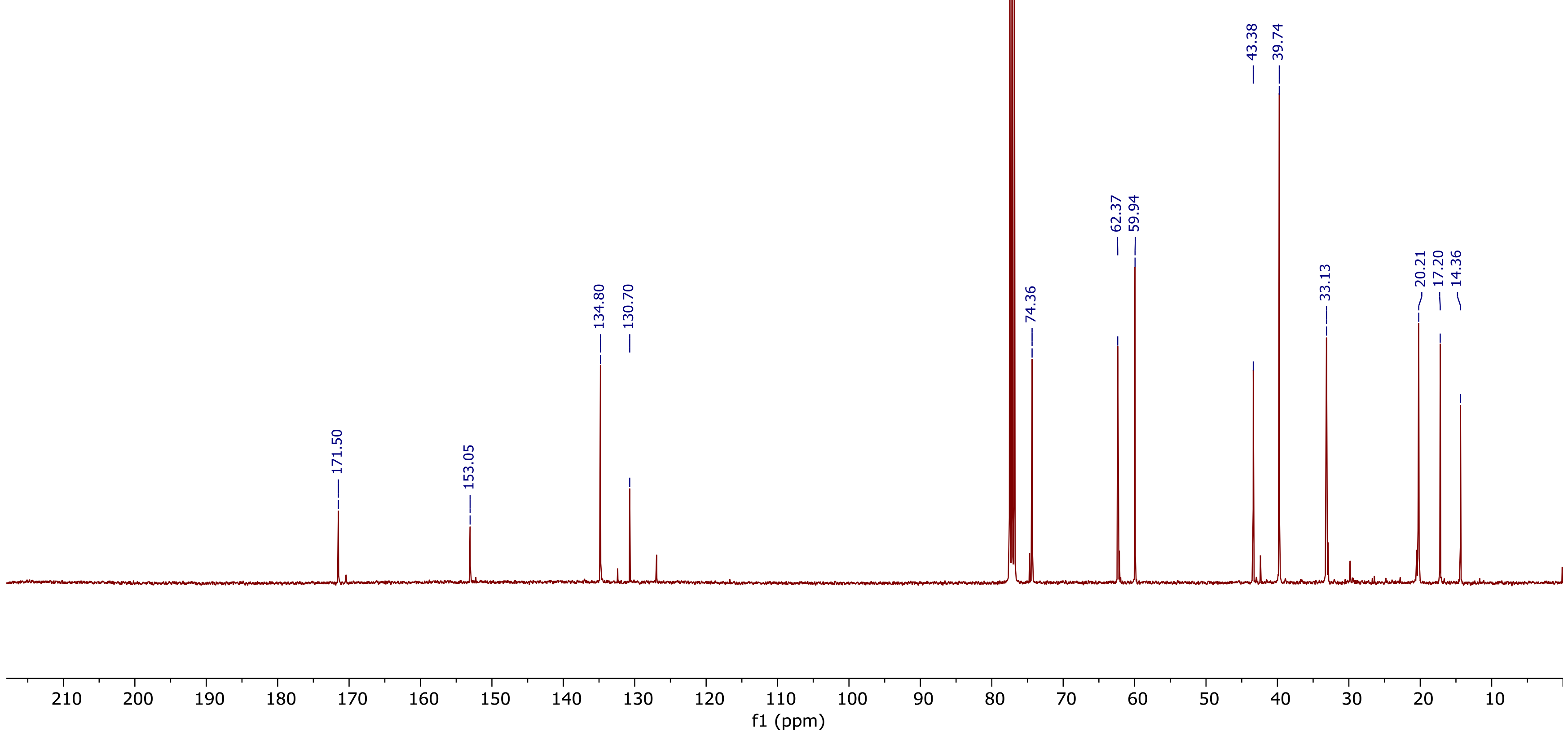

2D $^{1}H$ -$^{1}H$ COSY (400 MHz, $CDCl_3$)

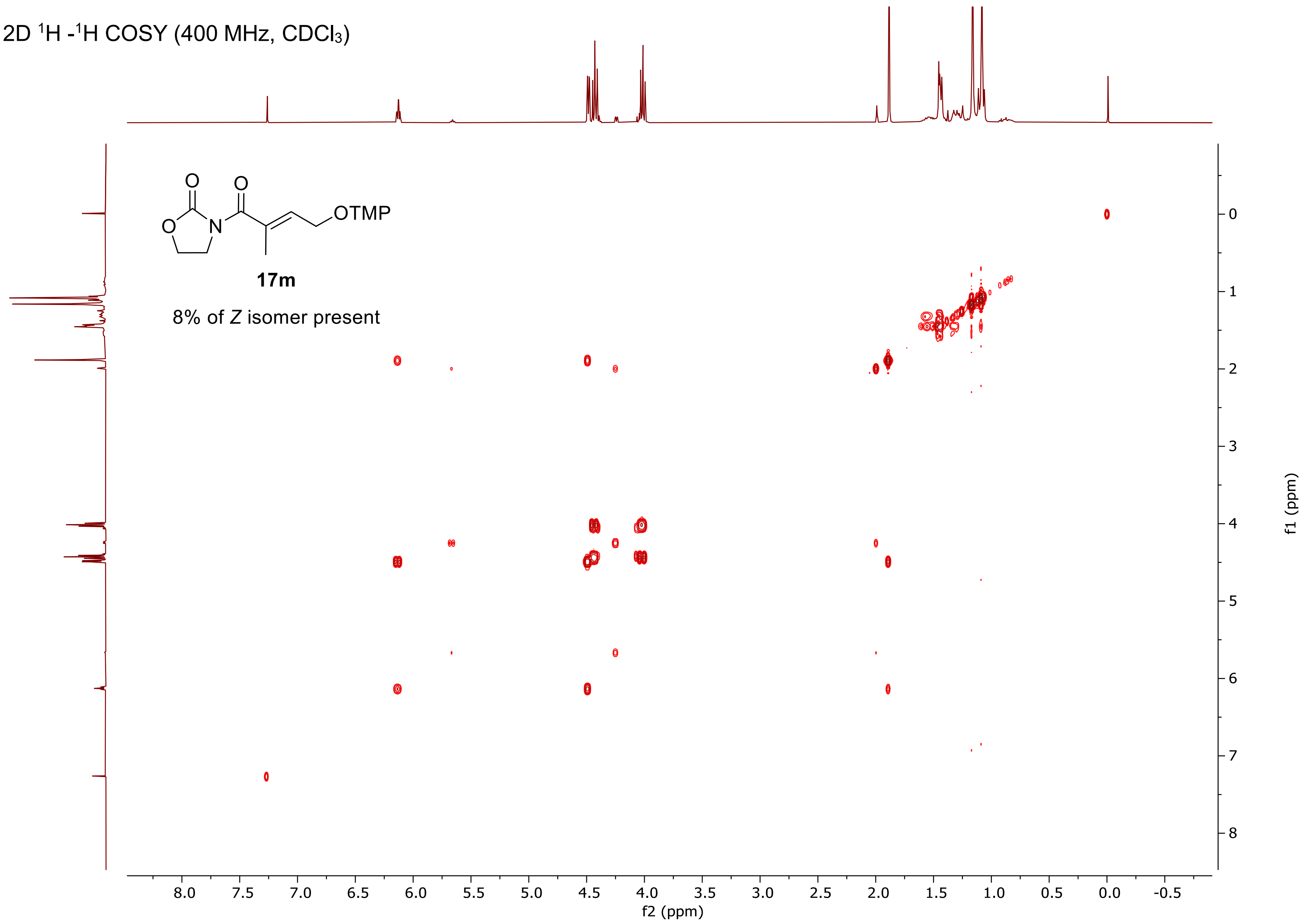

2D $^1$H -$^{13}$C HSQC (400 MHz, $CDCl_3$)

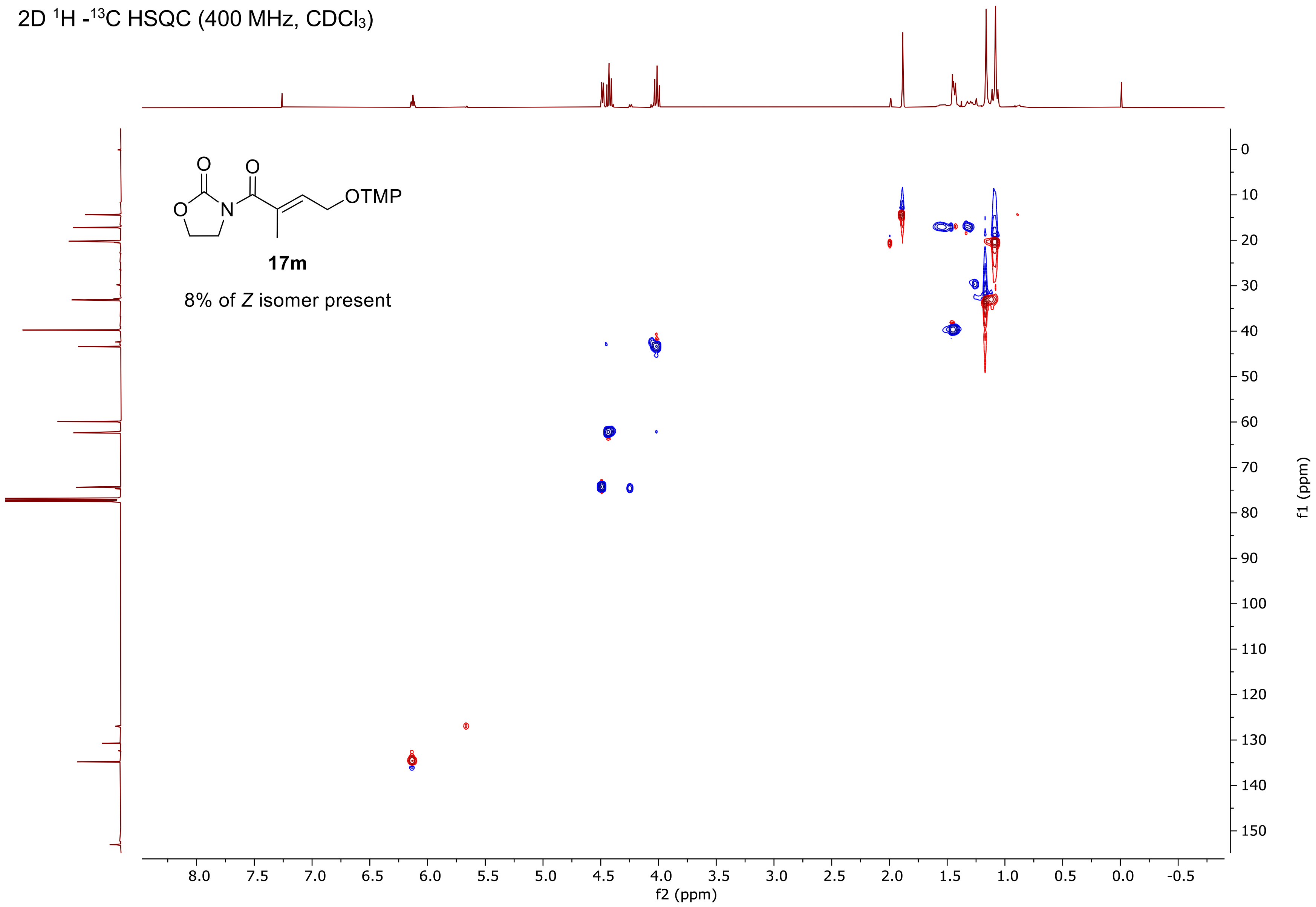

[1]H NMR (400 MHz, CDCl$_3$)

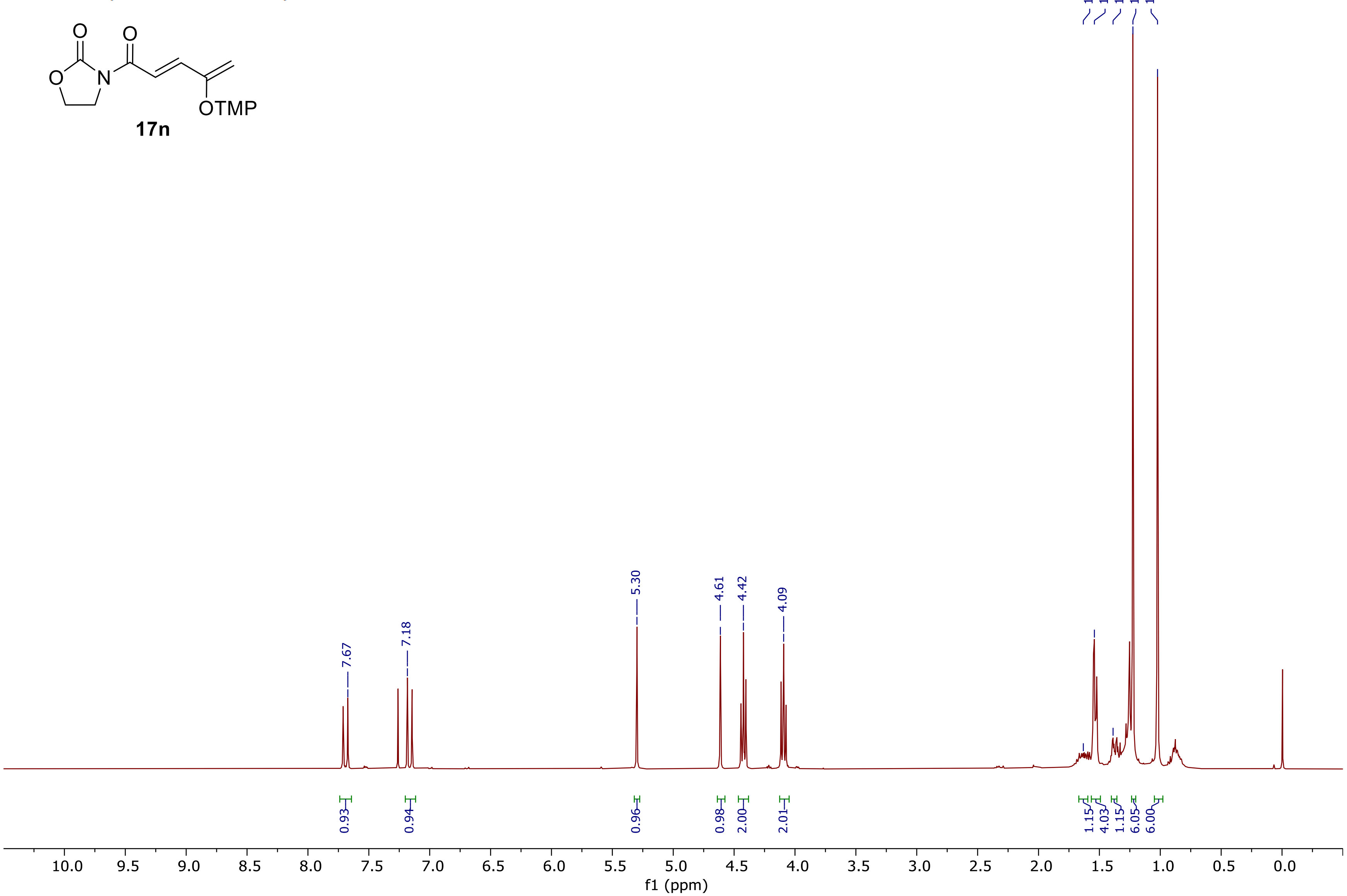


**17n**

$^{13}C\{^{1}H\}$ NMR (101 MHz, $CDCl_3$)

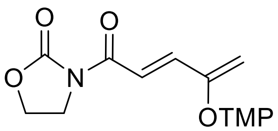


**17n**

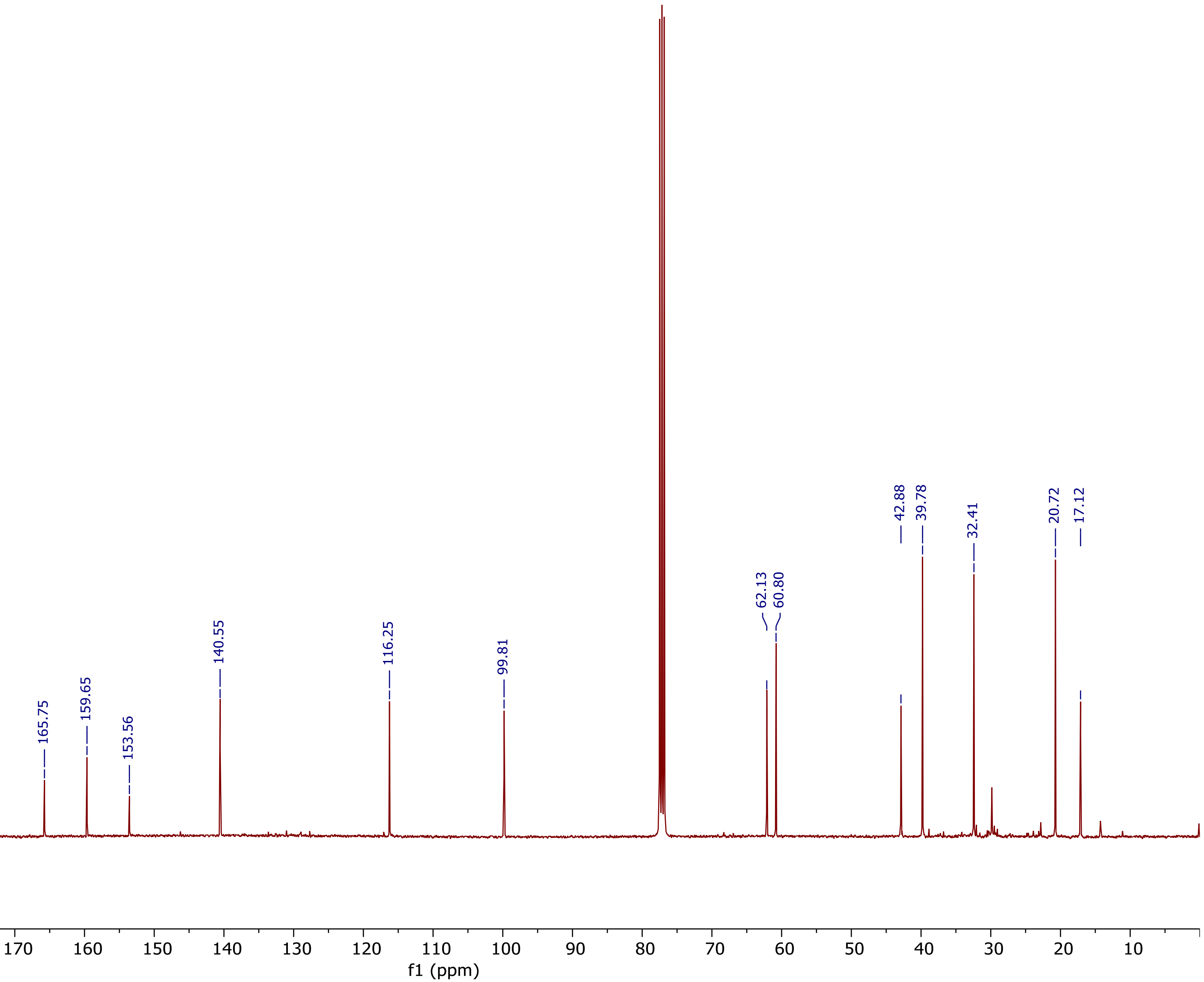

2D $^1H$ -$^1H$ COSY (400 MHz, $CDCl_3$)

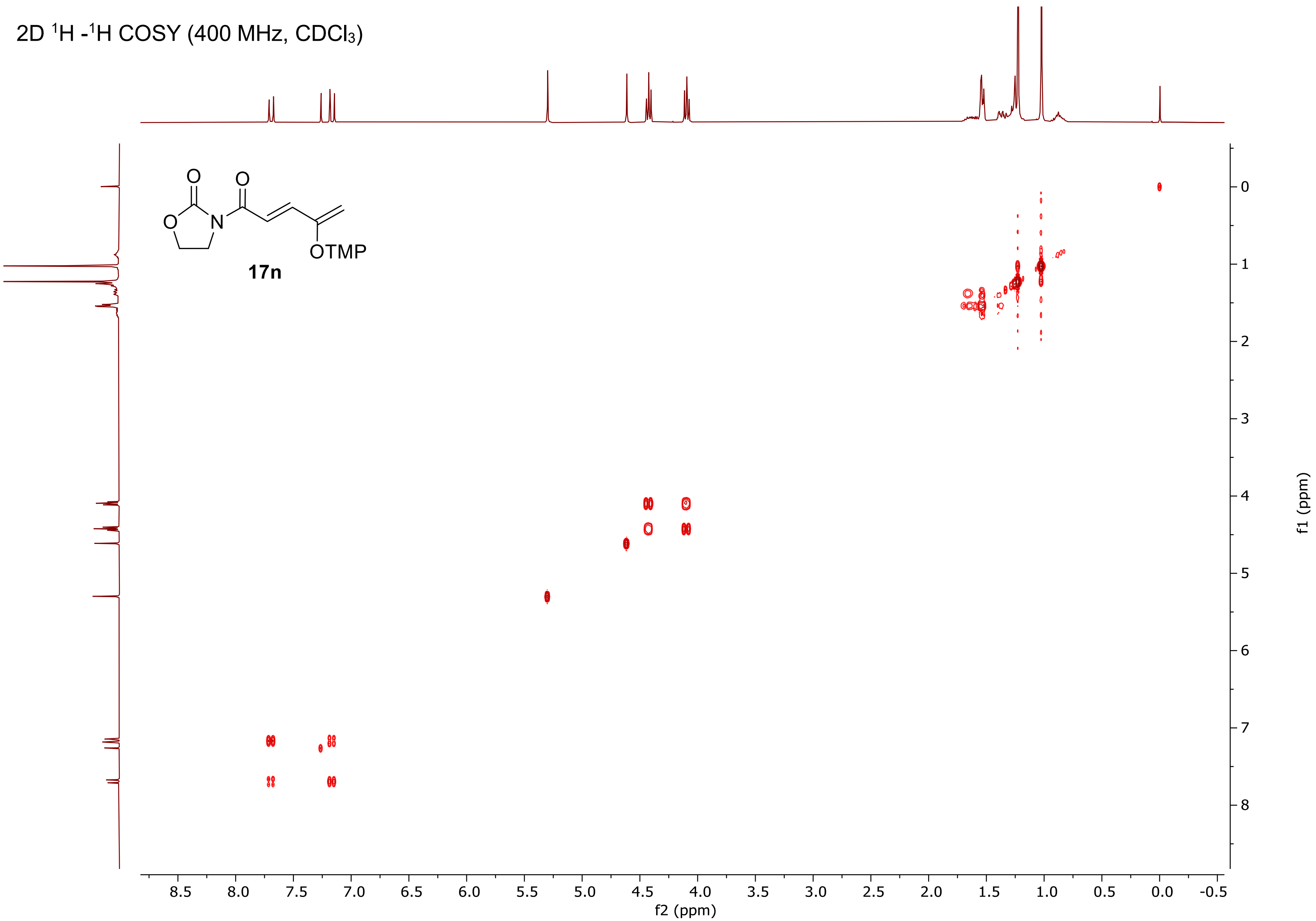

2D $^{1}H$ -$^{13}C$ HSQC (400 MHz, $CDCl_3$)

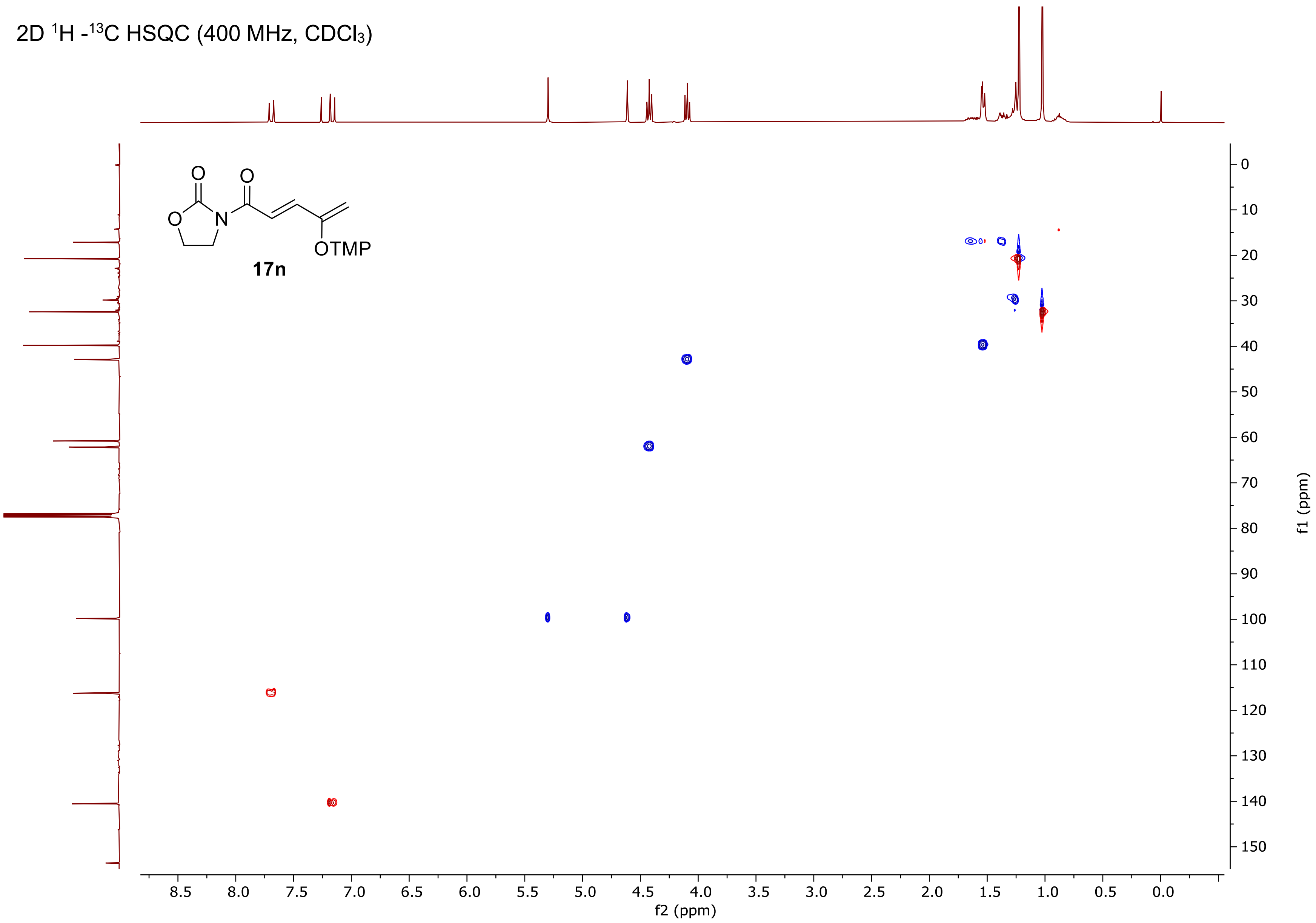

$^{1}H$ NMR (400 MHz, $CDCl_3$)

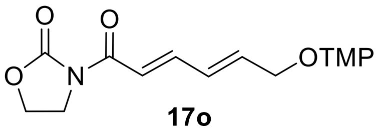


17o

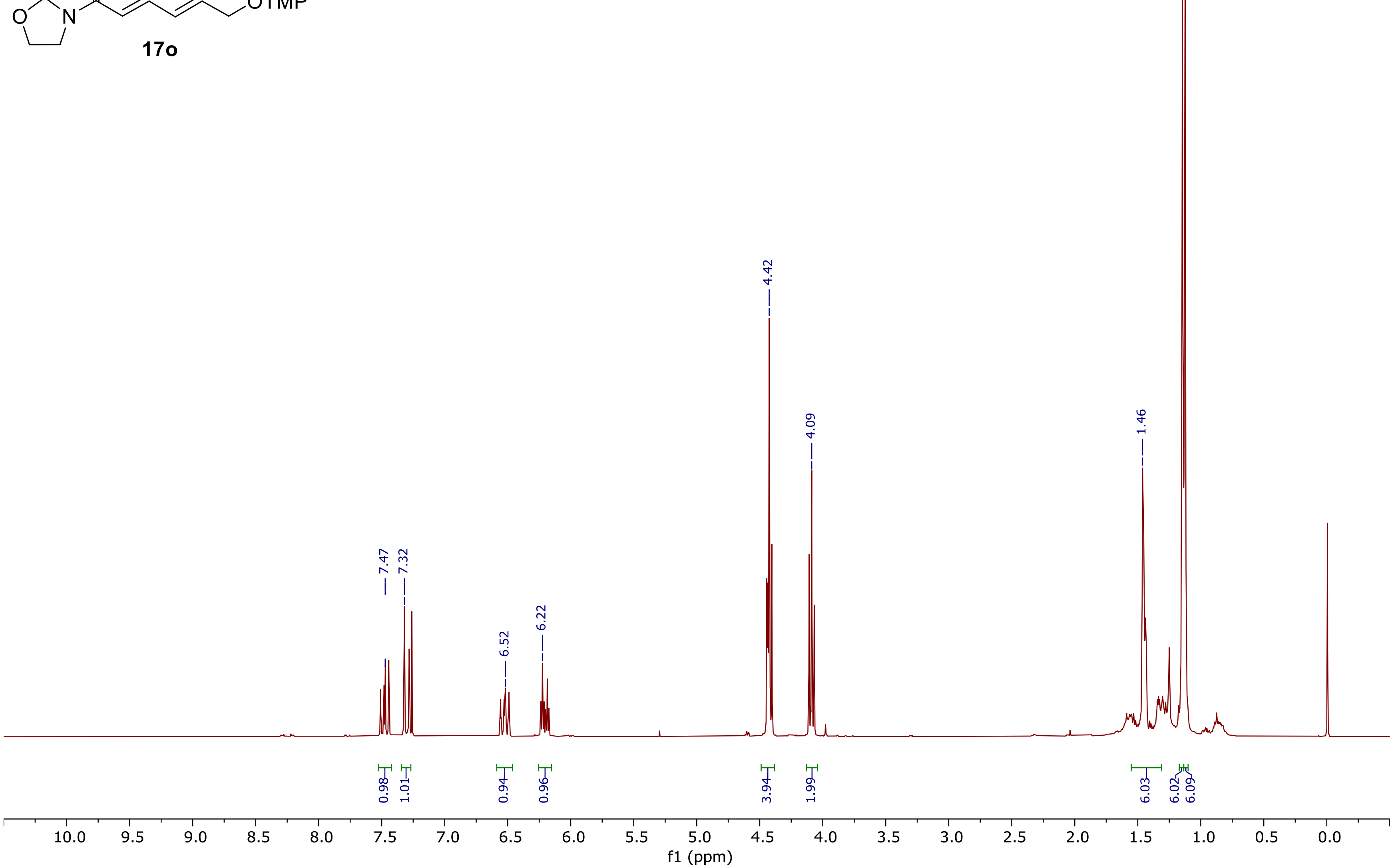

$^{13}C\{^{1}H\}$ NMR (101 MHz, $CDCl_3$)

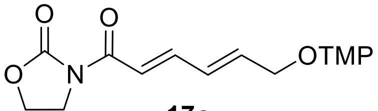


**17o**

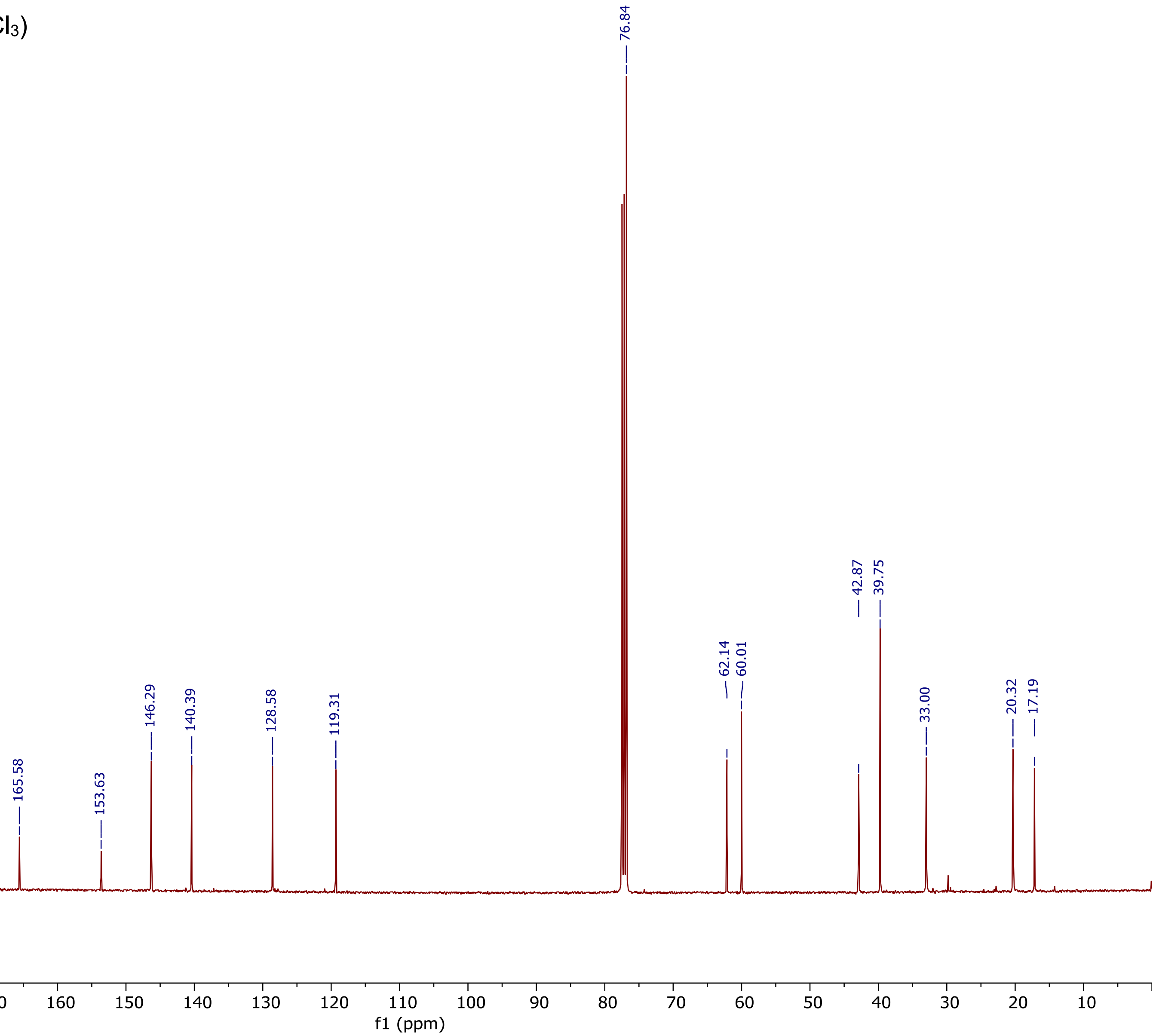

2D $^{1}H$ -$^{1}H$ COSY (400 MHz, $CDCl_3$)

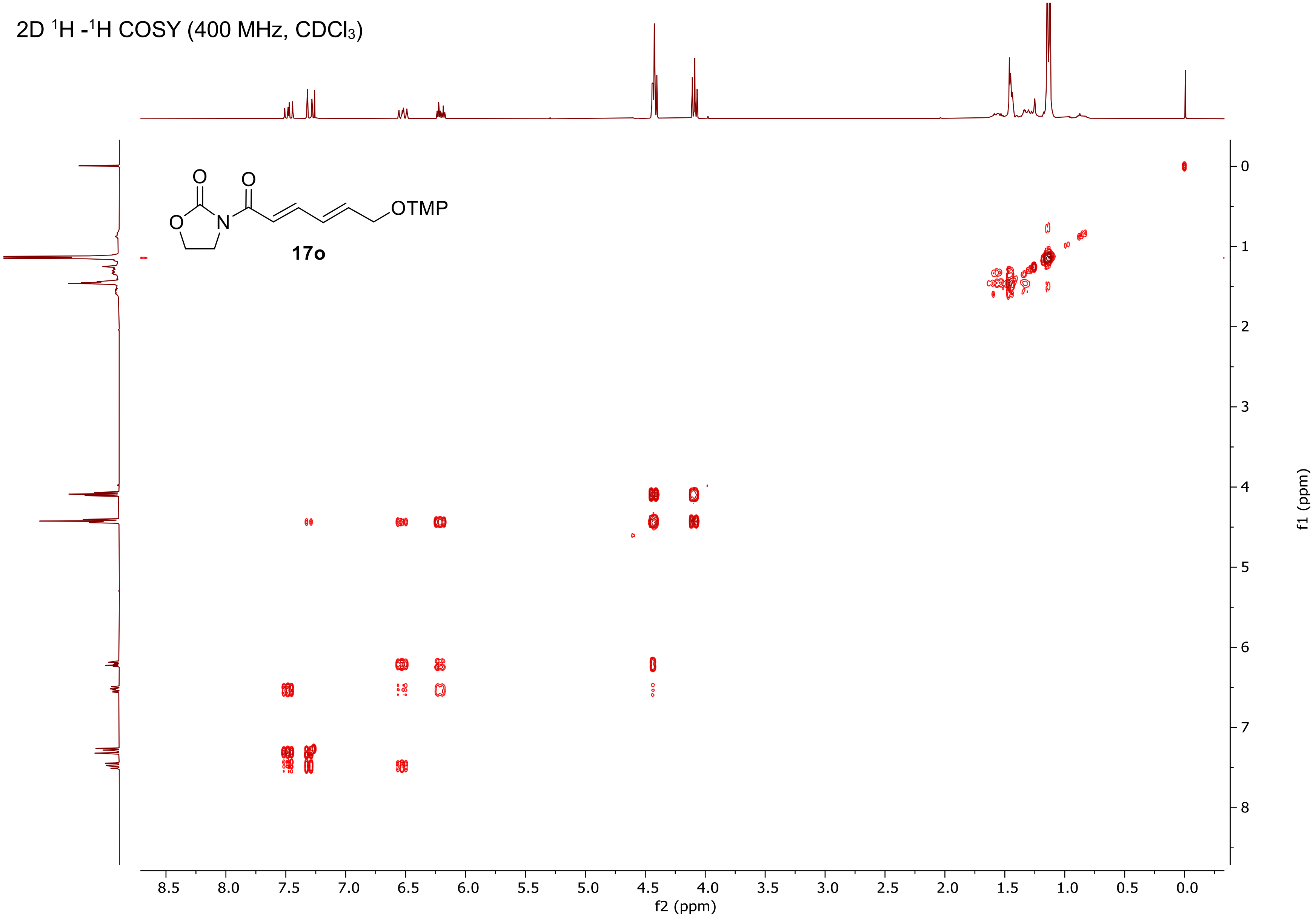

2D $^{1}H$ -$^{13}C$ HSQC (400 MHz, $CDCl_3$)

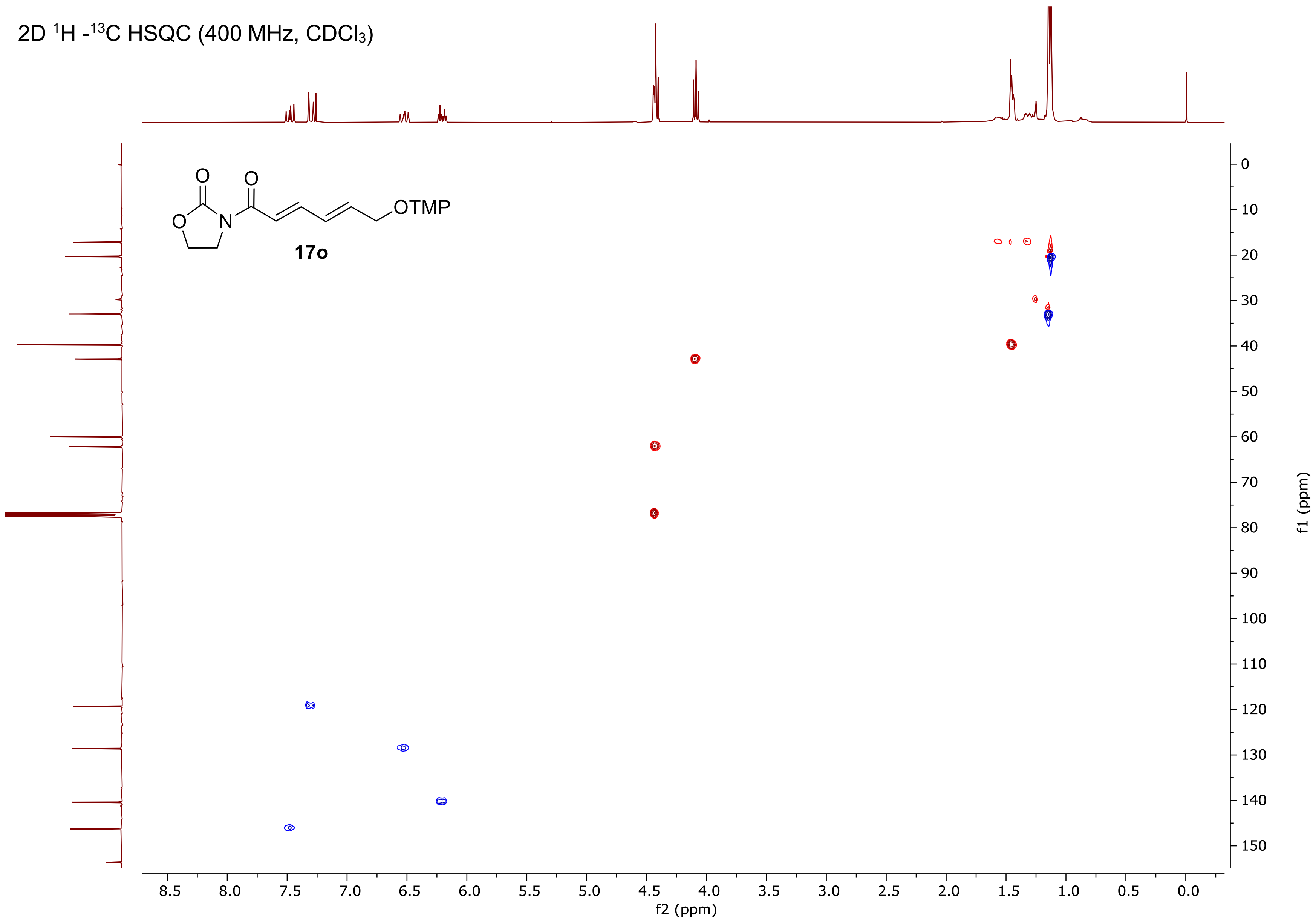

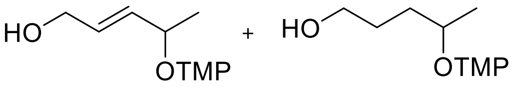


**18b** 55:45 **19b**

**18b** $^{1}$H NMR (500 MHz, $CDCl_3$)

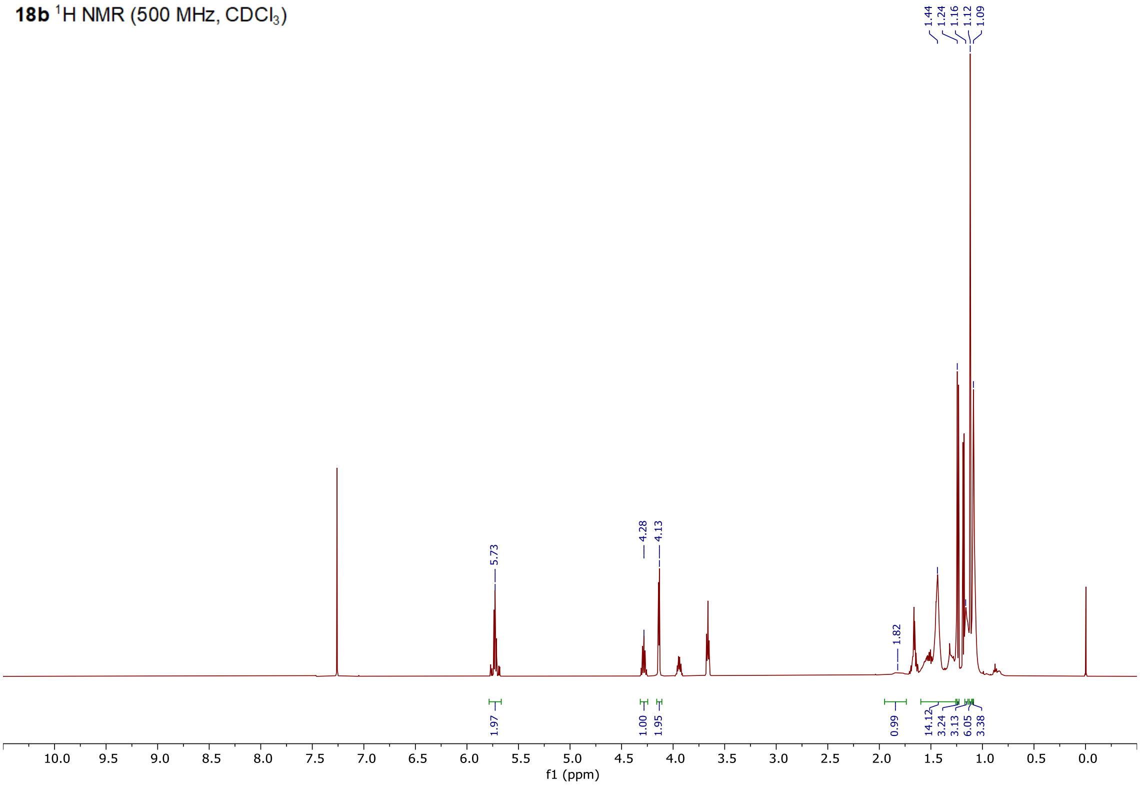


**19b** $^{1}$H NMR (500 MHz, $CDCl_3$)

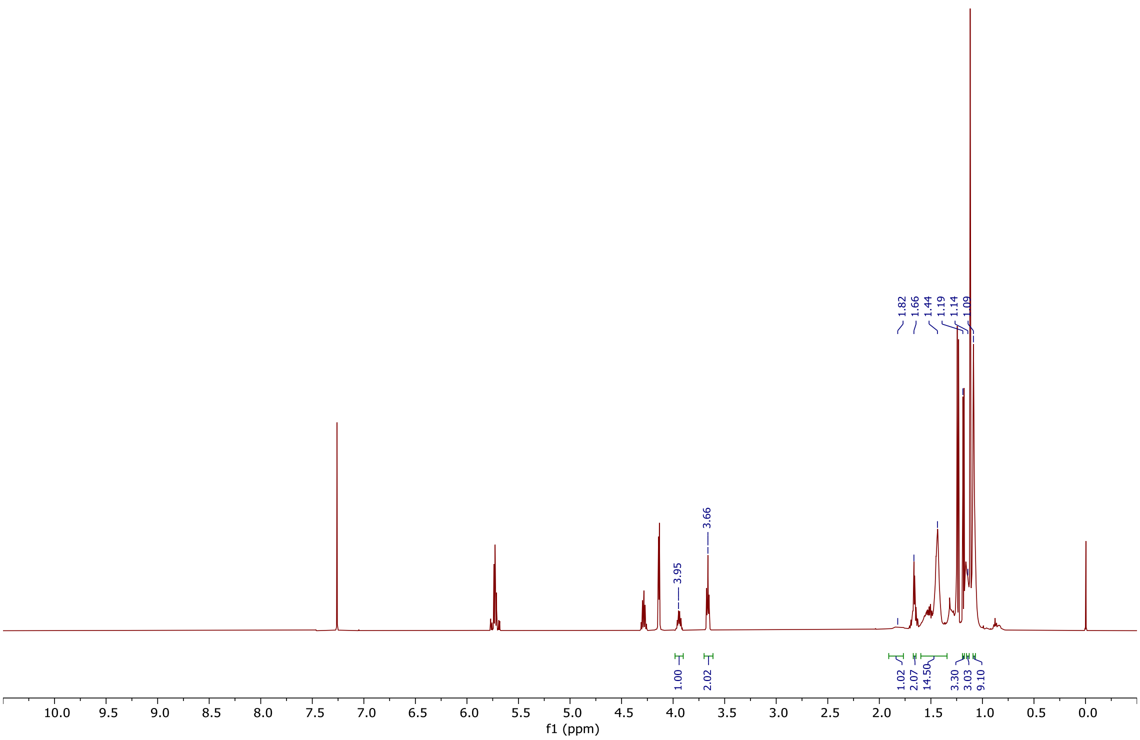

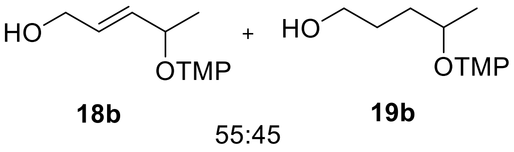

**18b** $^{13}C\{^{1}H\}$ NMR (126 MHz, $CDCl_3$)

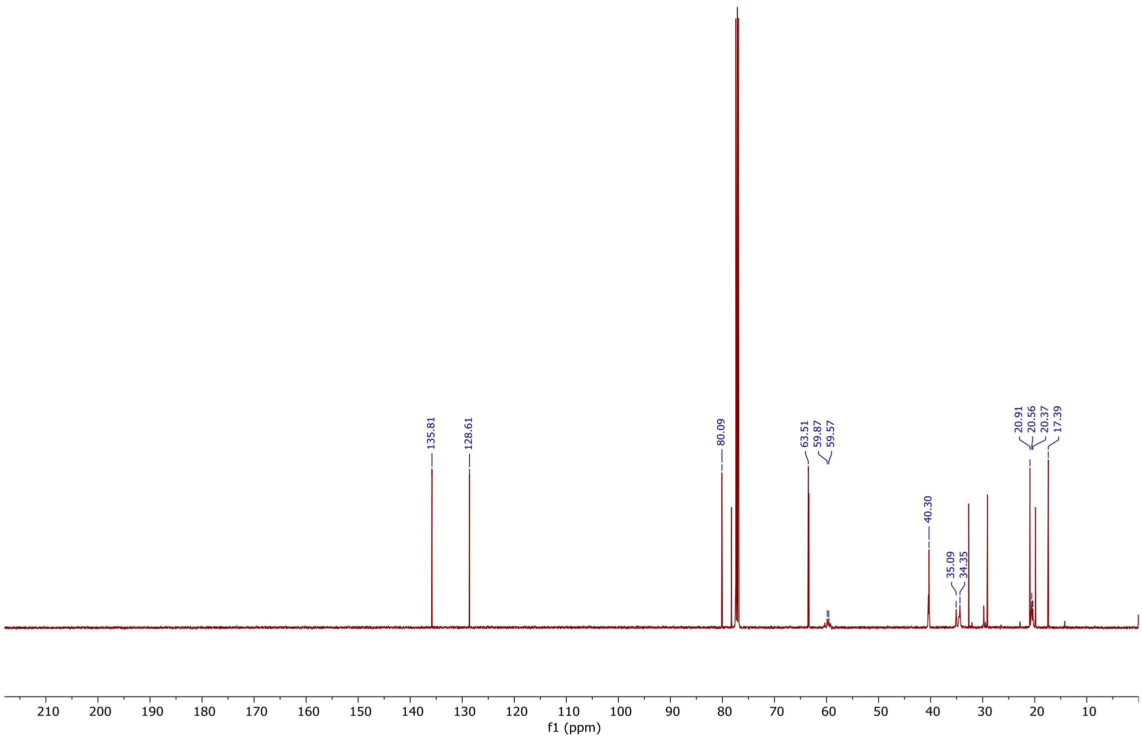


**19b** $^{13}C\{^{1}H\}$ NMR (126 MHz, $CDCl_3$)

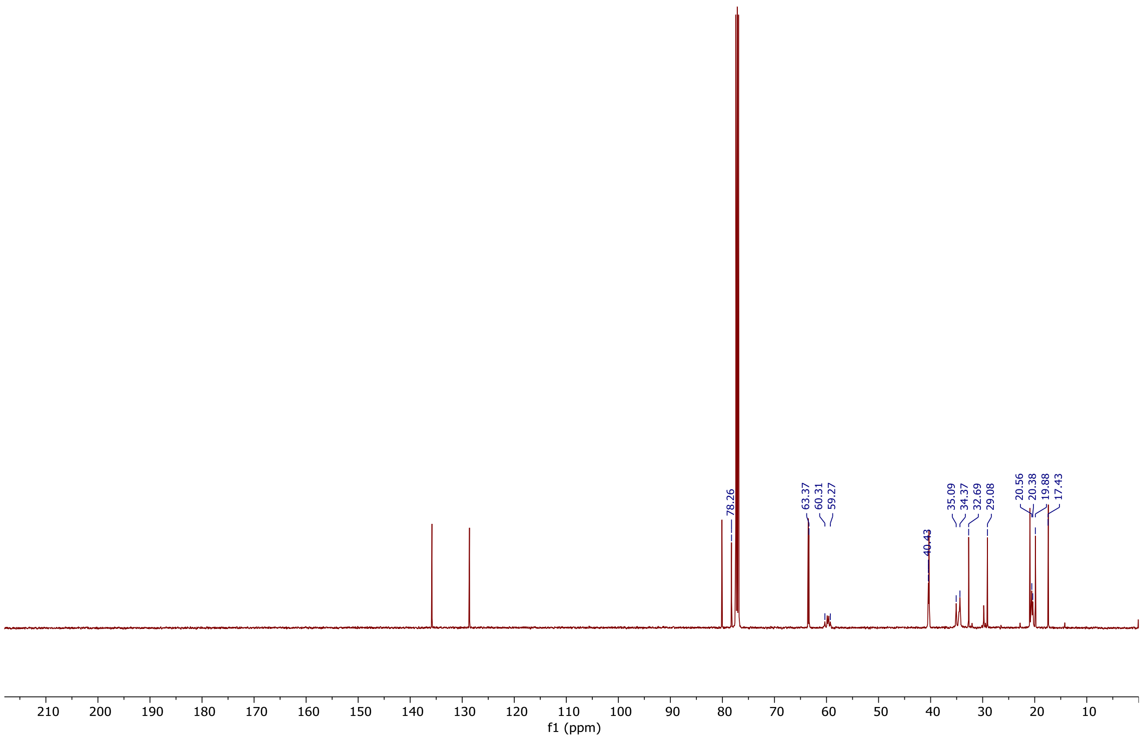

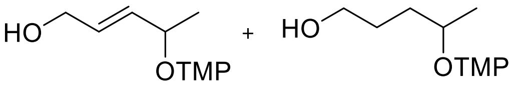


**18b** 55:45 **19b**

2D $^{1}H$ -$^{1}H$ COSY (500 MHz, $CDCl_3$)

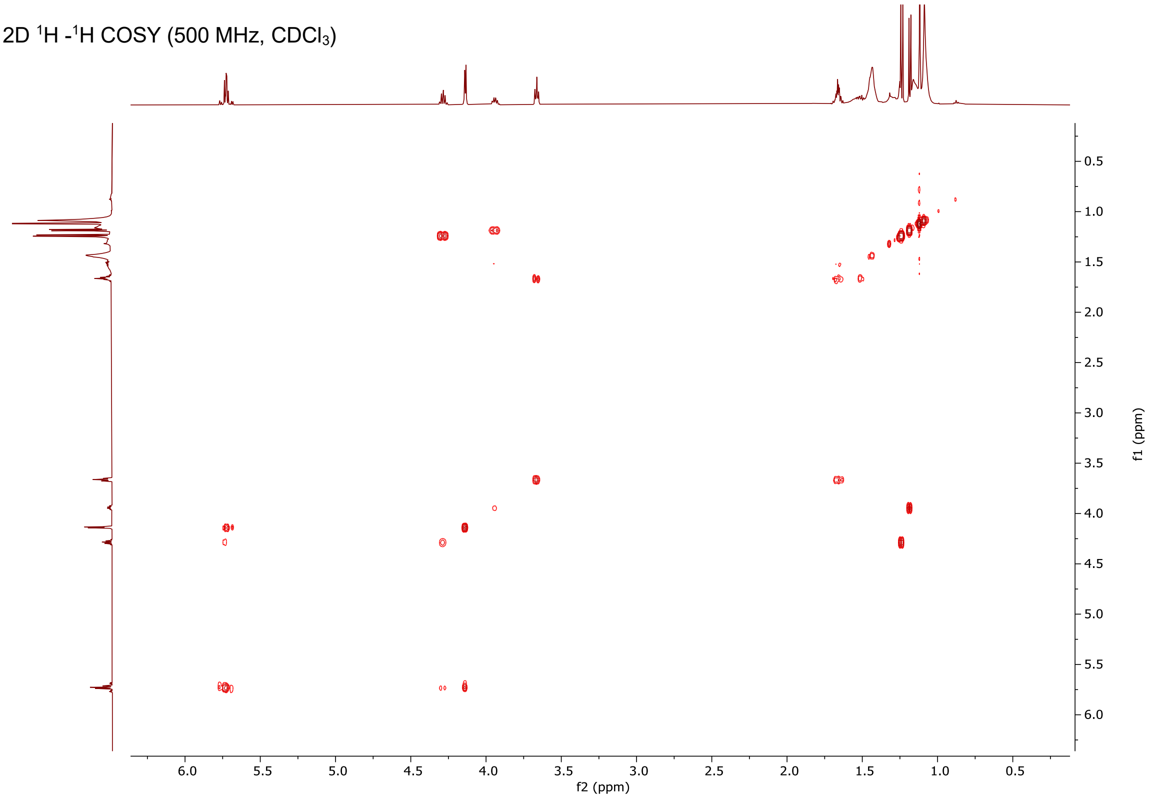


2D $^{1}H$ -$^{13}C$ HSQC (500 MHz, $CDCl_3$)

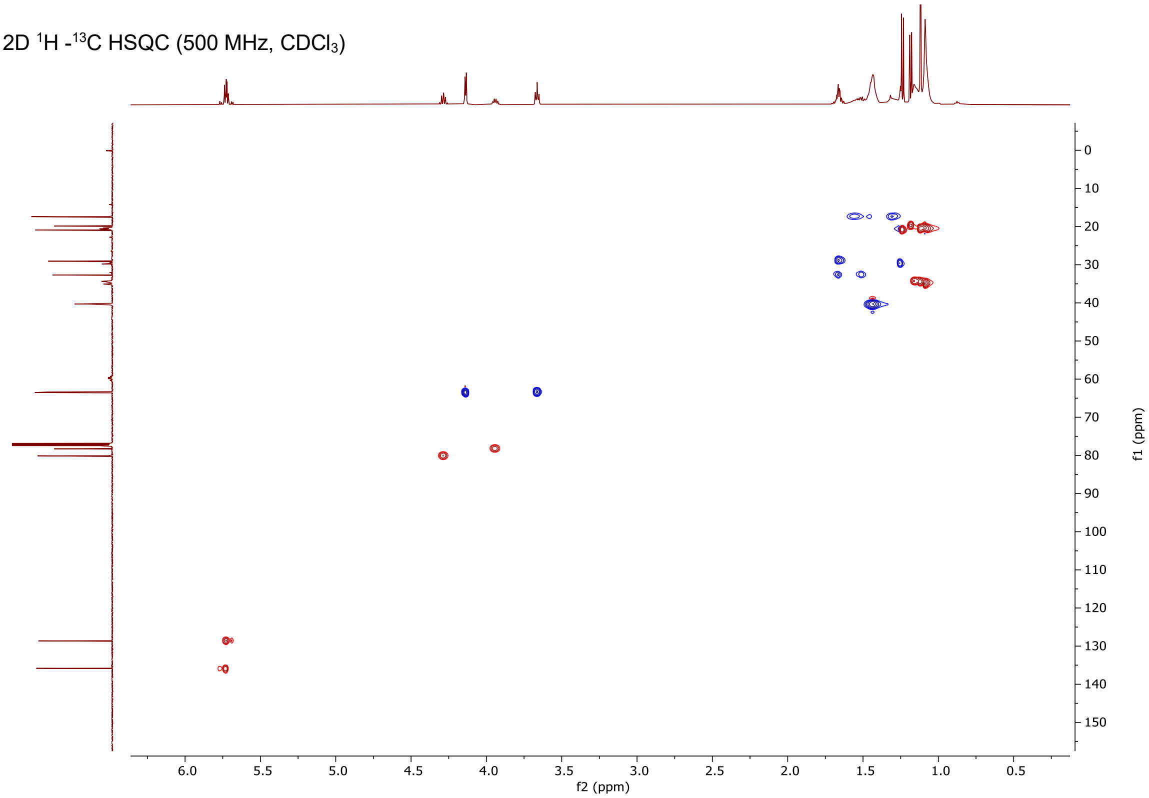

$^1H$ NMR (400 MHz, $CDCl_3$)

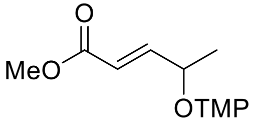


**20b**

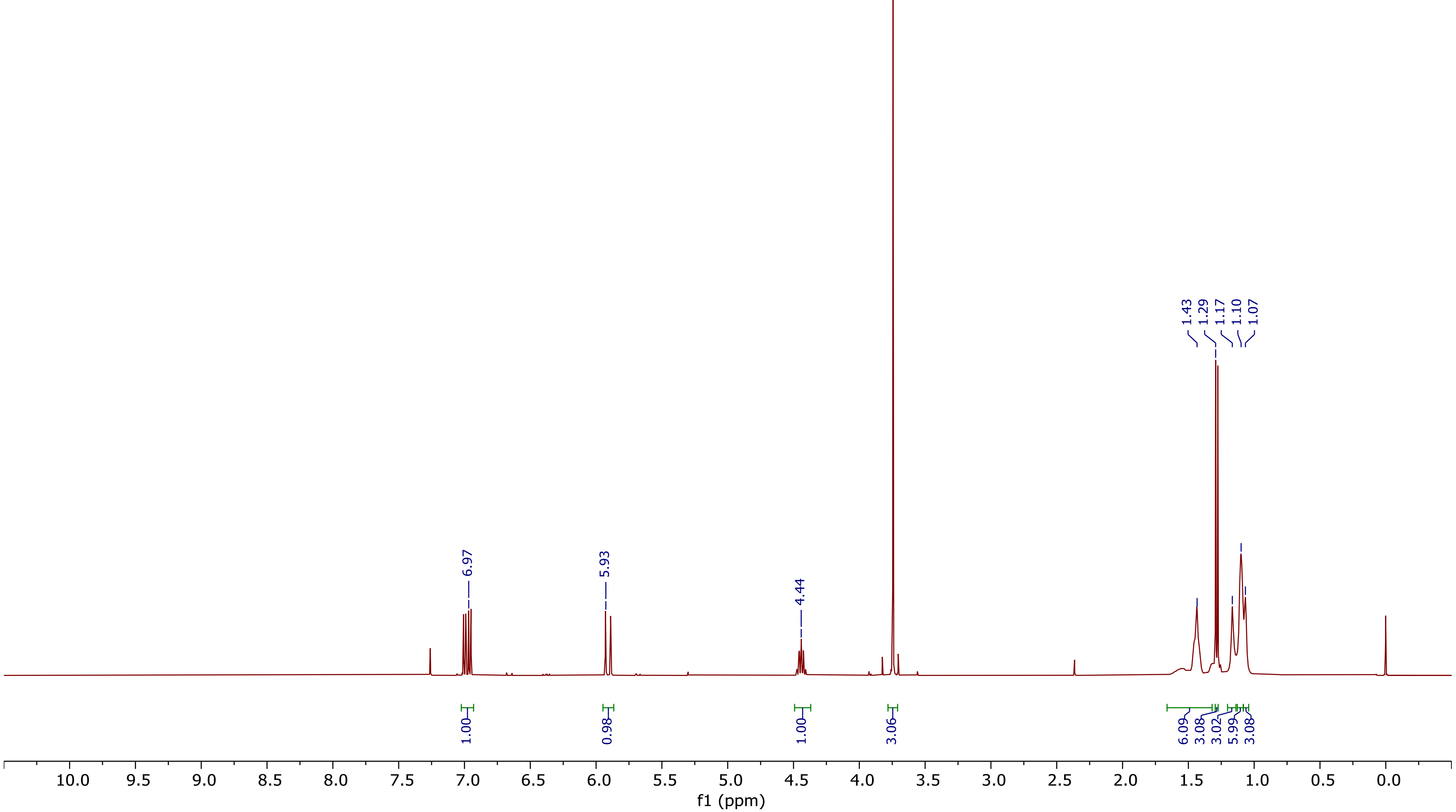

$^{13}C\{^{1}H\}$ NMR (101 MHz, $CDCl_3$)

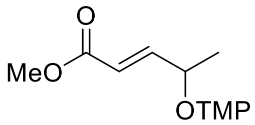


**20b**

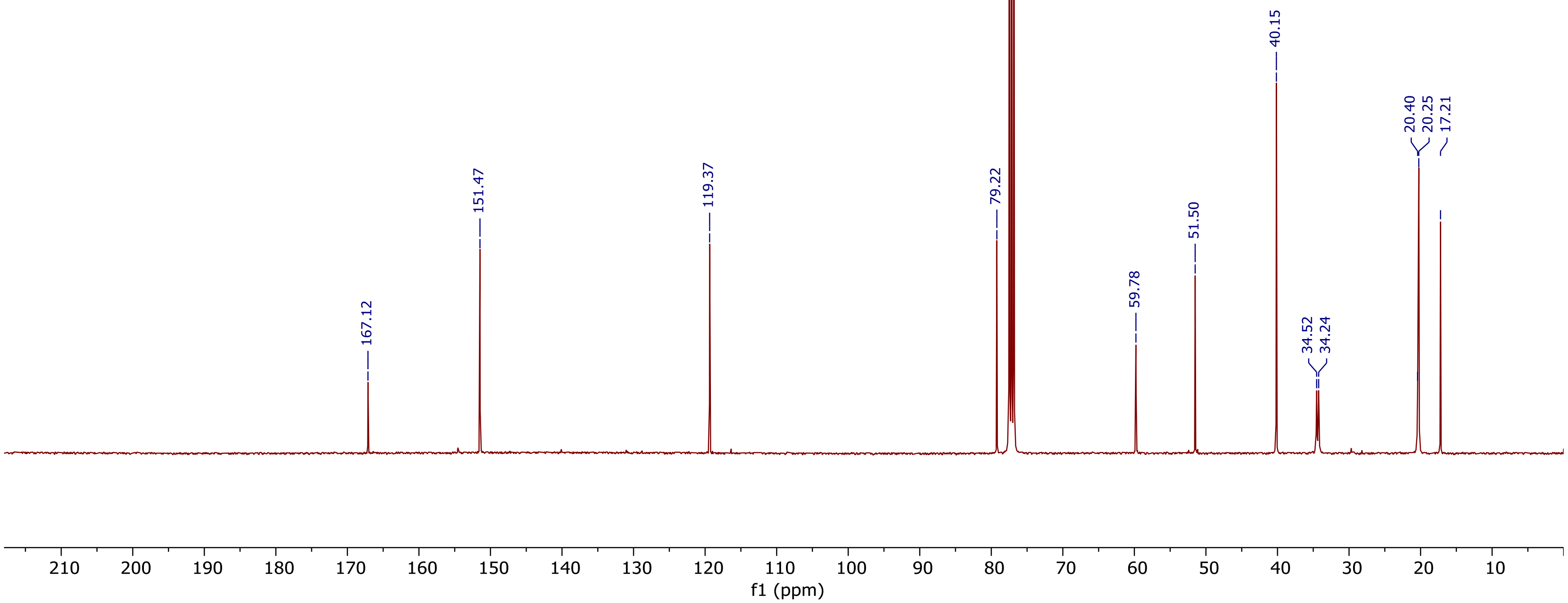

2D $^1$H -$^1$H COSY (400 MHz, $CDCl_3$)

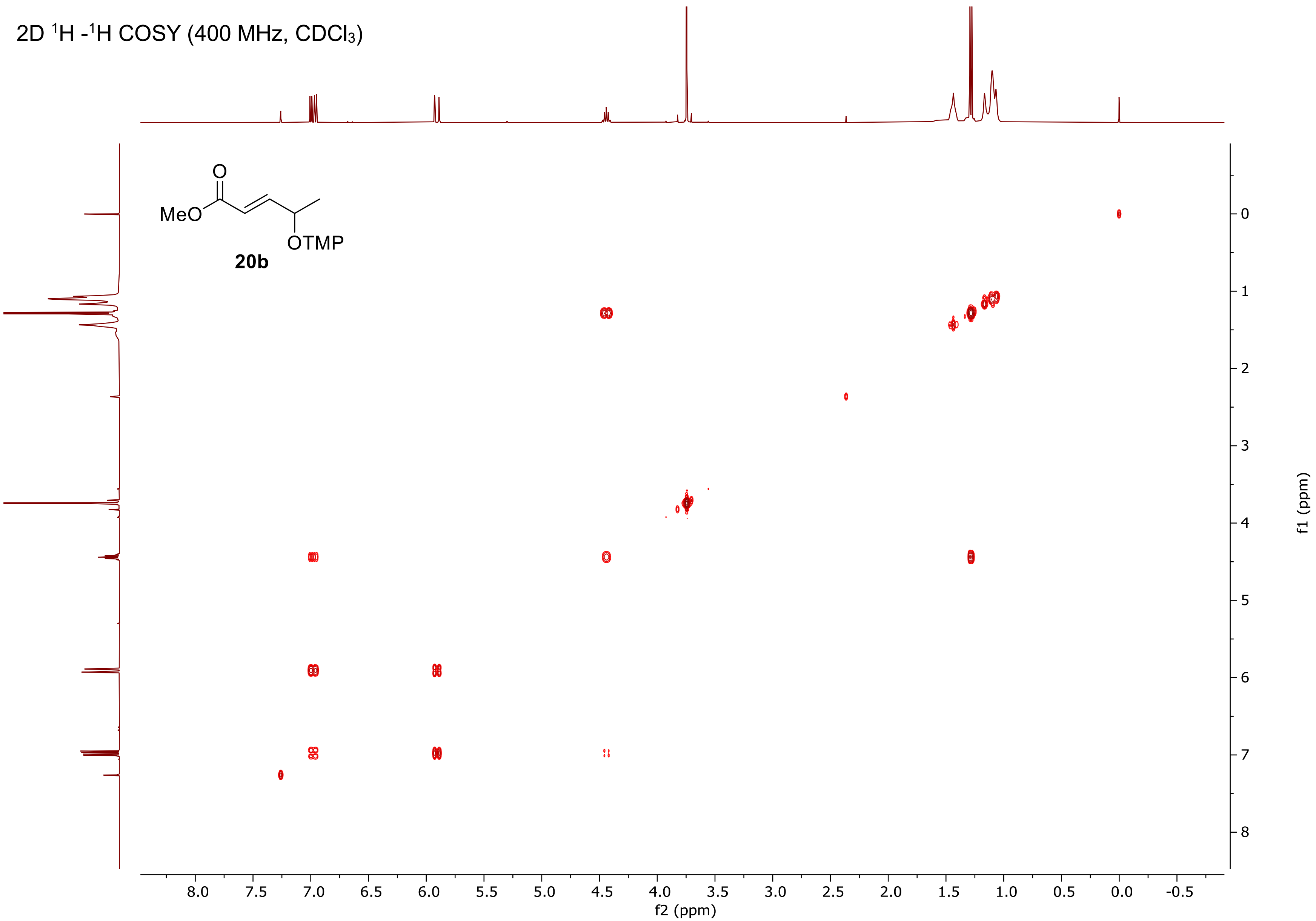

2D $^1$H -$^{13}$C HSQC (400 MHz, $CDCl_3$)

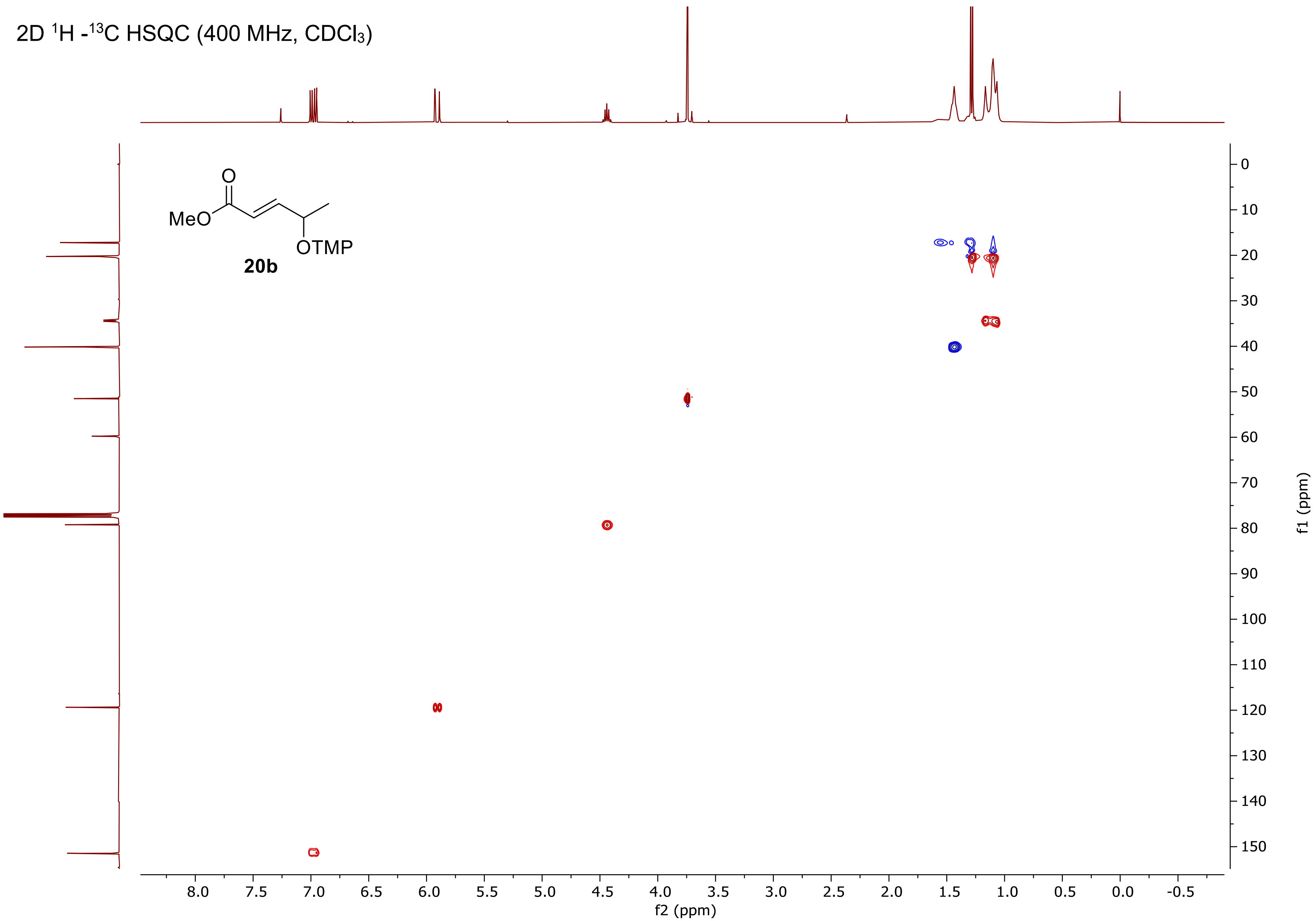

Supplement: Supplementary file 1 [file jo5c00457_si_001.pdf]
